# Photoinduced Copper-Catalyzed Late-Stage Azidoarylation of Alkenes via Arylthianthrenium Salts

**DOI:** 10.1021/jacs.3c04016

**Published:** 2023-06-12

**Authors:** Yuan Cai, Sagnik Chatterjee, Tobias Ritter

**Affiliations:** Max-Planck-Institut für Kohlenforschung, Kaiser-Wilhelm-Platz 1, D-45470 Mülheim an der Ruhr, Germany

## Abstract

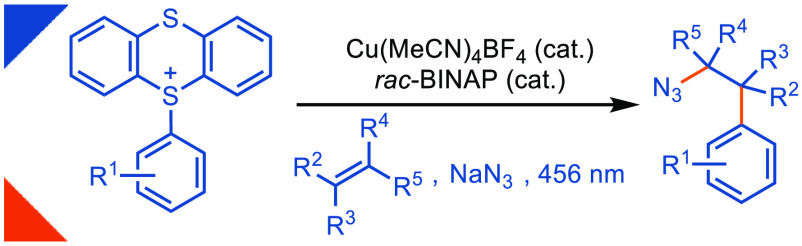

The arylethylamine pharmacophore is conserved across a range of biologically active natural products and pharmaceuticals, particularly in molecules that act on the central nervous system. Herein, we present a photoinduced copper-catalyzed azidoarylation of alkenes at a late stage with arylthianthrenium salts, allowing access to highly functionalized acyclic (hetero)arylethylamine scaffolds that are otherwise difficult to access. A mechanistic study is consistent with a *rac*-BINAP-Cu^I^-azide (**2**) as the photoactive catalytic species. We show the utility of the new method by the expedient synthesis of racemic melphalan in four steps through C–H functionalization.

Approximately 18% of the top 200 small molecule pharmaceuticals ranked by retail sales in 2021 contain an β-arylethylamine core.^1^ The widespread use as a pharmacophore has motivated extensive research into syntheses of β-arylethylamines (Figures 1A and S1).^2^ Conventional methods to prepare them often employ indirect and multistep sequences, such as homologation and reductive amination.^3^ Over the past few decades, a variety of synthetic methods have been developed,^4^ of which 1,2-aminoarylation of alkenes is a promising and straightforward approach because of the direct utilization of readily available arenes, alkenes, and amines.^5^ However, current methods generally require an intramolecular cyclization, while three-component couplings are underdeveloped; hence, general direct access to the valuable acyclic β-arylethylamine products, especially with complex arenes, is currently lacking. Herein, we report the first three-component late-stage 1,2-azidoarylation of alkenes enabled by arylthianthrenium salts. Highly functionalized acyclic β-arylethylamines can be easily accessed after the reduction of the azido groups. The combination of commercially available *rac*-BINAP and Cu(MeCN)_4_BF_4_, together with a nitrogen nucleophile, results in a standalone copper catalyst that plays a dual role as photocatalyst and group transfer catalyst, which avoids the use of additional photosensitizers.^6^ Because the thianthrenium substituent can be introduced at a late stage, complex arene frameworks are accessible that are otherwise difficult to access, and the reaction exhibits remarkable tolerance toward a diverse range of alkenes, encompassing mono-, di-, tri-, and even tetrasubstituted alkenes, as well as electron-rich and -deficient alkenes. Our work offers an approach to quickly synthesize complex β-arylethylamine products, which we demonstrate by the expedient synthesis of racemic melphalan, a chemotherapeutic agent.

**Figure 1:**
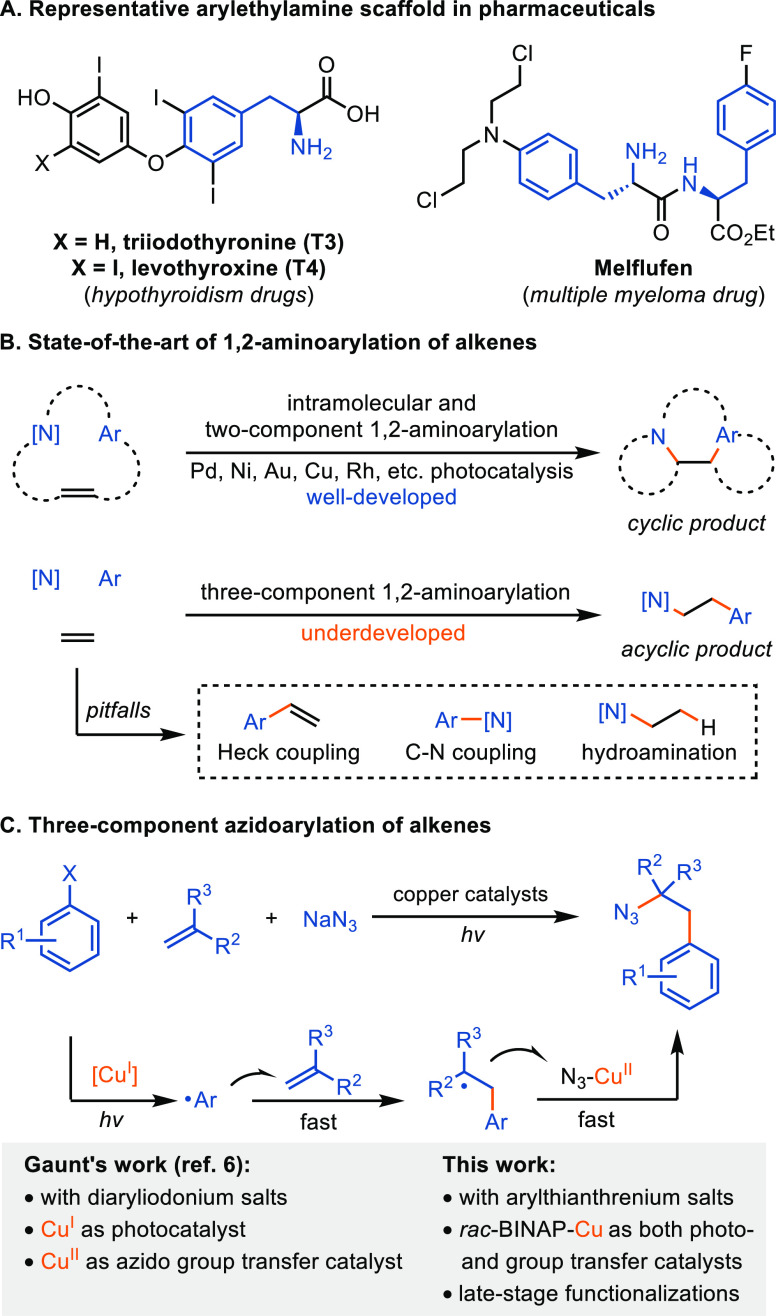
Synthesis of arylethylamines through 1,2-aminoarylation of alkenes.

1,2-Amino(hetero)arylation reactions of alkenes present a direct strategy for the synthesis of the medicinally relevant (hetero)arylethylamine unit by the simultaneous installation of amino and (hetero)aryl groups across a double bond. The synthesis of cyclic arylethylamine products by intramolecular 1,2-aminoarylation of alkenes has been accomplished when arene, amine, and alkene are mutually tethered.^7^ In addition, two-component 1,2-aminoarylations of alkenes, in which two of the three reaction partners are tethered, have been reported as well to synthesize cyclic arylethylamine products (Figure 1B, top).^8^ In some cases, cleavable linkers have been employed to obtain the synthetically useful acyclic arylethylamines upon linker cleavage.^9^ For example, the Stephenson group reported a visible-light mediated 1,2-aminoarylation of electron-rich styrenes with cleavable arylsulfonylacetamides as both aryl and amine sources to synthesize acyclic arylethylamine.^9c^ Three-component 1,2-aminoarylation of alkenes that avoid the tethering strategy are valuable because readily accessible (hetero)arene derivatives, nitrogen sources, and alkenes can be employed to obtain the desired acyclic products. However, three component coupling reactions are challenging due to facile deleterious side reactions of any two of the three reaction partners: aryl electrophiles and olefins can engage in Heck reactions, aryl electrophiles and amine sources result in C–N cross couplings, and Michael additions with electron deficient olefins and nucleophilic nitrogen make three-component couplings challenging (Figure 1B, bottom). Examples of how to address the problem via nonradical approaches include work that use a directing group strategy or rhodium-catalyzed syn-carboamidation with dioxazolones as nitrogen source.^10^ Alternatively, radical-based chemistry is promising in alkene difunctionalization.^11^ Taking advantage of the kinetically favored addition of electrophilic *N*-centered radicals to electron-rich alkenes, three-component 1,2-aminoarylation reactions of alkenes mediated by copper or nickel catalysis and Minisci reactions have been reported.^12^ Aminoarylation reactions with opposite regioselectivity were realized through aryl radical addition and subsequent carbodiazenylation, Ritter amidation, or copper-mediated azidation with aryldiazonium or -iodonium salts.^6,13^ The Gaunt group has achieved an azidoarylation reaction from diaryliodonium salts through anion-mediated dual copper catalysis, in which each copper catalyst exhibits a distinct function (Figure 1C).^6^ All reported three-component 1,2-aminoarylation reactions of alkenes to afford acyclic β-arylethylamine products have been shown with simple reagents; transformations of complex small molecule arenes have not been reported. Considering the frequent occurrence of complex aryl groups in arylethylamine-containing pharmaceuticals (Figures 1A and S1), we sought to develop the first late-stage azidoethylation reaction employing complex arylthianthrenium salts (Figure 1C).

Complex arylthianthrenium salts are readily accessible from arenes and arylboron compounds^14^ and can provide reactivity that goes beyond that of conventional aryl halides and pseudohalides.^15^ As part of our ongoing endeavors on alkene arylfunctionalizations,^16^ we explored the application of arylthianthrenium salts in the direct three-component late-stage azidoethylation. Our previously reported Meerwein bromoarylation of arylthianthrenium salts^16^ is not able to directly access the arylethylamino core, possibly due to the inefficient radical trapping of a nitrogen radical donor in the presence of the phenothiazine photocatalyst. Moreover, the Meerwein bromoarylation only proceeds with electron-poor alkenes, while in the transformation reported here, the substrate scope is not limited to a special class of alkenes. The increased substrate scope when compared to Meerwein bromoarylation and the mechanistically distinct approach to Gaunt’s azidoarylation make the azidoarylation reported here a conceptual advance to approach the significant challenge in the three-component aminoarylation of alkenes and grant expedient access to complex acyclic arylethylamine precursors.^6,16^ We propose that the conceptual novelty is enabled by identifying an appropriate copper catalyst that can both promote the generation of aryl radicals from arylthianthrenium salts and facilitate C–N bond formation through radical abstraction from a Cu(II) azide catalyst as depicted in Figure 1C. Use of a single transition-metal catalyst, such as the copper catalyst used here, both as photoredox and as redox catalyst can be beneficial over the use of combinations of independent photosensitizer and metal catalyst.^17^ To the best of our knowledge, the use of a standalone copper photocatalyst in the arylfunctionalization of alkenes is hitherto unknown.

The three-component azidoarylation via arylthianthrenium salts can be efficiently promoted using commercially available *rac*-BINAP and Cu(MeCN)_4_BF_4_ in the absence of additional photosensitizers. The combination of photosensitizers and copper sources resulted in low product yield (<26%, see Table S3), supporting the hypothesis that a standalone copper photocatalyst is important for the desired chemical reaction. Optimized reaction conditions (Table 1, entry 5) could not be extended to aryl bromides, aryl iodides, and aryldiazonium salts (entries 1–3), while a 44% yield of the desired product was observed with diphenyliodonium salts (entry 4). The copper catalysts reported by Gaunt and co-workers did not exhibit reactivity with arylthianthrenium salts in our study possibly owing to the lower reduction potential of arylthianthrenium salts when compared to diaryliodonium salts (Table S6).^6,15a^ The best yields were achieved with acrylonitrile as the radical acceptor compared with other acrylates (entries 5–8).

**Table 1:**
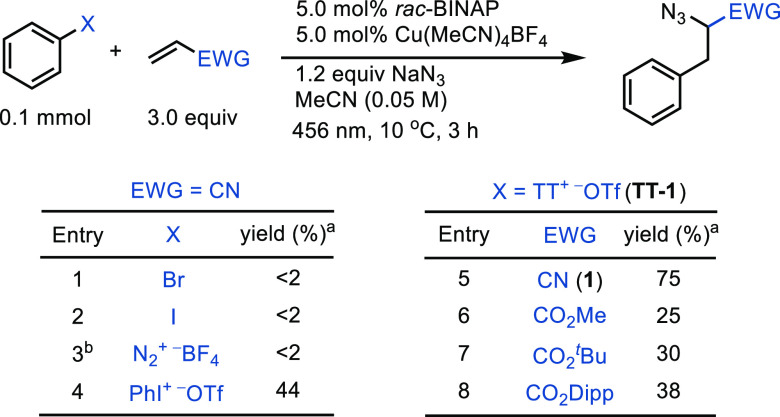
Conditions Optimization with Different Aryl Electrophiles and Radical Acceptors

a Yields were determined by ^1^H NMR with CH_2_Br_2_ as internal standard.

b With or without light. EWG, electron withdrawing group; *rac*-BINAP, racemic 2,2′-bis(diphenylphosphino)-1,1′-binaphthalene; Dipp, 2,6-diisopropylphenyl.

The use of visible-light excitation of Cu(I) has been established as an effective means of harvesting photon energy and promoting bond formation.^17,18^ Building upon this concept, we conducted a series of mechanism experiments to explore the possibility of a role similar to that of the copper catalyst in our research. First, the UV–vis absorption spectroscopy of the combination of *rac*-BINAP, Cu(MeCN)_4_BF_4_, and NaN_3_ in MeCN indicated the formation of copper complex *rac-*BINAPCu^I^N_3_ (**2)** in the reaction mixture (see Figure 2A). The identity of complex **2** was confirmed through X-ray crystal structure analysis (Figure 3). The absorption spectrum of complex **2**, which extends up to 550 nm, overlaps with the emission spectrum of blue LEDs (Figure 2A). Complex **2** is catalytically competent in the azidoarylation reaction, producing desired product **1** in 71% yield (Figure 2B). The luminescence of excited 2* was effectively quenched by arylthianthrenium salts, supporting the role of **2** as a photocatalyst (see Supporting Information, Figure S8). Furthermore, the formation of **1** upon heating the mixture of copper complex **3**, benzoyl peroxide (BPO), and acrylonitrile in MeCN provided evidence that copper complex **3** is competent for the azido group transfer step (Figure 2C). A radical clock experiment employing a cyclopropyl-substituted olefin yielded a mixture of rearranged and unopened products in a ratio of 5.2:1 (see Supporting Information, Figure S10), suggesting that the rate of capture of the homobenzyl radical by the copper-azide species is in the same order of magnitude as the ring-opening process, which occurs with a first-order rate constant of approximately 5 × 10^7^ s^–1^.^19^ Based on these results, we propose the operative mechanism shown in Figure 2D, wherein the combination of *rac*-BINAP, Cu(MeCN)_4_BF_4_, and NaN_3_ in MeCN produces the photoactive copper catalyst **2** in situ. Upon excitation, **2** engages in single-electron reduction of the arylthianthrenium salt to afford, after mesolytic cleavage, an aryl radical and copper(II) complex **3** or its cationic counterpart with only one azide coordinated. The addition of the aryl radical to acrylonitrile generates a homobenzyl radical, which subsequently attacks the terminal nitrogen atom of the azido group in complex **3** via an outer-sphere pathway to afford the desired product and regenerate catalyst **2**.^20^ This C–N_3_ bond formation pathway renders potential enantioselective azidoarylation possible using nonracemic BINAP-type ligands. However, we only observed less than 8% ee of the product, possibly due to the reaction site being too far away for efficient chiral induction (see Supporting Information, Table S12).^21^

**Figure 2:**
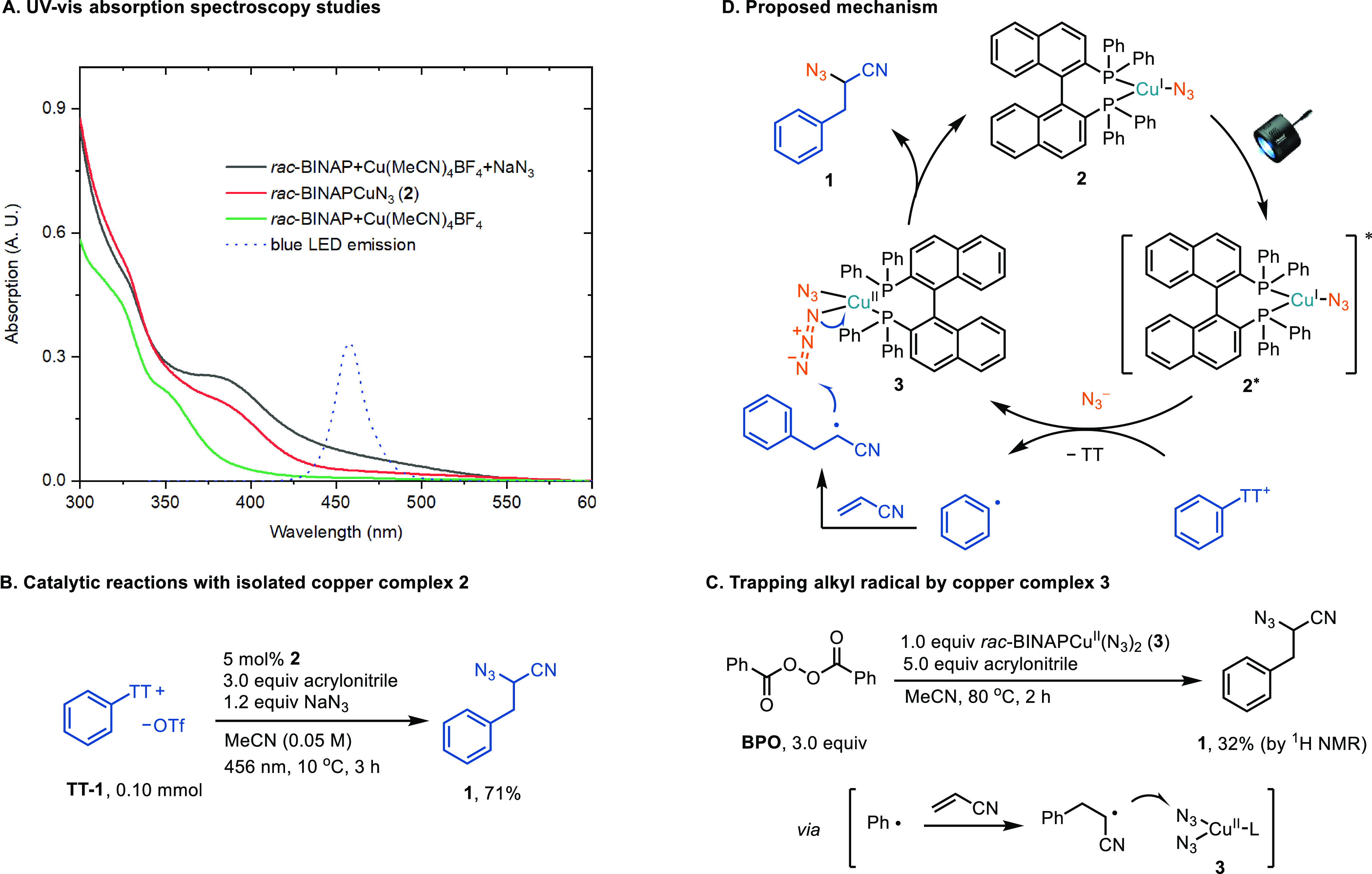
Mechanistic investigation and proposed mechanism.

**Figure 3:**
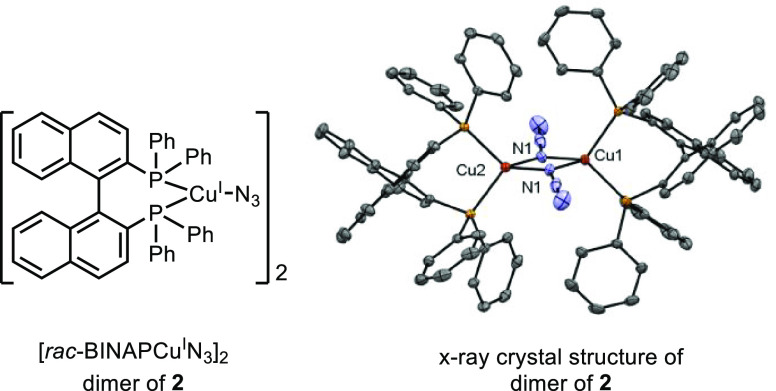
X-ray crystal structure of **2** (thermal ellipsoids drawn at 50% probability; hydrogens omitted for clarity). Selected bond distances (Å) and angles (deg): Cu(1)–N(1) 2.082; Cu(2)–N(1) 2.098; N(1)–Cu(1)–N(1) 86.31; Cu(1)–N(1)–Cu(2) 94.09.

The copper-catalyzed azidoarylation tolerates a variety of complex arylthianthrenium salts (Scheme 1, **4**–**18**). Highly functionalized acyclic (hetero)arylethylamines precursors were obtained as a single constitutional isomer in 40–71% yield, enabling straightforward purification. The substituents on the aryl group of the products can be electron-rich, -neutral (**12**), or -poor (**18**), and para-, meta- (**23**), or ortho-substitution (**24**) patterns are all within the scope of the reaction. Importantly, installation of azidoethyl groups at a late stage after converting arenes via C–H thianthrenation, as well as phenols (**10**, **13**) and aryl chlorides (**8**, **14**, **18**) to arylthianthrenium salts via arylboron compounds, is feasible as opposed to prior art. When acrylonitrile is used as the aryl radical acceptor, highly functionalized unnatural (hetero)aromatic amino acid derivatives, a significant subset of arylethylamines, are obtained.^22^ Numerous functional groups, including alkyl chlorides, aryl halides (F, Cl, Br), cyclopropyl, trifluoromethyl, ethers, ketones, esters, amides, sulfonyl, sulfonamides, nitriles, nitro groups, phosphonate, as well as protic groups, such as secondary amines and amides, are tolerated. Heteroarylthianthrenium salts are stable and readily synthesized from heteroarenes (**7**, **15**) or heteroarylboron compounds (**8**, **25**–**29**). Several heteroaromatics, including pyridine, benzofuran, quinoline, isoquinoline, thianaphthene, and pyrrole are well tolerated, despite the potential for undesired Minisci reactions. The products obtained are highly functionalized acyclic (hetero)arylethylamine precursors, which are important pharmacophores in drug discovery.

**Scheme 1:**
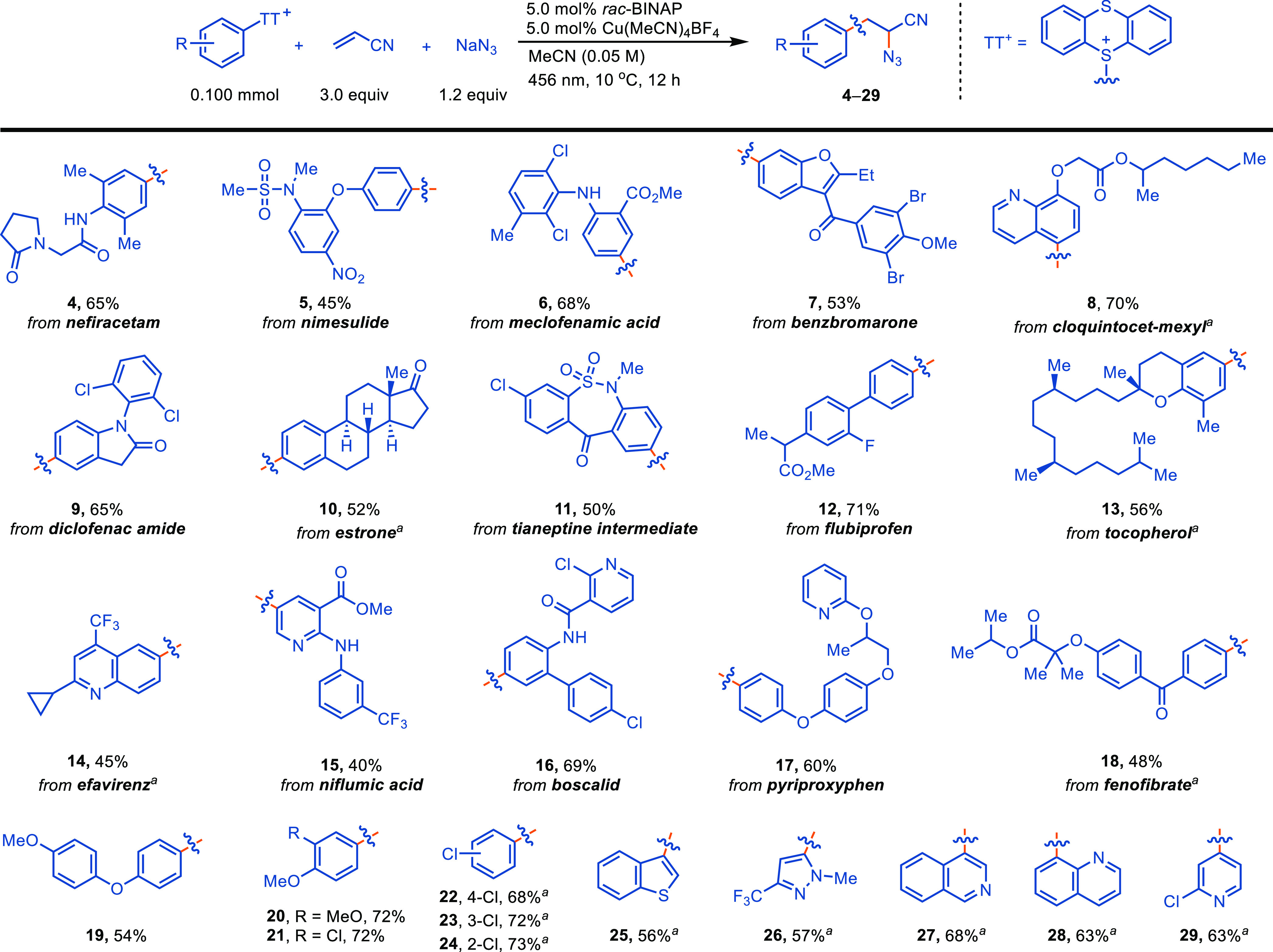
Scope of Arylthianthrenium Salts The corresponding (hetero)arylthianthrenium salts were synthesized from (hetero)arylbronic acids or esters.

A broad scope of alkenes is tolerated, including different substituted Michael acceptors (**30**–**34**, **50**, **51**), styrenes (**36**–**41**), and unactivated alkenes (**41**–**48**), with both electron-rich and electron-poor alkenes being compatible (Scheme 2). When electron-rich alkenes were used, the electrophilic pyridyl radical produced higher yields (**42**–**48**, 52–68%) than nucleophilic aryl radicals (20–30%), likely attributed to the polar match between electron-rich alkenes and the more electrophilic pyridyl radical.^23^ Remarkably, all kinds of substituted alkenes were compatible, including mono-, 1,1-di-, 1,2-di-, trisubstituted (**46**), and tetrasubstituted (**47**), which is rare for alkene difunctionalization reactions. The 1,2-azidoarylation addition of tetramethylethene produced two adjacent quaternary centers, which is generally challenging to access. We could also access different kinds of (hetero)arylethylamine precursors that are difficult to obtain through nucleophilic substitution reactions of corresponding alkyl halides, such as α-tertiary and α-alkoxy amines (**45**), as well as amines attached through a bridged ring (**35**). Our previously reported bromoarylation reaction mediated by a phenothiazine photocatalyst was not effective with electron-rich styrenes, 1,1-dialkyl- and trisubstituted alkenes, and vinyl ether, possible due to the highly oxidizing phenothiazine radical cation (*E*^⊖^ = 0.90 V vs SCE) to cause single electron oxidation of alkyl radicals to carbon cations and subsequent elimination.^16^ The oxidation potential of complex **3** is relatively small [*E*_1/2_(Cu^II^/Cu^I^) = 0.23 V vs Ag/AgCl], which may explain the broad substrate scope of alkenes accessible in the reaction. Generally, 3.0 equiv of alkenes was used to balance hydrodefunctionalization and oligmerization, which account for the main byproducts (Table S9). Yet, use of alkene as the limiting reagent is also possible when 1.5 equiv of arylthianthrenium salt is employed, which provides the opportunity for late-stage functionalization of complex alkenes (**48**, **51**, and **52**).

**Scheme 2:**
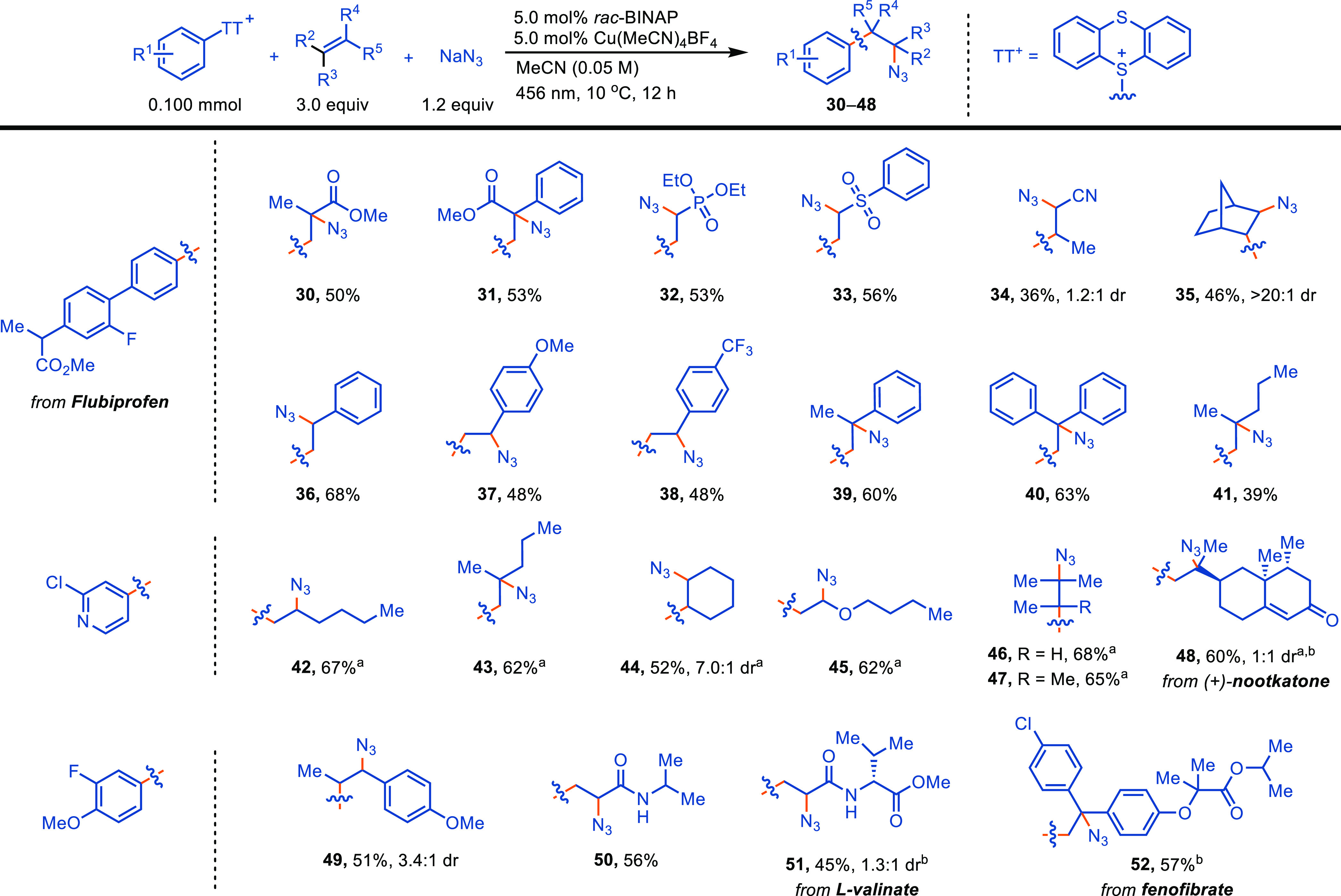
Scope of Alkenes (a) TFE was used as the solvent; (b) 1.0 equiv of alkene and 1.5 equiv of arylthianthrenium salts were used.

To show the synthetic utility of the method, we successfully synthesized racemic melphalan (**56**) from commercially available *N*,*N*-bis(2-chloroethyl)aniline (**53**) using a four-step process including thianthrenation, azidoarylation, azido group reduction, and nitrile hydrolysis (Scheme 3). This approach shows the ability for quick diversifications, unlike previous methods that require stepwise installation of the nitrogen mustard chloroethylamino groups from phenylalanines and the protection/deprotection steps of amino acids.^24^ Additionally, we demonstrate the potential synthesis of several important compounds, including thyronine (**19**) from 4-methoxydiphenyl ether, dopa (**20**) from veratrole, xylariamide A (**21**) from 2-chloroanisole, and fenclonine (**22**) from 4-chlorophenylboronic acid.

**Scheme 3:**
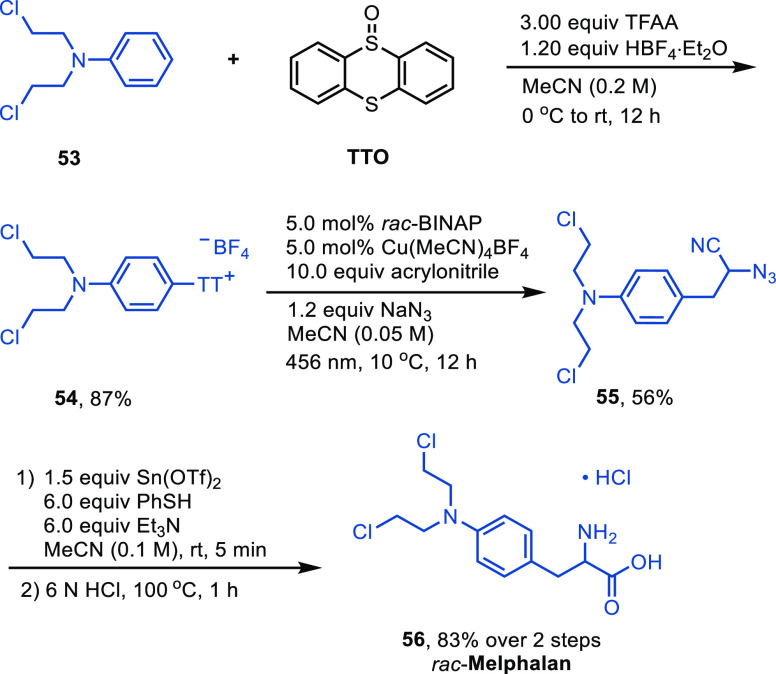
Synthesis of Racemic Melphalan from *N,N*-Bis(2-chloroethyl)aniline

In conclusion, we described an azidoarylation approach to medicinally relevant acyclic (hetero)arylethylamines. Keys to the success are the utilization of an in situ generated *rac*-BINAP-Cu^I^-azide (**2**) as catalyst and readily accessible complex arylthianthrenium salts as aryl radical precursor. Our research also enables medicinal chemists to rapidly access complex arylethylamines due to robust late-stage C–H thianthrenation. Further studies could focus on alkene aminoarylation reactions with other amines, apart from azide and enantioselective reactions.

## Supporting Information Available

Experimental procedures, spectroscopic data, and NMR spectra of all products (PDF)
