# Supplementary material for: Photoinduced Copper-Catalyzed Late-Stage Azidoarylation of Alkenes via Arylthianthrenium Salts
Source: J Am Chem Soc. 2023 Jun 12;145(25):13542–8. doi: 10.1021/jacs.3c04016 (PMC10311530; doi:10.1021/jacs.3c04016)
Supplement: Supplementary file 1 — ja3c04016_si_001.pdf [file ja3c04016_si_001.pdf]

SUPPORTING INFORMATION

# Photoinduced Copper-catalyzed Late-stage Azidoarylation of Alkenes via Arylthianthrenium Salts

Yuan Cai, Sagnik Chatterjee, and Tobias Ritter*

Max-Planck-Institut für Kohlenforschung, Kaiser-Wilhelm-Platz 1, D-45470 Mülheim an der Ruhr, Germany

*E-mail: ritter@kofo.mpg.de

# TABLE OF CONTENTS

TABLE OF CONTENTS ........ 1

MATERIALS AND METHODS ........ 10

EXPERIMENTAL DATA ........ 11

Selected complex pharmaceuticals containing arylethylamine fragment ........ 11

Reaction condition optimization ........ 12

Scope of azidoarylation of alkenes with arylthianthrenium salts ........ 18

General procedure for azidoarylation of alkenes with arylthianthrenium salts ........ 18

*rac*-Nefiracetam-derived phenylalanine analogue **4** ........ 19

*rac*-Nimesulide-derived phenylalanine analogue **5** ........ 19

*rac*-Meclofenamic acid-derived phenylalanine analogue **6** ........ 20

*rac*-Benzbromarone-derived phenylalanine analogue **7** ........ 21

Cloquintocet-mexyl-derived phenylalanine analogue **8** ........ 21

*rac*-Diclofenac amide-derived phenylalanine analogue **9** ........ 22

Estrone-derived phenylalanine analogue **10** ........ 23

*rac*-Tianeptine intermediate-derived phenylalanine analogue **11** ........ 24

Flubiprofen-derived phenylalanine analogue **12** ........ 24

Tocopherol-derived phenylalanine analogue **13** ........ 25

*rac*-Quinoline-derived phenylalanine analogue **14** ........ 26

*rac*-Niflumic acid-derived phenylalanine analogue **15** ........ 27

*rac*-Boscalid-derived phenylalanine analogue **16** ........ 27

Pyriproxyphen-derived phenylalanine analogue **17** ........ 28

*rac*-Fenofibrate-derived phenylalanine analogue **18** ........ 29

*rac*-Thyronine precursor **19** ........ 30

*rac*-Dopa precursor **20** ........ 30

*rac*-Xylariamide A precursor **21** ........ 31

*rac*-Fenclonine precursor **22** ........ 32

*rac*-Phenylalanine analogue **23** ........ 32

*rac*-Phenylalanine analogue **24** ........ 33

*rac*-Thianaphthene alanine analogue **25** ........ 33

*rac*-Pyrrole alanine analogue **26** ........ 34

*rac*-Isoquinoline alanine analogue **27** ........ 35

*rac*-Quinoline alanine analogue **28** ........ 35

*rac*-Pyridine alanine analogue **29** ........ 36

Flubiprofen-derived phenylethylazide **30** ........ 37

Flubiprofen-derived phenylethylazide **31** ........ 38

Flubiprofen-derived phenylethylazide **32** ........ 38
Flubiprofen-derived phenylethylazide **33** ........ 39
Flubiprofen-derived phenylethylazide **34** ........ 40
Flubiprofen-derived phenylethylazide **35** ........ 41
Flubiprofen-derived phenylethylazide **36** ........ 42
Flubiprofen-derived phenylethylazide **37** ........ 42
Flubiprofen-derived phenylethylazide **38** ........ 43
Flubiprofen-derived phenylethylazide **39** ........ 44
*rac*-Flubiprofen-derived phenylethylazide **40** ........ 45
Flubiprofen-derived phenylethylazide **41** ........ 46
*rac*-Pyridinylethylazide **42** ........ 46
*rac*-Pyridinylethylazide **43** ........ 47
Pyridinylethylazide **44** ........ 48
*rac*-Pyridinylethylazide **45** ........ 48
*rac*-Pyridinylethylazide **46** ........ 49
Pyridinylethylazide **47** ........ 50
Nootkatone-derived pyridinylethylazide **48** ........ 50
Phenylethylazide **49** ........ 51
*rac*-Phenylethylazide **50** ........ 52
Valinate-derived phenylethylazide **51** ........ 53
*rac*-Fenofibrate-derived phenylethylazide **52** ........ 53

Unsuccessful examples ........ 55

Synthesis of racemic melphalan ........ 56

Synthesis of arylthianthrenium salts ........ 58

Nimesulide-derived thianthrenium salt TT-**5** ........ 58
Meclofenamic acid-derived thianthrenium salt TT-**6** ........ 59
Benzbromarone-derived thianthrenium salt TT-**7** ........ 59
Estrone-derived thianthrenium salt TT-**10** ........ 60
Tianeptine intermediate-derived thianthrenium salt TT-**11** ........ 61
Quinoline-derived thianthrenium salt TT-**14** ........ 62
4-Methoxydiphenyl ether-derived thianthrenium salt TT-**19** ........ 63
2-Chloroanisole-derived thianthrenium salt TT-**21** ........ 64
Thianaphthene-derived thianthrenium salt TT-**25** ........ 64
Pyrazol-derived thianthrenium salt TT-**26** ........ 65
Isoquinoline-derived thianthrenium salt TT-**27** ........ 66
Quinoline-derived thianthrenium salt TT-**28** ........ 67
Pyridine-derived thianthrenium salt TT-**29** ........ 67

Synthesis of copper complexes....68

Synthesis of *rac*-BINAPCu(MeCN)$BF_4$ ....68
Synthesis of *rac*-BINAPCu$N_3$ (**2**)....69
Synthesis of *rac*-BINAPCu$(N_3)_2$ (**3**)....69

Mechanistic studies....70

UV-vis absorption spectroscopy studies....70
UV–vis absorption spectra upon irradiation of *rac*-BINAPCu$(N_3)_2$ (**3**)....71
Cyclic voltammograms....71
Stern-volmer luminescence quenching studies....72
Azidoarylation of alkenes catalyzed by copper complexes....73
Trapping alkyl radical by *rac*-BINAPCu$(N_3)_2$ (**3**)....74
Radical clock experiment....75

X-RAY CRYSTALLOGRAPHIC ANALYSIS....77

X-Ray Chrystallographic Data of *rac*-BINAPCu$N_3$ (**2**) (CCDC 2247825)....77

Experimental....77
Table S14. Crystal data and structure refinement.....77
Table S15. Bond lengths [Å] and angles [°].....79

SPECTROSCOPIC DATA....82

$^1$H NMR of *rac*-nefiracetam-derived phenylalanine analogue **4**....82

$^{13}$C NMR of *rac*-nefiracetam-derived phenylalanine analogue **4**....83

$^1$H NMR of *rac*-nimesulide-derived phenylalanine analogue **5**....84

$^{13}$C NMR of *rac*-nimesulide-derived phenylalanine analogue **5**....85

$^1$H NMR of *rac*-meclofenamic acid-derived phenylalanine analogue **6**....86

$^{13}$C NMR of *rac*-meclofenamic acid-derived phenylalanine analogue **6**....87

$^1$H NMR of *rac*-benzbromarone-derived phenylalanine analogue **7**....88

$^{13}$C NMR of *rac*-benzbromarone-derived phenylalanine analogue **7**....89

$^1$H NMR of cloquintocet-mexyl-derived phenylalanine analogue **8**....90

$^{13}$C NMR of cloquintocet-mexyl-derived phenylalanine analogue **8**....91

$^1$H NMR of *rac*-diclofenac amide-derived phenylalanine analogue **9**....92

$^{13}$C NMR of *rac*-diclofenac amide-derived phenylalanine analogue **9**....93

$^1$H NMR of estrone-derived phenylalanine analogue **10**....94

$^{13}$C NMR of estrone-derived phenylalanine analogue **10** ........ 95

$^{1}$H NMR *rac*-tianeptine intermediate-derived phenylalanine analogue **11** ........ 96

$^{13}$C NMR of *rac*-tianeptine intermediate-derived phenylalanine analogue **11** ........ 97

$^{1}$H NMR of flubiprofen-derived phenylalanine analogue **12** ........ 98

$^{19}$F NMR of flubiprofen-derived phenylalanine analogue **12** ........ 99

$^{13}$C NMR of flubiprofen-derived phenylalanine analogue **12** ........ 100

$^{1}$H NMR of tocopherol-derived phenylalanine analogue **13** ........ 101

$^{13}$C NMR of tocopherol-derived phenylalanine analogue **13** ........ 102

$^{1}$H NMR of *rac*-quinoline-derived phenylalanine analogue **14** ........ 103

$^{19}$F NMR of *rac*-quinoline-derived phenylalanine analogue **14** ........ 104

$^{13}$C NMR of *rac*-quinoline-derived phenylalanine analogue **14** ........ 105

$^{1}$H NMR of *rac*-niflumic acid-derived phenylalanine analogue **15** ........ 106

$^{19}$F NMR of *rac*-niflumic acid-derived phenylalanine analogue **15** ........ 107

$^{13}$C NMR of *rac*-niflumic acid-derived phenylalanine analogue **15** ........ 108

$^{1}$H NMR of *rac*-boscalid-derived phenylalanine analogue **16** ........ 109

$^{13}$C NMR of *rac*-boscalid-derived phenylalanine analogue **16** ........ 110

$^{1}$H NMR of pyriproxyphen-derived phenylalanine analogue **17** ........ 111

$^{13}$C NMR of pyriproxyphen-derived phenylalanine analogue **17** ........ 112

$^{1}$H NMR of *rac*-fenofibrate-derived phenylalanine analogue **18** ........ 113

$^{13}$C NMR of *rac*-fenofibrate-derived phenylalanine analogue **18** ........ 114

$^{1}$H NMR of *rac*-thyronine precursor **19** ........ 115

$^{13}$C NMR of *rac*-thyronine precursor **19** ........ 116

$^{1}$H NMR of *rac*-dopa precursor **20** ........ 117

$^{13}$C NMR of *rac*-dopa precursor **20** ........ 118

$^{1}$H NMR of *rac*-xylariamide A precursor **21** ........ 119

$^{13}$C NMR of *rac*-xylariamide A precursor **21** ........ 120

$^{1}$H NMR of *rac*-fenclonine precursor **22** ........ 121

$^{13}$C NMR of *rac*-fenclonine precursor **22** ........ 122

$^{1}$H NMR of *rac*-phenylalanine analogue **23** ........ 123

$^{13}$C NMR of *rac*-phenylalanine analogue **23** ........ 124

$^{1}$H NMR of *rac*-phenylalanine analogue **24** ........ 125

$^{13}$C NMR of *rac*-phenylalanine analogue **24** ........ 126

$^{1}$H NMR of *rac*-thianaphthene alanine analogue **25** ........ 127

$^{13}$C NMR of *rac*-thianaphthene alanine analogue **25** ........ 128

$^{1}$H NMR of *rac*-pyrrole alanine analogue **26** ........ 129

$^{19}$F NMR of *rac*-pyrrole alanine analogue **26** ........ 130

$^{13}$C NMR of *rac*-pyrrole alanine analogue **26** ........ 131

$^{1}$H NMR of *rac*-isoquinoline alanine analogue **27** ........ 132

$^{13}$C NMR of *rac*-isoquinoline alanine analogue **27** ........ 133

$^{1}$H NMR of *rac*-quinoline alanine analogue **28** ........ 134

$^{13}$C NMR of *rac*-quinoline alanine analogue **28** ........ 135

$^{1}$H NMR of *rac*-pyridine alanine analogue **29** ........ 136

$^{13}$C NMR of *rac*-pyridine alanine analogue **29** ........ 137

$^{1}$H NMR of flubiprofen-derived phenylethylazide **30** ........ 138

$^{19}$F NMR of flubiprofen-derived phenylethylazide **30** ........ 139

$^{13}$C NMR of flubiprofen-derived phenylethylazide **30** ........ 140

$^{1}$H NMR of flubiprofen-derived phenylethylazide **31** ........ 141

$^{19}$F NMR of flubiprofen-derived phenylethylazide **31** ........ 142

$^{13}$C NMR of flubiprofen-derived phenylethylazide **31** ........ 143

$^{1}$H NMR of flubiprofen-derived phenylethylazide **32** ........ 144

$^{19}$F NMR of flubiprofen-derived phenylethylazide **32** ........ 145

$^{31}$P NMR of flubiprofen-derived phenylethylazide **32** ........ 146

$^{13}$C NMR of flubiprofen-derived phenylethylazide **32** ........ 147

$^{1}$H NMR of flubiprofen-derived phenylethylazide **33** ........ 148

$^{19}$F NMR of flubiprofen-derived phenylethylazide **33** ........ 149

$^{13}$C NMR of flubiprofen-derived phenylethylazide **33** ........ 150

$^{1}$H NMR of flubiprofen-derived phenylethylazide **34** ........ 151

$^{19}$F NMR of flubiprofen-derived phenylethylazide **34** ........ 152

$^{13}C$ NMR of flubiprofen-derived phenylethylazide **34** ........ 153

$^{1}H$ NMR of flubiprofen-derived phenylethylazide **35** ........ 154

$^{19}F$ NMR of flubiprofen-derived phenylethylazide **35**........ 155

$^{13}C$ NMR of flubiprofen-derived phenylethylazide **35** ........ 156

$^{1}H$ NMR of flubiprofen-derived phenylethylazide **36** ........ 157

$^{19}F$ NMR of flubiprofen-derived phenylethylazide **36**........ 158

$^{13}C$ NMR of flubiprofen-derived phenylethylazide **36** ........ 159

$^{1}H$ NMR of flubiprofen-derived phenylethylazide **37** ........ 160

$^{19}F$ NMR of flubiprofen-derived phenylethylazide **37**........ 161

$^{13}C$ NMR of flubiprofen-derived phenylethylazide **37** ........ 162

$^{1}H$ NMR of flubiprofen-derived phenylethylazide **38** ........ 163

$^{19}F$ NMR of flubiprofen-derived phenylethylazide **38**........ 164

$^{13}C$ NMR of flubiprofen-derived phenylethylazide **38** ........ 165

$^{1}H$ NMR of flubiprofen-derived phenylethylazide **39** ........ 166

$^{19}F$ NMR of flubiprofen-derived phenylethylazide **39**........ 167

$^{13}C$ NMR of flubiprofen-derived phenylethylazide **39** ........ 168

$^{1}H$ NMR of *rac*-flubiprofen-derived phenylethylazide **40** ........ 169

$^{19}F$ NMR of *rac*-flubiprofen-derived phenylethylazide **40** ........ 170

$^{13}C$ NMR of *rac*-flubiprofen-derived phenylethylazide **40**........ 171

$^{1}H$ NMR of flubiprofen-derived phenylethylazide **41** ........ 172

$^{19}F$ NMR of flubiprofen-derived phenylethylazide **41**........ 173

$^{13}C$ NMR of flubiprofen-derived phenylethylazide **41** ........ 174

$^{1}H$ NMR of *rac*-pyridinylethylazide **42**........ 175

$^{13}C$ NMR of *rac*-pyridinylethylazide **42** ........ 176

$^{1}H$ NMR of *rac*-pyridinylethylazide **43**........ 177

$^{13}C$ NMR of *rac*-pyridinylethylazide **43** ........ 178

$^{1}H$ NMR of pyridinylethylazide **44**........ 179

$^{13}C$ NMR of pyridinylethylazide **44**........ 180

$^{1}H$ NMR of *rac*-pyridinylethylazide **45**........ 181

$^{13}$C NMR of *rac*-pyridinylethylazide **45** .......... 182

$^{1}$H NMR of *rac*-pyridinylethylazide **46** .......... 183

$^{13}$C NMR of *rac*-pyridinylethylazide **46** .......... 184

$^{1}$H NMR of pyridinylethylazide **47** .......... 185

$^{13}$C NMR of pyridinylethylazide **47** .......... 186

$^{1}$H NMR of nootkatone-derived pyridinylethylazide **48** .......... 187

$^{13}$C NMR of nootkatone-derived pyridinylethylazide **48** .......... 188

$^{1}$H NMR of phenylethylazide **49** .......... 189

$^{19}$F NMR of phenylethylazide **49** .......... 190

$^{13}$C NMR of phenylethylazide **49** .......... 191

$^{1}$H NMR of *rac*-phenylethylazide **50** .......... 192

$^{19}$F NMR of *rac*-phenylethylazide **50** .......... 193

$^{13}$C NMR of *rac*-phenylethylazide **50** .......... 194

$^{1}$H NMR of valinate-derived phenylethylazide **51** .......... 195

$^{19}$F NMR of valinate-derived phenylethylazide **51** .......... 196

$^{13}$C NMR of valinate-derived phenylethylazide **51** .......... 197

$^{1}$H NMR of *rac*-fenofibrate-derived phenylethylazide **52** .......... 198

$^{19}$F NMR of *rac*-fenofibrate-derived phenylethylazide **52** .......... 199

$^{13}$C NMR of *rac*-fenofibrate-derived phenylethylazide **52** .......... 200

$^{1}$H NMR of aniline-derived thianthrenium salt **54** .......... 201

$^{19}$F NMR of aniline-derived thianthrenium salt **54** .......... 202

$^{13}$C NMR of aniline-derived thianthrenium salt **54** .......... 203

$^{1}$H NMR of *rac*-melphalan precursor **55** .......... 204

$^{13}$C NMR of *rac*-melphalan precursor **55** .......... 205

$^{1}$H NMR of racemic melphalan (**56**) .......... 206

$^{13}$C NMR of racemic melphalan (**56**) .......... 207

$^{1}$H NMR of *rac*-pyridinylethylazide S**57** .......... 208

$^{13}$C NMR of *rac*-pyridinylethylazide S**57** .......... 209

$^{1}$H NMR of rearrangement product S**57**’ .......... 210

$^{13}$C NMR of rearrangement product S**57**’ .......... 211

$^{1}$H NMR of nimesulide-derived thianthrenium salt TT-**5** .......... 212

$^{19}$F NMR of nimesulide-derived thianthrenium salt TT-**5** .......... 213

$^{13}$C NMR of nimesulide-derived thianthrenium salt TT-**5** .......... 214

$^{1}$H NMR of meclofenamic acid-derived thianthrenium salt TT-**6** .......... 215

$^{19}$F NMR of meclofenamic acid-derived thianthrenium salt TT-**6** .......... 216

$^{13}$C NMR of meclofenamic acid-derived thianthrenium salt TT-**6** .......... 217

$^{1}$H NMR of benzbromarone-derived thianthrenium salt TT-**7** .......... 218

$^{19}$F NMR of benzbromarone-derived thianthrenium salt TT-**7** .......... 219

$^{13}$C NMR of benzbromarone-derived thianthrenium salt TT-**7** .......... 220

$^{1}$H NMR of estrone-derived thianthrenium salt TT-**10** .......... 221

$^{19}$F NMR of estrone-derived thianthrenium salt TT-**10** .......... 222

$^{13}$C NMR of estrone-derived thianthrenium salt TT-**10** .......... 223

$^{1}$H NMR of tianeptine intermediate-derived thianthrenium salt TT-**11** .......... 224

$^{19}$F NMR of tianeptine intermediate-derived thianthrenium salt TT-**11** .......... 225

$^{13}$C NMR of tianeptine intermediate-derived thianthrenium salt TT-**11** .......... 226

$^{1}$H NMR of quinoline-derived thianthrenium salt TT-**14** .......... 227

$^{19}$F NMR of quinoline -derived thianthrenium salt TT-**14** .......... 228

$^{13}$C NMR of quinoline-derived thianthrenium salt TT-**14** .......... 229

$^{1}$H–$^{13}$C HSQC of quinoline-derived thianthrenium salt TT-**14** .......... 230

$^{1}$H–$^{13}$C HMBC of quinoline-derived thianthrenium salt TT-**14** .......... 231

$^{1}$H–$^{1}$H COSY of quinoline-derived thianthrenium salt TT-**14** .......... 232

$^{1}$H–$^{1}$H NOSEY of quinoline-derived thianthrenium salt TT-**14** .......... 233

$^{1}$H NMR of 4-methoxydiphenyl ether-derived thianthrenium salt TT-**19** .......... 234

$^{19}$F NMR of 4-methoxydiphenyl ether-derived thianthrenium salt TT-**19** .......... 235

$^{13}$C NMR of 4-methoxydiphenyl ether-derived thianthrenium salt TT-**19** .......... 236

$^{1}$H NMR of 2-chloroanisole-derived thianthrenium salt TT-**21** .......... 237

$^{19}$F NMR of 2-chloroanisole-derived thianthrenium salt TT-**21** .......... 238

$^{13}$C NMR of 2-chloroanisole-derived thianthrenium salt TT-**21** .......... 239

$^{1}H$ NMR of thianaphthene-derived thianthrenium salt TT-**25** ........ 240
$^{19}F$ NMR of thianaphthene-derived thianthrenium salt TT-**25** ........ 241
$^{13}C$ NMR of thianaphthene-derived thianthrenium salt TT-**25** ........ 242
$^{1}H$ NMR of pyrazol-derived thianthrenium salt TT-**26** ........ 243
$^{19}F$ NMR of pyrazol-derived thianthrenium salt TT-**26** ........ 244
$^{13}C$ NMR of pyrazol-derived thianthrenium salt TT-**26** ........ 245
$^{1}H$ NMR of isoquinoline-derived thianthrenium salt TT-**27** ........ 246
$^{19}F$ NMR of isoquinoline-derived thianthrenium salt TT-**27** ........ 247
$^{13}C$ NMR of isoquinoline-derived thianthrenium salt TT-**27** ........ 248
$^{1}H$ NMR of quinoline-derived thianthrenium salt TT-**28** ........ 249
$^{19}F$ NMR of quinoline-derived thianthrenium salt TT-**28** ........ 250
$^{13}C$ NMR of quinoline-derived thianthrenium salt TT-**28** ........ 251
$^{1}H$ NMR of pyridine-derived thianthrenium salt TT-**29** ........ 252
$^{19}F$ NMR of pyridine-derived thianthrenium salt TT-**29** ........ 253
$^{13}C$ NMR of pyridine-derived thianthrenium salt TT-**29** ........ 254
$^{1}H$ NMR of *rac*-BINAPCu(MeCN)$BF_4$ ........ 255
$^{13}C$ NMR of *rac*-BINAPCu(MeCN)$BF_4$ ........ 256
$^{19}F$ NMR of *rac*-BINAPCu(MeCN)$BF_4$ ........ 257
$^{31}P$ NMR of *rac*-BINAPCu(MeCN)$BF_4$ ........ 258
$^{1}H$ NMR of *rac*-BINAPCu$N_3$ (**2**) ........ 259
$^{13}C$ NMR of *rac*-BINAPCu$N_3$ (**2**) ........ 260
$^{31}P$ NMR of *rac*-BINAPCu$N_3$ (**2**) ........ 261

REFERENCES ........ 262

# MATERIALS AND METHODS

All air- and moisture-insensitive reactions were carried out under ambient atmosphere and monitored by thin-layer chromatography (TLC). Concentration under reduced pressure was performed by rotary evaporation at 25–40 °C at an appropriate pressure. Purified compounds were further dried under high vacuum (0.010–0.005 mbar). Yields refer to purified and spectroscopically pure compounds. All air- and moisture-sensitive manipulations were performed using oven-dried glassware (120 °C for a minimum of 12 hours) and standard Schlenk techniques under an atmosphere of argon.

### Solvents

Anhydrous MeCN was obtained from *Phoenix Solvent Drying Systems*. Anhydrous acetone and DMSO were dried by storage over 4Å molecular sieves. All deuterated solvents were purchased from *Euriso-Top*.

### Chromatography

Thin layer chromatography (TLC) was performed using EMD TLC plates pre-coated with 250 μm thickness silica gel 60 F254 plates and visualized by fluorescence quenching under 254 nm UV light or permanganate stain. Flash chromatography was performed using silica gel (40–63 μm particle size) purchased from Geduran®.

### Spectroscopy and Instruments

NMR spectra were recorded on a *Bruker Ascend* TM 500 spectrometer operating at 500 MHz, 471 MHz and 126 MHz, for $^{1}H$, $^{19}F$, and $^{13}C$ acquisitions, respectively. Chemical shifts are reported in ppm with the solvent residual peak as the internal standard. For $^{1}H$ NMR: $CDCl_3$, 7.26; $CD_3CN$, 1.96; $CD_2Cl_2$, 5.32; For $^{13}C$ NMR: $CDCl_3$, 77.16; $CD_3CN$, 1.32; $CD_2Cl_2$, 53.84.[1] $^{19}F$ NMR spectra were referenced using a unified chemical shift scale based on the $^{1}H$ resonance of tetramethylsilane (1% v/v solution in the respective solvent). Data is reported as follows: s = singlet, d = doublet, t = triplet, q = quartet, quin = quintet, sext = sextet, sept = septet, m = multiplet, bs = broad singlet; coupling constants in Hz.

### Starting materials

All substrates were used as received from commercial suppliers or prepared according to published procedures, unless otherwise stated. Chemicals were purchased from *Sigma-Aldrich*, *TCI*, or *Alfa Aesar*. Thianthrene-S-oxide[2] (**TTO**), **TT-8**[3], **TT-13**[3], **TT-18**[3], **TT-23**[3], **TT-24**[3] were prepared according to the literature.

# EXPERIMENTAL DATA

## Selected complex pharmaceuticals containing arylethylamine fragment

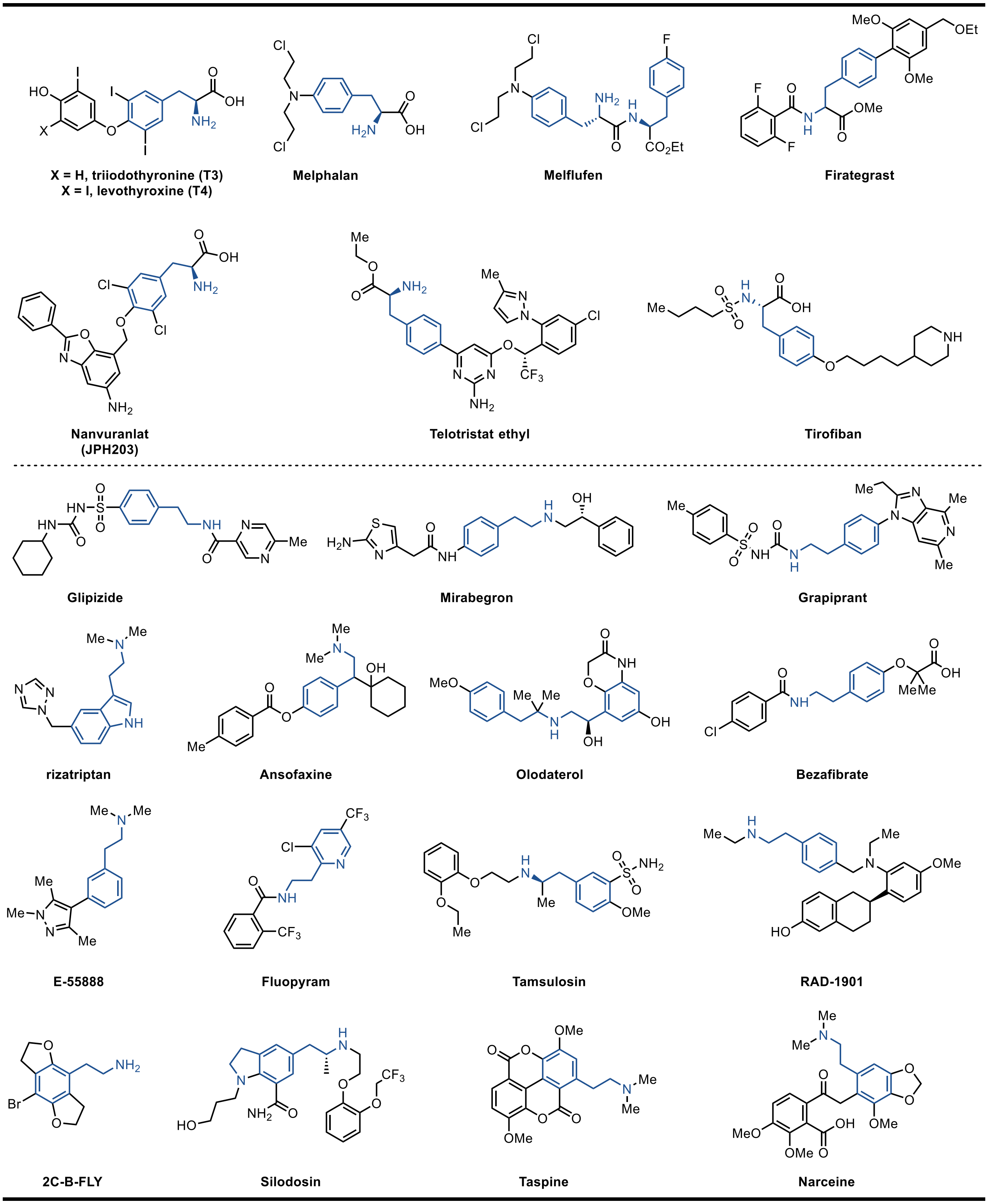


**Figure S1**. Selected complex pharmaceuticals containing arylethylamine fragment.

## Reaction condition optimization

### General procedure for condition optimization

To a 4-mL borosilicate vial containing a Teflon-coated magnetic stirring bar were added arylthianthrenium salt (0.100 mmol, 1.00 equiv), photocatalyst, copper catalyst, and amine source (0.200 mmol, 2.00 equiv). The vial was transferred into a nitrogen-filled glovebox. Dry MeCN and alkene (0.300 mmol, 3.00 equiv) were added. The vial was sealed with a Teflon-lined screw cap, removed from the glovebox, and irradiated under 456 nm LEDs. Subsequently, the reaction mixture was concentrated under reduced pressure. The resulting residue was dissolved in $CDCl_3$ (0.6 mL); internal standard $CH_2Br_2$ (17.4 mg, 0.100 mmol, 1.00 equiv.) was added, and the mixture was analyzed by $^1H$ NMR spectroscopy.

**Table S1**. Screening of amine sources with PTH as photocatalyst

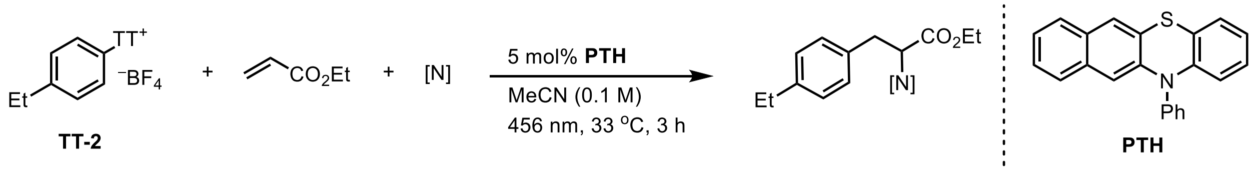


| Entry | Amine sources | bases | Yield (%) |
|---|---|---|---|
| 1 | potassium phthalimide | – | <2 |
| 2[a] | pyrrole | 2.0 equiv MeONa | <2 |
| 3 | Morpholine | 2.0 equiv $K_3PO_4$ | <2 |
| 4 | $PhNH_2$ | 2.0 equiv BTMG | <2 |
| 5 | KOCN | – | <2 |
| 6 | $NaN_3$ | – | <2 |

[a]amine and base were premixed for 30 min.

**Table S2**. Screening of amine sources with iridium photocatalyst and copper catalyst

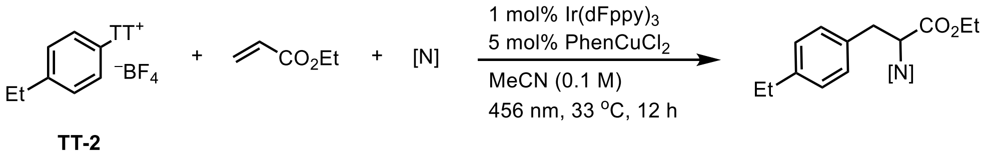


| Entry | Amine sources | bases | Yield (%) |
|---|---|---|---|
| 1 | Morpholine | 2.0 equiv $K_3PO_4$ | <2 |
| 2 | Methyl carbamate | 2.0 equiv NaH | <2 |
| 3 | Methyl carbamate | 2.0 equiv $K_3PO_4$ | <2 |
| **4** | **$NaN_3$** | **without base** | **24** |

**Table S3**. Screening of photocatalysts with $NaN_3$ as amine source

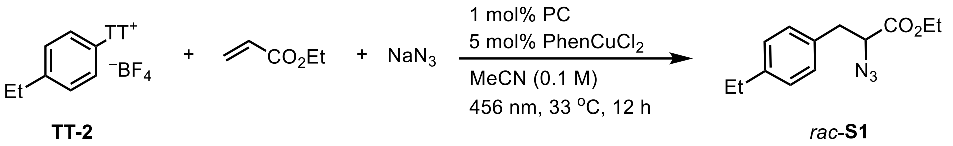


| Entry | Photocatalysts (PCs) | Yield (%) |
|---|---|---|
| 1 | $Ir[dFppy]_3$ | 24 |
| 2 | $Ir(ppy)_3$ | 21 |
| 3 | $Ir[dF(CF_3)ppy]_2dtbbpyPF_6$ | 26 |
| 4 | 10 mol% PTH | 20 |
| 5[a] | 10 mol% TXO | 12 |
| 6 | $Ru(bpy)_2Cl_2 \cdot 6H_2O$ | <2 |

[a]390 nm LEDs was used.

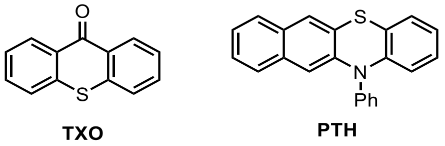


**Table S4**. Screening of ligands using the reported condition for amidation of alkyl halides[4]

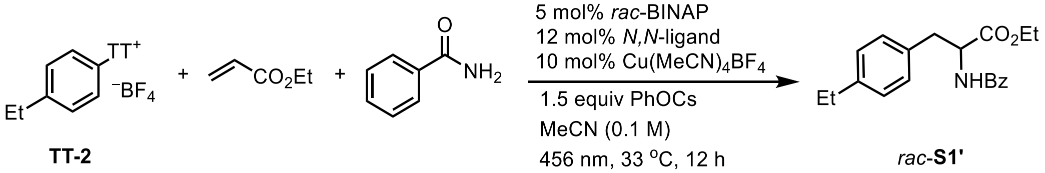


| Entry | *N,N*-ligands | Yield (%) |
|---|---|---|
| 1 | neocuproine | <2 |
| 2[a] | neocuproine | <2 |
| 3 | 1,10-phenanthroline | <2 |
| 4 | dtbbpy | <2 |
| 5 | 2,2':6',2''-terpyridine | <2 |
| 6 | 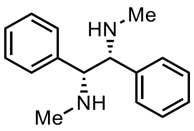 | <2 |
| 7[b] | – | <2 |
| 8[b,c] | – | 22 |

[a]without *rac*-BINAP; [b]15mol% *rac*-BINAP; [c]$NaN_3$ was used instead of benzamide in the absence of PhOCs.

**Table S5**. Screening of amine sources with *rac*-BINAP-Cu as both photocatalyst and copper catalyst

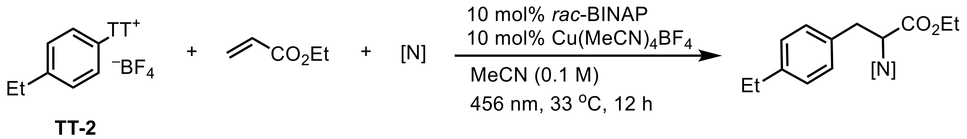


**TT-2**

| Entry | Amine sources | bases | Yield of (%) |
|---|---|---|---|
| 1 | potassium phthalimide | – | <2 |
| 2 | $LiNH_2$ | – | <2 |
| 3[a] | $PhNH_2$ | 2.0 equiv $^{n}$BuLi | <2 |
| 4[a] | 1,2,4-triazole | 2.0 equiv MeONa | <2 |
| 5 | sodium saccharin | – | <2 |
| 6 | $(NC)_2NNa$ | – | <2 |
| 7 | $Boc_2NK$ | – | <2 |
| 8 | Morpholine | 2.0 equiv $K_3PO_4$ | <2 |
| 9 | KOCN | – | <2 |
| 10 | $NaN_3$ | – | 18 |
| **11[b]** | **$NaN_3$** | **–** | **47** |

[a]amine and base were premixed for 30 min; [b]acrylonitrile instead of ethyl acrylate was used.

**Table S6**. Screening of diphosphine ligands with (P,P)-Cu as both photocatalyst and copper catalyst

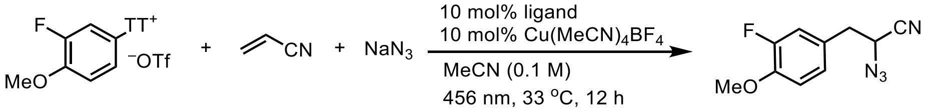


**TT-3**

*rac*-**S2**

| Entry | ligands | Yield (%) |
|---|---|---|
| 1 | Neocuproine | 0 |
| **2** | ***rac*-BINAP** | **57** |
| 3 | (*R,R*)-DTBM-Segphos | 14 |
| 4[a] | Dis(diphenylphosphino)benzene | 18 |
| 5 | Xantphos | <2 |
| 6 | DPPF | <2 |
| 7[b] | 2 mol% $Cu(dpp)_2PF_6$ | <2 |
| 8[b] | 2 mol% $Cu(dpp)_2PF_6$<br>8 mol% BQA<br>8 mol% $Cu(OTf)_2$ | <2 |

[a]390 nm LEDs was used; [b]without $Cu(MeCN)_4BF_4$

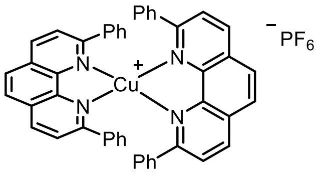


Cu(dpp)$_2$PF$_6$

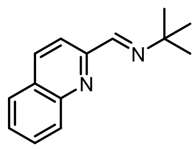


BQA

**Table S7**. Screening of solvents with *rac*-BINAP-Cu as catalysts

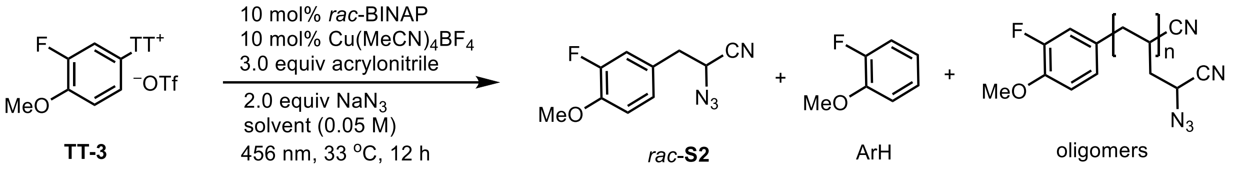


| Entry | solvents | Conv. (%) | Yield of **S2** (%) | Yield of ArH (%) | Yield of oligomers (%) |
|---|---|---|---|---|---|
| **1** | **MeCN** | **>99** | **57** | **14** | **10** |
| 2 | $^t$BuCN | 80 | 32 | 4 | 13 |
| 3 | acetone | >99 | 41 | 21 | 7 |
| 4 | EA | >99 | 23 | 10 | 20 |

**Table S8**. Screening of catalyst loading

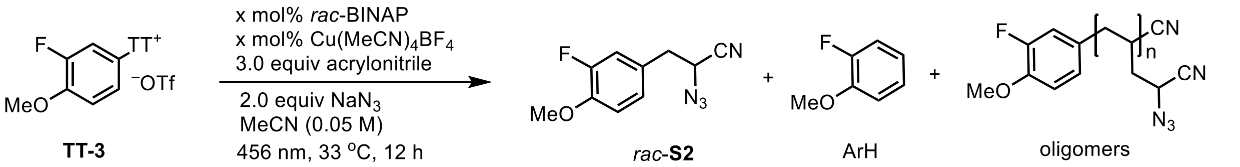


| Entry | x | Conv. (%) | Yield of **S2** (%) | Yield of ArH (%) | Yield of oligomers (%) |
|---|---|---|---|---|---|
| **1** | **10** | >**99** | **57** | **14** | **10** |
| **2** | **5** | >**99** | **56** | **12** | **10** |
| 3 | 2 | 94 | 56 | 14 | 8 |
| 4 | 1 | 22 | 14 | 3 | <2 |

**Table S9**. Screening of acrylonitrile loading

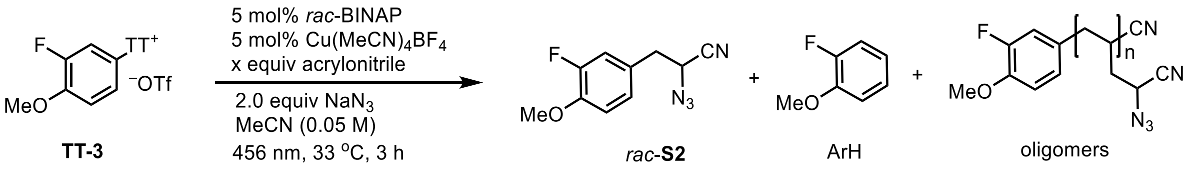

| Entry | x | Conv. (%) | Yield of **S2** (%) | Yield of ArH (%) | Yield of oligomers (%) |
|---|---|---|---|---|---|
| 1 | 1.5 | 94 | 43 | 21 | 9 |
| **2** | **3** | **>99** | **53** | **13** | **13** |
| 3 | 5 | >99 | 52 | 8 | 21 |
| 4 | 10 | >99 | 49 | 4 | 38 |

**Table S10**. Screening of temperature, concentration, solvents, and additives

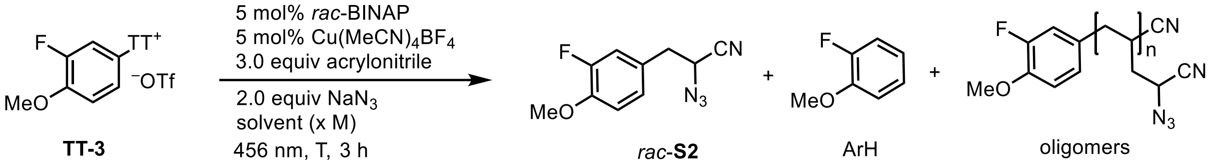


| Entry | x | T | Solvents | Conv. (%) | Yield of **S2** (%) | Yield of ArH (%) | Yield of oligomers (%) |
|---|---|---|---|---|---|---|---|
| 1 | 0.05 | 33 | MeCN | >99 | 53 | 13 | 13 |
| **2** | **0.05** | **10** | **MeCN** | >**99** | **74** | **10** | **6** |
| 3 | 0.05 | 0 | MeCN | 85 | 50 | 7 | 5 |
| 4 | 0.05 | -7 | MeCN | 54 | 42 | 3 | 3 |
| **5** | **0.1** | **10** | **MeCN** | >**99** | **74** | **7** | **14** |
| **6** | **0.2** | **10** | **MeCN** | **95** | **73** | **3** | **12** |
| 7 | 0.1 | 10 | acetone | >99 | 67 | 12 | 4 |
| 8 | 0.1 | 10 | MeCN/acetone = 1:1 | >99 | 66 | 9 | 3 |
| 9[a] | 0.1 | 10 | MeCN | >99 | 62 | 6 | 10 |
| 10[b] | 0.1 | 10 | MeCN | 0 | 0 | 0 | 0 |

[a]10 mol% $PPh_3$ was added; [b]10 mol% 1,10-phenanthroline was added.

**Table S11**. Screening of the loading of $NaN_3$

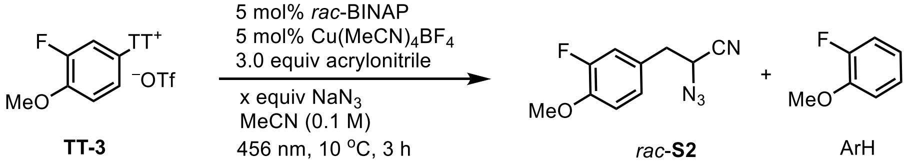


| Entry | x | Conv. (%) | Yield of **S2** (%) | Yield of ArH (%) |
|---|---|---|---|---|
| 1 | 3 | >99 | 74 | 6 |
| 2 | 2 | >99 | 73 | 6 |

| | | | | |
|---|---|---|---|---|
| **3** | **1.2** | >**99** | **75** | **5** |
| 4[a] | 1.2 | >99 | 73 | 6 |
| 5[b] | 1.2 | >99 | 27 | 15 |

[a]25 μL $H_2O$ was added; [b]DMSO was used as the solvent.

**Table S12**. Enantioselective azidoarylation[a]

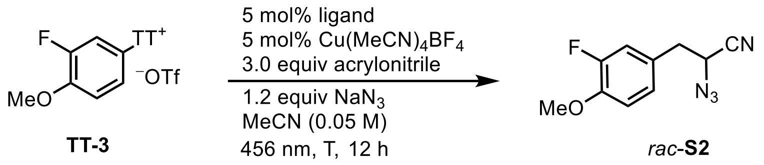


| Entry | Ligands | Temp./°C | Conv. (%) | Yield of **S2** (%) | ee of **S2** (%) |
|---|---|---|---|---|---|
| 1 | (*R,R*)-BINAP | 33 | >99 | 53 | 2 |
| 2 | (*R,R*)-BINAP | –40 | >99 | 68 | 8 |
| 3 | (*R,R*)-DTBM-segphos | –40 | >99 | 72 | 6 |

[a]HPLC condition: 150 mm Chiralpak IC-3, 4.6 mm i.D., *n*-Heptane / *i*-Propanol = 95:5, 1.0 mL / min, 7.3 MPa, 298 K, UV, 220 nm.

**Table S13**. Evaluation of other aryl electrophiles and radical acceptors

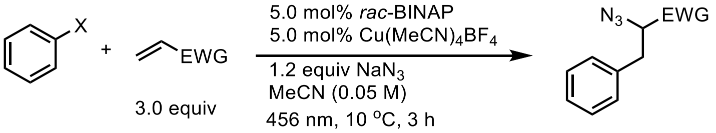


| Entry | X | EWG | Conv. (%) | Yield (%) |
|---|---|---|---|---|
| 1 | Br | CN (**1**) | <5 | <2 |
| 2 | I | CN (**1**) | <5 | <2 |
| 3[a] | $N_2^+$ $^-$BF4 | CN (**1**) | >99 | <2 |
| 4 | PhI$^+$ $^-$OTf | CN (**1**) | >99 | 44 |
| **5** | **TT$^+$ $^-$OTf (TT-1)** | **CN(1)** | **>99** | **75** |
| 6 | TT$^+$ $^-$OTf (**TT-1**) | $CO_2Me$ | >99 | 25 |
| 7 | TT$^+$ $^-$OTf (**TT-1**) | $CO_2Et$ | >99 | 30 |
| 8 | TT$^+$ $^-$OTf (**TT-1**) | $CO_2$Dipp | >99 | 38 |

[a]with or without light, $PhN_3$ was observed in 22–48% yield; Dipp, 2,6-diisopropylphenyl.

## Scope of azidoarylation of alkenes with arylthianthrenium salts

### General procedure for azidoarylation of alkenes with arylthianthrenium salts

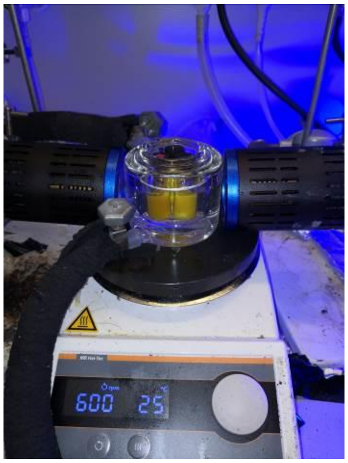

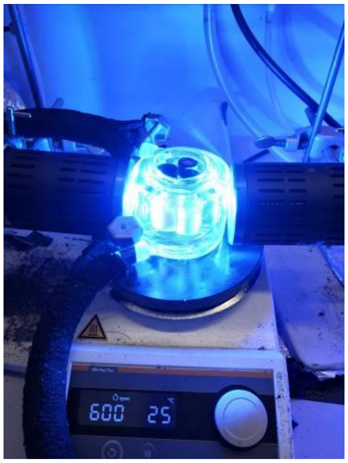

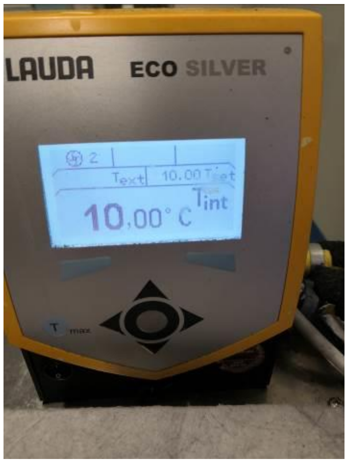


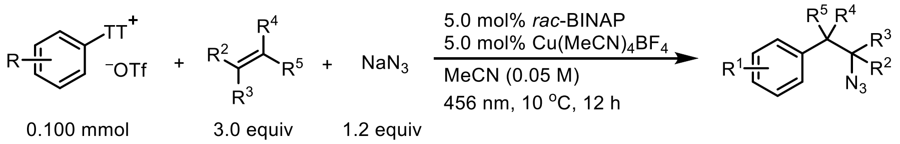


**General procedure using Schlenk line:** To a 4-mL borosilicate vial containing a Teflon-coated magnetic stirring bar were added arylthianthrenium salt (0.100 mmol, 1.00 equiv), *rac*-BINAP (3.1 mg, 5.0 μmol, 5.0 mol%), sodium azide (7.8 mg, 0.12 mmol, 1.2 equiv), and $Cu(MeCN)_4BF_4$ (1.6 mg, 5.0 μmol, 5.0 mol%). The vial was sealed with a Teflon-lined screw cap, and evacuated and backfilled with argon for three times using a Schlenk line. Dry MeCN (2 mL, c = 0.05 M) and alkene (0.30 mmol, 3.0 equiv) were added via syringe. The vial was sealed with parafilm and transferred to a cryocooler precooled at 10 °C where the reaction mixture was stirred for 5 min without irradiation and then irradiated by 456 nm LEDs (40 W) for 12 h. Subsequently, silica gel (5–10 mL) was added, and the reaction mixture was concentrated to dryness under reduced pressure. The resulting residue was purified by chromatography on silica gel to afford the desired product.

**General procedure using glovebox:** To a 4-mL borosilicate vial containing a Teflon-coated magnetic stirring bar were added arylthianthrenium salt (0.100 mmol, 1.00 equiv), *rac*-BINAP (3.1 mg, 5.0 μmol, 5.0 mol%), and sodium azide (7.8 mg, 0.12 mmol, 1.2 equiv). The vial was transferred into a nitrogen-filled glovebox. $Cu(MeCN)_4BF_4$ (1.6 mg, 5.0 μmol, 5.0 mol%), dry MeCN (2 mL, c = 0.05 M), and alkene (0.30 mmol, 3.0 equiv) were added. The vial was sealed with a Teflon-lined screw cap and transferred to a cryocooler precooled at 10 °C where the reaction mixture was stirred for 5 min without irradiation and then irradiated by 456 nm LEDs (40 W) for 12 h. Subsequently, silica gel (5–10 mL) was added, and the reaction mixture was concentrated to dryness under reduced pressure. The resulting residue was purified by chromatography on silica gel to afford the desired product.

For simplicity, in our research, we have opted to execute the transformation for most compounds by using a glovebox. Control experiments showed that yields were within error of measurement.

**Cautions: Sodium azide can be fatally toxic, and even minute amounts can cause symptoms.**

### *rac*-Nefiracetam-derived phenylalanine analogue 4

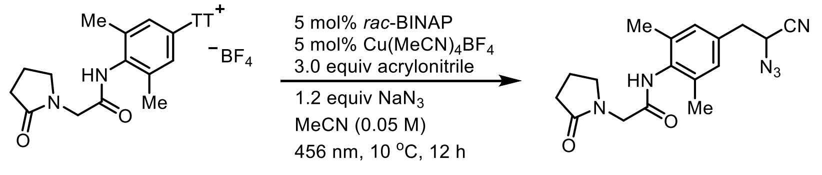


To a 4-mL borosilicate vial containing a Teflon-coated magnetic stirring bar were added **TT-4** (54.6 mg, 0.100 mmol, 1.00 equiv), *rac*-BINAP (3.1 mg, 5.0 μmol, 5.0 mol%), and sodium azide (7.8 mg, 0.12 mmol, 1.2 equiv). The vial was transferred into a nitrogen-filled glovebox. $Cu(MeCN)_4BF_4$ (1.6 mg, 5.0 μmol, 5.0 mol%), dry MeCN (2 mL, c = 0.05 M), and acrylonitrile (16 mg, 20 μL, 0.30 mmol, 3.0 equiv) were added. The vial was sealed with a Teflon-lined screw cap and transferred to a cryocooler precooled at 10 °C where the reaction mixture was stirred for 5 min without irradiation and then irradiated by 456 nm LEDs for 12 h. Subsequently, silica gel (5–10 mL) was added, and the reaction mixture was concentrated to dryness under reduced pressure. The resulting residue was purified by chromatography on silica gel (EtOAc/MeOH = 40:1) first and further purified by HPLC (Nucleodur C18 ISIS C18 (21×250 mm: 5 μm), 50:50 $MeOH/H_2O$ (v/v), flow rate = 20 mL/min, 35 ºC) to afford *rac*-**4** as a colorless oil in 65% yield (22.0 mg).

**R*f*** = 0.20 (EtOAc/MeOH = 40:1).

**NMR Spectroscopy:**

**$^1$H NMR** (500 MHz, $CDCl_3$, 23 ºC, δ): 7.79 (s, 1H), 7.00 (s, 2H), 4.35 (t, *J* = 7.1 Hz, 1H), 4.12 (s, 2H), 3.63 (t, *J* = 7.0 Hz, 2H), 3.09 – 2.97 (m, 2H), 2.48 (t, *J* = 8.1 Hz, 2H), 2.23 (s, 6H), 2.16 (p, *J* = 7.5 Hz, 2H).

**$^{13}$C NMR** (125 MHz, $CDCl_3$, 23 ºC, δ): 176.3, 167.0, 135.9, 133.4, 132.6, 129.2, 115.8, 52.4, 48.8, 48.0, 38.5, 30.4, 18.4, 18.2.

**HRMS-ESIpos (m/z)** calc'd for $C_{17}H_{20}O_2N_6Na$ $[M+Na]^+$, 363.1540; found, 363.1544; deviation: –1.0 ppm.

### *rac*-Nimesulide-derived phenylalanine analogue 5

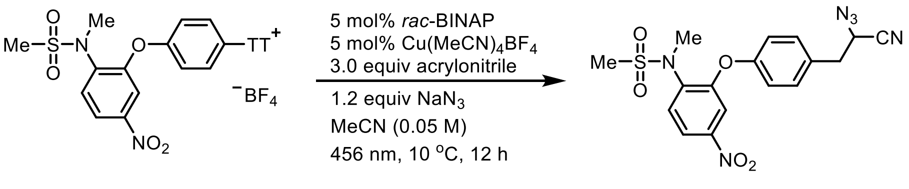


To a 4-mL borosilicate vial containing a Teflon-coated magnetic stirring bar were added **TT-5** (62.2 mg, 0.100 mmol, 1.00 equiv), *rac*-BINAP (3.1 mg, 5.0 μmol, 5.0 mol%), and sodium azide (7.8 mg, 0.12 mmol, 1.2 equiv). The vial was transferred into a nitrogen-filled glovebox. $Cu(MeCN)_4BF_4$ (1.6 mg, 5.0 μmol, 5.0 mol%),

dry MeCN (2 mL, c = 0.05 M), and acrylonitrile (16 mg, 20 μL, 0.30 mmol, 3.0 equiv) were added. The vial was sealed with a Teflon-lined screw cap and transferred to a cryocooler precooled at 10 °C where the reaction mixture was stirred for 5 min without irradiation and then irradiated by 456 nm LEDs for 12 h. Subsequently, silica gel (5–10 mL) was added, and the reaction mixture was concentrated to dryness under reduced pressure. The resulting residue was purified by chromatography on silica gel (hexanes/EtOAc = 3:1) to afford *rac*-**5** as a colorless oil in 45% yield (18.8 mg).

**R*f*** = 0.12 (hexanes/EtOAc = 3:1).

**NMR Spectroscopy:**

**$^{1}$H NMR** (500 MHz, $CDCl_3$, 23 ºC, δ): 8.00 (dd, *J* = 8.7, 2.5 Hz, 1H), 7.69 (d, *J* = 2.5 Hz, 1H), 7.66 (d, *J* = 8.7 Hz, 1H), 7.42 (d, *J* = 8.6 Hz, 2H), 7.13 (d, *J* = 8.6 Hz, 2H), 4.45 (t, *J* = 6.8 Hz, 1H), 3.39 (s, 3H), 3.18 (dd, *J* = 6.8, 1.5 Hz, 2H), 3.04 (s, 3H).

**$^{13}$C NMR** (125 MHz, $CDCl_3$, 23 ºC, δ): 154.5, 154.5, 147.8, 137.1, 132.5, 131.9, 131.0, 120.1, 118.5, 115.6, 113.1, 52.3, 38.7, 38.3, 37.8.

**HRMS-ESIpos (m/z)** calc'd for $C_{17}H_{16}O_5N_6SNa$ $[M+Na]^+$, 439.0795; found, 439.0799; deviation: –0.9 ppm.

***rac*-Meclofenamic acid-derived phenylalanine analogue 6**

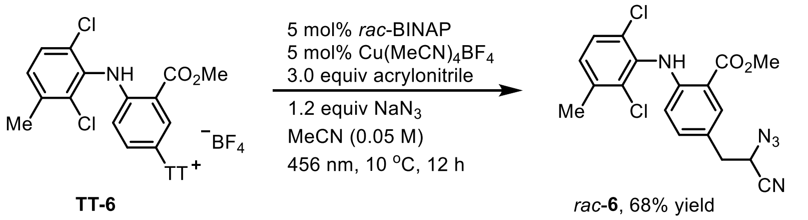


To a 4-mL borosilicate vial containing a Teflon-coated magnetic stirring bar were added **TT-6** (63.2 mg, 0.100 mmol, 1.00 equiv), *rac*-BINAP (3.1 mg, 5.0 μmol, 5.0 mol%), and sodium azide (7.8 mg, 0.12 mmol, 1.2 equiv). The vial was transferred into a nitrogen-filled glovebox. $Cu(MeCN)_4BF_4$ (1.6 mg, 5.0 μmol, 5.0 mol%), dry MeCN (2 mL, c = 0.05 M), and acrylonitrile (16 mg, 20 μL, 0.30 mmol, 3.0 equiv) were added. The vial was sealed with a Teflon-lined screw cap and transferred to a cryocooler precooled at 10 °C where the reaction mixture was stirred for 5 min without irradiation and then irradiated by 456 nm LEDs for 12 h. Subsequently, silica gel (5–10 mL) was added, and the reaction mixture was concentrated to dryness under reduced pressure. The resulting residue was purified by chromatography on silica gel (hexanes/EtOAc = 10:1) to afford *rac*-**6** as a colorless oil in 68% yield (27.1 mg).

**R*f*** = 0.20 (hexanes/EtOAc = 10:1).

**NMR Spectroscopy:**

**$^{1}$H NMR** (500 MHz, $CDCl_3$, 23 ºC, δ): 9.39 (s, 1H), 7.93 (d, *J* = 2.3 Hz, 1H), 7.34 (d, *J* = 8.3 Hz, 1H), 7.21 (dd, *J* = 8.6, 2.3 Hz, 1H), 7.15 (d, *J* = 8.3 Hz, 1H), 6.34 (d, *J* = 8.6 Hz, 1H), 4.33 (t, *J* = 7.1 Hz, 1H), 3.97 (s, 3H), 3.13 – 2.98 (m, 2H), 2.43 (s, 3H).

**$^{13}$C NMR** (125 MHz, $CDCl_3$, 23 ºC, δ): 168.5, 147.4, 136.6, 134.9, 134.8, 134.3, 132.3, 131.2, 128.8, 127.8, 121.9, 115.9, 114.4, 111.7, 52.6, 52.0, 38.2, 20.6.

**HRMS-ESIpos (m/z)** calc'd for $C_{18}H_{16}N_5O_2Cl_2$ $[M+H]^+$, 404.0676; found, 404.0674; deviation: +0.4 ppm.

### ***rac*-Benzbromarone-derived phenylalanine analogue 7**

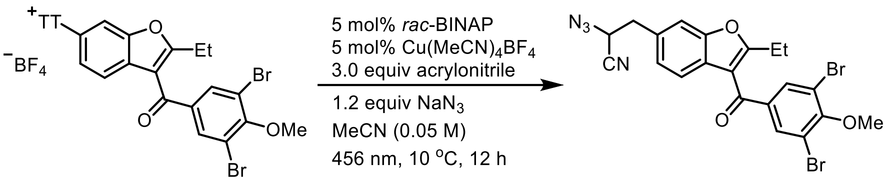


**TT-7** *rac*-**7**, 53% yield

To a 4-mL borosilicate vial containing a Teflon-coated magnetic stirring bar were added **TT-7** (73.8 mg, 0.100 mmol, 1.00 equiv), *rac*-BINAP (3.1 mg, 5.0 μmol, 5.0 mol%), and sodium azide (7.8 mg, 0.12 mmol, 1.2 equiv). The vial was transferred into a nitrogen-filled glovebox. $Cu(MeCN)_4BF_4$ (1.6 mg, 5.0 μmol, 5.0 mol%), dry MeCN (2 mL, c = 0.05 M), and acrylonitrile (16 mg, 20 μL, 0.30 mmol, 3.0 equiv) were added. The vial was sealed with a Teflon-lined screw cap and transferred to a cryocooler precooled at 10 °C where the reaction mixture was stirred for 5 min without irradiation and then irradiated by 456 nm LEDs for 12 h. Subsequently, silica gel (5–10 mL) was added, and the reaction mixture was concentrated to dryness under reduced pressure. The resulting residue was purified by chromatography on silica gel (hexanes/EtOAc = 10:1) to afford *rac*-**7** as a colorless oil in 53% yield (27.9 mg).

**R*f*** = 0.30 (hexanes/EtOAc = 10:1).

**NMR Spectroscopy:**

**$^1$H NMR** (500 MHz, $CDCl_3$, 23 ºC, δ): 8.00 (s, 2H), 7.48 (d, *J* = 1.4 Hz, 1H), 7.46 (d, *J* = 8.0 Hz, 1H), 7.19 (dd, *J* = 8.1, 1.5 Hz, 1H), 4.45 (t, *J* = 7.0 Hz, 1H), 4.01 (s, 3H), 3.25 (dd, *J* = 7.0, 1.6 Hz, 2H), 2.91 (q, *J* = 7.5 Hz, 2H), 1.38 (t, *J* = 7.6 Hz, 3H).

**$^{13}$C NMR** (125 MHz, $CDCl_3$, 23 ºC, δ): 187.8, 167.5, 157.8, 153.8, 137.0, 133.6, 130.4, 126.5, 125.2, 121.5, 118.6, 115.7, 115.3, 112.3, 60.9, 52.6, 39.1, 22.1, 12.2.

**HRMS-ESIpos (m/z)** calc'd for $C_{21}H_{16}N_4O_3Br_2Na$ $[M+Na]^+$, 552.9482; found, 552.9485; deviation: –0.5 ppm.

### **Cloquintocet-mexyl-derived phenylalanine analogue 8**

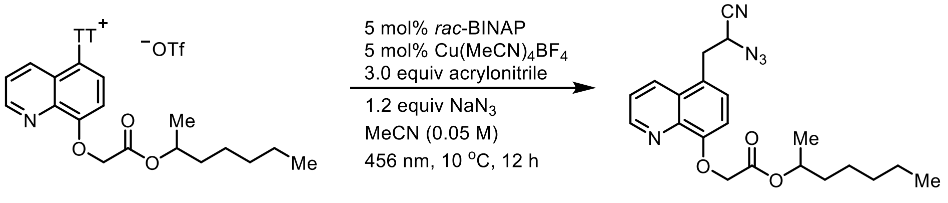


**TT-8** **8**, 70% yield

To a 4-mL borosilicate vial containing a Teflon-coated magnetic stirring bar were added **TT-8** (66.5 mg, 0.100 mmol, 1.00 equiv), *rac*-BINAP (3.1 mg, 5.0 μmol, 5.0 mol%), and sodium azide (7.8 mg, 0.12 mmol, 1.2 equiv). The vial was transferred into a nitrogen-filled glovebox. $Cu(MeCN)_4BF_4$ (1.6 mg, 5.0 μmol, 5.0 mol%), dry MeCN (2 mL, c = 0.05 M), and acrylonitrile (16 mg, 20 μL, 0.30 mmol, 3.0 equiv) were added. The vial was sealed with a Teflon-lined screw cap and transferred to a cryocooler precooled at 10 °C where the reaction mixture was stirred for 5 min without irradiation and then irradiated by 456 nm LEDs for 12 h. Subsequently, silica gel (5–10 mL) was added, and the reaction mixture was concentrated to dryness under reduced pressure. The resulting residue was purified by chromatography on silica gel (hexanes/acetone = 3:1) to afford **8** as a colorless oil in 70% yield (27.6 mg).

$\mathbf{R}_f$ = 0.19 (hexanes/acetone = 3:1).

**NMR Spectroscopy:**

$^{1}$**H NMR** (500 MHz, $CDCl_3$, 23 °C, δ): 9.01 (d, *J* = 4.1 Hz, 1H), 8.27 (d, *J* = 8.6 Hz, 1H), 7.54 (dd, *J* = 8.6, 4.1 Hz, 1H), 7.44 (d, *J* = 8.0 Hz, 1H), 6.96 (d, *J* = 8.0 Hz, 1H), 5.10 – 5.00 (m, 1H), 4.96 (s, 2H), 4.48 (t, *J* = 7.2 Hz, 1H), 3.56 – 3.44 (m, 2H), 1.71 – 1.53 (m, 1H), 1.53 – 1.43 (m, 1H), 1.34 – 1.14 (m, 9H), 0.89 – 0.83 (m, 3H).

$^{13}$**C NMR** (125 MHz, $CDCl_3$, 23 °C, δ): 168.2, 154.1, 149.5, 140.6, 131.2, 128.8, 127.8, 122.3, 122.2, 115.7, 108.8, 72.7, 66.2, 52.0, 35.7, 34.9, 31.5, 24.9, 22.5, 19.9, 14.0.

**HRMS-ESIpos (m/z)** calc'd for $C_{21}H_{26}N_5O_3$ $[M+H]^+$, 396.2030; found, 396.2033; deviation: –0.7 ppm.

***rac*-Diclofenac amide-derived phenylalanine analogue 9**

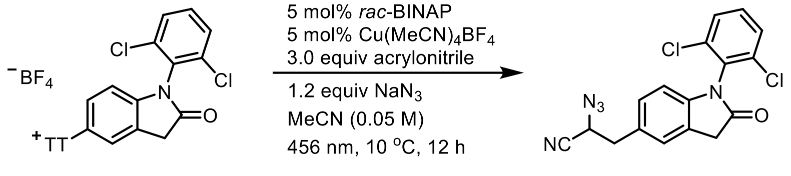


**TT-9** *rac*-**9**, 65% yield

To a 4-mL borosilicate vial containing a Teflon-coated magnetic stirring bar were added **TT-9** (58.0 mg, 0.100 mmol, 1.00 equiv), *rac*-BINAP (3.1 mg, 5.0 μmol, 5.0 mol%), and sodium azide (7.8 mg, 0.12 mmol, 1.2 equiv). The vial was transferred into a nitrogen-filled glovebox. $Cu(MeCN)_4BF_4$ (1.6 mg, 5.0 μmol, 5.0 mol%), dry MeCN (2 mL, c = 0.05 M), and acrylonitrile (16 mg, 20 μL, 0.30 mmol, 3.0 equiv) were added. The vial was sealed with a Teflon-lined screw cap and transferred to a cryocooler precooled at 10 °C where the reaction mixture was stirred for 5 min without irradiation and then irradiated by 456 nm LEDs for 12 h. Subsequently, silica gel (5–10 mL) was added, and the reaction mixture was concentrated to dryness under reduced pressure. The resulting residue was purified by chromatography on silica gel (hexanes/EtOAc = 3:1) to afford *rac*-**9** as a colorless oil in 65% yield (24.1 mg).

$\mathbf{R}_f$ = 0.18 (hexanes/EtOAc = 3:1).

**NMR Spectroscopy:**

**$^1$H NMR** (500 MHz, $CDCl_3$, 23 ºC, δ): 7.54 (d, *J* = 8.1 Hz, 2H), 7.41 (t, *J* = 8.2 Hz, 1H), 7.30 (s, 1H), 7.14 (dd, *J* = 8.0, 1.7 Hz, 1H), 6.42 (d, *J* = 8.0 Hz, 1H), 4.39 (t, *J* = 6.9 Hz, 1H), 3.81 (s, 2H), 3.22 – 3.07 (m, 2H).

**$^{13}$C NMR** (125 MHz, $CDCl_3$, 23 ºC, δ): 173.3, 143.3, 135.4, 130.9, 130.2, 129.3, 129.1, 128.2, 126.0, 125.1, 115.8, 109.5, 52.6, 38.8, 35.6.

**HRMS-ESIpos (m/z)** calc'd for $C_{17}H_{11}ON_5Cl_2Na$ $[M+Na]^+$, 394.0233; found, 394.0235; deviation: –0.5 ppm.

**Estrone-derived phenylalanine analogue 10**

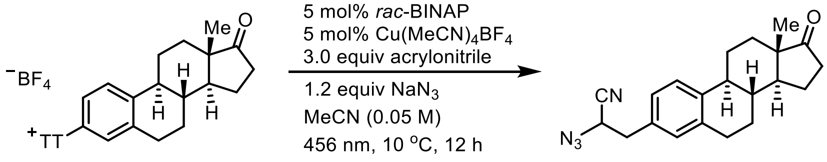


**TT-10** **10**, 52% yield

To a 4-mL borosilicate vial containing a Teflon-coated magnetic stirring bar were added **TT-10** (55.5 mg, 0.100 mmol, 1.00 equiv), *rac*-BINAP (3.1 mg, 5.0 μmol, 5.0 mol%), and sodium azide (7.8 mg, 0.12 mmol, 1.2 equiv). The vial was transferred into a nitrogen-filled glovebox. $Cu(MeCN)_4BF_4$ (1.6 mg, 5.0 μmol, 5.0 mol%), dry MeCN (2 mL, c = 0.05 M), and acrylonitrile (16 mg, 20 μL, 0.30 mmol, 3.0 equiv) were added. The vial was sealed with a Teflon-lined screw cap and transferred to a cryocooler precooled at 10 °C where the reaction mixture was stirred for 5 min without irradiation and then irradiated by 456 nm LEDs for 12 h. Subsequently, silica gel (5–10 mL) was added, and the reaction mixture was concentrated to dryness under reduced pressure. The resulting residue was purified by chromatography on silica gel (hexanes/EtOAc = 5:1) to afford **10** as a colorless oil in 52% yield (16.9 mg).

**$R_f$** = 0.21 (hexanes/EtOAc = 5:1).

**NMR Spectroscopy:**

**$^1$H NMR** (500 MHz, $CDCl_3$, 23 ºC, δ): 7.31 (d, *J* = 8.0 Hz, 1H), 7.08 (d, *J* = 7.9 Hz, 1H), 7.03 (s, 1H), 4.68 – 4.27 (m, 1H), 3.08 (dd, *J* = 7.2, 2.1 Hz, 2H), 2.94 (dd, *J* = 9.0, 4.2 Hz, 2H), 2.53 (dd, *J* = 19.0, 8.7 Hz, 1H), 2.47 – 2.40 (m, 1H), 2.37 – 2.27 (m, 1H), 2.23 – 2.12 (m, 1H), 2.13 – 2.02 (m, 2H), 2.02 – 1.96 (m, 1H), 1.70 – 1.61 (m, 3H), 1.58 – 1.45 (m, 3H), 0.94 (s, 3H).

**$^{13}$C NMR** (125 MHz, $CDCl_3$, 23 ºC, δ): 220.7, 139.8, 137.3, 130.9, 130.0, 126.7, 126.0, 115.9, 52.6, 50.5, 48.0, 44.3, 38.6, 38.0, 35.8, 31.6, 29.3, 26.4, 25.7, 21.6, 13.8.

**HRMS-ESIpos (m/z)** calc'd for $C_{21}H_{24}N_4ONa$ $[M+Na]^+$, 371.1842; found, 371.1841; deviation: +0.4 ppm.

***rac*-Tianeptine intermediate-derived phenylalanine analogue 11**

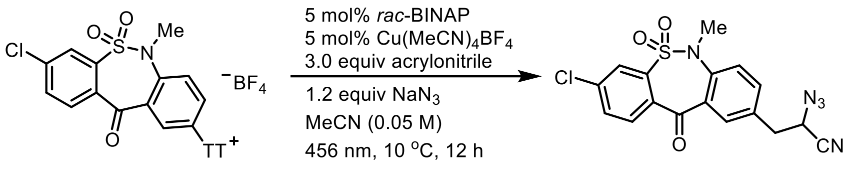


**TT-11** *rac*-**11**, 50% yield

To a 4-mL borosilicate vial containing a Teflon-coated magnetic stirring bar were added **TT-11** (60.9 mg, 0.100 mmol, 1.00 equiv), *rac*-BINAP (3.1 mg, 5.0 μmol, 5.0 mol%), and sodium azide (7.8 mg, 0.12 mmol, 1.2 equiv). The vial was transferred into a nitrogen-filled glovebox. $Cu(MeCN)_4BF_4$ (1.6 mg, 5.0 μmol, 5.0 mol%), dry MeCN (2 mL, c = 0.05 M), and acrylonitrile (16 mg, 20 μL, 0.30 mmol, 3.0 equiv) were added. The vial was sealed with a Teflon-lined screw cap and transferred to a cryocooler precooled at 10 °C where the reaction mixture was stirred for 5 min without irradiation and then irradiated by 456 nm LEDs for 12 h. Subsequently, silica gel (5–10 mL) was added, and the reaction mixture was concentrated to dryness under reduced pressure. The resulting residue was purified by chromatography on silica gel (hexanes/EtOAc = 4:1) to afford *rac*-**11** as a light yellow oil in 50% yield (20.2 mg).

**$R_f$** = 0.17 (hexanes/EtOAc = 4:1).

**NMR Spectroscopy:**

**$^1$H NMR** (500 MHz, $CDCl_3$, 23 ºC, δ): 8.23 (d, *J* = 2.3 Hz, 1H), 7.97 (d, *J* = 2.1 Hz, 1H), 7.93 (d, *J* = 8.3 Hz, 1H), 7.71 (dd, *J* = 8.3, 2.1 Hz, 1H), 7.61 (dd, *J* = 8.3, 2.3 Hz, 1H), 7.37 (d, *J* = 8.3 Hz, 1H), 4.49 (t, *J* = 6.9 Hz, 1H), 3.40 (s, 3H), 3.20 (dd, *J* = 7.0, 1.8 Hz, 2H).

**$^{13}$C NMR** (125 MHz, $CDCl_3$, 23 ºC, δ): 189.2, 141.2, 138.9, 138.3, 135.9, 134.2, 133.4, 133.3, 133.0, 131.6, 130.8, 125.3, 125.0, 115.5, 52.0, 38.8, 38.1.

**HRMS-ESIpos (m/z)** calc'd for $C_{17}H_{12}N_5O_3SClNa$ $[M+Na]^+$, 424.0242; found, 424.0245; deviation: –0.9 ppm.

**Flubiprofen-derived phenylalanine analogue 12**

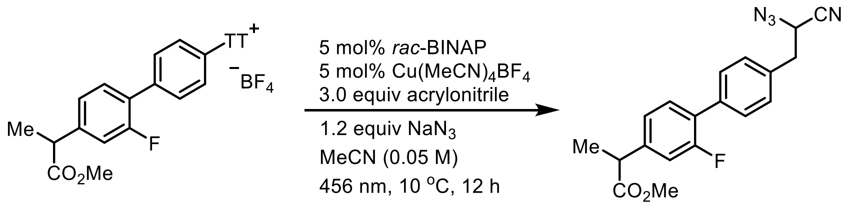


**TT-12** **12**, 71% yield

To a 4-mL borosilicate vial containing a Teflon-coated magnetic stirring bar were added **TT-12** (55.8 mg, 0.100 mmol, 1.00 equiv), *rac*-BINAP (3.1 mg, 5.0 μmol, 5.0 mol%), and sodium azide (7.8 mg, 0.12 mmol, 1.2 equiv). The vial was transferred into a nitrogen-filled glovebox. $Cu(MeCN)_4BF_4$ (1.6 mg, 5.0 μmol, 5.0 mol%), dry MeCN (2 mL, c = 0.05 M), and acrylonitrile (16 mg, 20 μL, 0.30 mmol, 3.0 equiv) were added. The vial

was sealed with a Teflon-lined screw cap and transferred to a cryocooler precooled at 10 °C where the reaction mixture was stirred for 5 min without irradiation and then irradiated by 456 nm LEDs for 12 h. Subsequently, silica gel (5–10 mL) was added, and the reaction mixture was concentrated to dryness under reduced pressure. The resulting residue was purified by chromatography on silica gel (hexanes/EtOAc = 10:1) to afford **12** as a colorless oil in 71% yield (24.9 mg).

**R*f*** = 0.23 (hexanes/EtOAc = 10:1).

**NMR Spectroscopy:**

**$^1$H NMR** (500 MHz, $CDCl_3$, 23 ºC, δ): 7.57 (dd, *J* = 8.2, 1.7 Hz, 2H), 7.41 (t, *J* = 8.0 Hz, 1H), 7.38 (d, *J* = 8.2 Hz, 2H), 7.18 (dd, *J* = 7.9, 1.8 Hz, 1H), 7.15 (dd, *J* = 11.5, 1.7 Hz, 1H), 4.43 (t, *J* = 7.1 Hz, 1H), 3.79 (q, *J* = 7.2 Hz, 1H), 3.73 (s, 3H), 3.24 – 3.12 (m, 2H), 1.56 (d, *J* = 7.2 Hz, 3H).

**$^{13}$C NMR** (125 MHz, $CDCl_3$, 23 ºC, δ): 174.4, 159.7 (d, *J* = 248.6 Hz), 142.2 (d, *J* = 7.7 Hz), 135.4, 132.9, 130.7 (d, *J* = 4.0 Hz), 129.6, 129.5 (d, *J* = 3.1 Hz), 127.0 (d, *J* = 13.7 Hz), 123.6 (d, *J* = 3.5 Hz), 115.8, 115.3 (d, *J* = 23.7 Hz), 52.4, 52.2, 44.9, 38.8, 18.4.

**$^{19}$F NMR** (470 MHz, $CDCl_3$, 23 ºC, δ): –117.46 (dd, *J* = 11.4, 8.3 Hz).

**HRMS-ESIpos (m/z)** calc'd for $C_{19}H_{17}N_4O_2FNa$ $[M+Na]^+$, 375.1228; found, 375.1229; deviation: –0.3 ppm.

**Tocopherol-derived phenylalanine analogue 13**

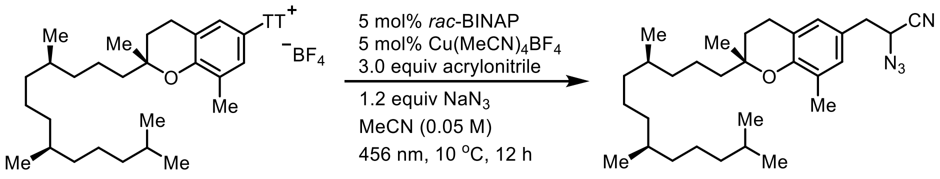


**TT-13** **13**, 56% yield

To a 4-mL borosilicate vial containing a Teflon-coated magnetic stirring bar were added **TT-13** (68.8 mg, 0.100 mmol, 1.00 equiv), *rac*-BINAP (3.1 mg, 5.0 μmol, 5.0 mol%), and sodium azide (7.8 mg, 0.12 mmol, 1.2 equiv). The vial was transferred into a nitrogen-filled glovebox. $Cu(MeCN)_4BF_4$ (1.6 mg, 5.0 μmol, 5.0 mol%), dry MeCN (2 mL, c = 0.05 M), and acrylonitrile (16 mg, 20 μL, 0.30 mmol, 3.0 equiv) were added. The vial was sealed with a Teflon-lined screw cap and transferred to a cryocooler precooled at 10 °C where the reaction mixture was stirred for 5 min without irradiation and then irradiated by 456 nm LEDs for 12 h. Subsequently, silica gel (5–10 mL) was added, and the reaction mixture was concentrated to dryness under reduced pressure. The resulting residue was purified by chromatography on silica gel (hexanes/EtOAc = 50:1) to afford **13** as a colorless oil in 56% yield (26.9 mg).

**R*f*** = 0.13 (hexanes/EtOAc = 50:1).

**NMR Spectroscopy:**

**$^1$H NMR** (500 MHz, $CDCl_3$, 23 ºC, δ): 6.86 (s, 1H), 6.83 (s, 1H), 4.29 (t, *J* = 7.2 Hz, 1H), 3.08 – 2.92 (m,

2H), 2.83 – 2.69 (m, 2H), 2.18 (s, 3H), 1.89 – 1.81 (m, 1H), 1.82 – 1.73 (m, 1H), 1.65 – 1.02 (m, 24H), 0.93 – 0.85 (m, 12H).

**$^{13}$C NMR** (125 MHz, $CDCl_3$, 23 ºC, δ): 152.0, 129.2, 127.8, 126.9, 123.5, 120.9, 116.1, 76.3, 52.9, 40.2, 39.4, 38.5, 37.5, 37.4, 37.3, 32.8, 32.7, 31.1, 28.0, 24.8, 24.5, 24.2, 22.7, 22.6, 22.3, 21.0, 19.8, 19.7, 16.1.

**HRMS-ESIpos (m/z)** calc'd for $C_{30}H_{48}N_4ONa$ $[M+Na]^+$, 503.3720; found, 503.3721; deviation: –0.1 ppm.

***rac*-Quinoline-derived phenylalanine analogue 14**

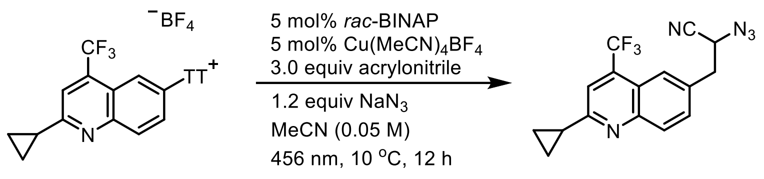


**TT-14** *rac*-**14**, 45% yield

To a 4-mL borosilicate vial containing a Teflon-coated magnetic stirring bar were added **TT-14** (58.2 mg, 0.100 mmol, 1.00 equiv), *rac*-BINAP (3.1 mg, 5.0 µmol, 5.0 mol%), and sodium azide (7.8 mg, 0.12 mmol, 1.2 equiv). The vial was transferred into a nitrogen-filled glovebox. $Cu(MeCN)_4BF_4$ (1.6 mg, 5.0 µmol, 5.0 mol%), dry MeCN (2 mL, c = 0.05 M), and acrylonitrile (16 mg, 20 µL, 0.30 mmol, 3.0 equiv) were added. The vial was sealed with a Teflon-lined screw cap and transferred to a cryocooler precooled at 10 °C where the reaction mixture was stirred for 5 min without irradiation and then irradiated by 456 nm LEDs for 12 h. Subsequently, silica gel (5–10 mL) was added, and the reaction mixture was concentrated to dryness under reduced pressure. The resulting residue was purified by chromatography on silica gel (hexanes/DCM = 1:2) to afford *rac*-**14** as a colorless oil in 45% yield (17.3 mg).

**$R_f$** = 0.21 (hexanes/DCM = 1:2).

**NMR Spectroscopy:**

**$^1$H NMR** (500 MHz, $CDCl_3$, 23 ºC, δ): 8.07 (d, *J* = 8.6 Hz, 1H), 7.97 (s, 1H), 7.66 (dd, *J* = 8.7, 1.9 Hz, 1H), 7.28 (s, 1H), 4.50 (t, *J* = 7.0 Hz, 1H), 3.33 (dd, *J* = 7.0, 2.9 Hz, 2H), 2.30 (tt, *J* = 8.4, 4.7 Hz, 1H), 1.27 (dt, *J* = 6.4, 4.0 Hz, 2H), 1.19 (dt, *J* = 8.4, 3.3 Hz, 2H).

**$^{13}$C NMR** (125 MHz, $CDCl_3$, 23 ºC, δ): 163.7, 148.4, 133.7 (q, *J* = 31.1 Hz), 132.0, 131.1, 130.4, 124.7 (q, *J* = 2.4 Hz), 123.4 (q, *J* = 274.8 Hz), 121.3, 117.8 (q, *J* = 5.4 Hz), 115.5, 52.3, 39.2, 18.2, 11.2.

**$^{19}$F NMR** (470 MHz, $CDCl_3$, 23 ºC, δ): –61.47.

**HRMS-ESIpos (m/z)** calc'd for $C_{16}H_{13}N_5F_3$ $[M+H]^+$, 332.1117; found, 332.1118; deviation: –0.4 ppm.

***rac*-Niflumic acid-derived phenylalanine analogue 15**

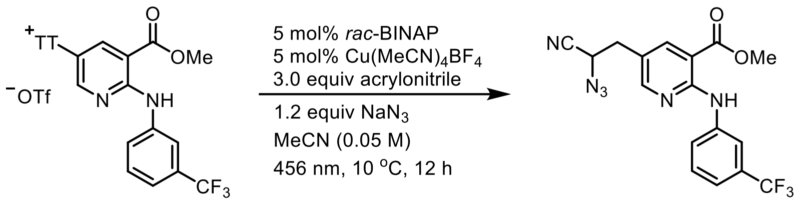


**TT-15** *rac*-**15**, 40% yield

To a 4-mL borosilicate vial containing a Teflon-coated magnetic stirring bar were added **TT-15** (66.0 mg, 0.100 mmol, 1.00 equiv), *rac*-BINAP (3.1 mg, 5.0 μmol, 5.0 mol%), and sodium azide (7.8 mg, 0.12 mmol, 1.2 equiv). The vial was transferred into a nitrogen-filled glovebox. $Cu(MeCN)_4BF_4$ (1.6 mg, 5.0 μmol, 5.0 mol%), dry MeCN (2 mL, c = 0.05 M), and acrylonitrile (16 mg, 20 μL, 0.30 mmol, 3.0 equiv) were added. The vial was sealed with a Teflon-lined screw cap and transferred to a cryocooler precooled at 10 °C where the reaction mixture was stirred for 5 min without irradiation and then irradiated by 456 nm LEDs for 12 h. Subsequently, silica gel (5–10 mL) was added, and the reaction mixture was concentrated to dryness under reduced pressure. The resulting residue was purified by chromatography on silica gel (hexanes/EtOAc = 4:1) to afford *rac*-**15** as a colorless oil in 40% yield (15.1 mg).

**R*f*** = 0.20 (hexanes/EtOAc = 4:1).

**NMR Spectroscopy:**

**$^1$H NMR** (500 MHz, $CDCl_3$, 23 ºC, δ): 10.41 (s, 1H), 8.36 (d, *J* = 2.5 Hz, 1H), 8.23 (d, *J* = 2.5 Hz, 1H), 8.11 (s, 1H), 7.88 (d, *J* = 8.2 Hz, 1H), 7.47 (t, *J* = 8.0 Hz, 1H), 7.33 (d, *J* = 7.7 Hz, 1H), 4.43 (t, *J* = 6.7 Hz, 1H), 3.99 (s, 3H), 3.08 (dd, *J* = 6.7, 2.4 Hz, 2H).

**$^{13}$C NMR** (125 MHz, $CDCl_3$, 23 ºC, δ): 167.5, 155.4, 153.7, 141.1, 140.0, 131.2 (q, *J* = 32.2 Hz), 129.2, 124.1 (q, *J* = 272.4 Hz), 123.6, 119.4 (q, *J* = 3.9 Hz), 119.0, 117.3 (q, *J* = 4.0 Hz), 115.4, 107.3, 52.6, 52.2, 35.4.

**$^{19}$F NMR** (470 MHz, $CDCl_3$, 23 ºC, δ): –62.64.

**HRMS-ESIneg (m/z)** calc'd for $C_{17}H_{12}N_6O_5F_3$ $[M–H]^–$, 389.0983; found, 389.0984; deviation: –1.3 ppm.

***rac*-Boscalid-derived phenylalanine analogue 16**

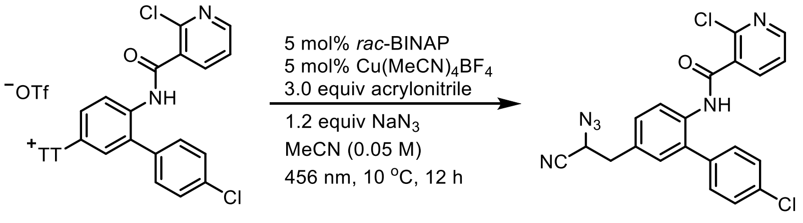


**TT-16** *rac*-**16**, 69% yield

To a 4-mL borosilicate vial containing a Teflon-coated magnetic stirring bar were added **TT-16** (70.7 mg,

0.100 mmol, 1.00 equiv), *rac*-BINAP (3.1 mg, 5.0 μmol, 5.0 mol%), and sodium azide (7.8 mg, 0.12 mmol, 1.2 equiv). The vial was transferred into a nitrogen-filled glovebox. $Cu(MeCN)_4BF_4$ (1.6 mg, 5.0 μmol, 5.0 mol%), dry MeCN (2 mL, c = 0.05 M), and acrylonitrile (16 mg, 20 μL, 0.30 mmol, 3.0 equiv) were added. The vial was sealed with a Teflon-lined screw cap and transferred to a cryocooler precooled at 10 °C where the reaction mixture was stirred for 5 min without irradiation and then irradiated by 456 nm LEDs for 12 h. Subsequently, silica gel (5–10 mL) was added, and the reaction mixture was concentrated to dryness under reduced pressure. The resulting residue was purified by chromatography on silica gel (hexanes/EtOAc = 2:1) to afford *rac*-**16** as a colorless oil in 69% yield (30.3 mg).

**$R_f$** = 0.17 (hexanes/EtOAc = 2:1).

**NMR Spectroscopy:**

**$^1$H NMR** (500 MHz, $CDCl_3$, 23 ºC, δ): 8.51 – 8.45 (m, 2H), 8.24 (s, 1H), 8.16 (dd, *J* = 7.6, 2.0 Hz, 1H), 7.47 (d, *J* = 8.4 Hz, 2H), 7.41 – 7.34 (m, 4H), 7.22 (d, *J* = 2.2 Hz, 1H), 4.45 (t, *J* = 6.9 Hz, 1H), 3.16 (d, *J* = 6.9 Hz, 2H).

**$^{13}$C NMR** (125 MHz, $CDCl_3$, 23 ºC, δ): 162.5, 151.4, 146.6, 140.2, 135.5, 134.8, 134.4, 132.6, 131.3, 130.9, 130.8, 130.3, 129.9, 129.5, 123.0, 122.4, 115.6, 52.4, 38.4.

**HRMS-ESIpos (m/z)** calc'd for $C_{21}H_{14}ON_6Cl_2Na$ $[M+H]^+$, 459.0498; found, 459.0497; deviation: +0.3 ppm.

**Pyriproxyphen-derived phenylalanine analogue 17**

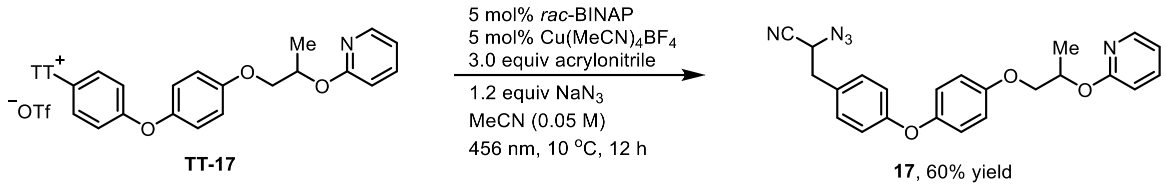


To a 4-mL borosilicate vial containing a Teflon-coated magnetic stirring bar were added **TT-17** (68.5 mg, 0.100 mmol, 1.00 equiv), *rac*-BINAP (3.1 mg, 5.0 μmol, 5.0 mol%), and sodium azide (7.8 mg, 0.12 mmol, 1.2 equiv). The vial was transferred into a nitrogen-filled glovebox. $Cu(MeCN)_4BF_4$ (1.6 mg, 5.0 μmol, 5.0 mol%), dry MeCN (2 mL, c = 0.05 M), and acrylonitrile (16 mg, 20 μL, 0.30 mmol, 3.0 equiv) were added. The vial was sealed with a Teflon-lined screw cap and transferred to a cryocooler precooled at 10 °C where the reaction mixture was stirred for 5 min without irradiation and then irradiated by 456 nm LEDs for 12 h. Subsequently, silica gel (5–10 mL) was added, and the reaction mixture was concentrated to dryness under reduced pressure. The resulting residue was purified by chromatography on silica gel (hexanes/EtOAc = 10:1) to afford **17** as a colorless oil in 60% yield (25.0 mg).

**$R_f$** = 0.20 (hexanes/EtOAc = 10:1).

**NMR Spectroscopy:**

**$^1$H NMR** (500 MHz, $CDCl_3$, 23 ºC, δ): 8.18 (dd, *J* = 5.3, 1.9 Hz, 1H), 7.59 (ddd, *J* = 8.5, 7.1, 2.0 Hz, 1H),

7.22 (d, *J* = 8.6 Hz, 2H), 7.02 – 6.91 (m, 6H), 6.89 (ddd, *J* = 7.1, 5.1, 1.0 Hz, 1H), 6.77 (d, *J* = 8.4 Hz, 1H), 5.66 – 5.56 (m, 1H), 4.34 (t, *J* = 7.0 Hz, 1H), 4.22 (dd, *J* = 9.9, 5.3 Hz, 1H), 4.10 (dd, *J* = 9.9, 4.9 Hz, 1H), 3.10 (dd, *J* = 7.0, 1.6 Hz, 2H), 1.51 (d, *J* = 6.4 Hz, 3H).

**$^{13}$C NMR** (125 MHz, $CDCl_3$, 23 ºC, δ): 163.1, 158.6, 155.5, 149.7, 146.8, 138.7, 130.7, 127.2, 121.0, 117.8, 116.8, 115.9, 115.8, 111.7, 71.1, 69.3, 52.6, 38.3, 17.0.

**HRMS-ESIpos (m/z)** calc'd for $C_{23}H_{22}N_5O_3$ [M+H]$^+$, 416.1717; found, 416.1719; deviation: –0.4 ppm.

***rac*-Fenofibrate-derived phenylalanine analogue 18**

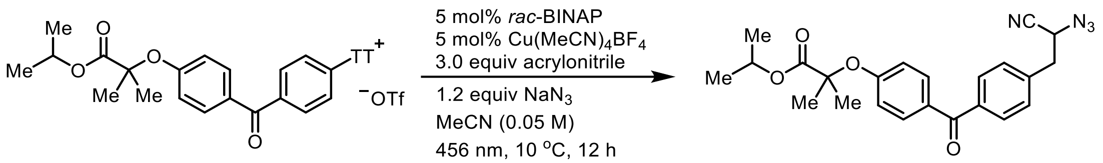


**TT-18** *rac*-**18**, 48% yield

To a 4-mL borosilicate vial containing a Teflon-coated magnetic stirring bar were added **TT-18** (64.5 mg, 0.100 mmol, 1.00 equiv), *rac*-BINAP (3.1 mg, 5.0 μmol, 5.0 mol%), and sodium azide (7.8 mg, 0.12 mmol, 1.2 equiv). The vial was transferred into a nitrogen-filled glovebox. $Cu(MeCN)_4BF_4$ (1.6 mg, 5.0 μmol, 5.0 mol%), dry MeCN (2 mL, c = 0.05 M), and acrylonitrile (16 mg, 20 μL, 0.30 mmol, 3.0 equiv) were added. The vial was sealed with a Teflon-lined screw cap and transferred to a cryocooler precooled at 10 °C where the reaction mixture was stirred for 5 min without irradiation and then irradiated by 456 nm LEDs for 12 h. Subsequently, silica gel (5–10 mL) was added, and the reaction mixture was concentrated to dryness under reduced pressure. The resulting residue was purified by chromatography on silica gel (hexanes/EtOAc = 5:1) to afford *rac*-**18** as a colorless oil in 48% yield (18.1 mg).

**R*f*** = 0.18 (hexanes/EtOAc = 5:1).

**NMR Spectroscopy:**

**$^{1}$H NMR** (500 MHz, $CDCl_3$, 23 ºC, δ): 7.79 (d, *J* = 2.5 Hz, 2H), 7.77 (d, *J* = 3.1 Hz, 2H), 7.42 (d, *J* = 8.2 Hz, 2H), 6.89 (d, *J* = 8.8 Hz, 2H), 5.11 (hept, *J* = 6.3 Hz, 1H), 4.47 (t, *J* = 7.0 Hz, 1H), 3.29 – 3.15 (m, 2H), 1.68 (s, 6H), 1.22 (d, *J* = 6.3 Hz, 6H).

**$^{13}$C NMR** (125 MHz, $CDCl_3$, 23 ºC, δ): 194.8, 173.1, 159.7, 138.0, 137.4, 132.0, 130.4, 130.3, 129.4, 117.2, 115.5, 79.4, 69.3, 52.1, 38.9, 25.4, 21.5.

**HRMS-ESIpos (m/z)** calc'd for $C_{23}H_{25}O_4N_4$ [M+H]$^+$, 421.1870; found, 421.1872; deviation: –0.4 ppm.

***rac*-Thyronine precursor 19**

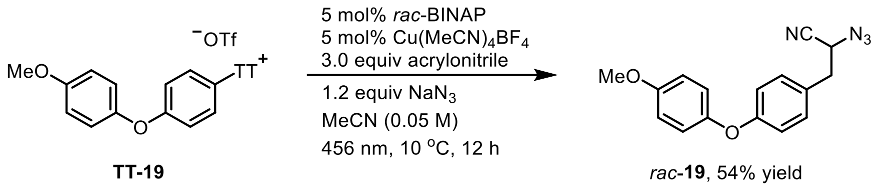


To a 4-mL borosilicate vial containing a Teflon-coated magnetic stirring bar were added **TT-19** (56.5 mg, 0.100 mmol, 1.00 equiv), *rac*-BINAP (3.1 mg, 5.0 μmol, 5.0 mol%), and sodium azide (7.8 mg, 0.12 mmol, 1.2 equiv). The vial was transferred into a nitrogen-filled glovebox. $Cu(MeCN)_4BF_4$ (1.6 mg, 5.0 μmol, 5.0 mol%), dry MeCN (2 mL, c = 0.05 M), and acrylonitrile (16 mg, 20 μL, 0.30 mmol, 3.0 equiv) were added. The vial was sealed with a Teflon-lined screw cap and transferred to a cryocooler precooled at 10 °C where the reaction mixture was stirred for 5 min without irradiation and then irradiated by 456 nm LEDs for 12 h. Subsequently, silica gel (5–10 mL) was added, and the reaction mixture was concentrated to dryness under reduced pressure. The resulting residue was purified by chromatography on silica gel (hexanes/EtOAc = 5:1) to afford *rac*-**19** as a colorless oil in 54% yield (16.2 mg).

**R*f*** = 0.28 (hexanes/EtOAc = 5:1).

**NMR Spectroscopy:**

**$^1$H NMR** (500 MHz, $CDCl_3$, 23 ºC, δ): 7.23 (d, *J* = 8.6 Hz, 2H), 7.01 (d, *J* = 9.0 Hz, 2H), 6.95 (d, *J* = 8.6 Hz, 2H), 6.92 (d, *J* = 9.0 Hz, 2H), 4.35 (t, *J* = 7.0 Hz, 1H), 3.84 (s, 3H), 3.10 (dd, *J* = 7.0, 1.4 Hz, 2H).

**$^{13}$C NMR** (125 MHz, $CDCl_3$, 23 ºC, δ): 158.7, 156.2, 149.6, 130.7, 127.2, 121.1, 117.8, 115.8, 114.9, 55.7, 52.6, 38.3.

**HRMS-ESIpos (m/z)** calc'd for $C_{16}H_{14}N_4O_2Na$ $[M+Na]^+$, 317.1009; found, 317.1011; deviation: –0.6 ppm.

***rac*-Dopa precursor 20**

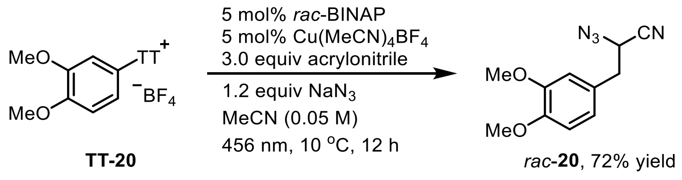


To a 4-mL borosilicate vial containing a Teflon-coated magnetic stirring bar were added **TT-20** (44.0 mg, 0.100 mmol, 1.00 equiv), *rac*-BINAP (3.1 mg, 5.0 μmol, 5.0 mol%), and sodium azide (7.8 mg, 0.12 mmol, 1.2 equiv). The vial was transferred into a nitrogen-filled glovebox. $Cu(MeCN)_4BF_4$ (1.6 mg, 5.0 μmol, 5.0 mol%), dry MeCN (2 mL, c = 0.05 M), and acrylonitrile (16 mg, 20 μL, 0.30 mmol, 3.0 equiv) were added. The vial was sealed with a Teflon-lined screw cap and transferred to a cryocooler precooled at 10 °C where the reaction mixture was stirred for 5 min without irradiation and then irradiated by 456 nm LEDs for 12 h. Subsequently, silica gel (5–10 mL) was added, and the reaction mixture was concentrated to dryness under

reduced pressure. The resulting residue was purified by chromatography on silica gel (hexanes/EtOAc = 5:1) to afford *rac*-**20** as a colorless oil in 72% yield (16.6 mg).

**R*f*** = 0.22 (hexanes/EtOAc = 5:1).

**NMR Spectroscopy:**

**$^{1}$H NMR** (500 MHz, $CDCl_3$, 23 ºC, δ): 6.89 – 6.84 (m, 2H), 6.81 (d, *J* = 1.8 Hz, 1H), 4.35 (t, *J* = 7.0 Hz, 1H), 3.91 (s, 3H), 3.90 (s, 3H), 3.08 (dd, *J* = 7.0, 1.3 Hz, 2H).

**$^{13}$C NMR** (125 MHz, $CDCl_3$, 23 ºC, δ): 149.2, 149.0, 125.8, 121.8, 115.9, 112.5, 111.5, 56.0, 55.9, 52.7, 38.8.

**HRMS-EI (m/z)** calc'd for $C_{11}H_{12}O_2N_4$ $[M]^+$, 232.0955; found, 232.0956; deviation: –0.4 ppm.

***rac*-Xylariamide A precursor 21**

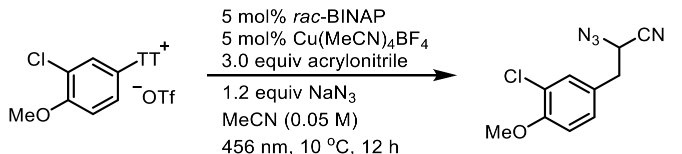


**TT-21** *rac*-**21**, 72% yield

To a 4-mL borosilicate vial containing a Teflon-coated magnetic stirring bar were added **TT-21** (50.6 mg, 0.100 mmol, 1.00 equiv), *rac*-BINAP (3.1 mg, 5.0 μmol, 5.0 mol%), and sodium azide (7.8 mg, 0.12 mmol, 1.2 equiv). The vial was transferred into a nitrogen-filled glovebox. $Cu(MeCN)_4BF_4$ (1.6 mg, 5.0 μmol, 5.0 mol%), dry MeCN (2 mL, c = 0.05 M), and acrylonitrile (16 mg, 20 μL, 0.30 mmol, 3.0 equiv) were added. The vial was sealed with a Teflon-lined screw cap and transferred to a cryocooler precooled at 10 °C where the reaction mixture was stirred for 5 min without irradiation and then irradiated by 456 nm LEDs for 12 h. Subsequently, silica gel (5–10 mL) was added, and the reaction mixture was concentrated to dryness under reduced pressure. The resulting residue was purified by chromatography on silica gel (hexanes/EtOAc = 5:1) to afford *rac*-**21** as a colorless oil in 72% yield (17.2 mg).

**R*f*** = 0.30 (hexanes/EtOAc = 5:1).

**NMR Spectroscopy:**

**$^{1}$H NMR** (500 MHz, $CDCl_3$, 23 ºC, δ): 7.31 (d, *J* = 2.3 Hz, 1H), 7.18 (dd, *J* = 8.5, 2.3 Hz, 1H), 6.94 (d, *J* = 8.4 Hz, 1H), 4.36 (t, *J* = 7.0 Hz, 1H), 3.93 (s, 3H), 3.06 (dd, *J* = 7.0, 1.7 Hz, 2H).

**$^{13}$C NMR** (125 MHz, $CDCl_3$, 23 ºC, δ): 155.0, 131.1, 128.9, 126.4, 122.9, 115.6, 112.3, 56.2, 52.5, 37.9.

**HRMS-EI (m/z)** calc'd for $C_{10}H_9N_4OCl$ $[M]^+$, 236.0459; found, 236.0464; deviation: –1.9 ppm.

### *rac*-Fenclonine precursor 22

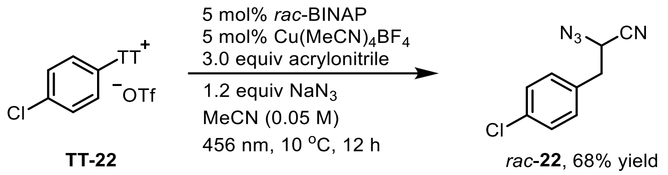


To a 4-mL borosilicate vial containing a Teflon-coated magnetic stirring bar were added **TT-22** (47.6 mg, 0.100 mmol, 1.00 equiv), *rac*-BINAP (3.1 mg, 5.0 μmol, 5.0 mol%), and sodium azide (7.8 mg, 0.12 mmol, 1.2 equiv). The vial was transferred into a nitrogen-filled glovebox. $Cu(MeCN)_4BF_4$ (1.6 mg, 5.0 μmol, 5.0 mol%), dry MeCN (2 mL, c = 0.05 M), and acrylonitrile (16 mg, 20 μL, 0.30 mmol, 3.0 equiv) were added. The vial was sealed with a Teflon-lined screw cap and transferred to a cryocooler precooled at 10 °C where the reaction mixture was stirred for 5 min without irradiation and then irradiated by 456 nm LEDs for 12 h. Subsequently, silica gel (5–10 mL) was added, and the reaction mixture was concentrated to dryness under reduced pressure. The resulting residue was purified by chromatography on silica gel (hexanes/EtOAc = 20:1) to afford *rac*-**22** as a colorless oil in 68% yield (14.0 mg).

**R*f*** = 0.22 (hexanes/EtOAc = 20:1).

**NMR Spectroscopy:**

$^{1}$**H NMR** (500 MHz, $CDCl_3$, 23 ºC, δ): 7.38 (d, *J* = 8.4 Hz, 2H), 7.25 (d, *J* = 8.2 Hz, 2H), 4.38 (t, *J* = 6.9 Hz, 1H), 3.11 (dd, *J* = 7.0, 2.0 Hz, 2H).

$^{13}$**C NMR** (125 MHz, $CDCl_3$, 23 ºC, δ): 134.3, 131.9, 130.8, 129.2, 115.5, 52.3, 38.4.

**HRMS-EI (m/z)** calc'd for $C_9H_7N_4Cl$ $[M]^+$, 206.0354; found, 206.0354; deviation: –0.4 ppm.

### *rac*-Phenylalanine analogue 23

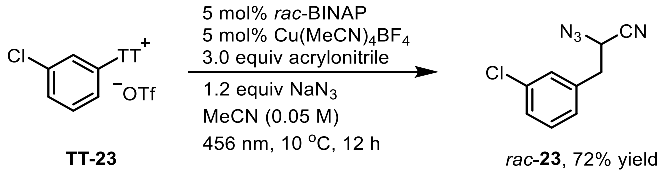


To a 4-mL borosilicate vial containing a Teflon-coated magnetic stirring bar were added **TT-23** (47.6 mg, 0.100 mmol, 1.00 equiv), *rac*-BINAP (3.1 mg, 5.0 μmol, 5.0 mol%), and sodium azide (7.8 mg, 0.12 mmol, 1.2 equiv). The vial was transferred into a nitrogen-filled glovebox. $Cu(MeCN)_4BF_4$ (1.6 mg, 5.0 μmol, 5.0 mol%), dry MeCN (2 mL, c = 0.05 M), and acrylonitrile (16 mg, 20 μL, 0.30 mmol, 3.0 equiv) were added. The vial was sealed with a Teflon-lined screw cap and transferred to a cryocooler precooled at 10 °C where the reaction mixture was stirred for 5 min without irradiation and then irradiated by 456 nm LEDs for 12 h. Subsequently, silica gel (5–10 mL) was added, and the reaction mixture was concentrated to dryness under reduced pressure. The resulting residue was purified by chromatography on silica gel (hexanes/EtOAc = 20:1) to afford *rac*-**23** as a colorless oil in 75% yield (15.4 mg).

**R*f*** = 0.23 (hexanes/EtOAc = 20:1).

**NMR Spectroscopy:**

**$^1$H NMR** (500 MHz, $CDCl_3$, 23 ºC, δ): 7.37 – 7.32 (m, 2H), 7.30 (s, 1H), 7.23 – 7.18 (m, 1H), 4.41 (t, *J* = 7.0 Hz, 1H), 3.20 – 3.04 (m, 2H).

**$^{13}$C NMR** (125 MHz, $CDCl_3$, 23 ºC, δ): 135.4, 134.8, 130.3, 129.6, 128.5, 127.7, 115.5, 52.2, 38.6.

**HRMS-ESIpos (m/z)** calc'd for $C_9H_7ClN_4Na$ $[M+Na]^+$, 229.0251; found, 229.0253; deviation: –0.5 ppm.

***rac*-Phenylalanine analogue 24**

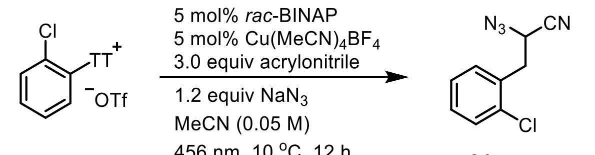


**TT-24** *rac*-**24**, 73% yield

To a 4-mL borosilicate vial containing a Teflon-coated magnetic stirring bar were added **TT-24** (47.6 mg, 0.100 mmol, 1.00 equiv), *rac*-BINAP (3.1 mg, 5.0 μmol, 5.0 mol%), and sodium azide (7.8 mg, 0.12 mmol, 1.2 equiv). The vial was transferred into a nitrogen-filled glovebox. $Cu(MeCN)_4BF_4$ (1.6 mg, 5.0 μmol, 5.0 mol%), dry MeCN (2 mL, c = 0.05 M), and acrylonitrile (16 mg, 20 μL, 0.30 mmol, 3.0 equiv) were added. The vial was sealed with a Teflon-lined screw cap and transferred to a cryocooler precooled at 10 °C where the reaction mixture was stirred for 5 min without irradiation and then irradiated by 456 nm LEDs for 12 h. Subsequently, silica gel (5–10 mL) was added, and the reaction mixture was concentrated to dryness under reduced pressure. The resulting residue was purified by chromatography on silica gel (hexanes/EtOAc = 20:1) to afford *rac*-**24** as a colorless oil in 73% yield (15.1 mg).

**R*f*** = 0.20 (hexanes/EtOAc = 20:1).

**NMR Spectroscopy:**

**$^1$H NMR** (500 MHz, $CDCl_3$, 23 ºC, δ): 7.47 – 7.42 (m, 1H), 7.39 – 7.30 (m, 3H), 4.55 (t, *J* = 7.6 Hz, 1H), 3.40 – 3.18 (m, 2H).

**$^{13}$C NMR** (125 MHz, $CDCl_3$, 23 ºC, δ): 134.1, 132.1, 131.3, 129.9, 129.8, 127.4, 115.7, 50.6, 37.1.

**HRMS-ESIpos (m/z)** calc'd for $C_9H_7ClN_4Na$ $[M+Na]^+$, 229.0251; found, 229.0253; deviation: –0.6 ppm.

***rac*-Thianaphthene alanine analogue 25**

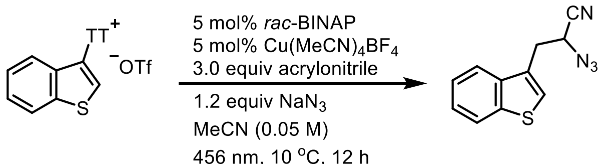


**TT-25** *rac*-**25**, 56% yield

To a 4-mL borosilicate vial containing a Teflon-coated magnetic stirring bar were added **TT-25** (49.8 mg, 0.100 mmol, 1.00 equiv), *rac*-BINAP (3.1 mg, 5.0 μmol, 5.0 mol%), and sodium azide (7.8 mg, 0.12 mmol, 1.2 equiv). The vial was transferred into a nitrogen-filled glovebox. $Cu(MeCN)_4BF_4$ (1.6 mg, 5.0 μmol, 5.0 mol%), dry MeCN (2 mL, c = 0.05 M), and acrylonitrile (16 mg, 20 μL, 0.30 mmol, 3.0 equiv) were added. The vial was sealed with a Teflon-lined screw cap and transferred to a cryocooler precooled at 10 °C where the reaction mixture was stirred for 5 min without irradiation and then irradiated by 456 nm LEDs for 12 h. Subsequently, silica gel (5–10 mL) was added, and the reaction mixture was concentrated to dryness under reduced pressure. The resulting residue was purified by chromatography on silica gel (hexanes/EtOAc = 10:1) to afford *rac*-**25** as a colorless oil in 56% yield (13.2 mg).

**R*f*** = 0.24 (hexanes/EtOAc = 10:1).

**NMR Spectroscopy:**

**$^1$H NMR** (500 MHz, $CDCl_3$, 23 ºC, δ): 7.93 (d, *J* = 7.7 Hz, 1H), 7.75 (d, *J* = 7.5 Hz, 1H), 7.51 – 7.40 (m, 3H), 4.53 (t, *J* = 7.1 Hz, 1H), 3.50 – 3.32 (m, 2H).

**$^{13}$C NMR** (125 MHz, $CDCl_3$, 23 ºC, δ): 140.5, 137.8, 127.9, 126.1, 124.8, 124.5, 123.2, 120.9, 115.9, 51.2, 32.0.

**HRMS-EI (m/z)** calc'd for $C_{11}H_8N_4S$ $[M]^+$, 228.0464; found, 228.0464; deviation: –0.1 ppm.

### *rac*-Pyrrole alanine analogue 26

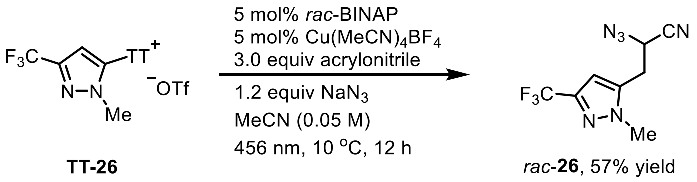


To a 4-mL borosilicate vial containing a Teflon-coated magnetic stirring bar were added **TT-26** (51.4 mg, 0.100 mmol, 1.00 equiv), *rac*-BINAP (3.1 mg, 5.0 μmol, 5.0 mol%), and sodium azide (7.8 mg, 0.12 mmol, 1.2 equiv). The vial was transferred into a nitrogen-filled glovebox. $Cu(MeCN)_4BF_4$ (1.6 mg, 5.0 μmol, 5.0 mol%), dry MeCN (2 mL, c = 0.05 M), and acrylonitrile (16 mg, 20 μL, 0.30 mmol, 3.0 equiv) were added. The vial was sealed with a Teflon-lined screw cap and transferred to a cryocooler precooled at 10 °C where the reaction mixture was stirred for 5 min without irradiation and then irradiated by 456 nm LEDs for 12 h. Subsequently, silica gel (5–10 mL) was added, and the reaction mixture was concentrated to dryness under reduced pressure. The resulting residue was purified by chromatography on silica gel (hexanes/EtOAc = 3:1) to afford *rac*-**26** as a colorless oil in 57% yield (14.1 mg).

**R*f*** = 0.18 (hexanes/EtOAc = 3:1).

**NMR Spectroscopy:**

**$^1$H NMR** (500 MHz, $CDCl_3$, 23 ºC, δ): 6.56 (s, 1H), 4.55 (t, *J* = 6.5 Hz, 1H), 3.96 (s, 3H), 3.29 – 3.11 (m,

2H).

**$^{13}$C NMR** (125 MHz, $CDCl_3$, 23 ºC, δ): 141.8 (q, *J* = 38.7 Hz), 136.4, 121.0 (q, *J* = 268.3 Hz), 114.9, 105.1, 50.6, 37.4, 29.1.

**$^{19}$F NMR** (470 MHz, $CDCl_3$, 23 ºC, δ): -62.22.

**HRMS-EI (m/z)** calc'd for $C_8H_7N_6F_3$ $[M]^+$, 244.0679; found, 244.0676; deviation: +1.3 ppm.

***rac*-Isoquinoline alanine analogue 27**

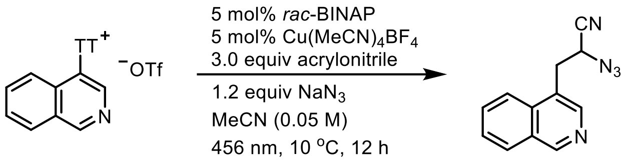


**TT-27** *rac*-**27**, 68% yield

To a 4-mL borosilicate vial containing a Teflon-coated magnetic stirring bar were added **TT-27** (49.3 mg, 0.100 mmol, 1.00 equiv), *rac*-BINAP (3.1 mg, 5.0 µmol, 5.0 mol%), and sodium azide (7.8 mg, 0.12 mmol, 1.2 equiv). The vial was transferred into a nitrogen-filled glovebox. $Cu(MeCN)_4BF_4$ (1.6 mg, 5.0 µmol, 5.0 mol%), dry MeCN (2 mL, c = 0.05 M), and acrylonitrile (16 mg, 20 µL, 0.30 mmol, 3.0 equiv) were added. The vial was sealed with a Teflon-lined screw cap and transferred to a cryocooler precooled at 10 °C where the reaction mixture was stirred for 5 min without irradiation and then irradiated by 456 nm LEDs for 12 h. Subsequently, silica gel (5–10 mL) was added, and the reaction mixture was concentrated to dryness under reduced pressure. The resulting residue was purified by chromatography on silica gel (hexanes/EtOAc = 1:1) to afford *rac*-**27** as a colorless oil in 63% yield (14.2 mg).

**R*f*** = 0.20 (hexanes/EtOAc = 1:1).

**NMR Spectroscopy:**

**$^{1}$H NMR** (500 MHz, $C_6D_6$, 23 ºC, δ): 9.04 (s, 1H), 8.31 (s, 1H), 7.37 (d, *J* = 8.1 Hz, 1H), 7.22 (d, *J* = 3.8 Hz, 2H), 7.13 – 7.06 (m, 1H), 3.38 (t, *J* = 7.3 Hz, 1H), 2.87 – 2.52 (m, 1H).

**$^{13}$C NMR** (125 MHz, $C_6D_6$, 23 ºC, δ): 153.1, 144.9, 133.9, 130.3, 128.3, 126.8, 121.8, 115.4, 51.1, 32.3. Two signals were overlapped with $C_6D_6$.

**HRMS-EI** (m/z) calc'd for $C_{12}H_9N_5$ $[M]^+$, 223.0852; found, 223.0852; deviation: +0.3 ppm.

***rac*-Quinoline alanine analogue 28**

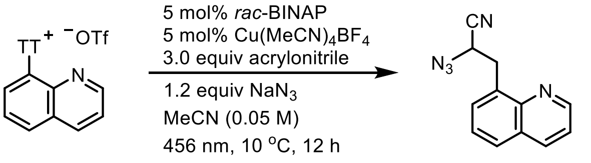


**TT-28** *rac*-**28**, 63% yield

To a 4-mL borosilicate vial containing a Teflon-coated magnetic stirring bar were added **TT-28** (49.3 mg, 0.100 mmol, 1.00 equiv), *rac*-BINAP (3.1 mg, 5.0 μmol, 5.0 mol%), and sodium azide (7.8 mg, 0.12 mmol, 1.2 equiv). The vial was transferred into a nitrogen-filled glovebox. $Cu(MeCN)_4BF_4$ (1.6 mg, 5.0 μmol, 5.0 mol%), dry MeCN (2 mL, c = 0.05 M), and acrylonitrile (16 mg, 20 μL, 0.30 mmol, 3.0 equiv) were added. The vial was sealed with a Teflon-lined screw cap and transferred to a cryocooler precooled at 10 °C where the reaction mixture was stirred for 5 min without irradiation and then irradiated by 456 nm LEDs for 12 h. Subsequently, silica gel (5–10 mL) was added, and the reaction mixture was concentrated to dryness under reduced pressure. The resulting residue was purified by chromatography on silica gel (hexanes/EtOAc = 10:1) to afford *rac*-**28** as a colorless oil in 63% yield (14.1 mg).

**R*f*** = 0.22 (hexanes/EtOAc = 10:1).

**NMR Spectroscopy:**

**$^1$H NMR** (500 MHz, $CDCl_3$, 23 ºC, δ): 9.05 – 8.97 (m, 1H), 8.34 (d, *J* = 8.2 Hz, 1H), 7.95 – 7.88 (m, 1H), 7.78 (d, *J* = 7.0 Hz, 1H), 7.62 (t, *J* = 7.6 Hz, 1H), 7.57 (dd, *J* = 8.4, 4.3 Hz, 1H), 5.21 (t, *J* = 7.6 Hz, 1H), 3.90 (dd, *J* = 13.3, 7.4 Hz, 1H), 3.78 (dd, *J* = 13.2, 7.7 Hz, 1H).

**$^{13}$C NMR** (125 MHz, $CDCl_3$, 23 ºC, δ): 149.9, 146.4, 136.6, 132.1, 131.4, 128.5, 128.5, 126.5, 121.5, 116.6, 51.4, 36.3.

**HRMS-ESIpos (m/z)** calc'd for $C_{12}H_{10}N_5$ $[M+H]^+$, 224.0931; found, 224.0932; deviation: –0.6 ppm.

***rac*-Pyridine alanine analogue 29**

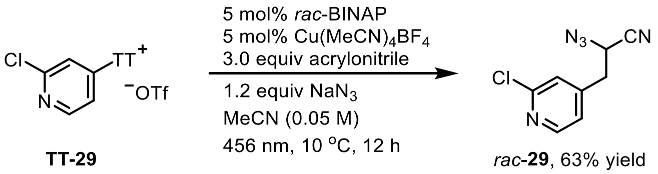


**TT-29** *rac*-**29**, 63% yield

To a 4-mL borosilicate vial containing a Teflon-coated magnetic stirring bar were added **TT-29** (47.7 mg, 0.100 mmol, 1.00 equiv), *rac*-BINAP (3.1 mg, 5.0 μmol, 5.0 mol%), and sodium azide (7.8 mg, 0.12 mmol, 1.2 equiv). The vial was transferred into a nitrogen-filled glovebox. $Cu(MeCN)_4BF_4$ (1.6 mg, 5.0 μmol, 5.0 mol%), dry MeCN (2 mL, c = 0.05 M), and acrylonitrile (16 mg, 20 μL, 0.30 mmol, 3.0 equiv) were added. The vial was sealed with a Teflon-lined screw cap and transferred to a cryocooler precooled at 10 °C where the reaction mixture was stirred for 5 min without irradiation and then irradiated by 456 nm LEDs for 12 h. Subsequently, silica gel (5–10 mL) was added, and the reaction mixture was concentrated to dryness under reduced pressure. The resulting residue was purified by chromatography on silica gel (hexanes/EtOAc = 4:1) to afford *rac*-**29** as a light yellow oil in 68% yield (13.9 mg).

**R*f*** = 0.18 (hexanes/EtOAc = 4:1).

**NMR Spectroscopy:**

**$^{1}$H NMR** (500 MHz, $CDCl_3$, 23 ºC, δ): 8.43 (d, *J* = 5.1 Hz, 1H), 7.30 (s, 1H), 7.19 (d, *J* = 5.0 Hz, 1H), 4.53 (t, *J* = 6.7 Hz, 1H), 3.24 – 3.00 (m, 2H).

**$^{13}$C NMR** (125 MHz, $CDCl_3$, 23 ºC, δ): 152.3, 150.3, 145.5, 125.1, 123.2, 115.0, 51.1, 37.7.

**HRMS-EI (m/z)** calc'd for $C_8H_6N_5Cl$ $[M]^+$, 207.0306; found, 207.0308; deviation: –1.0 ppm.

**Flubiprofen-derived phenylethylazide 30**

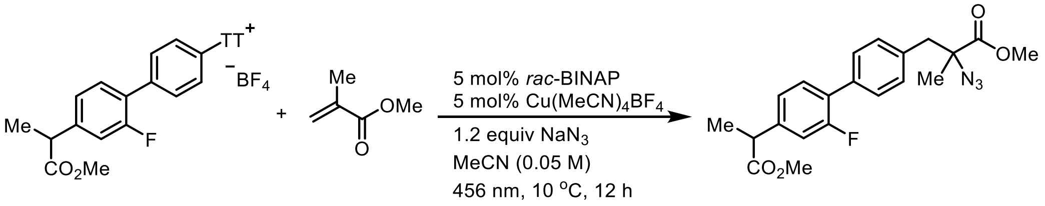


**TT-12** 3.0 equiv **30**, 50% yield

To a 4-mL borosilicate vial containing a Teflon-coated magnetic stirring bar were added **TT-12** (55.8 mg, 0.100 mmol, 1.00 equiv), *rac*-BINAP (3.1 mg, 5.0 μmol, 5.0 mol%), and sodium azide (7.8 mg, 0.12 mmol, 1.2 equiv). The vial was transferred into a nitrogen-filled glovebox. $Cu(MeCN)_4BF_4$ (1.6 mg, 5.0 μmol, 5.0 mol%), dry MeCN (2 mL, c = 0.05 M), and methyl methacrylate (30 mg, 32 μL, 0.30 mmol, 3.0 equiv) were added. The vial was sealed with a Teflon-lined screw cap and transferred to a cryocooler precooled at 10 °C where the reaction mixture was stirred for 5 min without irradiation and then irradiated by 456 nm LEDs for 12 h. Subsequently, silica gel (5–10 mL) was added, and the reaction mixture was concentrated to dryness under reduced pressure. The resulting residue was purified by chromatography on silica gel (hexanes/EtOAc = 10:1) to afford **30** as a colorless oil in 50% yield (19.8 mg).

**R*f*** = 0.20 (hexanes/EtOAc = 10:1).

**NMR Spectroscopy:**

**$^{1}$H NMR** (500 MHz, $CDCl_3$, 23 ºC, δ): 7.49 (d, *J* = 6.5 Hz, 2H), 7.41 (t, *J* = 8.0 Hz, 1H), 7.29 (d, *J* = 8.2 Hz, 2H), 7.20 – 7.10 (m, 2H), 3.81 (s, 3H), 3.78 (q, *J* = 7.3 Hz, 1H), 3.72 (s, 3H), 3.16 (d, *J* = 13.7 Hz, 1H), 3.04 (d, *J* = 13.7 Hz, 1H), 1.56 (d, *J* = 7.1 Hz, 3H), 1.53 (s, 3H).

**$^{13}$C NMR** (125 MHz, $CDCl_3$, 23 ºC, δ): 174.4, 172.6, 159.7 (d, *J* = 248.0 Hz), 141.8 (d, *J* = 7.7 Hz), 134.5, 134.4, 130.7 (d, *J* = 3.8 Hz), 130.4, 128.8 (d, *J* = 3.0 Hz), 127.4 (d, *J* = 13.6 Hz), 123.5 (d, *J* = 3.3 Hz), 115.3 (d, *J* = 23.5 Hz), 67.0, 52.8, 52.2, 44.9, 43.5, 22.3, 18.4 .

**$^{19}$F NMR** (470 MHz, $CDCl_3$, 23 ºC, δ): –117.44 (dd, *J* = 11.3, 8.3 Hz).

**HRMS-ESIpos (m/z)** calc'd for $C_{21}H_{22}N_3O_4FNa$ $[M+Na]^+$, 422.1487; found, 422.1486; deviation: +0.1 ppm.

**Flubiprofen-derived phenylethylazide 31**

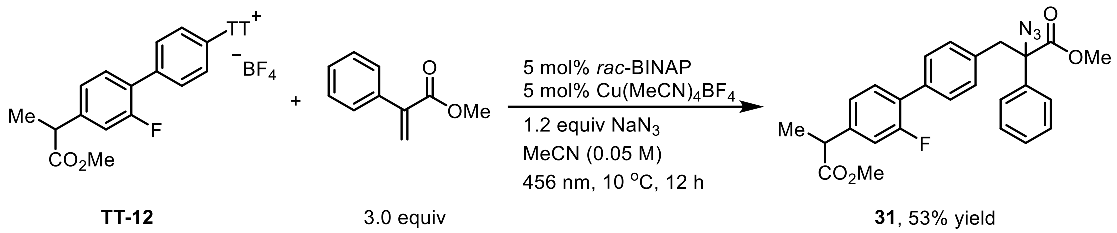


To a 4-mL borosilicate vial containing a Teflon-coated magnetic stirring bar were added **TT-12** (55.8 mg, 0.100 mmol, 1.00 equiv), *rac*-BINAP (3.1 mg, 5.0 μmol, 5.0 mol%), and sodium azide (7.8 mg, 0.12 mmol, 1.2 equiv). The vial was transferred into a nitrogen-filled glovebox. $Cu(MeCN)_4BF_4$ (1.6 mg, 5.0 μmol, 5.0 mol%), dry MeCN (2 mL, c = 0.05 M), and methyl 2-phenylacrylate (49 mg, 0.30 mmol, 3.0 equiv) were added. The vial was sealed with a Teflon-lined screw cap and transferred to a cryocooler precooled at 10 °C where the reaction mixture was stirred for 5 min without irradiation and then irradiated by 456 nm LEDs for 12 h. Subsequently, silica gel (5–10 mL) was added, and the reaction mixture was concentrated to dryness under reduced pressure. The resulting residue was purified by chromatography on silica gel (hexanes/EtOAc = 10:1) to afford **31** as a colorless oil in 53% yield (24.3 mg).

**R*f*** = 0.20 (hexanes/EtOAc = 10:1).

**NMR Spectroscopy:**

**$^1$H NMR** (500 MHz, $CDCl_3$, 23 °C, δ): 7.48 – 7.34 (m, 8H), 7.20 (d, *J* = 8.2 Hz, 2H), 7.18 – 7.08 (m, 2H), 3.85 (s, 3H), 3.78 (q, *J* = 7.3 Hz, 1H), 3.72 (s, 3H), 3.57 (d, *J* = 13.8 Hz, 1H), 3.35 (d, *J* = 13.8 Hz, 1H), 1.56 (d, *J* = 7.2 Hz, 3H).

**$^{13}$C NMR** (125 MHz, $CDCl_3$, 23 °C, δ): 174.4, 171.1, 159.7 (d, *J* = 248.5 Hz), 141.7 (d, *J* = 7.7 Hz), 137.7, 134.4, 134.2 (d, *J* = 1.4 Hz), 130.7, 130.7 (d, *J* = 4.4 Hz), 128.8, 128.6, 128.5 (d, *J* = 3.1 Hz), 126.0, 123.5 (d, *J* = 3.4 Hz), 115.2 (d, *J* = 23.7 Hz), 73.8, 53.0, 52.2, 44.9 (d, *J* = 1.5 Hz), 44.1, 18.4 .

**$^{19}$F NMR** (282 MHz, $CDCl_3$, 23 °C, δ): –117.16 – –117.57 (m).

**HRMS-ESIpos (m/z)** calc'd for $C_{26}H_{24}N_3O_4FNa$ $[M+Na]^+$, 484.1643; found, 484.1645; deviation: –0.3 ppm.

**Flubiprofen-derived phenylethylazide 32**

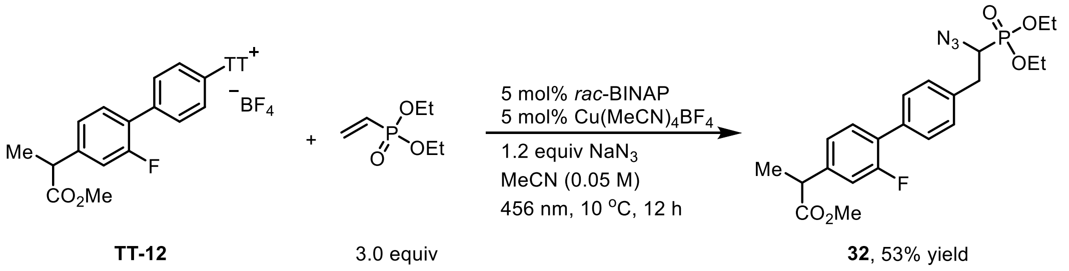


To a 4-mL borosilicate vial containing a Teflon-coated magnetic stirring bar were added **TT-12** (55.8 mg,

0.100 mmol, 1.00 equiv), *rac*-BINAP (3.1 mg, 5.0 μmol, 5.0 mol%), and sodium azide (7.8 mg, 0.12 mmol, 1.2 equiv). The vial was transferred into a nitrogen-filled glovebox. $Cu(MeCN)_4BF_4$ (1.6 mg, 5.0 μmol, 5.0 mol%), dry MeCN (2 mL, c = 0.05 M), and diethyl vinylphosphonate (33 mg, 0.20 mmol, 2.0 equiv) were added. The vial was sealed with a Teflon-lined screw cap and transferred to a cryocooler precooled at 10 °C where the reaction mixture was stirred for 5 min without irradiation and then irradiated by 456 nm LEDs for 12 h. Subsequently, silica gel (5–10 mL) was added, and the reaction mixture was concentrated to dryness under reduced pressure. The resulting residue was purified by chromatography on silica gel (hexanes/EtOAc = 1:2) to afford **32** as a colorless oil in 53% yield (24.7 mg).

**R*f*** = 0.23 (hexanes/EtOAc = 1:2).

**NMR Spectroscopy:**

**$^1$H NMR** (500 MHz, $CDCl_3$, 23 ºC, δ): 7.53 (d, *J* = 8.2 Hz, 2H), 7.41 (t, *J* = 8.0 Hz, 1H), 7.36 (d, *J* = 7.8 Hz, 2H), 7.19 – 7.11 (m, 2H), 4.34 – 4.18 (m, 4H), 3.82 – 3.72 (m, 2H), 3.72 (d, *J* = 1.2 Hz, 3H), 3.27 (ddd, *J* = 14.6, 6.7, 3.2 Hz, 1H), 2.94 (ddd, *J* = 14.7, 11.4, 8.0 Hz, 1H), 1.56 (d, *J* = 7.2 Hz, 3H), 1.41 (q, *J* = 6.9 Hz, 6H).

**$^{13}$C NMR** (125 MHz, $CDCl_3$, 23 ºC, δ): 174.4, 159.7 (d, *J* = 248.2 Hz), 141.9 (d, *J* = 7.3 Hz), 136.5 (d, *J* = 15.2 Hz), 134.4, 130.7 (d, *J* = 3.8 Hz), 129.2 (d, *J* = 2.9 Hz), 129.2, 127.4 (d, *J* = 13.5 Hz), 123.6 (d, *J* = 3.4 Hz), 115.3 (d, *J* = 23.8 Hz), 63.2 (d, *J* = 7.1 Hz), 63.1 (d, *J* = 6.9 Hz), 58.8 (d, *J* = 154.5 Hz), 52.2, 44.9, 34.6, 18.4, 16.5 (t, *J* = 5.3 Hz).

**$^{19}$F NMR** (470 MHz, $CDCl_3$, 23 ºC, δ): –117.46 – –117.56 (m).

**$^{31}$P NMR** (203 MHz, $CDCl_3$, 23 ºC, δ): 21.33.

**HRMS-ESIpos (m/z)** calc'd for $C_{22}H_{27}N_3O_5FPNa$ $[M+Na]^+$, 486.1565; found, 486.1569; deviation: –0.8 ppm.

**Flubiprofen-derived phenylethylazide 33**

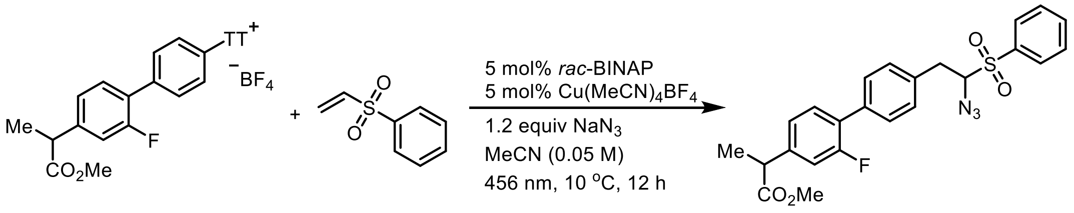


**TT-12** 2.0 equiv **33**, 56% yield

To a 4-mL borosilicate vial containing a Teflon-coated magnetic stirring bar were added **TT-12** (55.8 mg, 0.100 mmol, 1.00 equiv), *rac*-BINAP (3.1 mg, 5.0 μmol, 5.0 mol%), and sodium azide (7.8 mg, 0.12 mmol, 1.2 equiv). The vial was transferred into a nitrogen-filled glovebox. $Cu(MeCN)_4BF_4$ (1.6 mg, 5.0 μmol, 5.0 mol%), dry MeCN (2 mL, c = 0.05 M), and phenyl vinyl sulfone (34 mg, 0.20 mmol, 2.0 equiv) were added. The vial was sealed with a Teflon-lined screw cap and transferred to a cryocooler precooled at 10 °C where the reaction mixture was stirred for 5 min without irradiation and then irradiated by 456 nm LEDs for 12 h.

Subsequently, silica gel (5–10 mL) was added, and the reaction mixture was concentrated to dryness under reduced pressure. The resulting residue was purified by chromatography on silica gel (hexanes/acetone = 5:1) to afford **33** as a colorless oil in 56% yield (26.3 mg).

**R*f*** = 0.22 (hexanes/acetone = 5:1).

**NMR Spectroscopy:**

**$^{1}$H NMR** (500 MHz, $CDCl_3$, 23 ºC, δ): 7.49 (d, *J* = 6.5 Hz, 2H), 7.45 – 7.39 (m, 3H), 7.39 – 7.32 (m, 3H), 7.25 (d, *J* = 8.0 Hz, 2H), 7.19 – 7.12 (m, 2H), 4.74 (dd, *J* = 8.5, 5.9 Hz, 1H), 3.79 (q, *J* = 7.2 Hz, 1H), 3.73 (s, 3H), 3.15 (dd, *J* = 13.9, 8.5 Hz, 1H), 3.09 (dd, *J* = 13.9, 5.9 Hz, 1H), 1.56 (d, *J* = 7.2 Hz, 3H).

**$^{13}$C NMR** (125 MHz, $CDCl_3$, 23 ºC, δ): 174.4, 159.7 (d, *J* = 248.5 Hz), 141.8 (d, *J* = 7.7 Hz), 139.3, 137.0, 134.0, 130.7 (d, *J* = 3.8 Hz), 129.5, 128.9 (d, *J* = 3.0 Hz), 128.8, 128.4, 127.5 (d, *J* = 13.3 Hz), 127.0, 123.5 (d, *J* = 3.4 Hz), 115.3 (d, *J* = 23.7 Hz), 67.6, 52.2, 44.9, 42.7, 18.4.

**$^{19}$F NMR** (470 MHz, $CDCl_3$, 23 ºC, δ): –117.34 – –117.52 (m).

**HRMS-ESIpos (m/z)** calc'd for $C_{24}H_{22}N_3O_4FSNa$ $[M+Na]^+$, 490.1207; found, 490.1211; deviation: –0.7 ppm.

**Flubiprofen-derived phenylethylazide 34**

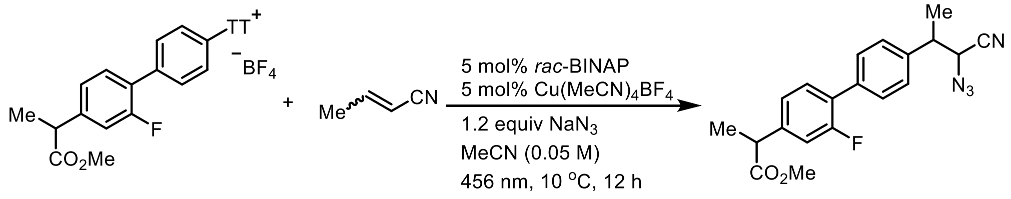


**TT-12** 3.0 equiv **34**, 36% yield, 1.2:1 dr

To a 4-mL borosilicate vial containing a Teflon-coated magnetic stirring bar were added **TT-12** (55.8 mg, 0.100 mmol, 1.00 equiv), *rac*-BINAP (3.1 mg, 5.0 μmol, 5.0 mol%), and sodium azide (7.8 mg, 0.12 mmol, 1.2 equiv). The vial was transferred into a nitrogen-filled glovebox. $Cu(MeCN)_4BF_4$ (1.6 mg, 5.0 μmol, 5.0 mol%), dry MeCN (2 mL, c = 0.05 M), and crotononitrile (20 mg, 25 μL, 0.30 mmol, 3.0 equiv) were added. The vial was sealed with a Teflon-lined screw cap and transferred to a cryocooler precooled at 10 °C where the reaction mixture was stirred for 5 min without irradiation and then irradiated by 456 nm LEDs for 12 h. To determine the diastereoselectivity, a 100 µL reaction mixture was extracted and analyzed using $^{1}$H NMR spectroscopy and GC-MS (1.2:1 dr). Subsequently, silica gel (5–10 mL) was added, and the reaction mixture was concentrated to dryness under reduced pressure. The resulting residue was purified by chromatography on silica gel (hexanes/EtOAc = 7.5:1) to afford **34** as a colorless oil in 36% yield (13.3 mg).

**R*f*** = 0.21 (hexanes/EtOAc = 7.5:1).

**NMR Spectroscopy:**

**$^{1}$H NMR** (300 MHz, $CDCl_3$, 23 ºC, δ): 7.57 (d, *J* = 7.9 Hz, 2H), 7.44 – 7.33 (m, 3H), 7.22 – 7.10 (m, 2H), 4.40 (d, *J* = 6.8 Hz, 1H), 3.79 (q, *J* = 7.2 Hz, 1H), 3.73 (s, 3H), 3.35 – 3.19 (m, 1H), 1.62 – 1.51 (m, 6H).

**$^{13}$C NMR** (125 MHz, $CDCl_3$, 23 ºC, δ): 174.3, 159.7 (d, *J* = 248.6 Hz), 144.3, 142.1, 138.5, 135.4 (d, *J* = 7.7 Hz), 130.6 (d, *J* = 2.1 Hz), 129.4 (d, *J* = 3.1 Hz), 127.7, 123.6 (d, *J* = 3.3 Hz), 115.3 (d, *J* = 23.6 Hz), 115.2, 57.6, 52.2, 44.9 (d, *J* = 1.5 Hz), 42.6, 18.4, 16.7 .

**$^{19}$F NMR** (282 MHz, $CDCl_3$, 23 ºC, δ): –117.37 – –117.59 (m).

**HRMS-ESIpos (m/z)** calc'd for $C_{20}H_{19}N_4O_2FNa$ $[M+Na]^+$, 389.1384; found, 389.1384; deviation: +0.2 ppm.

**Flubiprofen-derived phenylethylazide 35**

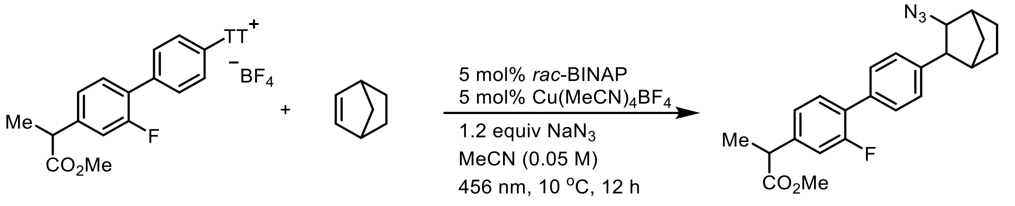


**TT-12** 3.0 equiv **35**, 46% yield, > 20:1 dr

To a 4-mL borosilicate vial containing a Teflon-coated magnetic stirring bar were added **TT-12** (55.8 mg, 0.100 mmol, 1.00 equiv), *rac*-BINAP (3.1 mg, 5.0 μmol, 5.0 mol%), and sodium azide (7.8 mg, 0.12 mmol, 1.2 equiv). The vial was transferred into a nitrogen-filled glovebox. $Cu(MeCN)_4BF_4$ (1.6 mg, 5.0 μmol, 5.0 mol%), dry MeCN (2 mL, c = 0.05 M), and norbornene (28 mg, 0.30 mmol, 3.0 equiv) were added. The vial was sealed with a Teflon-lined screw cap and transferred to a cryocooler precooled at 10 °C where the reaction mixture was stirred for 5 min without irradiation and then irradiated by 456 nm LEDs for 12 h. To determine the diastereoselectivity, a 100 μL reaction mixture was extracted and analyzed using $^1$H NMR spectroscopy and GC-MS (>20:1 dr). Subsequently, silica gel (5–10 mL) was added, and the reaction mixture was concentrated to dryness under reduced pressure. The resulting residue was purified by chromatography on silica gel (hexanes/EtOAc = 40:1) to afford **35** as a colorless oil in 46% yield (18.0 mg).

**$R_f$** = 0.23 (hexanes/EtOAc = 40:1).

**NMR Spectroscopy:**

**$^1$H NMR** (300 MHz, $CDCl_3$, 23 ºC, δ): 7.52 (dd, *J* = 8.4, 1.7 Hz, 2H), 7.45 – 7.32 (m, 3H), 7.21 – 7.10 (m, 2H), 3.99 – 3.89 (m, 1H), 3.79 (q, *J* = 7.2 Hz, 1H), 3.73 (s, 3H), 2.58 – 2.49 (m, 3H), 1.97 – 1.66 (m, 3H), 1.56 (d, *J* = 7.2 Hz, 3H), 1.54 – 1.43 (m, 3H).

**$^{13}$C NMR** (75 MHz, $CDCl_3$, 23 ºC, δ): 174.4, 159.7 (d, *J* = 248.3 Hz), 143.9, 141.7 (d, *J* = 7.6 Hz), 133.4, 130.6 (d, *J* = 3.9 Hz), 129.1 (d, *J* = 3.0 Hz), 126.9, 123.5 (d, *J* = 3.3 Hz), 115.2 (d, *J* = 23.7 Hz), 71.6, 53.5, 52.2, 44.9 (d, *J* = 1.5 Hz), 42.7, 41.4, 36.1, 30.7, 21.3, 18.4 .

**$^{19}$F NMR** (282 MHz, $CDCl_3$, 23 ºC, δ): –117.55 (dd, *J* = 11.3, 8.6 Hz).

**HRMS-ESIpos (m/z)** calc'd for $C_{23}H_{24}N_3O_2FNa$ $[M+Na]^+$, 416.1745; found, 416.1739; deviation: 1.4 ppm.

**Flubiprofen-derived phenylethylazide 36**

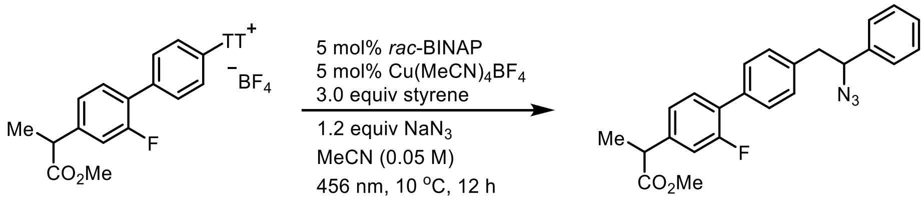


**TT-12** **36**, 68% yield

To a 4-mL borosilicate vial containing a Teflon-coated magnetic stirring bar were added **TT-12** (55.8 mg, 0.100 mmol, 1.00 equiv), *rac*-BINAP (3.1 mg, 5.0 μmol, 5.0 mol%), and sodium azide (7.8 mg, 0.12 mmol, 1.2 equiv). The vial was transferred into a nitrogen-filled glovebox. $Cu(MeCN)_4BF_4$ (1.6 mg, 5.0 μmol, 5.0 mol%), dry MeCN (2 mL, c = 0.05 M), and styrene (31 mg, 34 μL, 0.30 mmol, 3.0 equiv) were added. The vial was sealed with a Teflon-lined screw cap and transferred to a cryocooler precooled at 10 °C where the reaction mixture was stirred for 5 min without irradiation and then irradiated by 456 nm LEDs for 12 h. Subsequently, silica gel (5–10 mL) was added, and the reaction mixture was concentrated to dryness under reduced pressure. The resulting residue was purified by chromatography on silica gel (hexanes/EtOAc = 20:1) to afford **36** as a colorless oil in 68% yield (27.3 mg).

$\mathbf{R}_f$ = 0.21 (hexanes/EtOAc = 20:1).

**NMR Spectroscopy:**

**$^1$H NMR** (500 MHz, $CDCl_3$, 23 ºC, δ): 8.06 – 8.00 (m, 2H), 7.80 – 7.75 (m, 1H), 7.67 (t, *J* = 7.9 Hz, 2H), 7.51 (dd, *J* = 8.2, 1.7 Hz, 2H), 7.39 (t, *J* = 8.0 Hz, 1H), 7.33 – 7.27 (m, 2H), 7.21 – 7.11 (m, 2H), 4.48 (dd, *J* = 11.5, 2.6 Hz, 1H), 3.78 (q, *J* = 7.2 Hz, 1H), 3.72 (s, 3H), 3.54 (dd, *J* = 14.2, 2.6 Hz, 1H), 2.76 (dd, *J* = 14.2, 11.5 Hz, 1H), 1.56 (d, *J* = 7.2 Hz, 3H).

**$^{13}$C NMR** (125 MHz, $CDCl_3$, 23 ºC, δ): 174.4, 159.7 (d, *J* = 248.3 Hz), 142.0 (d, *J* = 7.7 Hz), 135.5, 134.8, 134.7, 134.2, 130.7 (d, *J* = 3.9 Hz), 129.5 (d, *J* = 6.9 Hz), 129.4 (d, *J* = 3.0 Hz), 129.3, 127.1 (d, *J* = 13.7 Hz), 123.6 (d, *J* = 3.4 Hz), 115.3 (d, *J* = 23.7 Hz), 80.7, 52.2, 44.9, 33.4, 18.4.

**$^{19}$F NMR** (470 MHz, $CDCl_3$, 23 ºC, δ): –117.53 (dd, *J* = 11.5, 8.3 Hz).

**HRMS-ESIpos (m/z)** calc'd for $C_{24}H_{22}N_3O_2FNa$ $[M+Na]^+$, 426.1588; found, 426.1590; deviation: –0.4 ppm.

**Flubiprofen-derived phenylethylazide 37**

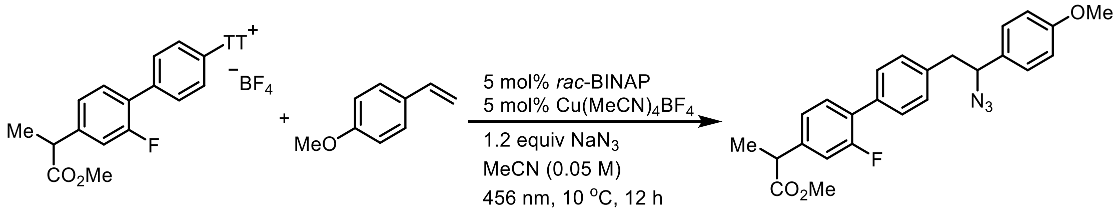


**TT-12** 3.0 equiv **37**, 48% yield

To a 4-mL borosilicate vial containing a Teflon-coated magnetic stirring bar were added **TT-12** (55.8 mg, 0.100 mmol, 1.00 equiv), *rac*-BINAP (3.1 mg, 5.0 μmol, 5.0 mol%), and sodium azide (7.8 mg, 0.12 mmol, 1.2 equiv). The vial was transferred into a nitrogen-filled glovebox. $Cu(MeCN)_4BF_4$ (1.6 mg, 5.0 μmol, 5.0 mol%), dry MeCN (2 mL, c = 0.05 M), and 4-methoxystyrene (40 mg, 40 μL, 0.30 mmol, 3.0 equiv) were added. The vial was sealed with a Teflon-lined screw cap and transferred to a cryocooler precooled at 10 °C where the reaction mixture was stirred for 5 min without irradiation and then irradiated by 456 nm LEDs for 12 h. Subsequently, silica gel (5–10 mL) was added, and the reaction mixture was concentrated to dryness under reduced pressure. The resulting residue was purified by chromatography on silica gel (hexanes/EtOAc = 10:1) to afford **37** as a colorless oil in 48% yield (21.0 mg).

**$R_f$** = 0.20 (hexanes/EtOAc = 10:1).

**NMR Spectroscopy:**

**$^1$H NMR** (500 MHz, $CDCl_3$, 23 ºC, δ): 7.48 (d, *J* = 6.5 Hz, 2H), 7.40 (t, *J* = 8.0 Hz, 1H), 7.27 (d, *J* = 8.5 Hz, 2H), 7.23 (d, *J* = 7.9 Hz, 2H), 7.15 (dd, *J* = 11.5, 9.7 Hz, 2H), 6.93 (d, *J* = 8.6 Hz, 2H), 4.69 (dd, *J* = 8.3, 6.1 Hz, 1H), 3.84 (s, 3H), 3.78 (q, *J* = 7.2 Hz, 1H), 3.72 (s, 3H), 3.13 (dd, *J* = 13.9, 8.4 Hz, 1H), 3.06 (dd, *J* = 13.8, 6.1 Hz, 1H), 1.56 (d, *J* = 7.2 Hz, 3H).

**$^{13}$C NMR** (125 MHz, $CDCl_3$, 23 ºC, δ): 174.4, 159.7 (d, *J* = 248.2 Hz), 159.6, 141.7 (d, *J* = 7.7 Hz), 137.1, 133.9, 131.2, 130.7 (d, *J* = 3.9 Hz), 129.5, 128.9 (d, *J* = 3.0 Hz), 128.3, 127.5 (d, *J* = 13.5 Hz), 123.5 (d, *J* = 3.3 Hz), 115.2 (d, *J* = 23.8 Hz), 114.1, 67.1, 55.3, 52.2, 44.9, 42.6, 18.4 .

**$^{19}$F NMR** (470 MHz, $CDCl_3$, 23 ºC, δ): –117.45 (dd, *J* = 11.2, 8.4 Hz).

**HRMS-ESIpos (m/z)** calc'd for $C_{25}H_{24}N_3O_3FNa$ $[M+Na]^+$, 456.1694; found, 456.1693; deviation: +0.2 ppm.

**Flubiprofen-derived phenylethylazide 38**

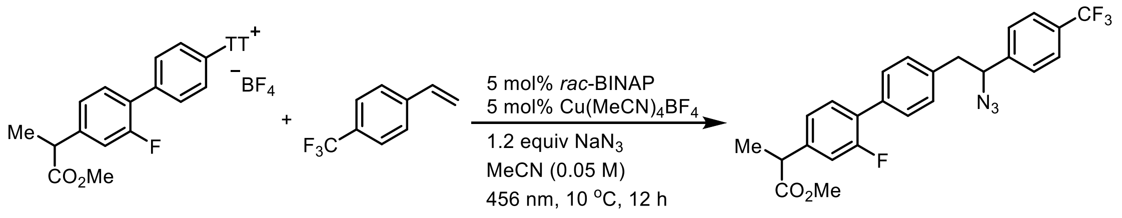


**TT-12** 3.0 equiv **38**, 48% yield

To a 4-mL borosilicate vial containing a Teflon-coated magnetic stirring bar were added **TT-12** (55.8 mg, 0.100 mmol, 1.00 equiv), *rac*-BINAP (3.1 mg, 5.0 μmol, 5.0 mol%), and sodium azide (7.8 mg, 0.12 mmol, 1.2 equiv). The vial was transferred into a nitrogen-filled glovebox. $Cu(MeCN)_4BF_4$ (1.6 mg, 5.0 μmol, 5.0 mol%), dry MeCN (2 mL, c = 0.05 M), and 4-(trifluoromethyl)styrene (52 mg, 44 μL, 0.30 mmol, 3.0 equiv) were added. The vial was sealed with a Teflon-lined screw cap and transferred to a cryocooler precooled at 10 °C where the reaction mixture was stirred for 5 min without irradiation and then irradiated by 456 nm LEDs for 12 h. Subsequently, silica gel (5–10 mL) was added, and the reaction mixture was concentrated to dryness

under reduced pressure. The resulting residue was purified by chromatography on silica gel (hexanes/EtOAc = 15:1) to afford **38** as a colorless oil in 48% yield (22.5 mg).

**R*f*** = 0.20 (hexanes/EtOAc = 15:1).

**NMR Spectroscopy:**

**$^{1}$H NMR** (500 MHz, $CDCl_3$, 23 ºC, δ): 7.66 (d, *J* = 8.1 Hz, 2H), 7.51 – 7.48 (m, 2H), 7.44 (d, *J* = 8.1 Hz, 2H), 7.41 (t, *J* = 8.0 Hz, 1H), 7.22 (d, *J* = 8.3 Hz, 2H), 7.19 – 7.11 (m, 2H), 4.81 (dd, *J* = 8.2, 6.0 Hz, 1H), 3.78 (q, *J* = 7.2 Hz, 1H), 3.73 (s, 3H), 3.15 (dd, *J* = 13.9, 8.2 Hz, 1H), 3.07 (dd, *J* = 13.9, 6.0 Hz, 1H), 1.56 (d, *J* = 7.2 Hz, 3H).

**$^{13}$C NMR** (125 MHz, $CDCl_3$, 23 ºC, δ): 174.6, 159.8 (d, *J* = 248.5 Hz), 143.4 (d, *J* = 1.5 Hz), 142.0 (d, *J* = 7.7 Hz), 136.3, 134.4 (d, *J* = 1.3 Hz), 130.8 (d, *J* = 3.8 Hz), 130.7 (q, *J* = 32.7 Hz), 129.6, 129.2 (d, *J* = 3.1 Hz), 127.5 (d, *J* = 13.4 Hz), 127.5, 125.9 (q, *J* = 3.8 Hz), 124.1 (q, *J* = 272.2 Hz). 123.7 (d, *J* = 3.2 Hz), 115.4 (d, *J* = 23.6 Hz), 67.1, 52.4, 45.1 (d, *J* = 1.4 Hz), 42.9, 18.6.

**$^{19}$F NMR** (470 MHz, $CDCl_3$, 23 ºC, δ): –62.60 (s, 3H), –117.47 (dd, *J* = 11.1, 8.2 Hz, 1H).

**HRMS-ESIpos (m/z)** calc'd for $C_{25}H_{21}N_3O_2F_4Na$ $[M+Na]^+$, 494.1462; found, 494.1464; deviation: –0.4 ppm.

**Flubiprofen-derived phenylethylazide 39**

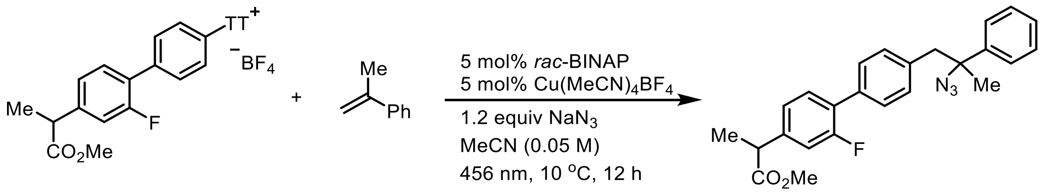


**TT-12** 3.0 equiv **39**, 60% yield

To a 4-mL borosilicate vial containing a Teflon-coated magnetic stirring bar were added **TT-12** (55.8 mg, 0.100 mmol, 1.00 equiv), *rac*-BINAP (3.1 mg, 5.0 μmol, 5.0 mol%), and sodium azide (7.8 mg, 0.12 mmol, 1.2 equiv). The vial was transferred into a nitrogen-filled glovebox. $Cu(MeCN)_4BF_4$ (1.6 mg, 5.0 μmol, 5.0 mol%), dry MeCN (2 mL, c = 0.05 M), and 2-phenyl-1-propene (36 mg, 39 μL, 0.30 mmol, 3.0 equiv) were added. The vial was sealed with a Teflon-lined screw cap and transferred to a cryocooler precooled at 10 °C where the reaction mixture was stirred for 5 min without irradiation and then irradiated by 456 nm LEDs for 12 h. Subsequently, silica gel (5–10 mL) was added, and the reaction mixture was concentrated to dryness under reduced pressure. The resulting residue was purified by chromatography on silica gel (hexanes/EtOAc = 30:1) to afford **39** as a colorless oil in 60% yield (25.1 mg).

**R*f*** = 0.16 (hexanes/EtOAc = 30:1).

**NMR Spectroscopy:**

**$^{1}$H NMR** (500 MHz, $CDCl_3$, 23 ºC, δ): 7.43 – 7.36 (m, 7H), 7.36 – 7.29 (m, 1H), 7.18 – 7.10 (m, 2H), 7.03 (d, *J* = 8.2 Hz, 2H), 3.78 (q, *J* = 7.2 Hz, 1H), 3.72 (s, 3H), 3.10 (s, 2H), 1.75 (s, 3H), 1.56 (d, *J* = 7.2 Hz,

3H).

**$^{13}$C NMR** (125 MHz, $CDCl_3$, 23 ºC, δ): 174.4, 159.7 (d, *J* = 248.5 Hz), 143.1, 141.7 (d, *J* = 7.7 Hz), 135.7, 133.9, 130.7, 130.7, 128.4, 128.3 (d, *J* = 3.0 Hz), 127.6, 127.4, 126.0, 123.5 (d, *J* = 3.1 Hz), 115.2 (d, *J* = 23.8 Hz), 67.2, 52.2, 48.8, 44.9, 24.1, 18.4.

**$^{19}$F NMR** (470 MHz, $CDCl_3$, 23 ºC, δ): –117.42 (dd, *J* = 11.4, 8.4 Hz).

**HRMS-ESIpos (m/z)** calc'd for $C_{25}H_{24}N_3O_2FNa$ $[M+Na]^+$, 440.1745; found, 440.1749; deviation: –0.9 ppm.

***rac*-Flubiprofen-derived phenylethylazide 40**

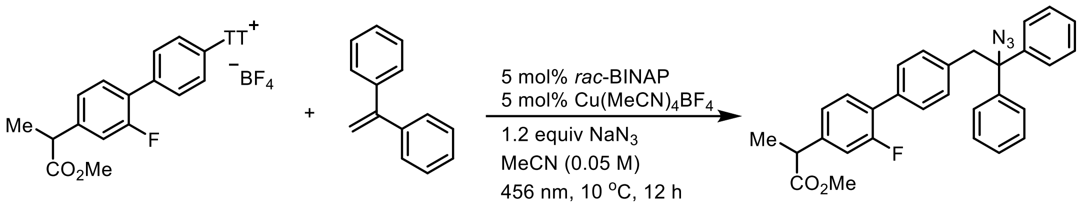


**TT-12** 3.0 equiv *rac*-**40**, 63% yield

To a 4-mL borosilicate vial containing a Teflon-coated magnetic stirring bar were added **TT-12** (55.8 mg, 0.100 mmol, 1.00 equiv), *rac*-BINAP (3.1 mg, 5.0 μmol, 5.0 mol%), and sodium azide (7.8 mg, 0.12 mmol, 1.2 equiv). The vial was transferred into a nitrogen-filled glovebox. $Cu(MeCN)_4BF_4$ (1.6 mg, 5.0 μmol, 5.0 mol%), dry MeCN (2 mL, c = 0.05 M), and 1,1-diphenylethylene (54 mg, 53 μL, 0.30 mmol, 3.0 equiv) were added. The vial was sealed with a Teflon-lined screw cap and transferred to a cryocooler precooled at 10 °C where the reaction mixture was stirred for 5 min without irradiation and then irradiated by 456 nm LEDs for 12 h. Subsequently, silica gel (5–10 mL) was added, and the reaction mixture was concentrated to dryness under reduced pressure. The resulting residue was purified by chromatography on silica gel (hexanes/EtOAc = 20:1) to afford *rac*-**40** as a colorless oil in 63% yield (30.0 mg).

**R*f*** = 0.31 (hexanes/EtOAc = 10:1).

**NMR Spectroscopy:**

**$^{1}$H NMR** (500 MHz, $CDCl_3$, 23 ºC, δ): 7.41 – 7.27 (m, 13H), 7.19 – 7.09 (m, 2H), 6.83 (d, *J* = 8.2 Hz, 2H), 3.78 (q, *J* = 7.1 Hz, 1H), 3.73 (s, 2H), 3.72 (s, 3H), 1.56 (d, *J* = 7.2 Hz, 3H).

**$^{13}$C NMR** (125 MHz, $CDCl_3$, 23 ºC, δ): 174.4, 159.7 (d, *J* = 248.5 Hz), 142.5, 141.6 (d, *J* = 7.7 Hz), 135.2, 133.7, 130.7 (d, *J* = 3.9 Hz), 130.6, 128.2, 128.1 (d, *J* = 3.3 Hz), 127.6, 127.5, 123.5 (d, *J* = 3.4 Hz), 115.2 (d, *J* = 23.7 Hz), 73.2, 52.2, 44.9, 44.8, 18.4.

**$^{19}$F NMR** (470 MHz, $CDCl_3$, 23 ºC, δ): –117.34 (dd, *J* = 11.3, 8.4 Hz).

**HRMS-ESIpos (m/z)** calc'd for $C_{30}H_{26}N_3O_2NaF$ $[M+Na]^+$, 502.1901; found, 502.1901; deviation: 0.0 ppm.

**Flubiprofen-derived phenylethylazide 41**

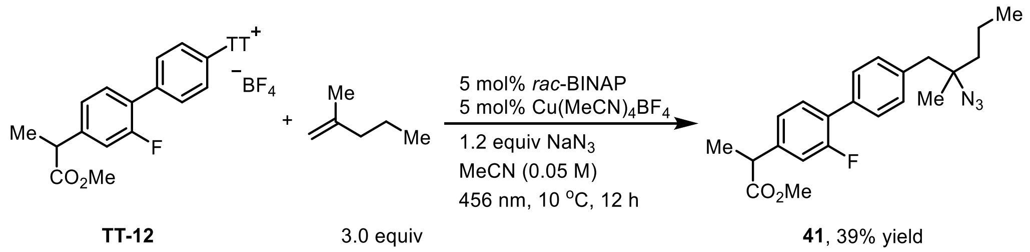


To a 4-mL borosilicate vial containing a Teflon-coated magnetic stirring bar were added **TT-12** (55.8 mg, 0.100 mmol, 1.00 equiv), *rac*-BINAP (3.1 mg, 5.0 μmol, 5.0 mol%), and sodium azide (7.8 mg, 0.12 mmol, 1.2 equiv). The vial was transferred into a nitrogen-filled glovebox. $Cu(MeCN)_4BF_4$ (1.6 mg, 5.0 μmol, 5.0 mol%), dry MeCN (2 mL, c = 0.05 M), and 2-methyl-1-pentene (84.2 mg, 123 μL, 1.00 mmol, 10.0 equiv) were added. The vial was sealed with a Teflon-lined screw cap and transferred to a cryocooler precooled at 10 °C where the reaction mixture was stirred for 5 min without irradiation and then irradiated by 456 nm LEDs for 12 h. Subsequently, silica gel (5–10 mL) was added, and the reaction mixture was concentrated to dryness under reduced pressure. The resulting residue was purified by chromatography on silica gel (hexanes/EtOAc = 40:1) to afford **41** as a colorless oil in 39% yield (15.1 mg).

$\mathbf{R}_f$ = 0.22 (hexanes/EtOAc = 40:1).

**NMR Spectroscopy:**

**$^{1}$H NMR** (500 MHz, $CDCl_3$, 23 ºC, δ): 7.55 – 7.48 (m, 2H), 7.42 (t, *J* = 8.0 Hz, 1H), 7.30 (d, *J* = 8.2 Hz, 2H), 7.19 – 7.10 (m, 2H), 3.79 (q, *J* = 7.2 Hz, 1H), 3.73 (s, 3H), 2.98 – 2.73 (m, 2H), 1.61 – 1.47 (m, 7H), 1.27 (s, 3H), 0.98 (t, *J* = 6.9 Hz, 3H).

**$^{13}$C NMR** (125 MHz, $CDCl_3$, 23 ºC, δ): 174.4, 159.7 (d, *J* = 248.4 Hz), 141.7 (d, *J* = 7.5 Hz), 136.2, 133.9, 130.7 (d, *J* = 4.0 Hz), 130.6, 128.6 (d, *J* = 3.0 Hz), 127.5 (d, *J* = 13.6 Hz), 123.5 (d, *J* = 3.4 Hz), 115.2 (d, *J* = 23.8 Hz), 64.5, 52.2, 45.6, 44.9, 41.8, 23.0, 18.4, 17.4, 14.4.

**$^{19}$F NMR** (470 MHz, $CDCl_3$, 23 ºC, δ): –117.46 (dd, *J* = 11.4, 8.4 Hz).

**HRMS-ESIpos (m/z)** calc'd for $C_{22}H_{26}N_3O_2FNa$ $[M+Na]^+$, 406.1901; found, 406.1903; deviation: –0.5 ppm.

***rac*-Pyridinylethylazide 42**

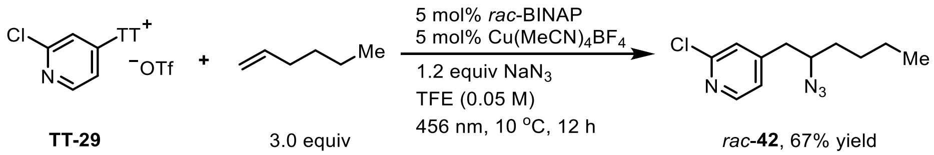


To a 4-mL borosilicate vial containing a Teflon-coated magnetic stirring bar were added **TT-29** (47.7 mg, 0.100 mmol, 1.00 equiv), *rac*-BINAP (3.1 mg, 5.0 μmol, 5.0 mol%), and sodium azide (7.8 mg, 0.12 mmol, 1.2 equiv). The vial was transferred into a nitrogen-filled glovebox. $Cu(MeCN)_4BF_4$ (1.6 mg, 5.0 μmol, 5.0 mol%),

dry 2,2,2-trifluoroethanol (TFE, 2 mL, c = 0.05 M), and 1-hexene (25 mg, 37 μL, 0.30 mmol, 3.0 equiv) were added. The vial was sealed with a Teflon-lined screw cap and transferred to a cryocooler precooled at 10 °C where the reaction mixture was stirred for 5 min without irradiation and then irradiated by 456 nm LEDs for 12 h. Subsequently, silica gel (5–10 mL) was added, and the reaction mixture was concentrated to dryness under reduced pressure. The resulting residue was purified by chromatography on silica gel (pure DCM as eluent) to afford *rac*-**42** as a colorless oil in 67% yield (15.8 mg).

**R*f*** = 0.21 (pure DCM).

**NMR Spectroscopy:**

**$^1$H NMR** (300 MHz, $CDCl_3$, 23 ºC, δ): 8.35 (s, 1H), 7.23 (s, 1H), 7.12 (s, 1H), 3.62 – 3.47 (m, 1H), 2.78 (qd, *J* = 13.9, 6.7 Hz, 2H), 1.65 – 1.51 (m, 2H), 1.53 – 1.29 (m, 4H), 0.95 (t, *J* = 7.1 Hz, 3H).

**$^{13}$C NMR** (75 MHz, $CDCl_3$, 23 ºC, δ): 151.8, 150.3, 149.6, 124.9, 123.3, 62.8, 39.8, 34.0, 28.1, 22.4, 13.9.

**HRMS-ESIpos (m/z)** calc'd for $C_{11}H_{16}N_4Cl$ $[M+H]^+$, 239.1058; found, 239.1058; deviation: +0.1 ppm.

***rac*-Pyridinylethylazide 43**

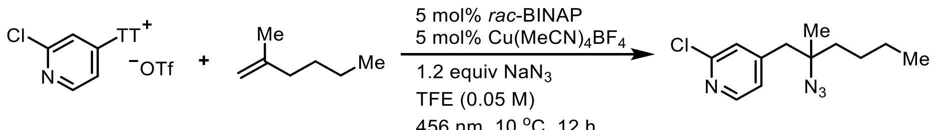


**TT-29** 3.0 equiv *rac*-**43**, 62% yield

To a 4-mL borosilicate vial containing a Teflon-coated magnetic stirring bar were added **TT-29** (47.7 mg, 0.100 mmol, 1.00 equiv), *rac*-BINAP (3.1 mg, 5.0 μmol, 5.0 mol%), and sodium azide (7.8 mg, 0.12 mmol, 1.2 equiv). The vial was transferred into a nitrogen-filled glovebox. $Cu(MeCN)_4BF_4$ (1.6 mg, 5.0 μmol, 5.0 mol%), dry 2,2,2-trifluoroethanol (TFE, 2 mL, c = 0.05 M), and 2-methyl-1-pentene (25 mg, 37 μL, 0.30 mmol, 3.0 equiv) were added. The vial was sealed with a Teflon-lined screw cap and transferred to a cryocooler precooled at 10 °C where the reaction mixture was stirred for 5 min without irradiation and then irradiated by 456 nm LEDs for 12 h. Subsequently, silica gel (5–10 mL) was added, and the reaction mixture was concentrated to dryness under reduced pressure. The resulting residue was purified by chromatography on silica gel (pure DCM as eluent) to afford *rac*-**43** as a colorless oil in 63% yield (15.0 mg).

**R*f*** = 0.21 (pure DCM).

**NMR Spectroscopy:**

**$^1$H NMR** (300 MHz, $CDCl_3$, 23 ºC, δ): 8.33 (d, *J* = 5.1 Hz, 1H), 7.22 (s, 1H), 7.11 (d, *J* = 5.1 Hz, 1H), 2.78 (d, *J* = 13.4 Hz, 1H), 2.68 (d, *J* = 13.4 Hz, 1H), 1.59 – 1.39 (m, 4H), 1.25 (s, 3H), 0.98 (t, *J* = 7.0 Hz, 3H).

**$^{13}$C NMR** (75 MHz, $CDCl_3$, 23 ºC, δ): 151.5, 149.2, 149.1, 126.0, 124.5, 63.5, 44.8, 42.1, 22.9, 17.3, 14.2.

**HRMS-ESIpos (m/z)** calc'd for $C_{11}H_{16}N_4Cl$ [M+H]$^+$, 239.1058; found, 239.1059; deviation: –0.5 ppm.

**Pyridinylethylazide 44**

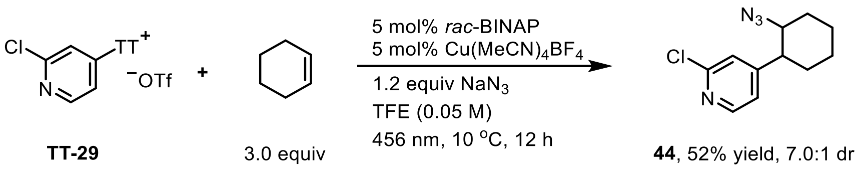

To a 4-mL borosilicate vial containing a Teflon-coated magnetic stirring bar were added **TT-29** (47.7 mg, 0.100 mmol, 1.00 equiv), *rac*-BINAP (3.1 mg, 5.0 μmol, 5.0 mol%), and sodium azide (7.8 mg, 0.12 mmol, 1.2 equiv). The vial was transferred into a nitrogen-filled glovebox. $Cu(MeCN)_4BF_4$ (1.6 mg, 5.0 μmol, 5.0 mol%), dry 2,2,2-trifluoroethanol (TFE, 2 mL, c = 0.05 M), and cyclohexene (25 mg, 30 μL, 0.30 mmol, 3.0 equiv) were added. The vial was sealed with a Teflon-lined screw cap and transferred to a cryocooler precooled at 10 °C where the reaction mixture was stirred for 5 min without irradiation and then irradiated by 456 nm LEDs for 12 h. To determine the diastereoselectivity, a 100 μL reaction mixture was extracted and analyzed using $^1$H NMR spectroscopy and GC-MS (7.0:1 dr). Subsequently, silica gel (5–10 mL) was added, and the reaction mixture was concentrated to dryness under reduced pressure. The resulting residue was purified by chromatography on silica gel (pure DCM as eluent) to afford **44** as a colorless oil in 52% yield (12.3 mg).

**R*f*** = 0.21 (pure DCM).

**NMR Spectroscopy:**

**$^1$H NMR** (300 MHz, $CDCl_3$, 23 ºC, δ): 8.34 (s, 1H), 7.22 (s, 1H), 7.10 (s, 1H), 3.41 (td, *J* = 10.8, 4.2 Hz, 1H), 2.59 – 2.43 (m, 1H), 2.33 – 2.22 (m, 1H), 2.02 – 1.79 (m, 3H), 1.61 – 1.29 (m, 4H).

**$^{13}$C NMR** (75 MHz, $CDCl_3$, 23 ºC, δ): 155.7, 151.8, 149.7, 123.4, 121.7, 63.7, 49.3, 33.2, 31.6, 25.3, 24.7.

**HRMS-ESIpos (m/z)** calc'd for $C_{11}H_{13}N_4ClNa$ [M+Na]$^+$, 259.0721; found, 259.0722; deviation: –0.4 ppm.

***rac*-Pyridinylethylazide 45**

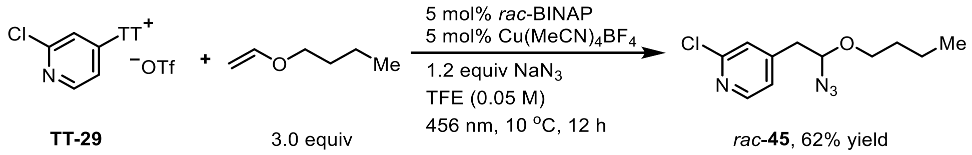

To a 4-mL borosilicate vial containing a Teflon-coated magnetic stirring bar were added **TT-29** (47.7 mg, 0.100 mmol, 1.00 equiv), *rac*-BINAP (3.1 mg, 5.0 μmol, 5.0 mol%), and sodium azide (7.8 mg, 0.12 mmol, 1.2 equiv). The vial was transferred into a nitrogen-filled glovebox. $Cu(MeCN)_4BF_4$ (1.6 mg, 5.0 μmol, 5.0 mol%), dry 2,2,2-trifluoroethanol (TFE, 2 mL, c = 0.05 M), and *n*-butyl vinyl ether (30 mg, 39 μL, 0.30 mmol, 3.0 equiv) were added. The vial was sealed with a Teflon-lined screw cap and transferred to a cryocooler

precooled at 10 °C where the reaction mixture was stirred for 5 min without irradiation and then irradiated by 456 nm LEDs for 12 h. Subsequently, silica gel (5–10 mL) was added, and the reaction mixture was concentrated to dryness under reduced pressure. The resulting residue was purified by chromatography on silica gel (pure DCM as eluent) to afford *rac*-**45** as a colorless oil in 62% yield (15.3 mg).

**R*f*** = 0.20 (pure DCM).

**NMR Spectroscopy:**

> **$^1$H NMR** (300 MHz, $CDCl_3$, 23 ºC, δ): 8.33 (d, *J* = 5.1 Hz, 1H), 7.26 (s, 1H), 7.14 (d, *J* = 5.2 Hz, 1H), 4.56 (dd, *J* = 6.9, 4.9 Hz, 1H), 3.81 (dt, *J* = 9.4, 6.4 Hz, 1H), 3.45 (dt, *J* = 9.4, 6.5 Hz, 1H), 3.04 (dd, *J* = 13.9, 6.9 Hz, 1H), 2.98 (dd, *J* = 13.9, 5.0 Hz, 1H), 1.62 – 1.53 (m, 2H), 1.45 – 1.30 (m, 2H), 0.91 (t, *J* = 7.4 Hz, 3H).
>
> **$^{13}$C NMR** (75 MHz, $CDCl_3$, 23 ºC, δ): 151.7, 149.5, 148.1, 125.3, 123.6, 91.4, 69.6, 40.4, 31.3, 19.2, 13.7.

**HRMS-ESIpos (m/z)** calc'd for $C_{11}H_{15}N_4OClNa$ $[M+Na]^+$, 277.0826; found, 277.0826; deviation: +0.2 ppm.

***rac*-Pyridinylethylazide 46**

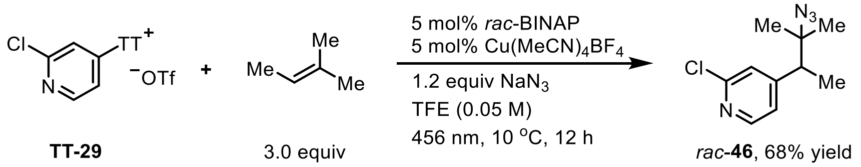


To a 4-mL borosilicate vial containing a Teflon-coated magnetic stirring bar were added **TT-29** (47.7 mg, 0.100 mmol, 1.00 equiv), *rac*-BINAP (3.1 mg, 5.0 μmol, 5.0 mol%), and sodium azide (7.8 mg, 0.12 mmol, 1.2 equiv). The vial was transferred into a nitrogen-filled glovebox. $Cu(MeCN)_4BF_4$ (1.6 mg, 5.0 μmol, 5.0 mol%), dry 2,2,2-trifluoroethanol (TFE, 2 mL, c = 0.05 M), and 2-methyl-2-butene (21 mg, 32 μL, 0.30 mmol, 3.0 equiv) were added. The vial was sealed with a Teflon-lined screw cap and transferred to a cryocooler precooled at 10 °C where the reaction mixture was stirred for 5 min without irradiation and then irradiated by 456 nm LEDs for 12 h. Subsequently, silica gel (5–10 mL) was added, and the reaction mixture was concentrated to dryness under reduced pressure. The resulting residue was purified by chromatography on silica gel (pure DCM as eluent) to afford *rac*-**46** as a colorless oil in 68% yield (15.1 mg).

**R*f*** = 0.23 (pure DCM).

**NMR Spectroscopy:**

> **$^1$H NMR** (300 MHz, $CDCl_3$, 23 ºC, δ): 8.37 – 8.25 (m, 1H), 7.24 (s, 1H), 7.14 (d, *J* = 5.2 Hz, 1H), 2.73 (q, *J* = 7.1 Hz, 1H), 1.39 – 1.32 (m, 6H), 1.23 (s, 3H).
>
> **$^{13}$C NMR** (75 MHz, $CDCl_3$, 23 ºC, δ): 155.1, 151.4, 149.2, 124.7, 123.1, 63.1, 48.7, 24.9, 24.4, 15.5.

**HRMS-ESIpos (m/z)** calc'd for $C_{10}H_{13}N_4ClNa$ $[M+Na]^+$, 247.0721; found, 247.0722; deviation: –0.4 ppm.

**Pyridinylethylazide 47**

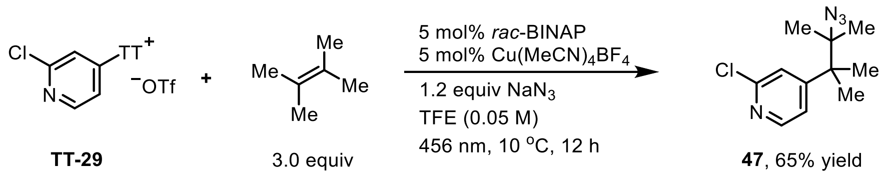

To a 4-mL borosilicate vial containing a Teflon-coated magnetic stirring bar were added **TT-29** (47.7 mg, 0.100 mmol, 1.00 equiv), *rac*-BINAP (3.1 mg, 5.0 μmol, 5.0 mol%), and sodium azide (7.8 mg, 0.12 mmol, 1.2 equiv). The vial was transferred into a nitrogen-filled glovebox. $Cu(MeCN)_4BF_4$ (1.6 mg, 5.0 μmol, 5.0 mol%), dry 2,2,2-trifluoroethanol (TFE, 2 mL, c = 0.05 M), and 2,3-dimethyl-2-butene (25 mg, 35 μL, 0.30 mmol, 3.0 equiv) were added. The vial was sealed with a Teflon-lined screw cap and transferred to a cryocooler precooled at 10 °C where the reaction mixture was stirred for 5 min without irradiation and then irradiated by 456 nm LEDs for 12 h. Subsequently, silica gel (5–10 mL) was added, and the reaction mixture was concentrated to dryness under reduced pressure. The resulting residue was purified by chromatography on silica gel (pure DCM as eluent) to afford **47** as a colorless oil in 65% yield (15.3 mg).

**R*f*** = 0.25 (pure DCM).

**NMR Spectroscopy:**

**$^{1}$H NMR** (300 MHz, $CDCl_3$, 23 ºC, δ): 8.23 (d, *J* = 5.4 Hz, 1H), 7.29 (d, *J* = 1.3 Hz, 1H), 7.21 (dd, *J* = 5.4, 1.7 Hz, 1H), 1.31 (s, 6H), 1.16 (s, 6H).

**$^{13}$C NMR** (75 MHz, $CDCl_3$, 23 ºC, δ): 158.2, 151.3, 148.7, 124.0, 122.3, 65.8, 44.7, 23.7, 22.5.

**HRMS-ESIpos (m/z)** calc'd for $C_{11}H_{16}N_4Cl$ $[M+H]^+$, 239.1058; found, 239.1058; deviation: –0.2 ppm.

**Nootkatone-derived pyridinylethylazide 48**

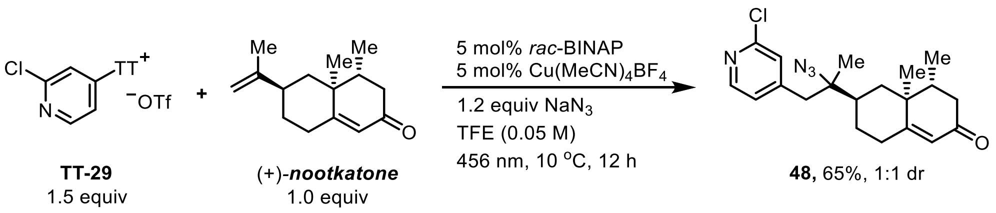

To a 4-mL borosilicate vial containing a Teflon-coated magnetic stirring bar were added **TT-29** (71.6 mg, 0.150 mmol, 1.50 equiv), *rac*-BINAP (3.1 mg, 5.0 μmol, 5.0 mol%), and sodium azide (7.8 mg, 0.12 mmol, 1.2 equiv). The vial was transferred into a nitrogen-filled glovebox. $Cu(MeCN)_4BF_4$ (1.6 mg, 5.0 μmol, 5.0 mol%), dry 2,2,2-trifluoroethanol (TFE, 2 mL, c = 0.05 M), and (+)-nootkatone (22 mg, 0.10 mmol, 1.0 equiv) were added. The vial was sealed with a Teflon-lined screw cap and transferred to a cryocooler precooled at 10 °C where the reaction mixture was stirred for 5 min without irradiation and then irradiated by 456 nm LEDs for 12 h. Subsequently, silica gel (5–10 mL) was added, and the reaction mixture was concentrated to dryness under reduced pressure. The resulting residue was purified by chromatography on silica gel (hexanes/EtOAc

= 3:2) to afford **48** as a colorless oil in 65% yield (24.1 mg).

**R*f*** = 0.25 (hexanes/EtOAc = 3:2).

**NMR Spectroscopy:**

**$^1$H NMR** (500 MHz, $CDCl_3$, 23 ºC, δ): 8.33 (dd, *J* = 5.0, 1.9 Hz, 1H), 7.24 (s, 1H), 7.14 (td, *J* = 4.8, 1.5 Hz, 1H), 5.77 (s, 1H), 2.89 – 2.64 (m, 2H), 2.52 – 2.40 (m, 2H), 2.34 – 2.21 (m, 2H), 2.10 – 1.98 (m, 3H), 1.90 – 1.81 (m, 1H), 1.39 – 1.28 (m, 1H), 1.22 (s, 3H), 1.07 (s, 3H), 1.01 (d, *J* = 5.5 Hz, 3H).

**$^{13}$C NMR** (75 MHz, $CDCl_3$, 23 ºC, δ): 199.20, 199.1, 168.9, 151.6, 151.5, 149.4, 148.9, 148.9, 126.2, 126.2, 124.8, 124.6, 124.6, 65.8, 65.7, 42.8, 42.2, 42.0, 41.8, 40.5, 40.5, 39.4, 39.3, 39.3, 32.6, 32.5, 27.6, 27.6, 20.0, 19.6, 16.9, 16.7, 15.0.

**HRMS-ESIpos (m/z)** calc'd for $C_{20}H_{25}N_4OClNa$ $[M+Na]^+$, 395.1609; found, 395.1608; deviation: +0.4 ppm.

**Phenylethylazide 49**

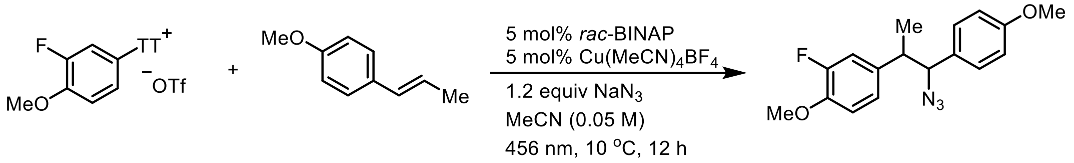


**TT-2** 3.0 equiv **49**, 51% yield, 3.4:1 dr

To a 4-mL borosilicate vial containing a Teflon-coated magnetic stirring bar were added **TT-2** (49.0 mg, 0.100 mmol, 1.00 equiv), *rac*-BINAP (3.1 mg, 5.0 μmol, 5.0 mol%), and sodium azide (7.8 mg, 0.12 mmol, 1.2 equiv). The vial was transferred into a nitrogen-filled glovebox. $Cu(MeCN)_4BF_4$ (1.6 mg, 5.0 μmol, 5.0 mol%), dry MeCN (2 mL, c = 0.05 M), and *trans*-anethole (44 mg, 45 μL, 0.30 mmol, 3.0 equiv) were added. The vial was sealed with a Teflon-lined screw cap and transferred to a cryocooler precooled at 10 °C where the reaction mixture was stirred for 5 min without irradiation and then irradiated by 456 nm LEDs for 12 h. To determine the diastereoselectivity, a 100 µL reaction mixture was extracted and analyzed using $^1$H NMR spectroscopy and GC-MS (3.4:1 dr). Subsequently, silica gel (5–10 mL) was added, and the reaction mixture was concentrated to dryness under reduced pressure. The resulting residue was purified by chromatography on silica gel (hexanes/EtOAc = 20:1) to afford **49** as a colorless oil in 51% yield (16.1 mg).

**R*f*** = 0.18 (hexanes/EtOAc = 20:1).

**NMR Spectroscopy:**

**$^1$H NMR** (500 MHz, $CDCl_3$, 23 ºC, δ): 7.19 (d, *J* = 8.7 Hz, 0.47H), 7.04 (d, *J* = 8.7 Hz, 1.63H), 7.01 – 6.70 (m, 5H), 4.49 (d, *J* = 7.9 Hz, 0.76H), 4.45 (d, *J* = 9.0 Hz, 0.22H), 3.91 (s, 0.70H), 3.85 (s, 3H), 3.80 (s, 2.32H), 3.07 – 2.96 (m, 1H), 1.36 (d, *J* = 6.9 Hz, 2.34H), 1.08 (d, *J* = 7.0 Hz, 0.67H).

**$^{13}$C NMR** (125 MHz, $CDCl_3$, 23 ºC, δ): 159.2, 152.0 (d, *J* = 245.0 Hz), 146.1 (d, *J* = 10.7 Hz), 135.9 (d, *J* = 5.9 Hz), 130.2, 128.6, 123.6 (d, *J* = 3.5 Hz), 115.5 (d, *J* = 18.4 Hz), 113.7, 113.0 (d, *J* = 2.3 Hz), 71.9, 56.2, 55.2, 45.0, 17.8 .

**$^{19}$F NMR** (470 MHz, $CDCl_3$, 23 ºC, δ): –135.47 (dd, *J* = 12.5, 8.5 Hz).

**HRMS-ESIpos (m/z)** calc'd for $C_{17}H_{18}N_3O_2FNa$ $[M+Na]^+$, 338.1275; found, 338.1274; deviation: +0.4 ppm.

***rac*-Phenylethylazide 50**

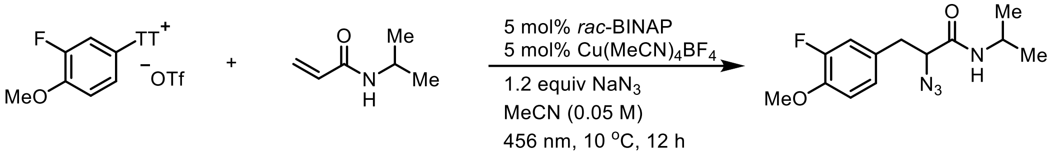


**TT-2** 3.0 equiv *rac*-**50**, 56%

To a 4-mL borosilicate vial containing a Teflon-coated magnetic stirring bar were added **TT-2** (49.0 mg, 0.100 mmol, 1.00 equiv), *rac*-BINAP (3.1 mg, 5.0 μmol, 5.0 mol%), and sodium azide (7.8 mg, 0.12 mmol, 1.2 equiv). The vial was transferred into a nitrogen-filled glovebox. $Cu(MeCN)_4BF_4$ (1.6 mg, 5.0 μmol, 5.0 mol%), dry MeCN (2 mL, c = 0.05 M), and *N*-isopropylacrylamide (34 mg, 0.30 mmol, 3.0 equiv) were added. The vial was sealed with a Teflon-lined screw cap and transferred to a cryocooler precooled at 10 °C where the reaction mixture was stirred for 5 min without irradiation and then irradiated by 456 nm LEDs for 12 h. Subsequently, silica gel (5–10 mL) was added, and the reaction mixture was concentrated to dryness under reduced pressure. The resulting residue was purified by chromatography on silica gel (hexanes/EtOAc = 4:1) to afford *rac*-**50** as a colorless oil in 56% yield (15.6 mg).

**R*f*** = 0.17 (hexanes/EtOAc = 4:1).

**NMR Spectroscopy:**

**$^1$H NMR** (500 MHz, $CDCl_3$, 23 ºC, δ): 7.01 (dd, *J* = 12.0, 2.1 Hz, 1H), 6.99 – 6.96 (m, 1H), 6.92 (t, *J* = 8.4 Hz, 1H), 6.01 (d, *J* = 8.1 Hz, 1H), 4.14 (dd, *J* = 7.3, 4.4 Hz, 1H), 4.10 – 4.00 (m, 1H), 3.89 (s, 3H), 3.24 (dd, *J* = 14.2, 4.4 Hz, 1H), 3.02 (dd, *J* = 14.2, 7.3 Hz, 1H), 1.15 (d, *J* = 6.6 Hz, 3H), 1.08 (d, *J* = 6.6 Hz, 3H).

**$^{13}$C NMR** (125 MHz, $CDCl_3$, 23 ºC, δ): 167.2, 152.2 (d, *J* = 245.9 Hz), 146.8 (d, *J* = 10.4 Hz), 128.9 (d, *J* = 6.4 Hz), 125.4 (d, *J* = 3.6 Hz), 117.2 (d, *J* = 18.4 Hz), 113.4 (d, *J* = 2.5 Hz), 65.4, 56.3, 41.5, 37.5, 22.5, 22.5.

**$^{19}$F NMR** (471 MHz, $CDCl_3$, 23 ºC, δ) δ -135.03 (dd, *J* = 12.1, 8.6 Hz).

**HRMS-ESIpos (m/z)** calc'd for $C_{13}H_{17}N_4O_2FNa$ $[M+Na]^+$, 303.1228; found, 303.1226; deviation: +0.6 ppm.

**Valinate-derived phenylethylazide 51**

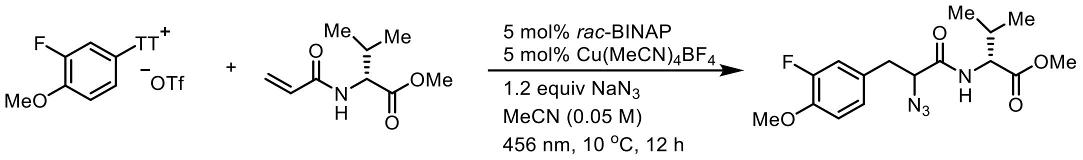


**TT-2** 1.5 equiv    1.0 equiv    **51,** 45%, 1.3:1 dr

To a 4-mL borosilicate vial containing a Teflon-coated magnetic stirring bar were added **TT-2** (74.0 mg, 0.150 mmol, 1.50 equiv), *rac*-BINAP (3.1 mg, 5.0 μmol, 5.0 mol%), and sodium azide (7.8 mg, 0.12 mmol, 1.2 equiv). The vial was transferred into a nitrogen-filled glovebox. $Cu(MeCN)_4BF_4$ (1.6 mg, 5.0 μmol, 5.0 mol%), dry MeCN (2 mL, c = 0.05 M), and methyl acryloyl-*L*-valinate (19 mg, 0.10 mmol, 1.0 equiv) were added. The vial was sealed with a Teflon-lined screw cap and transferred to a cryocooler precooled at 10 °C where the reaction mixture was stirred for 5 min without irradiation and then irradiated by 456 nm LEDs for 12 h. Subsequently, silica gel (5–10 mL) was added, and the reaction mixture was concentrated to dryness under reduced pressure. The resulting residue was purified by chromatography on silica gel (hexanes/EtOAc = 4:1) to afford **51** as a colorless oil in 45% yield (15.7 mg).

**R*f*** = 0.30 (hexanes/EtOAc = 2:1).

**NMR Spectroscopy:**

**$^1$H NMR** (500 MHz, $CDCl_3$, 23 °C, δ): 7.06 – 6.96 (m, 2H), 6.95 – 6.88 (m, 1H), 6.74 (d, *J* = 8.8 Hz, 1H), 4.49 (td, *J* = 8.5, 5.0 Hz, 1H), 4.22 (dd, *J* = 7.5, 4.2 Hz, 1H), 3.88 (s, 3H), 3.75 (s, 3H), 3.24 (t, *J* = 4.1 Hz, 1H), 3.03 (dd, *J* = 14.3, 7.5 Hz, 1H), 2.21 – 2.07 (m, 1H), 0.91 (dd, *J* = 11.0, 6.9 Hz, 3H), 0.83 (dd, *J* = 9.9, 6.9 Hz, 3H).

**$^{13}$C NMR** (125 MHz, $CDCl_3$, 23 °C, δ): 171.8, 171.6, 168.4, 168.1, 152.3 (d, *J* = 246.3 Hz), 152.2 (d, *J* = 246.1 Hz), 146.9 (d, *J* = 10.7 Hz), 146.9 (d, *J* = 10.6 Hz), 128.9 (d, *J* = 6.3 Hz), 128.7 (d, *J* = 6.1 Hz), 125.4 (d, *J* = 3.5 Hz), 125.3 (d, *J* = 3.5 Hz), 117.2 (d, *J* = 18.4 Hz), 117.0 (d, *J* = 18.4 Hz), 113.5 (d, *J* = 2.3 Hz), 65.4, 65.3, 57.2, 56.3, 56.3, 52.3, 52.2, 37.6, 37.4, 31.2, 31.1, 18.9, 18.7, 17.8, 17.7.

**$^{19}$F NMR** (471 MHz, $CDCl_3$, 23 °C, δ) δ –134.94 (dd, *J* = 12.0, 8.5 Hz).

**HRMS-ESIpos (m/z)** calc'd for $C_{16}H_{21}N_4O_4NaF$ $[M+Na]^+$, 375.1439; found, 375.1437; deviation: +0.5 ppm.

***rac*-Fenofibrate-derived phenylethylazide 52**

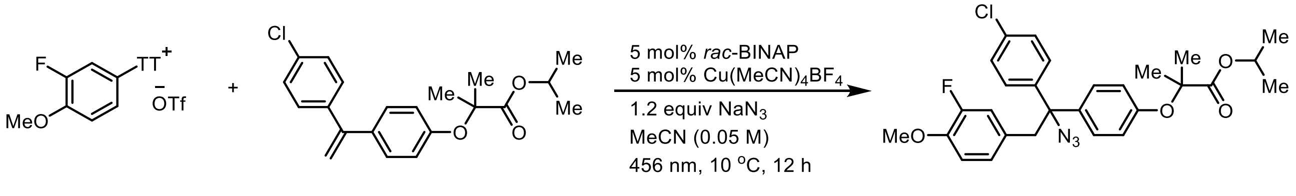


**TT-2** 1.5 equiv    1.0 equiv    **52,** 57% *from* ***fenofibrate***

To a 4-mL borosilicate vial containing a Teflon-coated magnetic stirring bar were added **TT-2** (74.0 mg, 0.150 mmol, 1.50 equiv), *rac*-BINAP (3.1 mg, 5.0 μmol, 5.0 mol%), and sodium azide (7.8 mg, 0.12 mmol, 1.2 equiv). The vial was transferred into a nitrogen-filled glovebox. $Cu(MeCN)_4BF_4$ (1.6 mg, 5.0 μmol, 5.0 mol%), dry MeCN (2 mL, c = 0.05 M), and fenofibrate-derived olefin (36 mg, 0.10 mmol, 1.0 equiv) were added. The vial was sealed with a Teflon-lined screw cap and transferred to a cryocooler precooled at 10 °C where the reaction mixture was stirred for 5 min without irradiation and then irradiated by 456 nm LEDs for 12 h. Subsequently, silica gel (5–10 mL) was added, and the reaction mixture was concentrated to dryness under reduced pressure. The resulting residue was purified by chromatography on silica gel (hexanes/EtOAc = 20:1) to afford *rac*-**52** as a colorless oil in 57% yield (29.8 mg).

**$R_f$** = 0.19 (hexanes/EtOAc = 20:1).

**NMR Spectroscopy:**

**$^1$H NMR** (500 MHz, $CDCl_3$, 23 ºC, δ): 7.31 – 7.26 (m, 2H), 7.14 (d, *J* = 8.7 Hz, 2H), 7.11 (d, *J* = 8.8 Hz, 2H), 6.81 (d, *J* = 8.8 Hz, 2H), 6.72 (t, *J* = 8.7 Hz, 1H), 6.48 (s, 1H), 6.47 – 6.44 (m, 1H), 5.09 (hept, *J* = 6.3 Hz, 1H), 3.84 (s, 3H), 3.55 (d, *J* = 13.5 Hz, 1H), 3.48 (d, *J* = 13.5 Hz, 1H), 1.62 (s, 6H), 1.22 (dd, *J* = 6.3, 2.5 Hz, 6H).

**$^{13}$C NMR** (125 MHz, $CDCl_3$, 23 ºC, δ): 173.5, 155.2, 151.5 (d, *J* = 244.7 Hz), 146.4 (d, *J* = 10.3 Hz), 141.1, 135.1, 133.5, 128.8, 128.3, 128.2, 128.2 (d, *J* = 6.3 Hz), 126.2 (d, *J* = 3.5 Hz), 118.3, 118.0 (d, *J* = 18.5 Hz), 112.5 (d, *J* = 2.2 Hz), 79.2, 72.4, 69.0, 56.1, 44.2, 25.4, 25.4, 21.5.

**$^{19}$F NMR** (471 MHz, $CDCl_3$, 23 ºC, δ) δ –136.08 (dd, *J* = 12.3, 8.7 Hz).

**HRMS-ESIpos (m/z)** calc'd for $C_{28}H_{29}N_3O_4FClNa$ $[M+H]^+$, 548.1723; found, 548.1723; deviation: +0.0 ppm.

## Unsuccessful examples

**Limitations of arylthianthrenium salts**

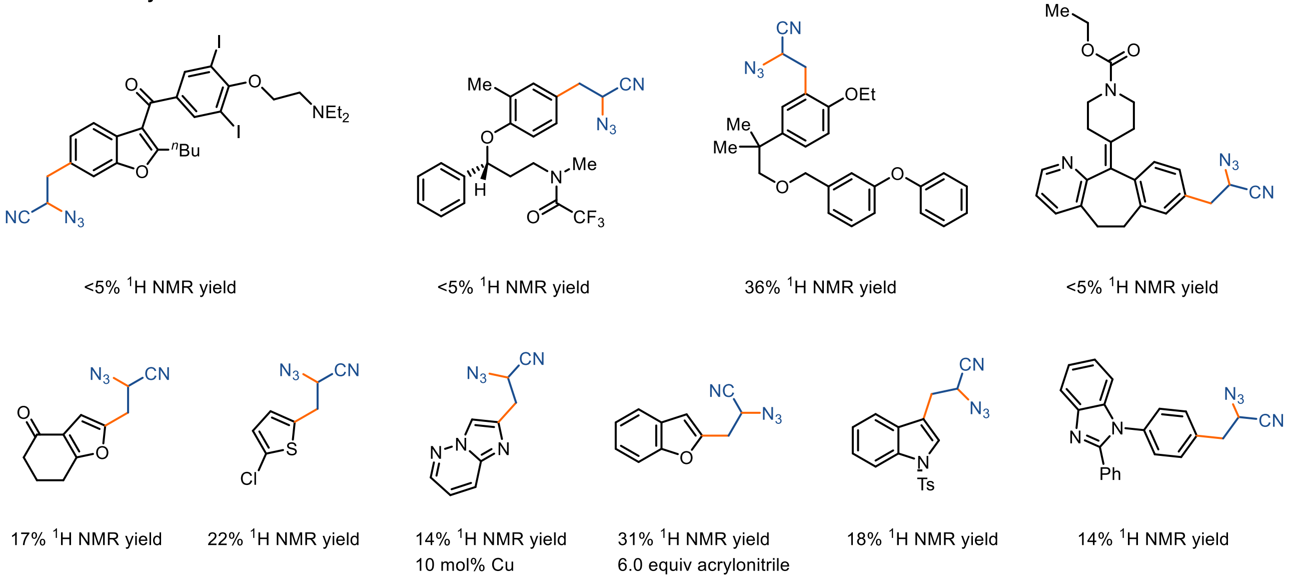


**Figure S2**. Arylthianthrenium salts that out of the scope of current approach.

**Limitations of alkenes**

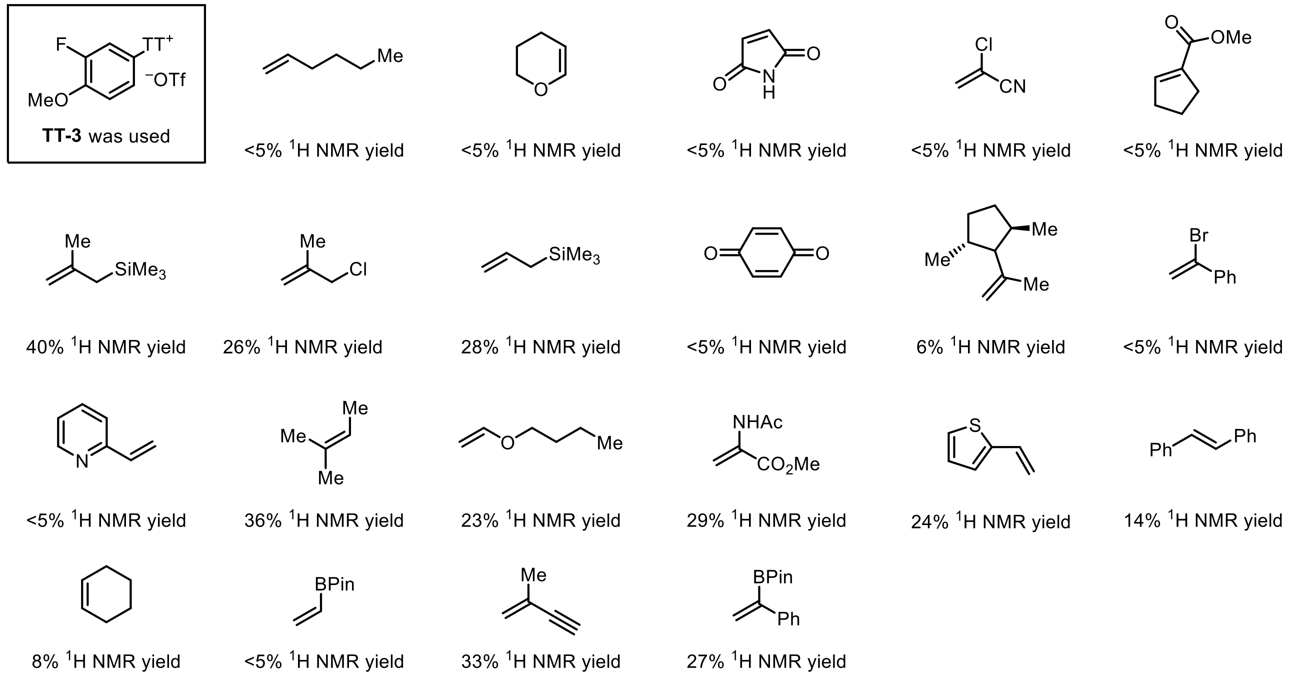


**Figure S3**. Alkenes that out of the scope of current approach.

## Synthesis of racemic melphalan

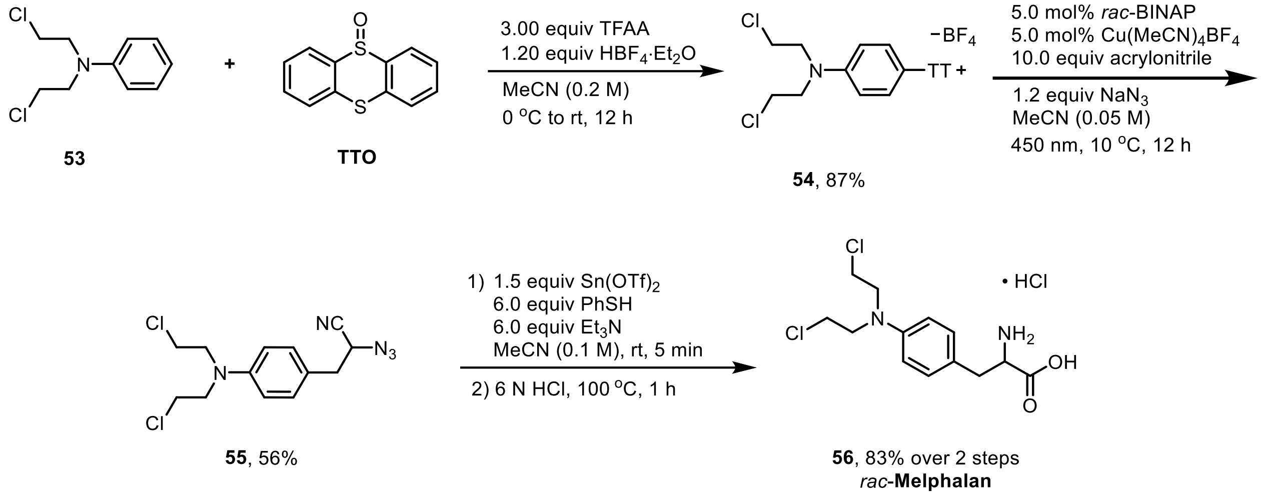


**Scheme S1**. Synthesis routes of racemic melphalan from *N,N*-bis(2-chloroethyl)aniline.

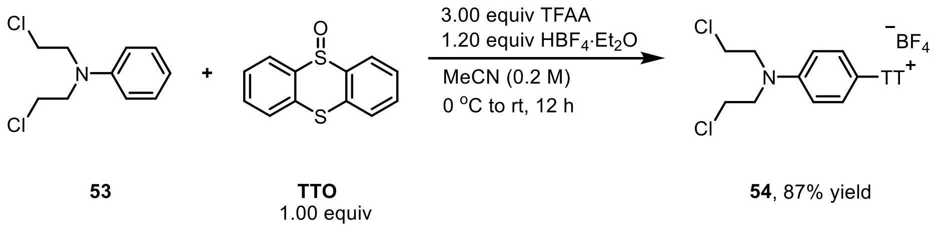


Under an ambient atmosphere, a 20 mL glass vial was charged with *N,N*-bis(2-chloroethyl)aniline (218 mg, 1.00 mmol, 1.00 equiv), **TTO** (232 mg, 1.00 mmol, 1.00 equiv), and dry MeCN (5 mL). Trifluoroacetic anhydride (630 mg, 417 μL, 3.00 mmol, 3.00 equiv) and $HBF_4$·$Et_2O$ (195 mg, 150 μL, 1.20 mmol, 1.20 equiv) were added respectively at 0 °C. The mixture was gradually warmed to room temperature and stirred overnight. 10 mL saturated aqueous $NaHCO_3$ solution was added slowly. The mixture was stirred for 10 min, extracted by DCM (2 × 10 mL), and washed with aqueous $NaBF_4$ solution (3 × 10 mL, 5 % w/w). The organic layer was dried over $Na_2SO_4$, filtered, and the solvent was removed under reduced pressure. The resulting residue was purified by flash column chromatography on silica gel (DCM/MeOH = 50:1 to 30:1) to afford **54** as a brown solid in 87% yield (450 mg).

**R*f*** = 0.18 (DCM/MeOH = 30:1).

**NMR Spectroscopy:**

**$^1$H NMR** (500 MHz, $CDCl_3$, 23 ºC, δ): 8.22 (dd, *J* = 7.9, 1.3 Hz, 2H), 7.83 (dd, *J* = 7.9, 1.3 Hz, 2H), 7.77 (td, *J* = 7.7, 1.4 Hz, 2H), 7.69 (td, *J* = 7.7, 1.4 Hz, 2H), 7.39 (d, *J* = 9.3 Hz, 2H), 6.81 (d, *J* = 9.4 Hz, 2H), 3.80 (t, *J* = 6.6 Hz, 4H), 3.64 (t, *J* = 6.6 Hz, 4H).

**$^{13}$C NMR** (125 MHz, $CDCl_3$, 23 ºC, δ): 151.0, 135.3, 134.2, 133.3, 131.8, 130.2, 130.1, 120.9, 113.8, 106.8, 53.2, 40.2.

**$^{19}$F NMR** (470 MHz, $CDCl_3$, 23 ºC, δ): –151.56 – –151.70 (m).

**HRMS-ESIpos (m/z)** calc'd for $C_{22}H_{20}Cl_2NS_2$ $[M–BF_4]^+$, 432.0409; found, 432.0414; deviation: –1.1 ppm.

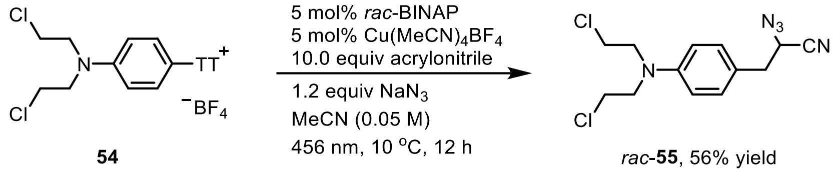


To a 4-mL borosilicate vial containing a Teflon-coated magnetic stirring bar were added **54** (51.8 mg, 0.100 mmol, 1.00 equiv), *rac*-BINAP (3.1 mg, 5.0 μmol, 5.0 mol%), and sodium azide (7.8 mg, 0.12 mmol, 1.2 equiv). The vial was transferred into a nitrogen-filled glovebox. $Cu(MeCN)_4BF_4$ (1.6 mg, 5.0 μmol, 5.0 mol%), dry MeCN (2 mL, c = 0.05 M), and acrylonitrile (53.1 mg, 65.8 μL, 1.00 mmol, 10.0 equiv) were added. The vial was sealed with a Teflon-lined screw cap and transferred to a cryocooler precooled at 10 °C where the reaction mixture was stirred for 5 min without irradiation and then irradiated by 456 nm LEDs for 12 h. Subsequently, silica gel (5–10 mL) was added, and the reaction mixture was concentrated to dryness under reduced pressure. The resulting residue was purified by chromatography on silica gel (hexanes/EtOAc = 10:1) to afford *rac*-**55** as a colorless oil in 56% yield (17.5 mg).

**R*f*** = 0.21 (hexanes/EtOAc = 10:1).

**NMR Spectroscopy:**

**$^1$H NMR** (500 MHz, $CDCl_3$, 23 ºC, δ): 7.19 (d, *J* = 8.7 Hz, 2H), 6.70 (d, *J* = 8.7 Hz, 2H), 4.31 (t, *J* = 6.9 Hz, 1H), 3.76 (t, *J* = 7.1 Hz, 4H), 3.66 (t, *J* = 7.0 Hz, 4H), 3.05 (d, *J* = 7.0 Hz, 2H).

**$^{13}$C NMR** (125 MHz, $CDCl_3$, 23 ºC, δ): 145.9, 130.8, 122.2, 116.0, 112.2, 53.4, 52.8, 40.3, 38.1.

**HRMS-ESIpos (m/z)** calc'd for $C_{13}H_{15}N_5Cl_2Na$ $[M+Na]^+$, 334.0597; found, 334.0597; deviation: –0.1 ppm.

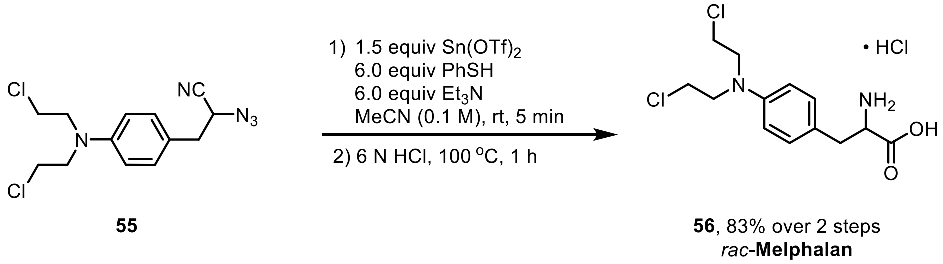


Prepared according to a modified reaction condition[5]: under an ambient atmosphere, a 4 mL vial was charged with $Sn(OTf)_2$ (31 mg, 0.75 mmol, 1.5 equiv) and acetonitrile (0.5 mL, c = 0.1 M). Thiophenol (25 mg, 23 μL, 0.26 mmol, 4.5 equiv) and triethylamine (26 mg, 31 μL, 0.26 mmol, 4.5 equiv) were added. The mixture was stirred at room temperature for 5 min then *rac*-**55** (15.6 mg, 50.0 μmol, 1.00 equiv) was added. The mixture was stirred at room temperature for another 5 min. The mixture was diluted with EA (10 mL), washed with 2.0 M NaOH aqueous solution (10 mL), and then washed with 3.0 M aqueous HCl solution. The aqueous layer was adjusted with 2.0 M NaOH solution to pH ~ 8 until the solution was turbid, then extracted

with EA (2 × 10 mL). The combined organic layer was brined and dried over $Na_2SO_4$, filtered, and the solvent was removed under reduced pressure. The residue was dissolved in 6 N HCl (0.25 mL, c = 0.2 M). The mixture was heated at 100 °C for 1 h and then cooled to rt. The solvent was removed under reduced pressure to afford pure racemic melphalan **56** in 83% yield (14.3 mg).

**NMR Spectroscopy:**

**$^1$H NMR** (300 MHz, $D_2O$, 23 ºC, δ): 7.61 – 7.46 (m, 4H), 4.33 (dd, *J* = 7.2, 6.3 Hz, 1H), 4.02 (t, *J* = 6.1 Hz, 4H), 3.58 (t, *J* = 6.1 Hz, 4H), 3.35 (dd, *J* = 14.6, 6.3 Hz, 1H), 3.27 (dd, *J* = 14.6, 7.2 Hz, 1H).

**$^{13}$C NMR** (75 MHz, $D_2O$, 23 ºC, δ): 171.0, 136.1, 135.4, 131.8, 122.0, 58.4, 53.7, 37.6, 35.1.

**HRMS-ESIpos (m/z)** calc'd for $C_{13}H_{19}N_2O_2Cl_2$ $[M–Cl]^+$, 305.0818; found, 305.0816; deviation: +0.6 ppm

## Synthesis of arylthianthrenium salts

### Nimesulide-derived thianthrenium salt TT-5

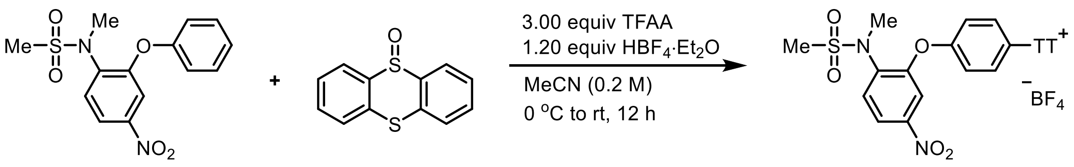


**S5** **TTO** 1.00 equiv **TT-5**, 72% yield

Under an ambient atmosphere, a 20 mL glass vial was charged with nimesulide **S5** (312 mg, 1.00 mmol, 1.00 equiv), **TTO** (232 mg, 1.00 mmol, 1.00 equiv), and dry MeCN (5 mL). Trifluoroacetic anhydride (630 mg, 417 μL, 3.00 mmol, 3.00 equiv) and $HBF_4·Et_2O$ (195 mg, 150 μL, 1.20 mmol, 1.20 equiv) were added respectively at 0 °C. The mixture was gradually warmed to room temperature and stirred overnight. 10 mL saturated aqueous $NaHCO_3$ solution was added slowly. The mixture was stirred for 10 min, extracted by DCM (2 × 10 mL) and washed with aqueous $NaBF_4$ solution (3 × 10 mL, 5 % w/w). The organic layer was dried over $Na_2SO_4$, filtered, and the solvent was removed under reduced pressure. The resulting residue was purified by flash column chromatography on silica gel (DCM/MeOH = 50:1 to 30:1) to afford **TT-5** as a colorless solid in 72% yield (443 mg).

**$R_f$** = 0.18 (DCM/MeOH = 30:1).

**NMR Spectroscopy:**

**$^1$H NMR** (500 MHz, $CDCl_3$, 23 ºC, δ): 8.55 (dd, *J* = 8.0, 1.5 Hz, 2H), 8.06 (dd, *J* = 8.8, 2.6 Hz, 1H), 7.90 (dd, *J* = 7.9, 1.5 Hz, 2H), 7.85 (td, *J* = 7.7, 1.4 Hz, 2H), 7.79 (td, *J* = 7.6, 1.5 Hz, 2H), 7.75 (d, *J* = 2.6 Hz, 1H), 7.67 (d, *J* = 8.7 Hz, 1H), 7.32 (d, *J* = 9.1 Hz, 2H), 7.14 (d, *J* = 9.1 Hz, 2H), 3.22 (s, 3H), 2.94 (s, 3H).

**$^{13}$C NMR** (125 MHz, $CDCl_3$, 23 ºC, δ): 159.7, 152.3, 147.4, 139.0, 136.5, 135.2, 135.1, 131.6, 131.0, 130.5, 130.4, 120.5, 120.3, 118.7, 118.7, 115.7, 38.6, 37.9.

**$^{19}$F NMR** (470 MHz, $CDCl_3$, 23 ºC, δ): –150.51 – –150.89 (m).

**HRMS-ESIpos (m/z)** calc'd for $C_{26}H_{21}N_2O_5S_3$ $[M–BF_4]^+$, 537.0607; found, 537.0613; deviation: –1.0 ppm.

**Meclofenamic acid-derived thianthrenium salt TT-6**

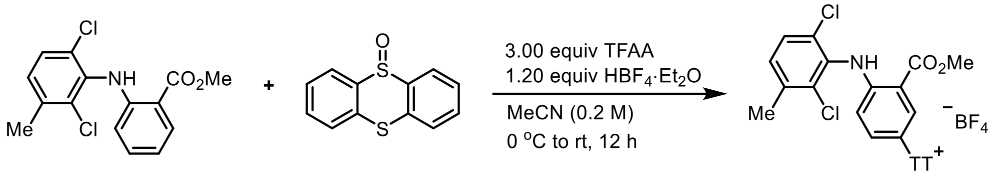


**S6** **TTO** 1.00 equiv **TT-6**, 83% yield

Under an ambient atmosphere, a 20 mL glass vial was charged with methyl meclofenamate **S6** (309 mg, 1.00 mmol, 1.00 equiv), **TTO** (232 mg, 1.00 mmol, 1.00 equiv), and dry MeCN (5 mL). Trifluoroacetic anhydride (630 mg, 417 μL, 3.00 mmol, 3.00 equiv) and $HBF_4·Et_2O$ (195 mg, 150 μL, 1.20 mmol, 1.20 equiv) were added respectively at 0 °C. The mixture was gradually warmed to room temperature and stirred overnight. 10 mL saturated aqueous $NaHCO_3$ solution was added slowly. The mixture was stirred for 10 min, extracted by DCM (2 × 10 mL) and washed with aqueous $NaBF_4$ solution (3 × 10 mL, 5 % w/w). The organic layer was dried over $Na_2SO_4$, filtered, and the solvent was removed under reduced pressure. The resulting residue was purified by flash column chromatography on silica gel (DCM/MeOH = 50:1 to 30:1) to afford **TT-6** as a colorless solid in 83% yield (505 mg).

**R*f*** = 0.20 (DCM/MeOH = 30:1).

**NMR Spectroscopy:**

**$^{1}$H NMR** (500 MHz, $CDCl_3$, 23 ºC, δ): 9.80 (s, 1H), 8.41 (d, *J* = 7.9 Hz, 2H), 8.09 (d, *J* = 2.7 Hz, 1H), 7.86 – 7.77 (m, 4H), 7.71 (t, *J* = 7.6 Hz, 2H), 7.35 – 7.24 (m, 2H), 7.18 (d, *J* = 8.2 Hz, 1H), 6.32 (d, *J* = 9.3 Hz, 1H), 3.91 (s, 3H), 2.37 (s, 3H).

**$^{13}$C NMR** (125 MHz, $CDCl_3$, 23 ºC, δ): 166.8, 151.3, 136.9, 135.8, 134.6, 134.3, 134.2, 133.4, 133.3, 132.6, 131.1, 130.2, 130.1, 130.1, 127.9, 119.3 (d, *J* = 2.0 Hz), 116.0, 112.6, 109.5, 52.6, 20.4.

**$^{19}$F NMR** (470 MHz, $CDCl_3$, 23 ºC, δ): –151.28, –151.33.

**HRMS-ESIpos (m/z)** calc'd for $C_{27}H_{20}Cl_2NO_2S_2$ $[M–BF_4]^+$, 524.0307; found, 524.0312; deviation: –1.0 ppm.

**Benzbromarone-derived thianthrenium salt TT-7**

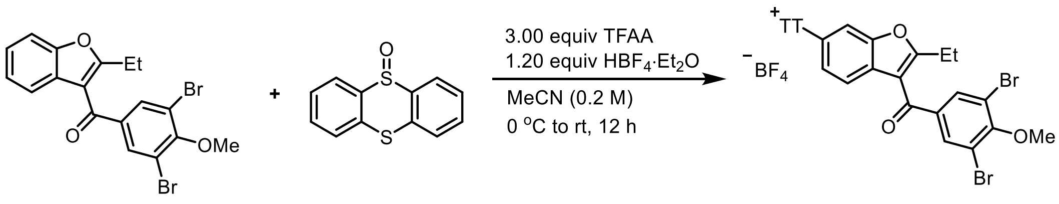


**S7** **TTO** 1.00 equiv **TT-7**, 68% yield

Under an ambient atmosphere, a 20 mL glass vial was charged with benzbromarone **S7** (437 mg, 1.00 mmol, 1.00 equiv), **TTO** (232 mg, 1.00 mmol, 1.00 equiv), and dry MeCN (5 mL). Trifluoroacetic anhydride (630 mg, 417 μL, 3.00 mmol, 3.00 equiv) and $HBF_4·Et_2O$ (195 mg, 150 μL, 1.20 mmol, 1.20 equiv) were added respectively at 0 °C. The mixture was gradually warmed to room temperature and stirred overnight. 10 mL saturated aqueous $NaHCO_3$ solution was added slowly. The mixture was stirred for 10 min, extracted by DCM (2 × 10 mL) and washed with aqueous $NaBF_4$ solution (3 × 10 mL, 5 % w/w). The organic layer was dried over $Na_2SO_4$, filtered, and the solvent was removed under reduced pressure. The resulting residue was purified by flash column chromatography on silica gel (DCM/MeOH = 50:1 to 30:1) to afford **TT-7** as a colorless solid in 68% yield (503 mg).

**R*f*** = 0.20 (DCM/MeOH = 30:1).

**NMR Spectroscopy:**

**$^1$H NMR** (500 MHz, $CDCl_3$, 23 ºC, δ): 8.62 – 8.51 (m, 2H), 7.79 (s, 2H), 7.78 – 7.67 (m, 6H), 7.46 (d, *J* = 8.5 Hz, 1H), 7.35 (d, *J* = 1.8 Hz, 1H), 6.97 (dd, *J* = 8.5, 1.9 Hz, 1H), 3.88 (s, 3H), 2.73 (q, *J* = 7.5 Hz, 2H), 1.22 (t, *J* = 7.5 Hz, 3H).

**$^{13}$C NMR** (125 MHz, $CDCl_3$, 23 ºC, δ): 186.8, 169.8, 158.2, 153.1, 136.4, 136.2, 135.5, 135.0, 133.4, 131.3, 130.4, 130.3, 123.1, 122.9, 119.9, 119.0, 118.8, 115.3, 112.1, 60.9, 22.3, 11.8.

**$^{19}$F NMR** (470 MHz, $CDCl_3$, 23 ºC, δ): –150.69, –150.70.

**HRMS-ESIpos (m/z)** calc'd for $C_{30}H_{21}Br_2O_3S_2$ $[M–BF_4]^+$, 650.9293; found, 650.9299; deviation: –0.9 ppm.

**Estrone-derived thianthrenium salt TT-10**

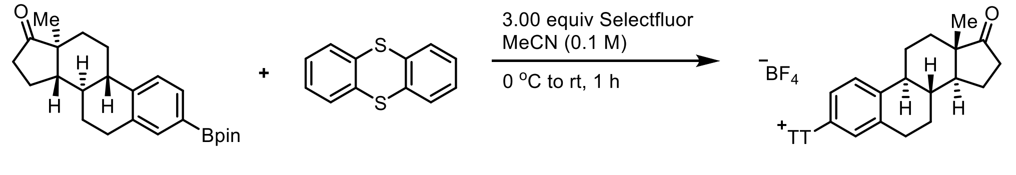


Under an ambient atmosphere, a 20 mL glass vial was charged with estrone-derived boronic ester **S10** (379 mg, 1.00 mmol, 1.00 equiv), **TT** (648 mg, 3.00 mmol, 3.00 equiv), Selectfluor (1.06 g, 3.00 mmol, 3.00 equiv) and dry MeCN (10 mL) at 0 °C. The mixture was gradually warmed to room temperature and stirred for 1 h. Subsequently, silica gel (5–10 mL) was added, and the reaction mixture was concentrated to dryness under reduced pressure. The resulting residue was purified by flash column chromatography on silica gel (hex/EA = 1:1 then DCM/MeOH = 50:1 to 30:1) to afford **TT-10** as a colorless solid in 49% yield (270 mg).

**R*f*** = 0.25 (DCM/MeOH = 30:1).

**NMR Spectroscopy:**

**$^1$H NMR** (500 MHz, $CDCl_3$, 23 ºC, δ): 8.57 (d, *J* = 7.9 Hz, 2H), 7.88 – 7.75 (m, 6H), 7.32 (d, *J* = 9.1 Hz,

1H), 7.04 (d, $J$ = 2.4 Hz, 1H), 6.86 (dd, $J$ = 8.6, 2.4 Hz, 1H), 2.88 – 2.81 (m, 2H), 2.50 (dd, $J$ = 19.4, 8.6 Hz, 1H), 2.32 – 2.20 (m, 2H), 2.13 (dd, $J$ = 19.2, 9.0 Hz, 1H), 2.08 – 1.96 (m, 2H), 1.97 – 1.91 (m, 1H), 1.56 – 1.35 (m, 6H), 0.87 (s, 3H).

**$^{13}$C NMR** (125 MHz, $CDCl_3$, 23 ºC, δ): 220.0, 146.0, 140.5, 136.4, 135.3, 134.7, 130.2, 130.1, 128.5, 127.6, 124.8, 120.6, 119.0, 50.3, 47.7, 44.2, 37.2, 35.7, 31.3, 29.2, 25.7, 25.3, 21.4, 13.7.

**$^{19}$F NMR** (470 MHz, $CDCl_3$, 23 ºC, δ): –150.98, –150.99.

**HRMS-ESIpos (m/z)** calc'd for $C_{30}H_{29}OS_2$ $[M–BF_4]^+$, 469.1654; found, 469.1656; deviation: –0.4 ppm.

**Tianeptine intermediate-derived thianthrenium salt TT-11**

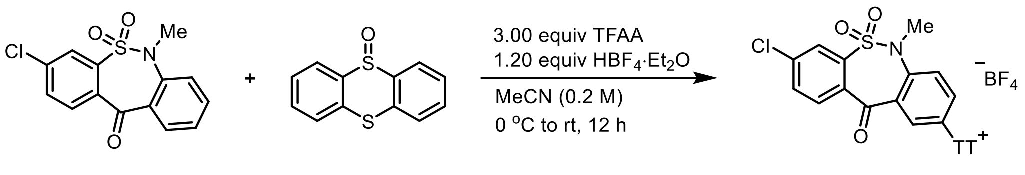


**S11** **TTO** 1.00 equiv **TT-11**, 73% yield

Under an ambient atmosphere, a 20 mL glass vial was charged with tianeptine intermediate **S11** (308 mg, 1.00 mmol, 1.00 equiv), **TTO** (232 mg, 1.00 mmol, 1.00 equiv), and dry MeCN (5 mL). Trifluoroacetic anhydride (630 mg, 417 μL, 3.00 mmol, 3.00 equiv) and $HBF_4·Et_2O$ (195 mg, 150 μL, 1.20 mmol, 1.20 equiv) were added respectively at 0 °C. The mixture was gradually warmed to room temperature and stirred overnight. 10 mL saturated aqueous $NaHCO_3$ solution was added slowly. The mixture was stirred for 10 min, extracted by DCM (2 × 10 mL) and washed with aqueous $NaBF_4$ solution (3 × 10 mL, 5 % w/w). The organic layer was dried over $Na_2SO_4$, filtered, and the solvent was removed under reduced pressure. The resulting residue was purified by flash column chromatography on silica gel (DCM/MeOH = 50:1 to 30:1) to afford **TT-11** as a colorless solid in 73% yield (446 mg).

**$R_f$** = 0.18 (DCM/MeOH = 30:1).

**NMR Spectroscopy:**

**$^1$H NMR** (500 MHz, $CDCl_3$, 23 ºC, δ): 8.60 (d, $J$ = 7.4 Hz, 2H), 7.94 (d, $J$ = 2.7 Hz, 1H), 7.93 – 7.85 (m, 5H), 7.83 (td, $J$ = 7.6, 1.6 Hz, 2H), 7.78 (d, $J$ = 8.3 Hz, 1H), 7.70 (dd, $J$ = 8.3, 2.1 Hz, 1H), 7.66 – 7.58 (m, 1H), 7.47 (dd, $J$ = 8.9, 1.1 Hz, 1H), 3.42 (s, 3H).

**$^{13}$C NMR** (125 MHz, $CDCl_3$, 23 ºC, δ): 187.7, 144.8, 139.3, 137.9, 136.7, 135.5, 135.2, 134.0, 133.8, 133.4, 133.2, 131.5, 130.7, 130.5, 130.5, 125.4, 124.6, 120.3, 118.3, 38.2.

**$^{19}$F NMR** (470 MHz, $CDCl_3$, 23 ºC, δ): –150.63, –150.64.

**HRMS-ESIpos (m/z)** calc'd for $C_{26}H_{17}ClNO_3S_3$ $[M–BF_4]^+$, 522.0054; found, 522.0058; deviation: –0.9 ppm.

**Quinoline-derived thianthrenium salt TT-14**

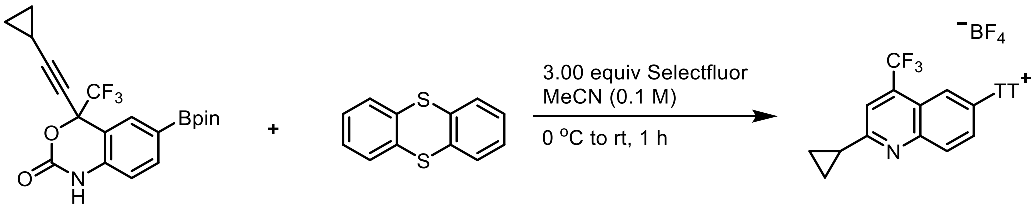


**S14** **TT** 3.00 equiv **TT-14**, 36% yield

Under an ambient atmosphere, a 20 mL glass vial was charged with efavirenz derived boronic ester **S14** (406 mg, 1.00 mmol, 1.00 equiv), **TT** (648 mg, 3.00 mmol, 3.00 equiv), Selectfluor (1.06 g, 3.00 mmol, 3.00 equiv) and dry MeCN (10 mL) at 0 °C. The mixture was gradually warmed to room temperature and stirred for 1 h. Subsequently, silica gel (5–10 mL) was added, and the reaction mixture was concentrated to dryness under reduced pressure. The resulting residue was purified by flash column chromatography on silica gel (hex/EA = 1:1 then DCM/MeOH = 50:1 to 30:1) to afford **TT-14** as a brown solid in 36% yield (210 mg).

**R*f*** = 0.18 (DCM/MeOH = 30:1).

**NMR Spectroscopy:**

**$^1$H NMR** (600 MHz, $CDCl_3$, 23 ºC, δ): 8.70 (dd, *J* = 7.7, 1.3 Hz, 2H), 8.05 (d, *J* = 9.1 Hz, 1H), 7.88 – 7.78 (m, 6H), 7.58 (s, 1H), 7.55 (dd, *J* = 9.2, 2.3 Hz, 1H), 7.44 (s, 1H), 2.24 (tt, *J* = 8.0, 4.7 Hz, 1H), 1.26 – 1.23 (m, 2H), 1.21 – 1.17 (m, 2H).

**$^{13}$C NMR** (151 MHz, $CDCl_3$, 23 ºC, δ): 167.9, 149.6, 136.8, 135.9, 135.4, 134.0 (q, *J* = 32.4 Hz), 132.9, 130.5, 130.2, 127.4, 124.8 (q, *J* = 2.4 Hz), 122.7 (q, *J* = 274.9 Hz), 121.6, 120.7, 119.8 (q, *J* = 5.0 Hz), 118.5 (d, *J* = 1.5 Hz), 18.8, 12.8.

**$^{19}$F NMR** (565 MHz, $CDCl_3$, 23 ºC, δ): –61.64 (s, 3H), –150.58, –150.63.

**HRMS-ESIpos (m/z)** calc'd for $C_{25}H_{17}NF_3S_2$ $[M–BF_4]^+$, 452.0749; found, 452.0750; deviation: –0.3 ppm.

**A plausible rearrangement pathway:**

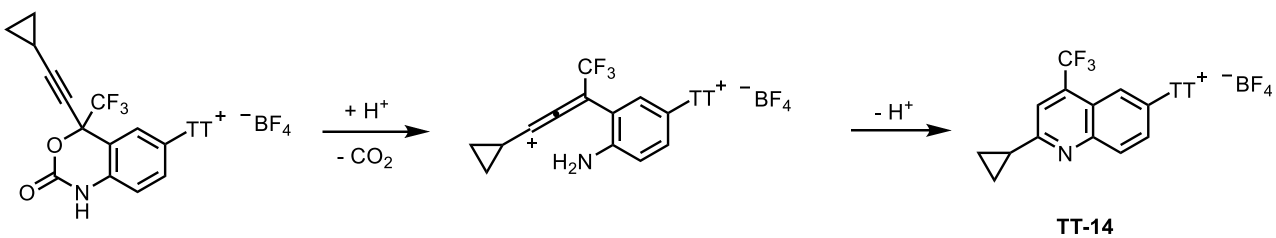


**TT-14**

The structure of the rearranged product **TT-14** was supported by HRMS and 2D NMR analysis (see pages S227 to S233) :

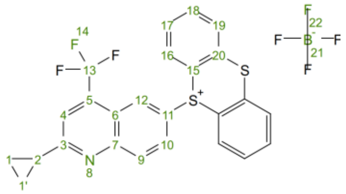

| Atom | δ (ppm) | J | COSY | HSQC | HMBC | NOESY |
|---|---|---|---|---|---|---|
| 1 C | 12.84 | | | 1a, 1b | 1'a, 1'b, 2 | |
| Ha | 1.25 | | 1'a, 1'b, 1b, 2 | 1 | 1', 2, 3 | |
| Hb | 1.19 | | 1'a, 1'b, 1a, 2 | 1 | 1', 2, 3 | |
| 1' C | 12.84 | | | 1'a, 1'b | 1a, 1b, 2 | |
| Ha | 1.25 | | 1'b, 1a, 1b, 2 | 1' | 1, 2, 3 | |
| Hb | 1.19 | | 1'a, 1a, 1b, 2 | 1' | 1, 2, 3 | |
| 2 C | 18.77 | | | | 1'a, 1'b, 1a, 1b, 4 | |
| H | 2.24 | 8.00(?), 4.70(?) | 1'a, 1'b, 1a, 1b | | 1, 1', 4 | 4 |
| 3 C | 167.87 | | | | 1'a, 1'b, 1a, 1b, 4 | |
| 4 C | 119.85 | 5.00(14) | | 4 | 2 | |
| H | 7.58 | | | 4 | 2, 3, 6, 13 | 2 |
| 5 C | 134.03 | 31.90(14) | | | 12 | |
| 6 C | 120.71 | | | | 4, 9 | |
| 7 C | 149.61 | | | | 10, 12 | |
| 8 N | -75.96 | | | | | |
| 9 C | 132.89 | | | 9 | | |
| H | 8.05 | 9.10(?) | 10 | 9 | 6, 10, 11 | |
| 10 C | 127.36 | | | 10 | 9, 12 | |
| H | 7.55 | 9.20(?), 2.30(?) | 9, 12 | 10 | 7, 12 | |
| 11 C | 121.60 | | | | 9, 12 | |
| 12 C | 124.85 | 2.40(14) | | 12 | 10 | |
| H | 7.44 | | 10 | 12 | 5, 7, 10, 11 | |
| 13 C | 122.68 | 274.90(14) | | | 4 | |
| 14 F | -61.64 | 274.90(13), 2.40(12), 31.90(5), 5.00(4) | | | | |
| 15 C | 118.45 | | | | 17 | |
| 16 C | 135.92 | | | 16 | 18 | |
| H | 8.70 | | | 16 | 18, 20 | |
| 17 C | 130.54 | | | 17 | 19 | |
| H | 7.82 | | | 17 | 15, 19 | |
| 18 C | 135.35 | | | 18 | 16 | |
| H | 7.86 | | | 18 | 16, 20 | |
| 19 C | 130.25 | | | 19 | 17 | |
| H | 7.84 | | | 19 | 17 | |
| 20 C | 136.79 | | | | 16, 18 | |
| 21 B | -0.69 | | | | | |
| 22 F | -150.63 | | | | | |

**4-Methoxydiphenyl ether-derived thianthrenium salt TT-19**

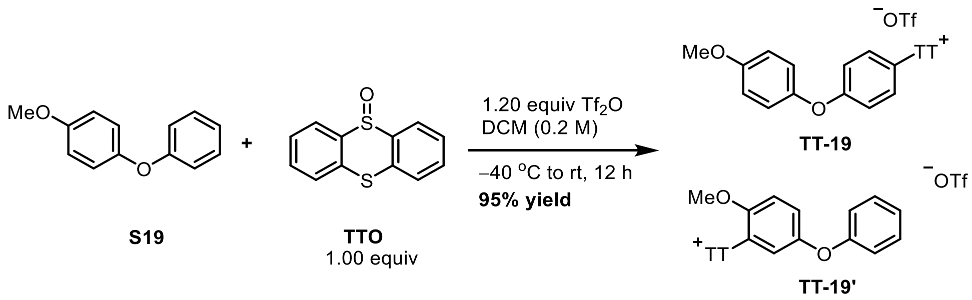


Under an ambient atmosphere, a 20 mL glass vial was charged with 4-methoxydiphenyl ether **S19** (400 mg, 2.00 mmol, 1.00 equiv), **TTO** (464 mg, 2.00 mmol, 1.00 equiv), and dry DCM (10 mL). Triflate anhydride (677 mg, 404 μL, 2.40 mmol, 1.20 equiv) was added slowly at –40 °C. The mixture was gradually warmed to room temperature and stirred overnight. 20 mL saturated aqueous $NaHCO_3$ solution was added slowly. The mixture was stirred for 10 min and extracted by DCM (2 × 20 mL). The organic layer was dried over $Na_2SO_4$, filtered, and the solvent was removed under reduced pressure. The resulting residue was purified by flash column chromatography on silica gel (DCM/MeOH = 50:1 to 30:1) to afford a mixture of **TT-19** and **TT-19'** (2.2:1) as a colorless solid in 95% yield (1.07 g).

**$R_f$** = 0.20 (DCM/MeOH = 30:1).

**NMR Spectroscopy of TT-19:**

**$^{1}$H NMR** (500 MHz, $CDCl_3$, 23 ºC, δ): 8.54 (dd, *J* = 7.9, 1.3 Hz, 2H), 7.86 – 7.71 (m, 6H), 7.22 (d, *J* = 9.1 Hz, 2H), 6.95 – 6.89 (m, 6H), 3.79 (s, 3H).

**$^{13}$C NMR** (125 MHz, $CDCl_3$, 23 ºC, δ): 163.2, 157.2, 147.4, 136.2, 134.9, 134.8, 130.5, 130.2, 121.8, 119.2, 119.0, 118.5, 115.3, 55.7.

**$^{19}$F NMR** (470 MHz, $CDCl_3$, 23 ºC, δ): –78.10.

**HRMS-ESIpos (m/z)** calc'd for $C_{25}H_{19}O_2S_2$ [M–OTf]$^+$, 415.0821; found, 415.0826; deviation: –1.2 ppm.

**2-Chloroanisole-derived thianthrenium salt TT-21**

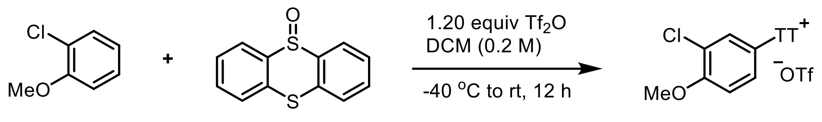


**S21** **TTO** 1.00 equiv **TT-21**, 95% yield

Under an ambient atmosphere, a 20 mL glass vial was charged with 2-chloroanisole **S21** (254 mg, 2.00 mmol, 1.00 equiv), **TTO** (464 mg, 2.00 mmol, 1.00 equiv), and dry DCM (10 mL). Triflate anhydride (677 mg, 404 μL, 2.40 mmol, 1.20 equiv) was added slowly at −40 °C. The mixture was gradually warmed to room temperature and stirred overnight. 20 mL saturated aqueous $NaHCO_3$ solution was added slowly. The mixture was stirred for 10 min and extracted by DCM (2 × 20 mL). The organic layer was dried over $Na_2SO_4$, filtered, and the solvent was removed under reduced pressure. The resulting residue was purified by flash column chromatography on silica gel (DCM/MeOH = 50:1 to 30:1) to afford **TT-21** as a colorless solid in 95% yield (960 mg).

**R*f*** = 0.20 (DCM/MeOH = 30:1).

**NMR Spectroscopy:**

**$^{1}$H NMR** (500 MHz, $CDCl_3$, 23 ºC, δ): 8.58 (d, *J* = 7.7 Hz, 2H), 7.94 – 7.69 (m, 6H), 7.35 (dd, *J* = 9.0, 2.7 Hz, 1H), 7.16 (d, *J* = 2.6 Hz, 1H), 7.05 (d, *J* = 9.0 Hz, 1H), 3.90 (s, 3H).

**$^{13}$C NMR** (125 MHz, $CDCl_3$, 23 ºC, δ): 159.0, 136.2, 135.0, 134.9, 130.3, 130.3, 129.4, 129.1, 125.0, 118.8, 114.4, 113.7, 56.8.

**$^{19}$F NMR** (470 MHz, $CDCl_3$, 23 ºC, δ): –78.11.

**HRMS-ESIpos (m/z)** calc'd for $C_{19}H_{14}ClOS_2$ [M–OTf]$^+$, 357.0169; found, 357.0173; deviation: –1.1 ppm.

**Thianaphthene-derived thianthrenium salt TT-25**

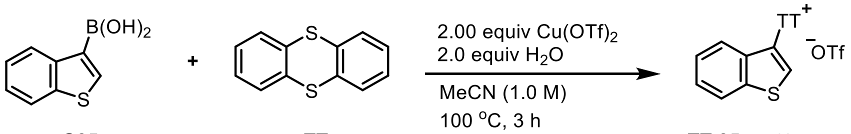


**S25** **TT** 1.50 equiv **TT-25**, 58% yield

Prepared according to a reported reaction condition[3]: under an ambient atmosphere, a 8 mL glass vial was charged with benzo[*b*]thiophen-3-ylboronic acid **S25** (178 mg, 1.00 mmol, 1.00 equiv), **TT** (324 mg, 1.50 mmol, 1.50 equiv), $Cu(OTf)_2$ (723 mg, 2.00 mmol, 2.00 equiv), $H_2O$ (36 μL, 2.0 mmol, 2.0 equiv), and dry MeCN (1.0 mL, c = 1.0 M). The mixture was heated to 100 °C for 3 h. After cooling to room temperature, the reaction mixture was added into ammonia solution (20 mL, 25%–28% solution in water), and the water phase was extracted with DCM (2 × 20 mL). The combined organic layers were dried over anhydrous $Na_2SO_4$, filtered and concentrated under vacuum. The resulting residue was purified by flash column chromatography on silica gel (DCM/MeOH = 50:1 to 30:1) to afford **TT-25** as a brown solid in 58% yield (290 mg).

$\mathbf{R}_f$ = 0.20 (DCM/MeOH = 30:1).

**NMR Spectroscopy:**

**$^1$H NMR** (500 MHz, $CDCl_3$, 23 °C, δ): 8.42 (d, *J* = 8.0 Hz, 2H), 7.96 – 7.83 (m, 5H), 7.76 (t, *J* = 7.7 Hz, 2H), 7.67 (t, *J* = 7.6 Hz, 2H), 7.47 (t, *J* = 5.5 Hz, 2H).

**$^{13}$C NMR** (125 MHz, $CDCl_3$, 23 °C, δ): 140.4, 138.2, 135.9, 134.6, 133.7, 133.3, 130.5, 130.3, 126.8, 126.9, 123.8, 121.5, 118.6, 108.1.

**$^{19}$F NMR** (470 MHz, $CDCl_3$, 23 °C, δ): –78.03.

**HRMS-ESIpos (m/z)** calc'd for $C_{20}H_{13}S_3$ $[M–OTf]^+$, 349.0174; found, 349.0173; deviation: +0.2 ppm.

**Pyrazol-derived thianthrenium salt TT-26**

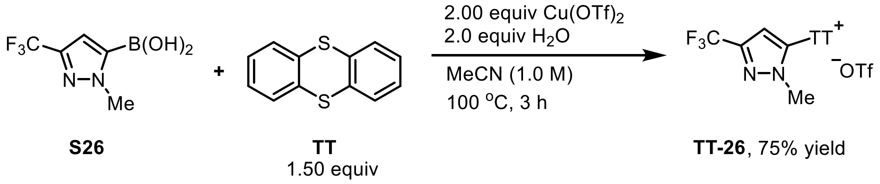


Prepared according to a reported reaction condition[3]: under an ambient atmosphere, a 8 mL glass vial was charged with (1-methyl-3-(trifluoromethyl)-1*H*-pyrazol-5-yl)boronic acid **S26** (194 mg, 1.00 mmol, 1.00 equiv), **TT** (324 mg, 1.50 mmol, 1.50 equiv), $Cu(OTf)_2$ (723 mg, 2.00 mmol, 2.00 equiv), $H_2O$ (36 μL, 2.0 mmol, 2.0 equiv), and dry MeCN (1.0 mL, c = 1.0 M). The mixture was heated to 100 °C for 3 h. After cooling to room temperature, the reaction mixture was added into ammonia solution (20 mL, 25%–28% solution in water), and the water phase was extracted with DCM (2 × 20 mL). The combined organic layers were dried over anhydrous $Na_2SO_4$, filtered and concentrated under vacuum. The resulting residue was purified by flash column chromatography on silica gel (DCM/MeOH = 50:1 to 30:1) to afford **TT-26** as a colorless solid in 75% yield (386 mg).

$\mathbf{R}_f$ = 0.21 (DCM/MeOH = 30:1).

**NMR Spectroscopy:**

**$^1$H NMR** (500 MHz, $CDCl_3$, 23 °C, δ): 8.75 (dd, *J* = 7.9, 1.4 Hz, 2H), 7.98 – 7.75 (m, 6H), 6.46 (d, *J* = 0.6

Hz, 1H), 4.27 (s, 3H).

**$^{13}$C NMR** (125 MHz, $CDCl_3$, 23 ºC, δ): 142.3 (q, *J* = 40.1 Hz), 136.6, 135.6, 135.1, 130.7, 130.6, 122.4, 119.6 (q, *J* = 269.8 Hz), 116.6, 111.2 (q, *J* = 2.2 Hz), 39.9.

**$^{19}$F NMR** (470 MHz, $CDCl_3$, 23 ºC, δ): –62.48 (s, 3F), –78.36 (s, 3F).

**HRMS-ESIpos (m/z)** calc'd for $C_{17}H_{12}F_3N_2S_2$ $[M–OTf]^+$, 365.0389; found, 365.0388; deviation: +0.2 ppm.

**Isoquinoline-derived thianthrenium salt TT-27**

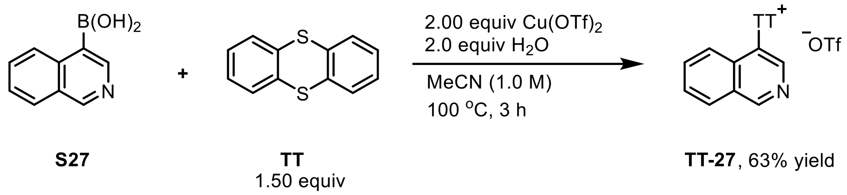


Prepared according to a reported reaction condition[3]: under an ambient atmosphere, a 8 mL glass vial was charged with isoquinolin-4-ylboronic acid **S27** (173 mg, 1.00 mmol, 1.00 equiv), **TT** (324 mg, 1.50 mmol, 1.50 equiv), $Cu(OTf)_2$ (723 mg, 2.00 mmol, 2.00 equiv), $H_2O$ (36 μL, 2.0 mmol, 2.0 equiv), and dry MeCN (1.0 mL, c = 1.0 M). The mixture was heated to 100 ºC for 3 h. After cooling to room temperature, the reaction mixture was added into ammonia solution (20 mL, 25%–28% solution in water), and the water phase was extracted with DCM (2 × 20 mL). The combined organic layers were dried over anhydrous $Na_2SO_4$, filtered and concentrated under vacuum. The resulting residue was purified by flash column chromatography on silica gel (DCM/MeOH = 50:1 to 30:1) to afford **TT-27** as a brown solid in 63% yield (311 mg).

**R*f*** = 0.15 (DCM/MeOH = 30:1).

**NMR Spectroscopy:**

**$^{1}$H NMR** (500 MHz, $CDCl_3$, 23 ºC, δ): 9.07 (dd, *J* = 4.2, 1.5 Hz, 1H), 8.66 – 8.61 (m, 2H), 8.35 (d, *J* = 8.4 Hz, 1H), 8.17 (d, *J* = 8.1 Hz, 1H), 7.89 – 7.84 (m, 2H), 7.80 – 7.75 (m, 4H), 7.67 – 7.59 (m, 2H), 7.23 (d, *J* = 7.7 Hz, 1H).

**$^{13}$C NMR** (125 MHz, $CDCl_3$, 23 ºC, δ): 151.5, 144.3, 137.8, 137.2, 135.9, 134.6, 134.4, 131.2, 130.6, 130.0, 129.8, 126.5, 123.6, 118.9, 117.6.

**$^{19}$F NMR** (470 MHz, $CDCl_3$, 23 ºC, δ): –77.97.

**HRMS-ESIpos (m/z)** calc'd for $C_{21}H_{14}NS_2$ $[M–OTf]^+$, 344.0562; found, 344.0564; deviation: –0.6 ppm.

**Quinoline-derived thianthrenium salt TT-28**

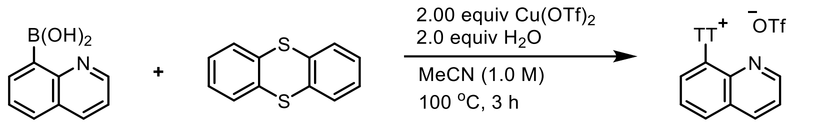


**S28** **TT** 1.50 equiv **TT-28**, 64% yield

Prepared according to a reported reaction condition[3]: under an ambient atmosphere, a 8 mL glass vial was charged with quinolin-8-ylboronic acid **S28** (173 mg, 1.00 mmol, 1.00 equiv), **TT** (324 mg, 1.50 mmol, 1.50 equiv), $Cu(OTf)_2$ (723 mg, 2.00 mmol, 2.00 equiv), $H_2O$ (36 μL, 2.0 mmol, 2.0 equiv), and dry MeCN (1.0 mL, c = 1.0 M). The mixture was heated to 100 °C for 3 h. After cooling to room temperature, the reaction mixture was added into ammonia solution (20 mL, 25%–28% solution in water), and the water phase was extracted with DCM (2 × 20 mL). The combined organic layers were dried over anhydrous $Na_2SO_4$, filtered and concentrated under vacuum. The resulting residue was purified by flash column chromatography on silica gel (DCM/MeOH = 50:1 to 30:1) to afford **TT-28** as a light yellow solid in 64% yield (315 mg).

**R*f*** = 0.15 (DCM/MeOH = 30:1).

**NMR Spectroscopy:**

**$^1$H NMR** (500 MHz, $CDCl_3$, 23 °C, δ): 9.39 (br, 1H), 8.91 (d, *J* = 8.5 Hz, 1H), 8.81 (dd, *J* = 7.5, 1.8 Hz, 2H), 8.23 – 8.10 (m, 3H), 7.89 – 7.82 (m, 3H), 7.82 – 7.73 (m, 4H).

**$^{13}$C NMR** (125 MHz, $CDCl_3$, 23 °C, δ): 157.9, 143.9, 137.4, 135.5, 134.9, 134.7, 132.2, 130.6, 130.4, 130.0, 129.4, 122.3, 116.6.

**$^{19}$F NMR** (470 MHz, $CDCl_3$, 23 °C, δ): –78.14.

**HRMS-ESIpos (m/z)** calc'd for $C_{21}H_{14}NS_2$ $[M–OTf]^+$, 344.0562; found, 344.0564; deviation: –0.6 ppm.

**Pyridine-derived thianthrenium salt TT-29**

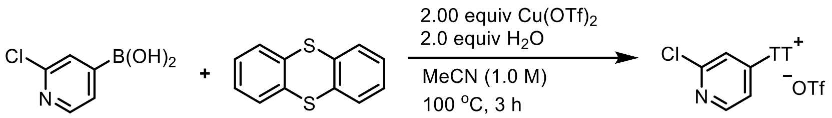


**S29** **TT** 1.50 equiv **TT-29**, 78% yield

Prepared according to a reported reaction condition[3]: under an ambient atmosphere, a 8 mL glass vial was charged with (2-chloropyridin-4-yl)boronic acid **S29** (157 mg, 1.00 mmol, 1.00 equiv), **TT** (324 mg, 1.50 mmol, 1.50 equiv), $Cu(OTf)_2$ (723 mg, 2.00 mmol, 2.00 equiv), $H_2O$ (36 μL, 2.0 mmol, 2.0 equiv), and dry MeCN (1.0 mL, c = 1.0 M). The mixture was heated to 100 °C for 3 h. After cooling to room temperature, the reaction mixture was added into ammonia solution (20 mL, 25%–28% solution in water), and the water phase was extracted with DCM (2 × 20 mL). The combined organic layers were dried over anhydrous $Na_2SO_4$, filtered and concentrated under vacuum. The resulting residue was purified by flash column chromatography

on silica gel (DCM/MeOH = 50:1 to 30:1) to afford **TT-29** as a colorless solid in 78% yield (372 mg).

**R*f*** = 0.15 (DCM/MeOH = 30:1).

**NMR Spectroscopy:**

**$^{1}$H NMR** (500 MHz, $CDCl_3$, 23 ºC, δ): 8.85 – 8.70 (m, 2H), 8.47 (d, *J* = 5.5 Hz, 1H), 7.93 – 7.84 (m, 6H), 7.05 (dd, *J* = 5.4, 1.9 Hz, 1H), 6.96 (d, *J* = 1.9 Hz, 1H).

**$^{13}$C NMR** (125 MHz, $CDCl_3$, 23 ºC, δ): 152.9, 151.1, 138.1, 136.9, 136.5, 135.6, 130.6, 122.2, 120.7, 117.6.

**$^{19}$F NMR** (470 MHz, $CDCl_3$, 23 ºC, δ): –78.35.

**HRMS-ESIpos (m/z)** calc'd for $C_{19}H_{11}ClNS_2$ [M–OTf]$^+$, 328.0016; found, 328.0020; deviation: –1.3 ppm.

## Synthesis of copper complexes

### Synthesis of *rac*-BINAPCu(MeCN)$BF_4$

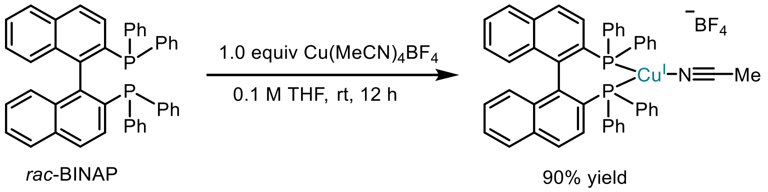


Prepared according to a reported reaction condition[6]: In a nitrogen filled glovebox, to an oven-dried 100 mL flask was added $Cu(MeCN)_4BF_4$ (629 mg, 2.00 mmol, 1.00 equiv), *rac*-BINAP (1.24 g, 2.00 mmol, 1.00 equiv), and 50 mL dry THF. The solution was allowed to stir at room temperature for 12 hours. Approximately 80 mL of dry hexanes was added to precipitate a white solid. There resulting solid was collected by vacuum filtration and dried under reduced pressure to give *rac*-BINAPCu(MeCN)$BF_4$ as a white solid in 90% yield (1.46 g).

**NMR Spectroscopy:**

**$^{1}$H NMR** (500 MHz, $CD_2Cl_2$, 23 ºC, δ): 7.94 – 7.83 (m, 4H), 7.61 (td, *J* = 10.0, 6.2 Hz, 10H), 7.43 – 7.34 (m, 2H), 7.27 (dt, *J* = 9.0, 4.7 Hz, 2H), 7.15 – 7.04 (m, 6H), 6.78 (q, *J* = 8.3 Hz, 4H), 6.63 (t, *J* = 7.6 Hz, 4H), 2.37 (s, 3H).

**$^{13}$C NMR** (125 MHz, $CDCl_3$, 23 ºC, δ): 139.0 (t, *J* = 8.7 Hz), 134.8 (t, *J* = 9.8 Hz), 133.3 (t, *J* = 3.8 Hz), 133.2, 133.0 (t, *J* = 8.9 Hz), 131.2, 130.9 (t, *J* = 19.6 Hz), 130.5 (t, *J* = 14.7 Hz), 129.6, 129.4 (t, *J* = 5.4 Hz), 128.9 (t, *J* = 3.8 Hz), 127.9, 127.9 (t, *J* = 17.2 Hz), 127.5 (t, *J* = 5.5 Hz), 127.0, 126.9, 126.5, 2.7.

**$^{19}$F NMR** (470 MHz, $CD_2Cl_2$, 23 ºC, δ): –152.88, –152.94

**$^{31}$P NMR** (203 MHz, $CD_2Cl_2$, 23 ºC, δ): 0.73.

**HRMS-ESIpos (m/z)** calc'd for $C_{46}H_{35}CuNP_2$ [M–$BF_4$]$^+$, 726.1535; found, 726.1540; deviation: –0.7 ppm.

**Synthesis of *rac*-BINAPCuN$_3$ (2)**

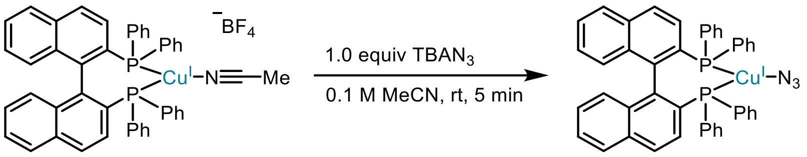


**2**, 95% yield

In a nitrogen filled glovebox, to an oven-dried 25 mL flask was added *rac*-BINAPCu(MeCN)$BF_4$ (407 mg, 0.500 mmol, 1.00 equiv), tetrabutylammonium azide (142 mg, 0.500 mmol, 1.00 equiv), and 20 mL dry MeCN. The solution was allowed to stir at room temperature for 5 min. The resulting solid was collected by vacuum filtration, washed with MeCN (2 × 10 mL), and dried under reduced pressure to give **2** as a bright yellow solid in 95% yield (345 mg).

**NMR Spectroscopy:**

**$^1$H NMR** (500 MHz, $CD_2Cl_2$, 23 ºC, δ): 7.94 (q, *J* = 5.9 Hz, 4H), 7.49 (dd, *J* = 11.8, 8.4 Hz, 4H), 7.33 – 7.23 (m, 6H), 7.21 (dt, *J* = 9.0, 4.6 Hz, 2H), 7.10 – 7.03 (m, 4H), 6.91 (m, 4H), 6.86 (d, *J* = 8.5 Hz, 2H), 6.71 (t, *J* = 7.4 Hz, 2H), 6.58 (t, *J* = 7.5 Hz, 4H).

**$^{13}$C NMR** (125 MHz, $CD_2Cl_2$, 23 ºC, δ): 138.8 (t, *J* = 8.3 Hz), 135.7 (t, *J* = 10.2 Hz), 133.7 (t, *J* = 3.8 Hz), 133.2, 130.3, 129.1, 129.0 (t, *J* = 3.8 Hz), 128.8 (t, *J* = 3.7 Hz), 128.3, 127.8, 127.8 (t, *J* = 3.8 Hz), 127.6 (t, *J* = 5.3 Hz), 126.5, 126.3.

**$^{31}$P NMR** (203 MHz, $CD_2Cl_2$, 23 ºC, δ): –2.02.

**Synthesis of *rac*-BINAPCu(N$_3$)$_2$ (3)**

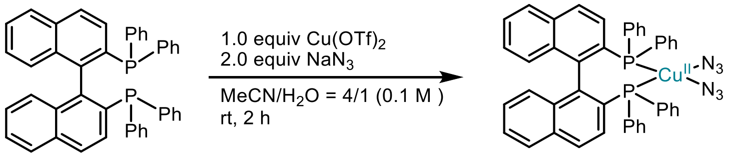


*rac*-BINAP **3**, 78% yield

Under an ambient atmosphere, a 50 mL flask was charged with Cu(OTf)$_2$ (723 mg, 2.00 mmol, 1.00 equiv), *rac*-BINAP (1.24 g, 2.00 mmol, 1.00 equiv), $NaN_3$ (260 mg, 4.00 mmol, 2.00 equiv), $H_2O$ (4 mL), and MeCN (16 mL). The mixture was stirred at room temperature for 2 h. The resulting solid was collected by vacuum filtration, washed with $H_2O$ (2 mL) and MeCN (2 mL), and dried under reduced pressure to give **3** as a tan solid in 78% yield (1.20 g).

**IR** (neat, cm$^{-1}$): 3051, 2066 ($N_3$), 2052 ($N_3$), 1584, 1501, 1481, 1312, 1435, 1312, 1275, 1092, 1026, 999, 868, 817, 773, 748, 739, 694, 673, 638.

**UV-Vis** (DCM, 1.0 mM, 25 ºC): $\lambda_{max}$ = 392 nm.

**Elemental Analysis**: calculated for $C_{44}H_{32}N_6P_2Cu$: C (%) 68.61, H (%) 4.19, N (%) 10.91: found

C (%) 68.29, H (%) 4.27, N (%) 10.72.

## Mechanistic studies

### UV-vis absorption spectroscopy studies

All UV-Vis spectrum measurements were recorded on a Shimadzu UV-Vis spectrophotometer UV-2600 with temperature controller using a quartz cuvette (10 × 10 mm, 3.5 mL) in acetonitrile as solvent.

UV-Vis spectra of the reaction mixture were measured with the following concentrations:

$Cu(MeCN)_4BF_4$ (0.6 mg, 2 μmol, c = 0.1 mM) in MeCN (20 mL);

*rac*-BINAP (1.2 mg, 2 μmol, c = 0.1 mM) in MeCN (20 mL);

$Cu(MeCN)_4BF_4$ (0.6 mg, 2 μmol, c = 0.1 mM) and *rac*-BINAP (1.2 mg, 2 μmol, c = 0.1 mM) in MeCN (20 mL);

*rac*-BINAPCu(MeCN)$BF_4$ (1.6 mg, 2 μmol, c = 0.1 mM) in MeCN (20 mL);

*rac*-BINAPCu$N_3$ (**2**) (1.5 mg, 2 μmol, c = 0.1 mM) in MeCN (20 mL);

*rac*-BINAPCu$(N_3)_2$ (**3**) (1.6 mg, 2 μmol, c = 0.1 mM) in MeCN (20 mL);

$Cu(MeCN)_4BF_4$ (0.6 mg, 2 μmol, c = 0.1 mM), *rac*-BINAP (1.2 mg, 2 μmol, c = 0.1 mM), and $NaN_3$ (2.6 mg, 40 μmol, c = 2.0 mM) in MeCN (20 mL);

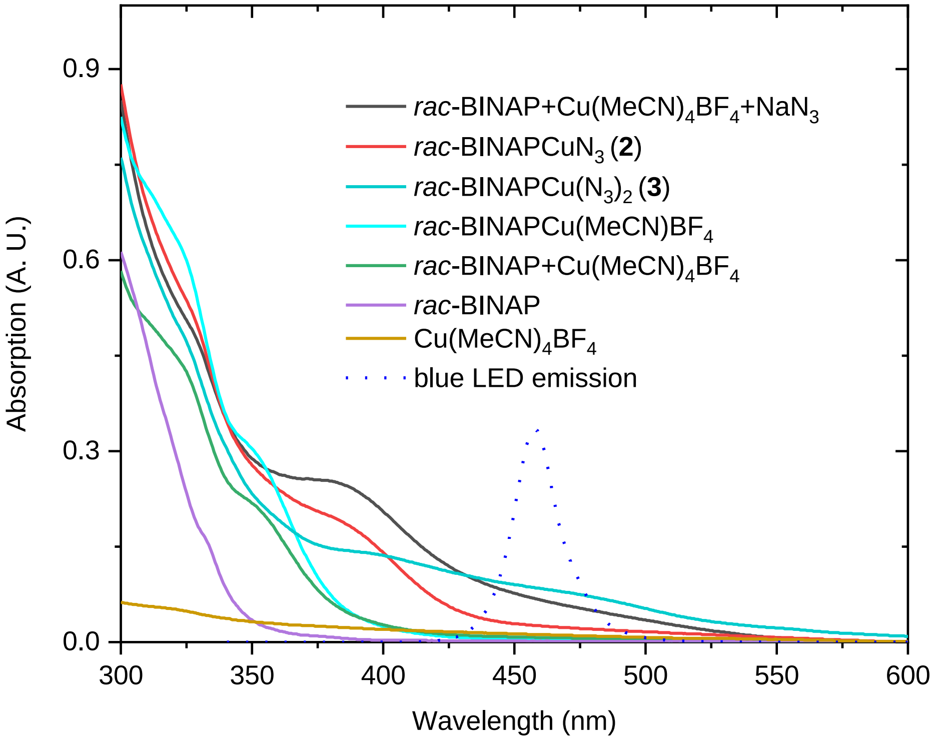


**Figure S4.** UV-Vis absorption spectra of the reaction mixture in MeCN.

Note: While the absorption of the mixture containing *rac*-BINAP, $Cu(MeCN)_4BF_4$, and $NaN_3$ exhibited higher overall absorption compared to complex **2**, the shape of the absorption curve was similar. We hypothesize that the presence of additional azide (20 equiv) in the mixture may coordinate with complex **2** and contribute to the

observed differences.

**UV–vis absorption spectra upon irradiation of *rac*-BINAPCu($N_3$)$_2$ (3)**

In a nitrogen-filled glovebox, a mixture of *rac*-BINAPCu($N_3$)$_2$ (**3**) (c = 0.05 mM) in 3.0 mL MeCN was transferred to a screw-top quartz cuvette. The quartz cuvette was sealed and taken out of the glovebox. The absorption spectra were recorded after the cuvette was irradiated by 456 nm LEDs at various times (0 min, 0.5 min, 1.0 min, 2.0 min, 3.5 min, 6.0 min, 10.0 min).

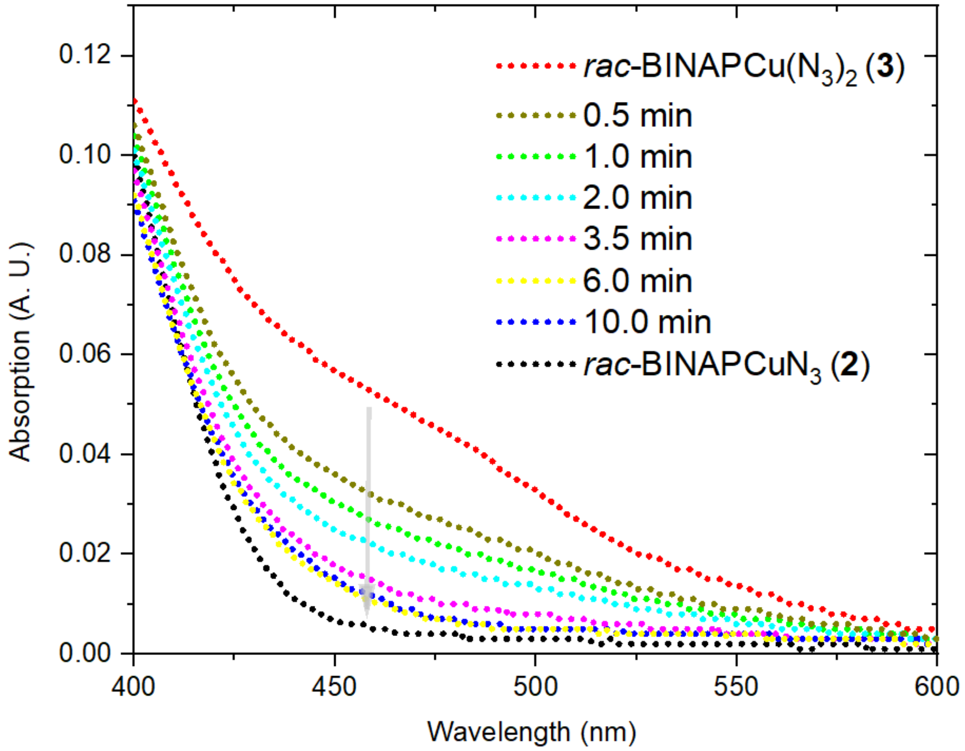


**Figure S5**. UV-Vis absorption spectra of the photolyzed solution of *rac*-BINAPCu($N_3$)$_2$ (**3**) in MeCN.

Complex **3** shows similar UV-Vis absorption to complex **2** as shown in Figure S4, and is also catalytically competent, suggesting it could potentially serve as a photocatalyst. However, based on the observations from the UV studies presented in Figure S5, it is unlikely that complex **3** functions as the photocatalyst owing to its rapid decomposition (may be to complex **2**) after 6 minutes under 456 nm LED irradiation. Additionally, both the oxidative quenching of excited complex **3** by arylthianthrenium salts and the reductive quenching by acrylonitrile are unlikely.

**Cyclic voltammograms**

Cyclic voltammograms were recorded using an Autolab PGSTAT204 potentiostat and a Pt working electrode, a Ag/AgCl reference electrode and a Pt wire auxiliary electrode. The voltammograms were recorded at room temperature in 0.1 M tetrabutylammonium tetrafluoroborate in DMSO (3 mL, c = 3.3 mM) containing *rac*-BINAPCu$N_3$ (**2**) (7.3 mg, 10 μmol) or *rac*-BINAPCu($N_3$)$_2$ (**3**) (7.7 mg, 10 μmol). The scan rate was 50 mV·s$^{-1}$.

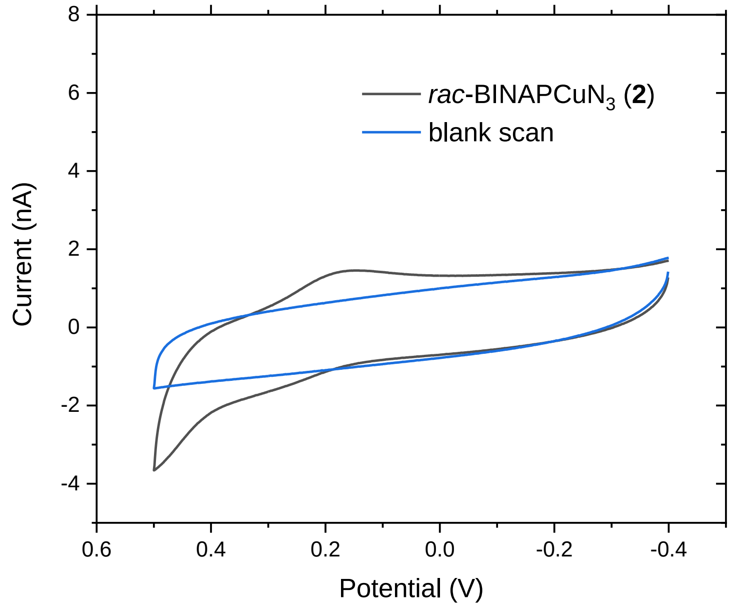


**Figure S6.** Cyclic voltammetry of *rac*-BINAPCu$N_3$ (**2**). $E_{pa}$ = 0.17 V, $E_{pc}$ = 0.29 V, $E_{1/2}$ = 0.23 V.

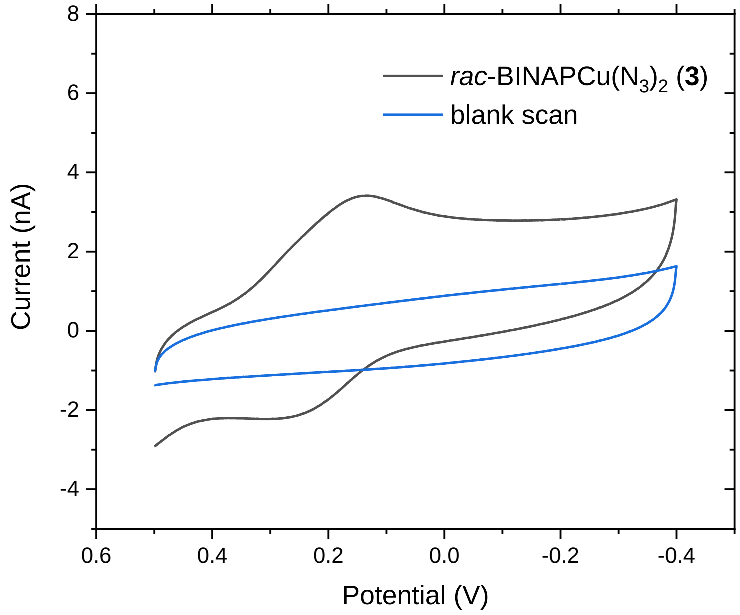


**Figure S7.** Cyclic voltammetry of *rac*-BINAPCu$(N_3)_2$ (**3**). $E_{pa}$ = 0.17 V, $E_{pc}$ = 0.29 V, $E_{1/2}$ = 0.23 V.

**Stern-volmer luminescence quenching studies**

Visible light luminescence intensities were recorded using an Edinburgh Instruments FS5 spectrofluorometer. All luminescence measurements were recorded using a screw-top quartz cuvette (Hellma fluorescence quartz cuvette, 10 × 10 mm, 3.5 mL). All solutions of *rac*-BINAPCu$N_3$ (**2**), **TT-3**, thianthrene, $NaN_3$, and methyl acrylate were prepared in DMSO in a nitrogen-filled glovebox. The solutions were transferred to the screw-top cuvette inside the glovebox, the cuvette was sealed, and then, brought out of the glovebox for visible light luminescence

measurements.

In a typical procedure, **TT-3** (24.5 mg, 50.0 μmol) was dissolved and diluted to a final volume of 10 mL (c = 5.0 mM) with a stock solution of *rac*-BINAPCuN3 (**2**) in DMSO (c = 0.10 mM). Serial dilution of this 5.0 mM **TT-3** solution was carried out by dilution of 6 mL of the 5.0 mM **TT-3** solution to 10 mL (3.0 mM) with the 0.10 mM stock solution of *rac*-BINAPCuN3 (**2**). All subsequent solutions were prepared by dilution of 6 mL of the preceding solution to a final volume of 10 mL. All solutions were excited at 400 nm and the emission was measured from 430 to 800 nm.

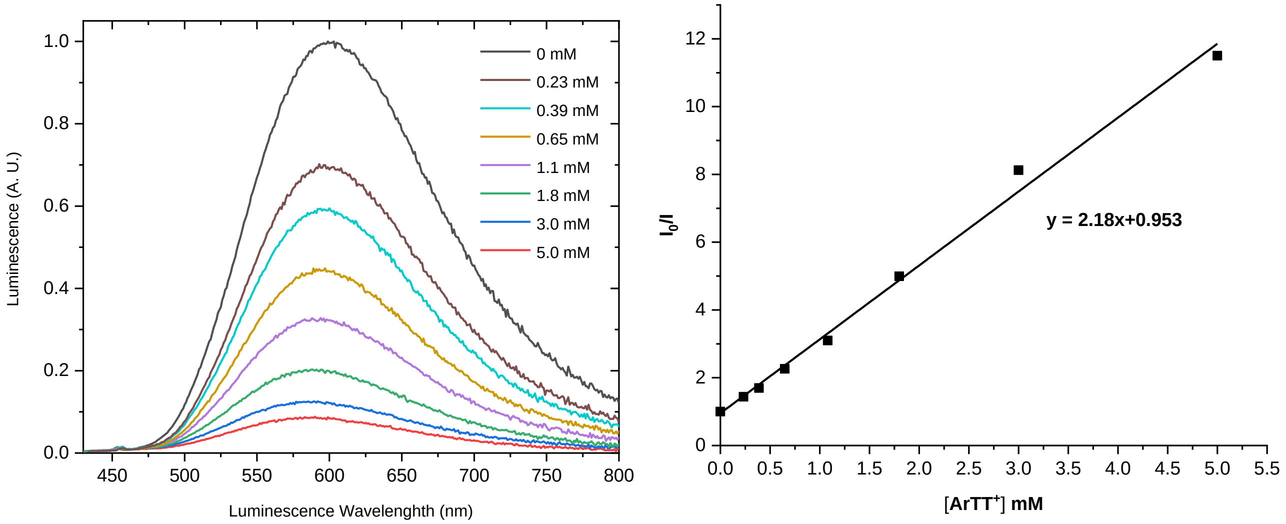


**Figure S8**. Emission spectra and Stern–Volmer plot for *rac*-BINAPCuN3 (**2**) luminescence quenching by **TT-3** ($K_{SV}$ = 20.8).

### Azidoarylation of alkenes catalyzed by copper complexes

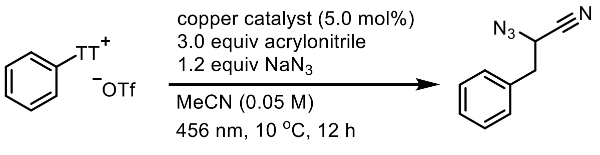


To a 4-mL borosilicate vial containing a Teflon-coated magnetic stirring bar were added **TT-1** (44.2 mg, 0.100 mmol, 1.00 equiv) and sodium azide (7.8 mg, 0.12 mmol, 1.2 equiv). The vial was transferred into a nitrogen-filled glovebox. *rac*-BINAPCu(MeCN)BF4 (4.1 mg, 5.0 μmol, 5.0 mol%), **2** (3.6 mg, 5.0 μmol, 5.0 mol%), or **3** (3.9 mg, 5.0 μmol, 5.0 mol%), dry MeCN (2 mL, c = 0.05 M), and acrylonitrile (16 mg, 20 μL, 0.30 mmol, 3.0 equiv) were added. The vial was sealed with a Teflon-lined screw cap and transferred to a cryocooler precooled at 10 °C where the reaction mixture was stirred for 5 min without irradiation and then irradiated by 456 nm LEDs for 2 h. Subsequently, the reaction mixture was concentrated to dryness under reduced pressure. The resulting residue was dissolved in $CDCl_3$ (0.5 mL); internal standard $CH_2Br_2$ (40 μL, 0.05 mmol, 1.25 mM in $CDCl_3$) was added, and the mixture was analyzed by $^1$H NMR spectroscopy. The results were shown as bellow:

| copper catalyst | conv./% | yield/% |
|---|---|---|
| *rac*-BINAPCu(MeCN)$BF_4$ | >99 | 75 |
| **2** | >99 | 71 |
| **3** | >99 | 65 |

**Trapping alkyl radical by *rac*-BINAPCu($N_3$)$_2$ (3)**

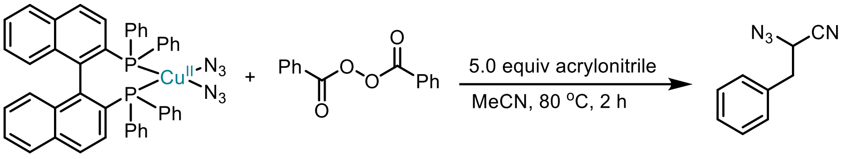


**3**, 0.050 mmol **BPO**, 3.0 equiv *rac*-**1**, 32%

To a 4-mL borosilicate vial containing a Teflon-coated magnetic stirring bar were added **3** (39 mg, 50 μmol, 1.0 equiv) and 75% purity BPO (51 mg, 0.15 mmol, 3.0 equiv). The vial was transferred into a nitrogen-filled glovebox and acrylonitrile (16 mg, 20 μL, 0.30 mmol, 3.0 equiv), dry MeCN (1.0 mL, c = 50 μM) was added. The vial was sealed with a Teflon-lined screw cap and heated at 80 °C for 2 h. The reaction mixture was concentrated under reduced pressure. The resulting residue was dissolved in EA (10 mL) and passed through a short silica gel column. Solvent was removed under reduced pressure. Internal standard 1,3,5-trimethoxybenzene (8.4 mg, 50 μmol, 1.0 equiv) and CDCl3 (500 μL) were added and the mixture was analyzed by $^1$H NMR spectroscopy. The yield was determined to be 32% by integrating the signal of the proton at 4.39 ppm.

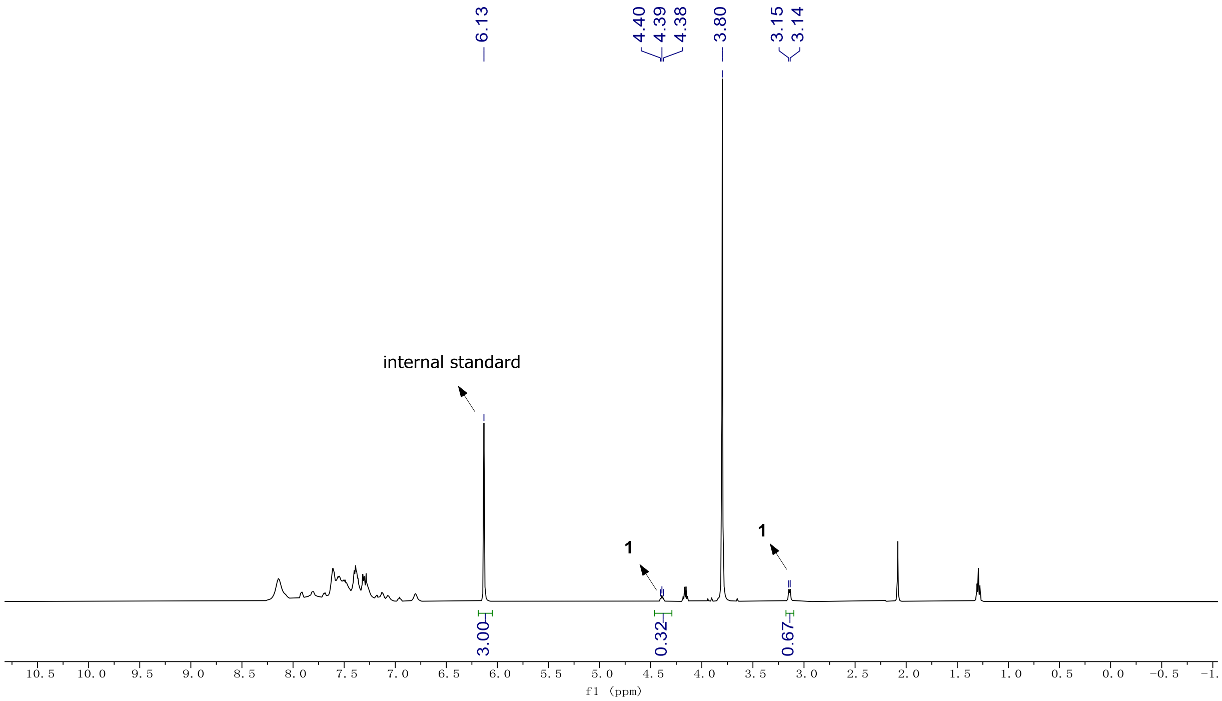


**Figure S9**. Reaction of copper complex **3**, benzoyl peroxide (BPO), and acrylonitrile in MeCN.

**Radical clock experiment**

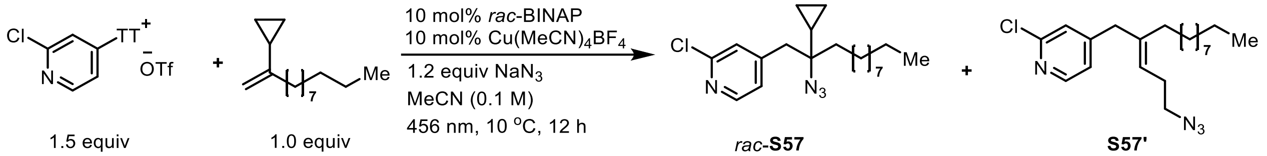


1.5 equiv **TT-29** 1.0 equiv *rac*-**S57** **S57'**

1 : 5.2

To a 20-mL borosilicate vial containing a Teflon-coated magnetic stirring bar were added **TT-29** (286 mg, 0.600 mmol, 1.50 equiv), *rac*-BINAP (25 mg, 40 μmol, 10 mol%), and sodium azide (31.2 mg, 0.48 mmol, 1.2 equiv). The vial was transferred into a nitrogen-filled glovebox. $Cu(MeCN)_4BF_4$ (13 mg, 40 μmol, 10 mol%), dry MeCN (4 mL, c = 0.1 M), and 2-cyclopropyl-1-dodecene (83 mg, 0.40 mmol, 1.0 equiv) were added. The vial was sealed with a Teflon-lined screw cap and transferred to a cryocooler precooled at 10 °C where the reaction mixture was stirred for 5 min without irradiation and then irradiated by 456 nm LEDs for 12 h. The reaction mixture was concentrated under reduced pressure. The resulting residue was dissolved in EA (20 mL) and passed through a short silica gel column. Solvent was removed under reduced pressure. Crude $^1$H NMR was measured to determine the ratio of *rac*-**S57** and S**57**’ (1:5.2) at this time slot. The resulting residue was purified by chromatography on silica gel (hexanes/EtOAc = 40:1 to 20:1), then preparative TLC to afford *rac*-**S57** (7.1 mg, 5% yield) and S**57**’ (34.6 mg, 24% yield).

*rac*-Pyridinylethylazide *rac*-**S57:**

**R*f*** = 0.21 (hexanes/EtOAc = 20:1).

**NMR Spectroscopy:**

**$^1$H NMR** (500 MHz, $CDCl_3$, 23 ºC, δ): 8.32 (d, *J* = 5.1 Hz, 1H), 7.26 (s, 1H), 7.15 (dd, *J* = 5.1, 1.5 Hz, 1H), 2.86 (d, *J* = 13.1 Hz, 1H), 2.78 (d, *J* = 13.1 Hz, 1H), 1.55 – 1.48 (m, 2H), 1.31 (d, *J* = 16.4 Hz, 16H), 0.91 (t, *J* = 6.9 Hz, 3H), 0.82 (tt, *J* = 8.3, 5.5 Hz, 1H), 0.51 – 0.30 (m, 4H).

**$^{13}$C NMR** (125 MHz, $CDCl_3$, 23 ºC, δ): 151.4, 149.1, 126.2, 124.6, 65.1, 43.6, 37.1, 34.1, 31.9, 29.9, 29.6, 29.3, 23.8, 22.7, 22.4, 18.1, 14.1, 14.1, 1.0, 0.9.

**HRMS-ESIpos (m/z)** calc’d for $C_{20}H_{31}N_4NaCl$ $[M+Na]^+$, 385.2129; found, 385.2127; deviation: +0.7 ppm.

Rearrangement product **S57’:**

**R*f*** = 0.20 (hexanes/EtOAc = 20:1).

**NMR Spectroscopy:**

**$^1$H NMR** (500 MHz, $CDCl_3$, 23 ºC, δ): 8.30 (d, *J* = 5.1 Hz, 1H), 7.17 (s, 1H), 7.06 (dd, *J* = 5.2, 1.5 Hz, 1H), 5.22 (t, *J* = 7.2 Hz, 1H), 3.39 – 3.30 (m, 4H), 2.39 (q, *J* = 7.0 Hz, 2H), 2.04 – 1.87 (m, 2H), 1.39 – 1.22 (m, 16H), 0.90 (t, *J* = 6.9 Hz, 3H).

**$^{13}$C NMR** (125 MHz, $CDCl_3$, 23 ºC, δ): 152.7, 151.7, 149.5, 140.0, 124.6, 124.4, 123.1, 51.2, 42.4, 34.1, 31.9, 30.0, 29.6, 29.6, 29.5, 29.3, 28.2, 27.8, 22.7, 22.4, 14.1, 14.0.

**HRMS-ESIpos (m/z)** calc'd for $C_{20}H_{32}N_4Cl$ $[M+H]^+$, 363.2310; found, 363.2308; deviation: +0.5 ppm.

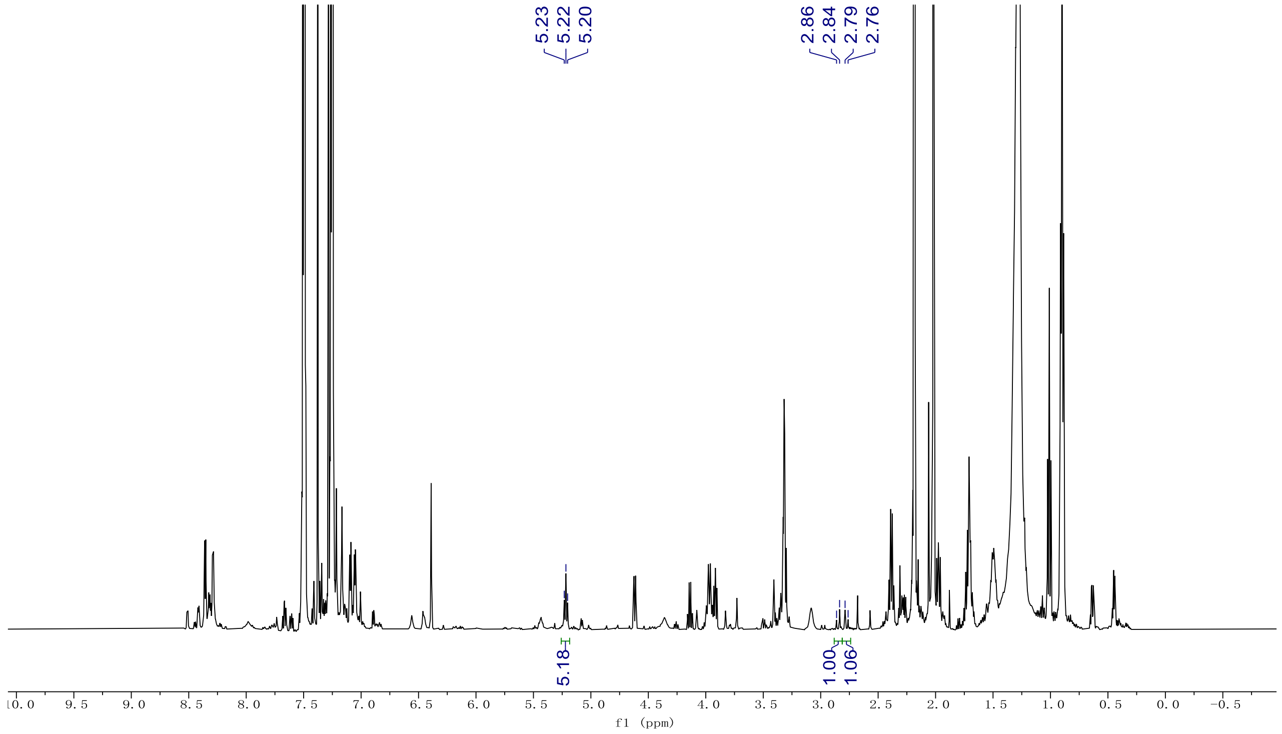


**Figure S10**. Crude $^{1}$H NMR of radical clock experiment based on the characteristic peak of *rac*-**S57** (2.85 ppm) and S**57**' (5.22 ppm).

# X-RAY CRYSTALLOGRAPHIC ANALYSIS

## X-Ray Chrystallographic Data of *rac*-BINAPCuN$_3$ (2) (CCDC 2247825)

### Experimental

Single crystal suitable for X-ray analysis was obtained as following: A solution of *rac*-BINAPCu(MeCN)BF$_4$ (1.0 equiv, 0.050 mmol, 41 mg) in acetonitrile (1.0 mL) was slowly layered over a solution of tetrabutylammonium azide (2.0 equiv, 0.10 mmol, 28 mg) in trifluoroethanol (1.0 ml) under nitrogen atmosphere. The reaction mixture was left at room temperature for 2 weeks without stirring until the single crystal formation was observed. The crystallographic data are summarized in the following tables:

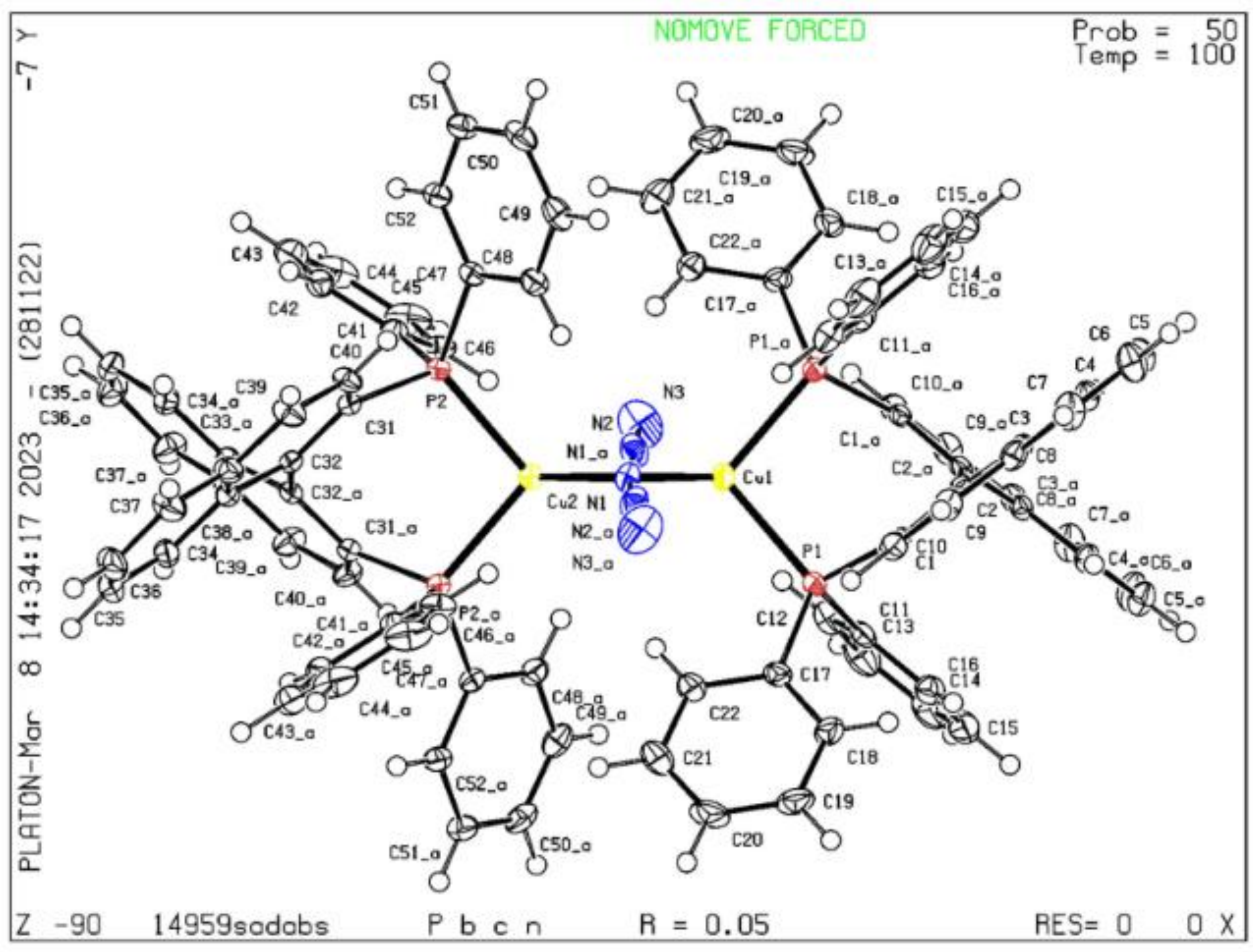


**Table S14. Crystal data and structure refinement.**

| | |
|---|---|
| Identification code | 14959 |
| Empirical formula | $C_{88} H_{64} Cu_2 N_6 P_4$ |
| Color | yellow |
| Formula weight | 1456.41 g mol$^{-1}$ |

| | | |
|---|---|---|
| Temperature | 100(2) K | |
| Wavelength | 0.71073 Å | |
| Crystal system | ORTHORHOMBIC | |
| Space group | Pbcn, (no. 60) | |
| Unit cell dimensions | a = 11.6660(6) Å | $\alpha$= 90°. |
| | b = 30.4160(15) Å | $\beta$= 90°. |
| | c = 19.4229(10) Å | $\gamma$ = 90°. |
| Volume | 6891.9(6) $Å^3$ | |
| Z | 4 | |
| Density (calculated) | 1.404 mg $m^{-3}$ | |
| Absorption coefficient | 0.764 $mm^{-1}$ | |
| F(000) | 3008 e | |
| Crystal size | 0.13 × 0.061 × 0.027 $mm^3$ | |
| $\theta$ range for data collection | 1.339 to 31.276°. | |
| Index ranges | $-17 \le h \le 17$, $-42 \le k \le 44$, $-28 \le l \le 28$ | |
| Reflections collected | 222210 | |
| Independent reflections | 11232 [$R_{int}$ = 0.0777] | |
| Reflections with $I>2\sigma(I)$ | 8100 | |
| Completeness to $\theta$ = 25.242° | 100.0% | |
| Absorption correction | Gaussian | |
| Max. and min. transmission | 0.98 and 0.93 | |
| Refinement method | Full-matrix least-squares on $F^2$ | |
| Data / restraints / parameters | 11232 / 0 / 452 | |
| Goodness-of-fit on $F^2$ | 1.049 | |
| Final R indices [$I>2\sigma(I)$] | $R_1$ = 0.0467 | $wR^2$ = 0.1043 |
| R indices (all data) | $R_1$ = 0.0760 | $wR^2$ = 0.1173 |
| Largest diff. peak and hole | 0.7 and -1.1 e $Å^{-3}$ | |

**Table S15. Bond lengths [Å] and angles [°].**

**Bond lengths lists:**

| | | | |
|---|---|---|---|
| Cu(1)-Cu(2) | 3.0592(5) | Cu(1)-P(1)#1 | 2.2563(5) |
| Cu(1)-P(1) | 2.2563(5) | Cu(1)-N(1)#1 | 2.0821(19) |
| Cu(1)-N(1) | 2.0820(19) | Cu(2)-P(2)#1 | 2.2681(5) |
| Cu(2)-P(2) | 2.2681(5) | Cu(2)-N(1) | 2.0978(19) |
| Cu(2)-N(1)#1 | 2.0978(19) | P(1)-C(1) | 1.8352(19) |
| P(1)-C(11) | 1.826(2) | P(1)-C(17) | 1.8232(19) |
| P(2)-C(31) | 1.8437(18) | P(2)-C(41) | 1.8196(19) |
| P(2)-C(47) | 1.8222(19) | N(1)-N(2) | 1.167(3) |
| N(2)-N(3) | 1.173(3) | C(1)-C(2) | 1.389(2) |
| C(1)-C(10) | 1.424(3) | C(2)-C(2)#1 | 1.494(3) |
| C(2)-C(3) | 1.434(3) | C(3)-C(4) | 1.416(3) |
| C(3)-C(8) | 1.422(3) | C(4)-C(5) | 1.371(3) |
| C(5)-C(6) | 1.407(3) | C(6)-C(7) | 1.362(3) |
| C(7)-C(8) | 1.413(3) | C(8)-C(9) | 1.415(3) |
| C(9)-C(10) | 1.360(3) | C(11)-C(12) | 1.394(3) |
| C(11)-C(16) | 1.393(3) | C(12)-C(13) | 1.389(3) |
| C(13)-C(14) | 1.376(4) | C(14)-C(15) | 1.381(4) |
| C(15)-C(16) | 1.391(3) | C(17)-C(18) | 1.388(3) |
| C(17)-C(22) | 1.388(3) | C(18)-C(19) | 1.388(3) |
| C(19)-C(20) | 1.375(4) | C(20)-C(21) | 1.380(4) |
| C(21)-C(22) | 1.393(3) | C(31)-C(32) | 1.387(2) |
| C(31)-C(40) | 1.420(3) | C(32)-C(32)#1 | 1.491(4) |
| C(32)-C(33) | 1.441(3) | C(33)-C(34) | 1.422(3) |
| C(33)-C(38) | 1.418(3) | C(34)-C(35) | 1.368(3) |
| C(35)-C(36) | 1.404(3) | C(36)-C(37) | 1.368(3) |
| C(37)-C(38) | 1.413(3) | C(38)-C(39) | 1.410(3) |
| C(39)-C(40) | 1.369(3) | C(41)-C(42) | 1.396(3) |
| C(41)-C(46) | 1.392(3) | C(42)-C(43) | 1.390(3) |
| C(43)-C(44) | 1.382(4) | C(44)-C(45) | 1.374(4) |
| C(45)-C(46) | 1.395(3) | C(47)-C(48) | 1.396(3) |
| C(47)-C(52) | 1.391(3) | C(48)-C(49) | 1.385(3) |
| C(49)-C(50) | 1.382(3) | C(50)-C(51) | 1.375(3) |
| C(51)-C(52) | 1.388(3) | | |

**Bond angles lists:**

| | | | |
|---|---|---|---|
| P(1)#1-Cu(1)-Cu(2) | 130.093(14) | P(1)-Cu(1)-Cu(2) | 130.093(13) |
| P(1)#1-Cu(1)-P(1) | 99.81(3) | N(1)#1-Cu(1)-Cu(2) | 43.16(5) |
| N(1)-Cu(1)-Cu(2) | 43.15(5) | N(1)#1-Cu(1)-P(1)#1 | 123.72(5) |
| N(1)-Cu(1)-P(1)#1 | 112.62(5) | N(1)#1-Cu(1)-P(1) | 112.61(5) |
| N(1)-Cu(1)-P(1) | 123.72(5) | N(1)-Cu(1)-N(1)#1 | 86.31(11) |
| P(2)#1-Cu(2)-Cu(1) | 130.531(13) | P(2)-Cu(2)-Cu(1) | 130.533(13) |
| P(2)#1-Cu(2)-P(2) | 98.94(3) | N(1)#1-Cu(2)-Cu(1) | 42.75(5) |
| N(1)-Cu(2)-Cu(1) | 42.75(5) | N(1)-Cu(2)-P(2)#1 | 116.13(5) |
| N(1)#1-Cu(2)-P(2) | 116.13(5) | N(1)#1-Cu(2)-P(2)#1 | 120.93(5) |
| N(1)-Cu(2)-P(2) | 120.93(5) | N(1)-Cu(2)-N(1)#1 | 85.51(11) |
| C(1)-P(1)-Cu(1) | 104.25(6) | C(11)-P(1)-Cu(1) | 120.34(7) |
| C(11)-P(1)-C(1) | 107.09(9) | C(17)-P(1)-Cu(1) | 116.76(7) |
| C(17)-P(1)-C(1) | 105.15(9) | C(17)-P(1)-C(11) | 102.09(9) |
| C(31)-P(2)-Cu(2) | 107.65(6) | C(41)-P(2)-Cu(2) | 117.34(6) |
| C(41)-P(2)-C(31) | 106.48(9) | C(41)-P(2)-C(47) | 103.87(8) |
| C(47)-P(2)-Cu(2) | 117.73(6) | C(47)-P(2)-C(31) | 102.29(8) |
| Cu(1)-N(1)-Cu(2) | 94.09(9) | N(2)-N(1)-Cu(1) | 125.05(15) |
| N(2)-N(1)-Cu(2) | 134.15(16) | N(1)-N(2)-N(3) | 179.7(3) |
| C(2)-C(1)-P(1) | 122.84(14) | C(2)-C(1)-C(10) | 118.96(17) |
| C(10)-C(1)-P(1) | 117.27(14) | C(1)-C(2)-C(2)#1 | 120.14(15) |
| C(1)-C(2)-C(3) | 120.22(16) | C(3)-C(2)-C(2)#1 | 119.54(14) |
| C(4)-C(3)-C(2) | 122.56(16) | C(4)-C(3)-C(8) | 118.12(17) |
| C(8)-C(3)-C(2) | 119.27(17) | C(5)-C(4)-C(3) | 120.98(18) |
| C(4)-C(5)-C(6) | 120.5(2) | C(7)-C(6)-C(5) | 120.0(2) |
| C(6)-C(7)-C(8) | 121.04(19) | C(7)-C(8)-C(3) | 119.36(18) |
| C(7)-C(8)-C(9) | 121.68(18) | C(9)-C(8)-C(3) | 118.90(18) |
| C(10)-C(9)-C(8) | 120.92(18) | C(9)-C(10)-C(1) | 121.48(18) |
| C(12)-C(11)-P(1) | 118.13(16) | C(16)-C(11)-P(1) | 123.00(16) |
| C(16)-C(11)-C(12) | 118.8(2) | C(13)-C(12)-C(11) | 120.2(2) |
| C(14)-C(13)-C(12) | 120.3(2) | C(13)-C(14)-C(15) | 120.2(2) |
| C(14)-C(15)-C(16) | 119.8(2) | C(15)-C(16)-C(11) | 120.6(2) |
| C(18)-C(17)-P(1) | 124.87(16) | C(22)-C(17)-P(1) | 115.64(16) |
| C(22)-C(17)-C(18) | 119.38(18) | C(19)-C(18)-C(17) | 120.5(2) |
| C(20)-C(19)-C(18) | 119.6(2) | C(19)-C(20)-C(21) | 120.7(2) |
| C(20)-C(21)-C(22) | 119.8(2) | C(17)-C(22)-C(21) | 120.0(2) |

| | | | |
|---|---|---|---|
| C(32)-C(31)-P(2) | 121.94(14) | C(32)-C(31)-C(40) | 119.04(17) |
| C(40)-C(31)-P(2) | 118.43(14) | C(31)-C(32)-C(32)#1 | 122.05(15) |
| C(31)-C(32)-C(33) | 120.06(17) | C(33)-C(32)-C(32)#1 | 117.89(14) |
| C(34)-C(33)-C(32) | 122.28(17) | C(38)-C(33)-C(32) | 119.54(17) |
| C(38)-C(33)-C(34) | 118.12(18) | C(35)-C(34)-C(33) | 120.67(19) |
| C(34)-C(35)-C(36) | 120.93(19) | C(37)-C(36)-C(35) | 119.8(2) |
| C(36)-C(37)-C(38) | 120.8(2) | C(37)-C(38)-C(33) | 119.66(18) |
| C(39)-C(38)-C(33) | 118.82(18) | C(39)-C(38)-C(37) | 121.51(19) |
| C(40)-C(39)-C(38) | 120.95(19) | C(39)-C(40)-C(31) | 121.51(18) |
| C(42)-C(41)-P(2) | 123.83(15) | C(46)-C(41)-P(2) | 117.09(15) |
| C(46)-C(41)-C(42) | 119.06(18) | C(43)-C(42)-C(41) | 120.6(2) |
| C(44)-C(43)-C(42) | 119.7(2) | C(45)-C(44)-C(43) | 120.4(2) |
| C(44)-C(45)-C(46) | 120.3(2) | C(41)-C(46)-C(45) | 119.9(2) |
| C(48)-C(47)-P(2) | 117.28(14) | C(52)-C(47)-P(2) | 123.50(14) |
| C(52)-C(47)-C(48) | 119.18(17) | C(49)-C(48)-C(47) | 119.92(19) |
| C(50)-C(49)-C(48) | 120.25(19) | C(51)-C(50)-C(49) | 120.23(19) |
| C(50)-C(51)-C(52) | 120.0(2) | C(51)-C(52)-C(47) | 120.27(19) |

Symmetry transformations used to generate equivalent atoms: #1 -x+1,y,-z+1/2

## SPECTROSCOPIC DATA

**$^{1}$H NMR of *rac*-nefiracetam-derived phenylalanine analogue 4**

CDCl$_3$, 23 ºC

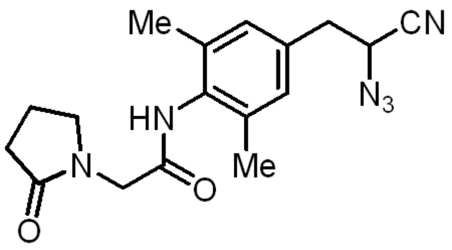

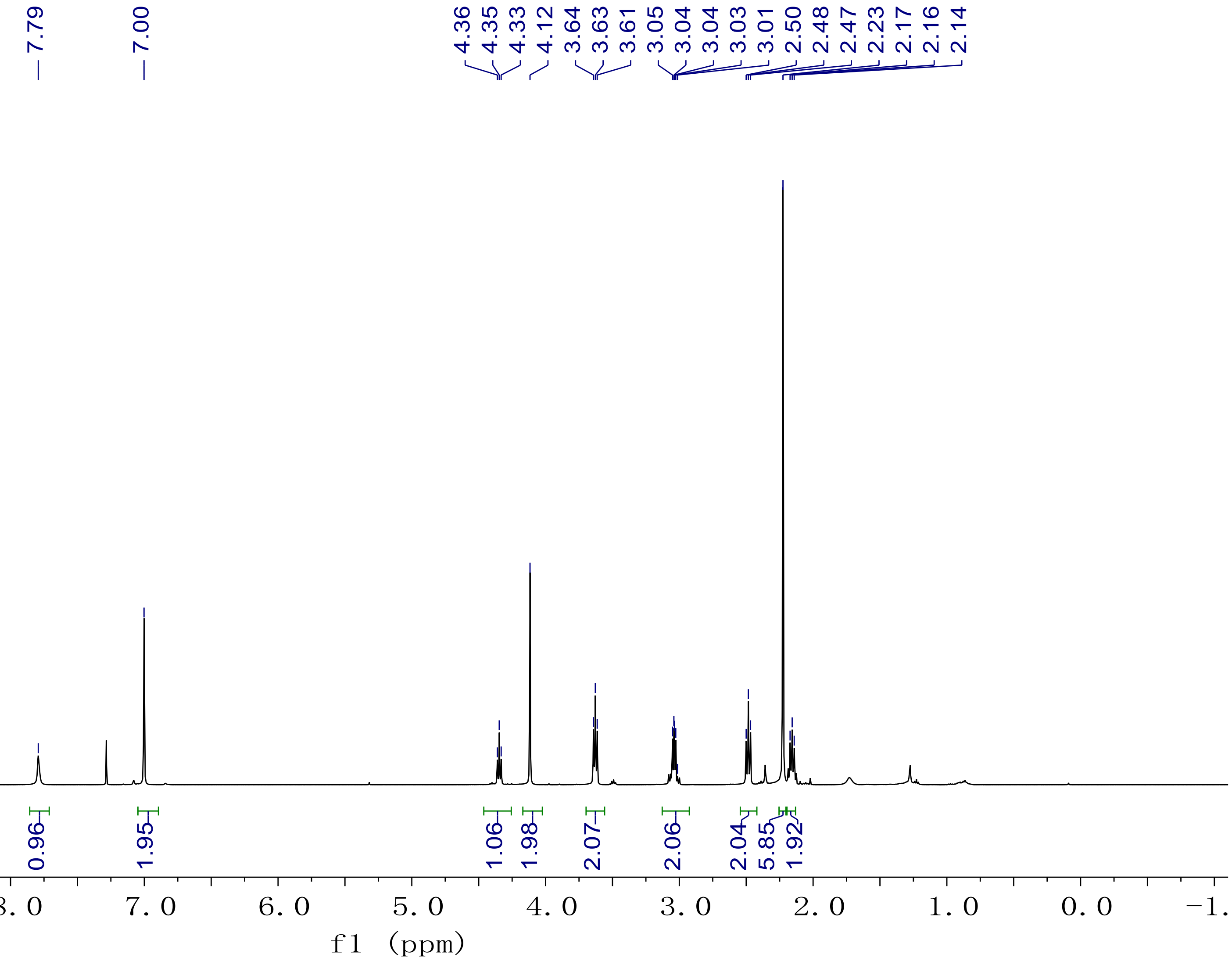

**$^{13}$C NMR of *rac*-nefiracetam-derived phenylalanine analogue 4**

$CDCl_3$, 23 ºC

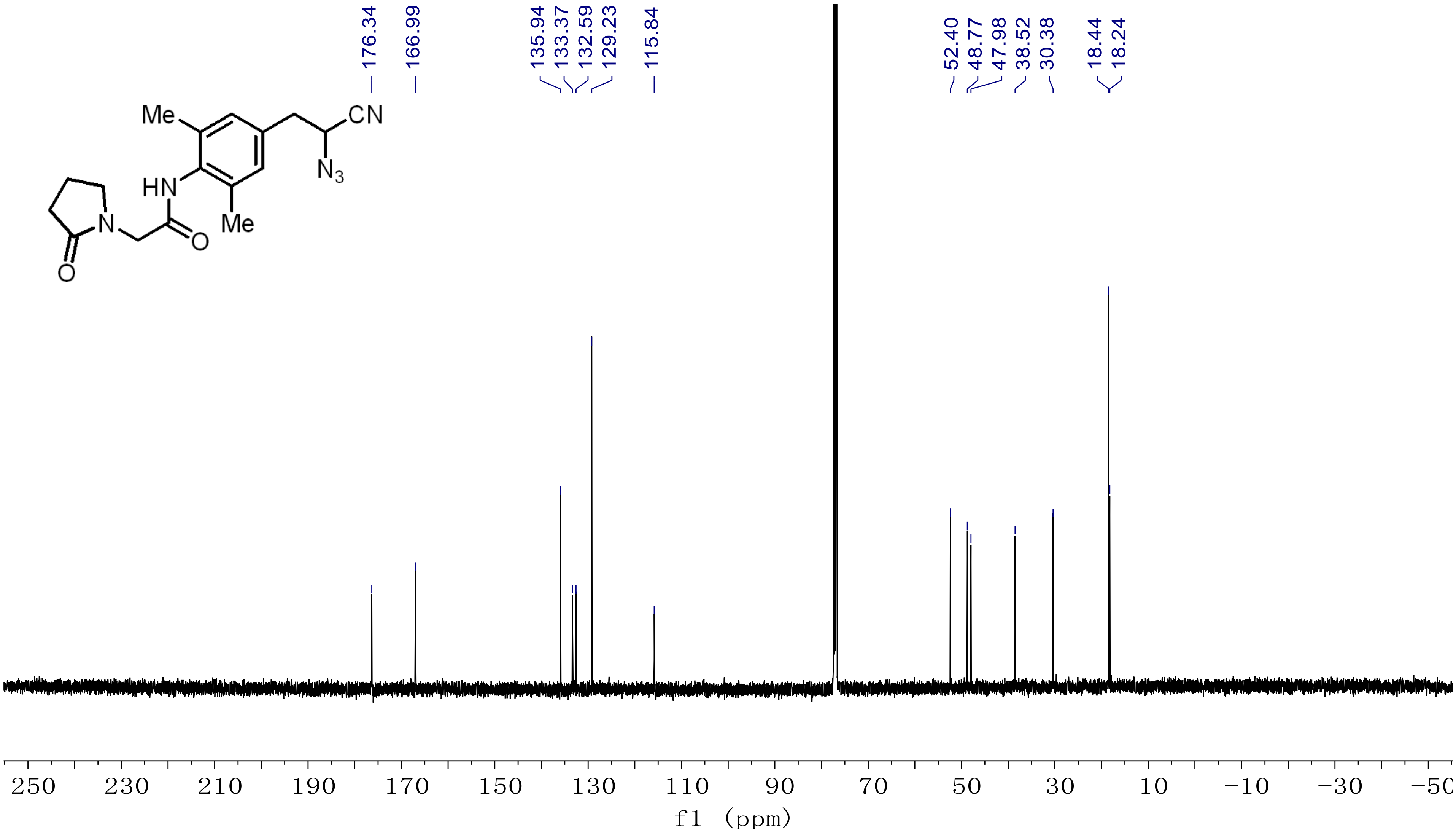

**$^1$H NMR of *rac*-nimesulide-derived phenylalanine analogue 5**

CDCl$_3$, 23 ºC

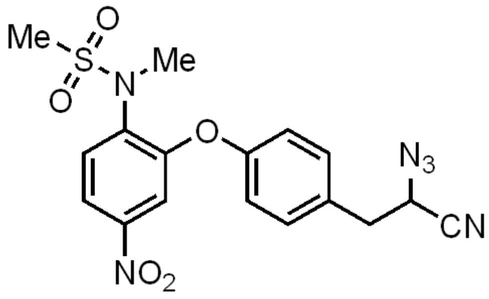


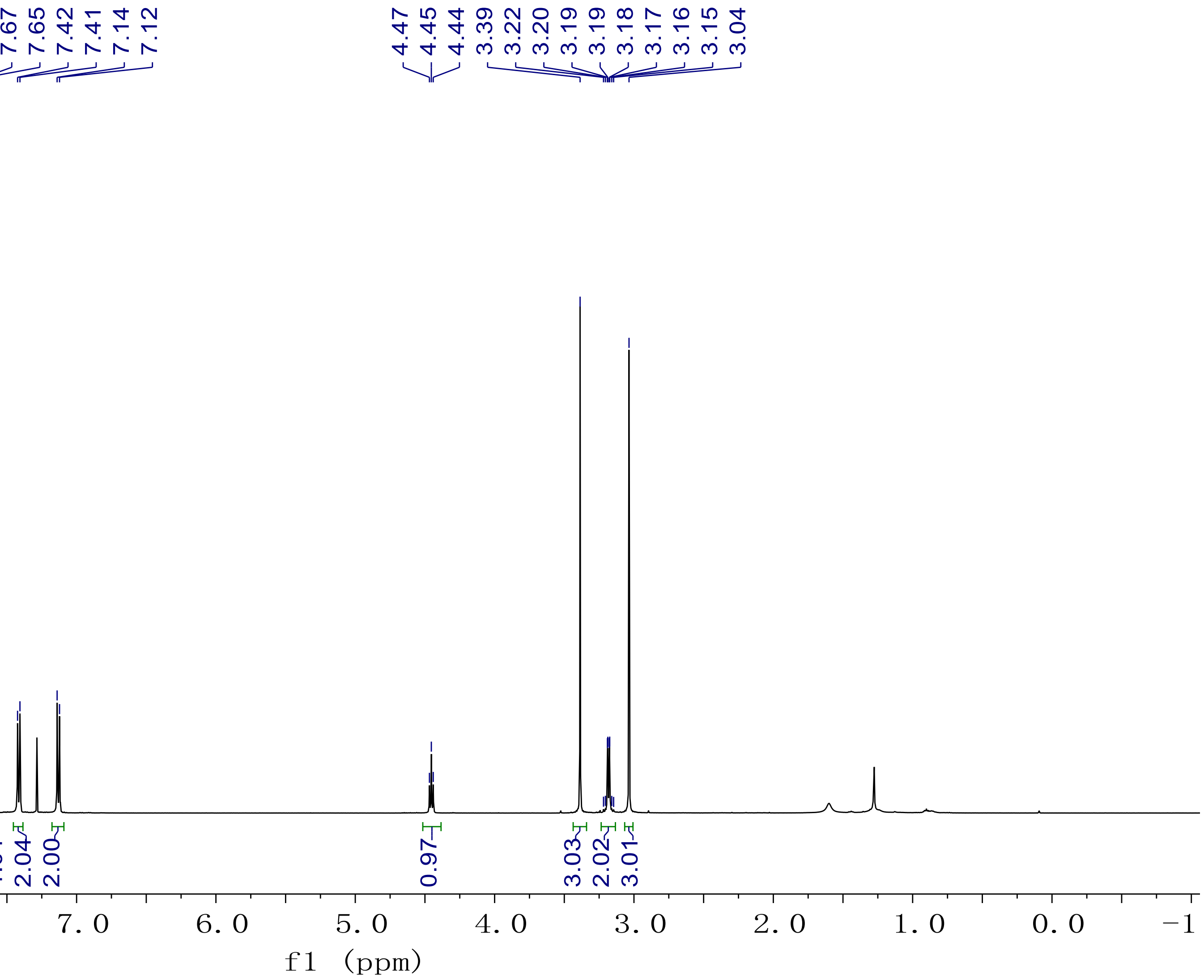

**$^{13}$C NMR of *rac*-nimesulide-derived phenylalanine analogue 5**

$CDCl_3$, 23 ºC

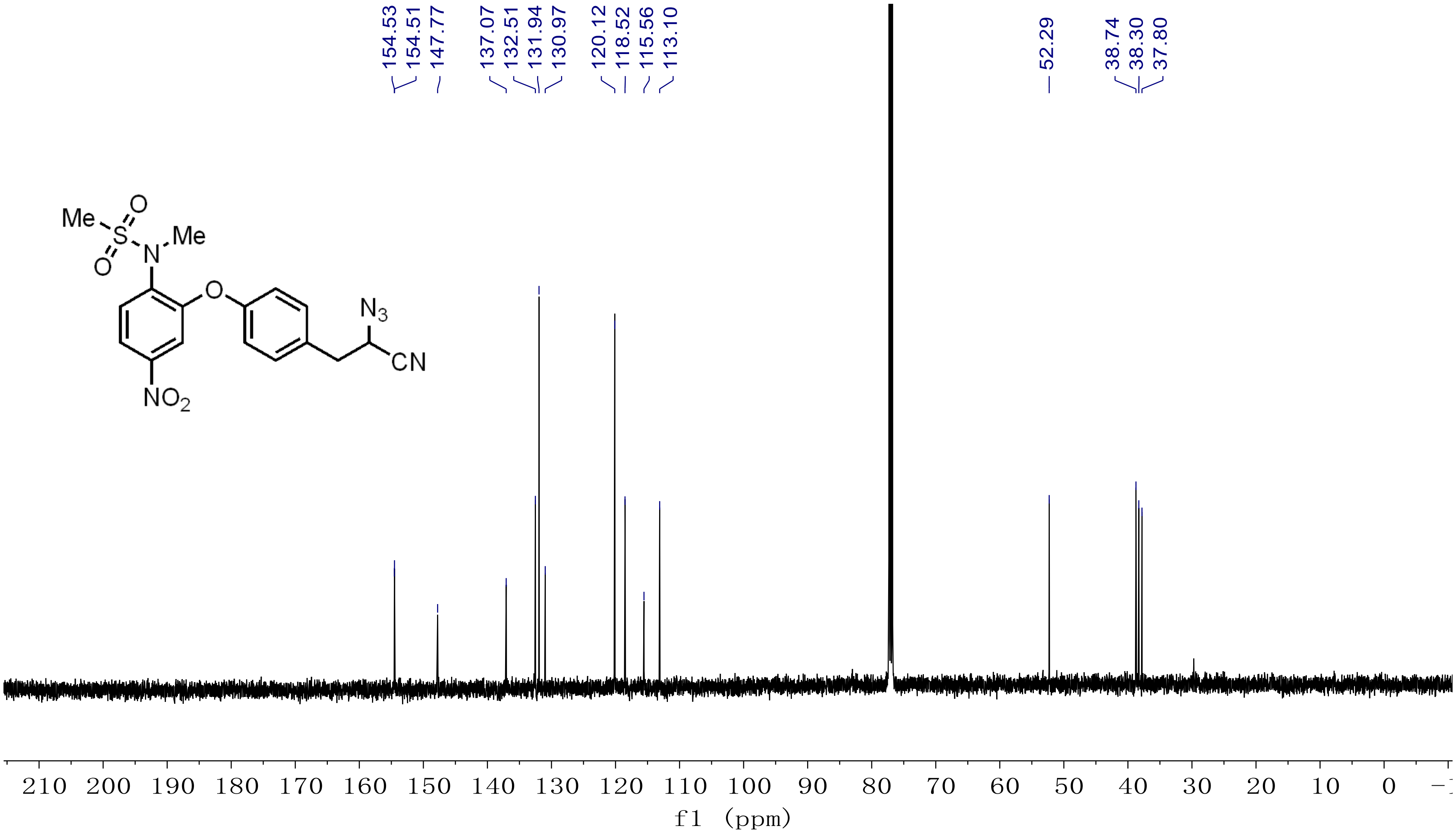

**$^{1}$H NMR of *rac*-meclofenamic acid-derived phenylalanine analogue 6**

$CDCl_3$, 23 ºC

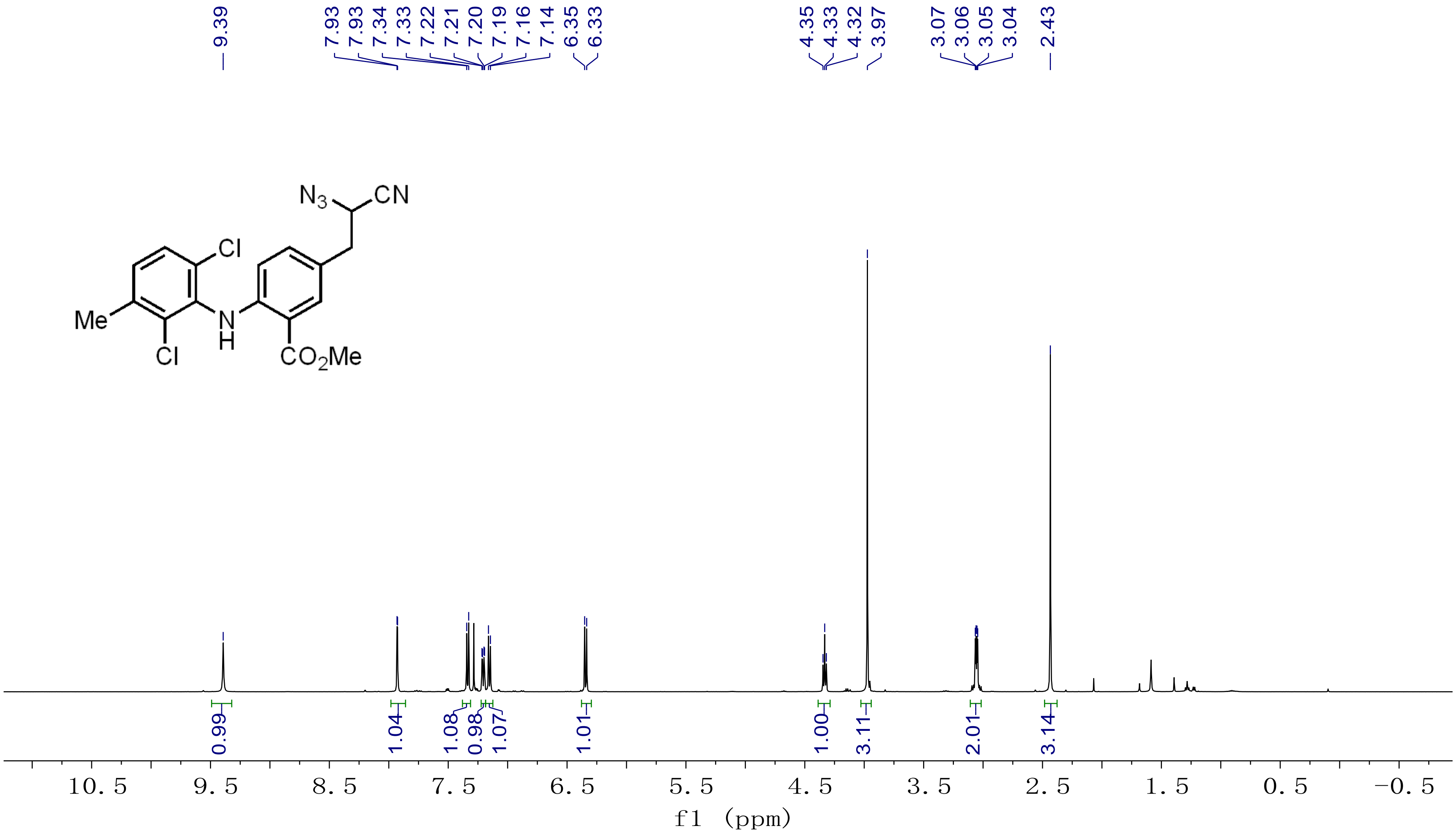

**$^{13}$C NMR of *rac*-meclofenamic acid-derived phenylalanine analogue 6**

CDCl3, 23 ºC

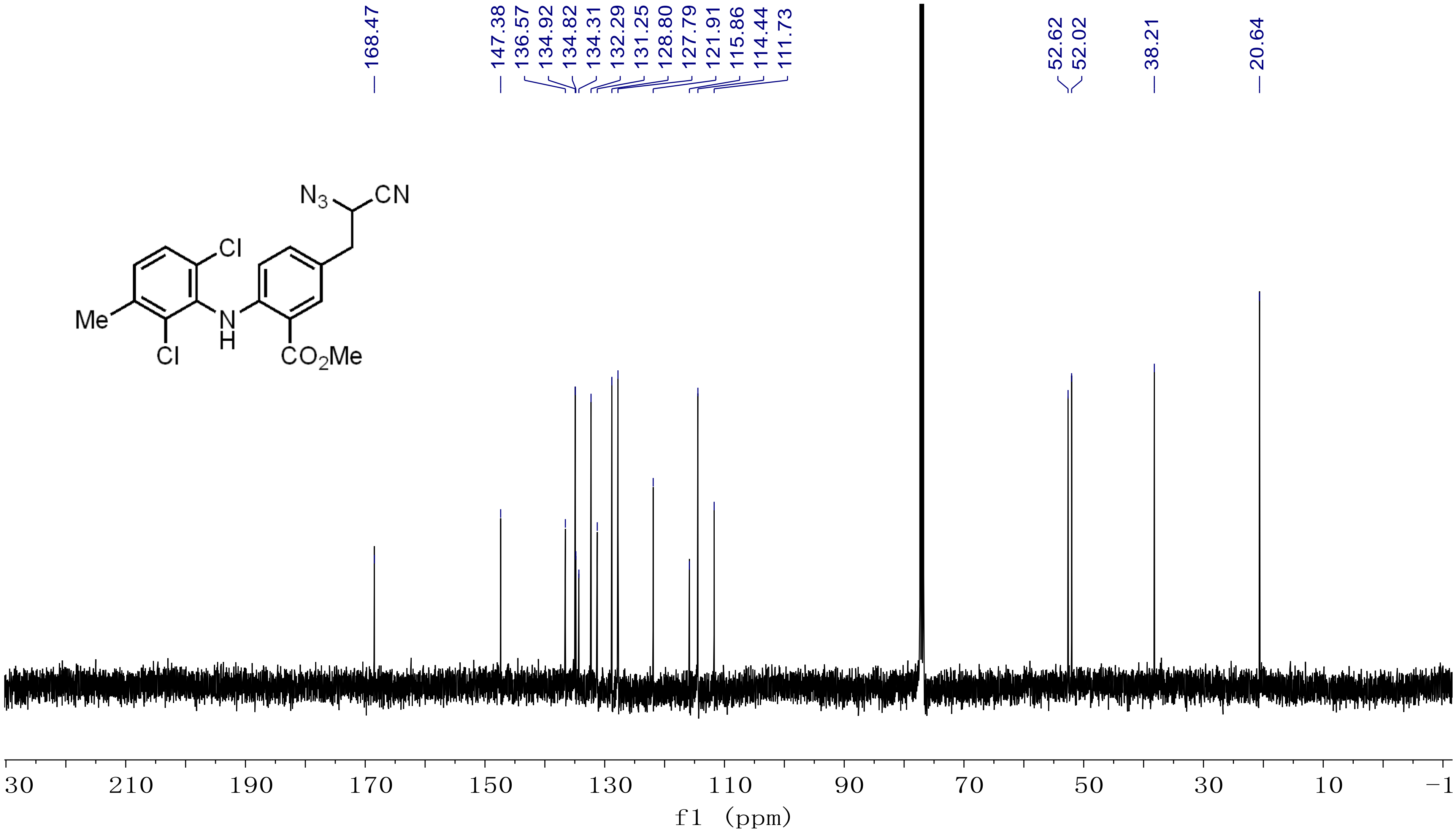

**$^{1}$H NMR of *rac*-benzbromarone-derived phenylalanine analogue 7**

$CDCl_3$, 23 ºC

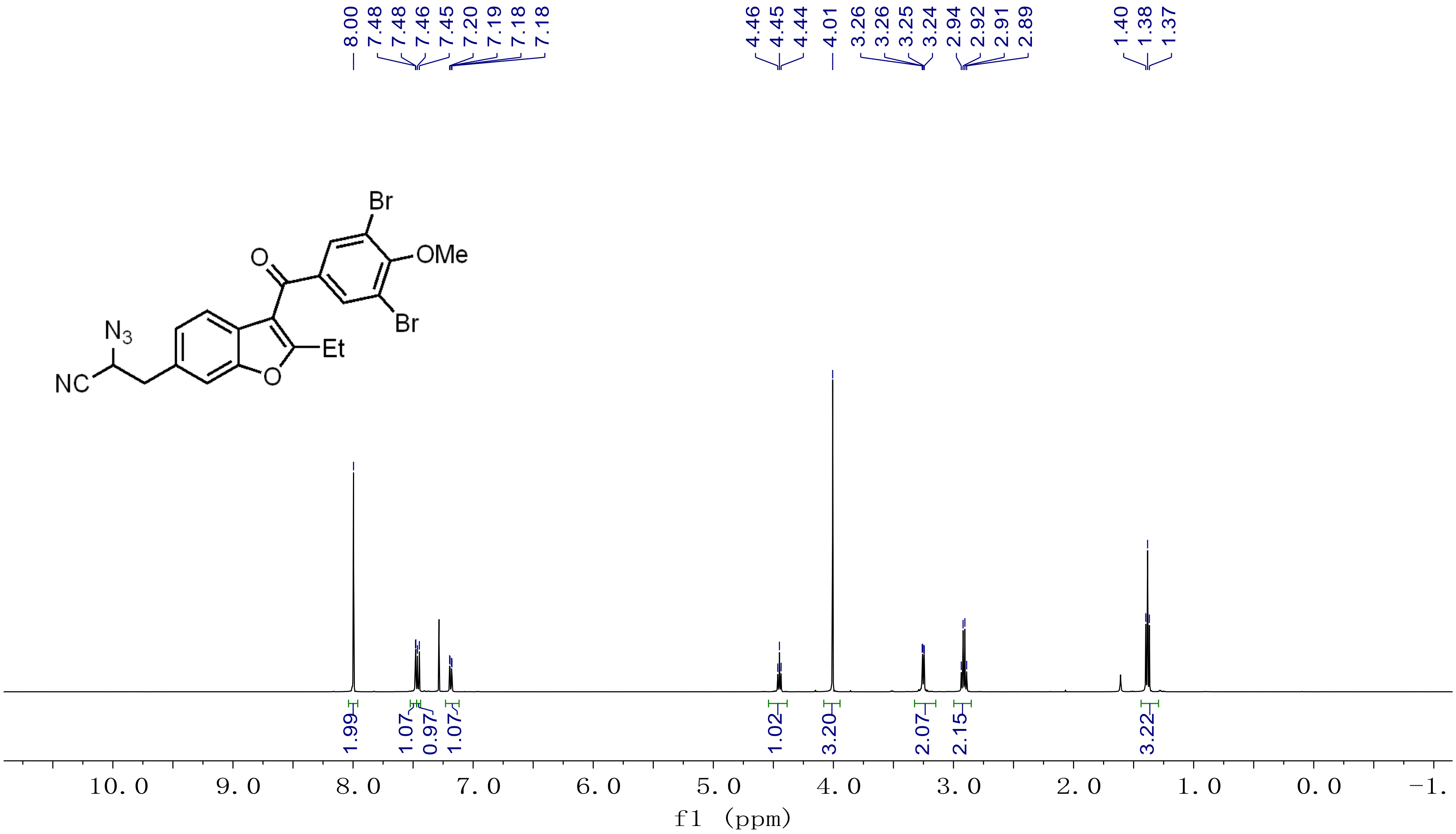

**$^{13}$C NMR of *rac*-benzbromarone-derived phenylalanine analogue 7**

CDCl$_3$, 23 ºC

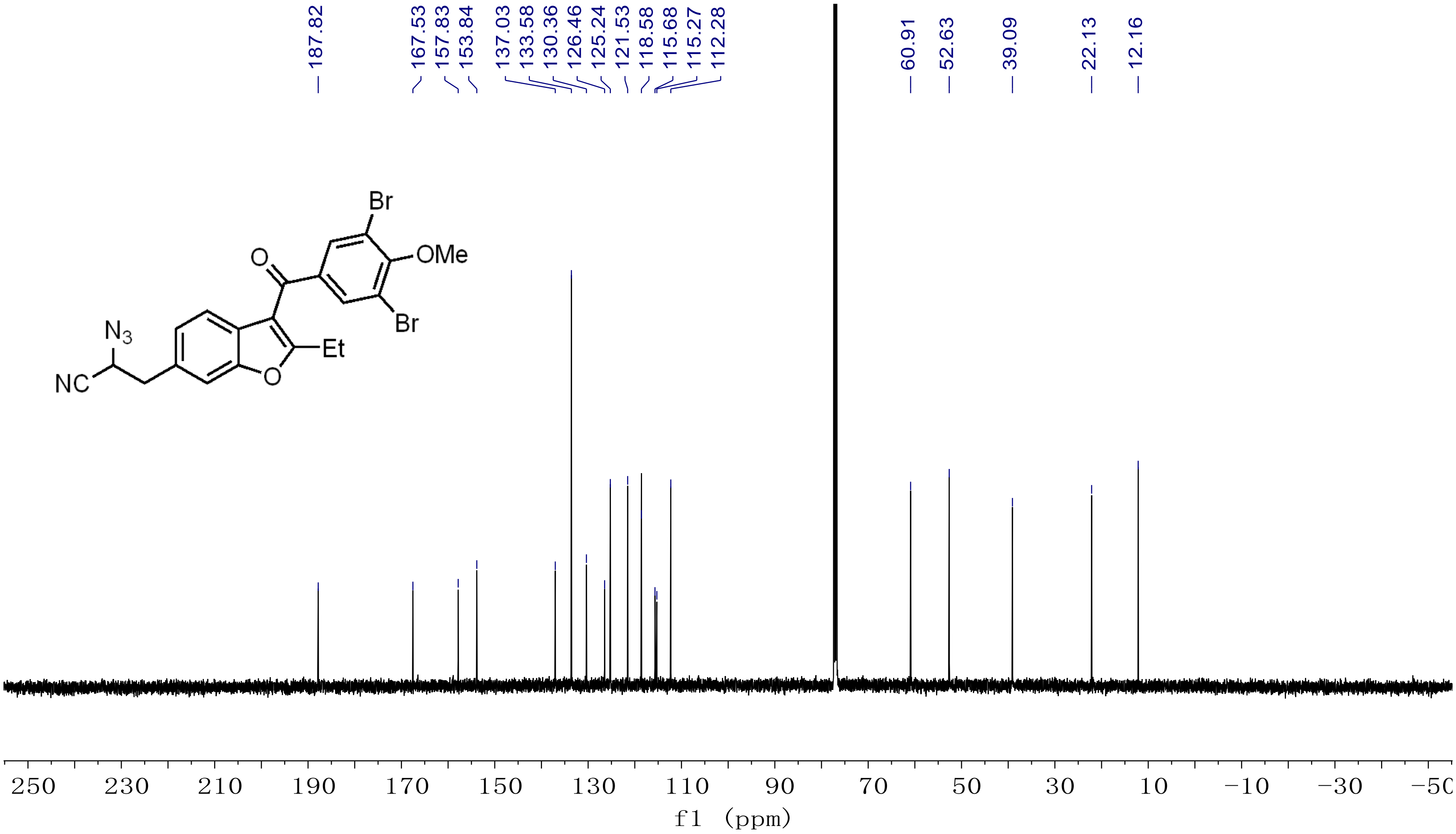

**$^{1}$H NMR of cloquintocet-mexyl-derived phenylalanine analogue 8**

$CDCl_3$, 23 ºC

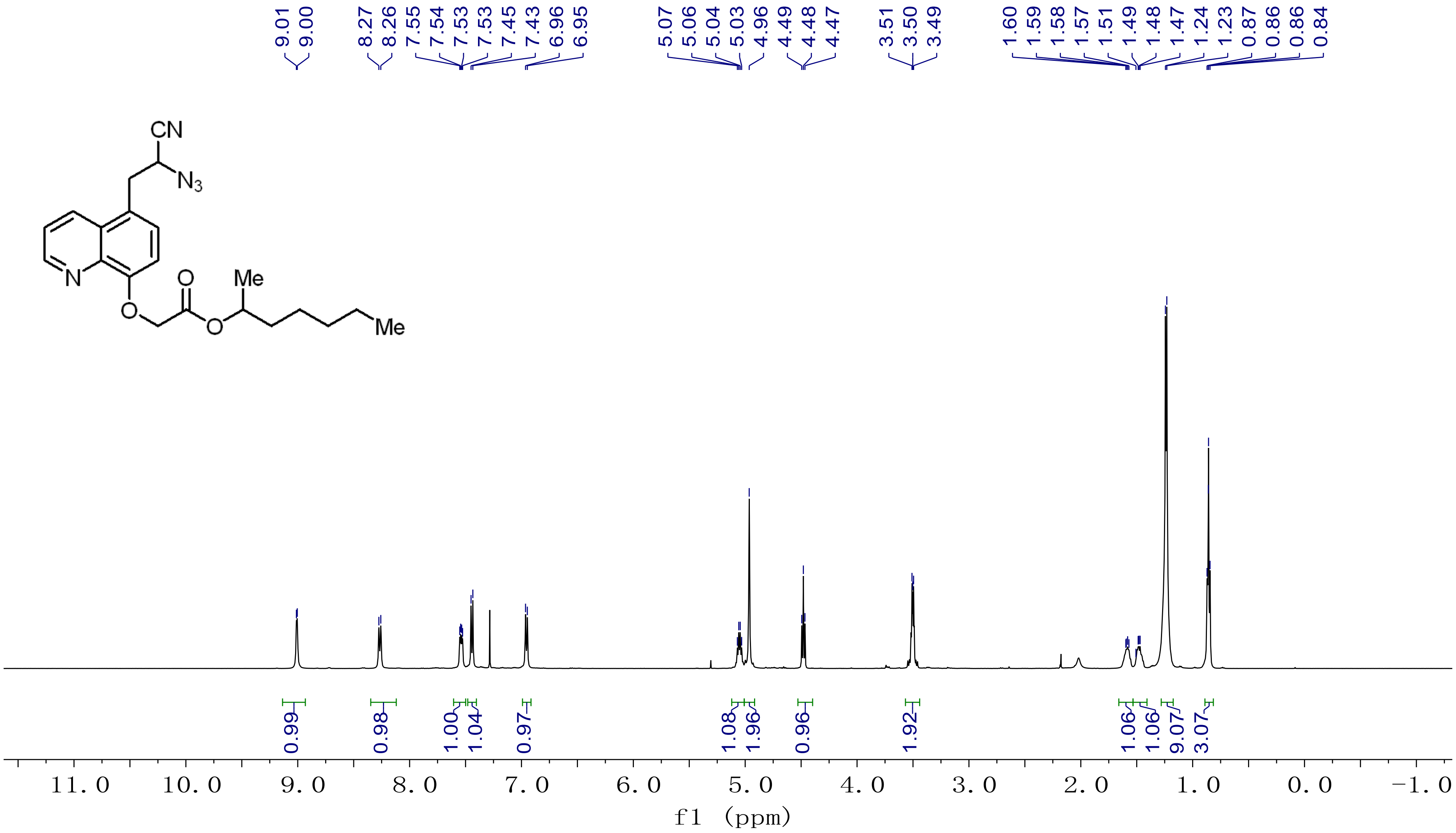

## $^{13}$C NMR of cloquintocet-mexyl-derived phenylalanine analogue 8

$CDCl_3$, 23 ºC

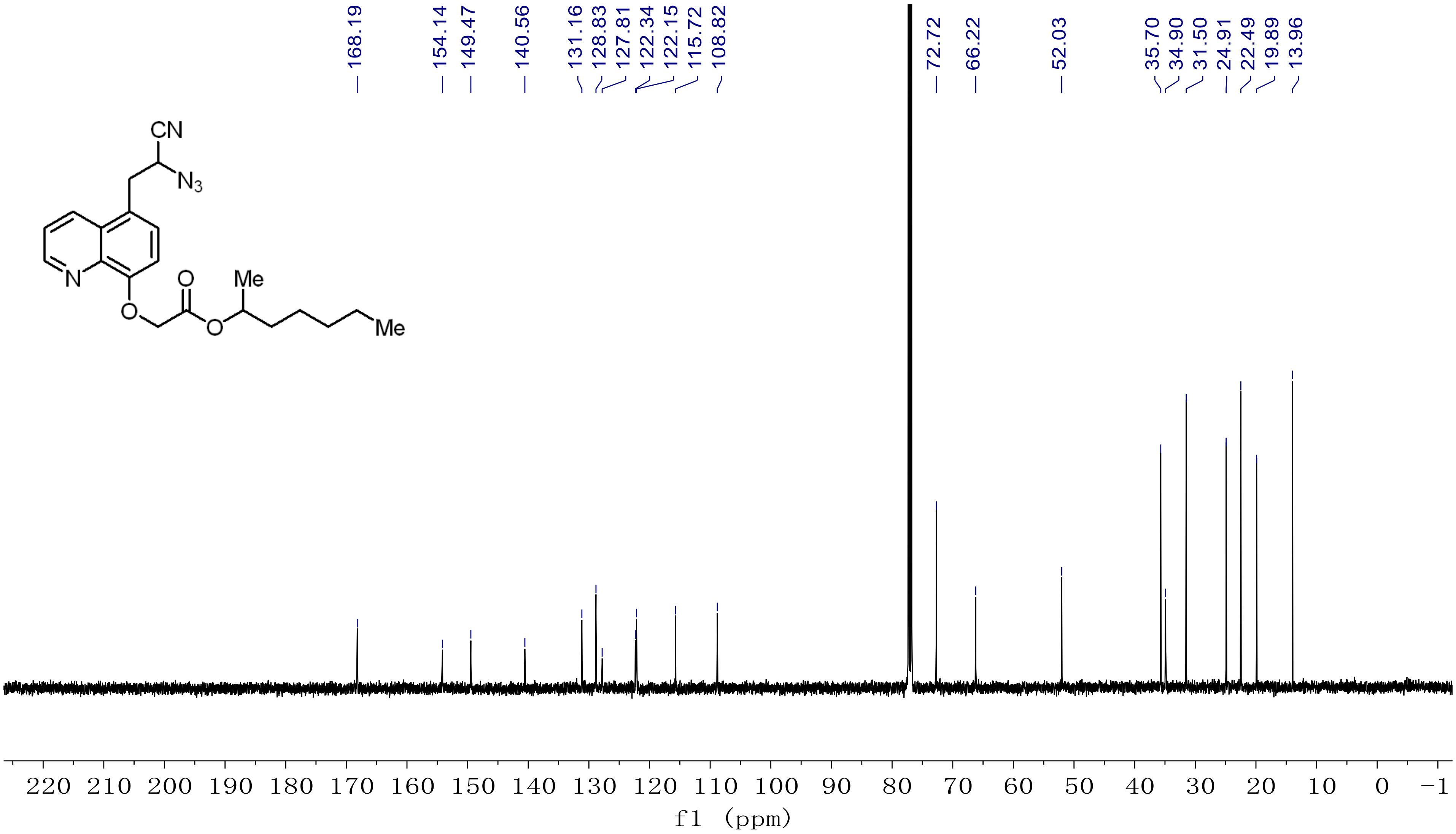

**$^{1}$H NMR of *rac*-diclofenac amide-derived phenylalanine analogue 9**

$CDCl_3$, 23 ºC

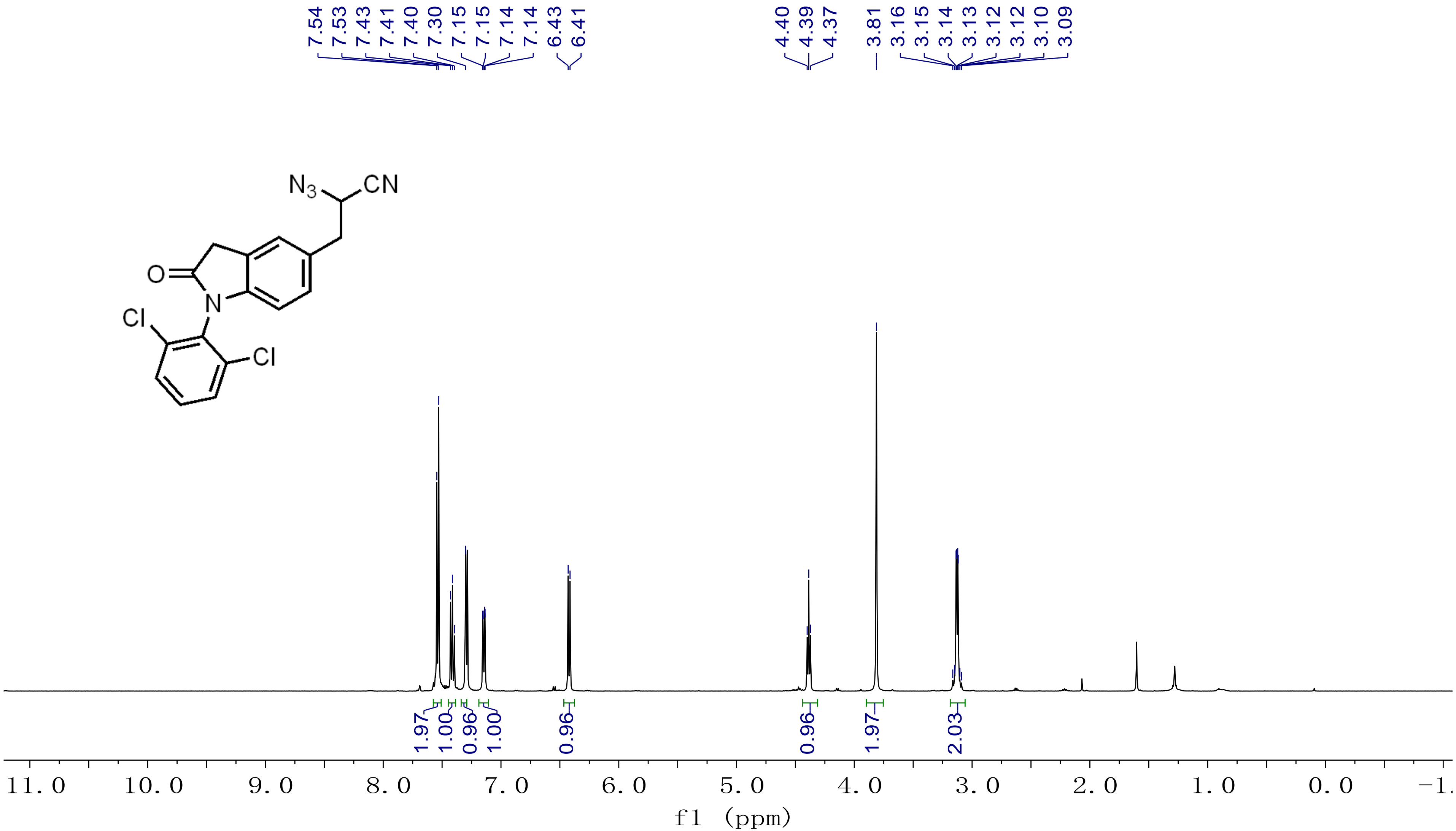

**$^{13}$C NMR of *rac*-diclofenac amide-derived phenylalanine analogue 9**

CDCl$_3$, 23 ºC

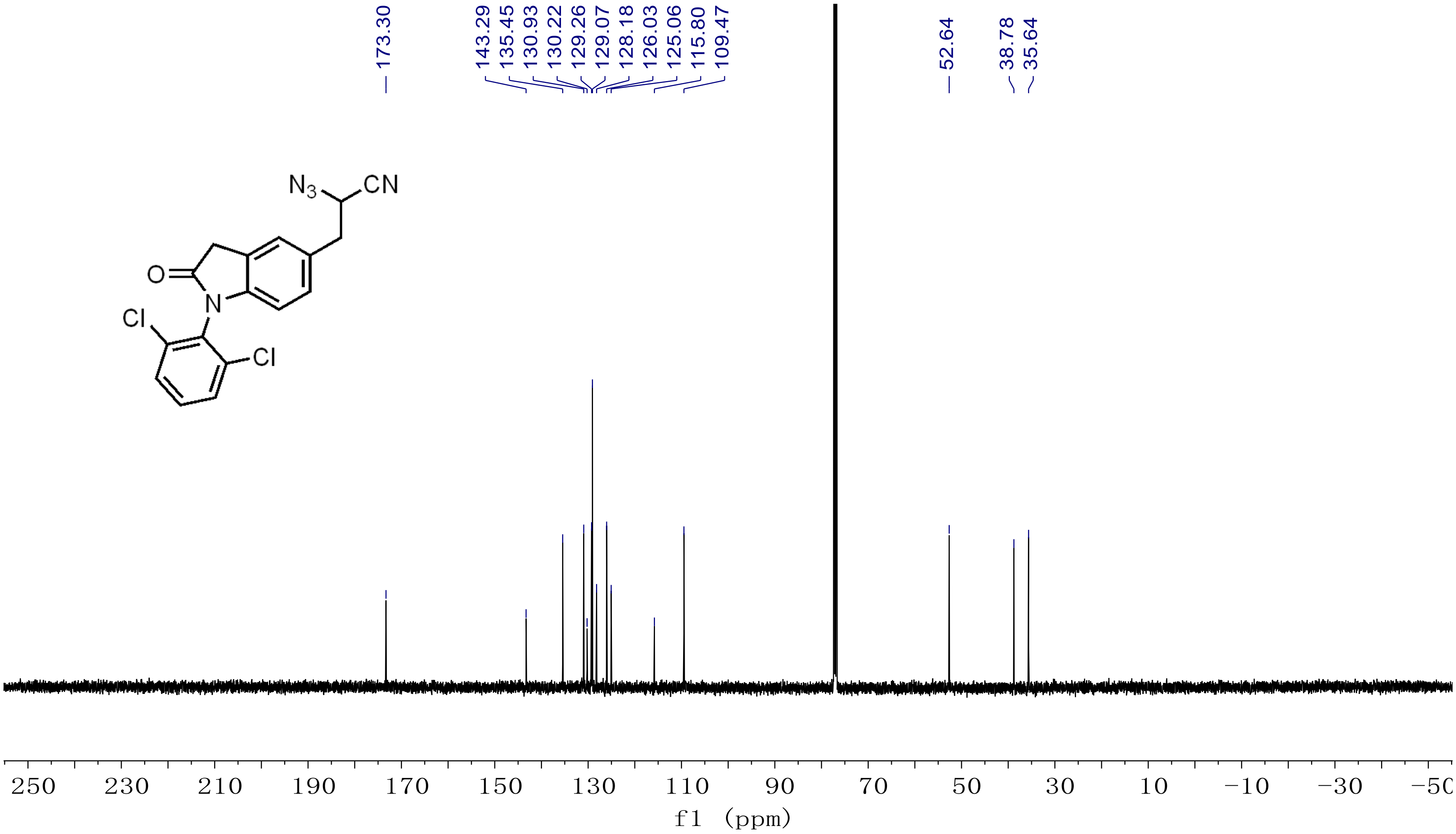

**$^{1}$H NMR of estrone-derived phenylalanine analogue 10**

$CDCl_3$, 23 ºC

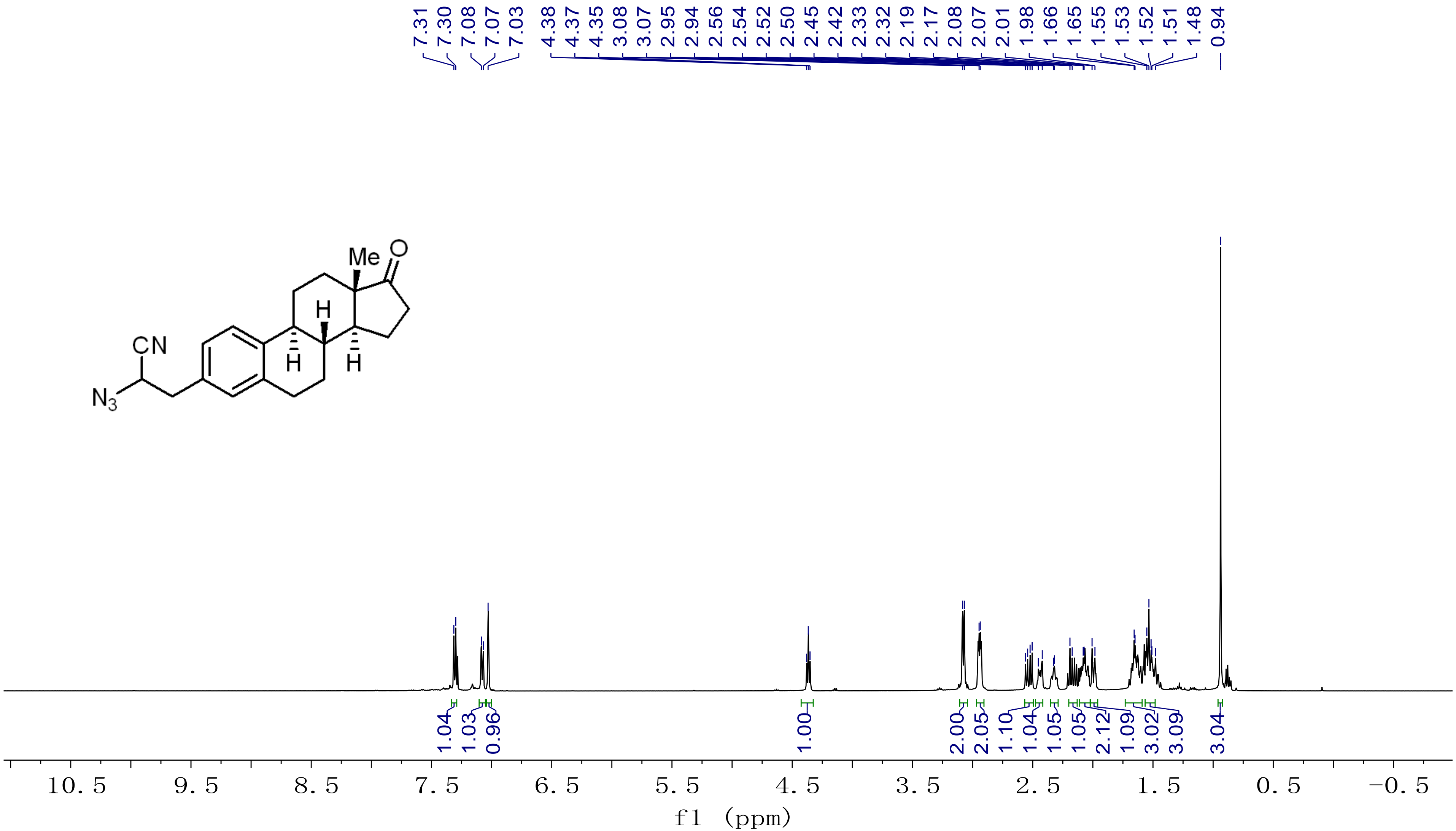

**$^{13}$C NMR of estrone-derived phenylalanine analogue 10**

$CDCl_3$, 23 ºC

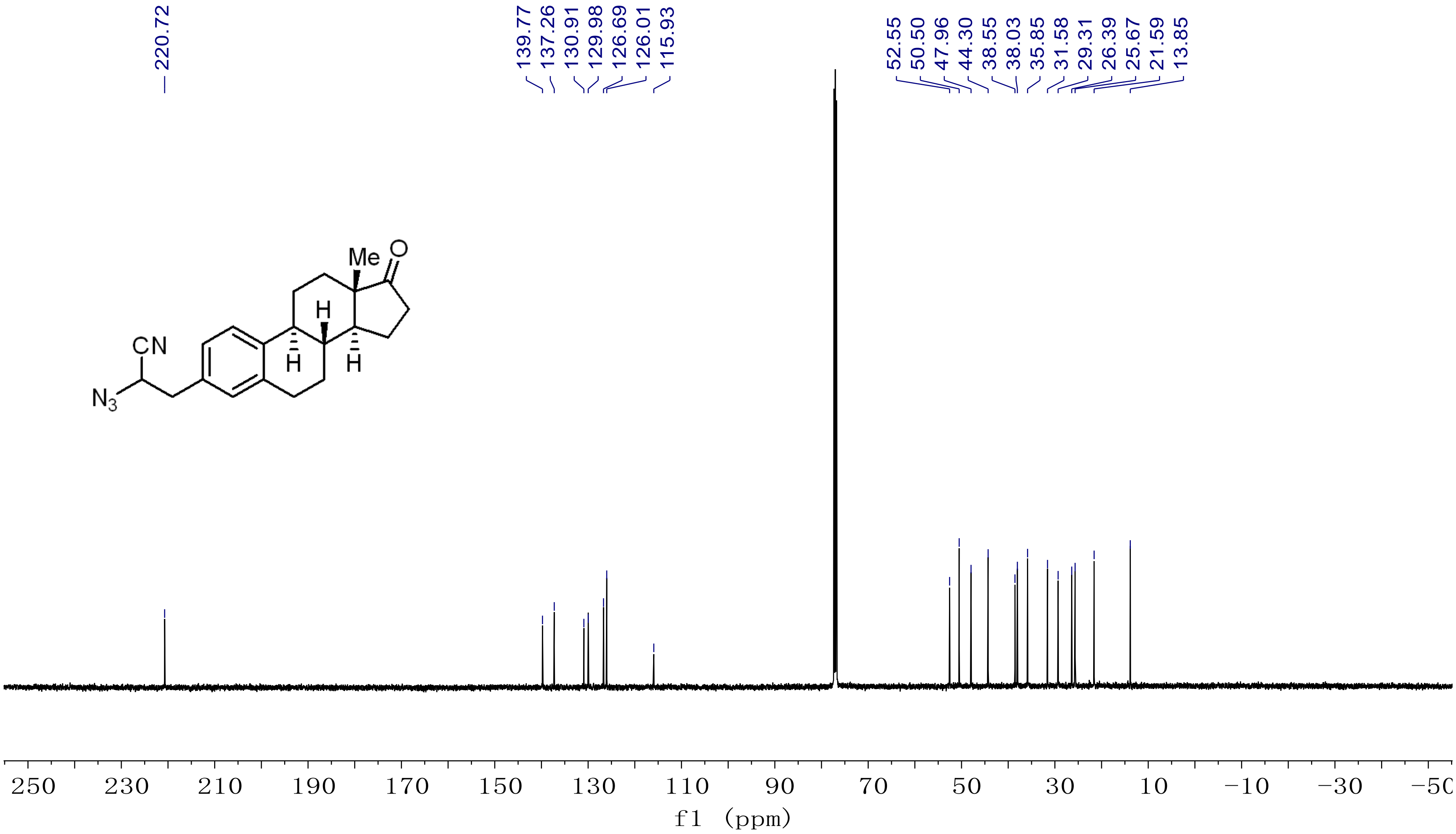

**$^{1}$H NMR *rac*-tianeptine intermediate-derived phenylalanine analogue 11**

CDCl$_3$, 23 ºC

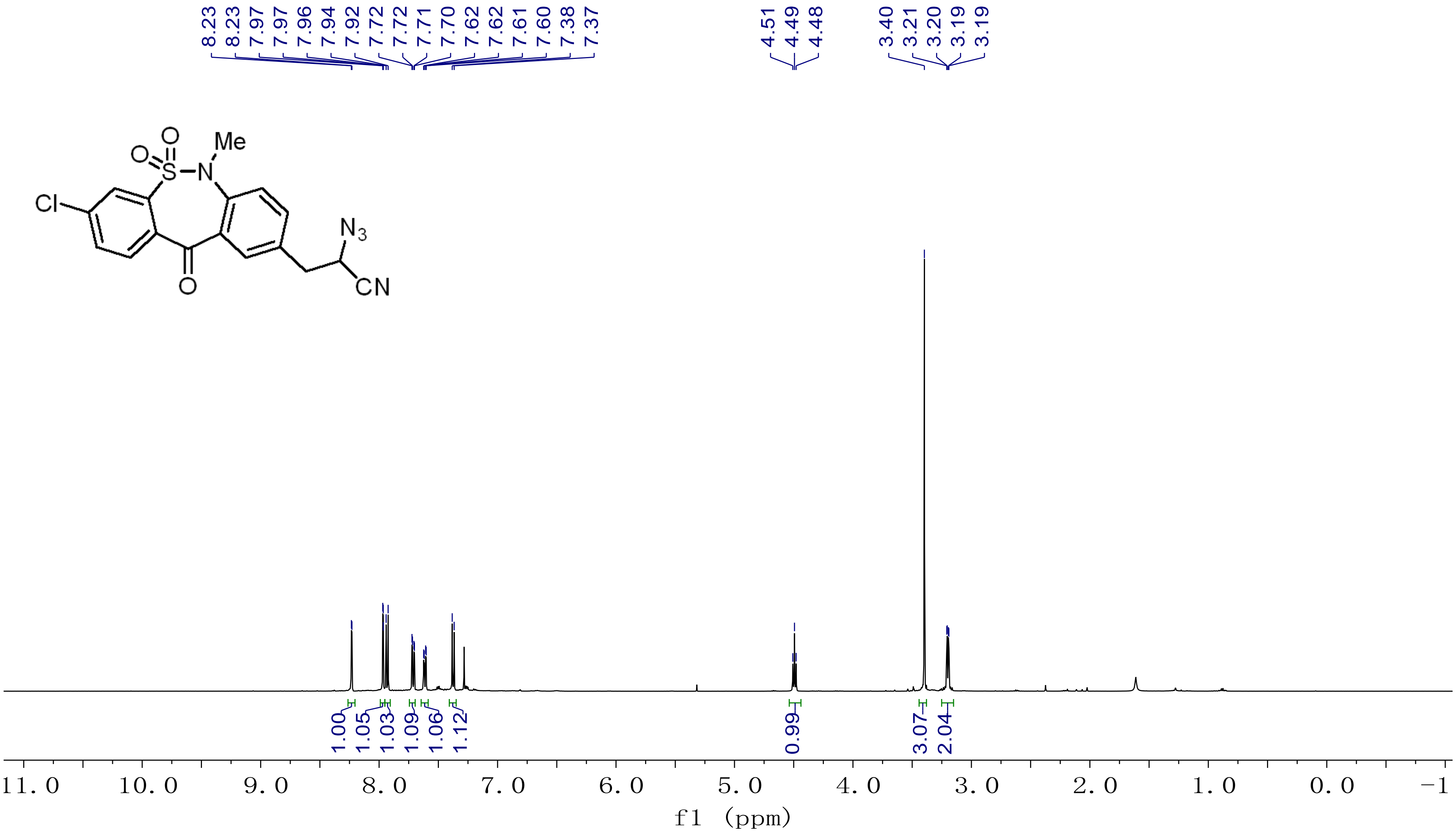

**$^{13}$C NMR of *rac*-tianeptine intermediate-derived phenylalanine analogue 11**

$CDCl_3$, 23 ºC

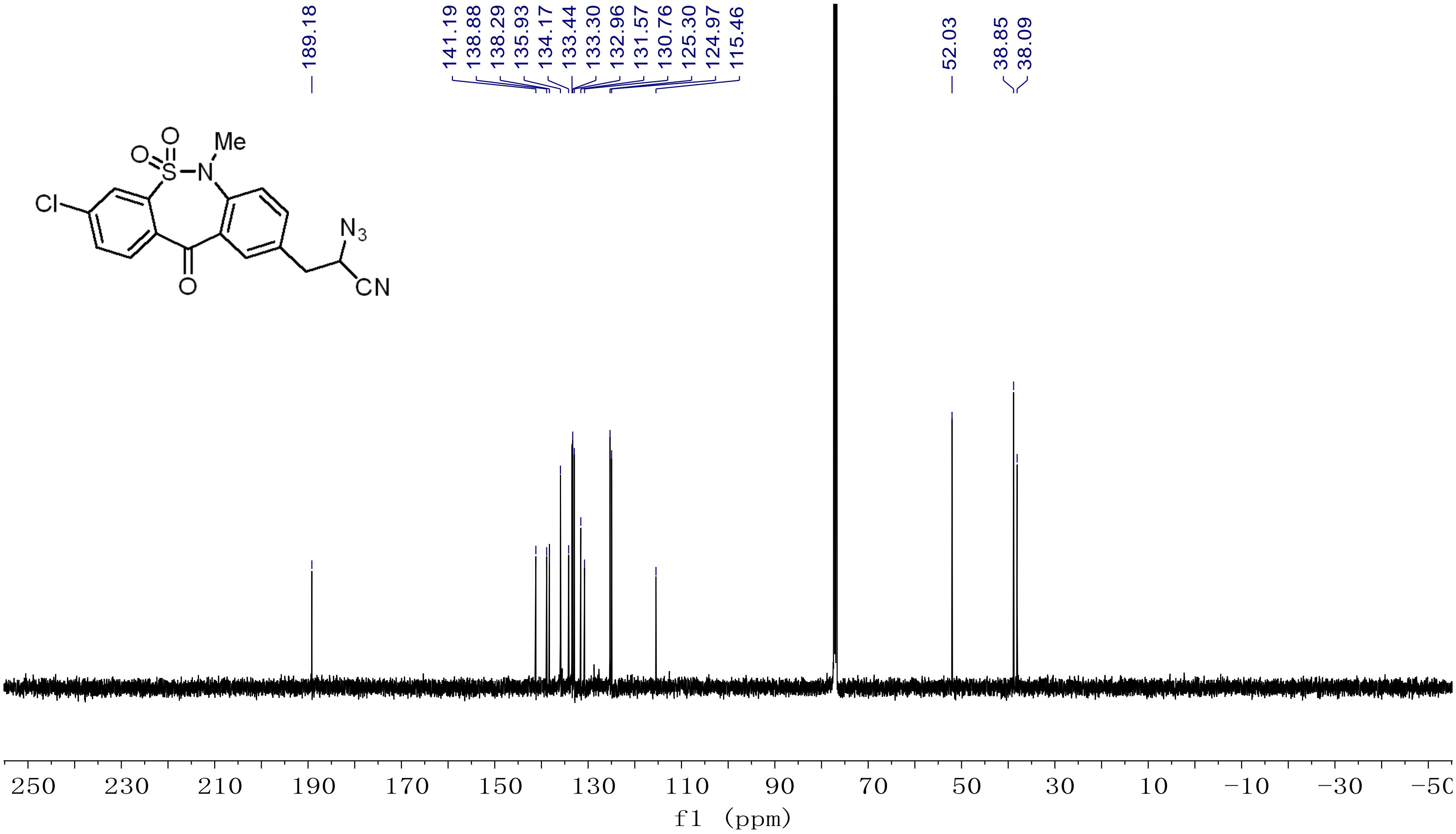

## $^1$H NMR of flubiprofen-derived phenylalanine analogue 12

CDCl$_3$, 23 ºC

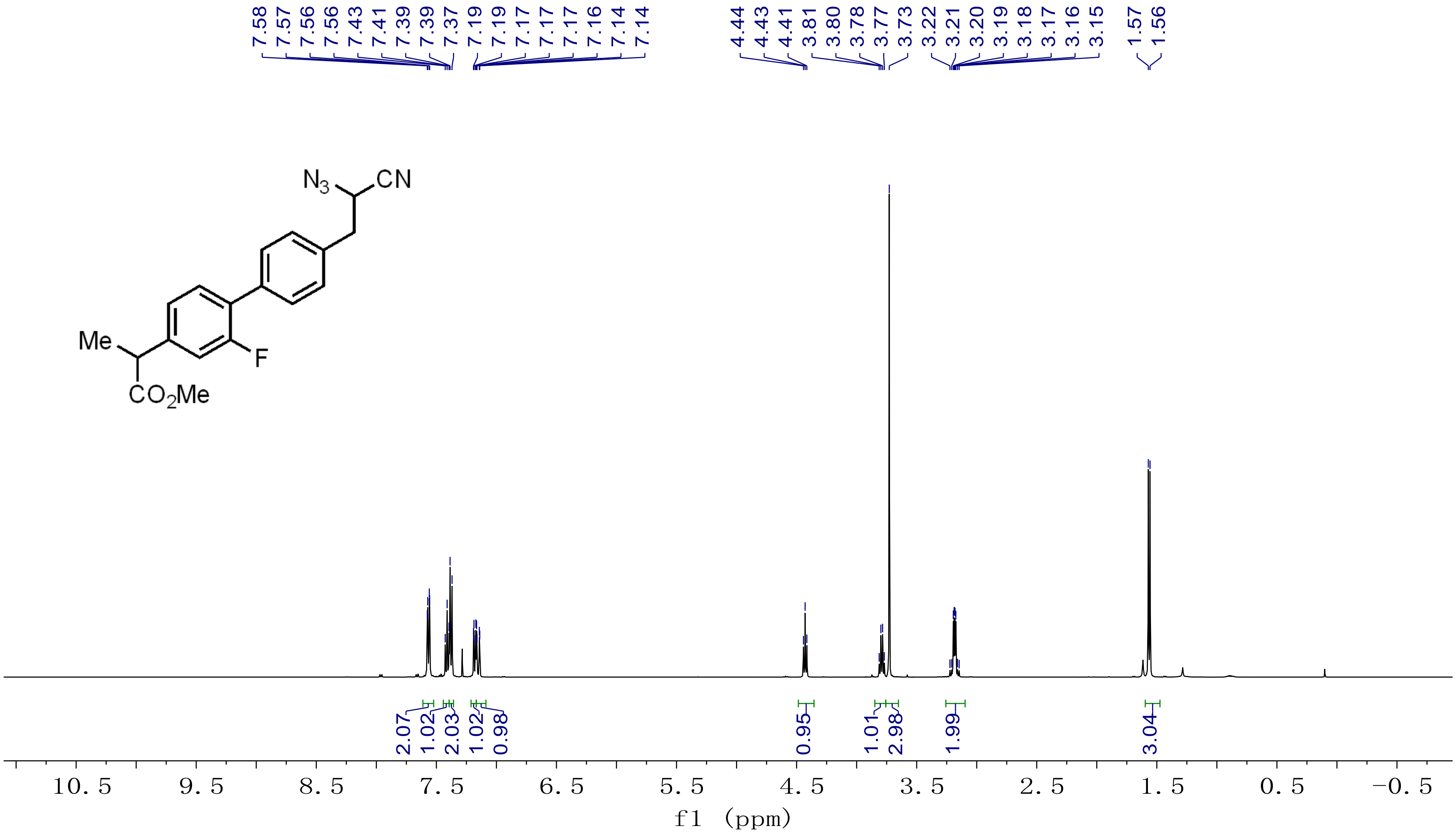

**$^{19}$F NMR of flubiprofen-derived phenylalanine analogue 12**

$CDCl_3$, 23 ºC

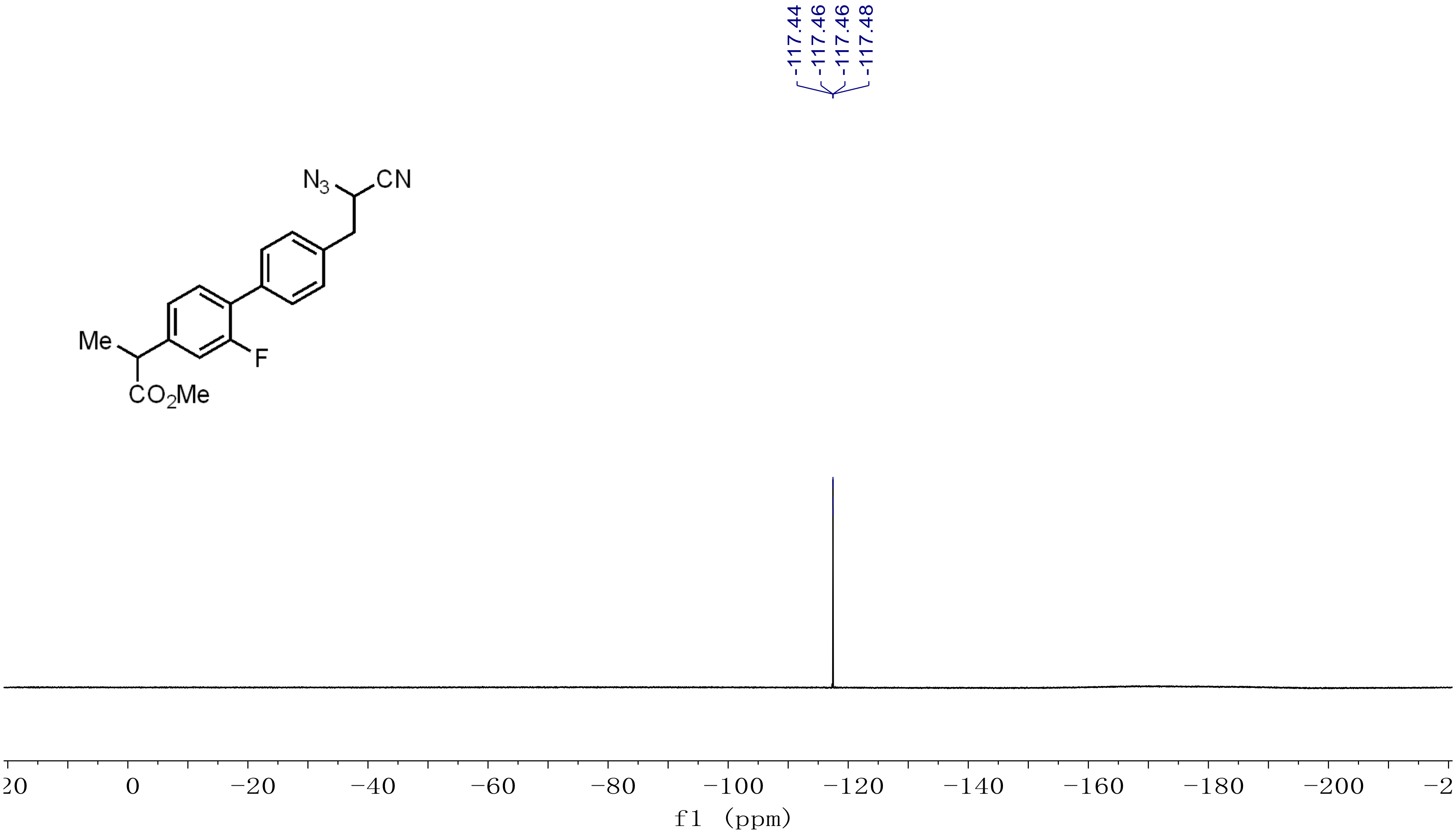

**$^{13}$C NMR of flubiprofen-derived phenylalanine analogue 12**

$CDCl_3$, 23 ºC

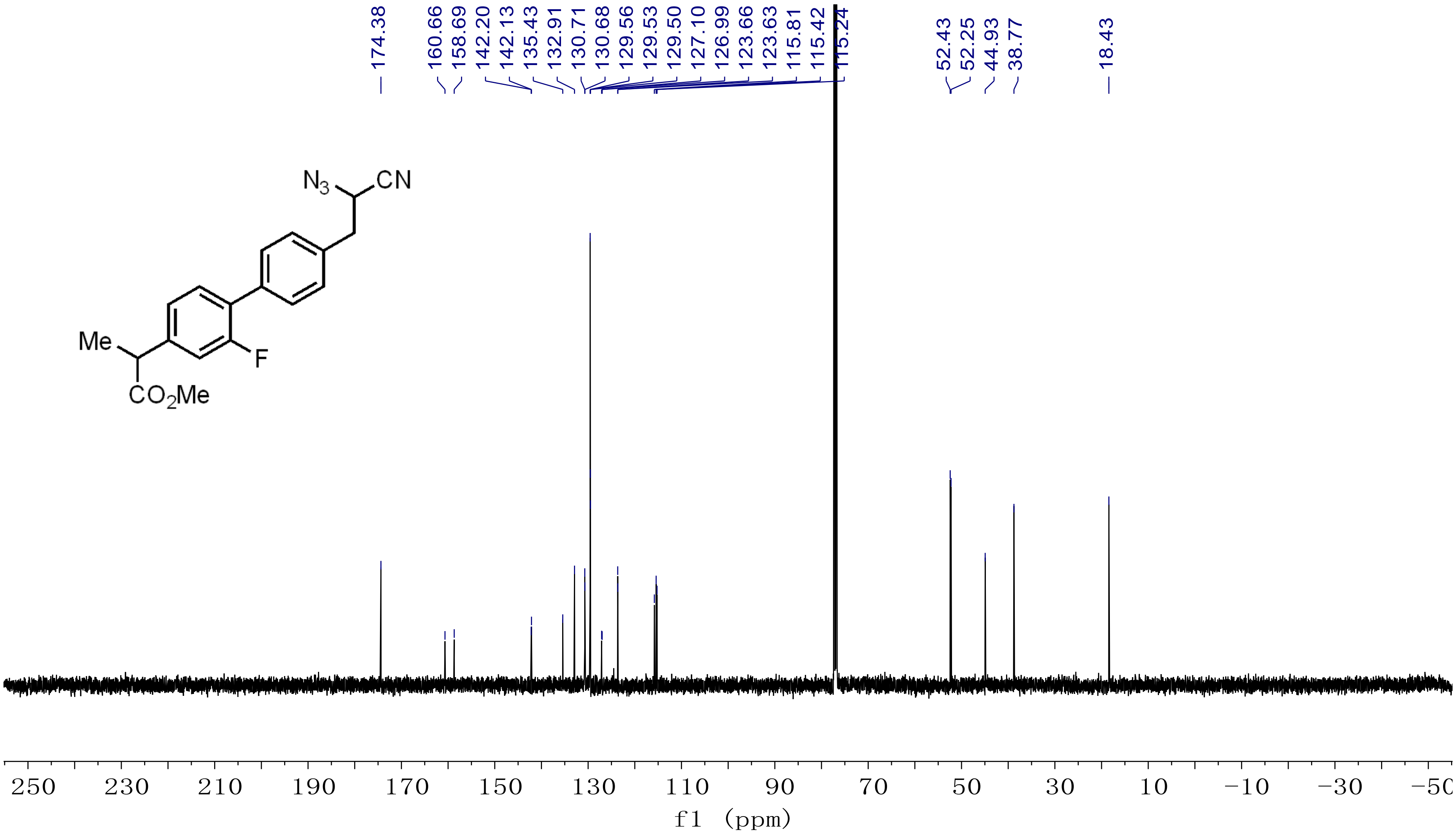

**$^{1}$H NMR of tocopherol-derived phenylalanine analogue 13**

CDCl3, 23 ºC

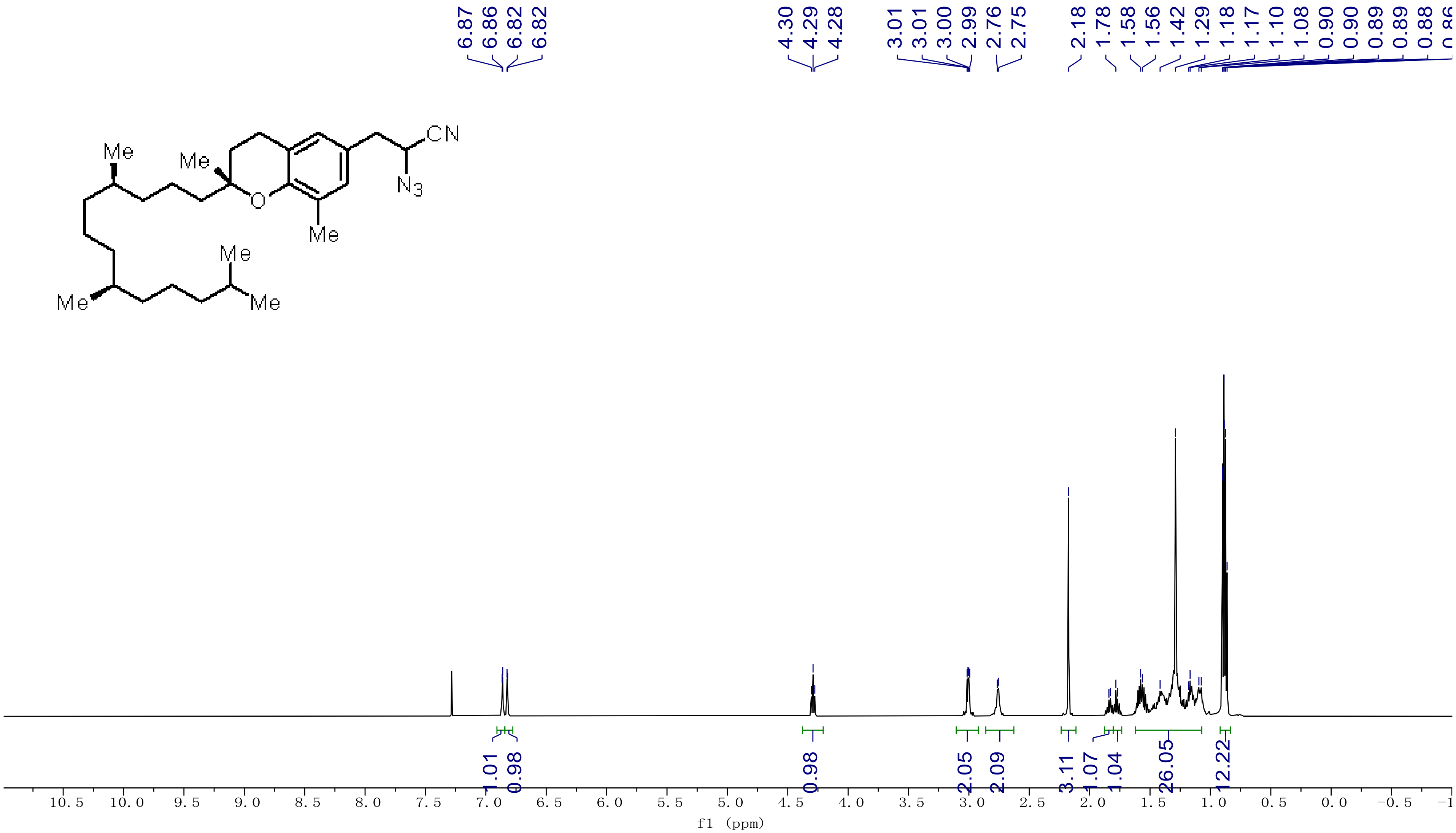

**$^{13}$C NMR of tocopherol-derived phenylalanine analogue 13**

$CDCl_3$, 23 ºC

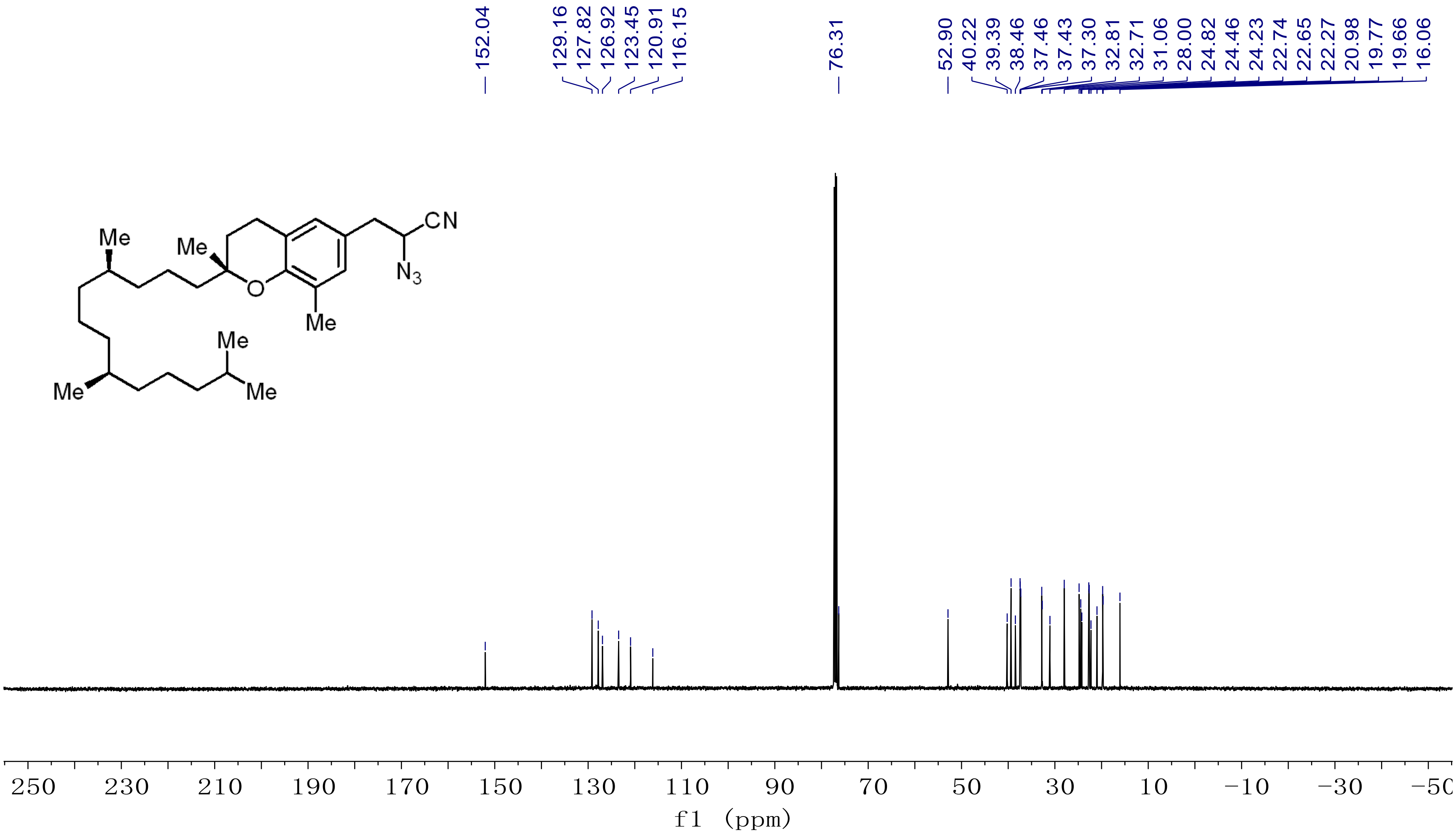

**$^{1}$H NMR of *rac*-quinoline-derived phenylalanine analogue 14**

$CDCl_3$, 23 ºC

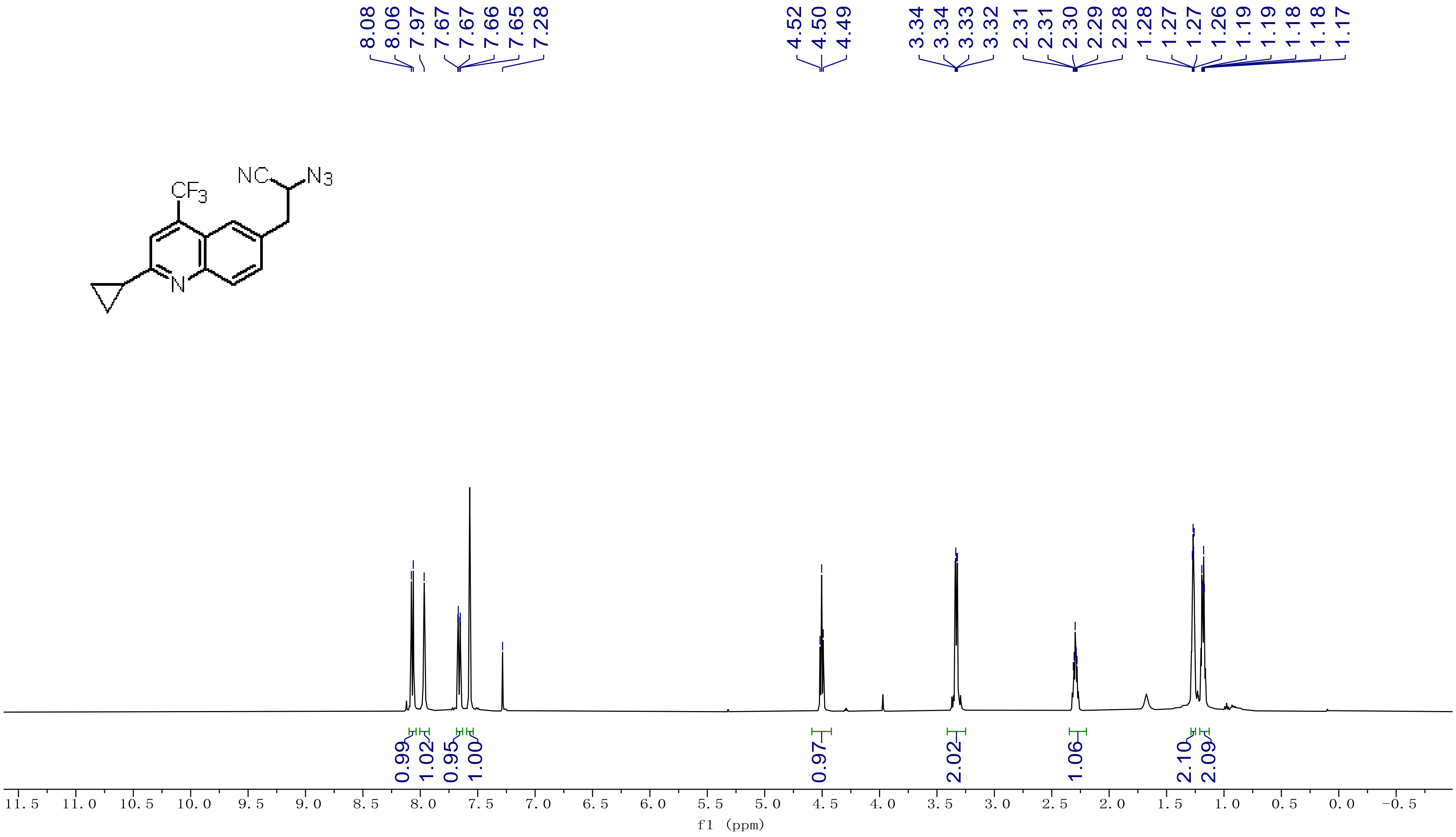

### $^{19}$F NMR of *rac*-quinoline-derived phenylalanine analogue 14

CDCl₃, 23 ºC

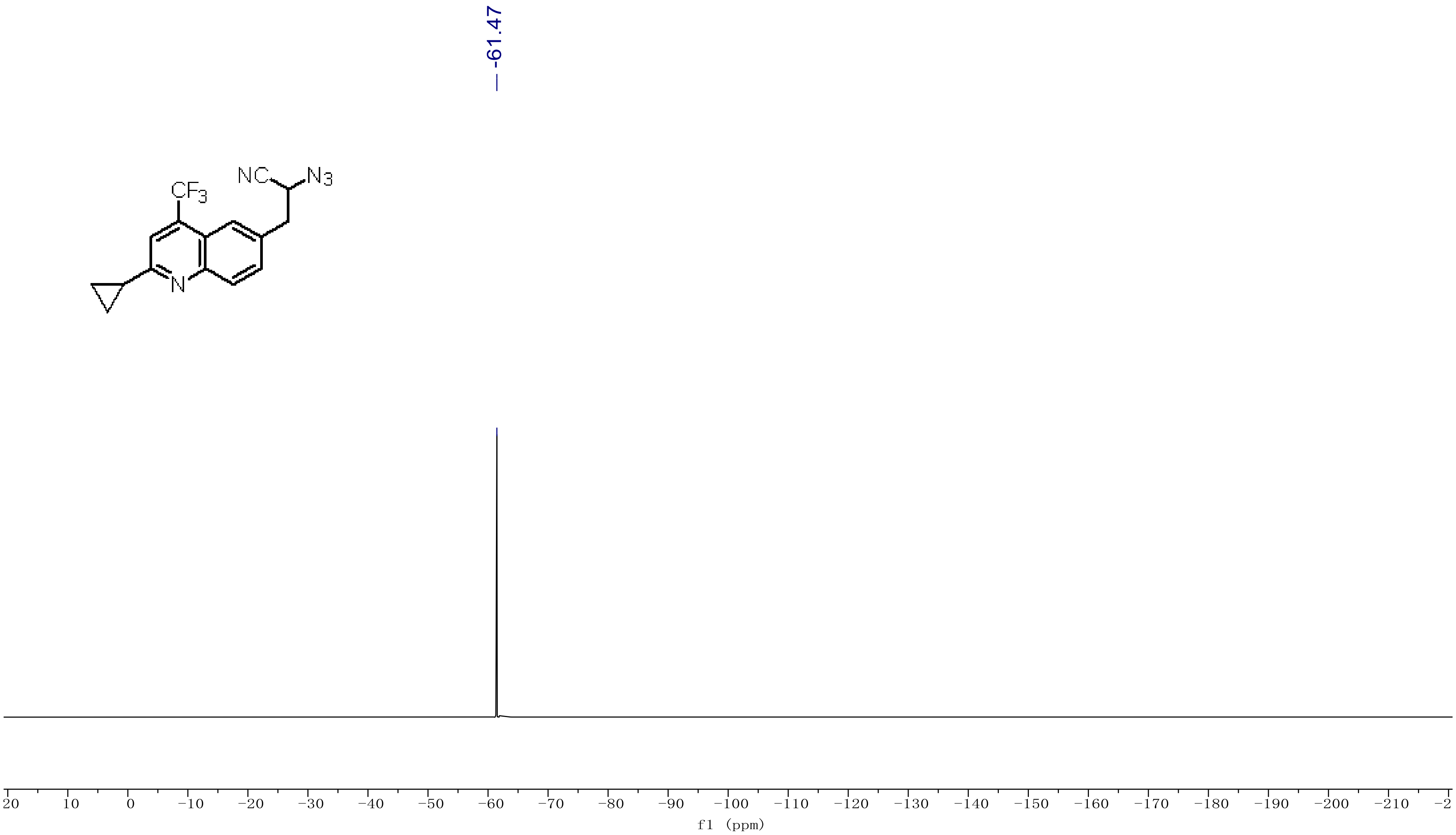

**$^{13}$C NMR of *rac*-quinoline-derived phenylalanine analogue 14**

$CDCl_3$, 23 ºC

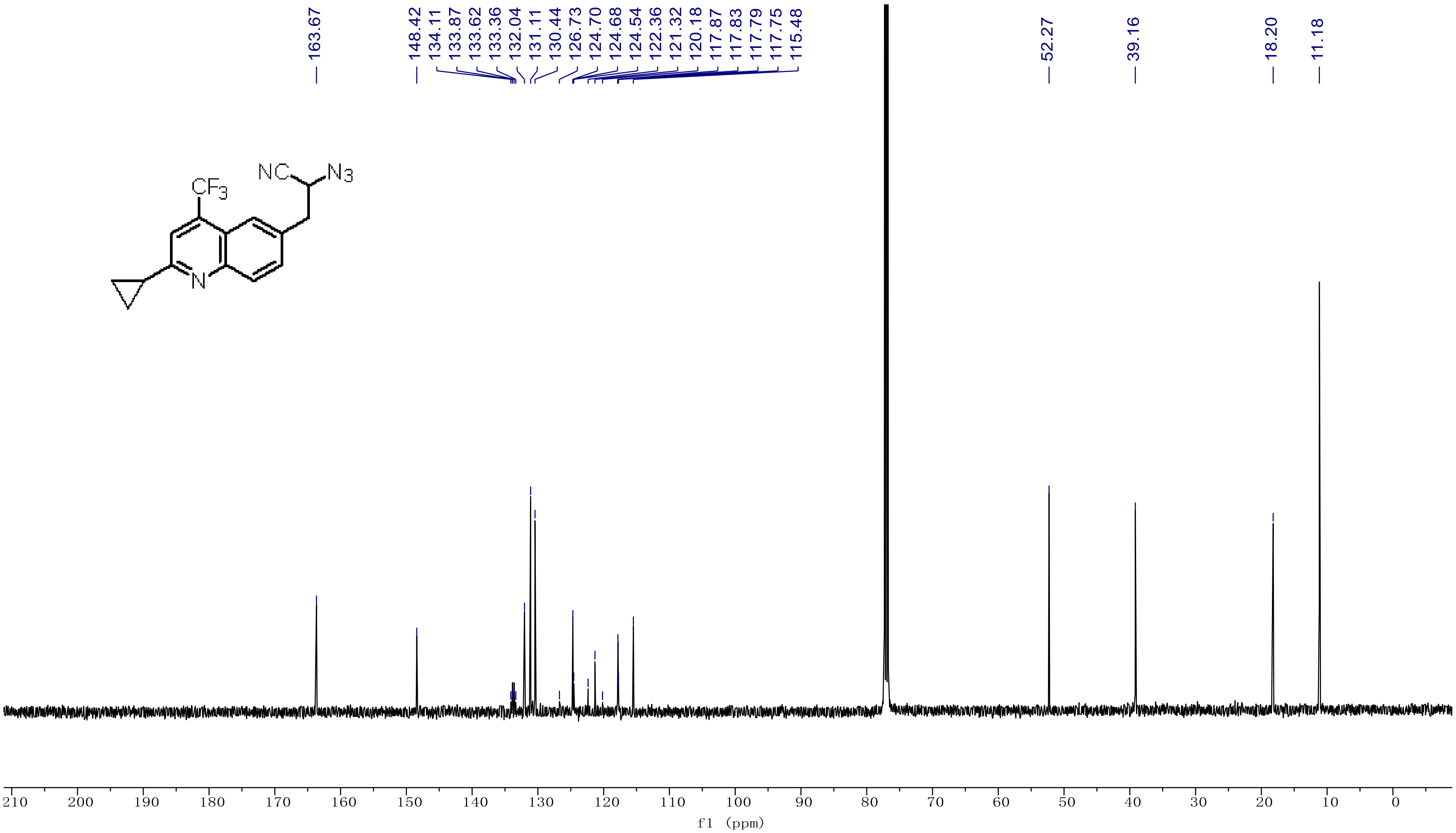

**$^{1}$H NMR of *rac*-niflumic acid-derived phenylalanine analogue 15**

$CDCl_3$, 23 ºC

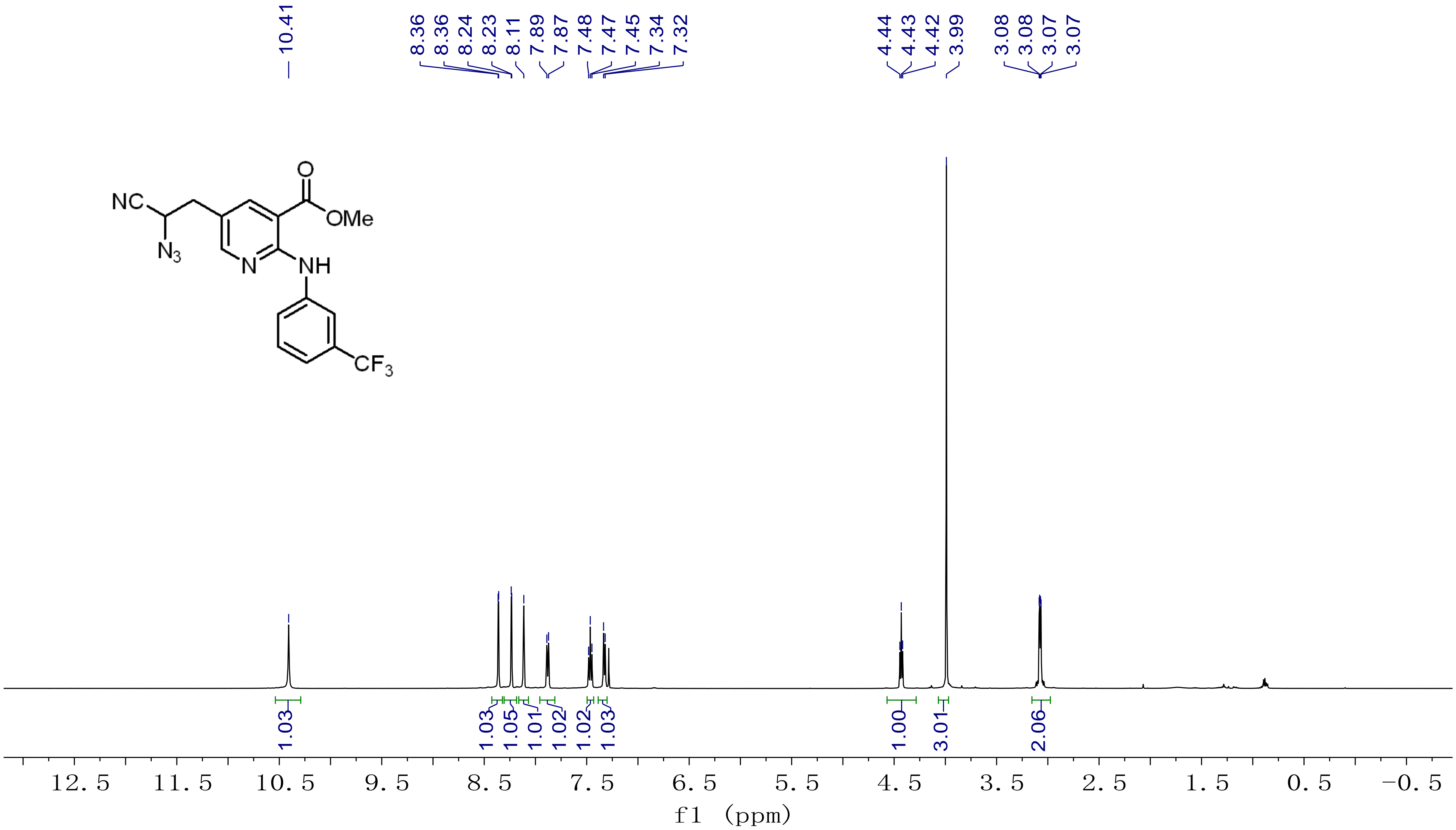

**$^{19}$F NMR of *rac*-niflumic acid-derived phenylalanine analogue 15**

$CDCl_3$, 23 ºC

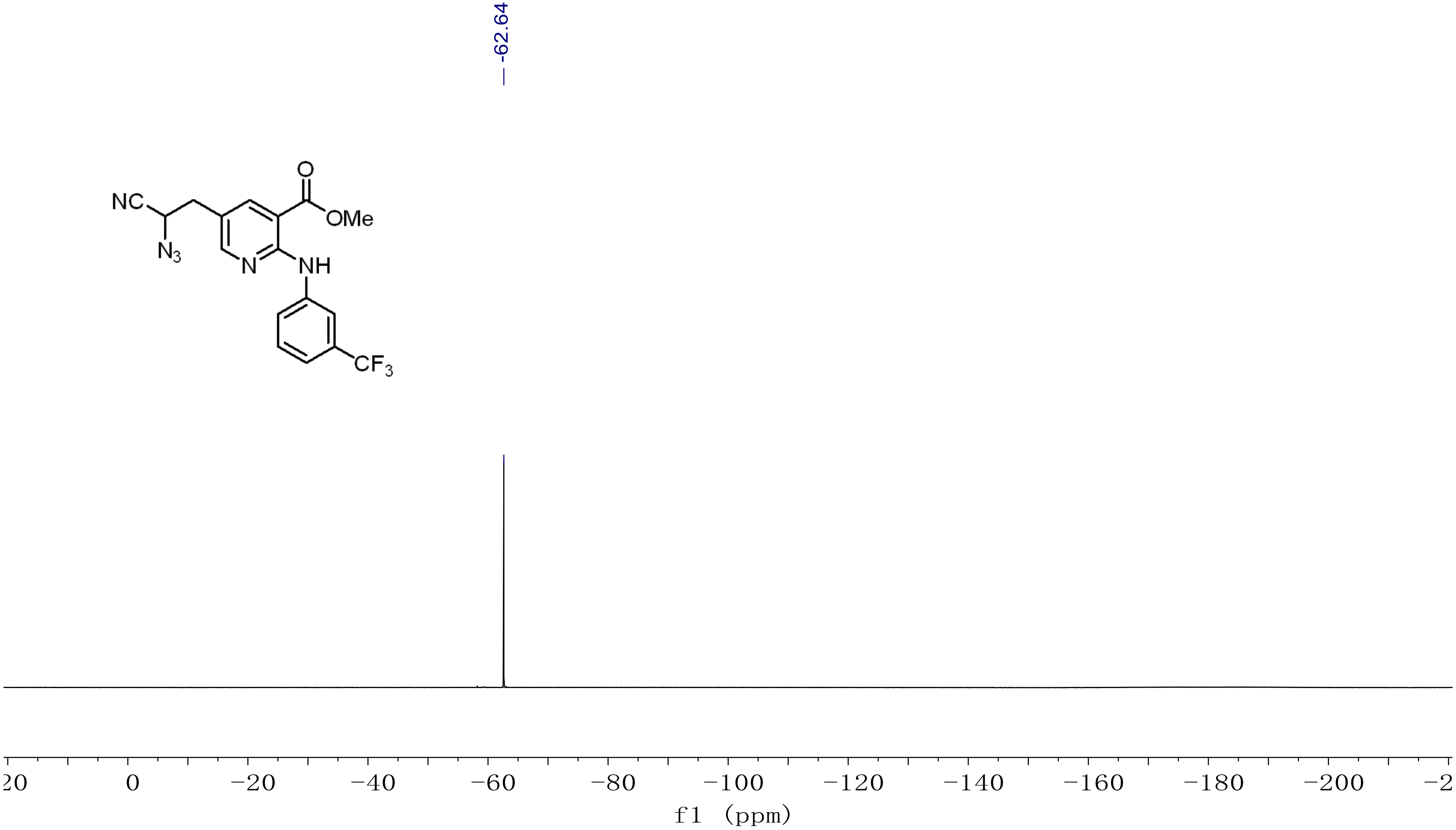

**$^{13}$C NMR of *rac*-niflumic acid-derived phenylalanine analogue 15**

$CDCl_3$, 23 ºC

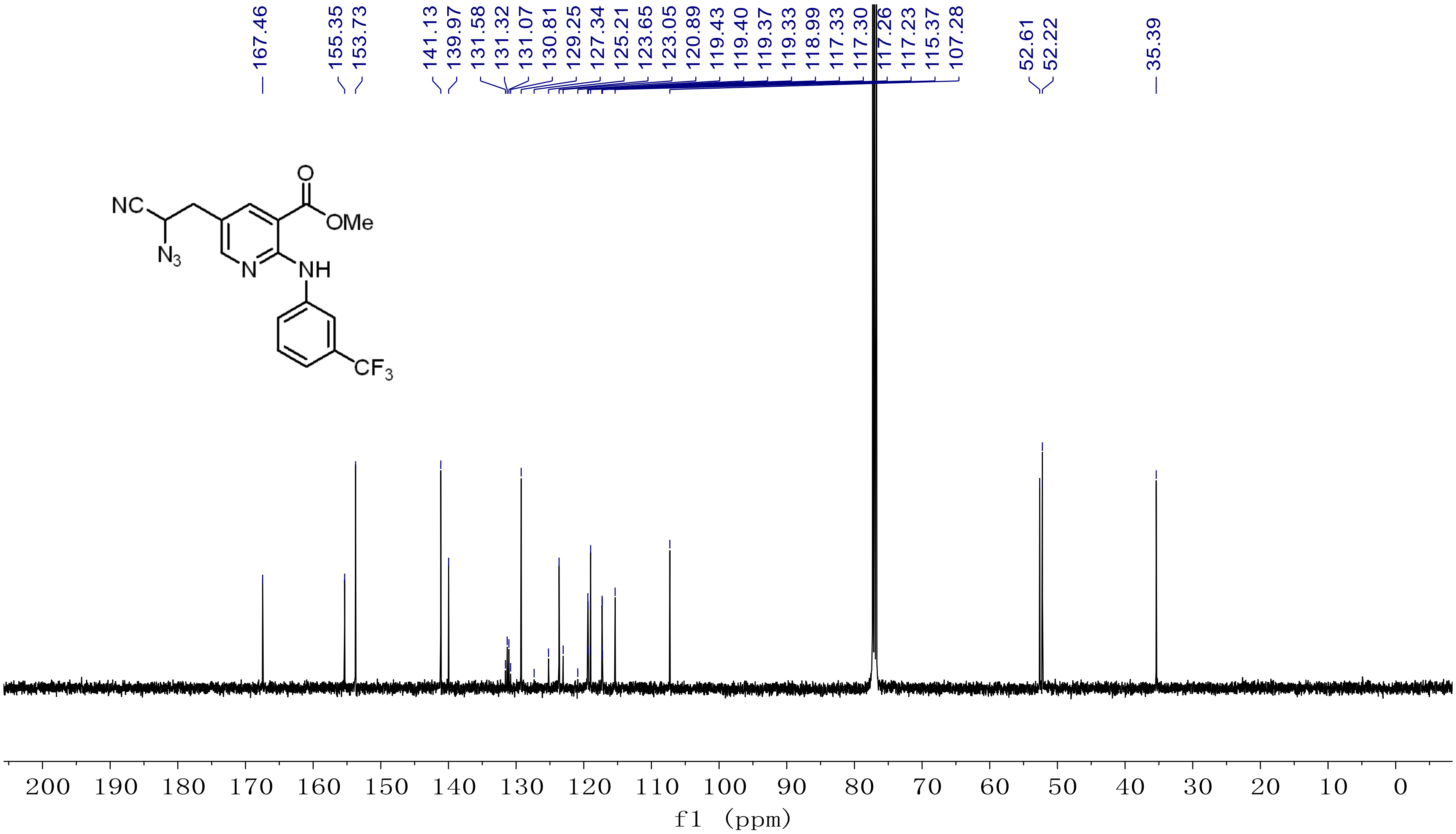

**$^{1}$H NMR of *rac*-boscalid-derived phenylalanine analogue 16**

$CDCl_3$, 23 ºC

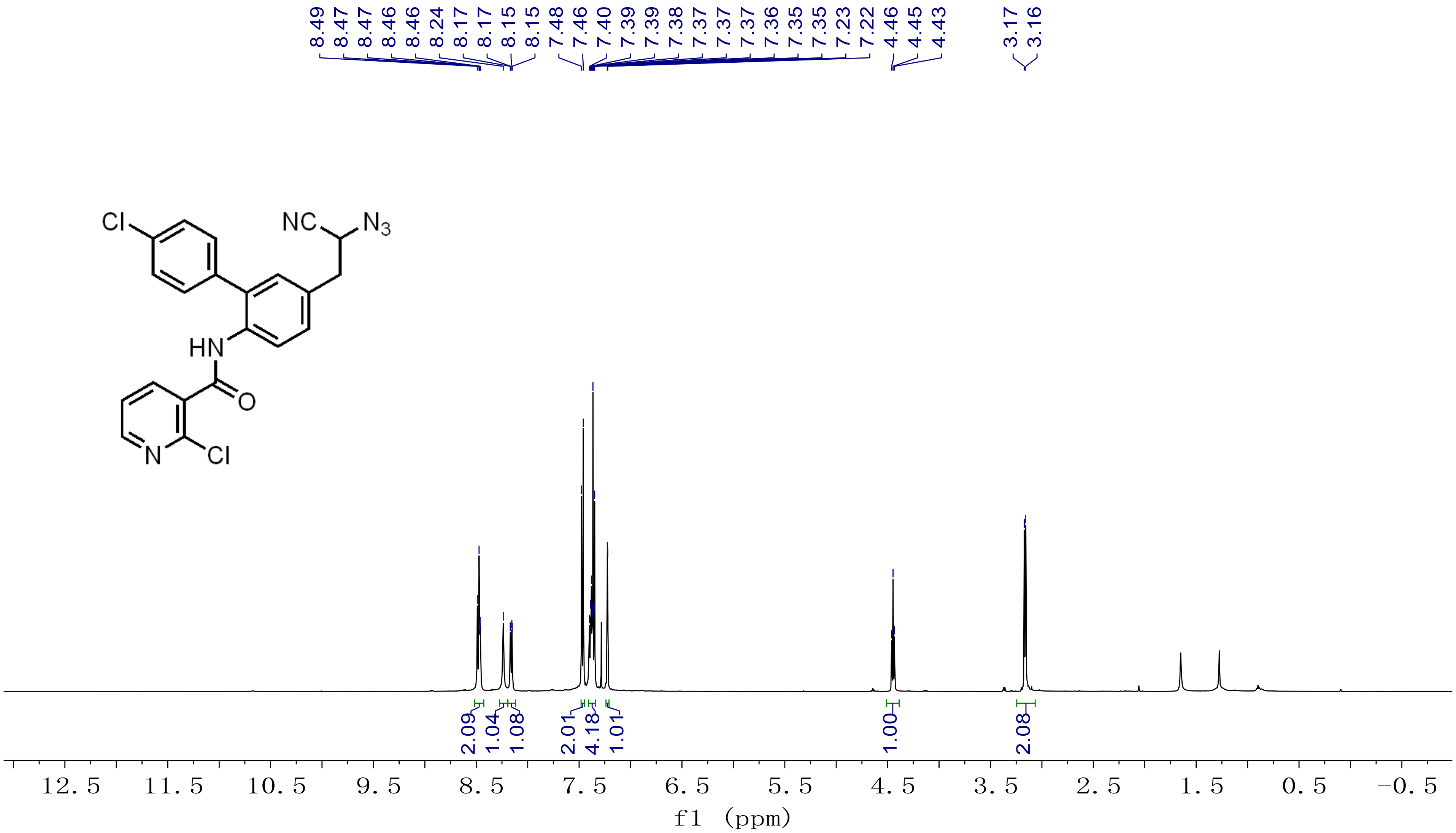

**$^{13}$C NMR of *rac*-boscalid-derived phenylalanine analogue 16**

CDCl$_3$, 23 ºC

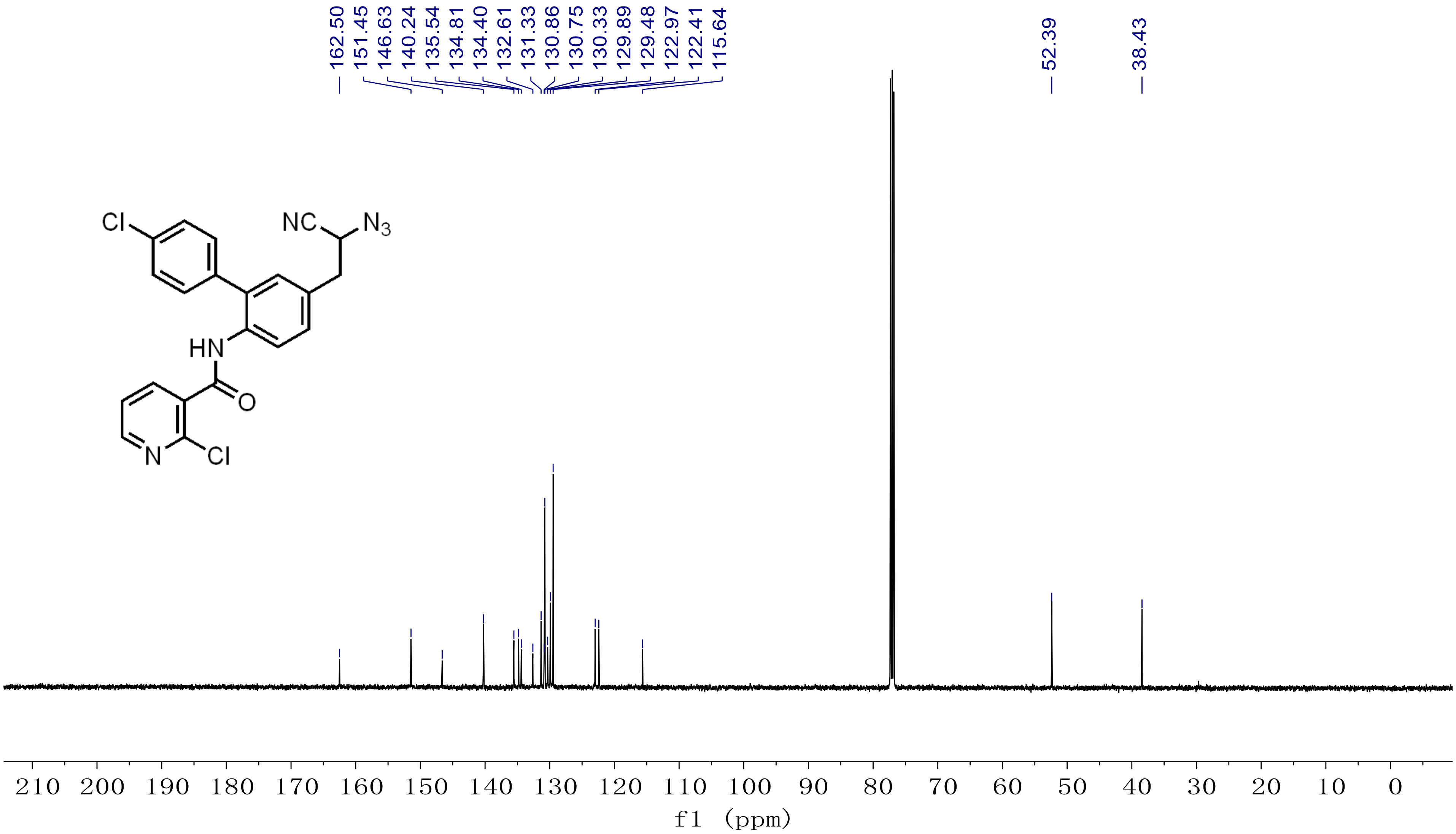

**$^{1}$H NMR of pyriproxyphen-derived phenylalanine analogue 17**

$CDCl_3$, 23 ºC

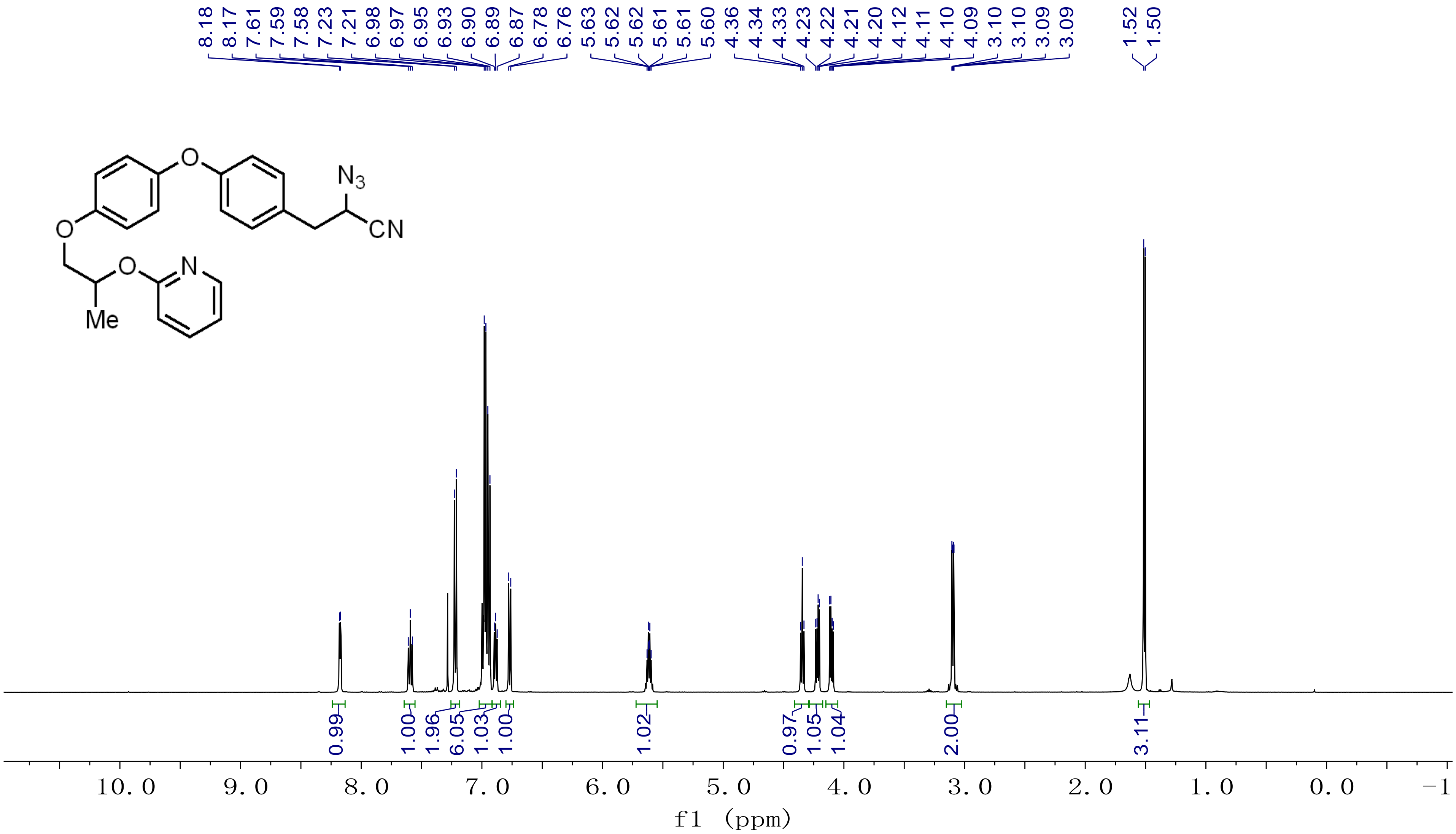

**$^{13}$C NMR of pyriproxyphen-derived phenylalanine analogue 17**

$CDCl_3$, 23 ºC

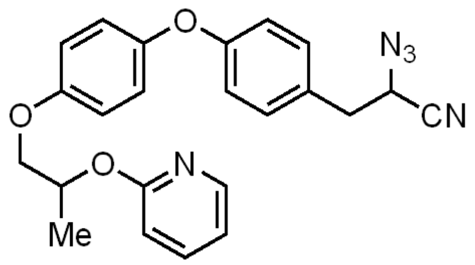


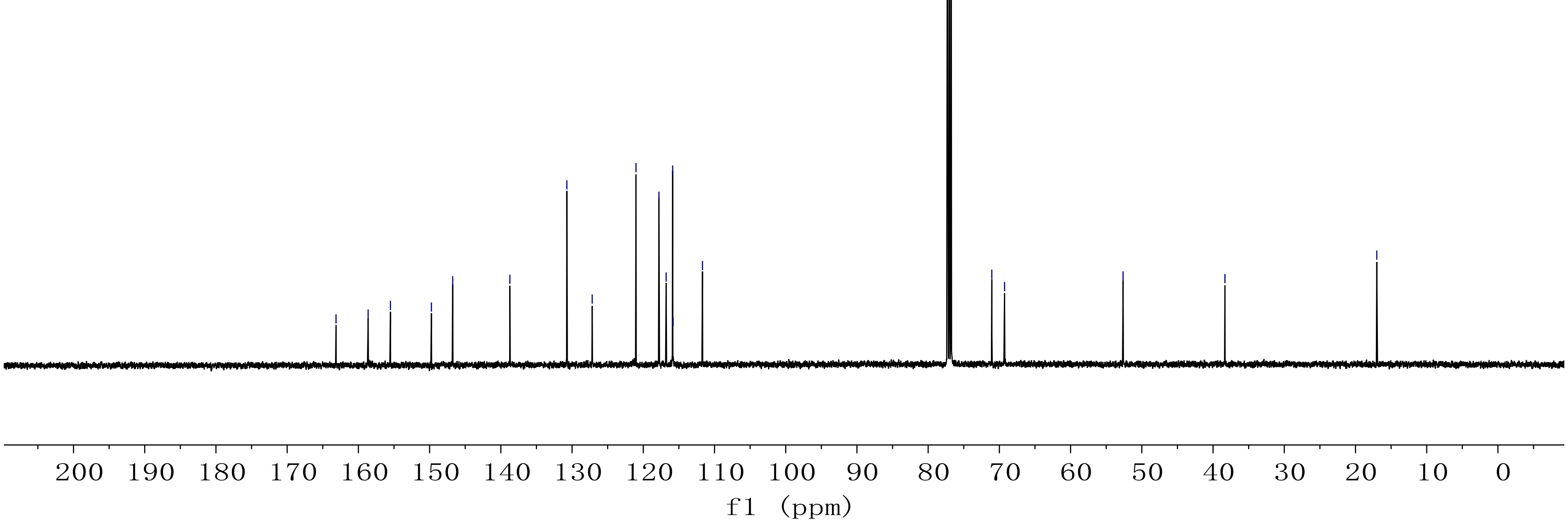

## $^{1}$H NMR of *rac*-fenofibrate-derived phenylalanine analogue 18

$CDCl_3$, 23 ºC

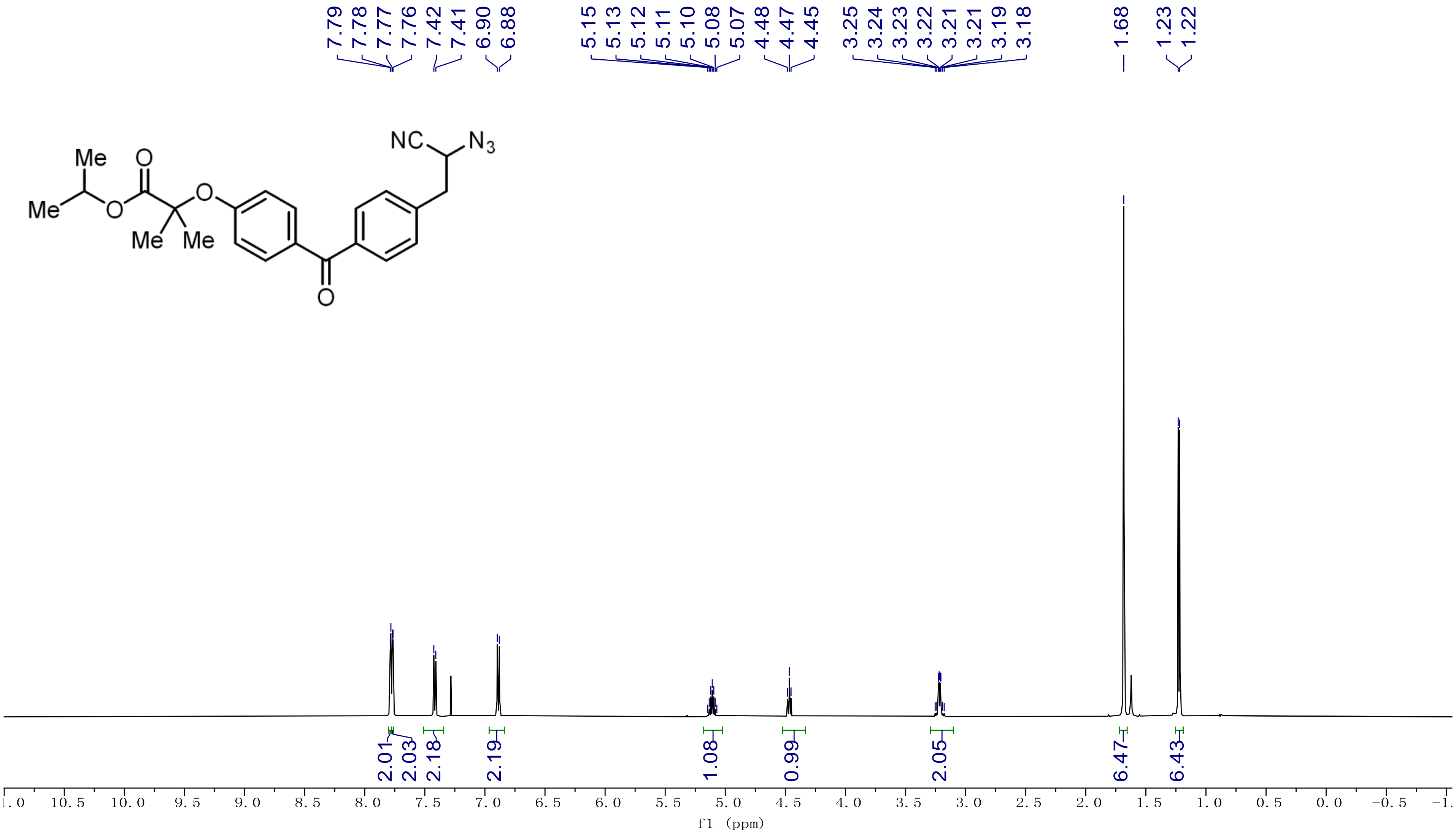

**$^{13}$C NMR of *rac*-fenofibrate-derived phenylalanine analogue 18**

$CDCl_3$, 23 ºC

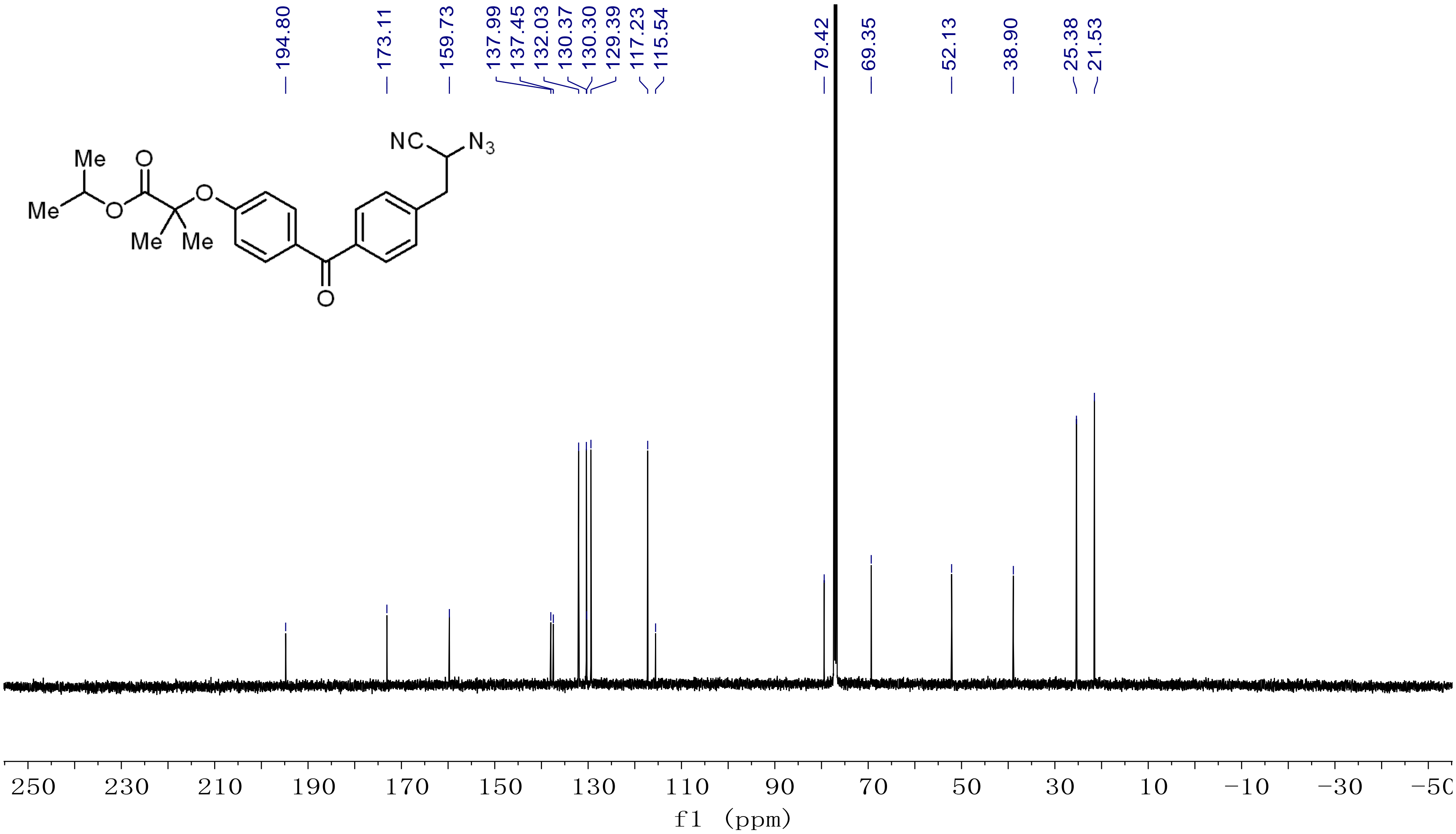

**$^{1}$H NMR of *rac*-thyronine precursor 19**

$CDCl_3$, 23 ºC

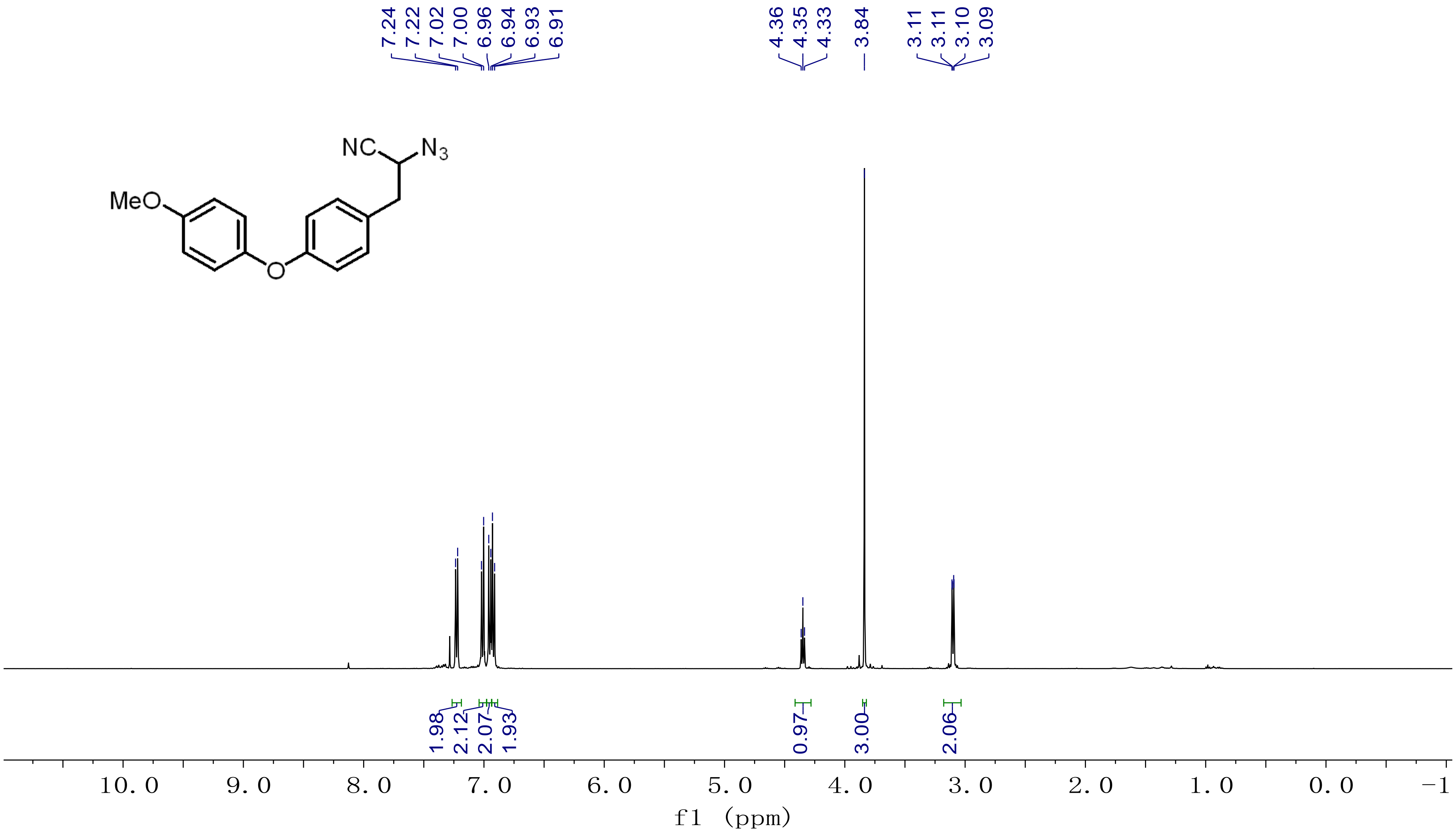

## $^{13}$C NMR of *rac*-thyronine precursor 19

$CDCl_3$, 23 ºC

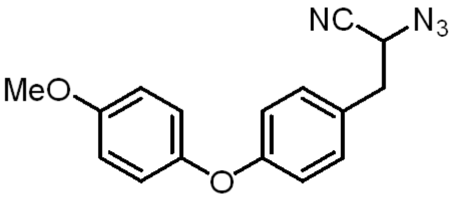


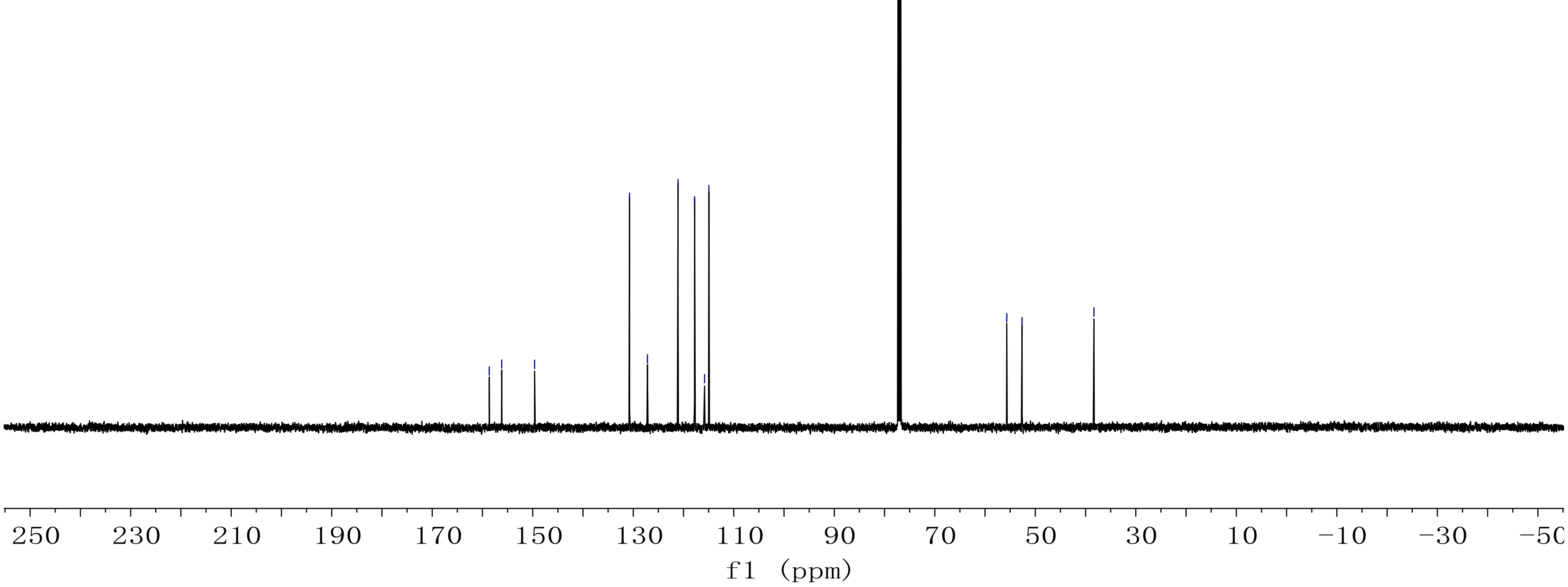

## $^{1}$H NMR of *rac*-dopa precursor 20

$CDCl_3$, 23 ºC

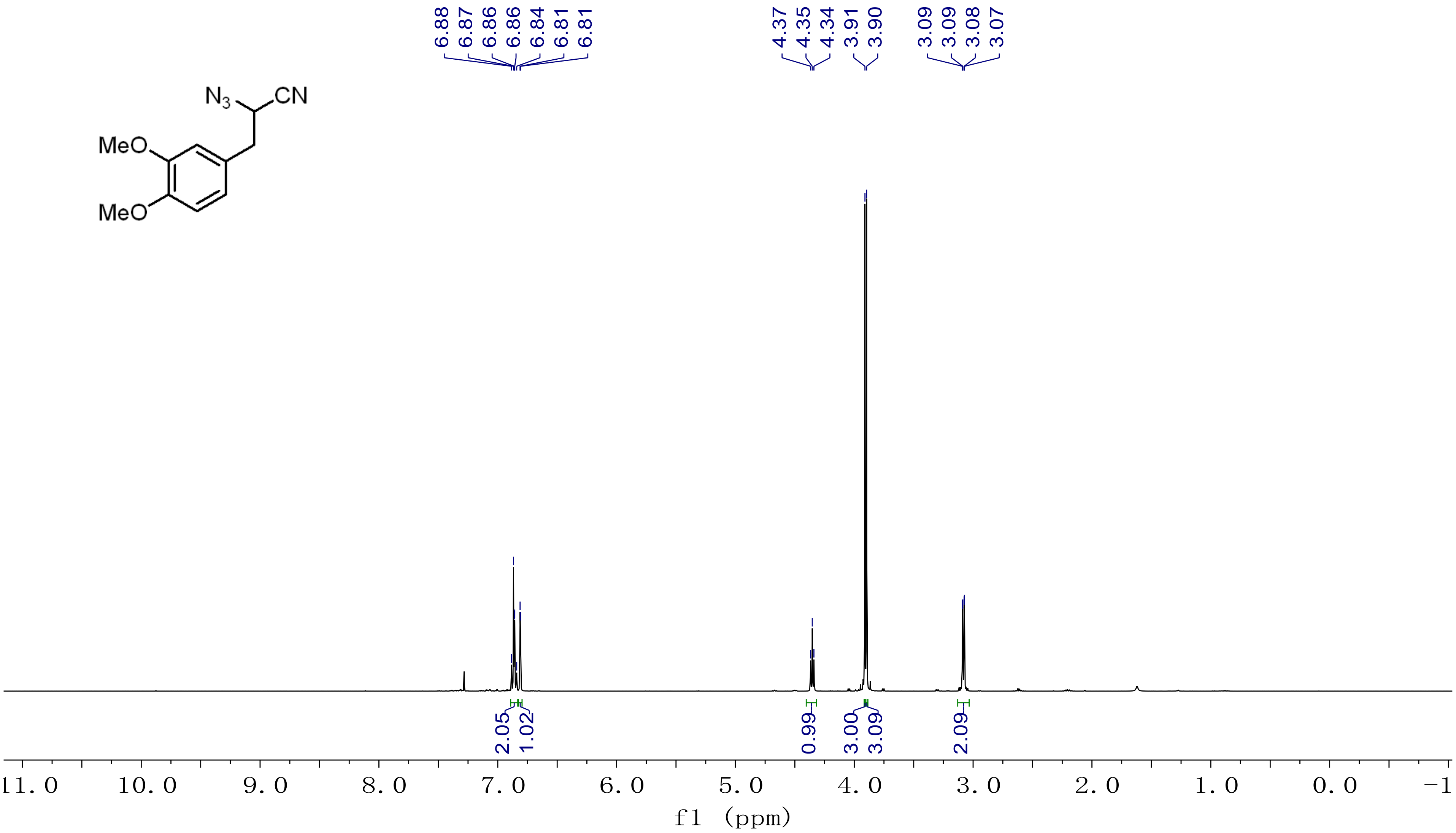

**$^{13}$C NMR of *rac*-dopa precursor 20**

$CDCl_3$, 23 ºC

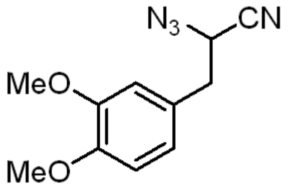


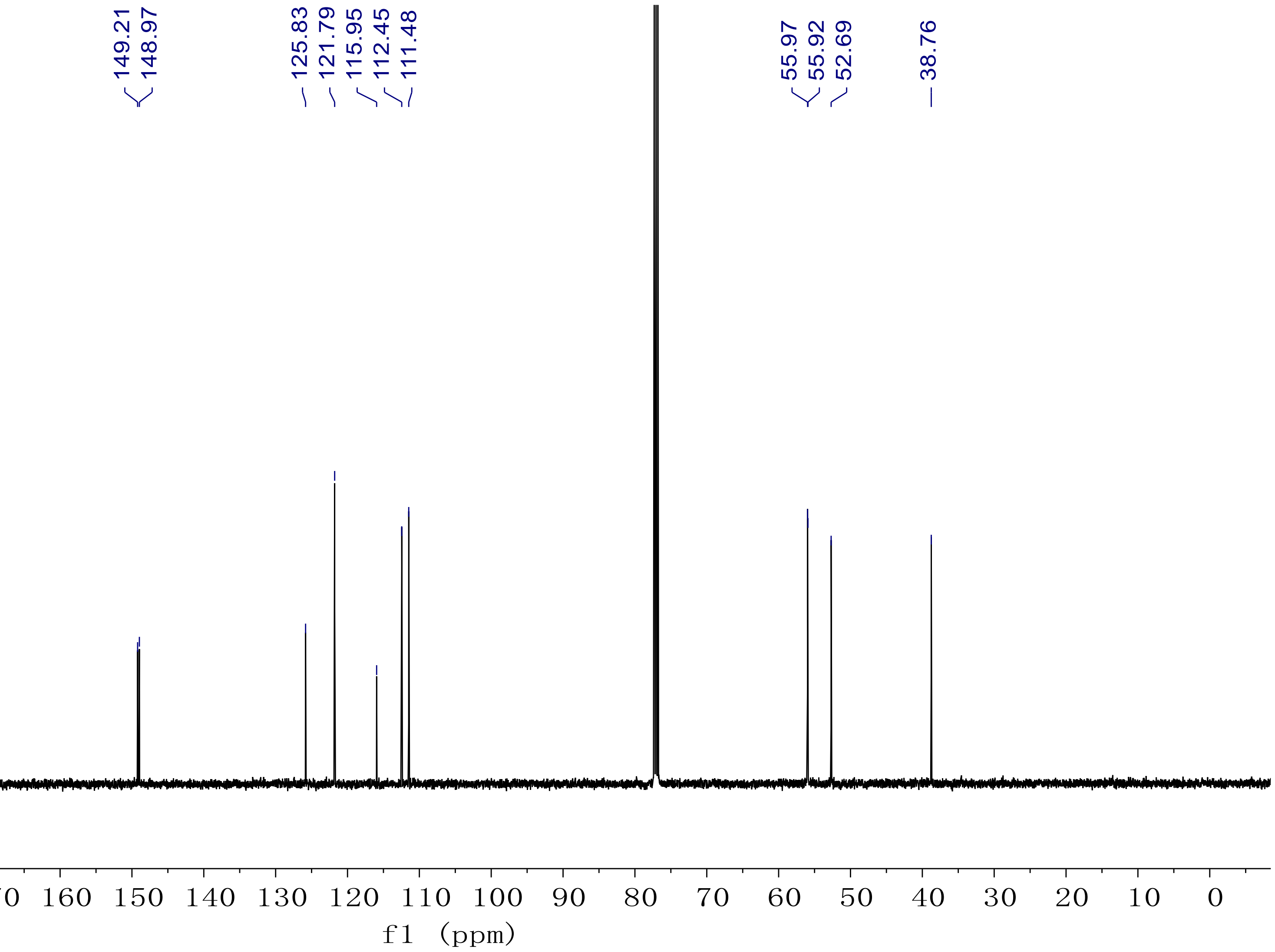

## $^1$H NMR of *rac*-xylariamide A precursor 21

CDCl$_3$, 23 ºC

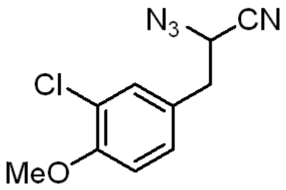

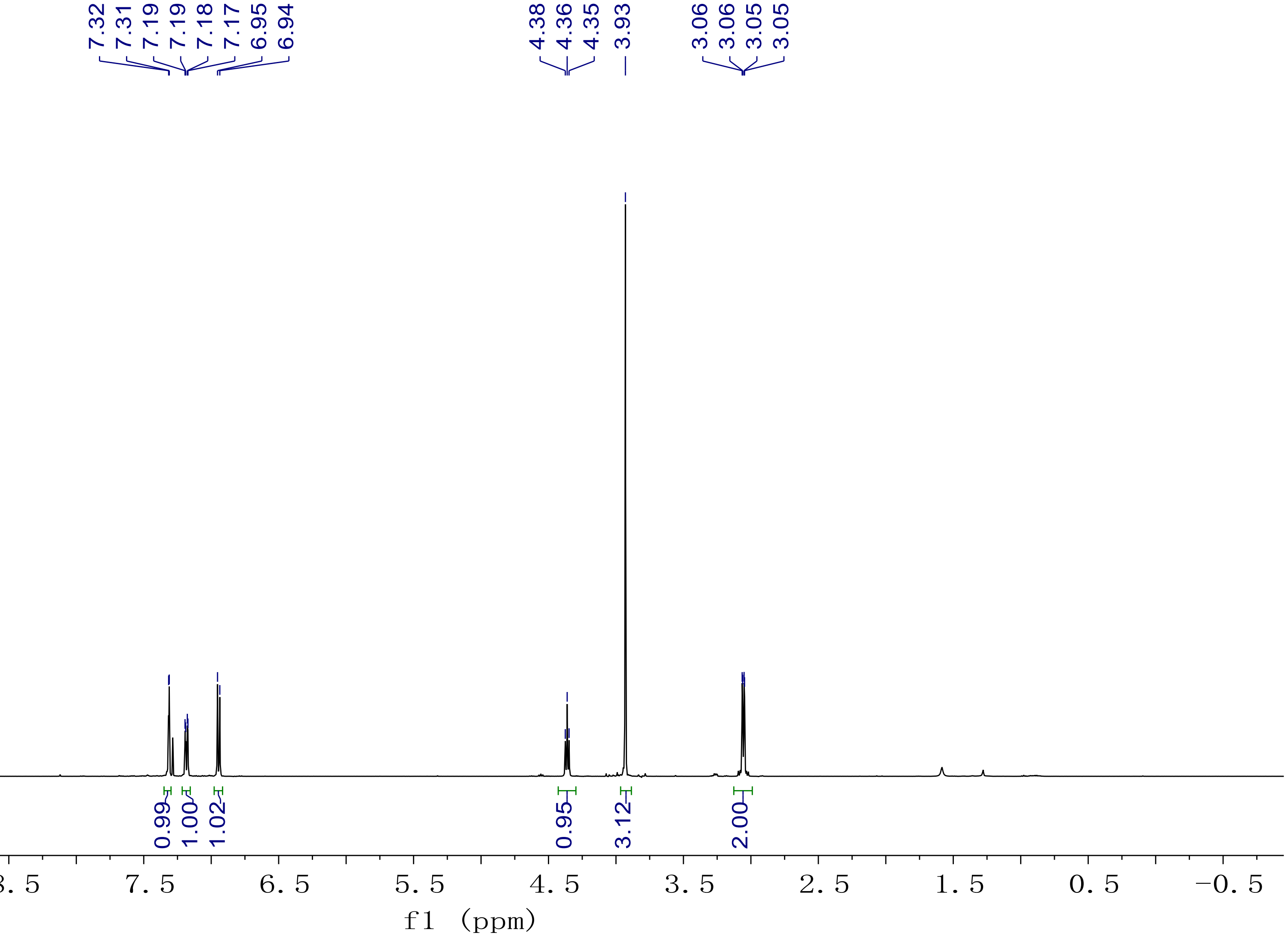

**$^{13}$C NMR of *rac*-xylariamide A precursor 21**

$CDCl_3$, 23 ºC

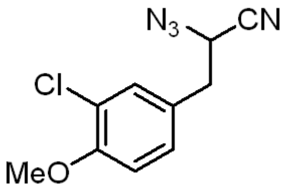

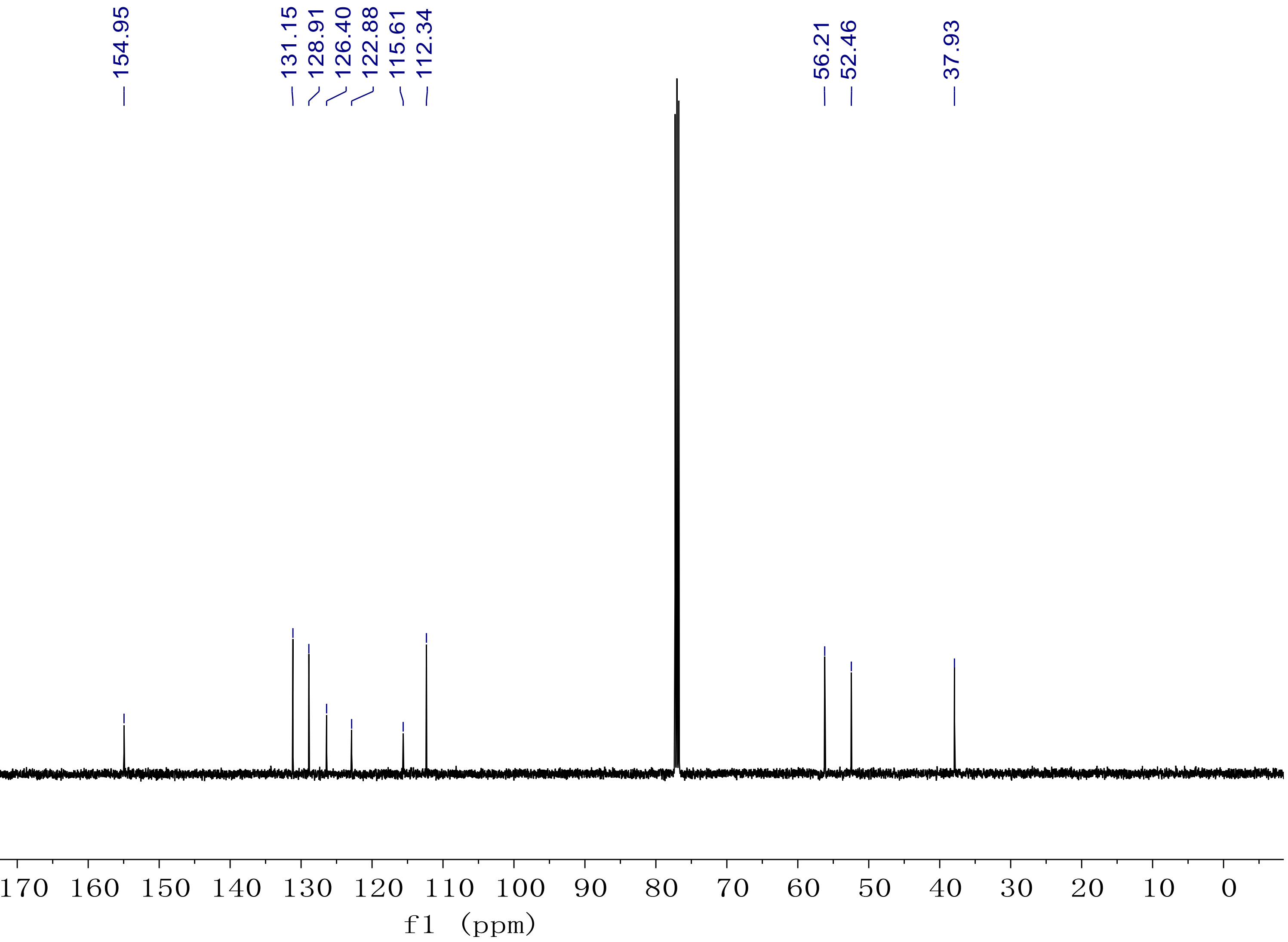

**$^{1}$H NMR of *rac*-fenclonine precursor 22**

$CDCl_3$, 23 ºC

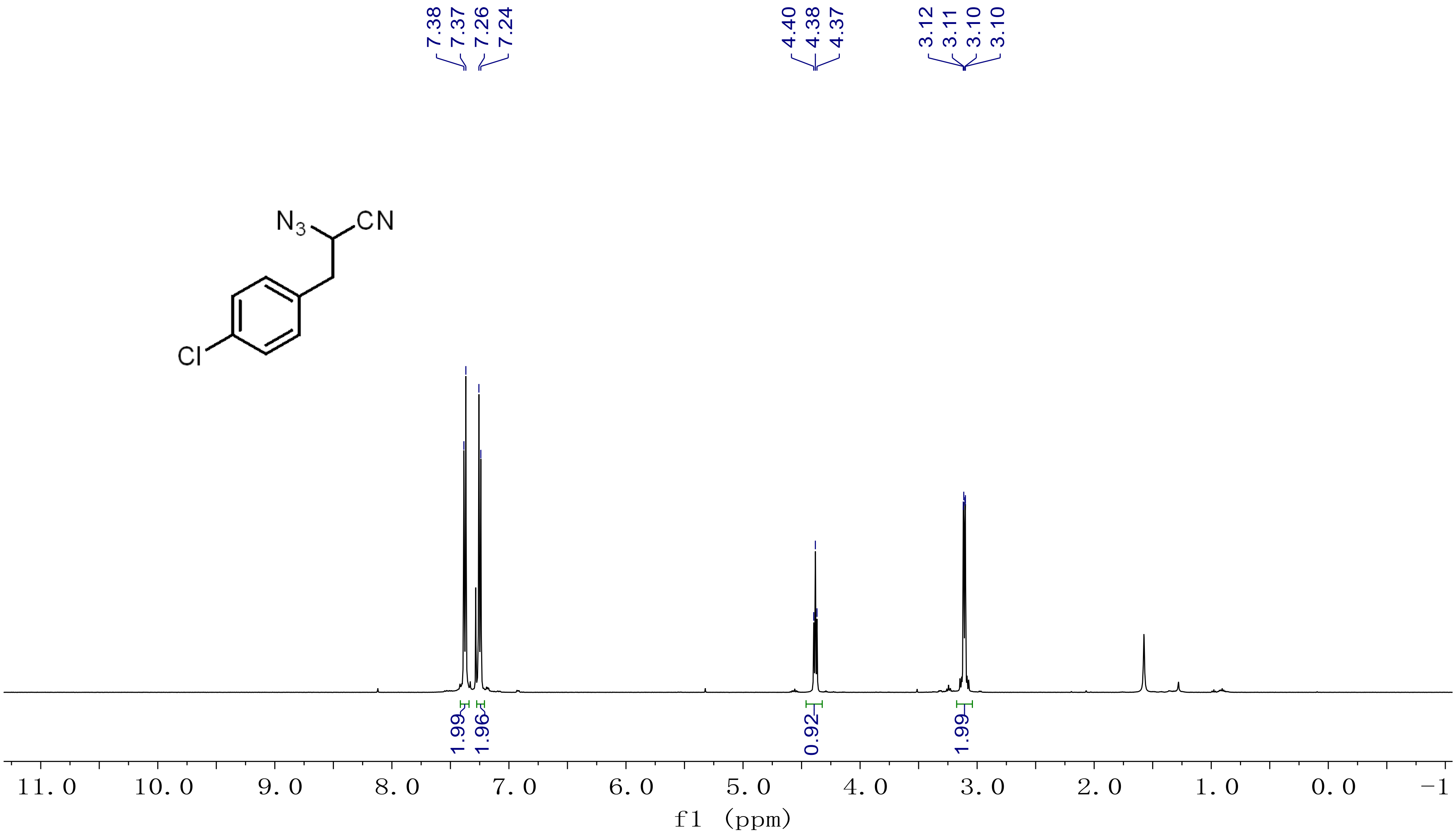

**$^{13}$C NMR of *rac*-fenclonine precursor 22**

CDCl$_3$, 23 ºC

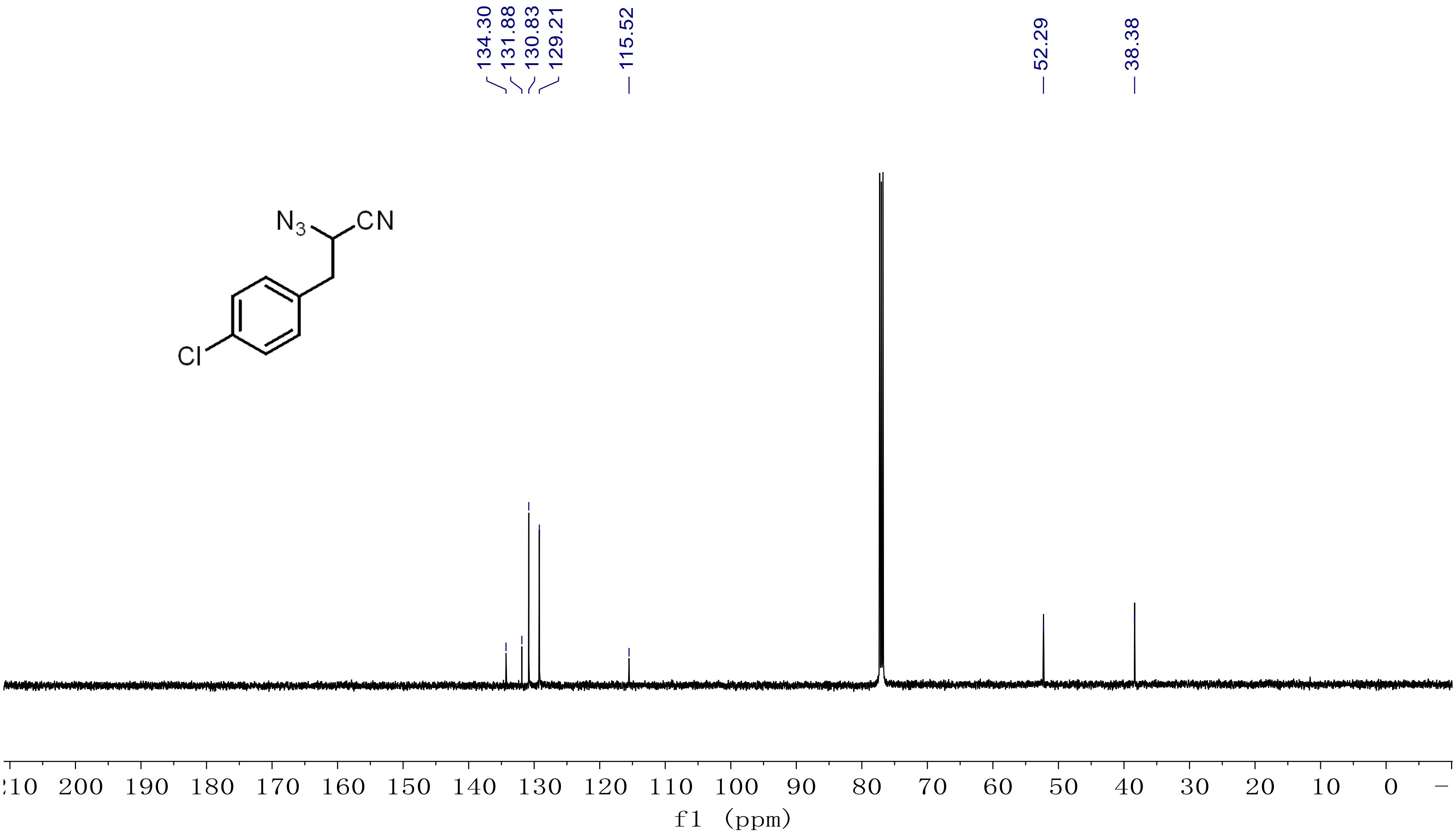

**$^1$H NMR of *rac*-phenylalanine analogue 23**

$CDCl_3$, 23 ºC

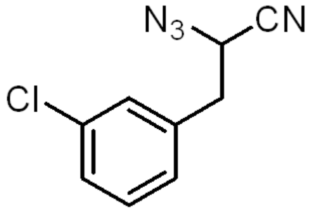

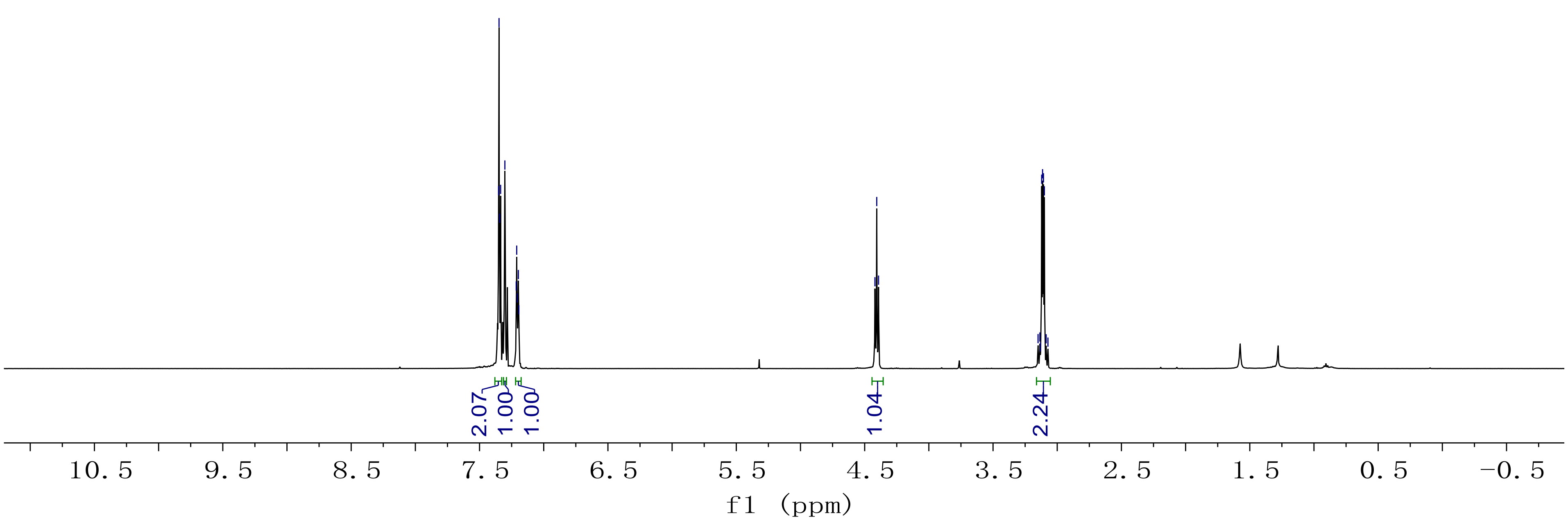

**$^{13}$C NMR of *rac*-phenylalanine analogue 23**

$CDCl_3$, 23 ºC

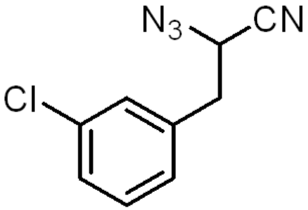

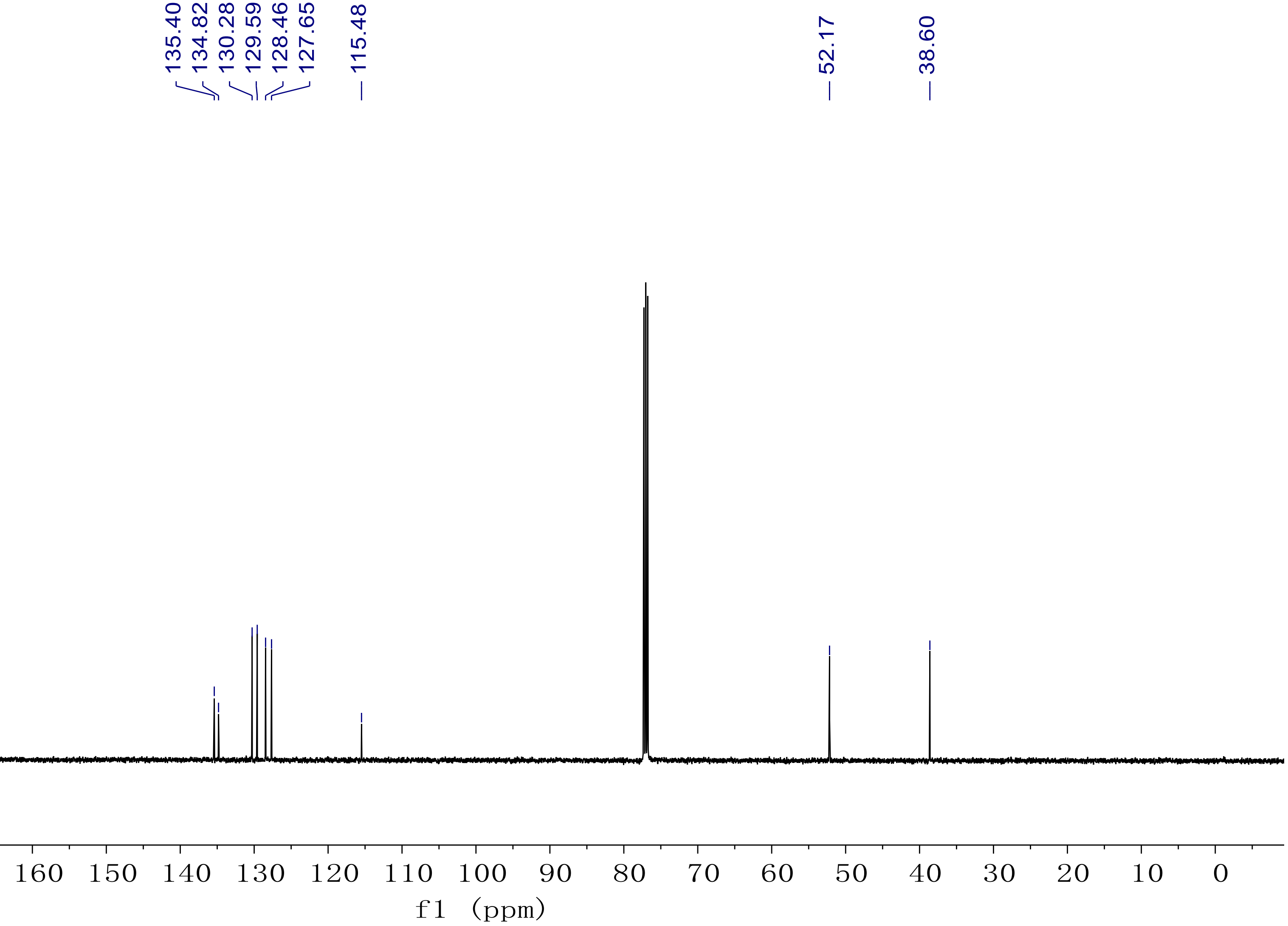

**$^{1}$H NMR of *rac*-phenylalanine analogue 24**

$CDCl_3$, 23 ºC

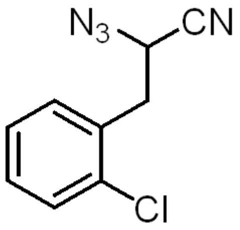


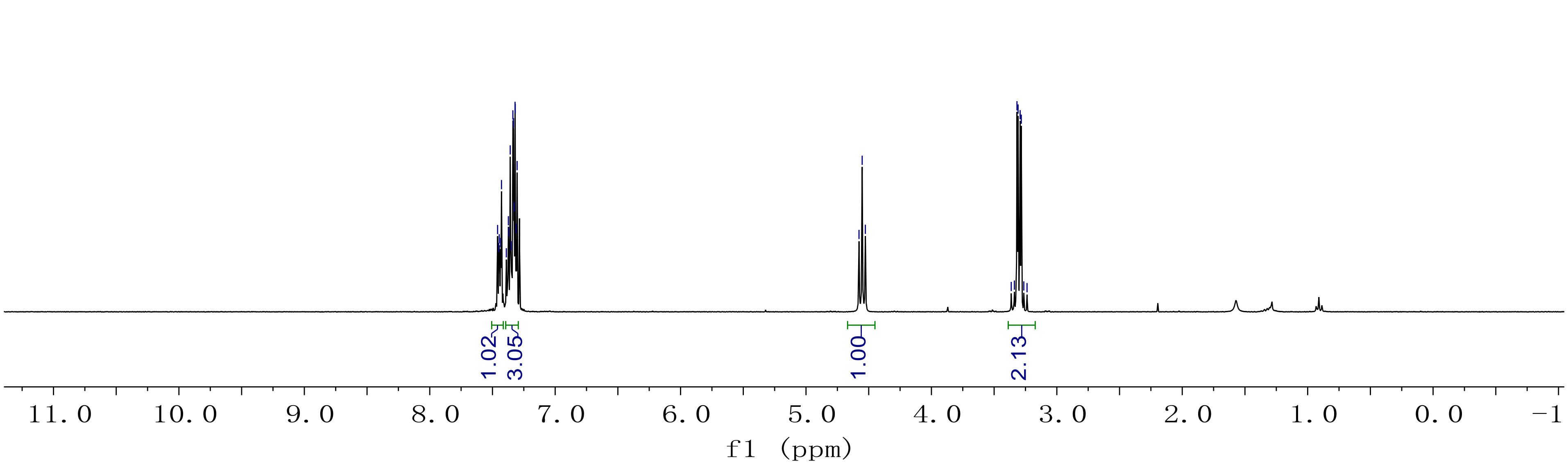

**$^{13}$C NMR of *rac*-phenylalanine analogue 24**

CDCl$_3$, 23 ºC

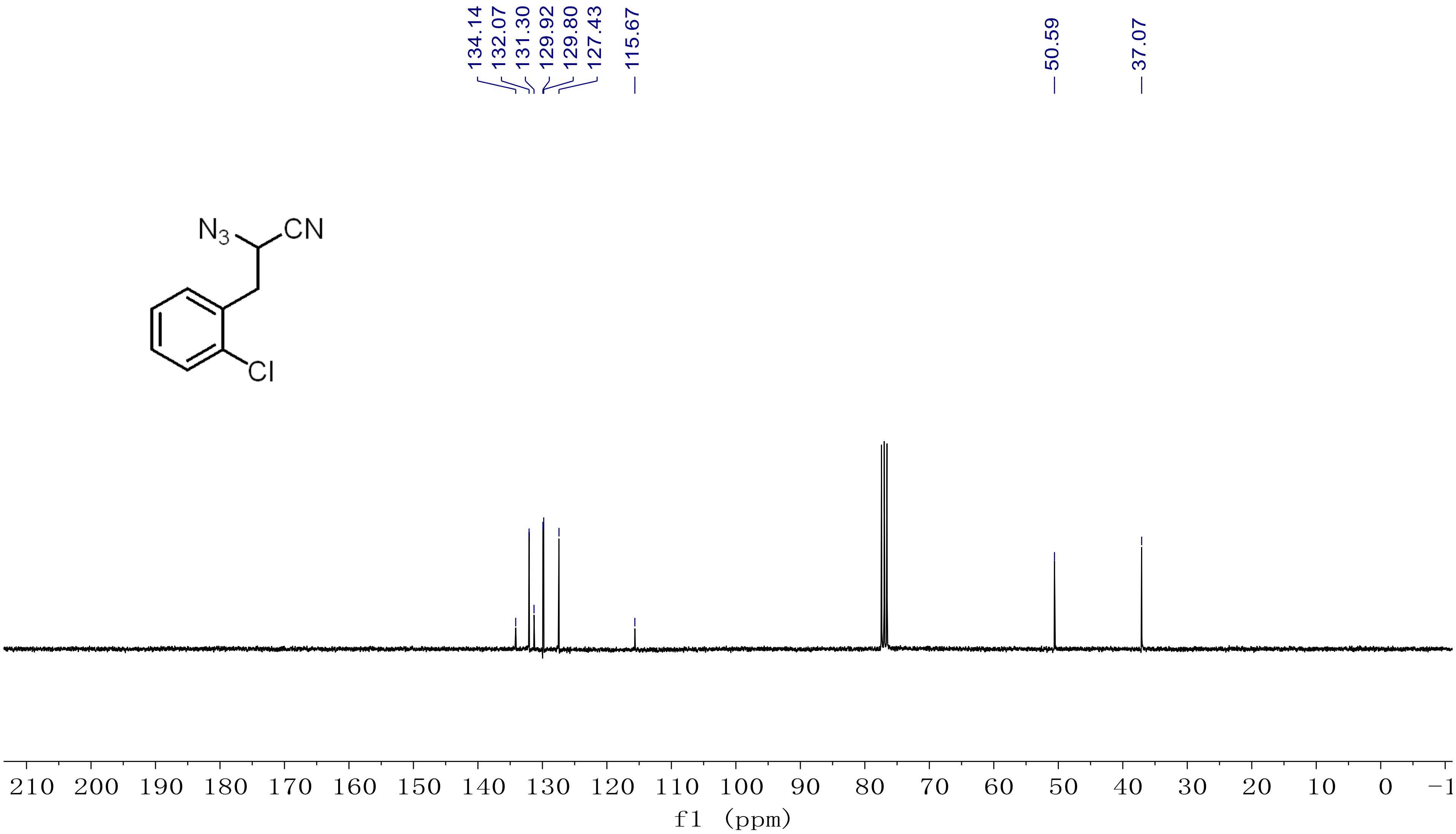

## $^{1}$H NMR of *rac*-thianaphthene alanine analogue 25

CDCl3, 23 ºC

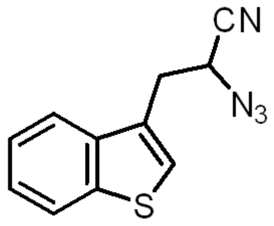

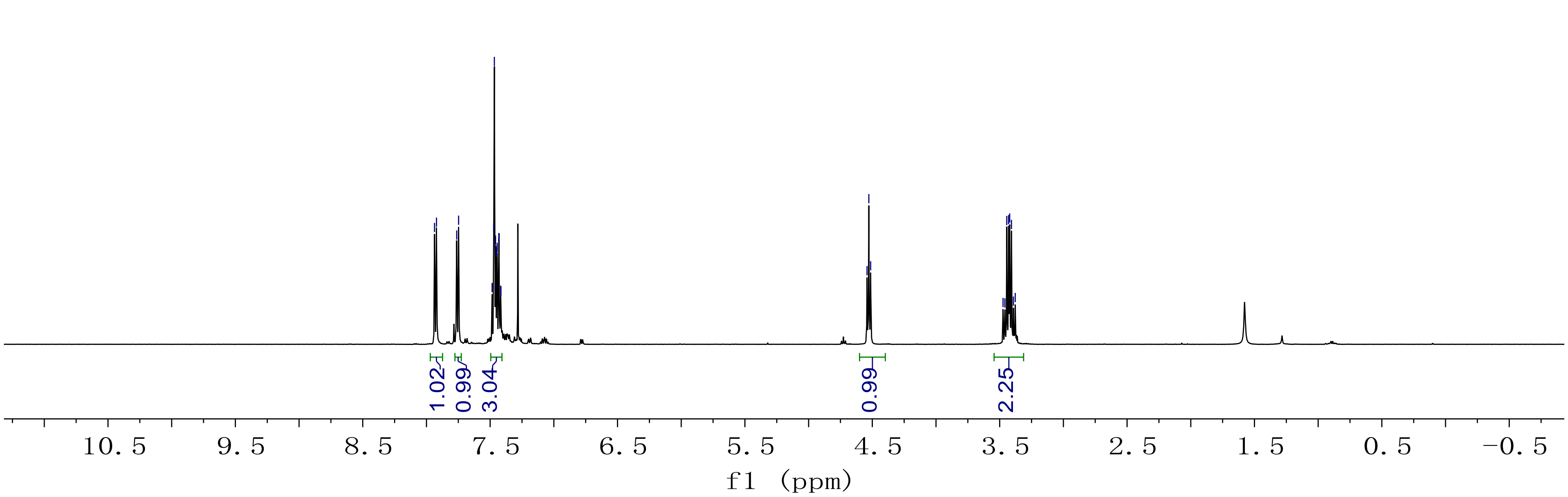

**$^{13}$C NMR of *rac*-thianaphthene alanine analogue 25**

$CDCl_3$, 23 ºC

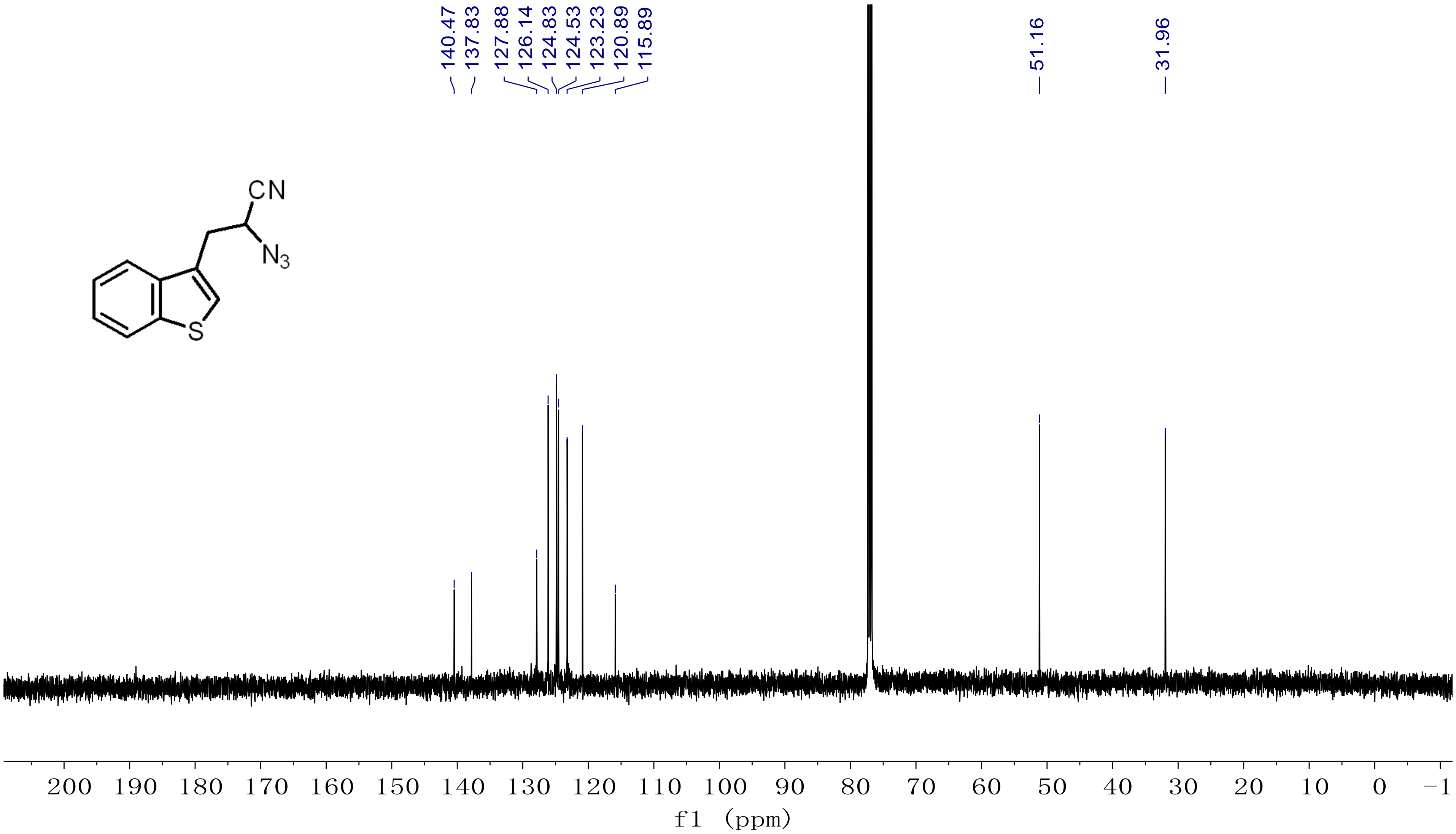

**$^{1}$H NMR of *rac*-pyrrole alanine analogue 26**

CDCl3, 23 ºC

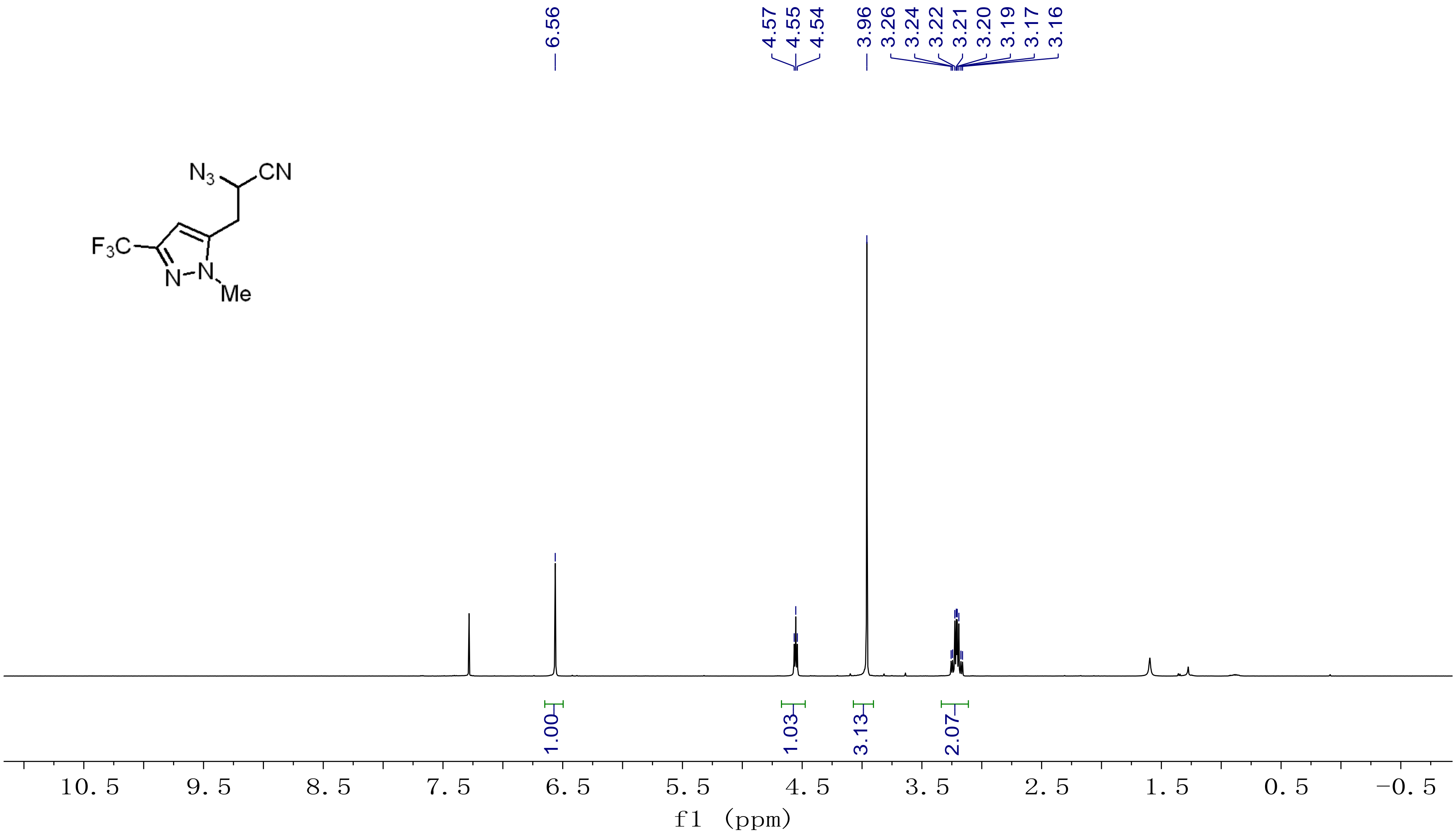

**$^{19}$F NMR of *rac*-pyrrole alanine analogue 26**

$CDCl_3$, 23 ºC

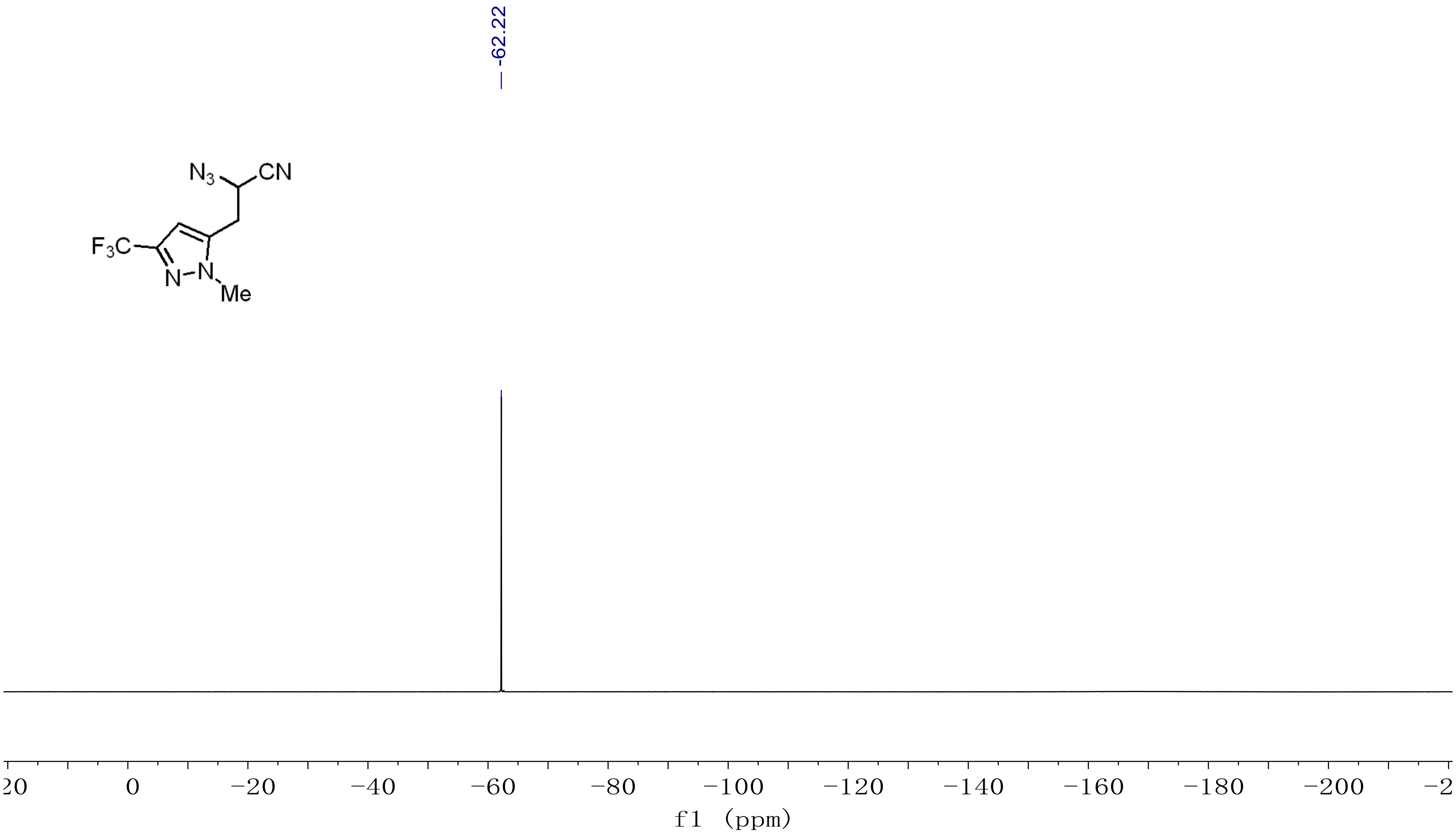

**$^{13}$C NMR of *rac*-pyrrole alanine analogue 26**

CDCl$_3$, 23 ºC

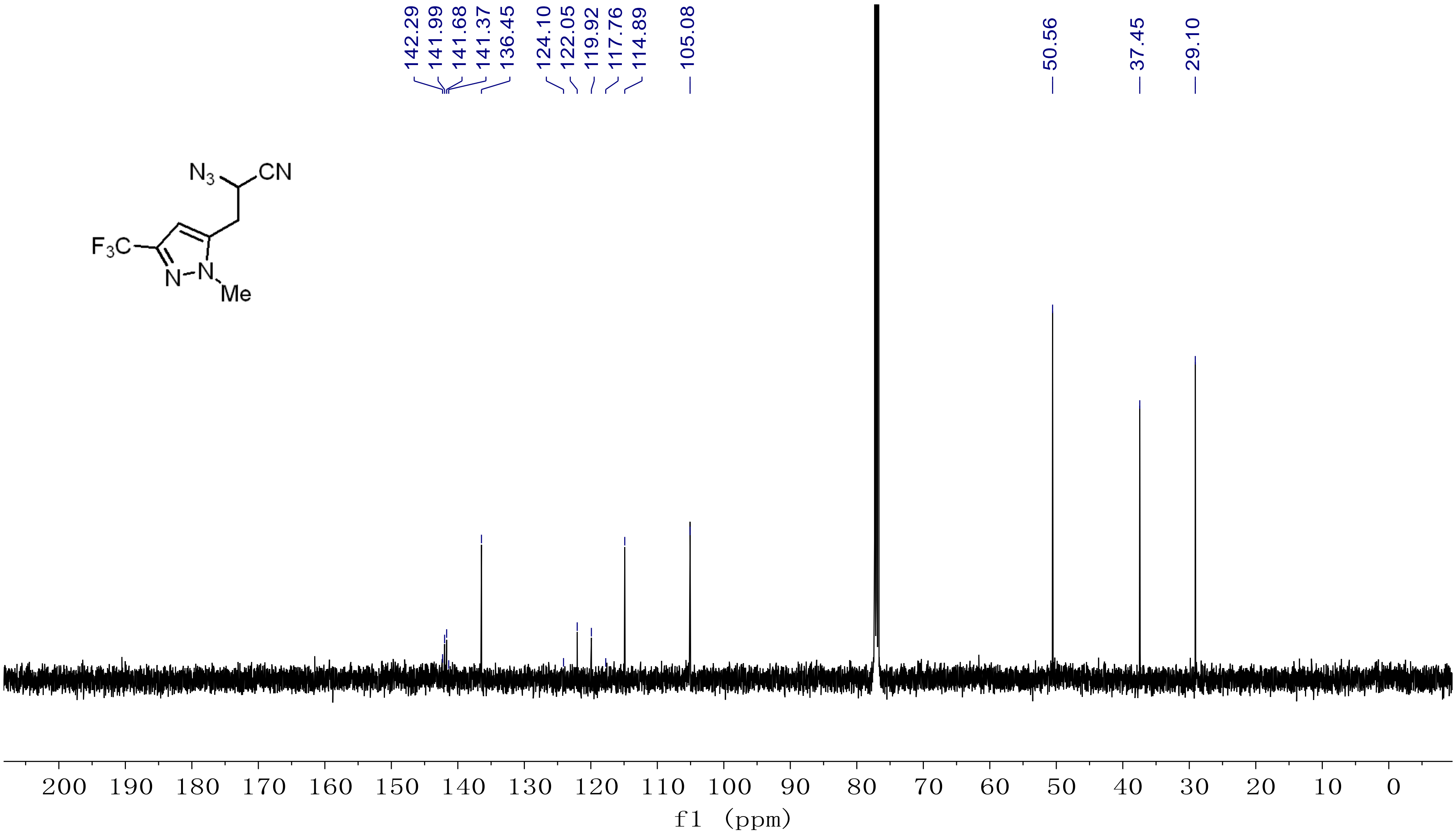

**$^{1}$H NMR of *rac*-isoquinoline alanine analogue 27**

$C_6D_6$, 23 ºC

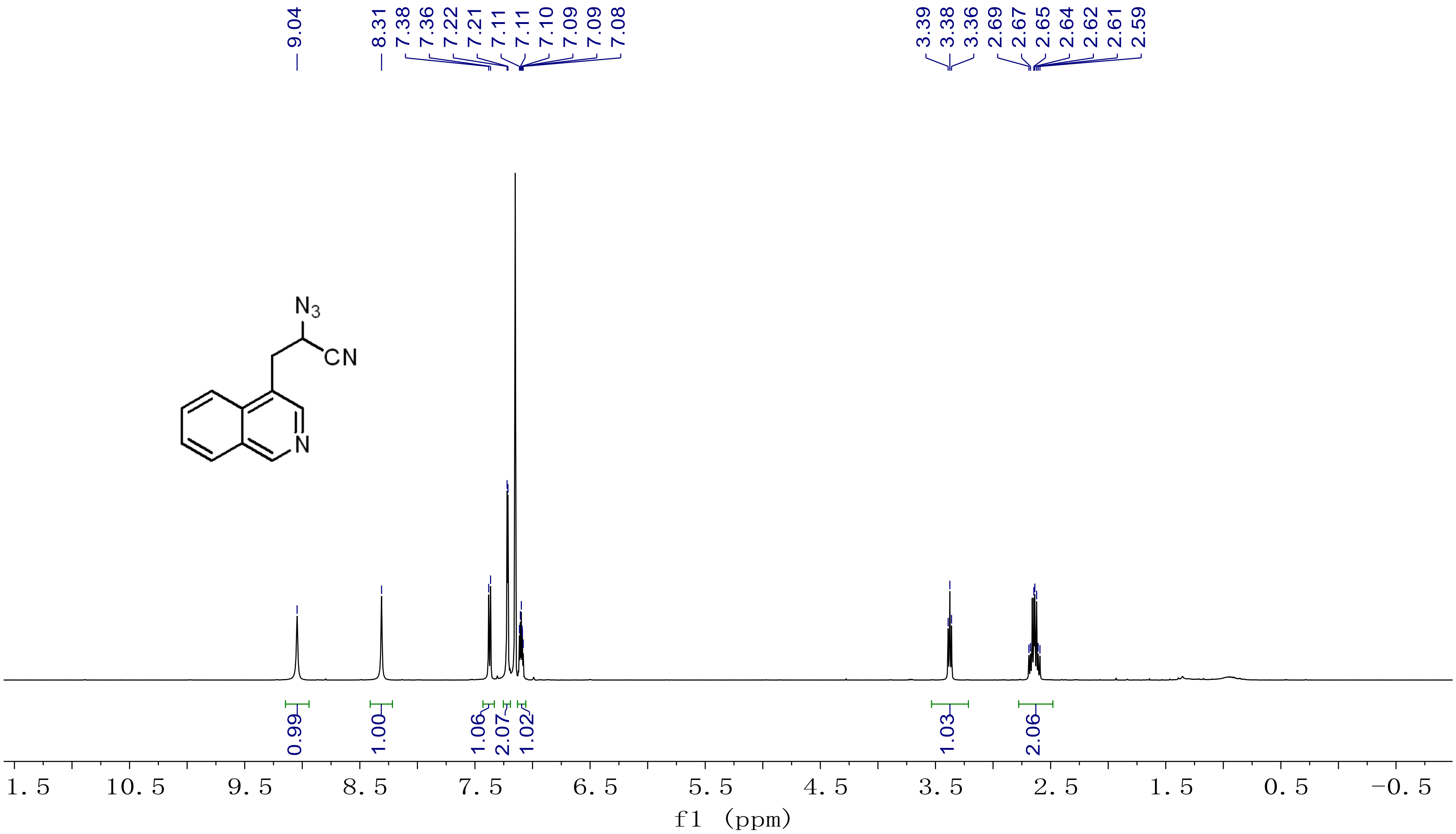

**$^{13}$C NMR of *rac*-isoquinoline alanine analogue 27**

$C_6D_6$, 23 ºC

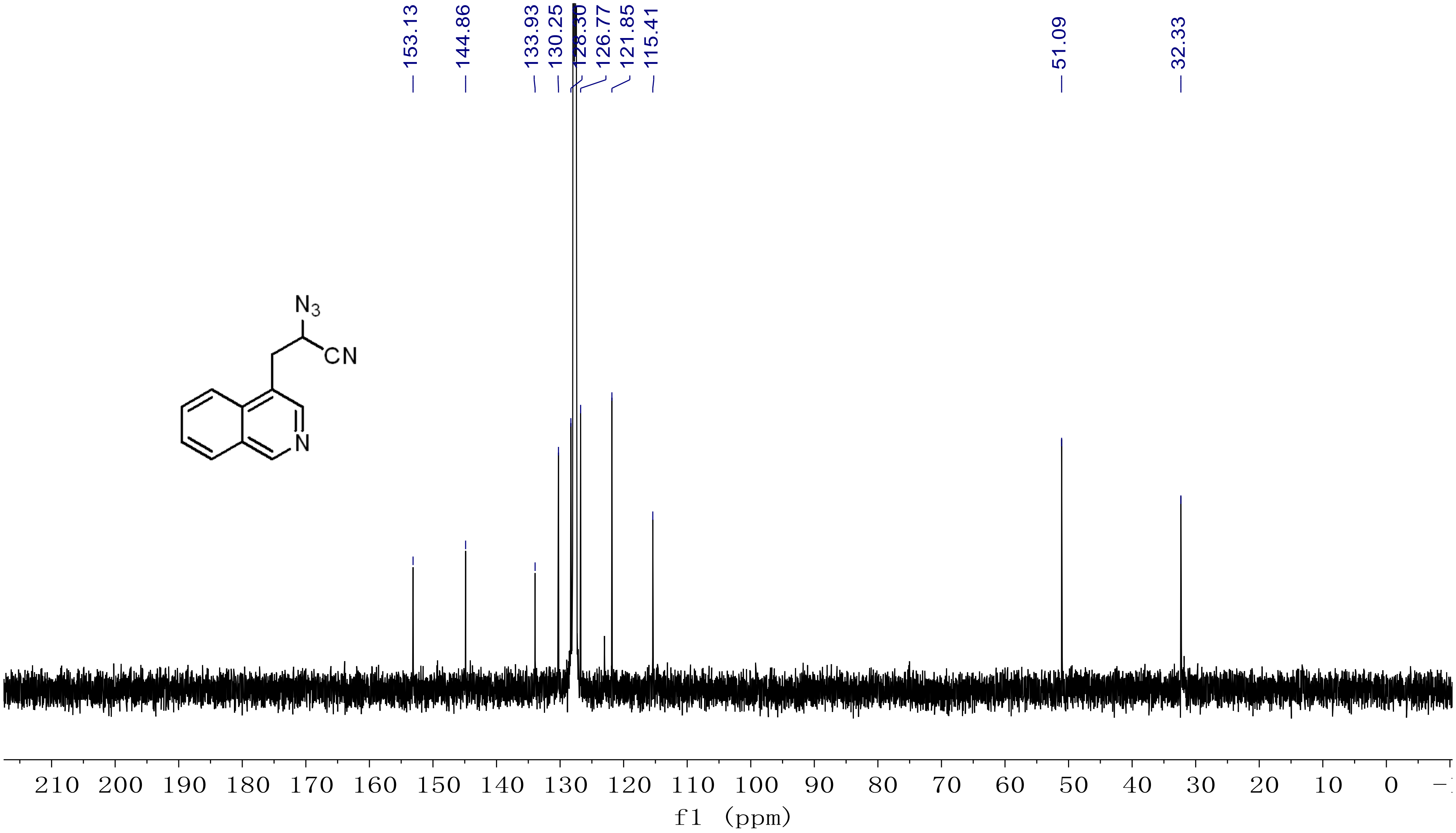

## $^{1}$H NMR of *rac*-quinoline alanine analogue 28

$CDCl_3$, 23 ºC

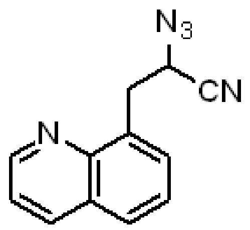


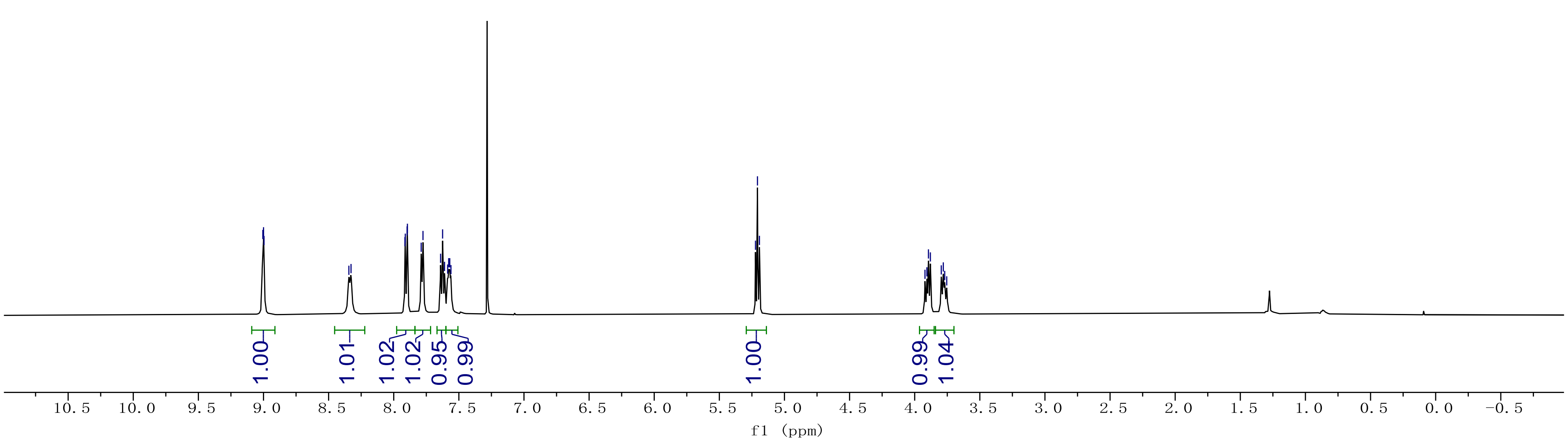

**$^{13}$C NMR of *rac*-quinoline alanine analogue 28**

$CDCl_3$, 23 ºC

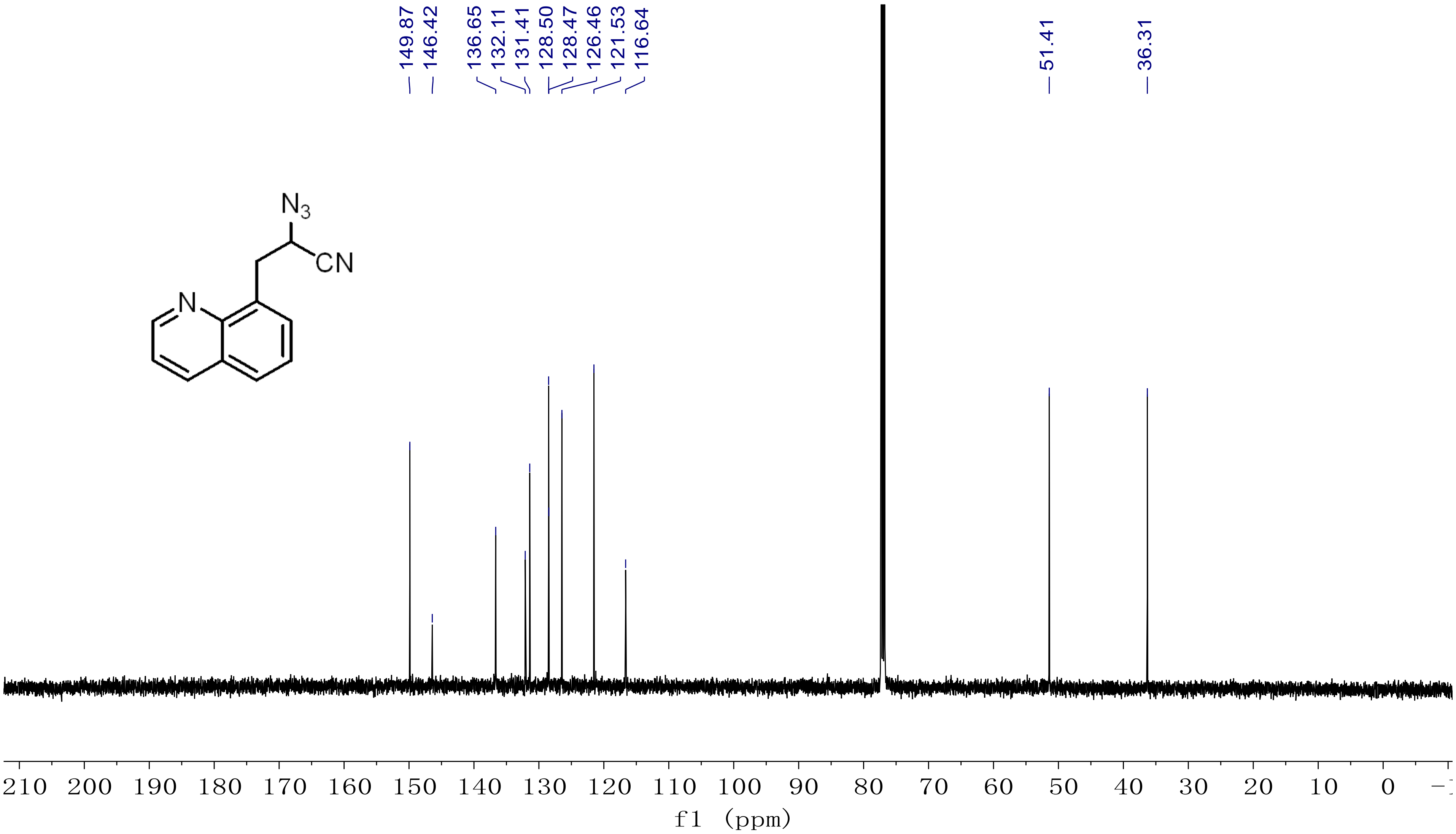

**$^{1}$H NMR of *rac*-pyridine alanine analogue 29**

$CDCl_3$, 23 ºC

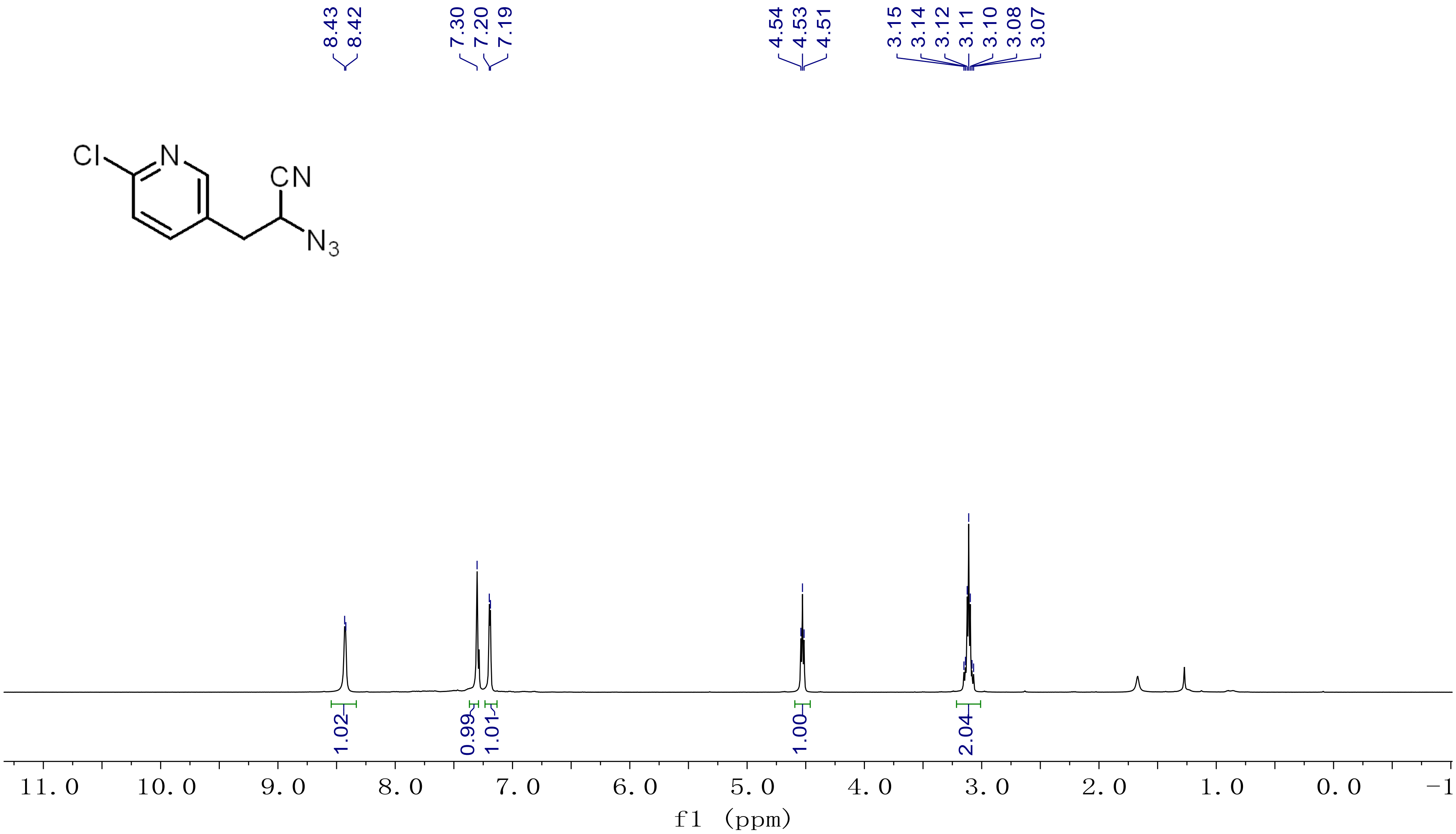

**$^{13}$C NMR of *rac*-pyridine alanine analogue 29**

CDCl$_3$, 23 °C

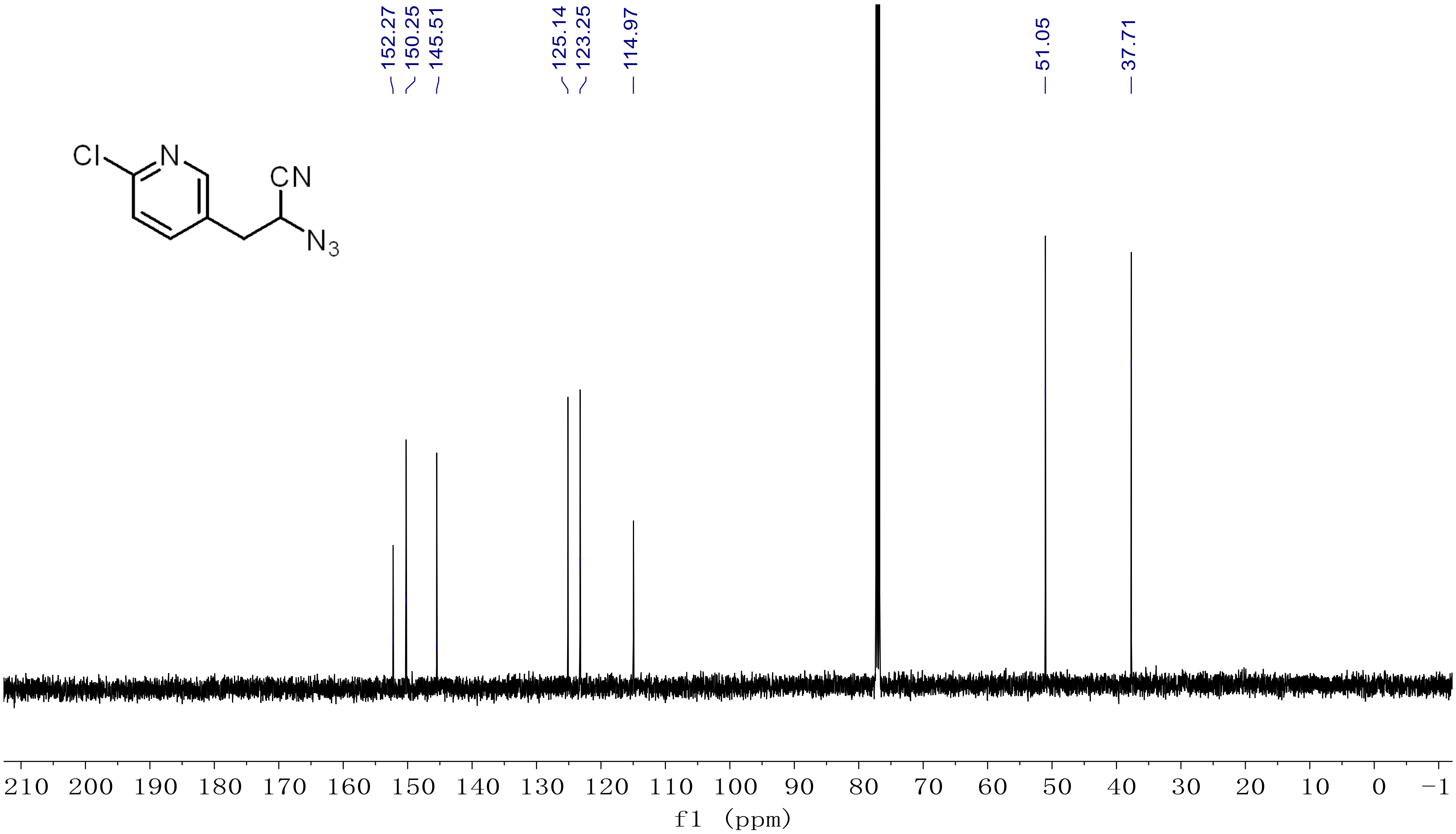

**$^{1}$H NMR of flubiprofen-derived phenylethylazide 30**

$CDCl_3$, 23 ºC

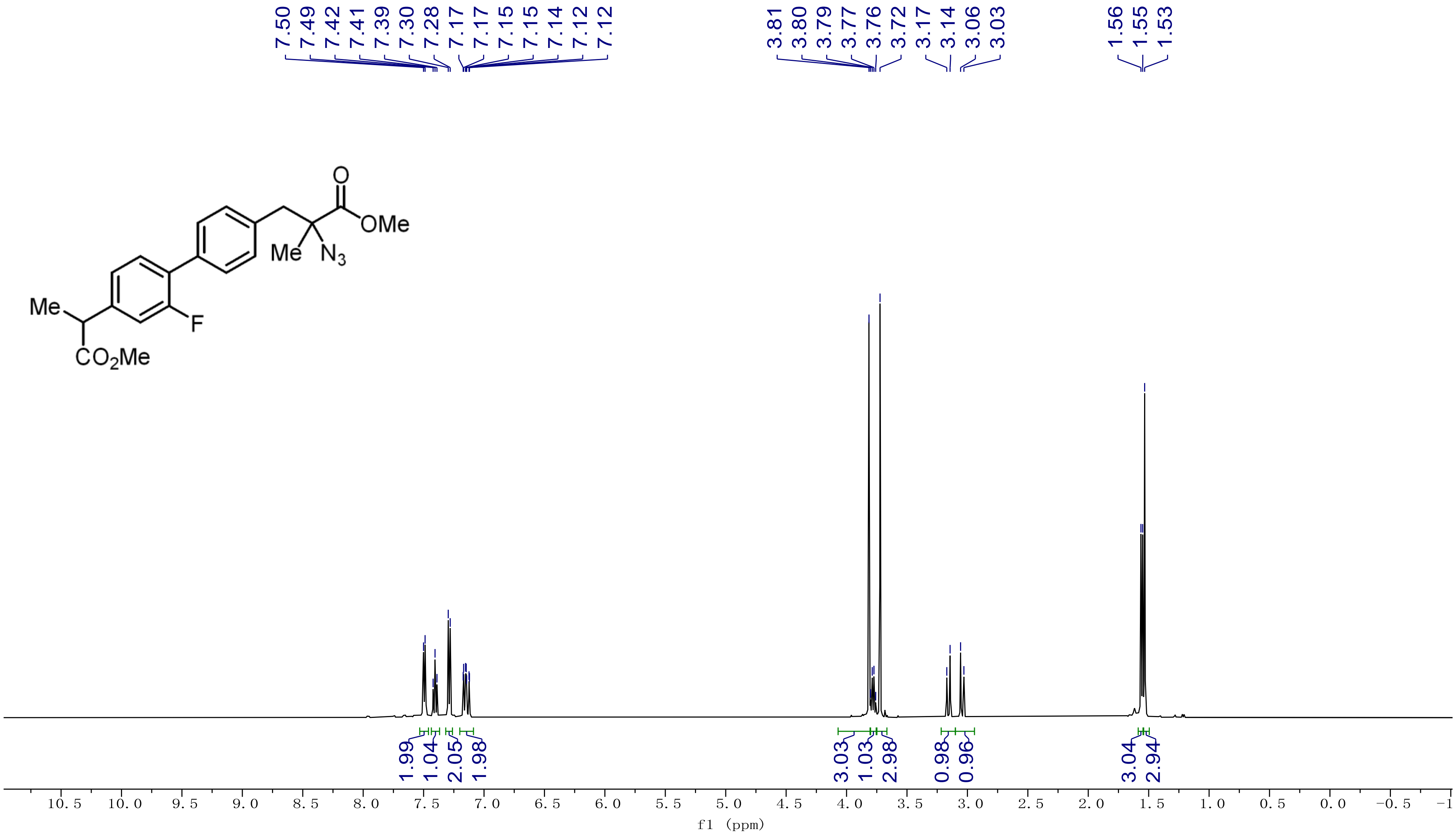

## $^{19}$F NMR of flubiprofen-derived phenylethylazide 30

CDCl$_3$, 23 ºC

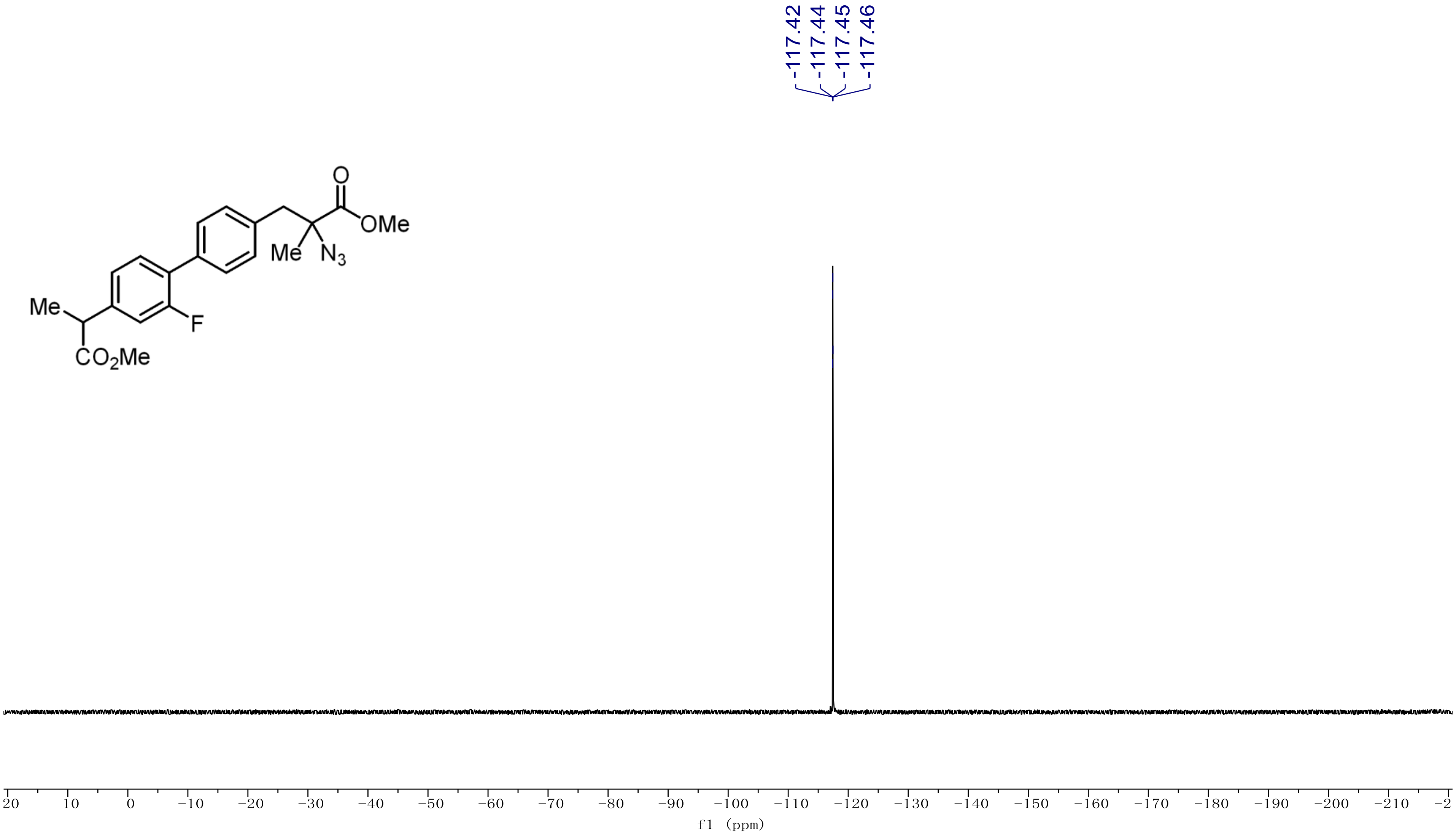

**$^{13}$C NMR of flubiprofen-derived phenylethylazide 30**

$CDCl_3$, 23 ºC

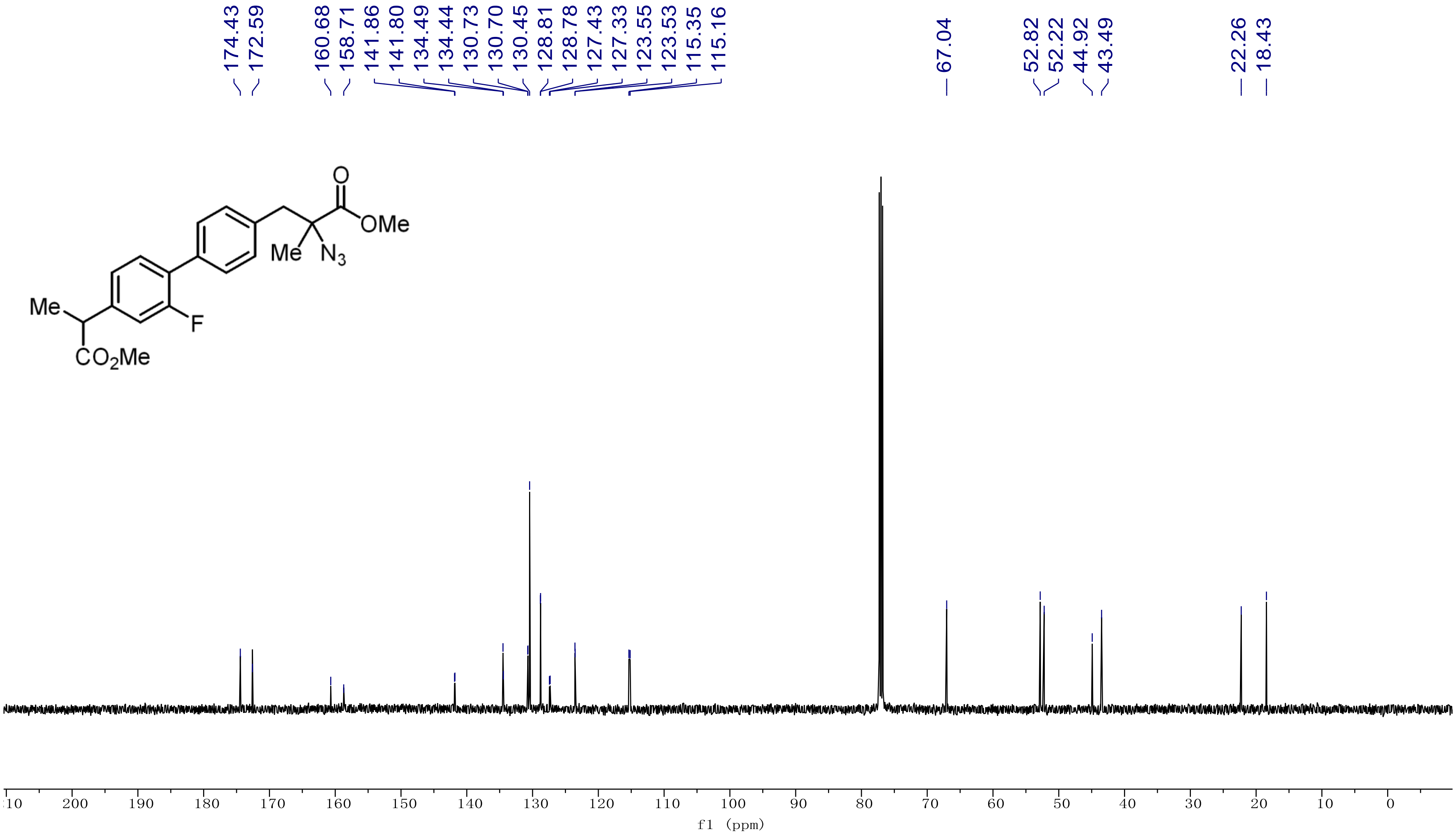

**$^{1}$H NMR of flubiprofen-derived phenylethylazide 31**

CDCl$_3$, 23 ºC

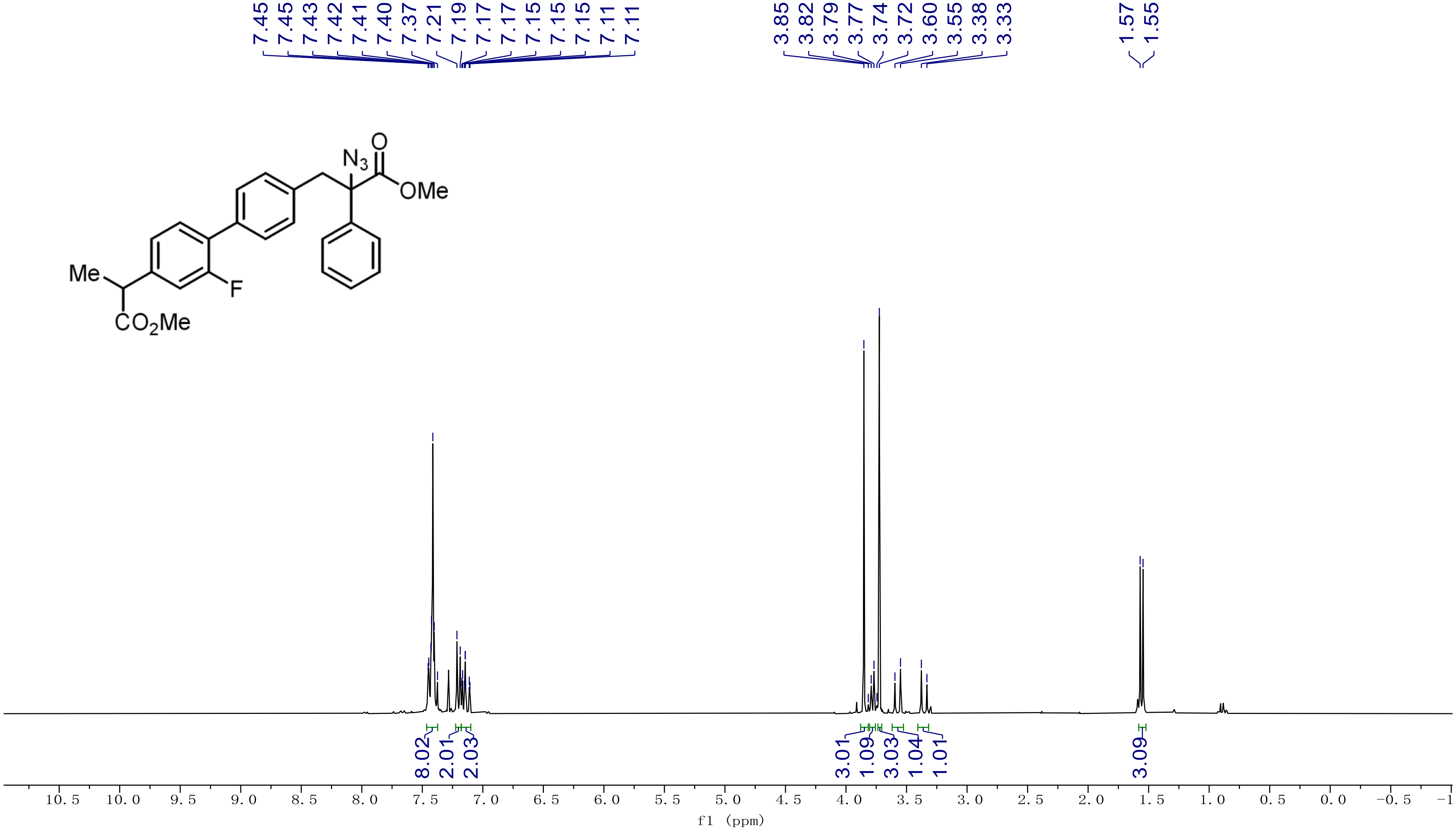

**$^{19}$F NMR of flubiprofen-derived phenylethylazide 31**

$CDCl_3$, 23 ºC

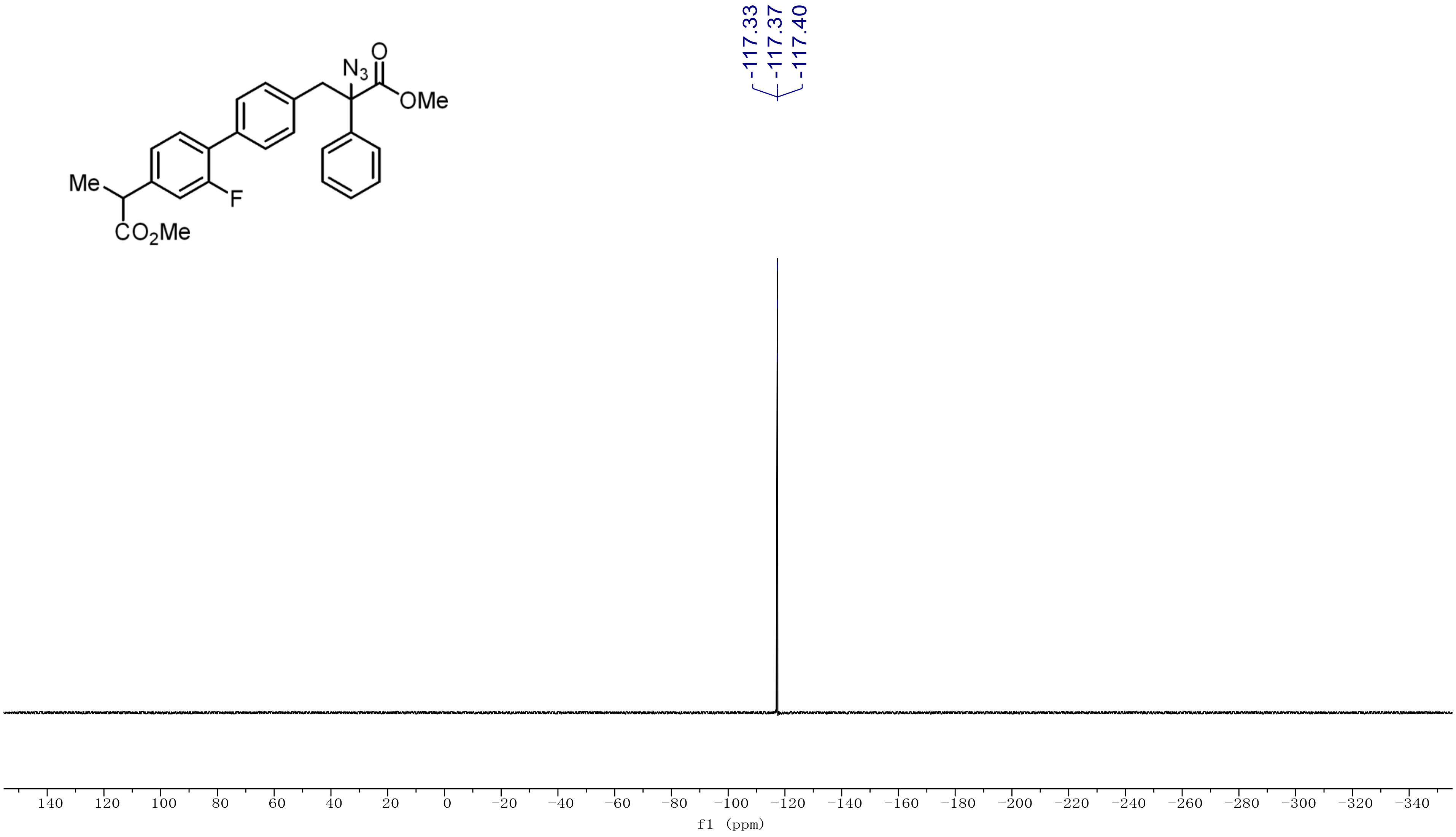

**$^{13}$C NMR of flubiprofen-derived phenylethylazide 31**

$CDCl_3$, 23 ºC

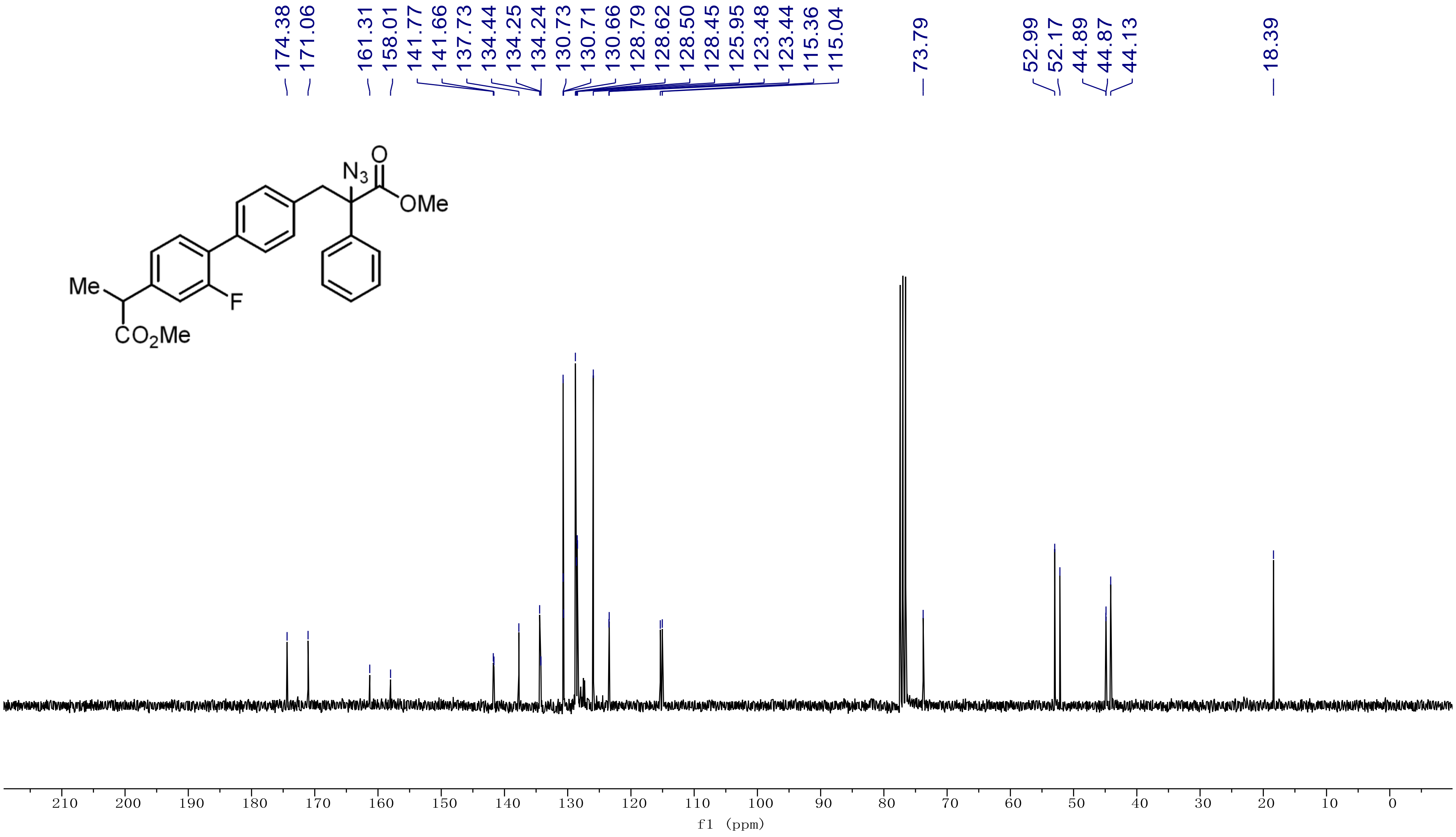

**$^{1}$H NMR of flubiprofen-derived phenylethylazide 32**

$CDCl_3$, 23 ºC

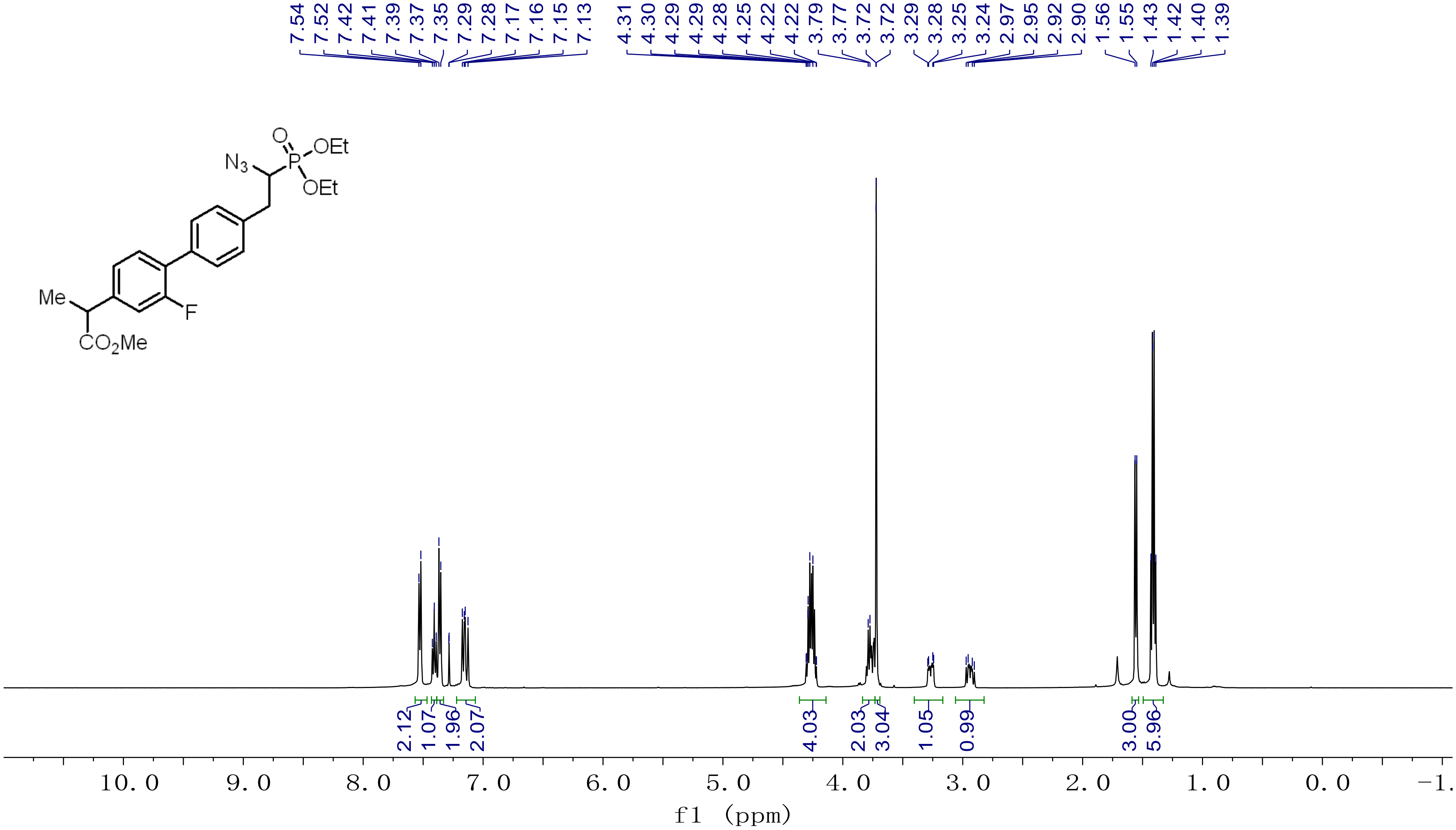

**$^{19}$F NMR of flubiprofen-derived phenylethylazide 32**

$CDCl_3$, 23 ºC

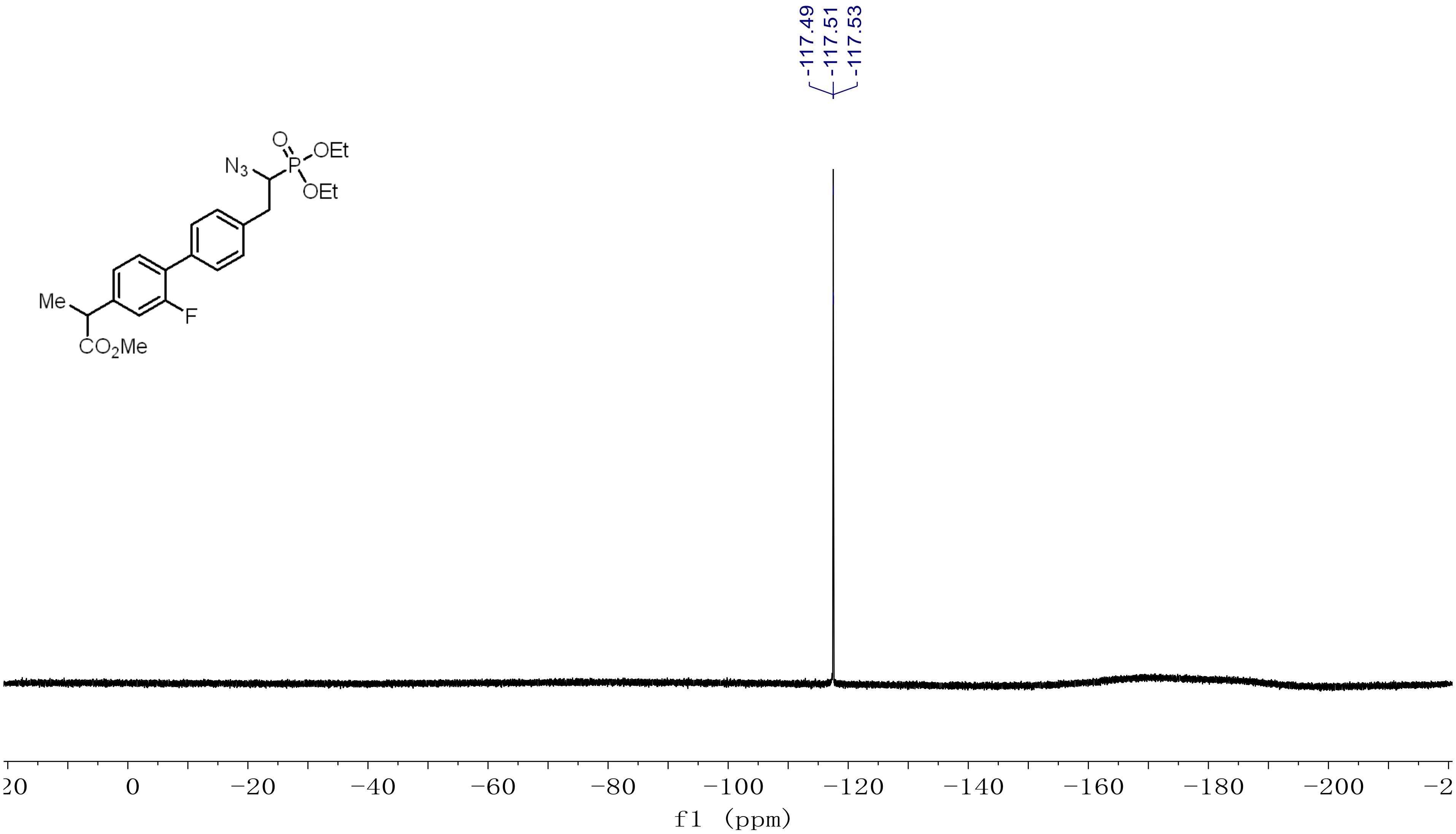

**$^{31}$P NMR of flubiprofen-derived phenylethylazide 32**

$CDCl_3$, 23 ºC

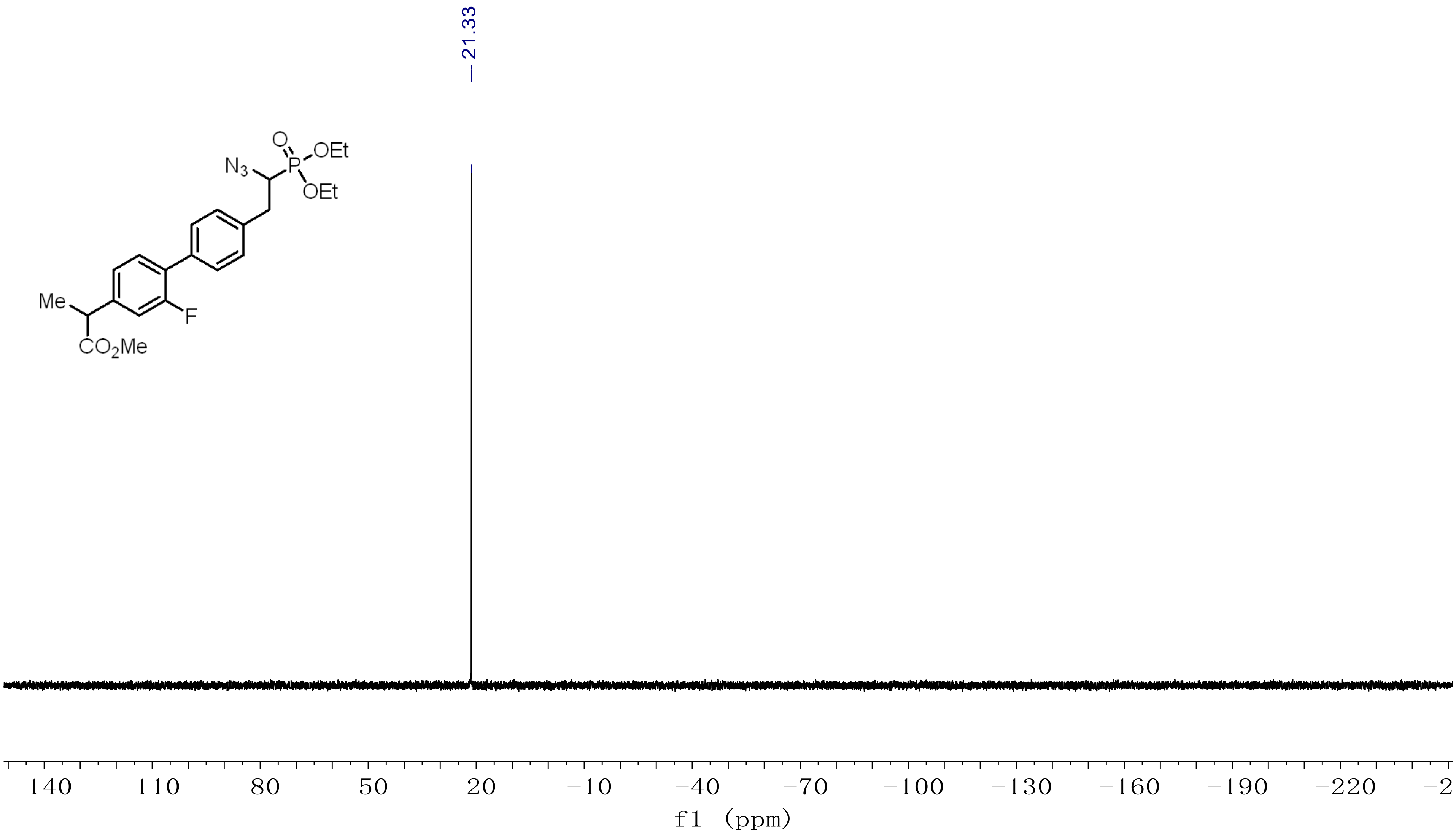

**$^{13}$C NMR of flubiprofen-derived phenylethylazide 32**

$CDCl_3$, 23 ºC

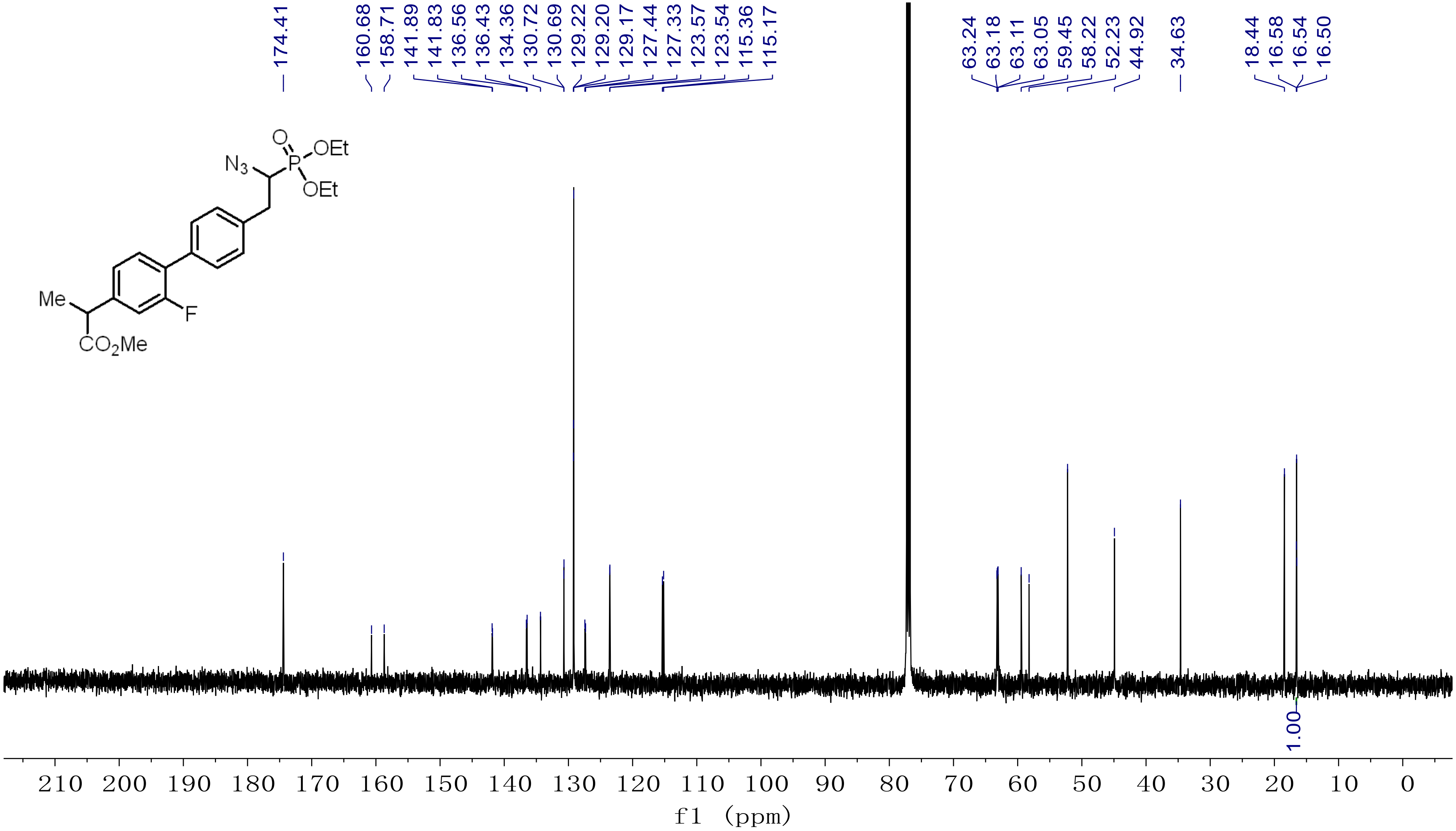

**$^{1}$H NMR of flubiprofen-derived phenylethylazide 33**

$CDCl_3$, 23 ºC

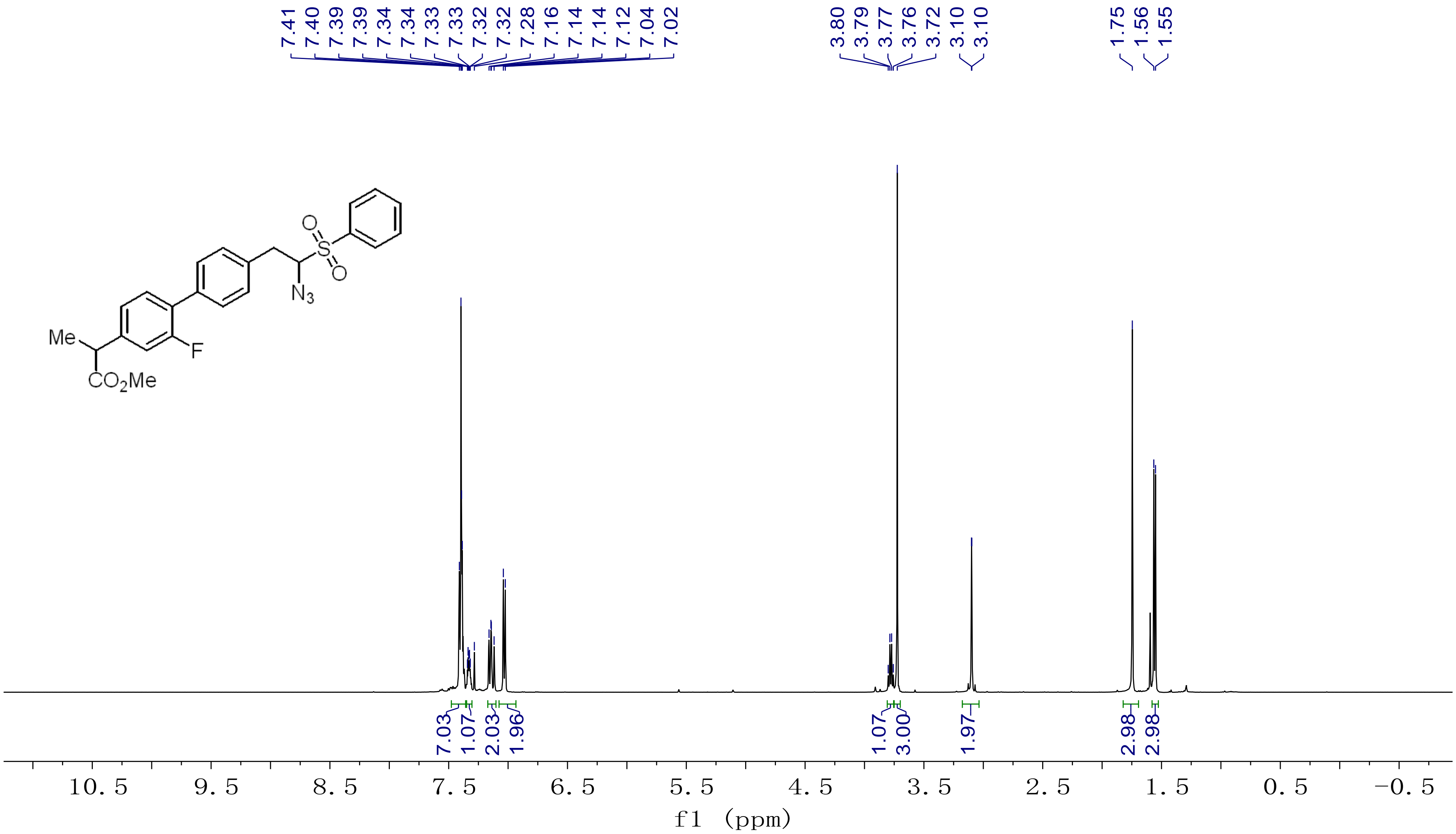

**$^{19}$F NMR of flubiprofen-derived phenylethylazide 33**

$CDCl_3$, 23 ºC

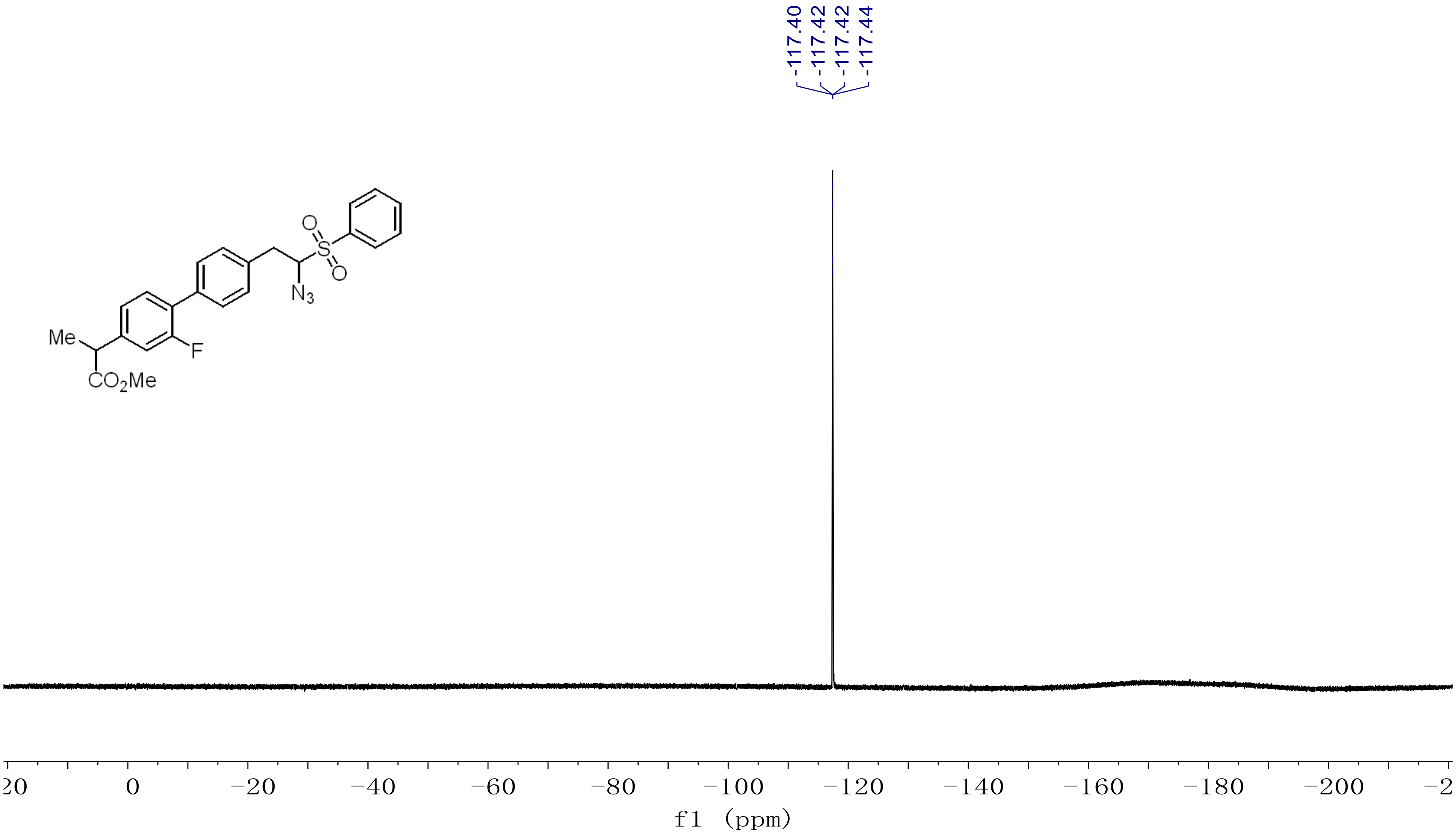

**$^{13}$C NMR of flubiprofen-derived phenylethylazide 33**

$CDCl_3$, 23 ºC

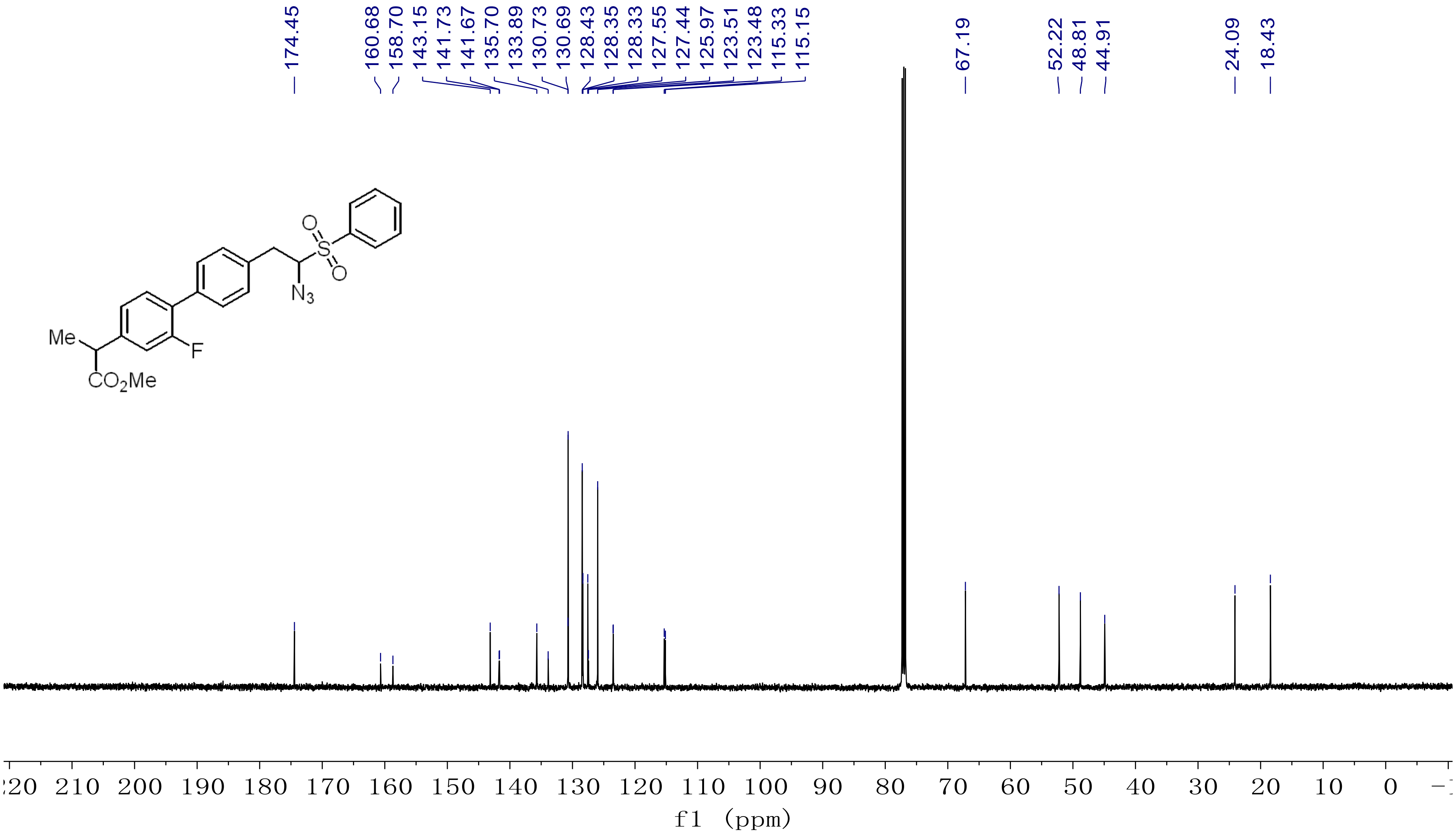

## $^{1}$H NMR of flubiprofen-derived phenylethylazide 34

CDCl3, 23 ºC

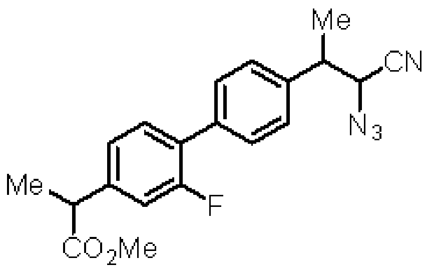


**1.2:1 dr**

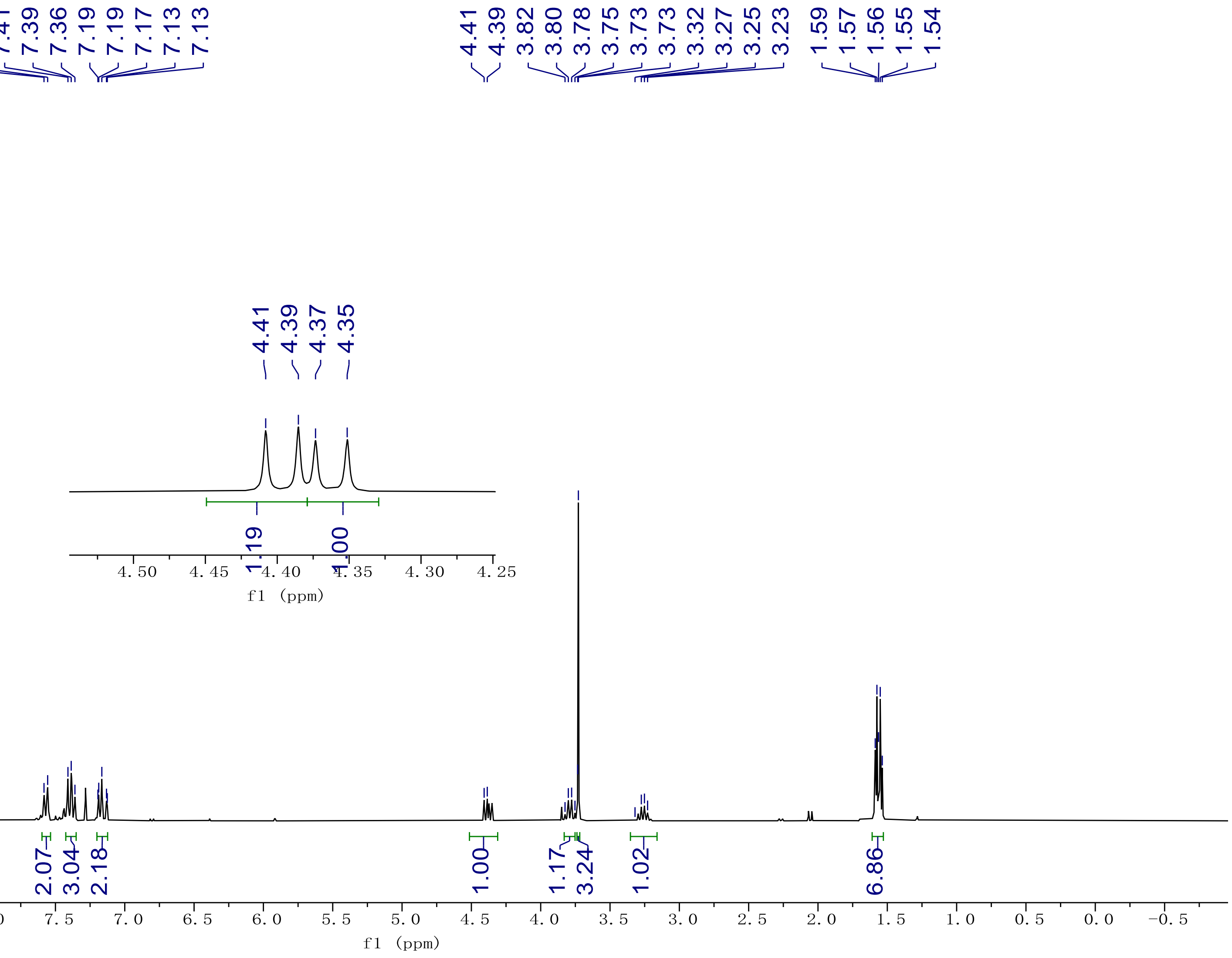

## $^{19}$F NMR of flubiprofen-derived phenylethylazide 34

CDCl3, 23 ºC

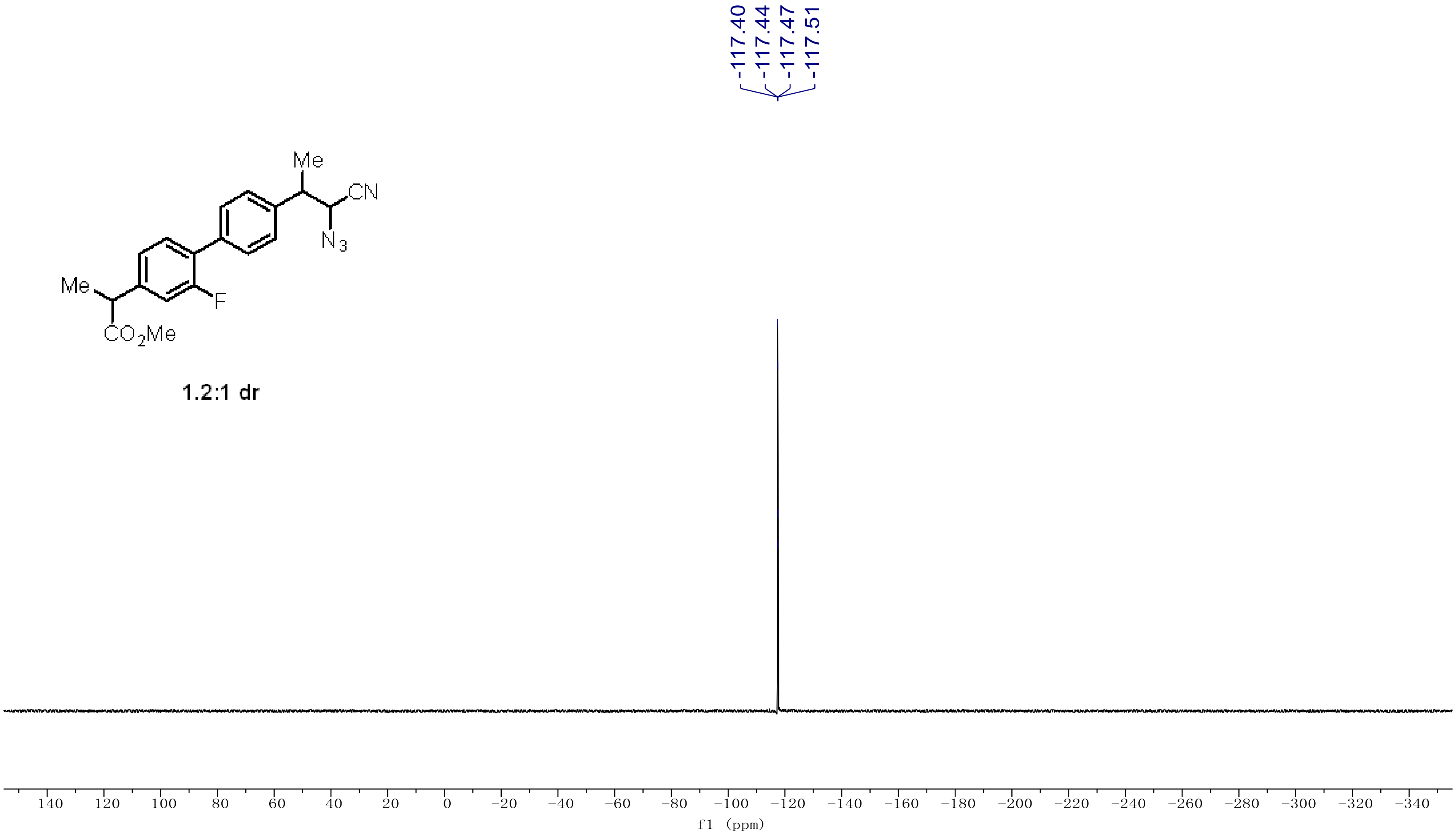

## $^{13}$C NMR of flubiprofen-derived phenylethylazide 34

CDCl$_3$, 23 ºC

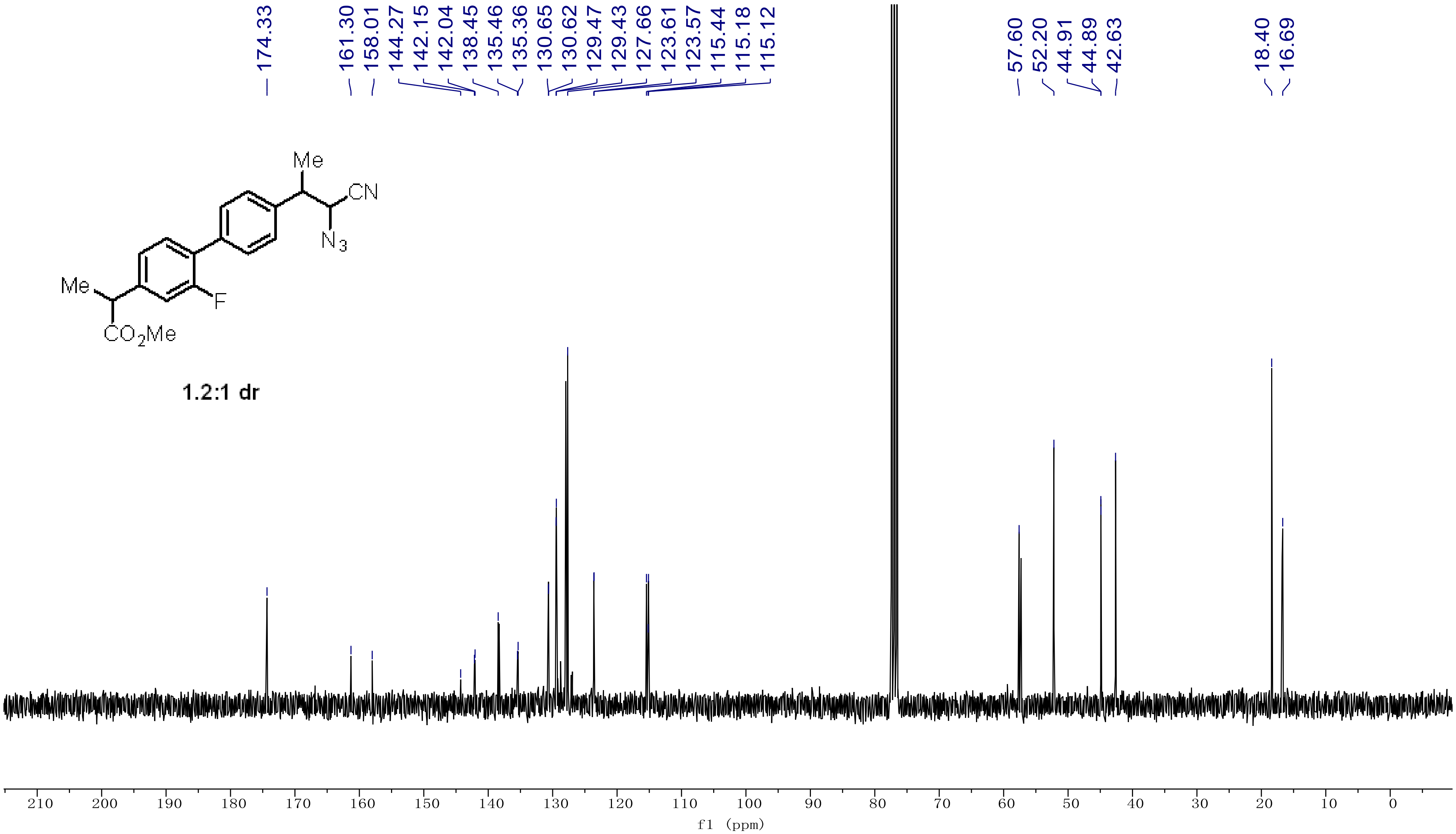


**1.2:1 dr**

### $^{1}$H NMR of flubiprofen-derived phenylethylazide 35

$CDCl_3$, 23 ºC

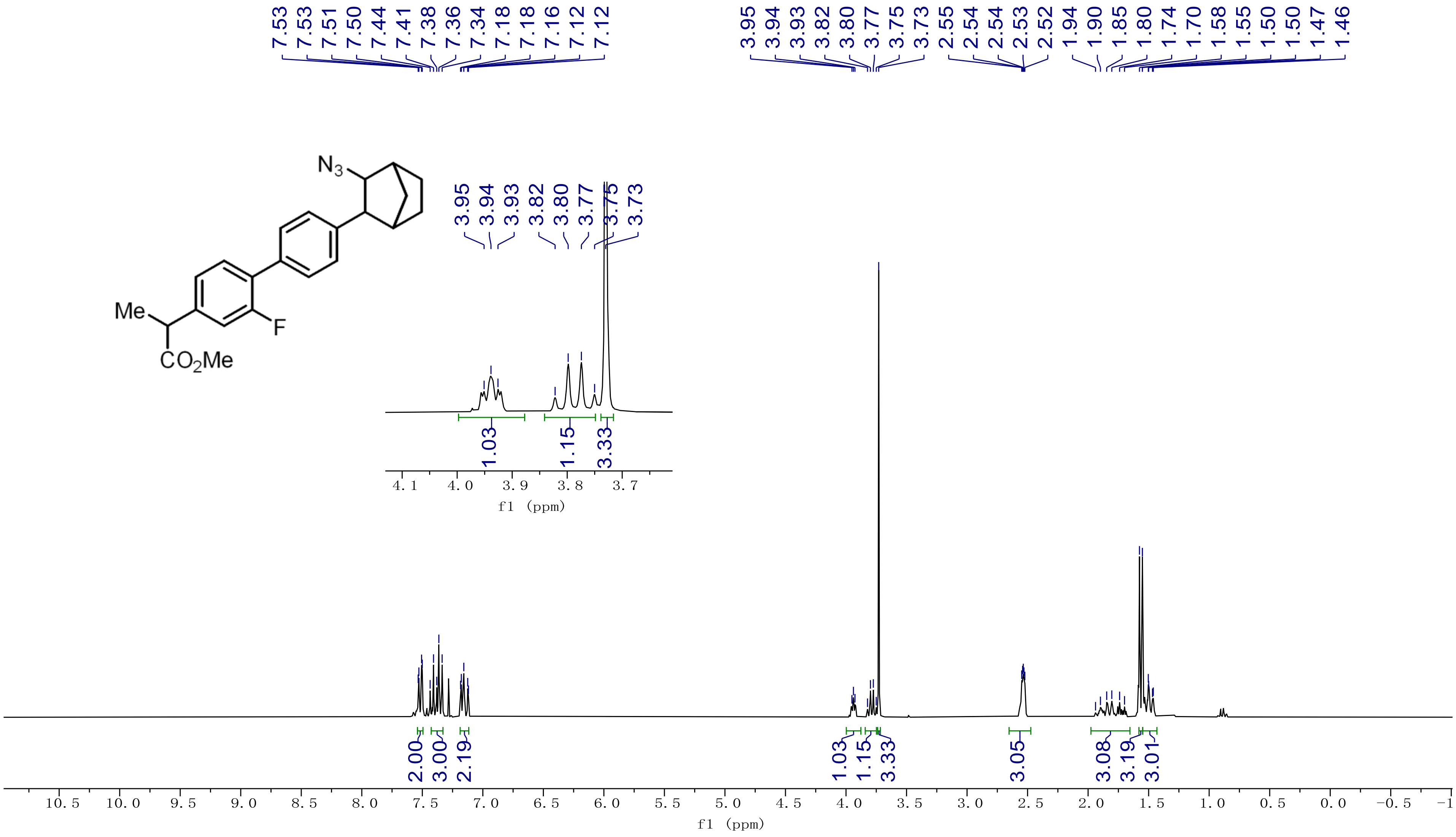

**$^{19}$F NMR of flubiprofen-derived phenylethylazide 35**

$CDCl_3$, 23 ºC

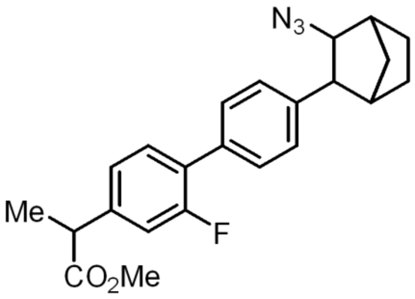


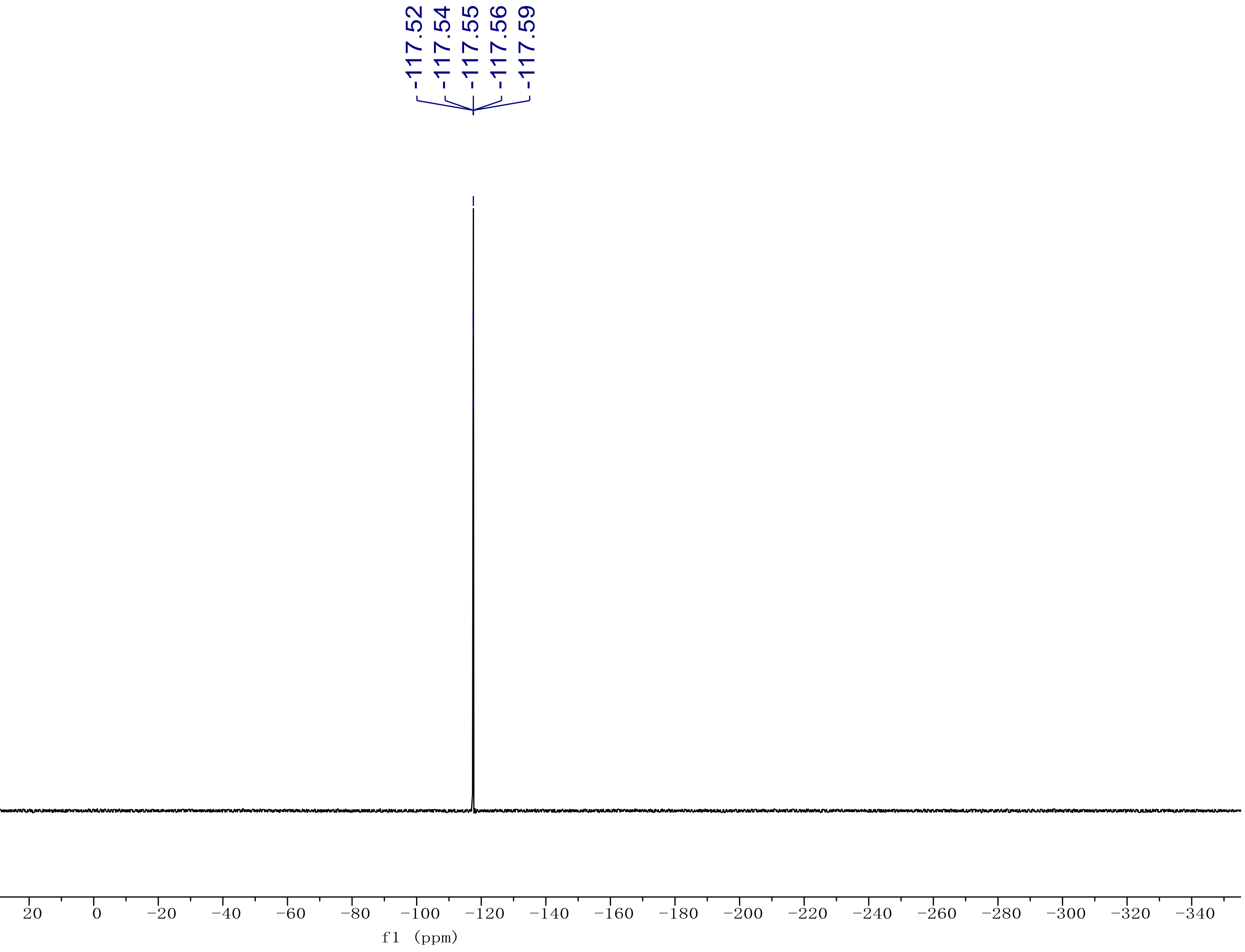

**$^{13}$C NMR of flubiprofen-derived phenylethylazide 35**

$CDCl_3$, 23 ºC

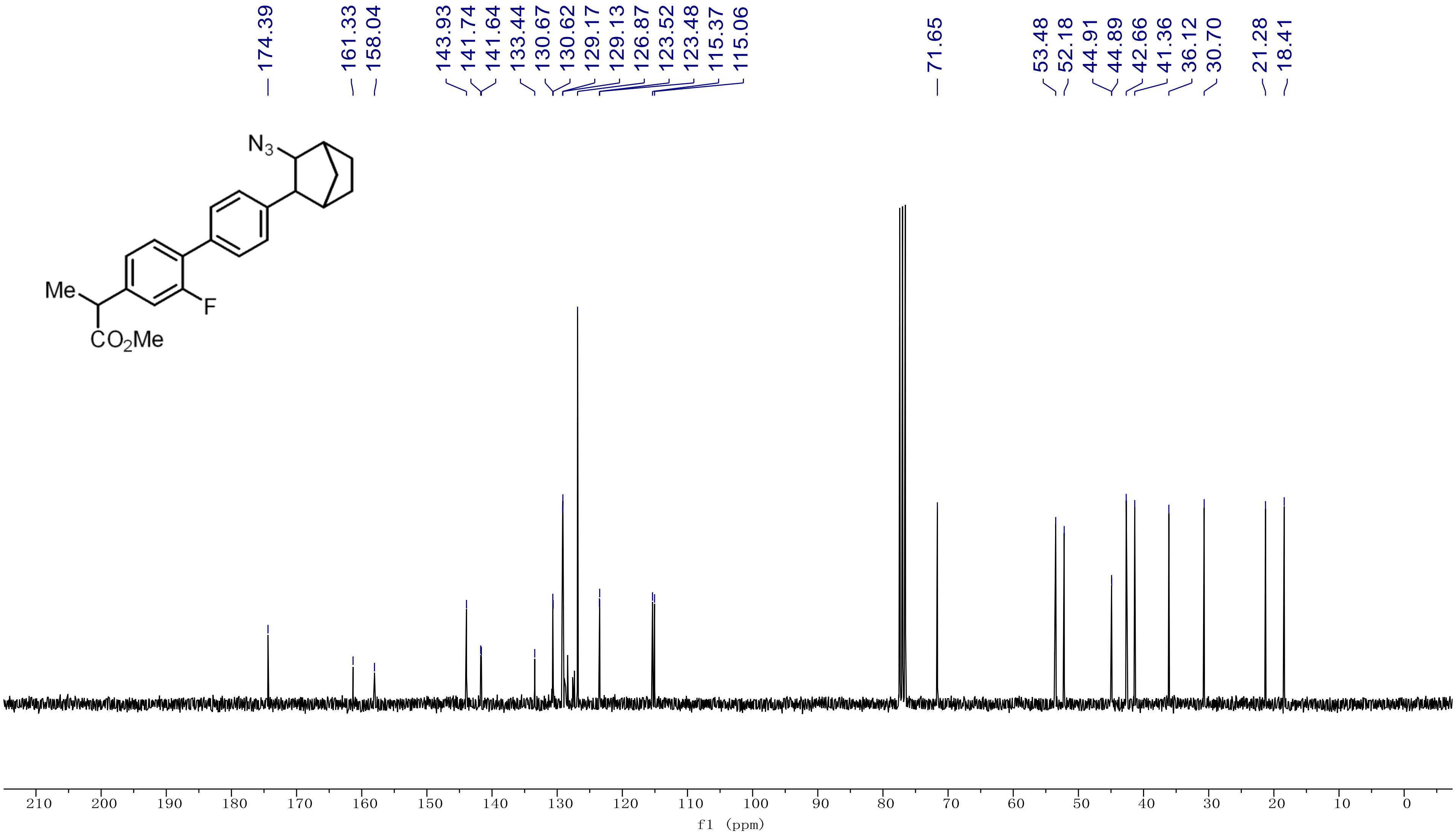

**$^{1}$H NMR of flubiprofen-derived phenylethylazide 36**

$CDCl_3$, 23 ºC

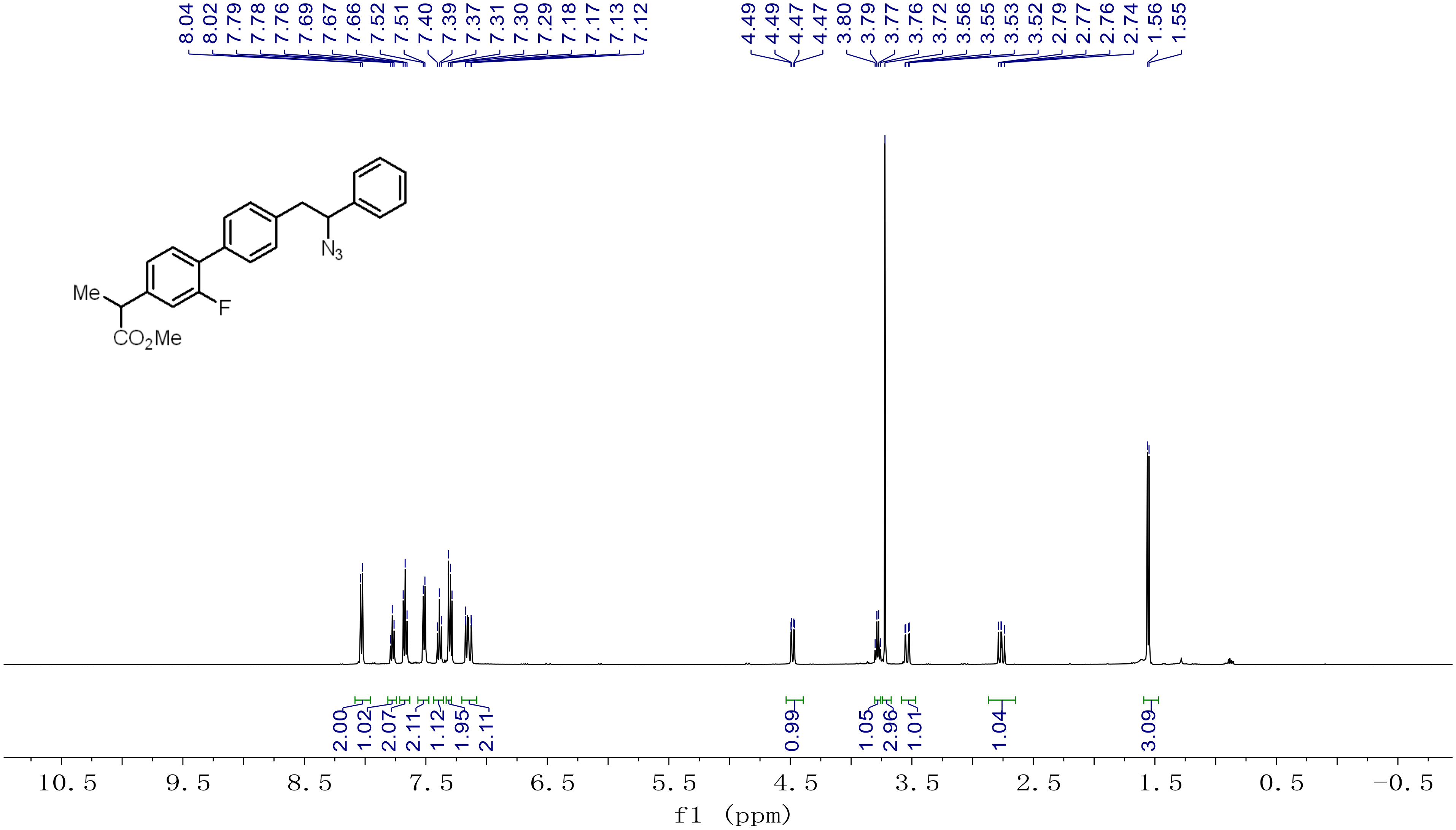

**$^{19}$F NMR of flubiprofen-derived phenylethylazide 36**

$CDCl_3$, 23 ºC

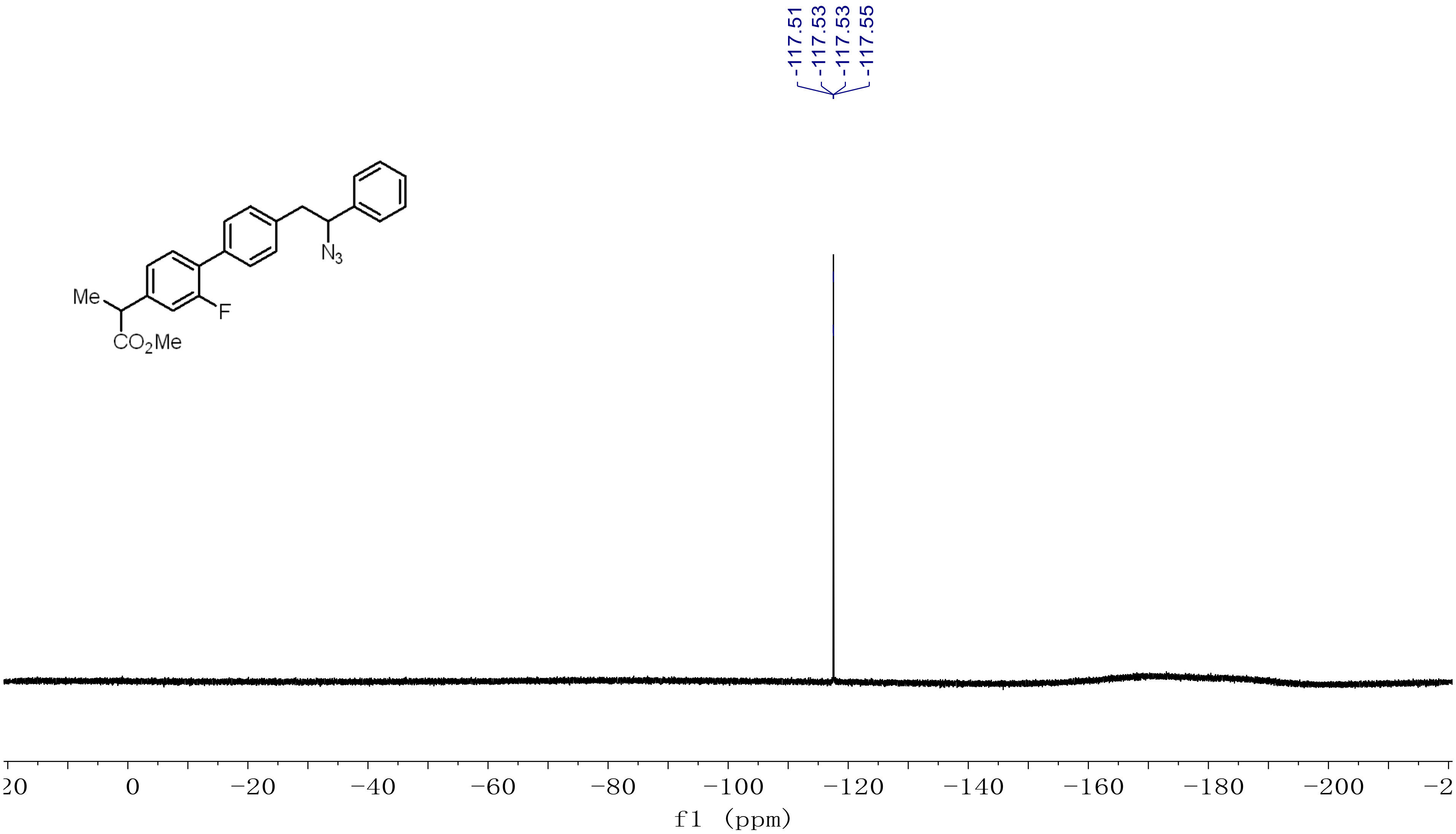

**$^{13}$C NMR of flubiprofen-derived phenylethylazide 36**

$CDCl_3$, 23 ºC

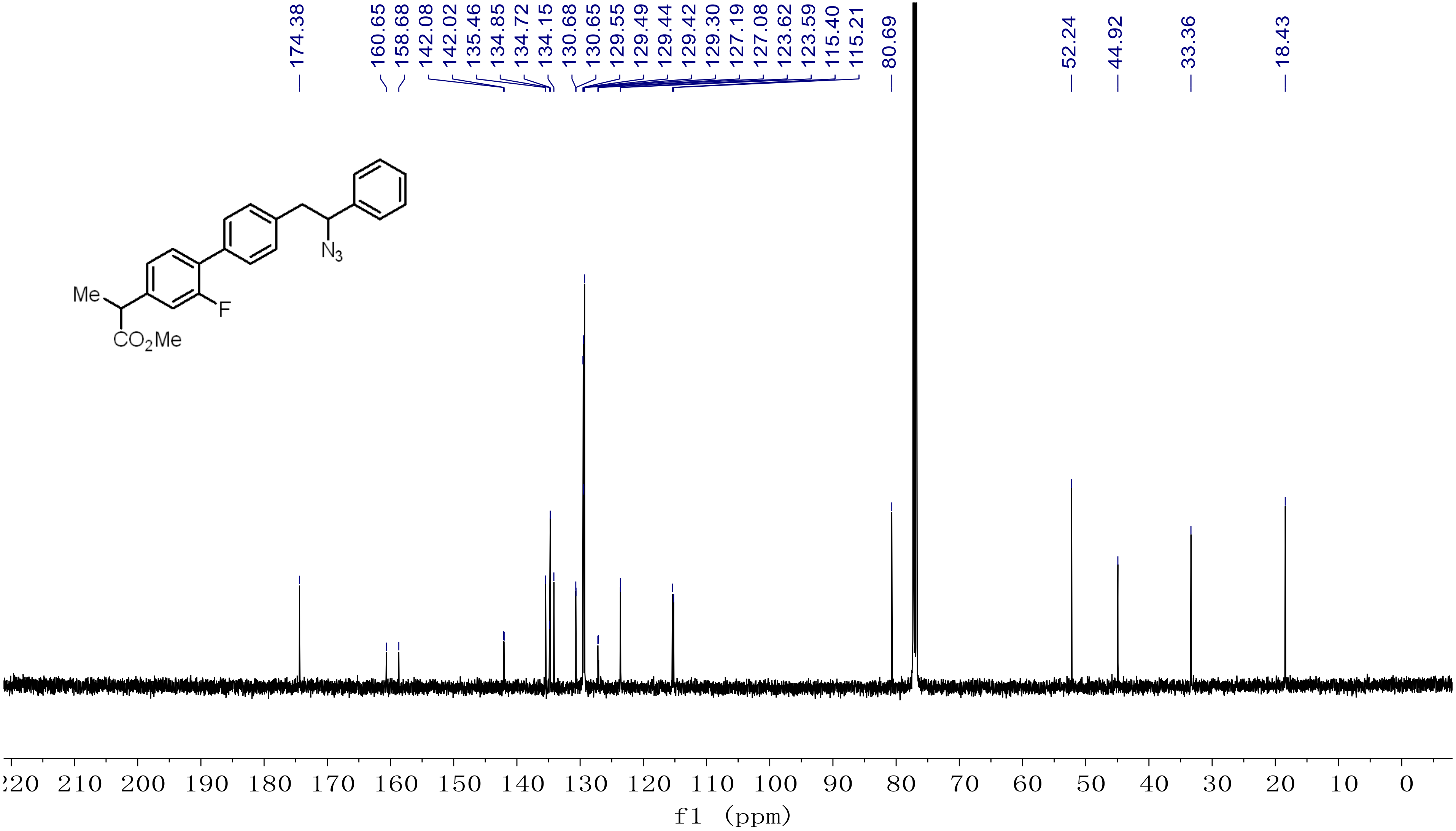

**$^{1}$H NMR of flubiprofen-derived phenylethylazide 37**

$CDCl_3$, 23 ºC

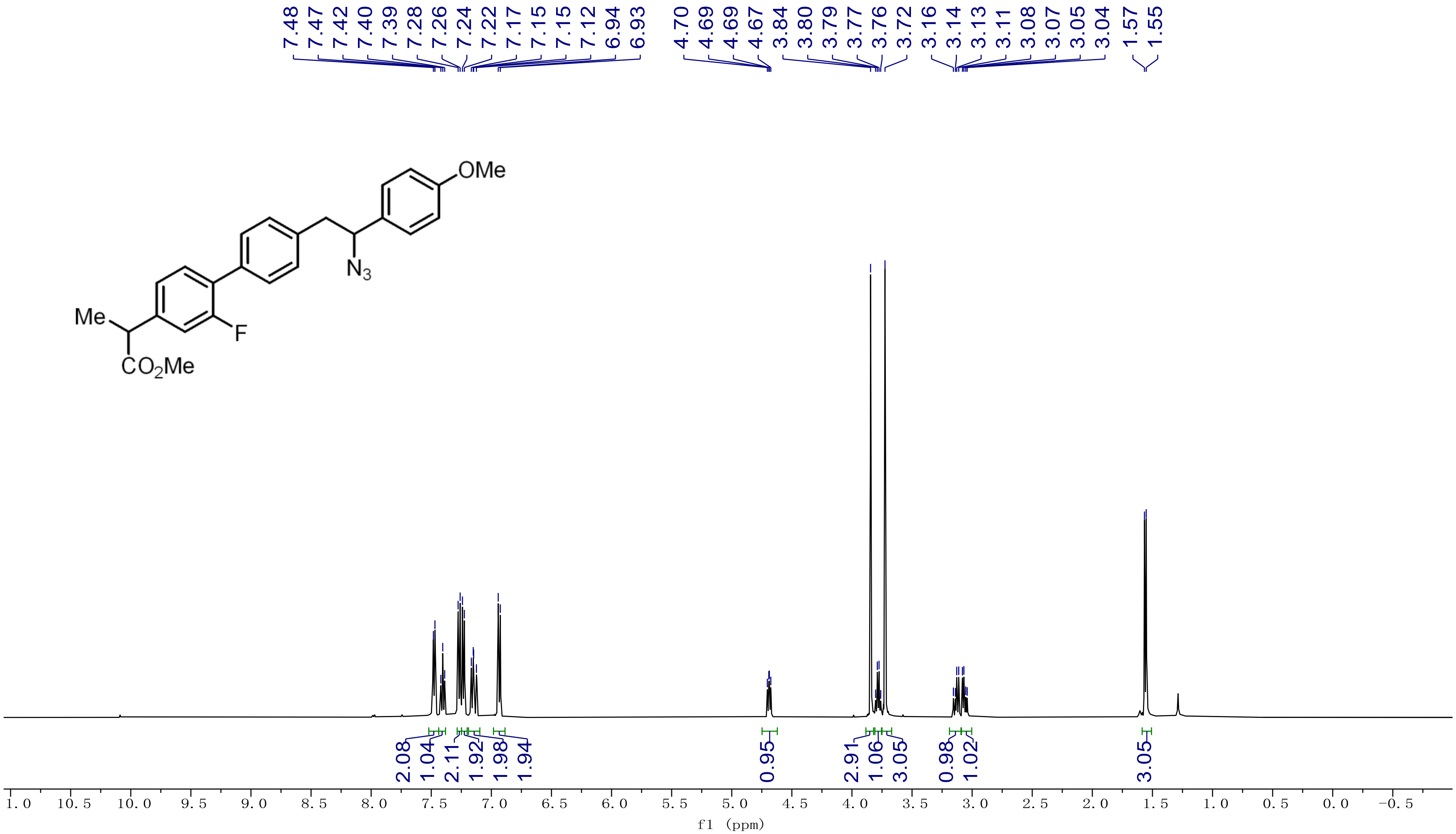

## $^{19}$F NMR of flubiprofen-derived phenylethylazide 37

$CDCl_3$, 23 ºC

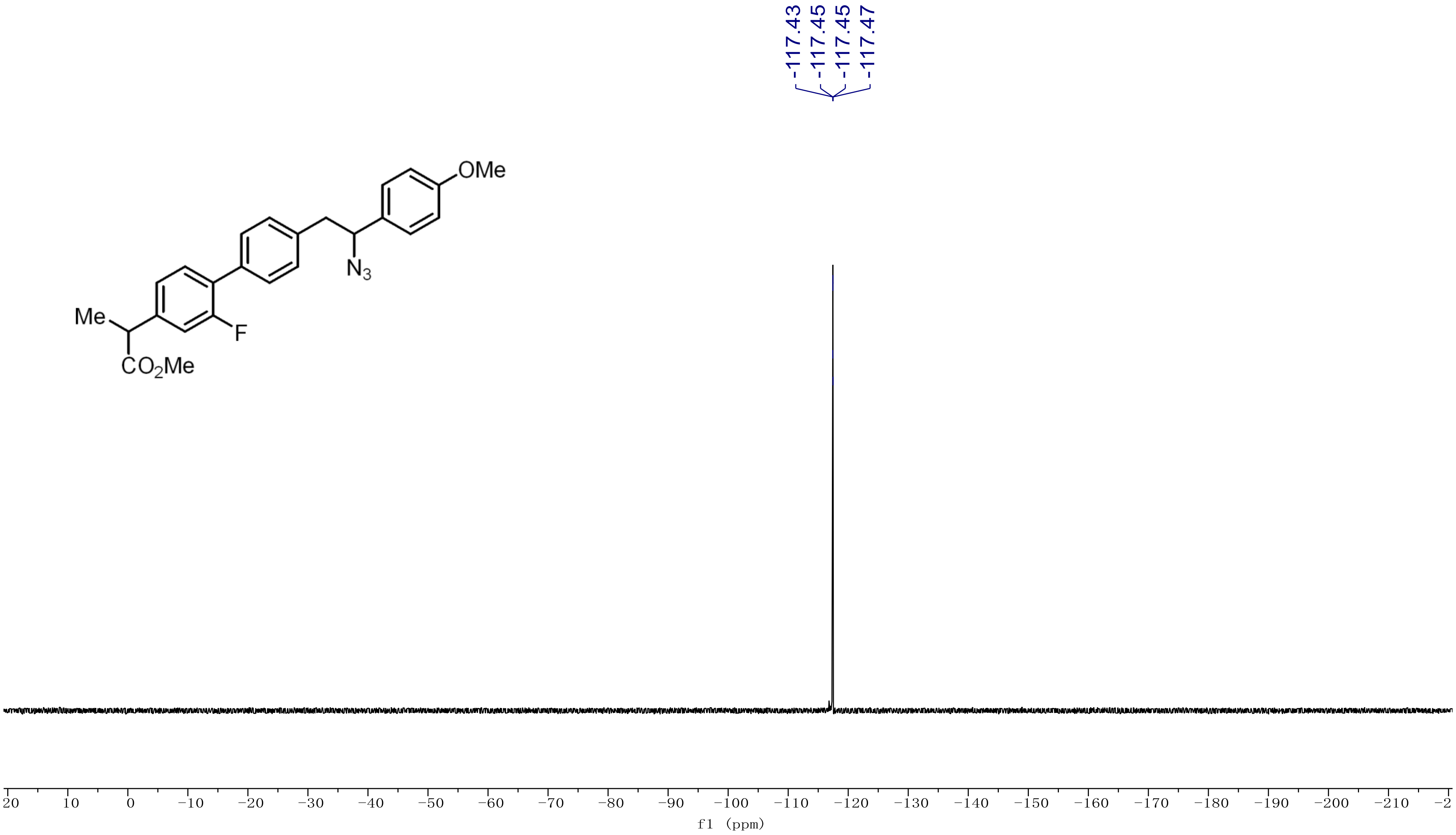

## $^{13}$C NMR of flubiprofen-derived phenylethylazide 37

$CDCl_3$, 23 ºC

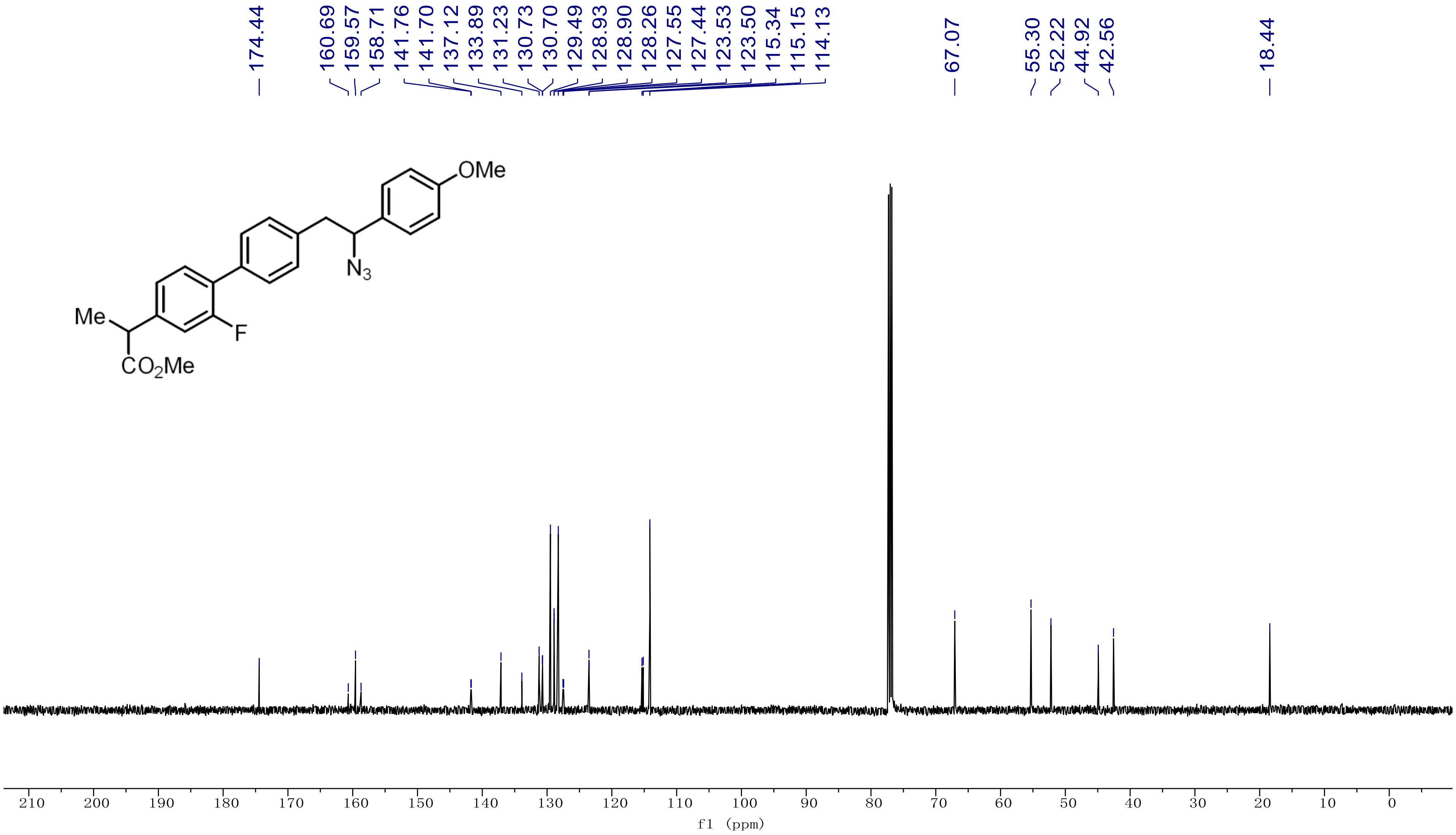

**$^{1}$H NMR of flubiprofen-derived phenylethylazide 38**

$CDCl_3$, 23 ºC

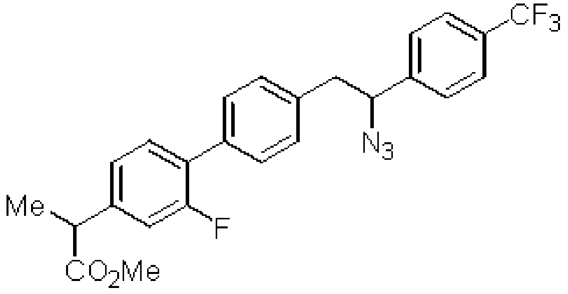


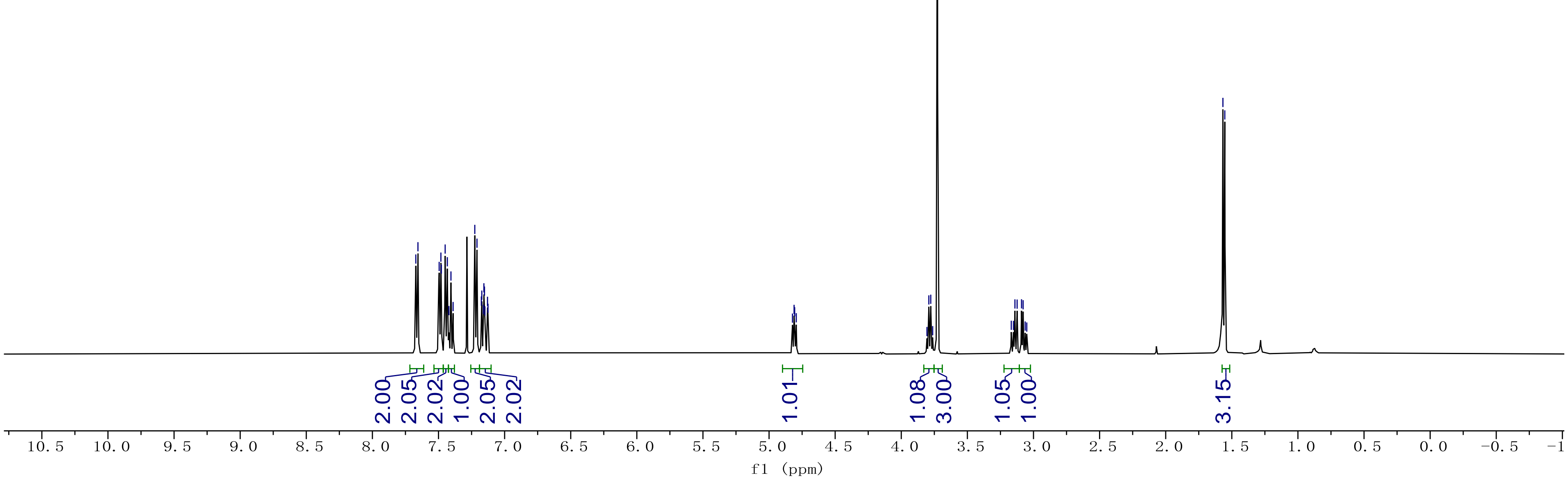

**$^{19}$F NMR of flubiprofen-derived phenylethylazide 38**

$CDCl_3$, 23 ºC

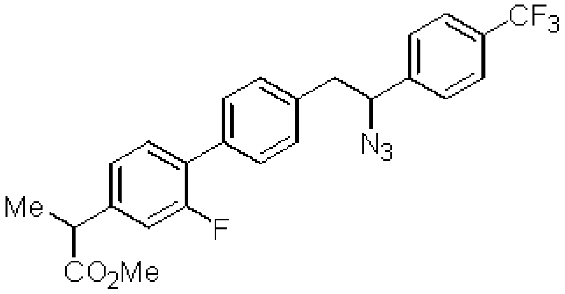


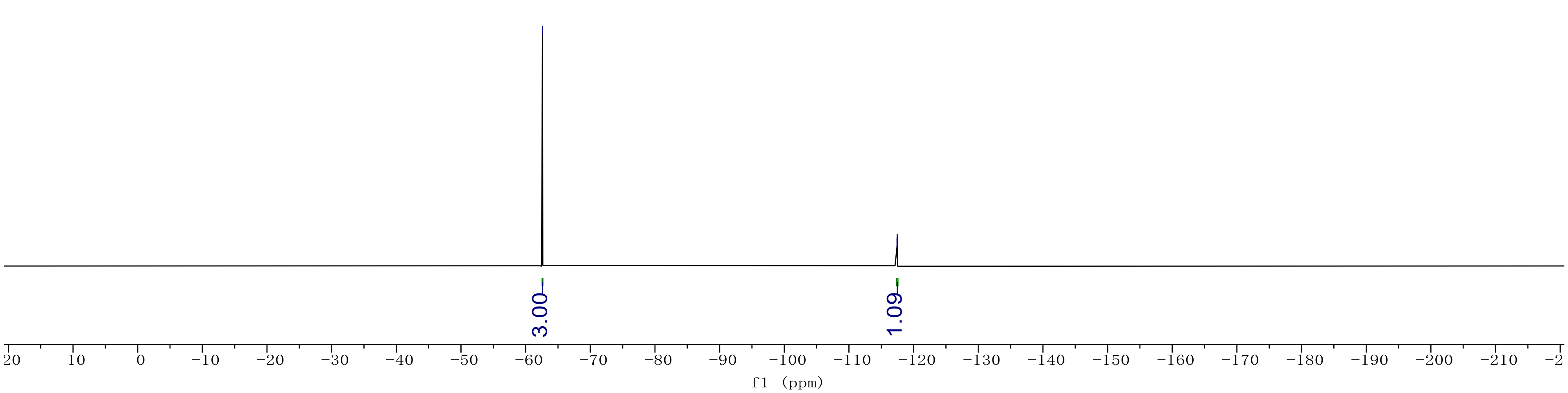

## $^{13}$C NMR of flubiprofen-derived phenylethylazide 38

$CDCl_3$, 23 ºC

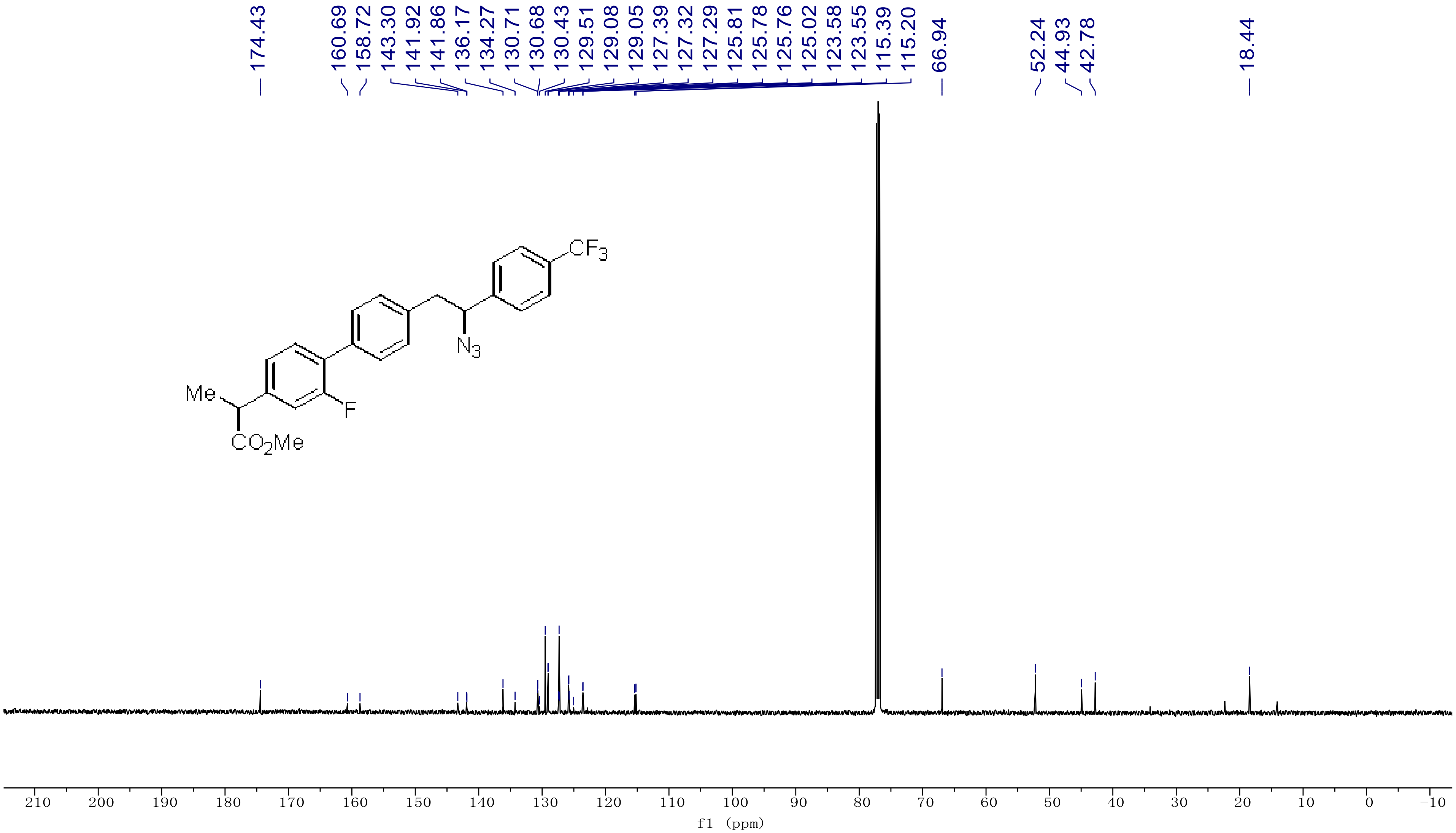

**$^{1}$H NMR of flubiprofen-derived phenylethylazide 39**

$CDCl_3$, 23 ºC

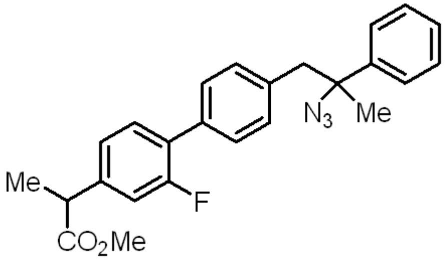


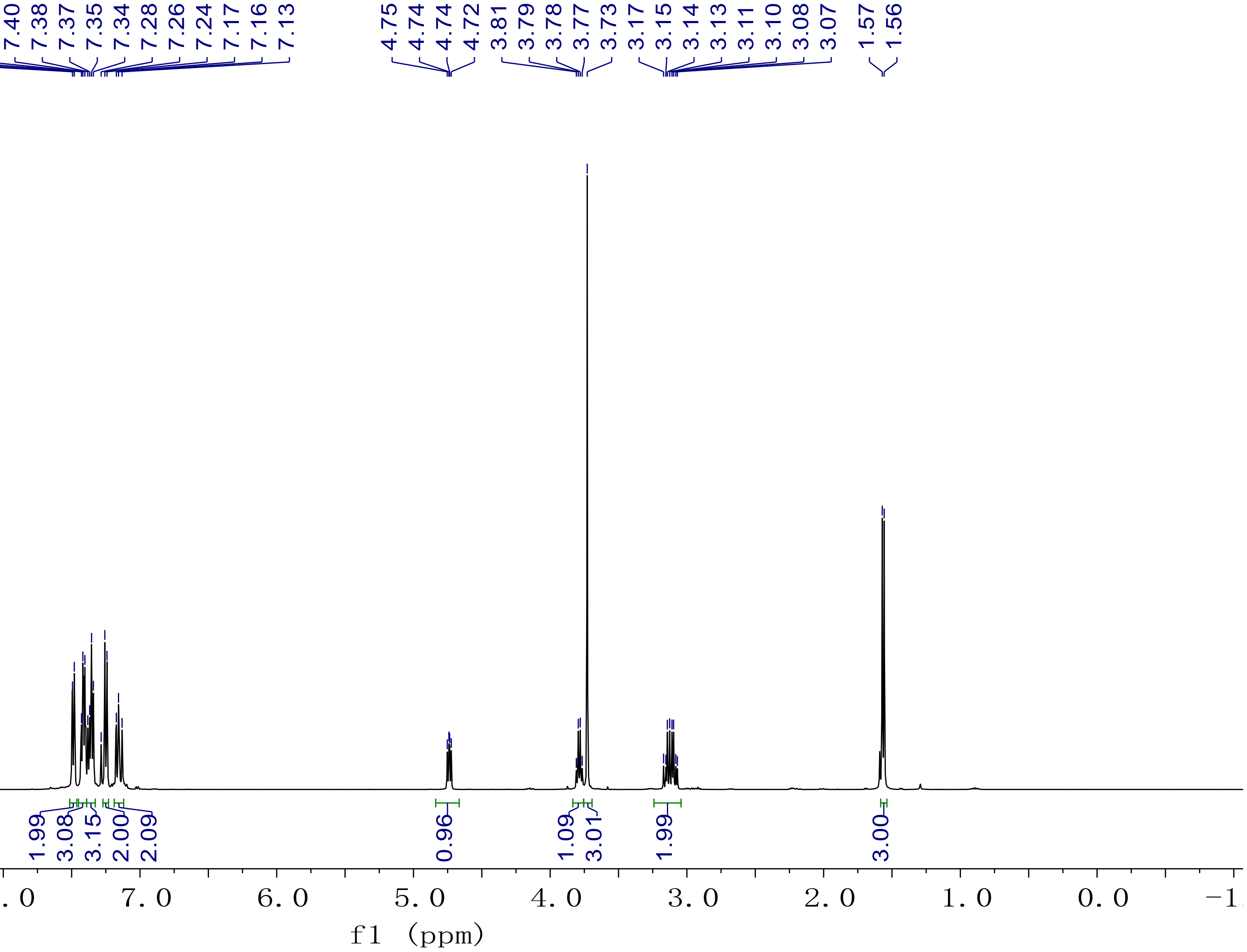

**$^{19}$F NMR of flubiprofen-derived phenylethylazide 39**

$CDCl_3$, 23 ºC

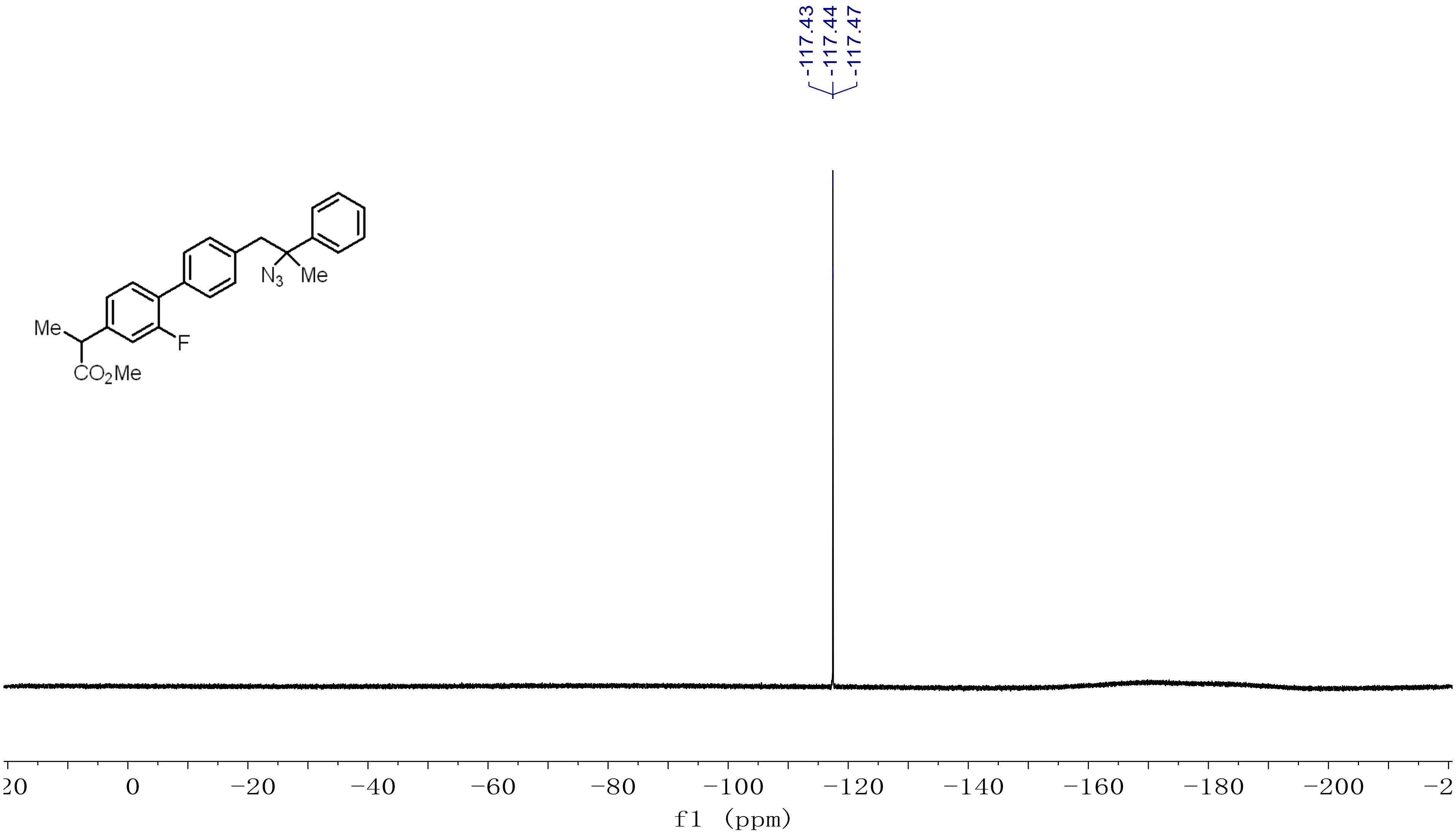

## $^{13}$C NMR of flubiprofen-derived phenylethylazide 39

CDCl$_3$, 23 ºC

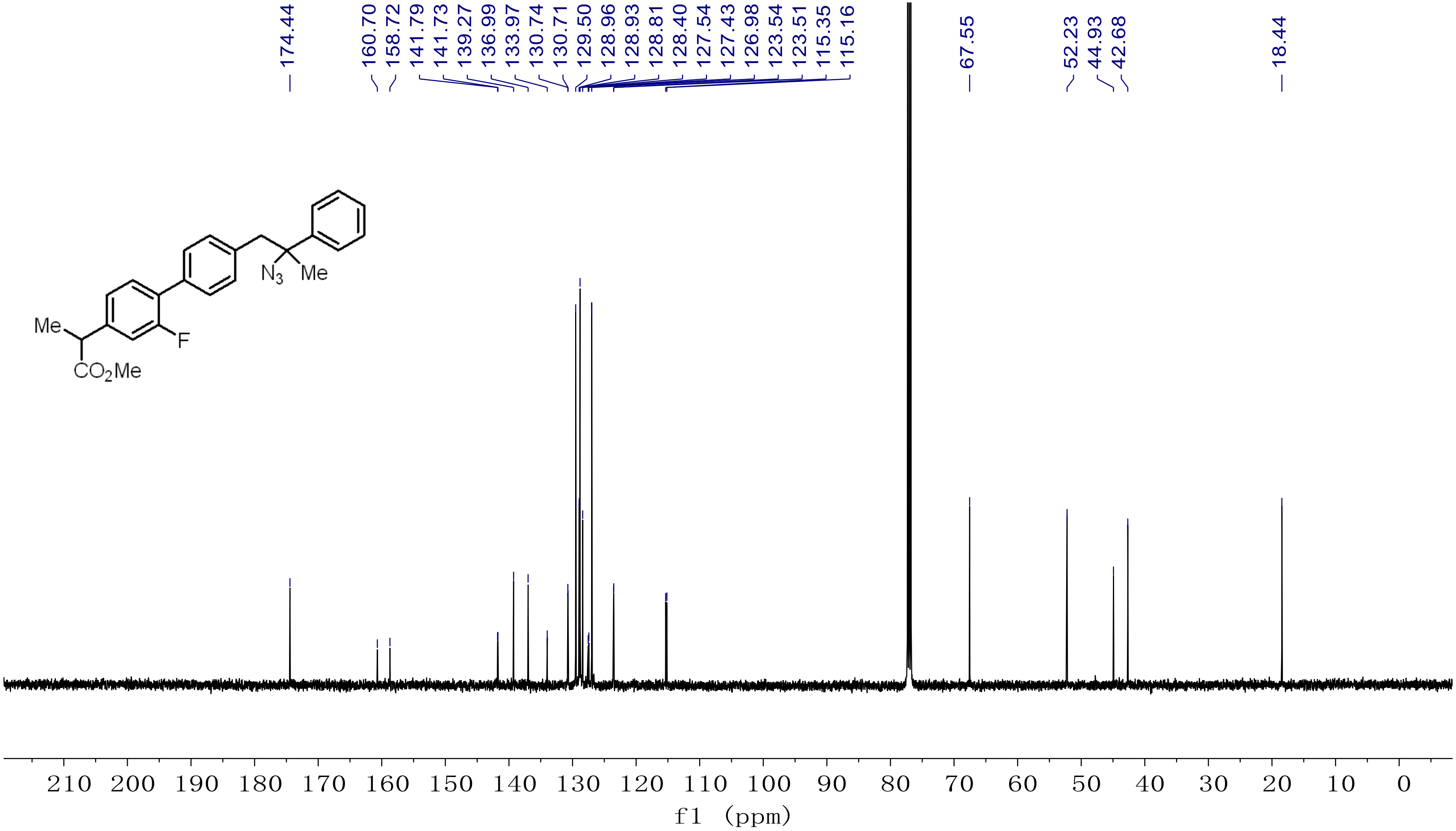

# $^1$H NMR of *rac*-flubiprofen-derived phenylethylazide 40

CDCl$_3$, 23 ºC

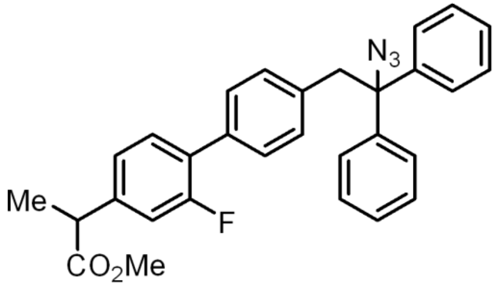


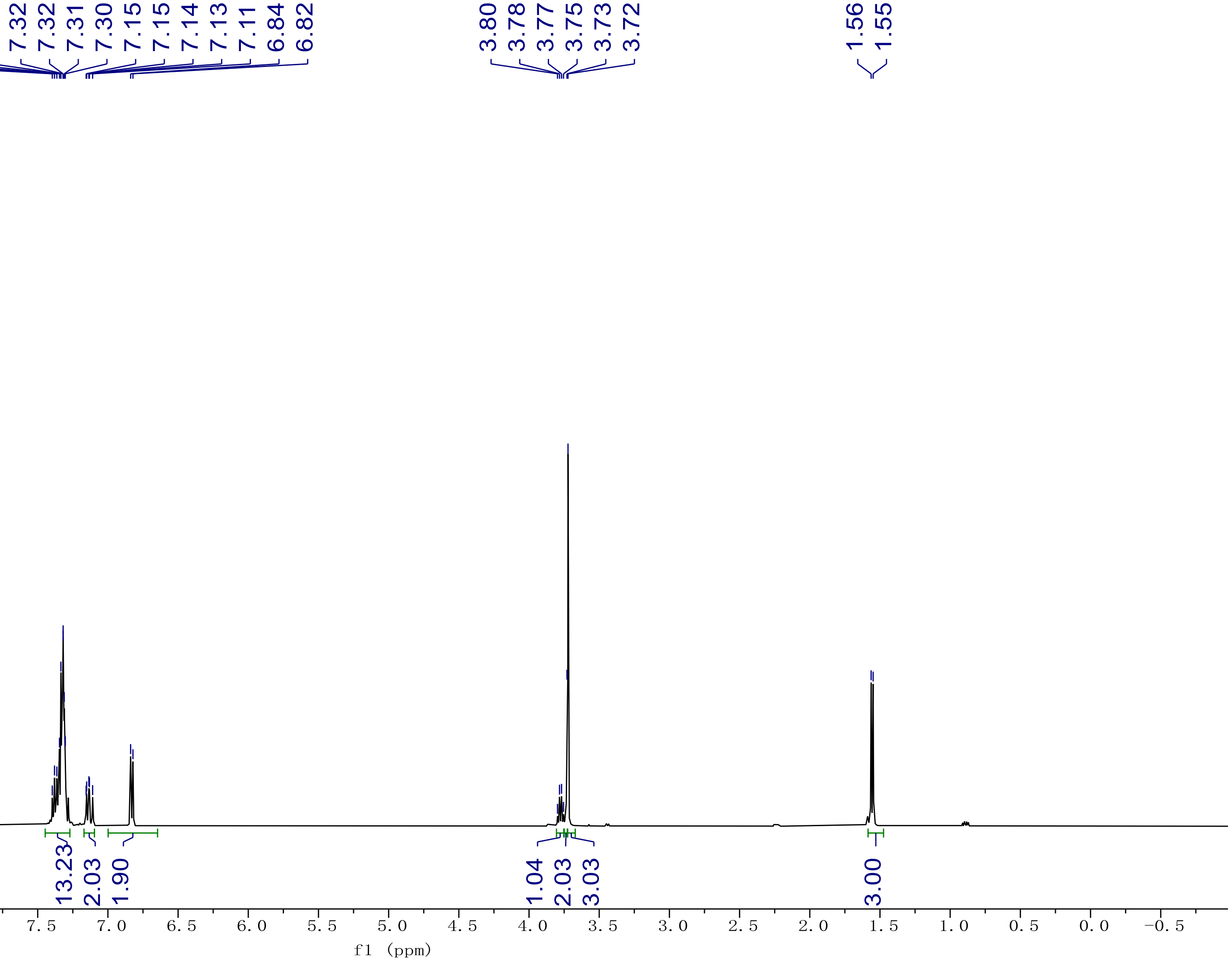

**$^{19}$F NMR of *rac*-flubiprofen-derived phenylethylazide 40**

$CDCl_3$, 23 ºC

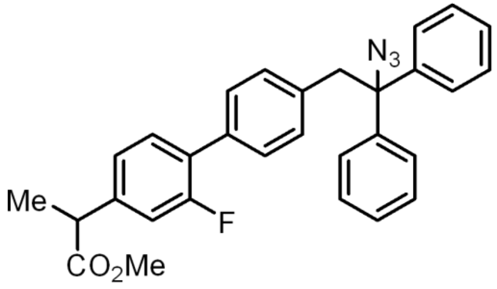


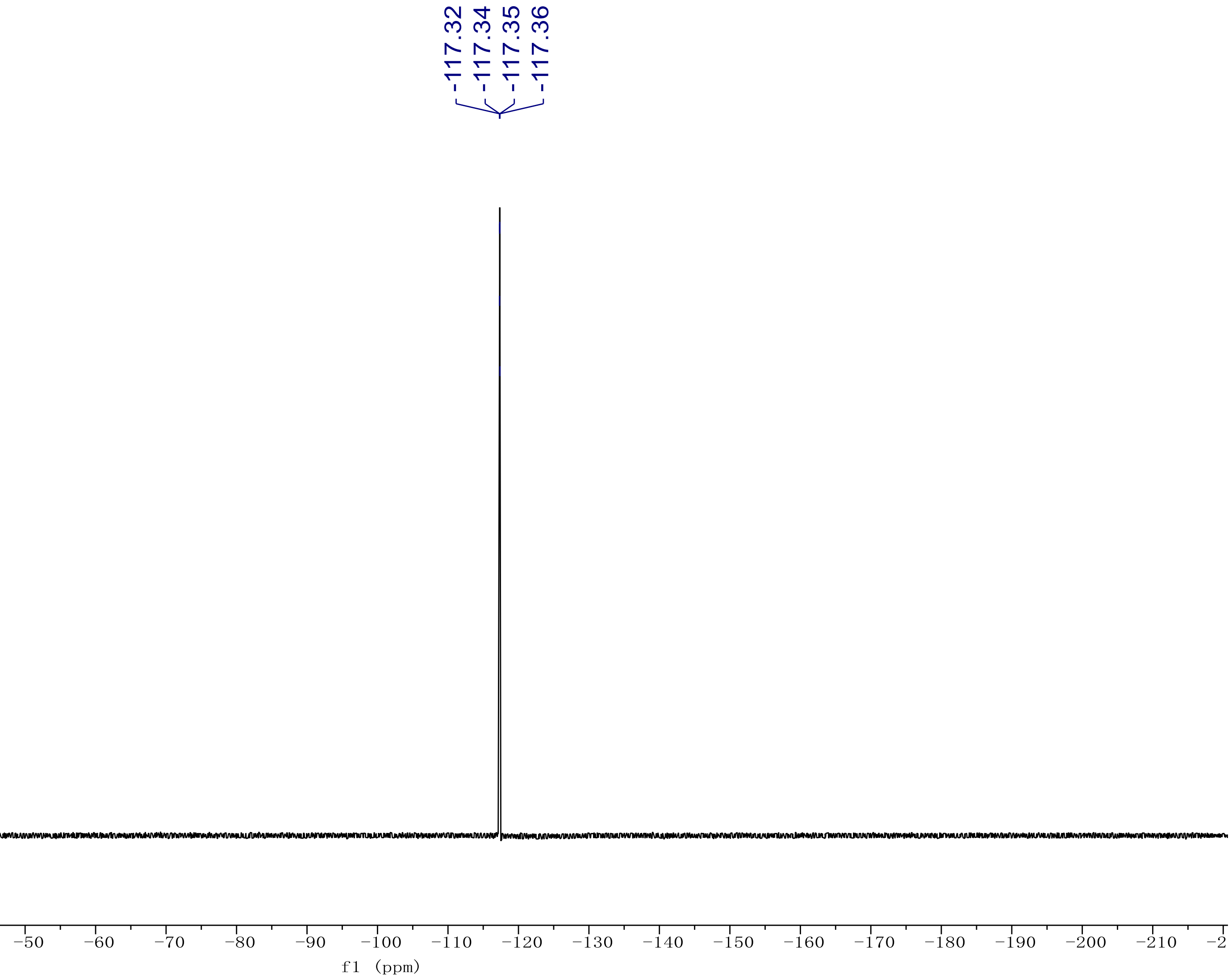

## $^{13}$C NMR of *rac*-flubiprofen-derived phenylethylazide 40

CDCl$_3$, 23 ºC

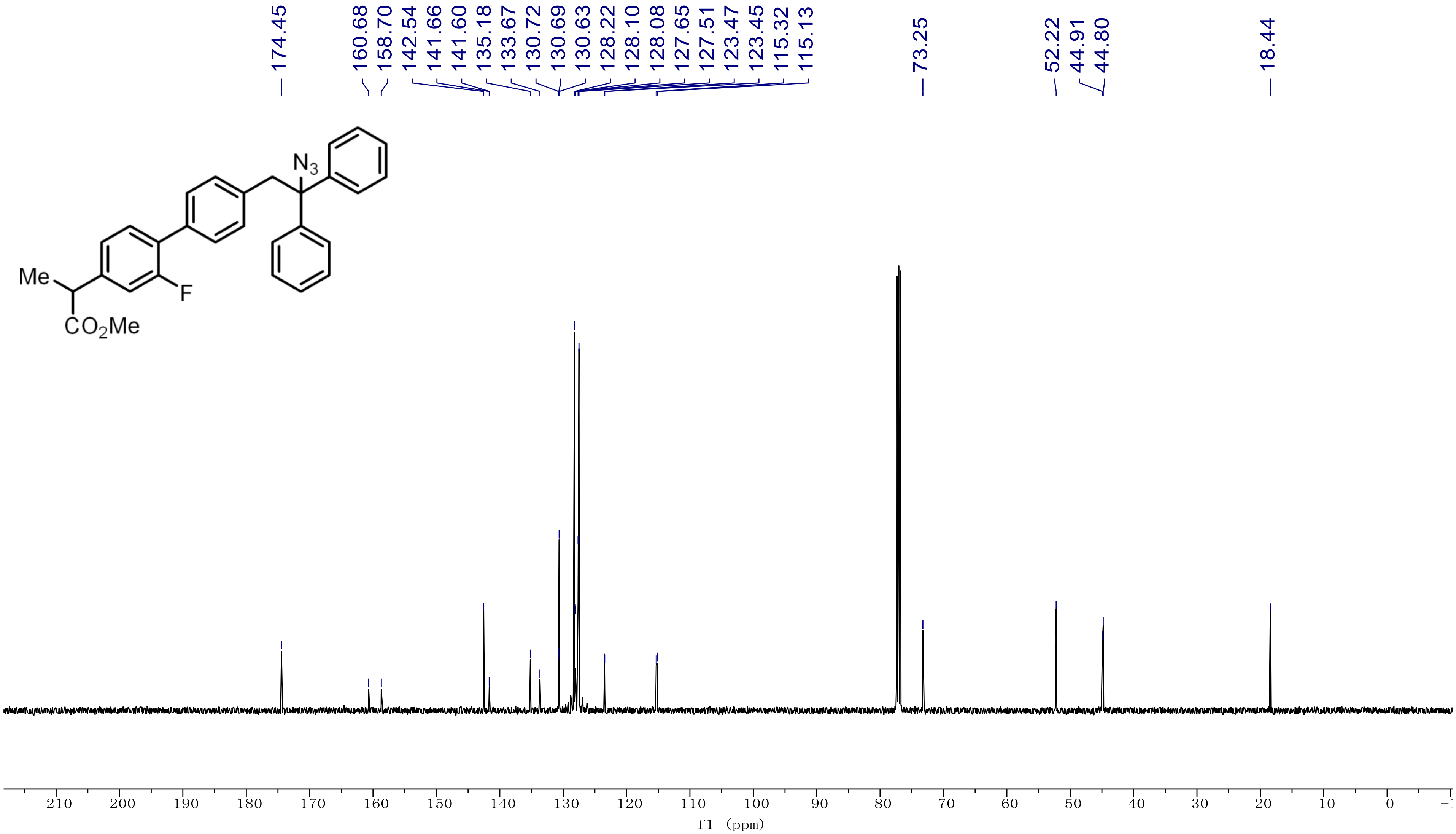

**$^{1}$H NMR of flubiprofen-derived phenylethylazide 41**

$CDCl_3$, 23 ºC

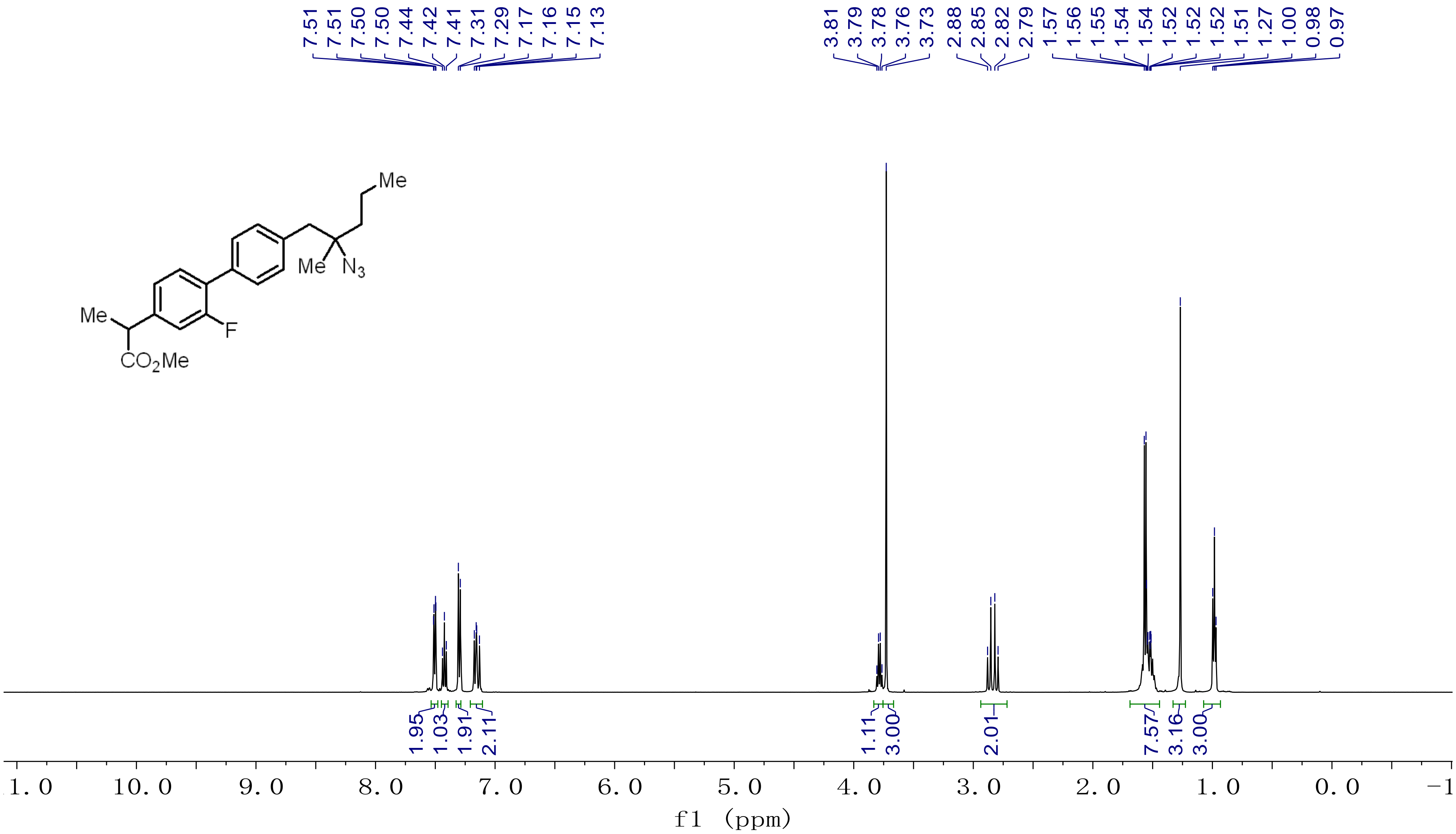

**$^{19}$F NMR of flubiprofen-derived phenylethylazide 41**

$CDCl_3$, 23 ºC

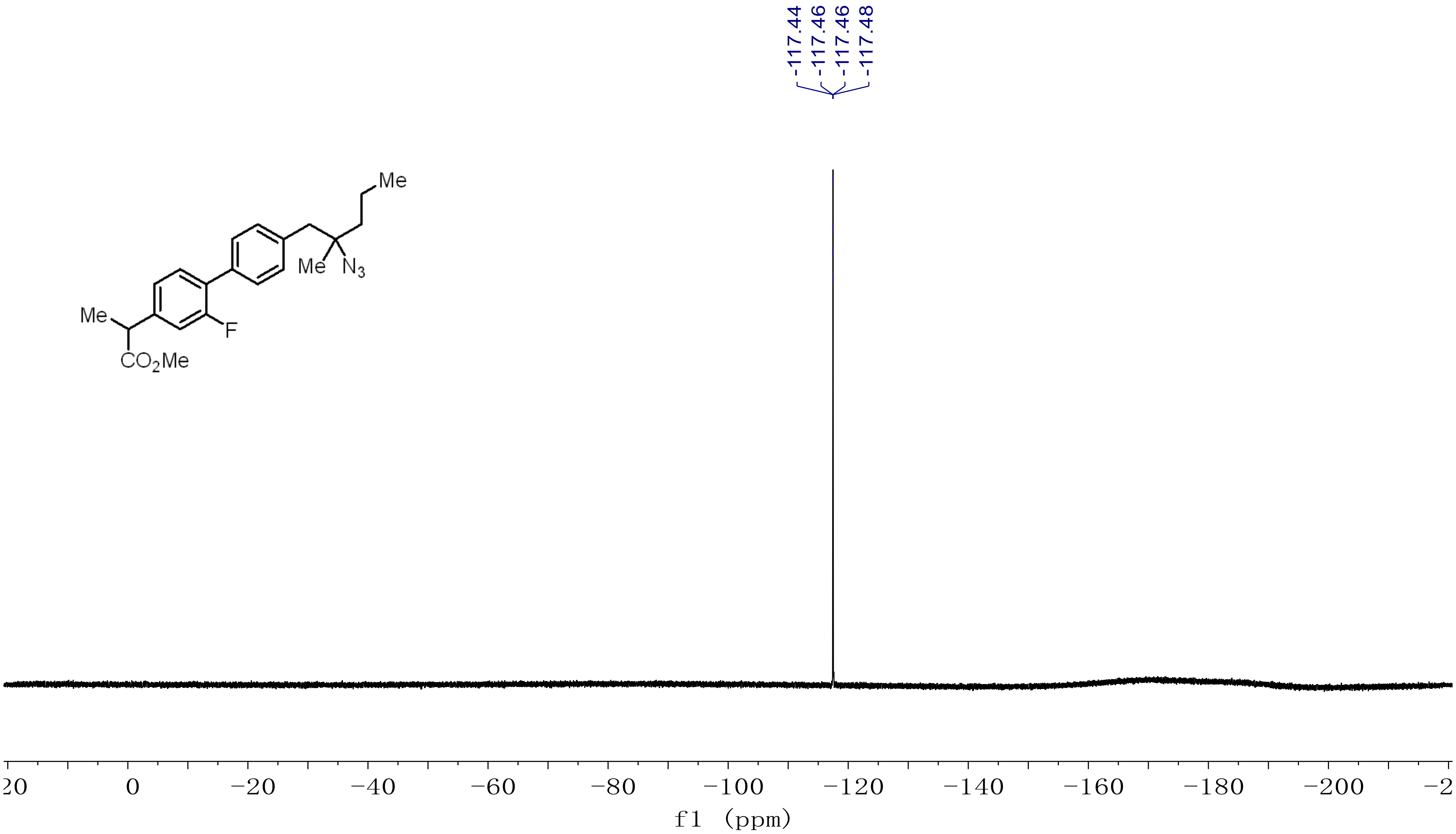

**$^{13}$C NMR of flubiprofen-derived phenylethylazide 41**

$CDCl_3$, 23 ºC

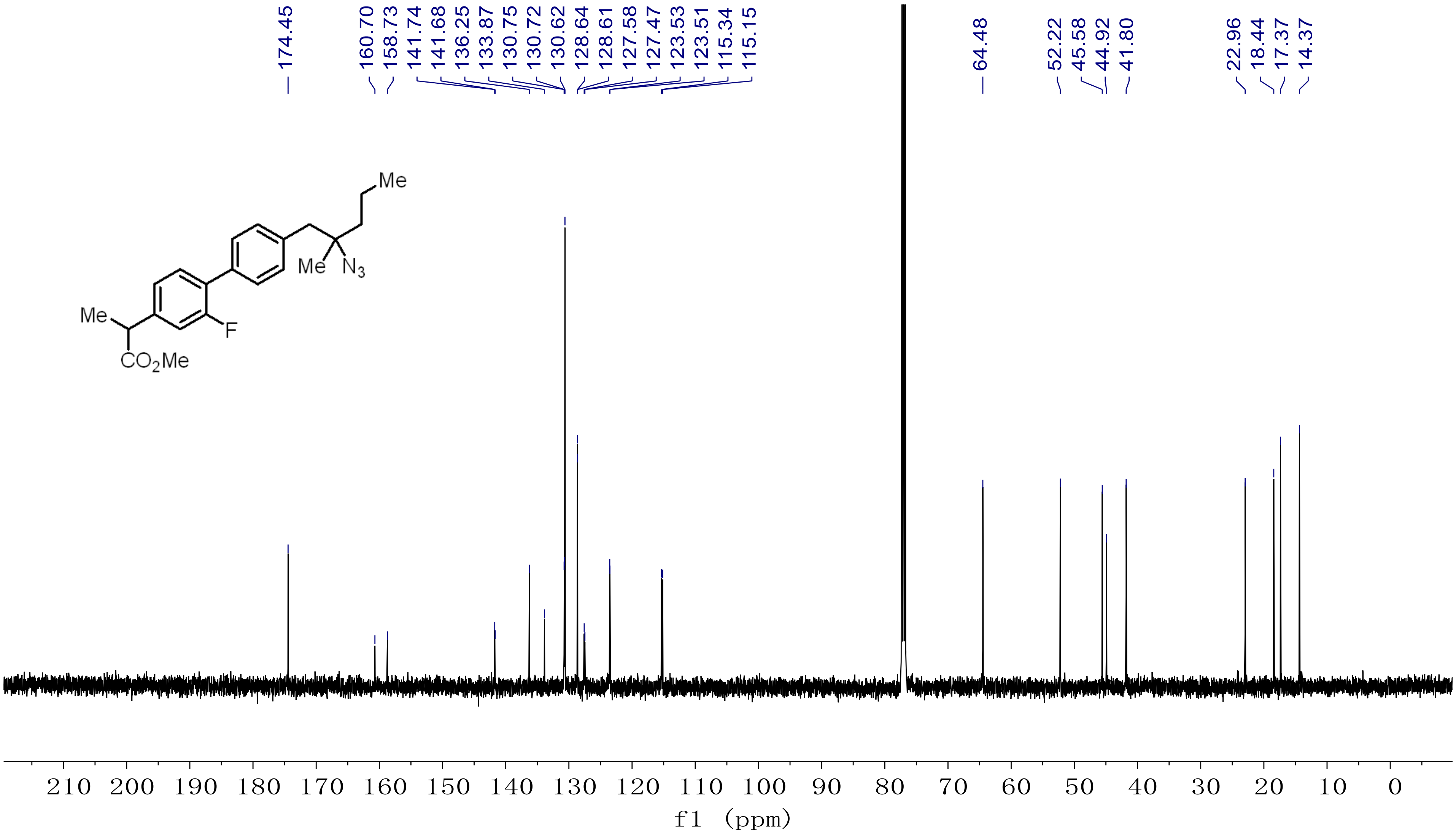

**$^{1}$H NMR of *rac*-pyridinylethylazide 42**

$CDCl_3$, 23 ºC

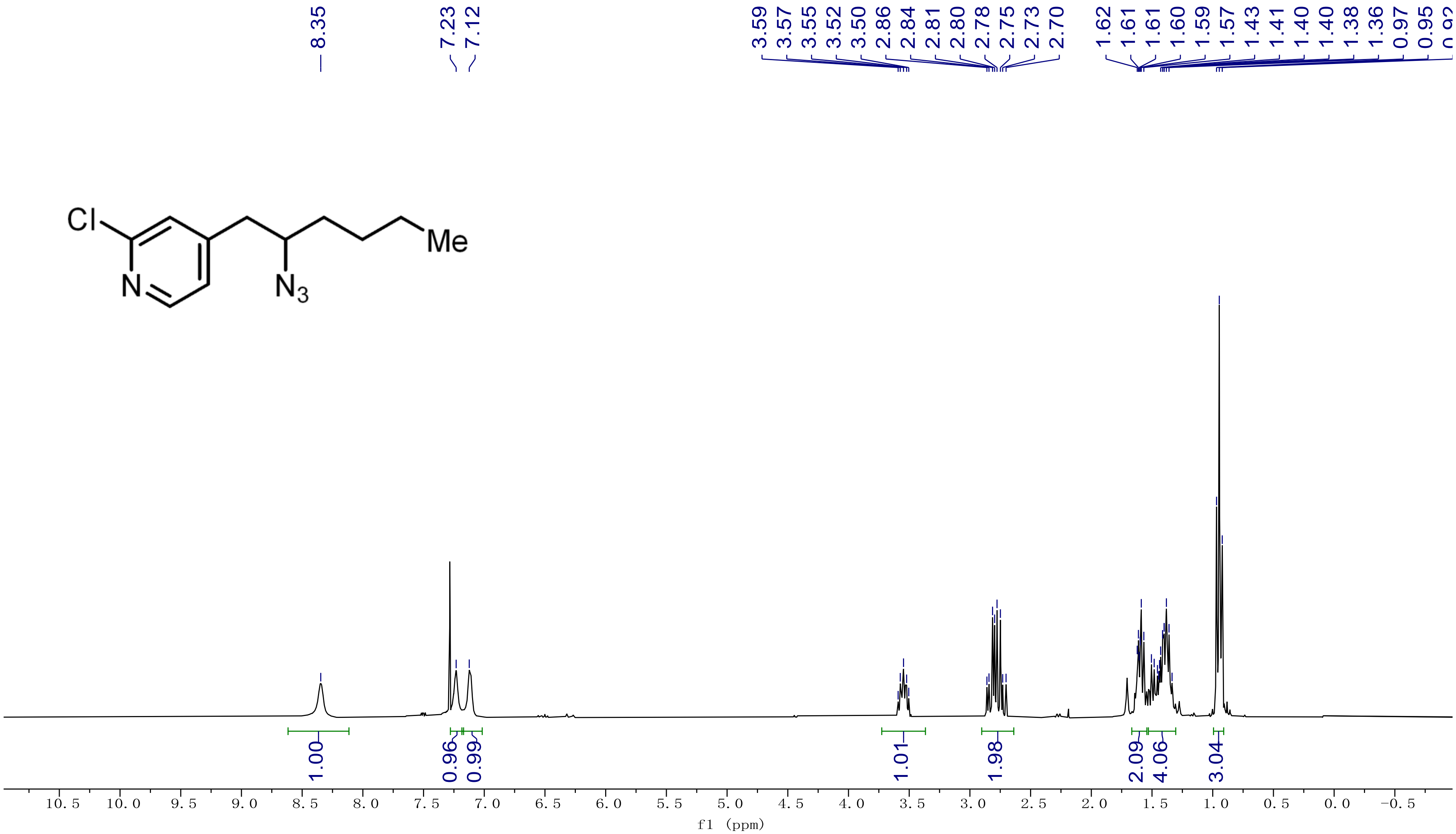

## $^{13}$C NMR of *rac*-pyridinylethylazide 42

$CDCl_3$, 23 ºC

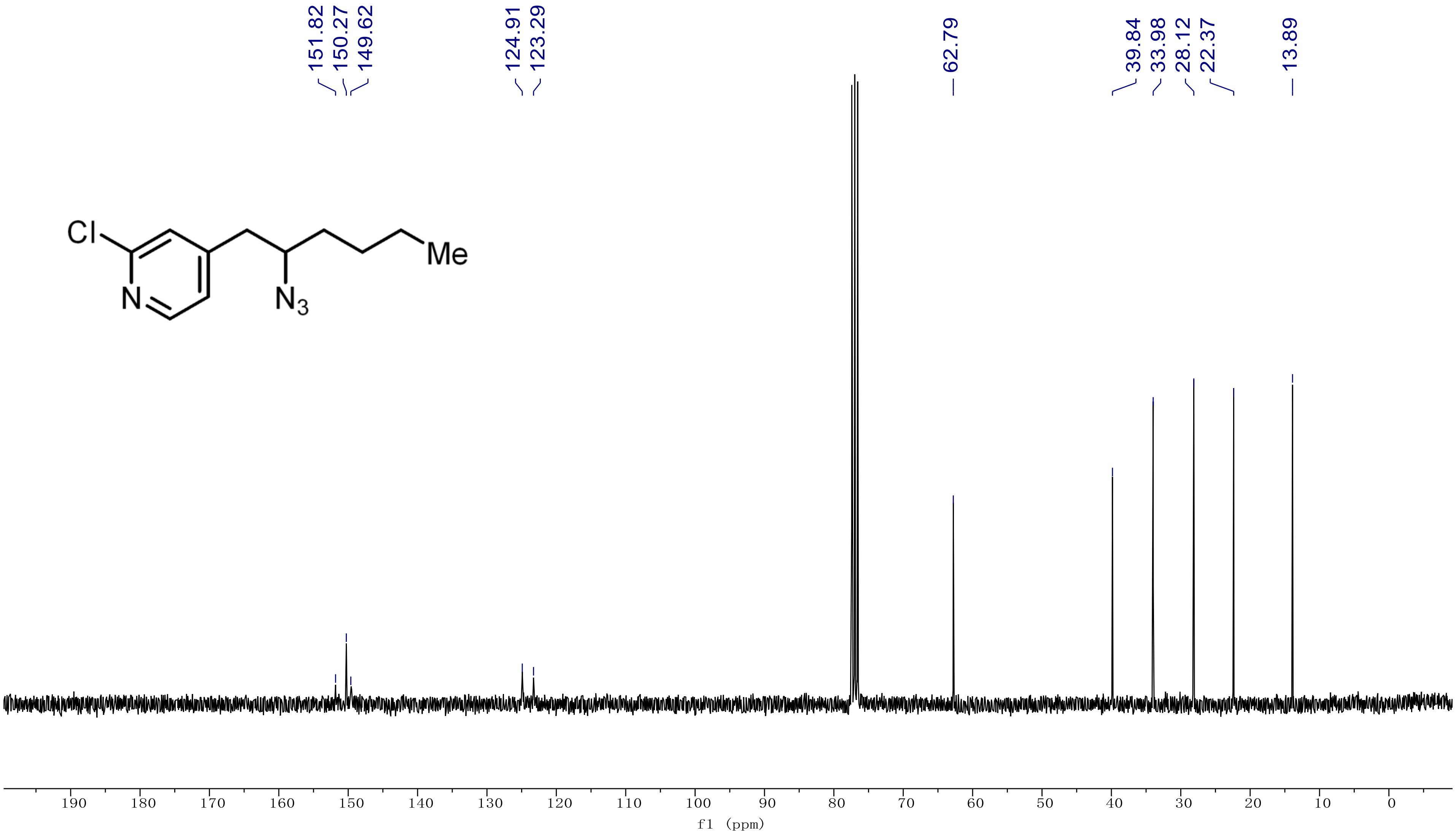

**$^{1}$H NMR of *rac*-pyridinylethylazide 43**

$CDCl_3$, 23 ºC

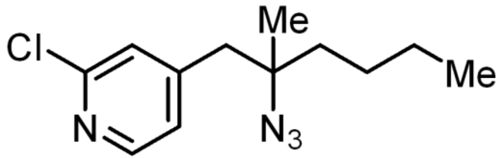


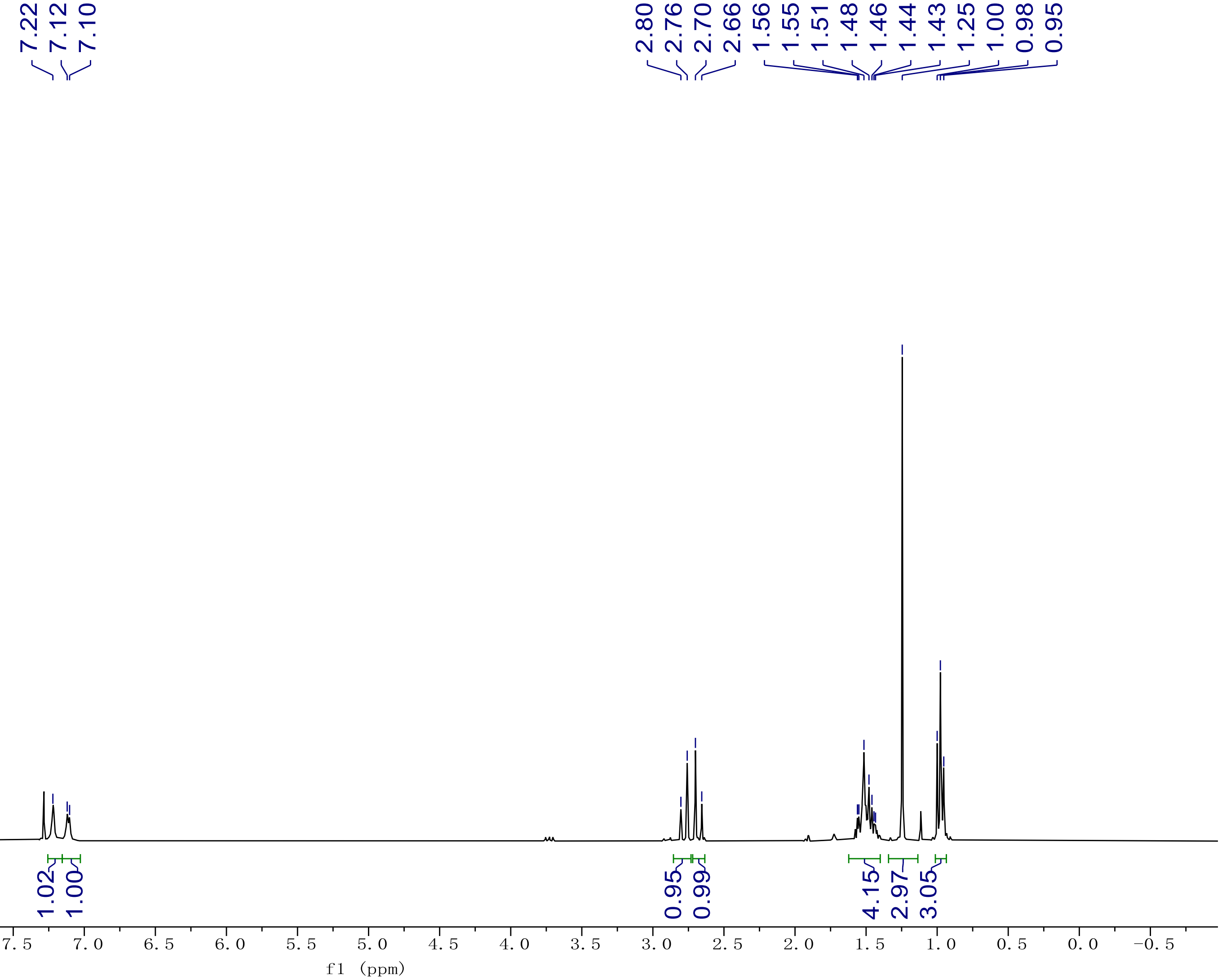

## $^{13}$C NMR of *rac*-pyridinylethylazide 43

$CDCl_3$, 23 ºC

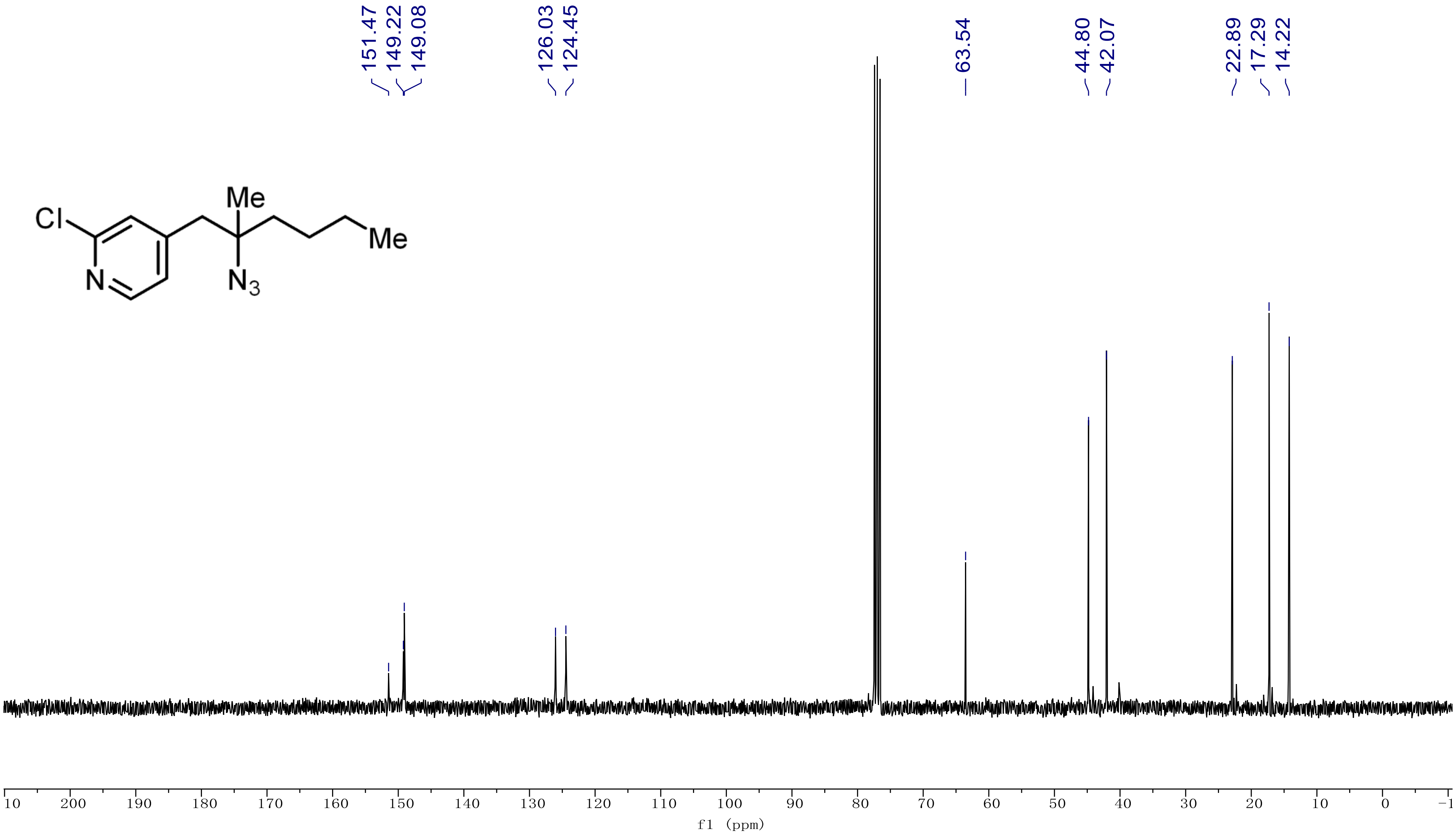

## $^{1}$H NMR of pyridinylethylazide 44

CDCl$_3$, 23 ºC

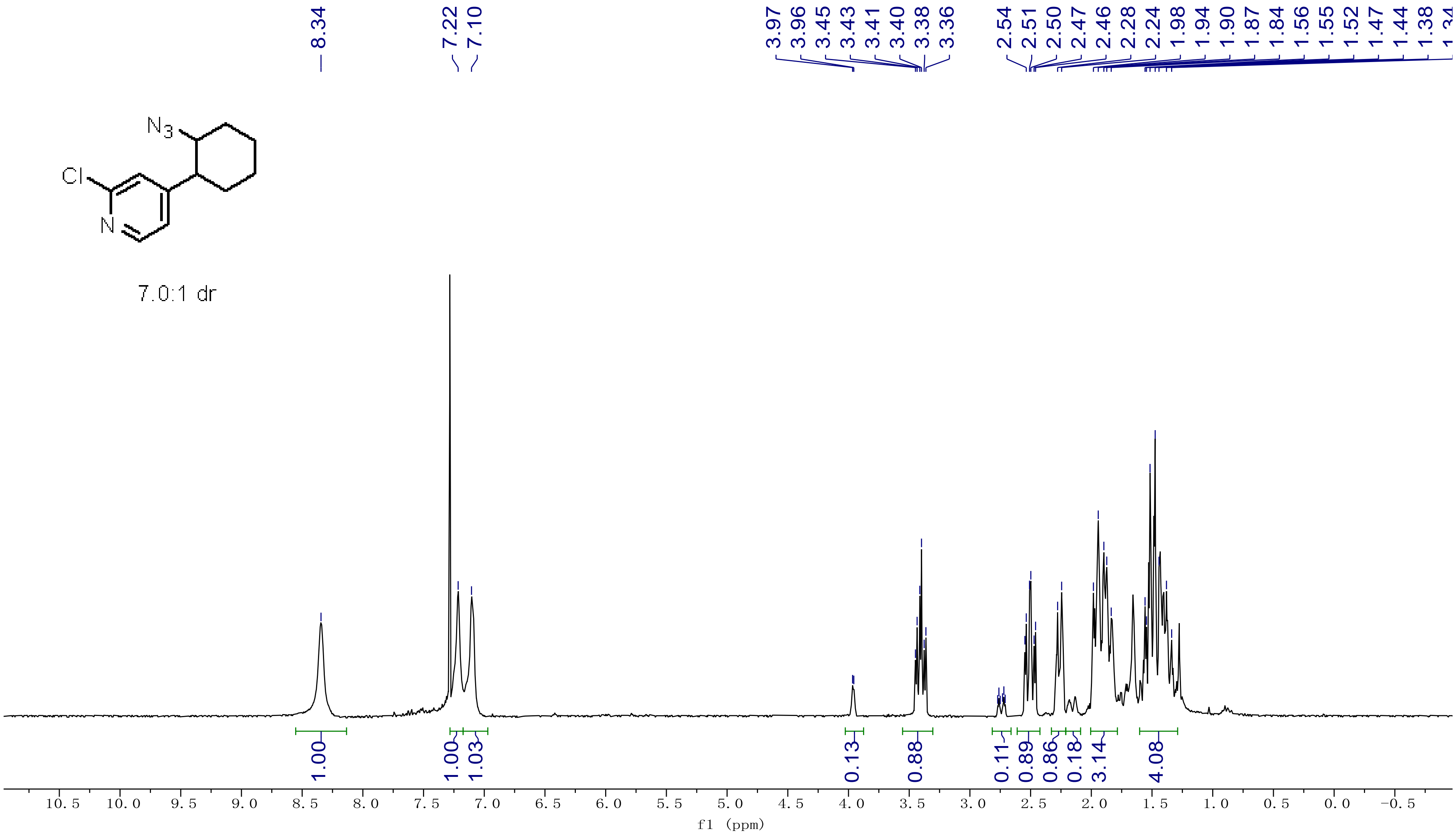

## $^{13}$C NMR of pyridinylethylazide 44

$CDCl_3$, 23 ºC

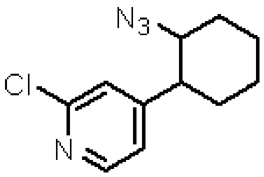


7.0:1 dr

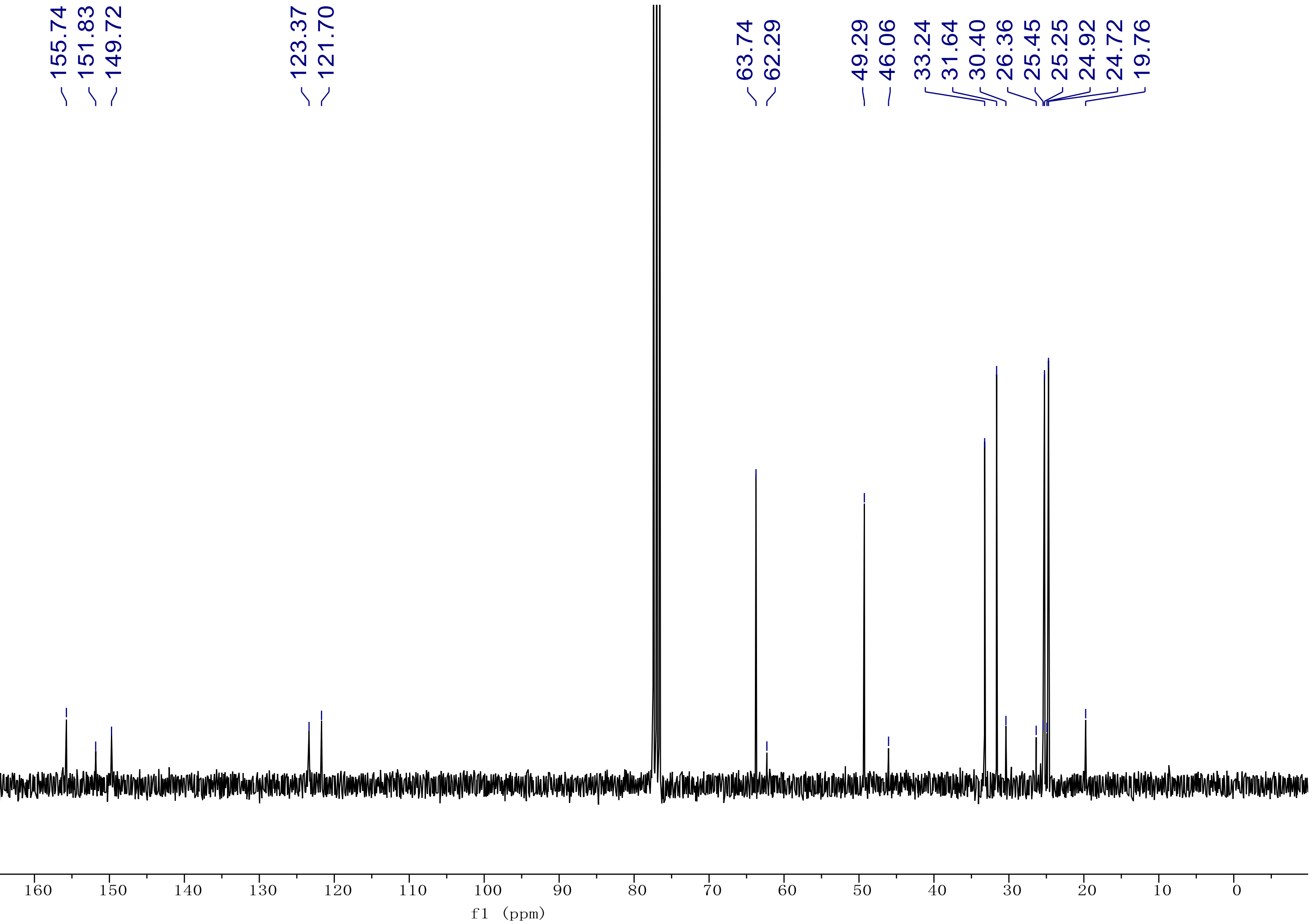

**$^{1}$H NMR of *rac*-pyridinylethylazide 45**

$CDCl_3$, 23 ºC

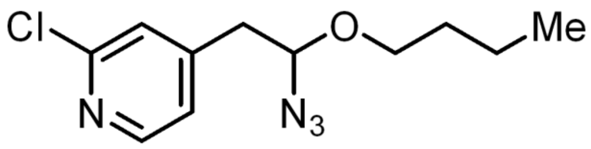


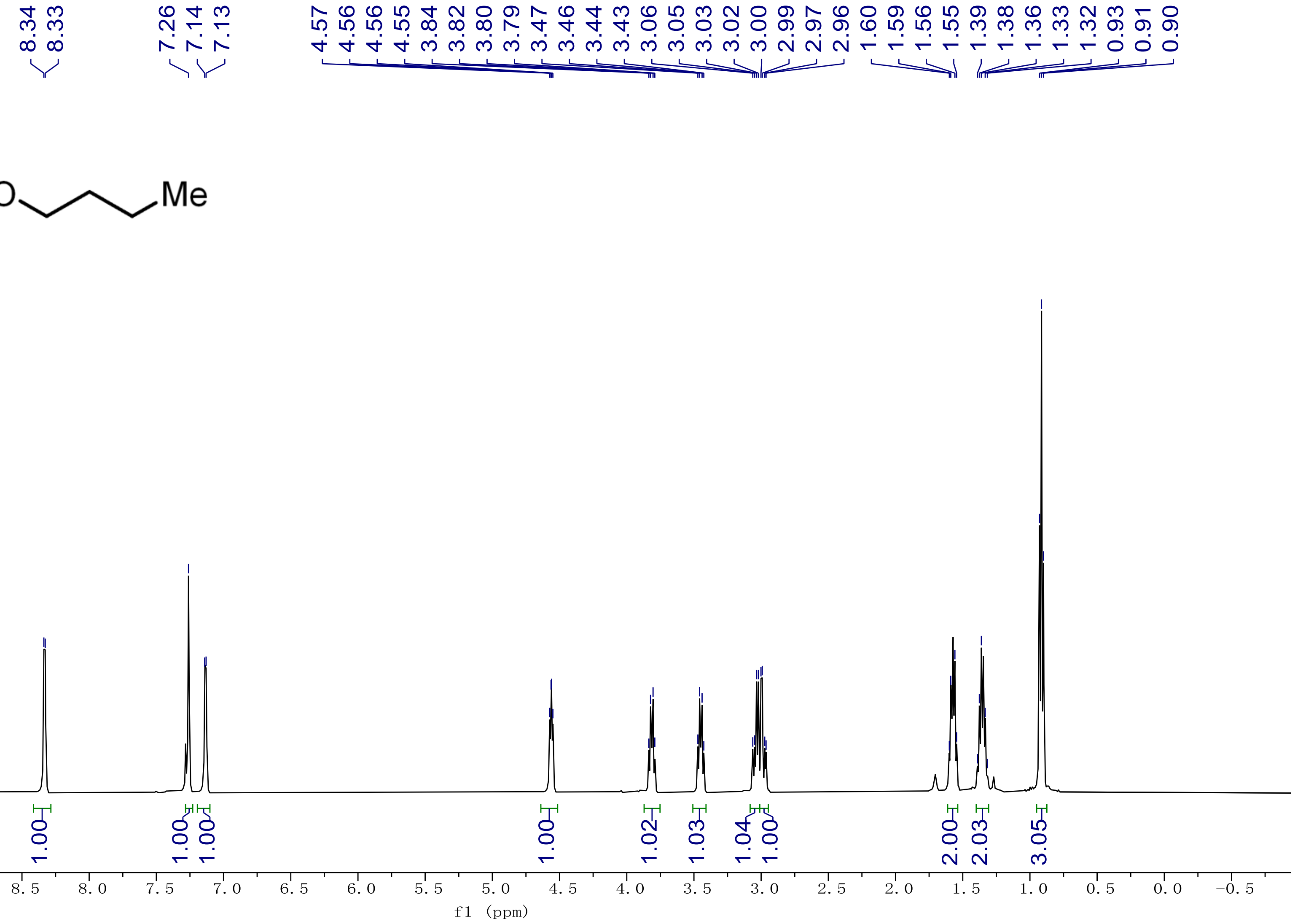

**$^{13}$C NMR of *rac*-pyridinylethylazide 45**

CDCl$_3$, 23 ºC

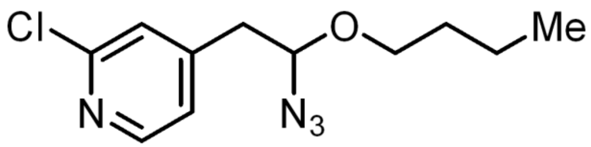


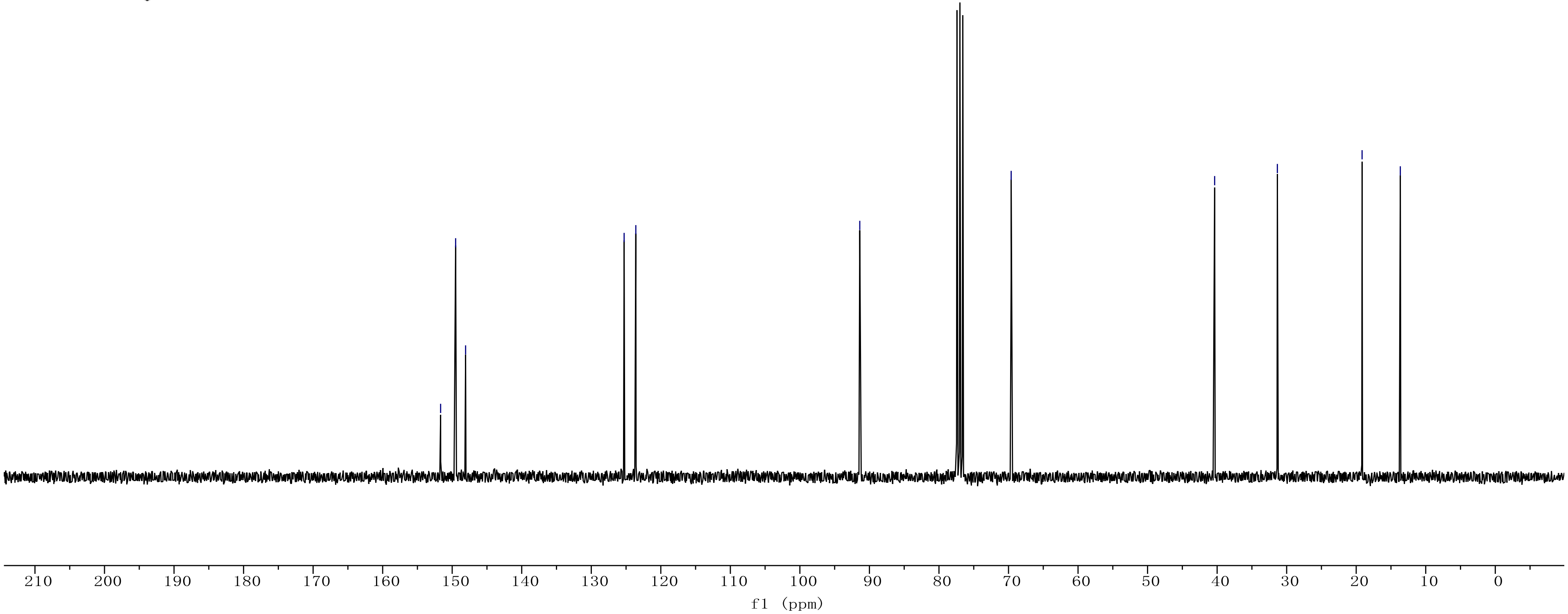

**$^{1}$H NMR of *rac*-pyridinylethylazide 46**

$CDCl_3$, 23 ºC

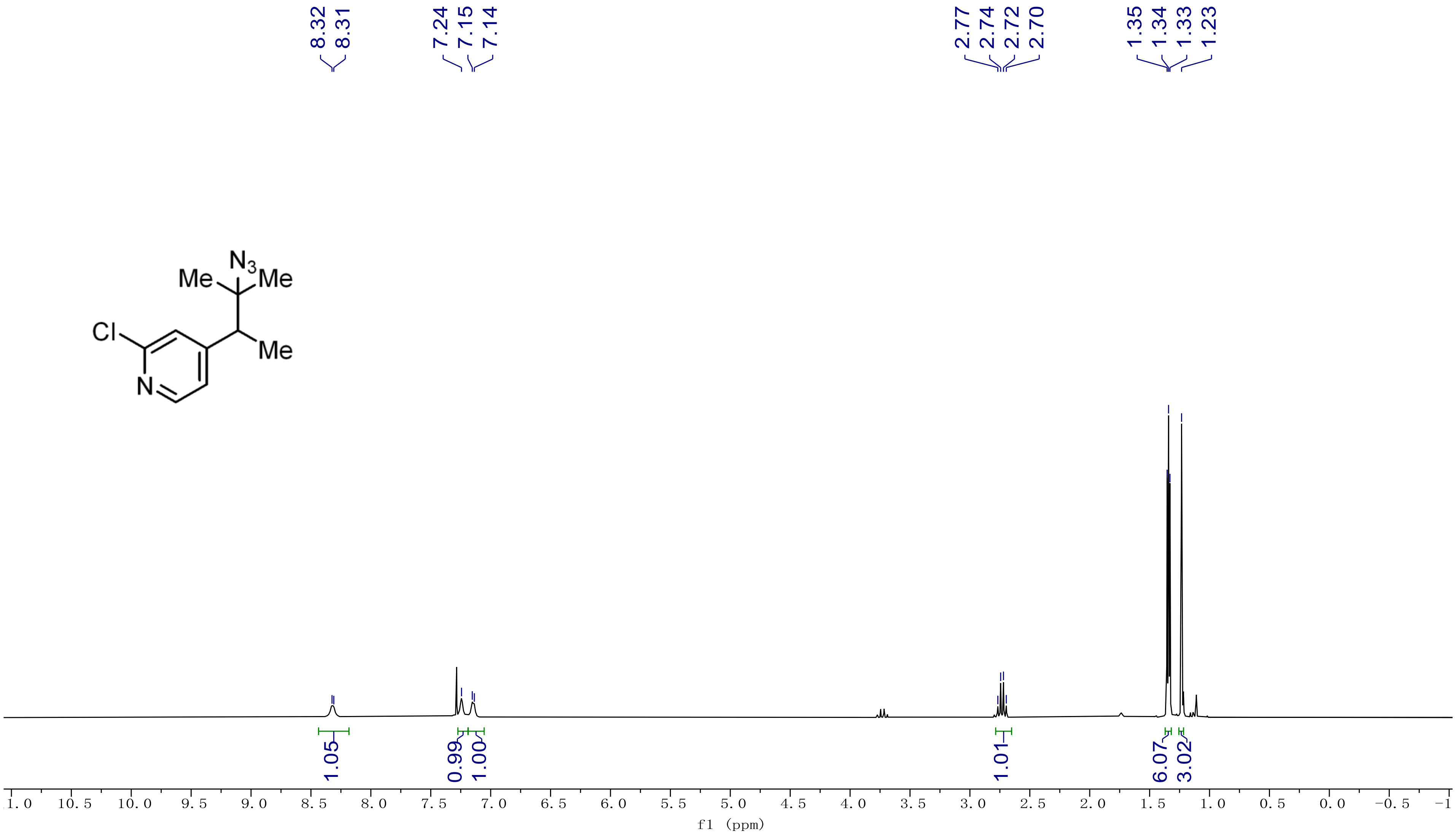

**$^{13}$C NMR of *rac*-pyridinylethylazide 46**

CDCl$_3$, 23 ºC

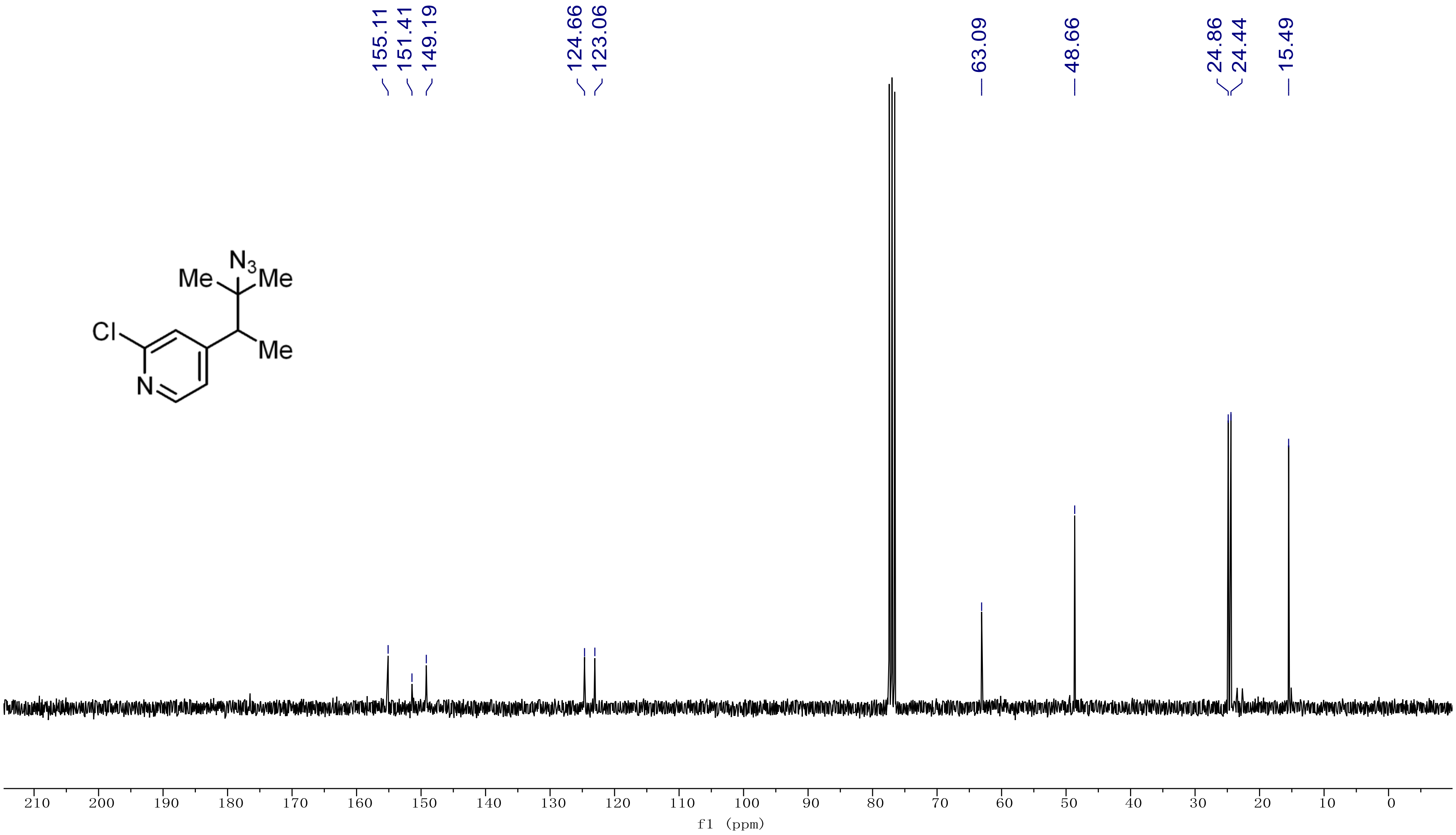

**$^{1}$H NMR of pyridinylethylazide 47**

$CDCl_3$, 23 ºC

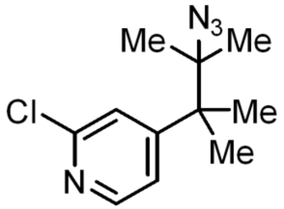


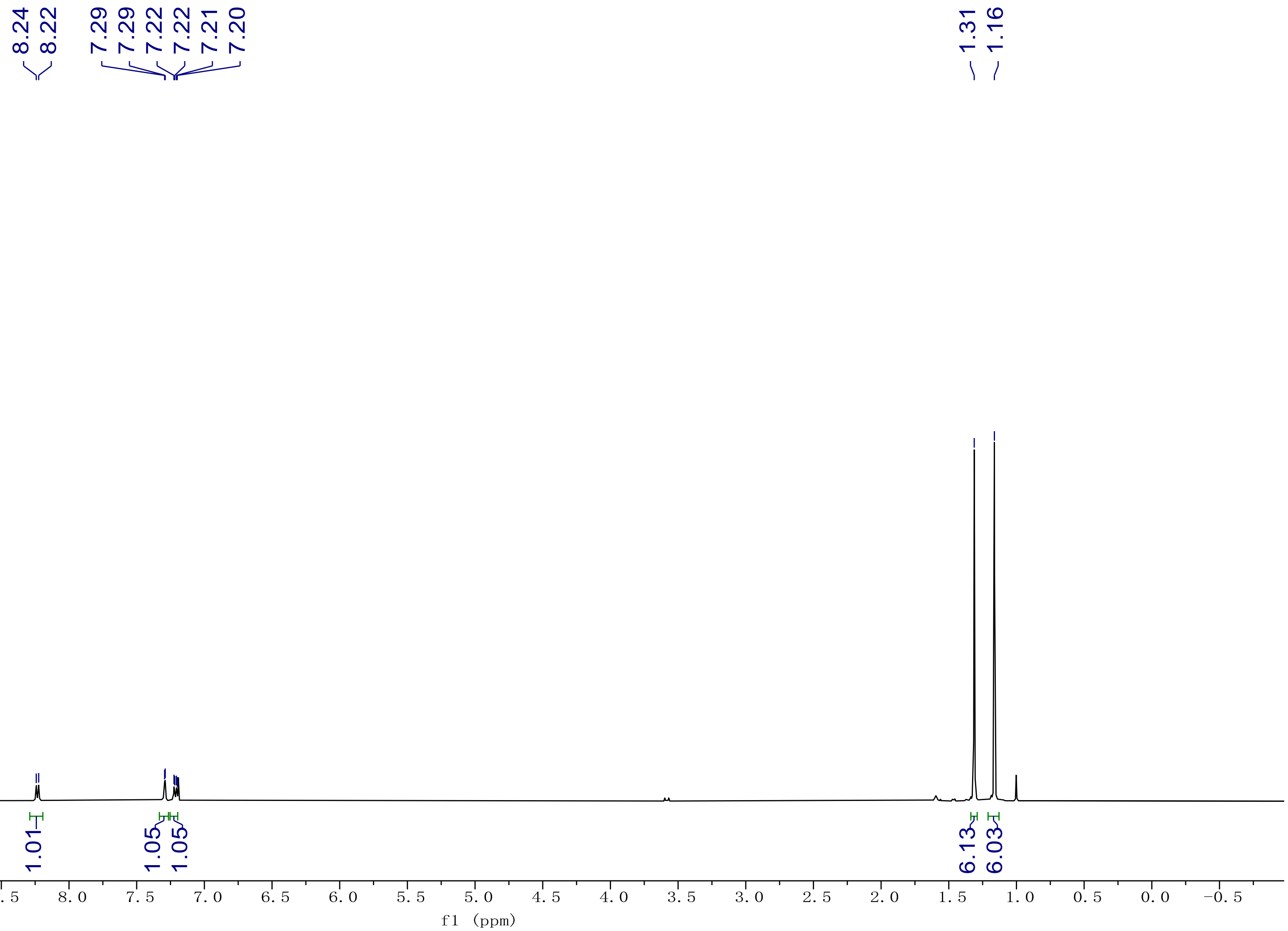

## $^{13}$C NMR of pyridinylethylazide 47

$CDCl_3$, 23 ºC

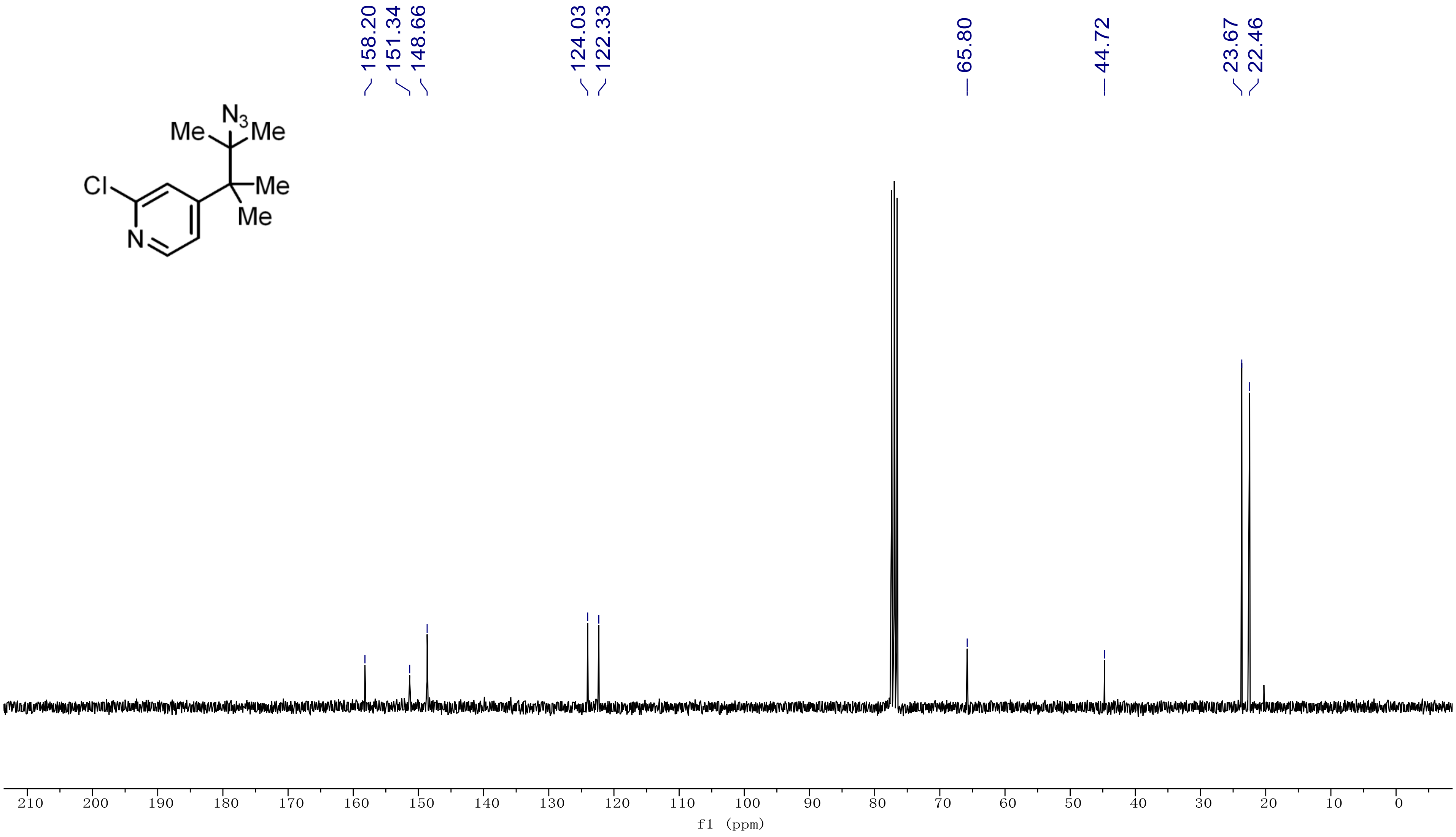

**$^{1}$H NMR of nootkatone-derived pyridinylethylazide 48**

CDCl3, 23 ºC

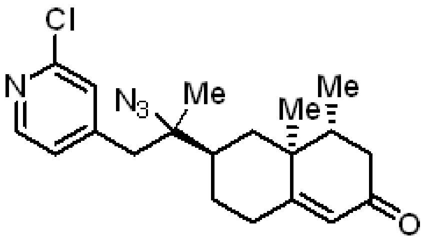

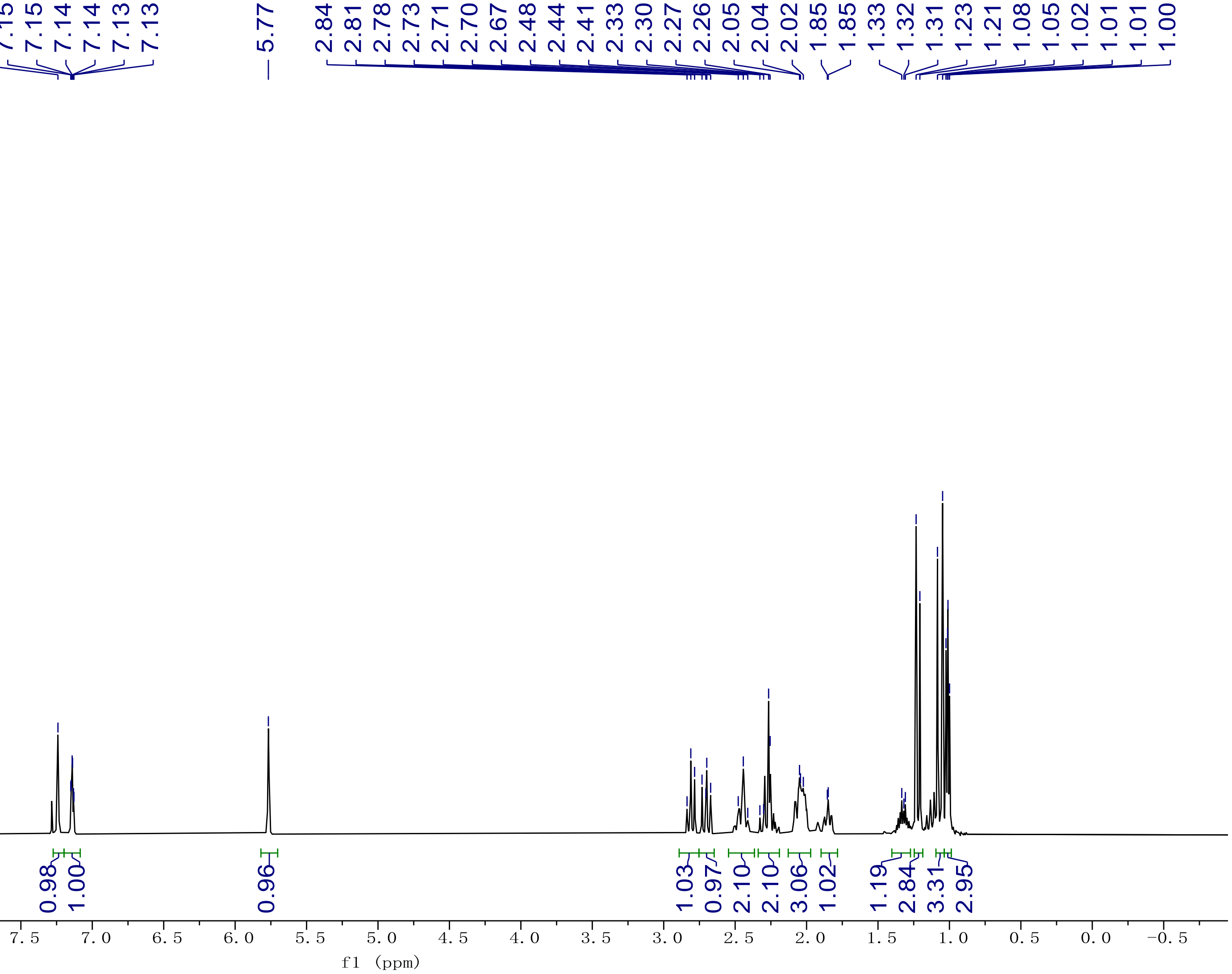

**$^{13}$C NMR of nootkatone-derived pyridinylethylazide 48**

CDCl$_3$, 23 ºC

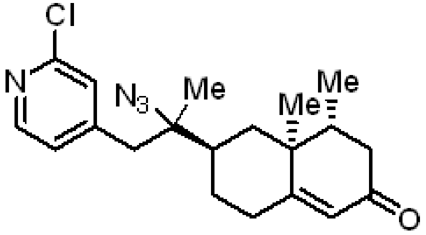


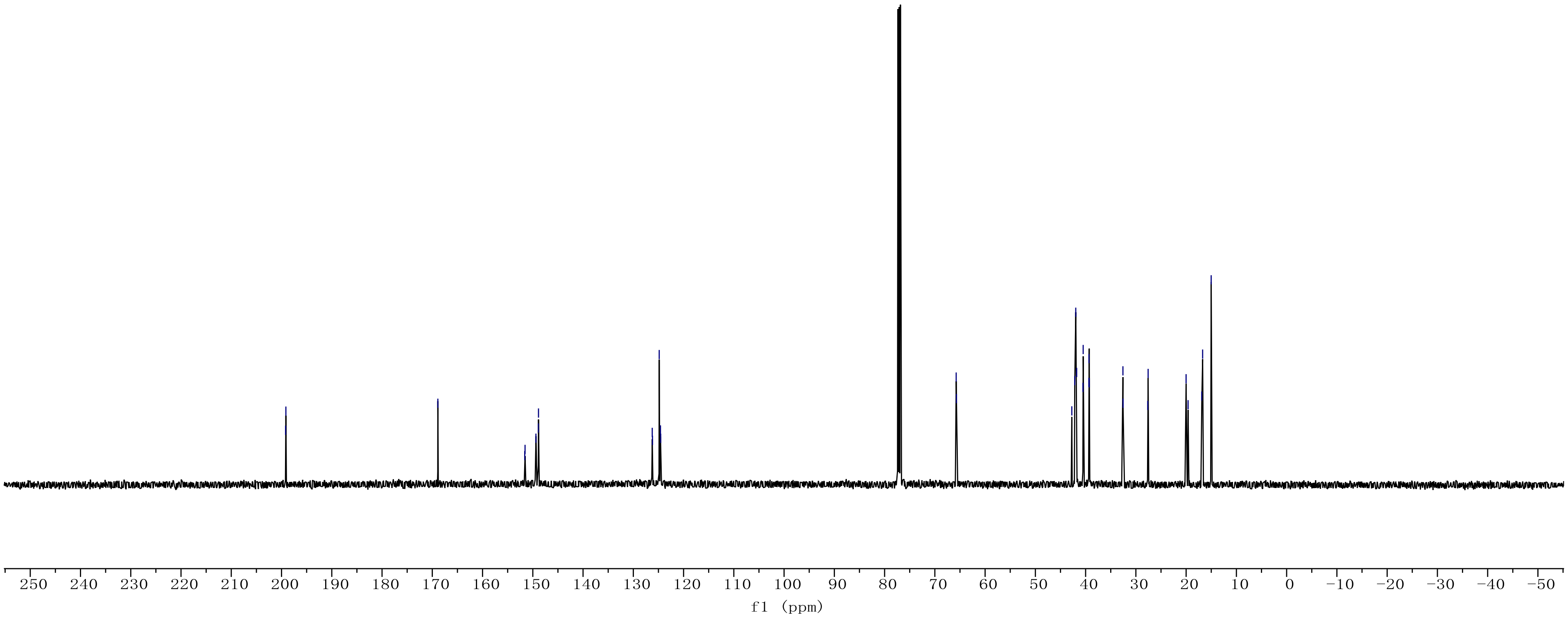

## $^{1}$H NMR of phenylethylazide 49

CDCl3, 23 ºC

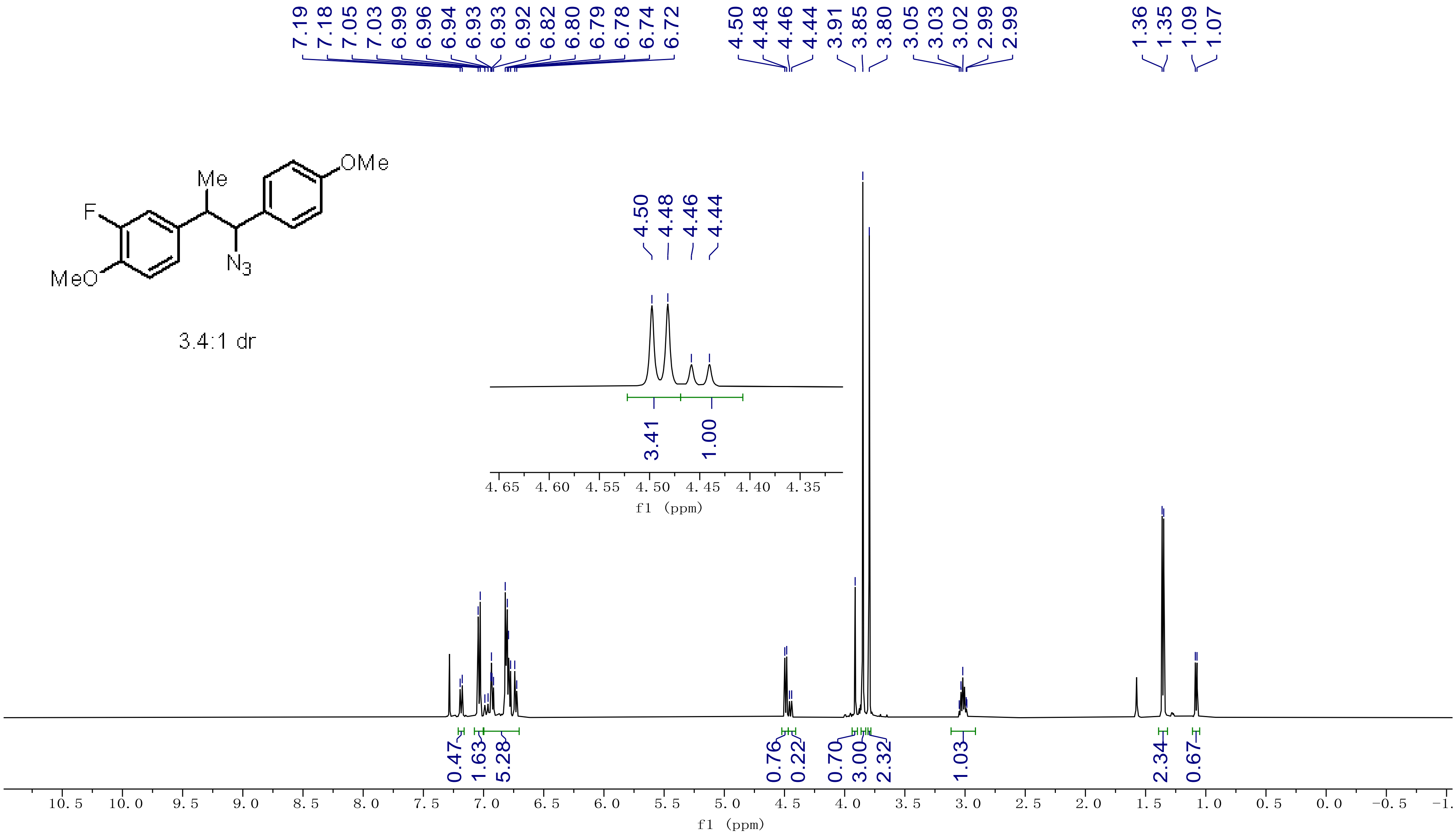

# $^{19}$F NMR of phenylethylazide 49

CDCl$_3$, 23 ºC

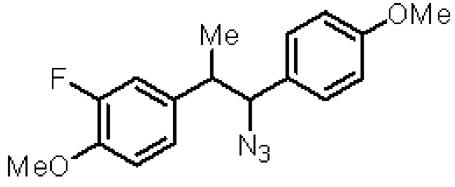


3.4:1 dr

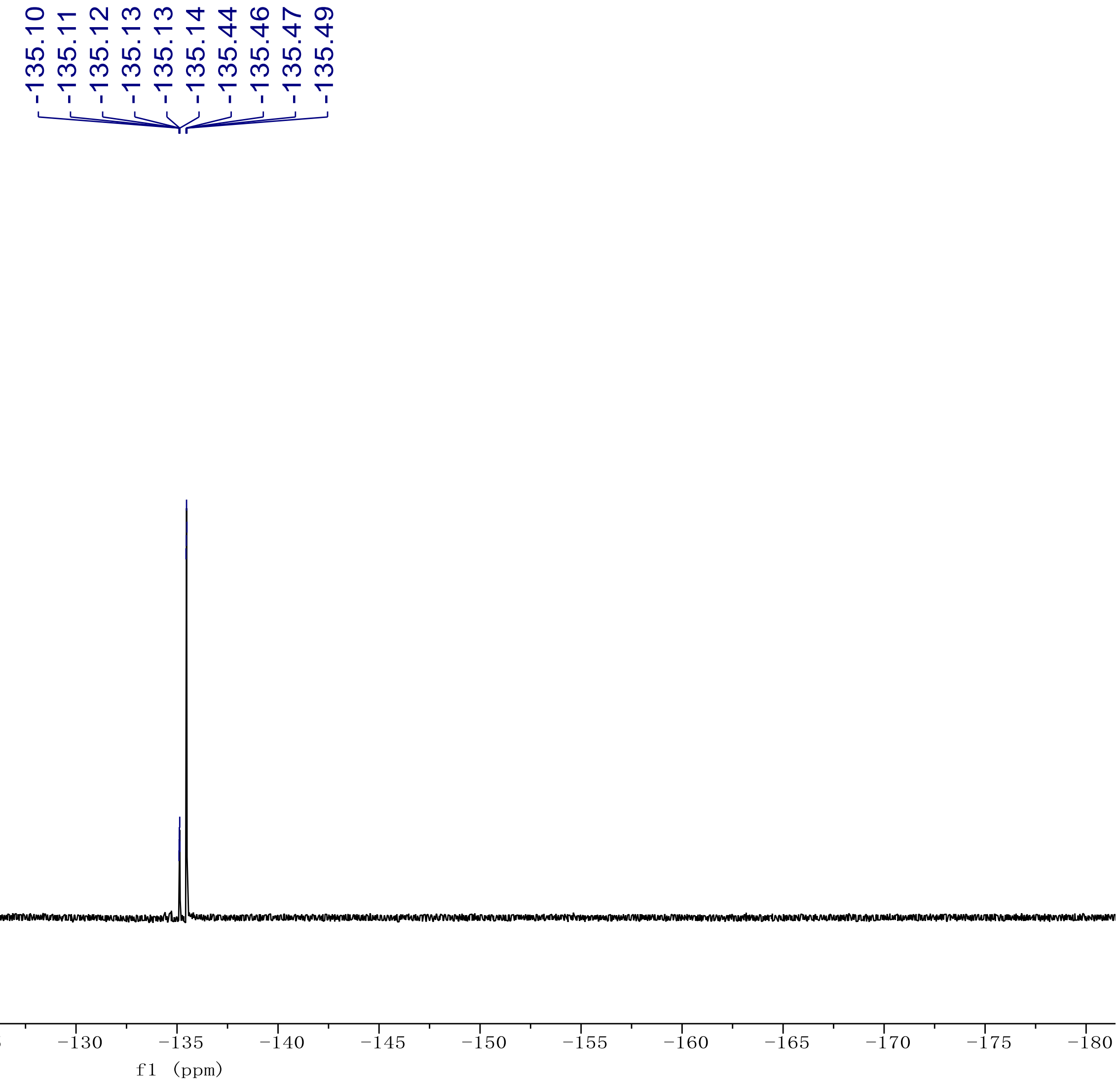

## $^{13}$C NMR of phenylethylazide 49

$CDCl_3$, 23 ºC

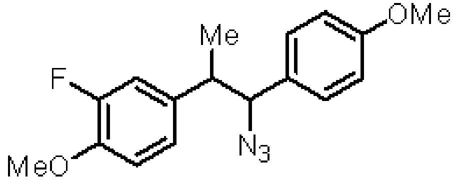


3.4:1 dr

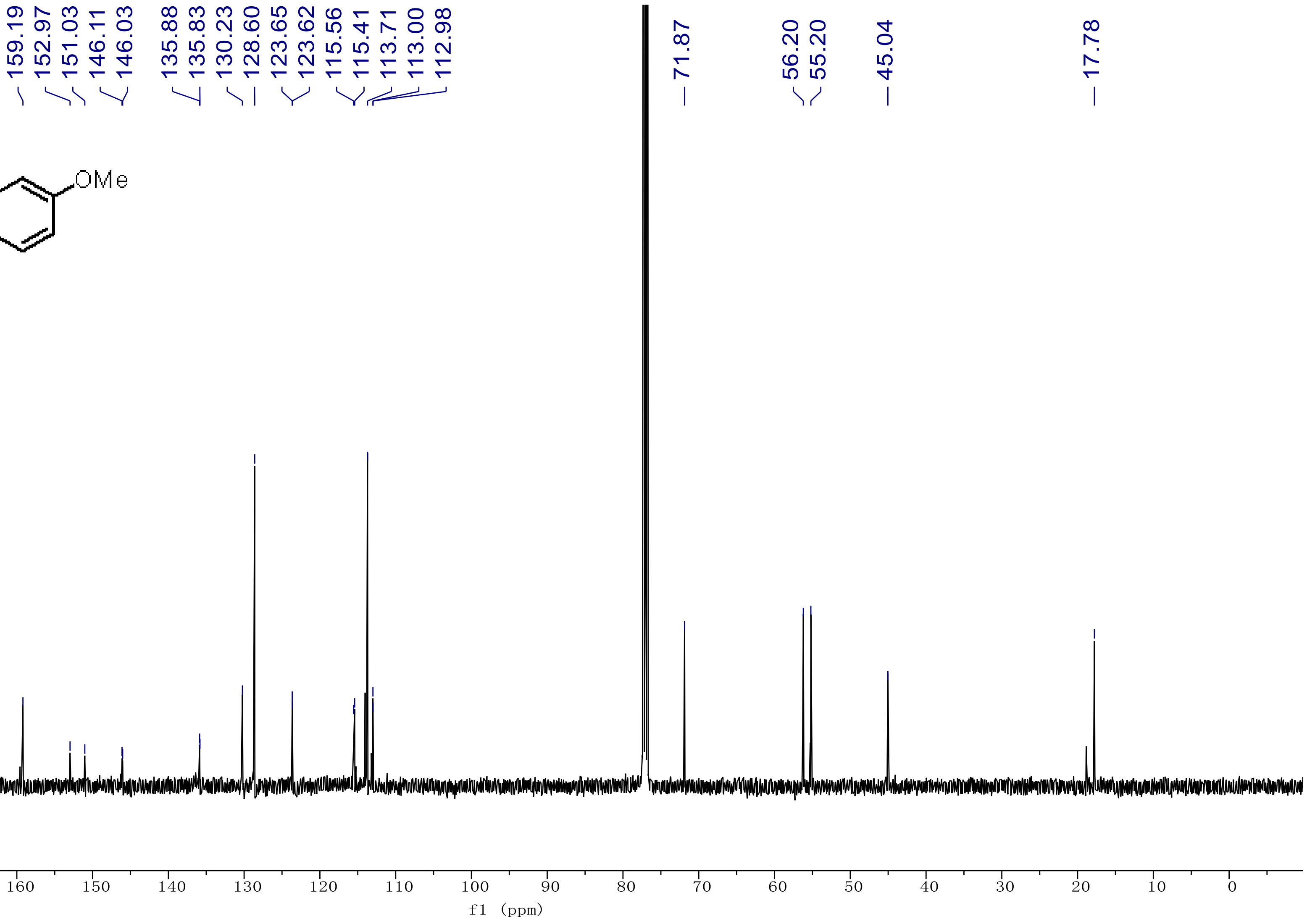

## $^{1}$H NMR of *rac*-phenylethylazide 50

$CDCl_3$, 23 ºC

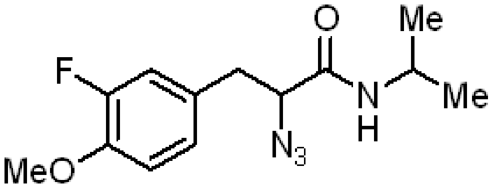


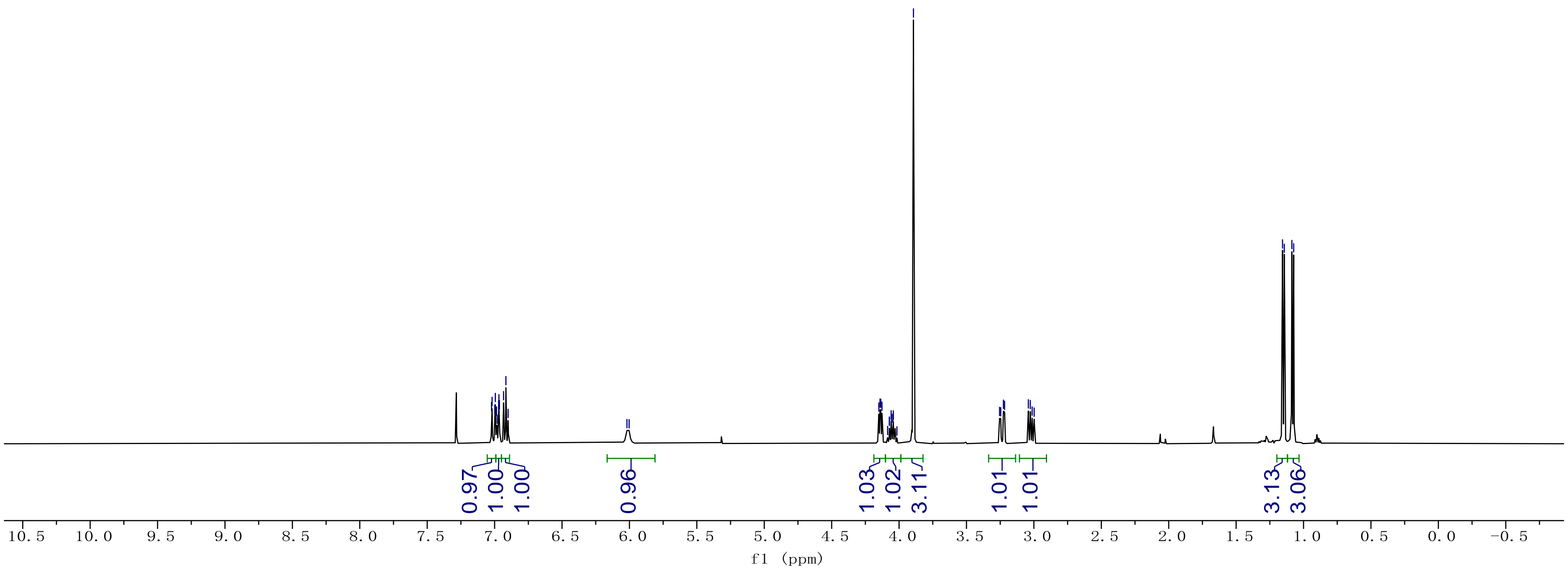

**$^{19}$F NMR of *rac*-phenylethylazide 50**

$CDCl_3$, 23 ºC

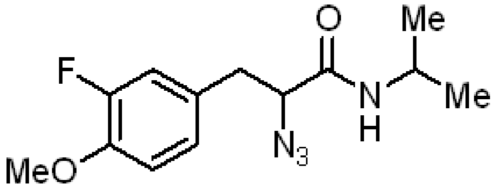


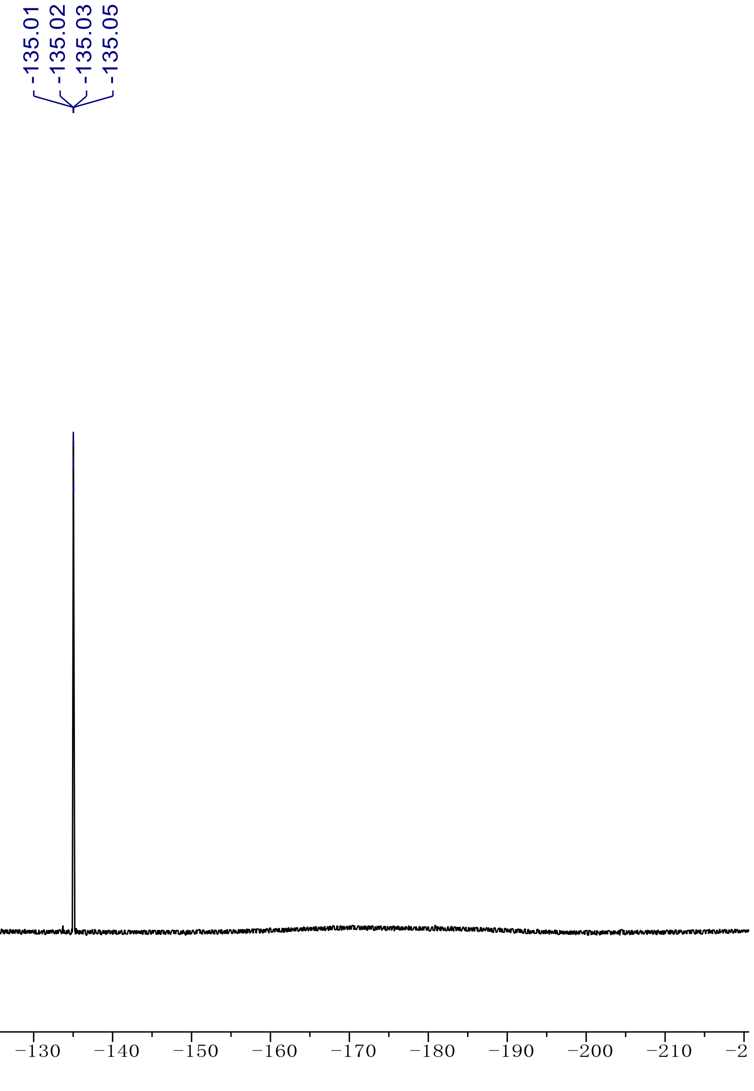

**$^{13}$C NMR of *rac*-phenylethylazide 50**

$CDCl_3$, 23 ºC

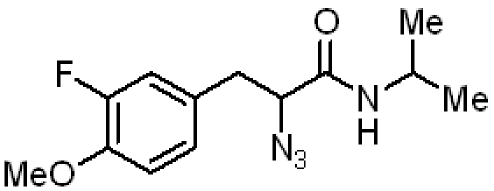

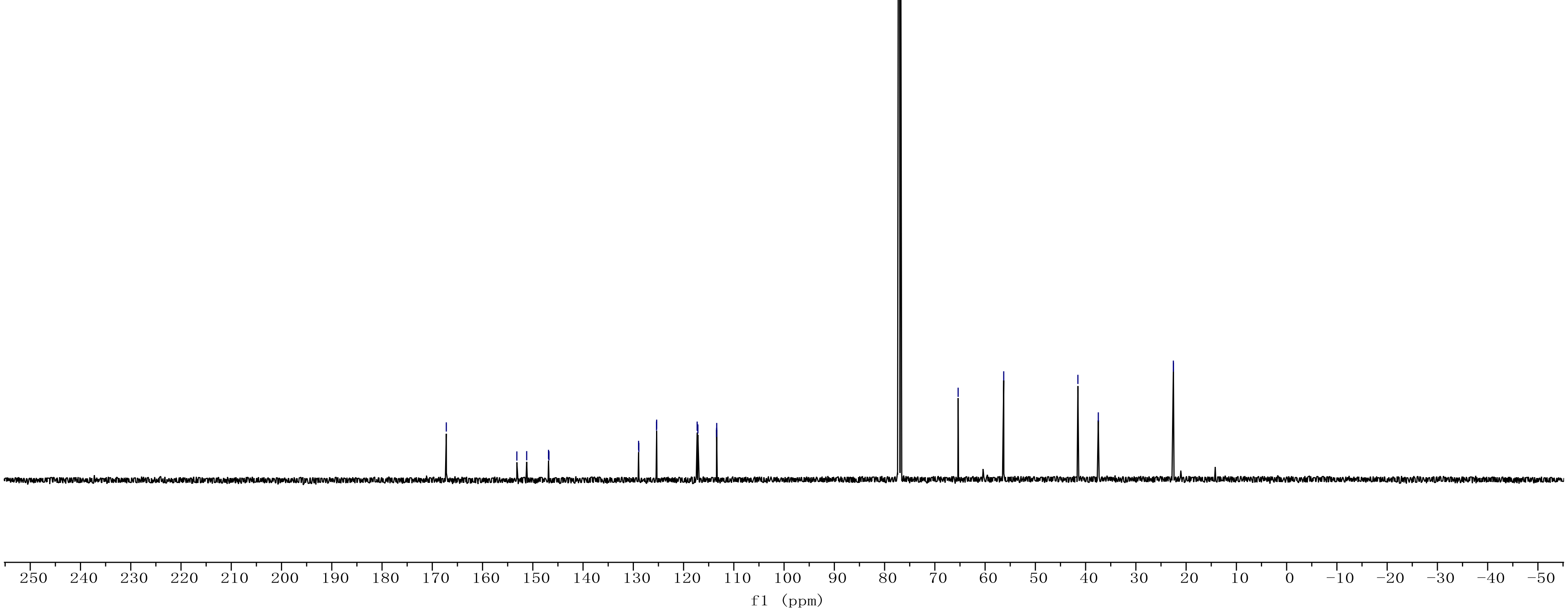

## $^1$H NMR of valinate-derived phenylethylazide 51

CDCl$_3$, 23 ºC

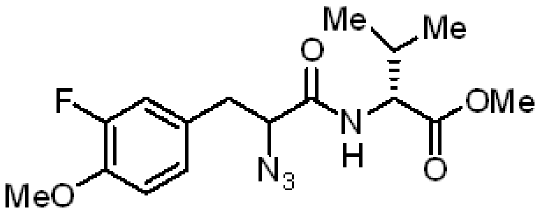


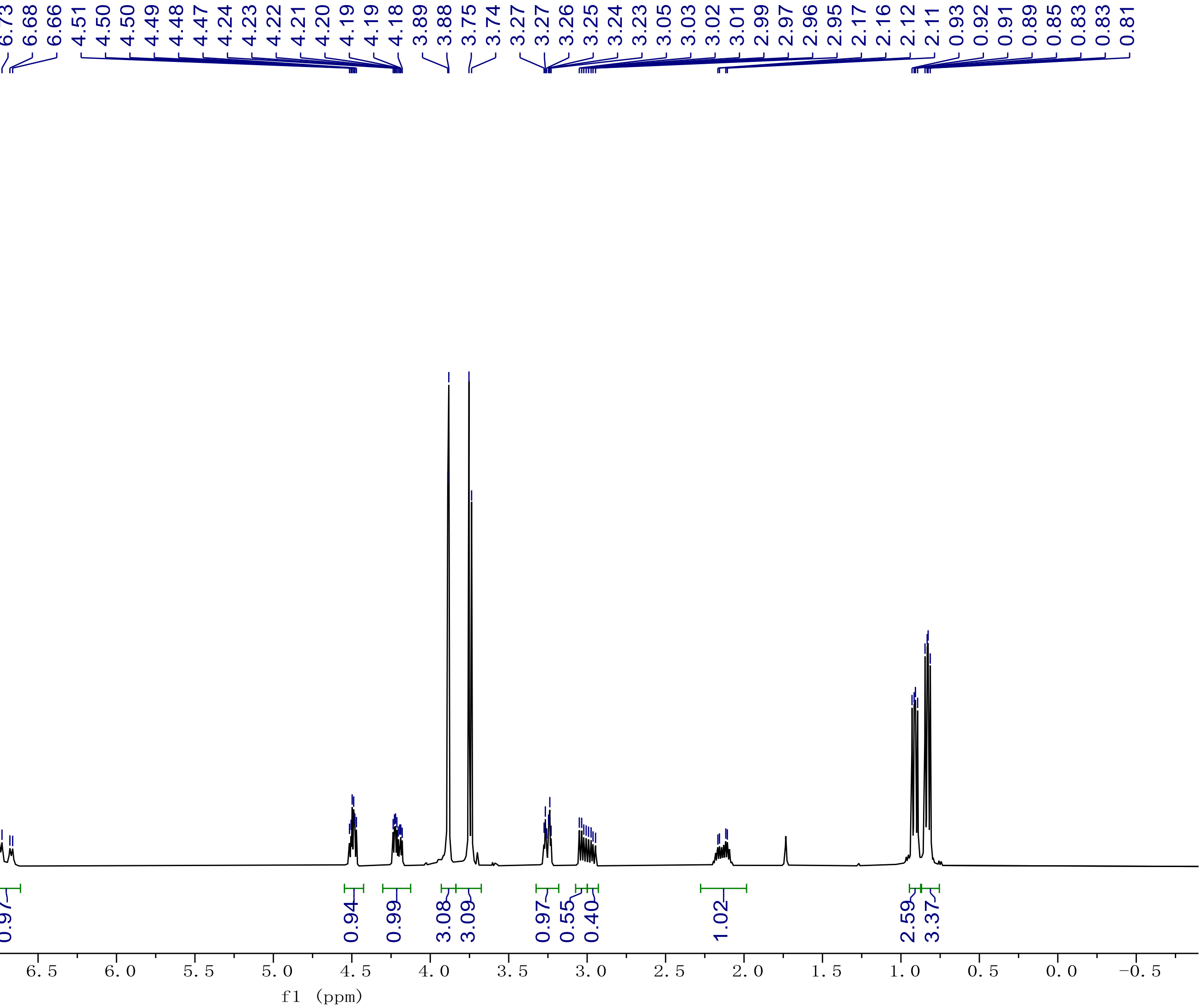

## $^{19}$F NMR of valinate-derived phenylethylazide 51

$CDCl_3$, 23 ºC

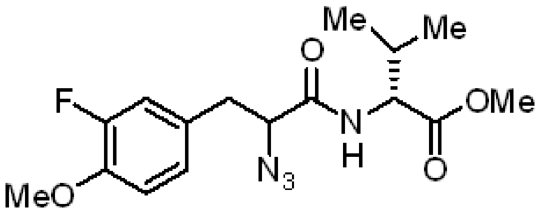


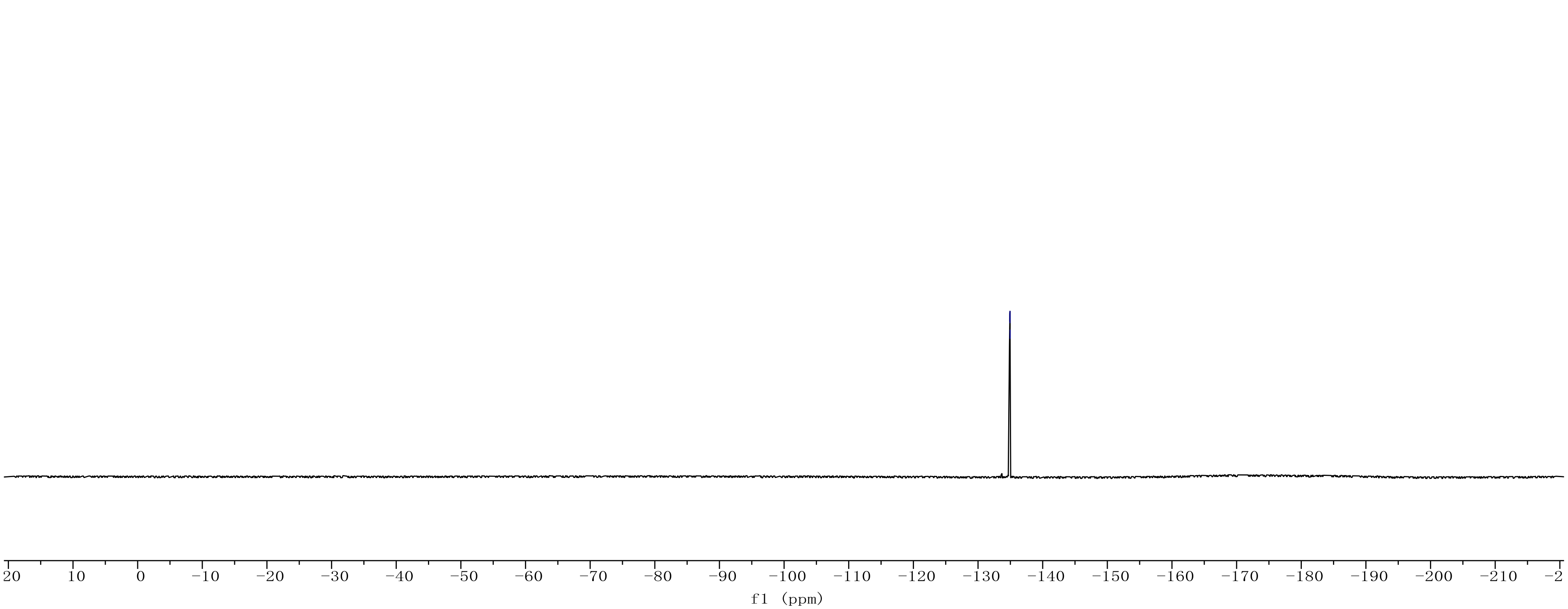

## $^{13}$C NMR of valinate-derived phenylethylazide 51

$CDCl_3$, 23 ºC

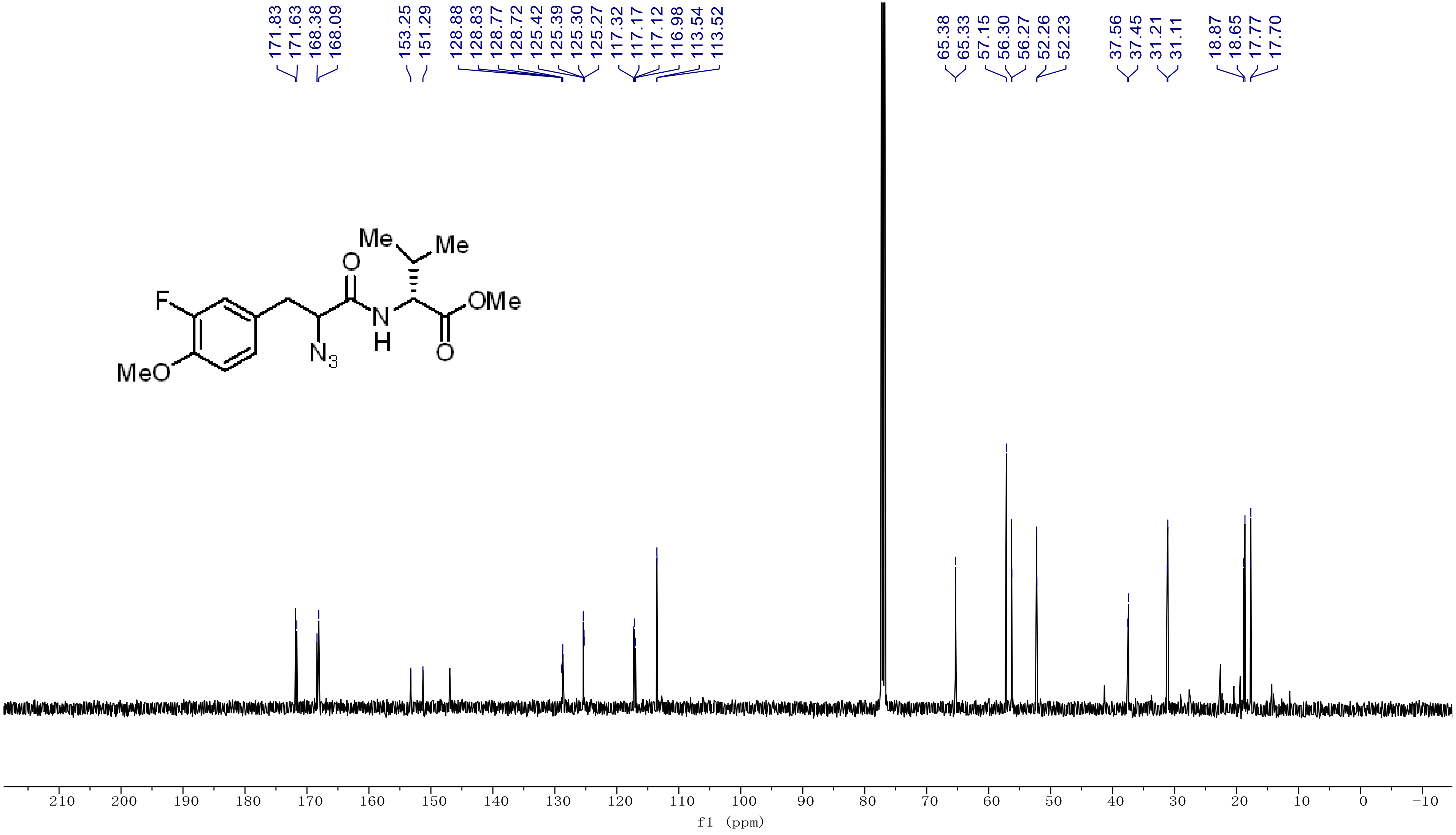

## $^{1}$H NMR of *rac*-fenofibrate-derived phenylethylazide 52

$CDCl_3$, 23 ºC

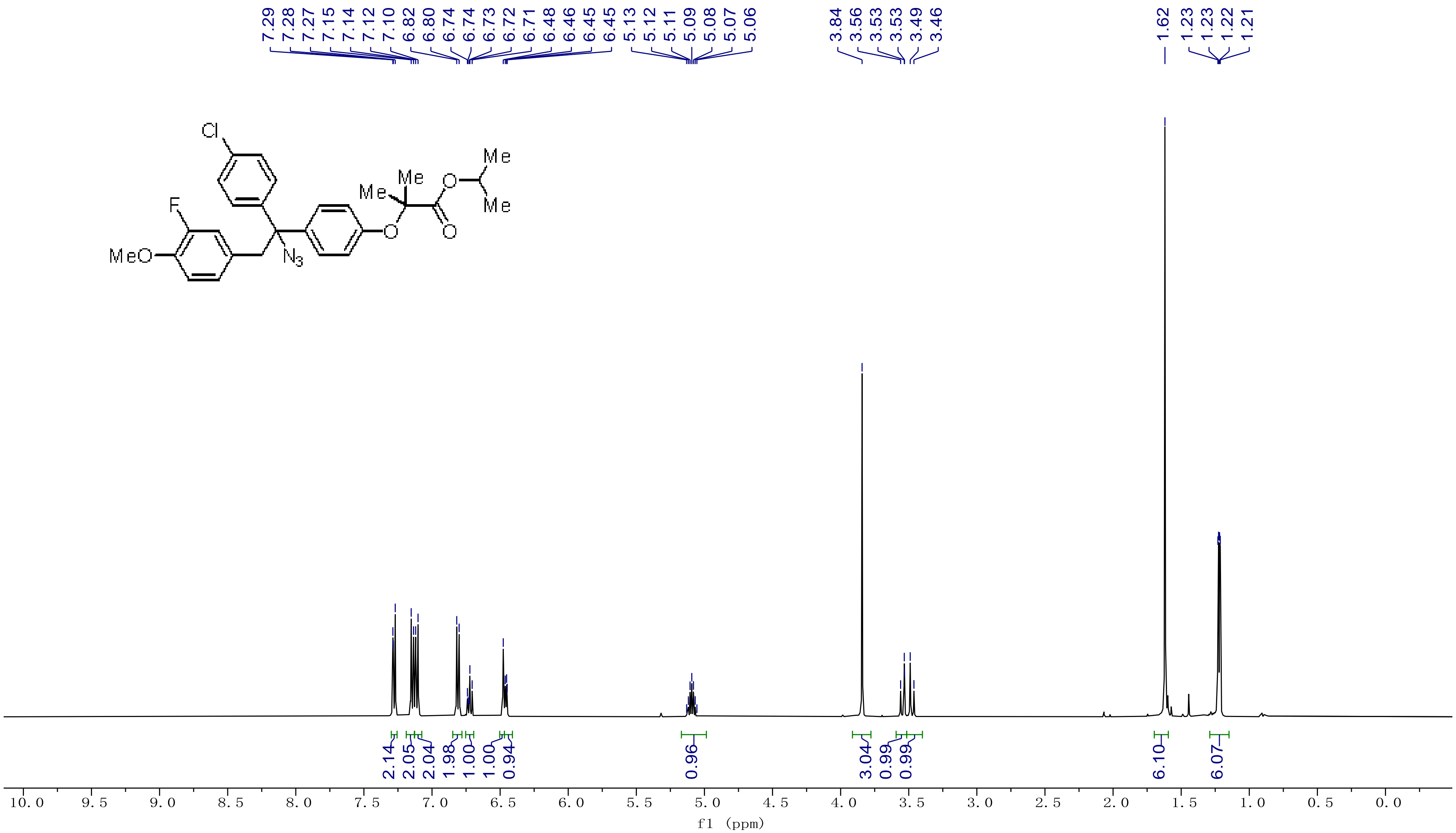

**$^{19}$F NMR of *rac*-fenofibrate-derived phenylethylazide 52**

$CDCl_3$, 23 ºC

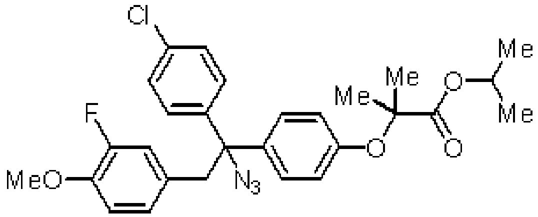


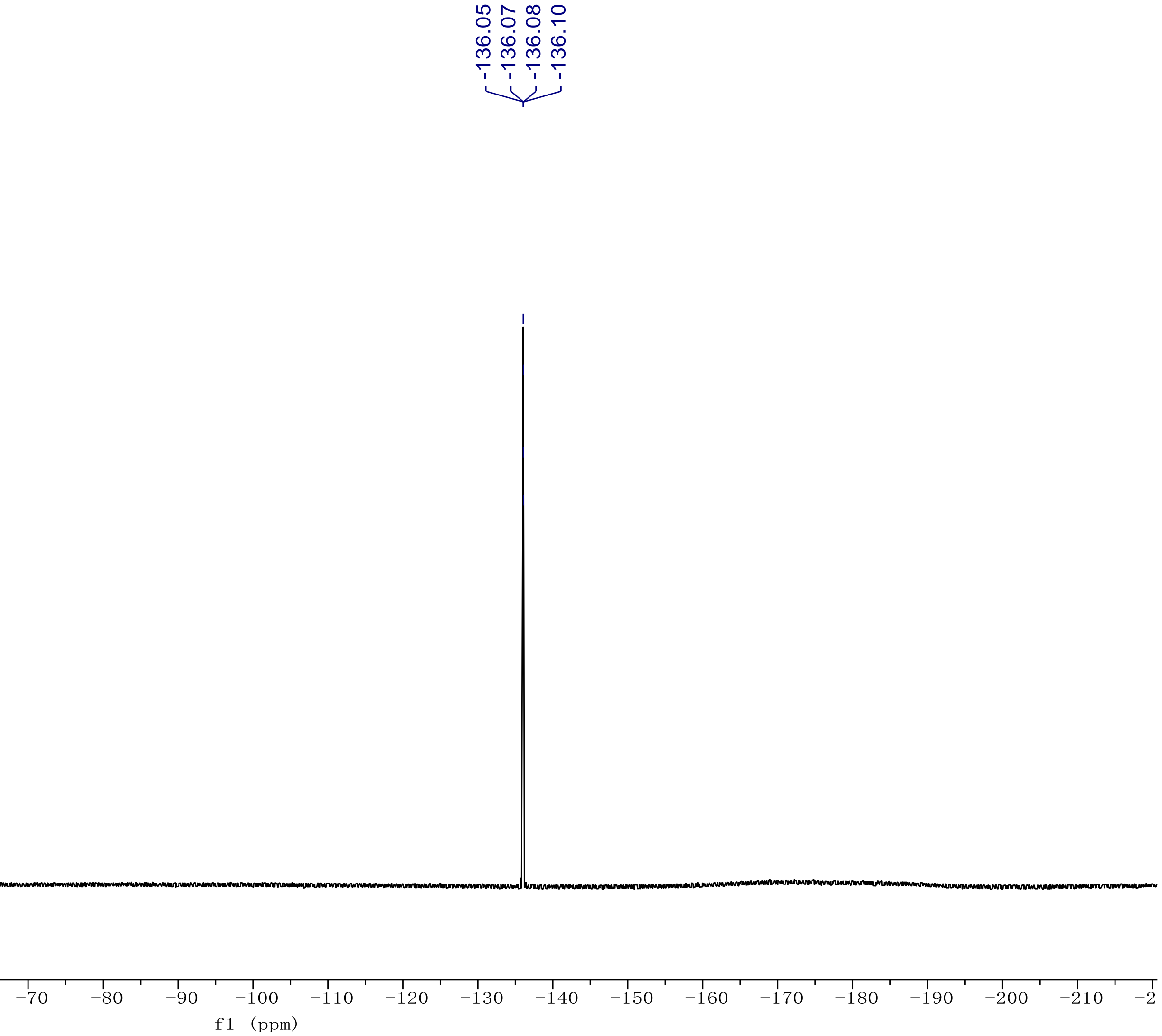

**$^{13}$C NMR of *rac*-fenofibrate-derived phenylethylazide 52**

CDCl$_3$, 23 ºC

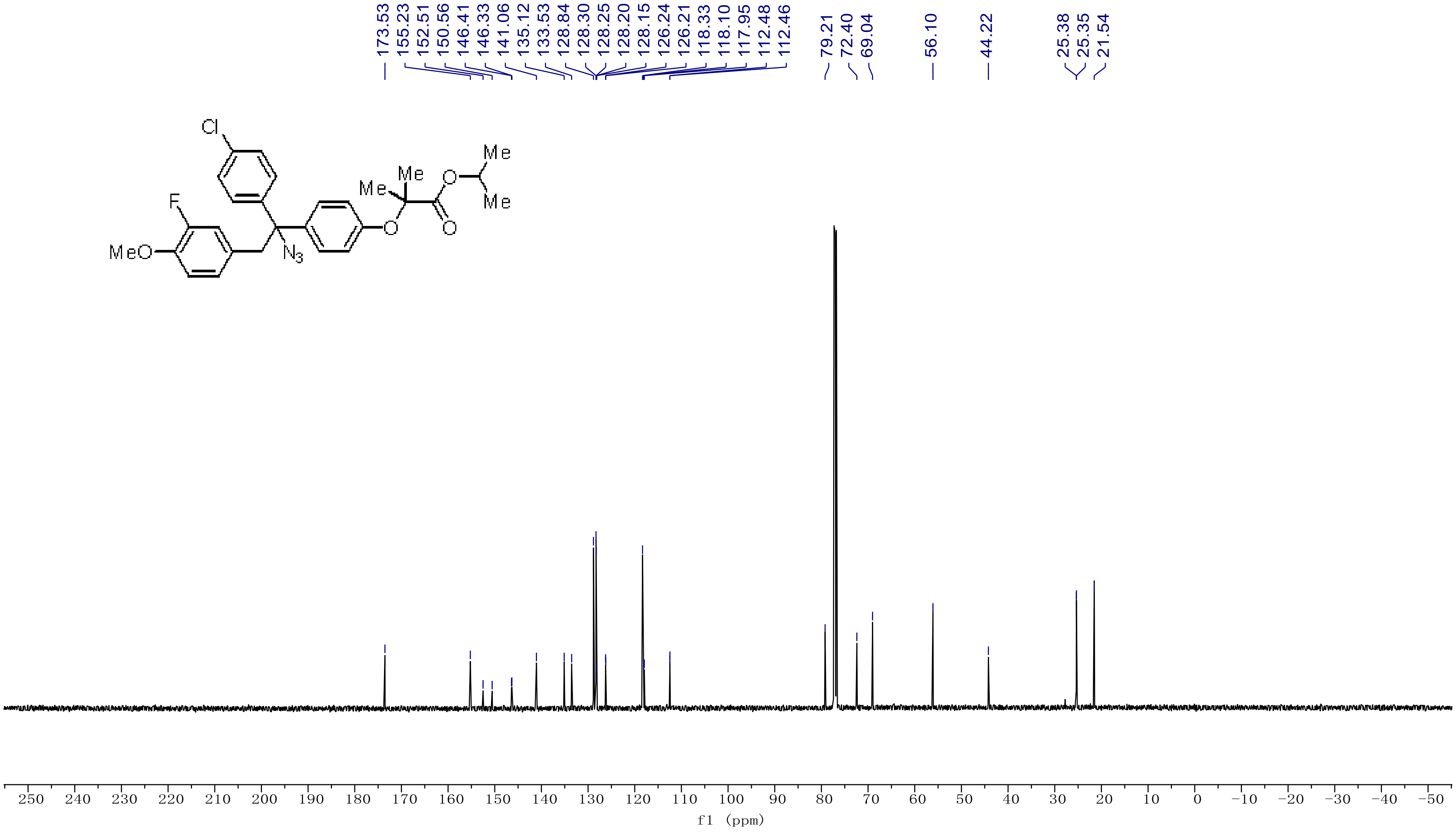

**$^{1}$H NMR of aniline-derived thianthrenium salt 54**

$CDCl_3$, 23 ºC

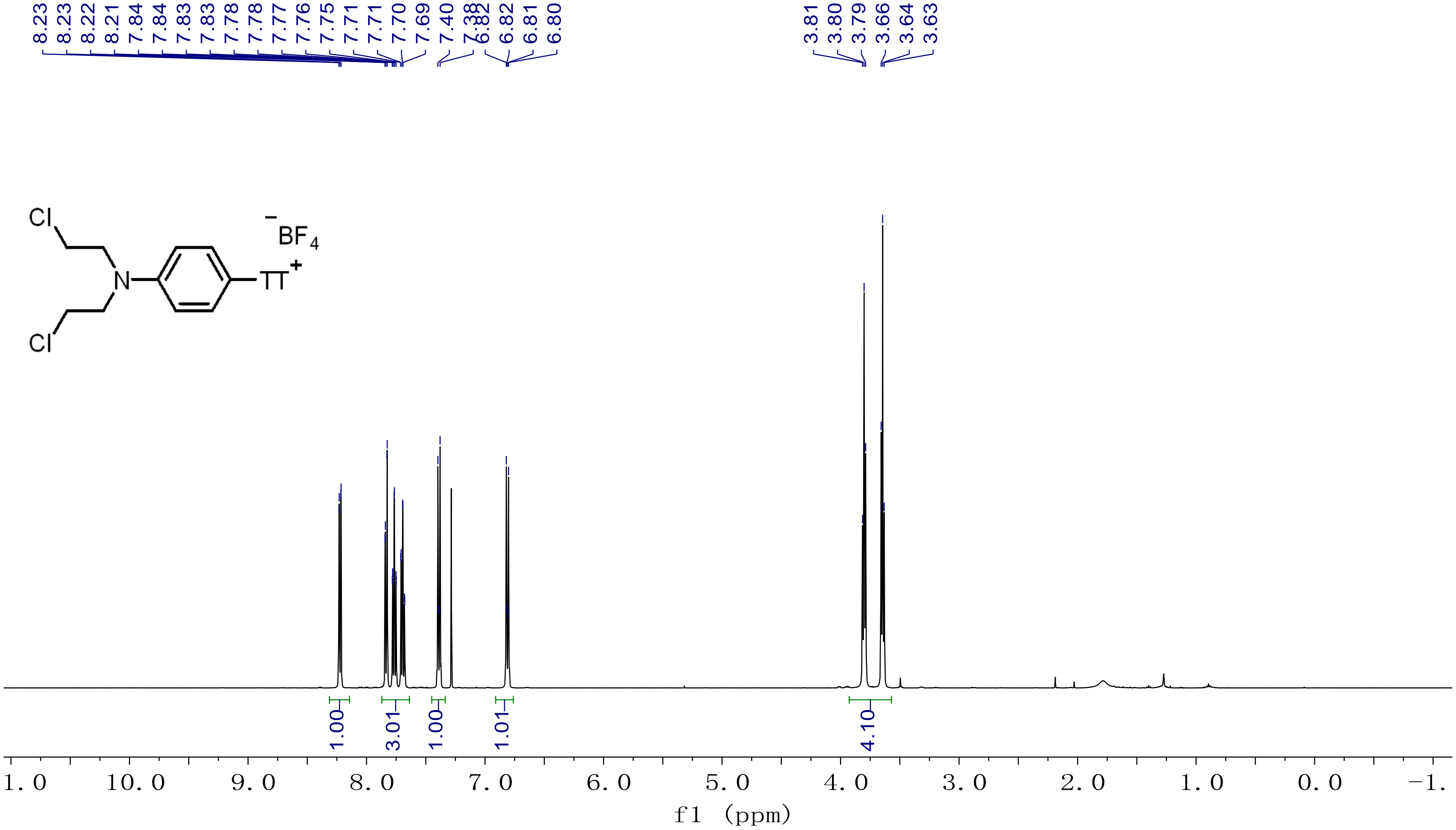

**$^{19}$F NMR of aniline-derived thianthrenium salt 54**

$CDCl_3$, 23 ºC

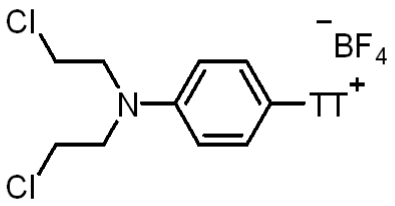


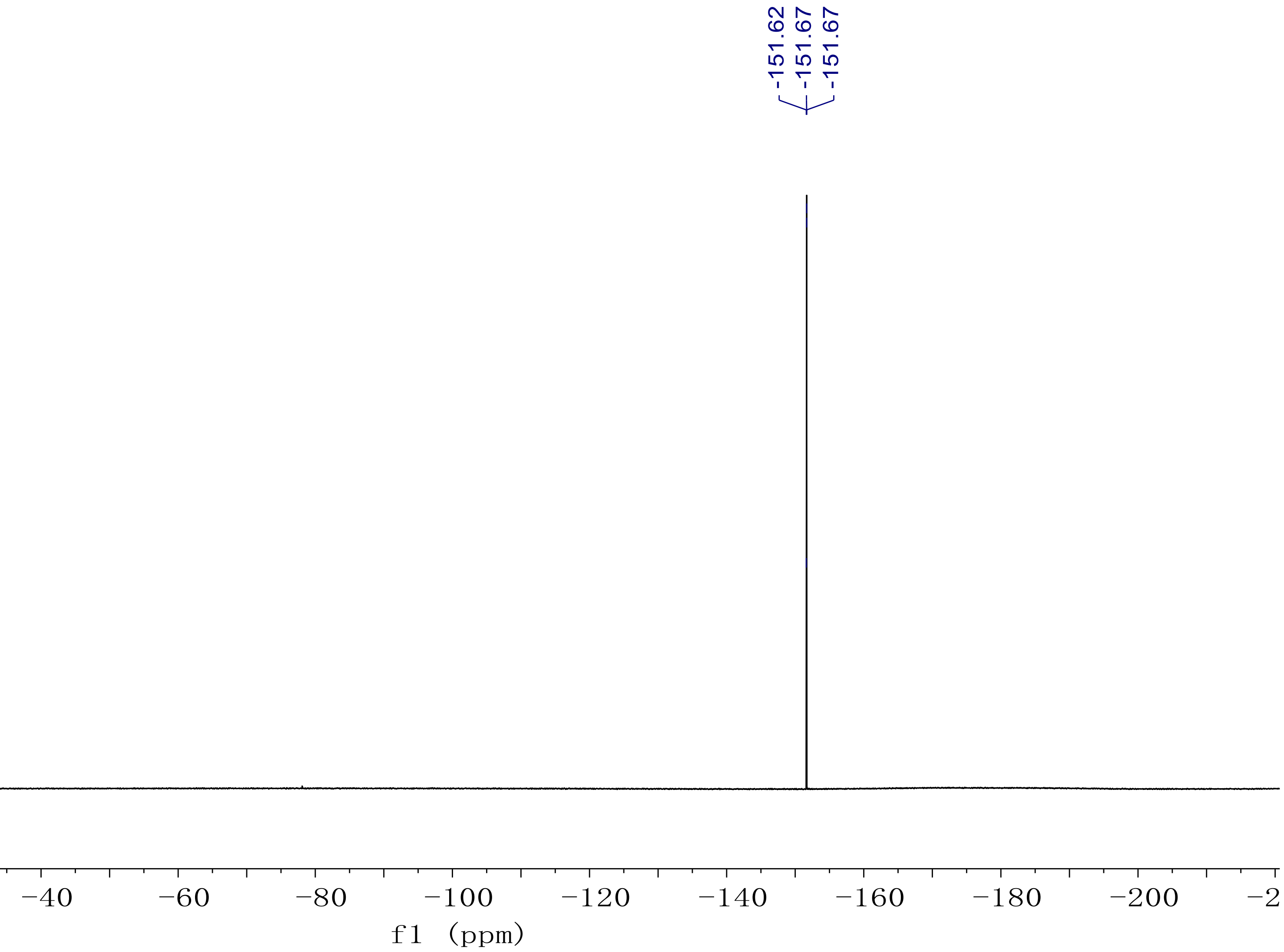

**$^{13}$C NMR of aniline-derived thianthrenium salt 54**

$CDCl_3$, 23 ºC

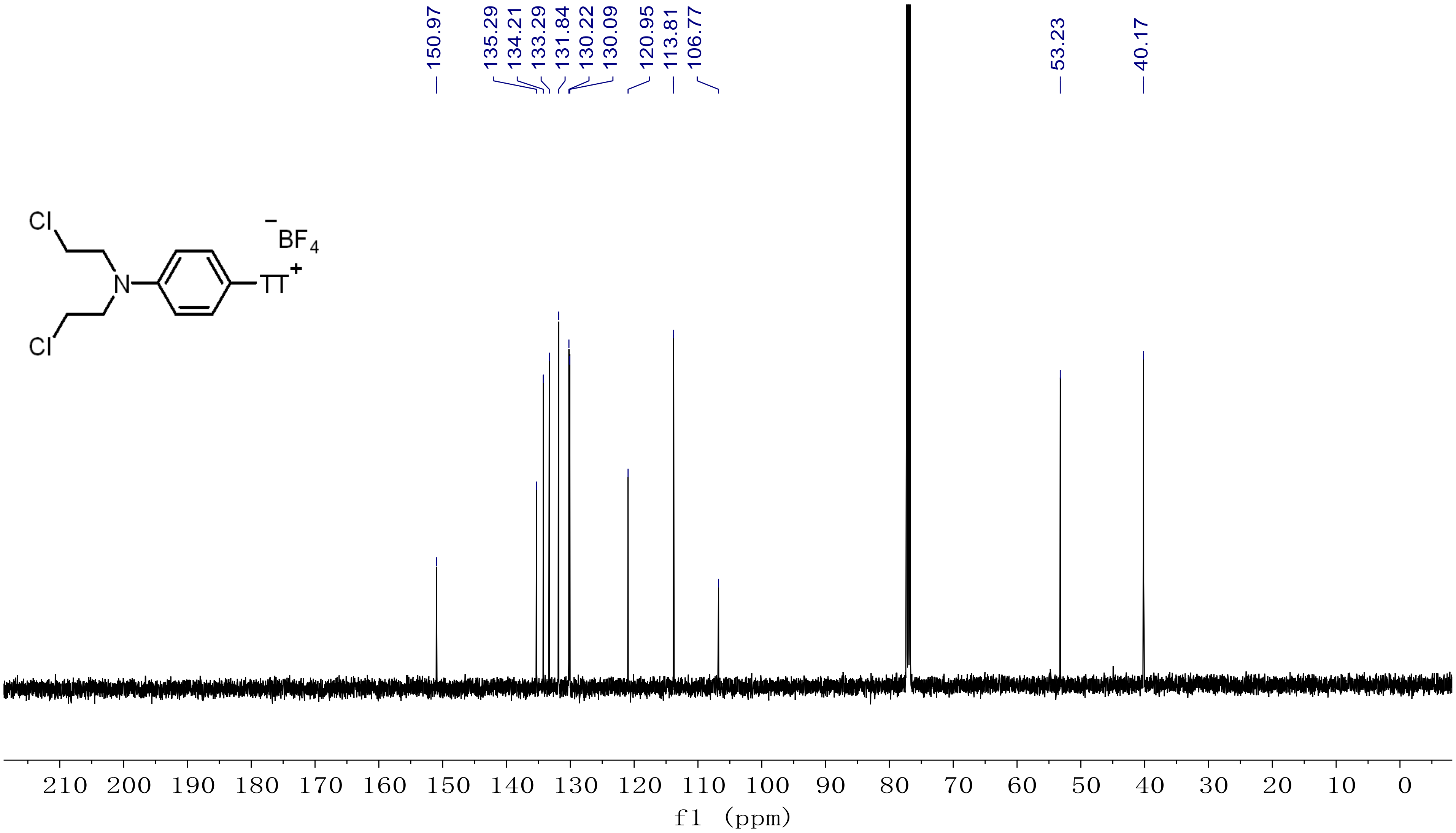

**$^{1}$H NMR of *rac*-melphalan precursor 55**

$CDCl_3$, 23 ºC

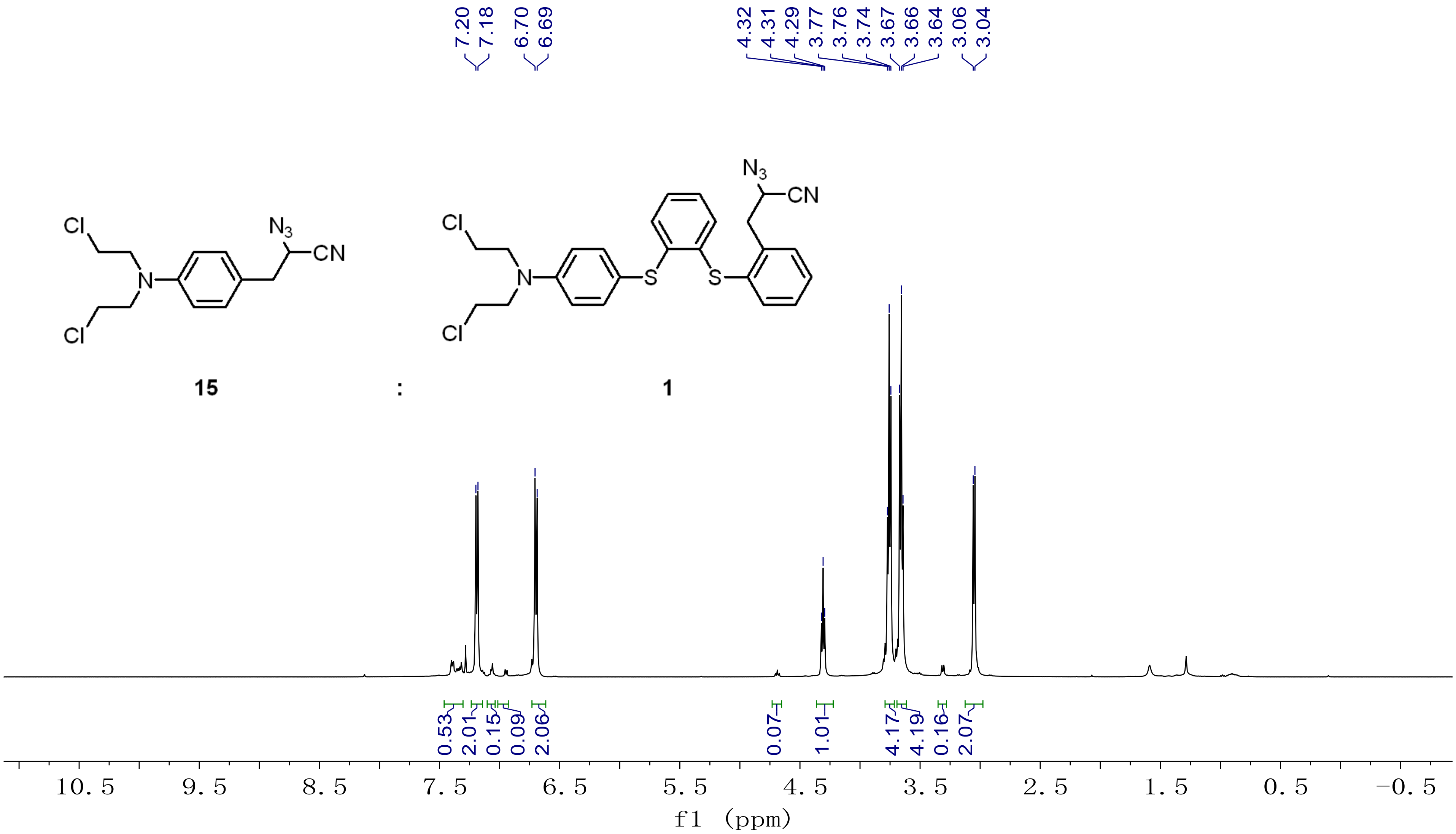

## $^{13}$C NMR of *rac*-melphalan precursor 55

CDCl₃, 23 ºC

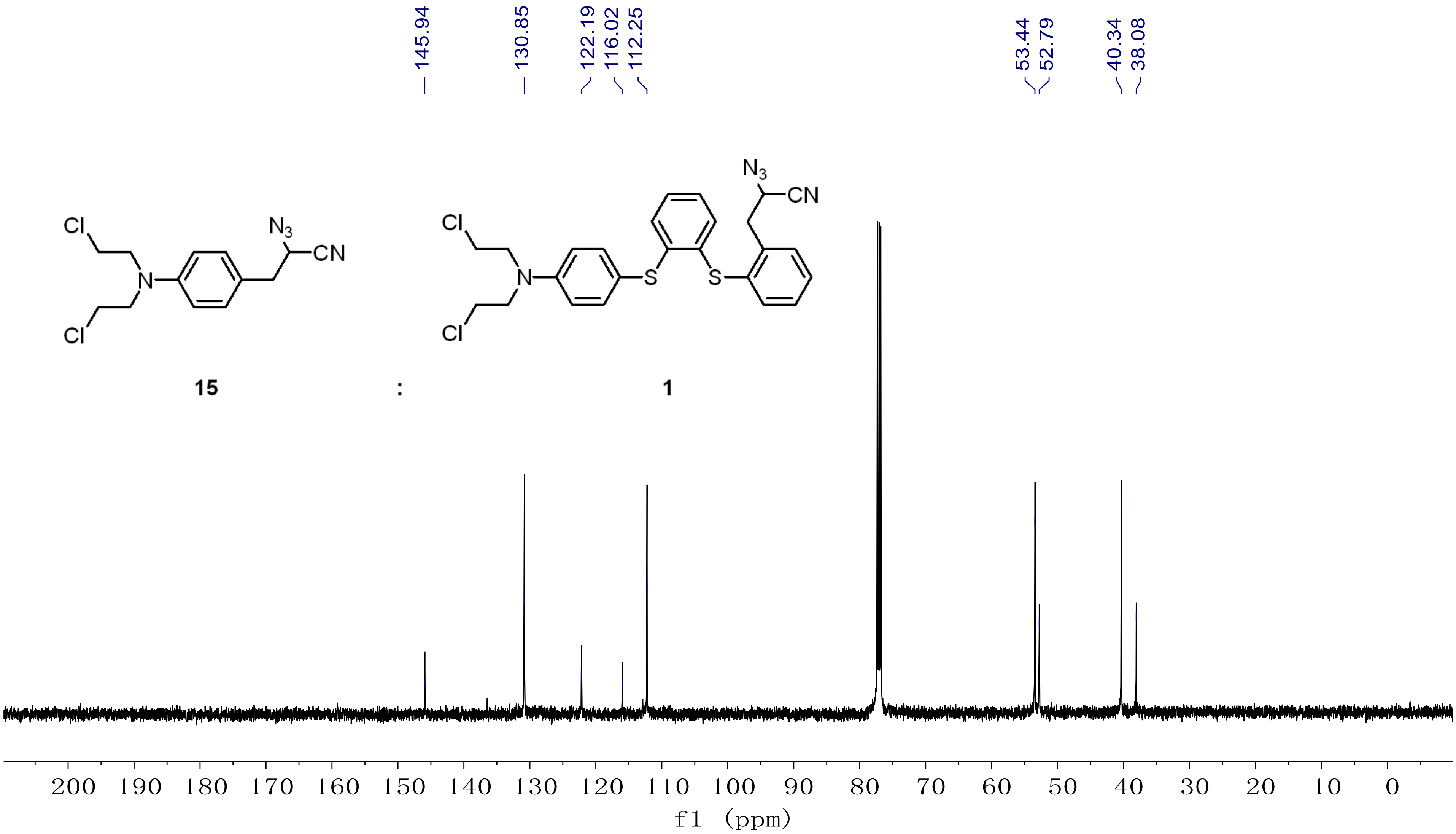

## $^{1}$H NMR of racemic melphalan (56)

$D_2O$, 23 ºC

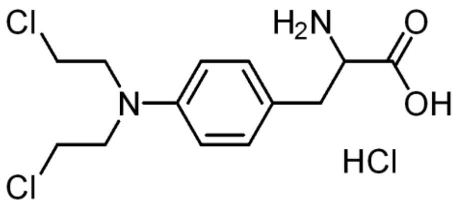


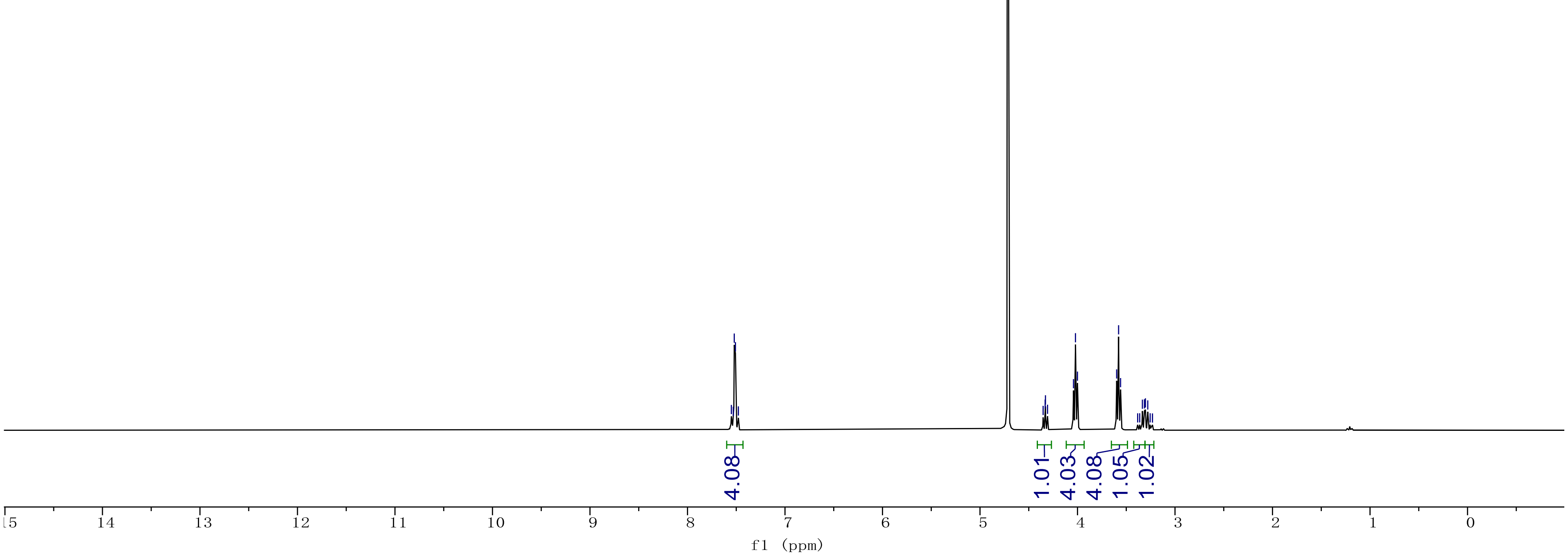

## $^{13}$C NMR of racemic melphalan (56)

$D_2O$, 23 ºC

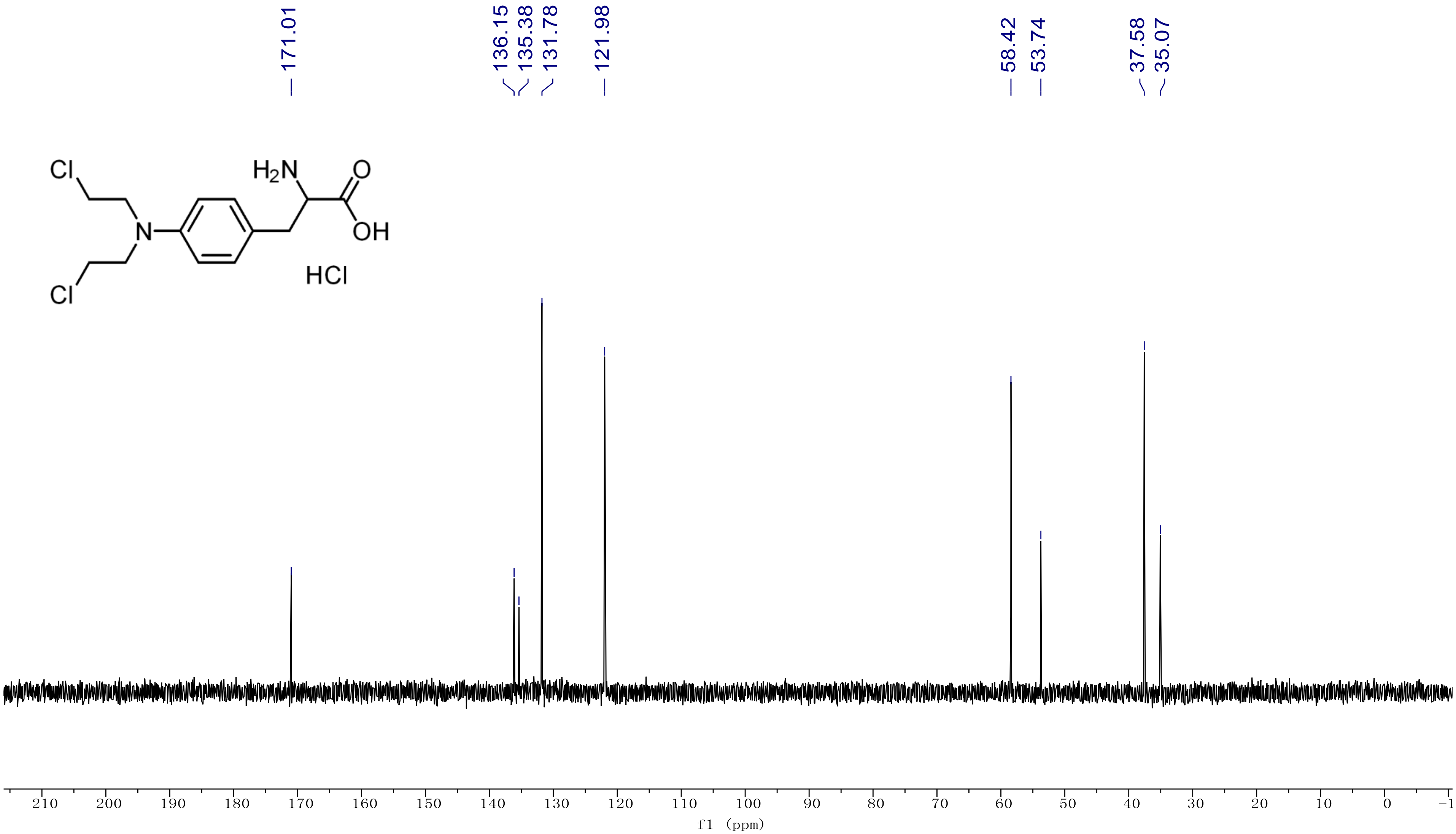

**$^{1}$H NMR of *rac*-pyridinylethylazide S57**

$CDCl_3$, 23 ºC

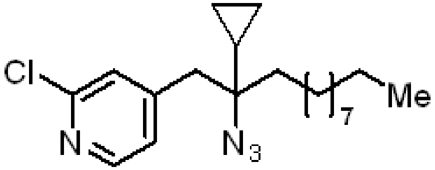

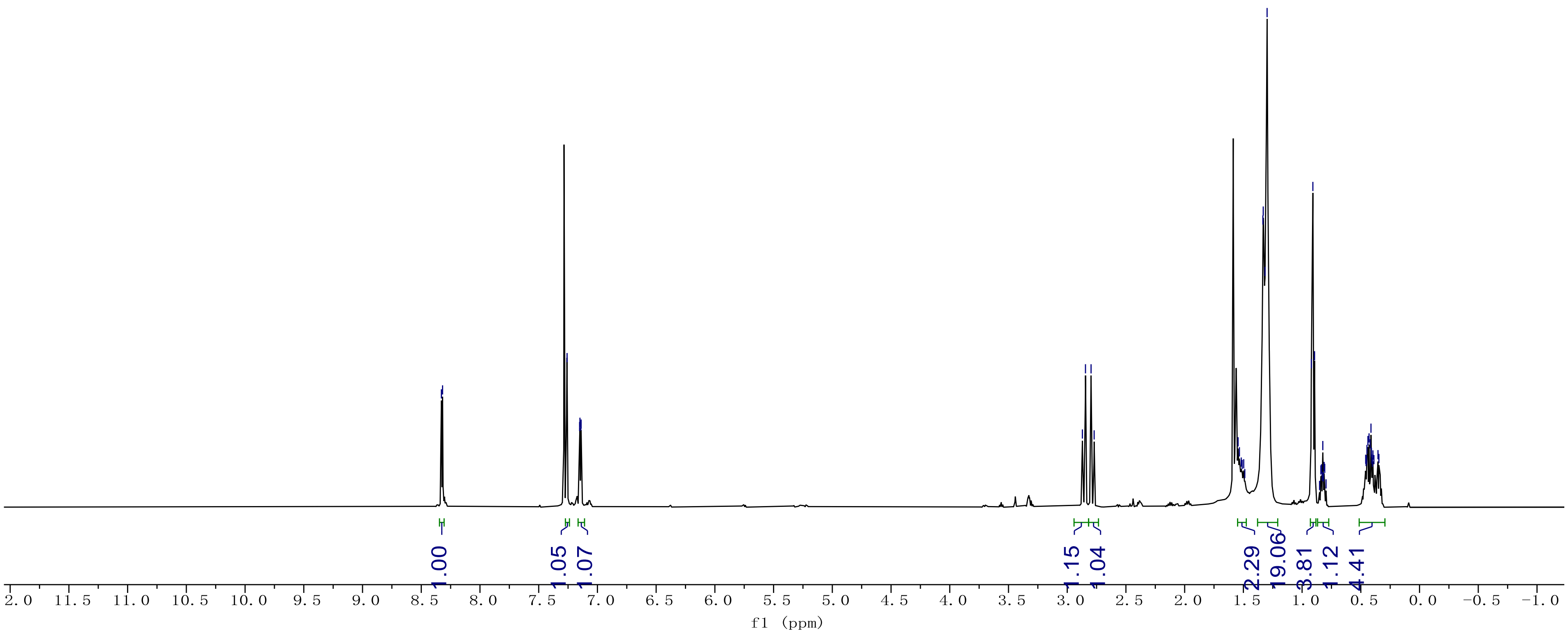

## $^{13}$C NMR of *rac*-pyridinylethylazide S57

CDCl$_3$, 23 ºC

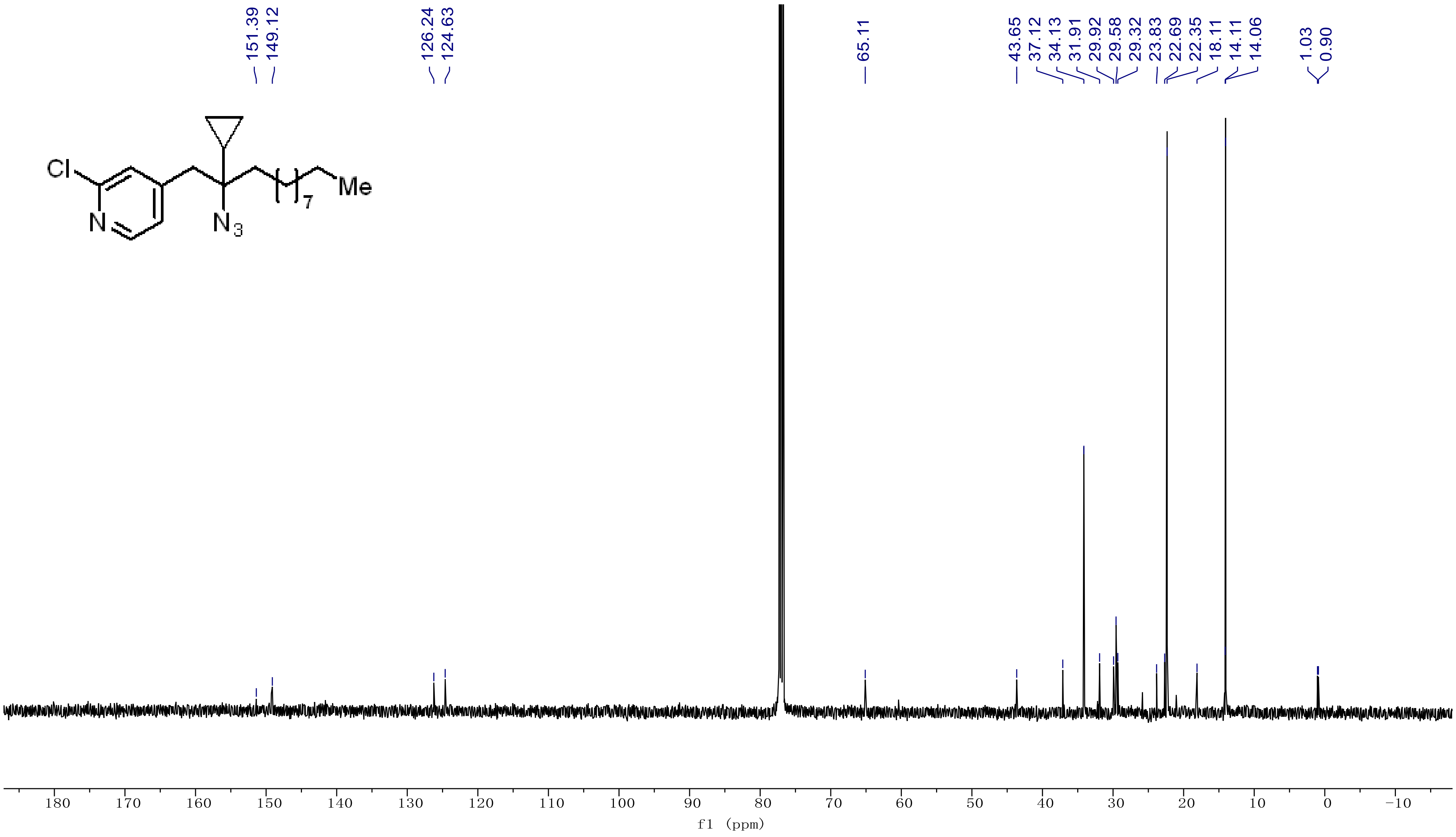

**$^{1}$H NMR of rearrangement product S57’**

$CDCl_3$, 23 ºC

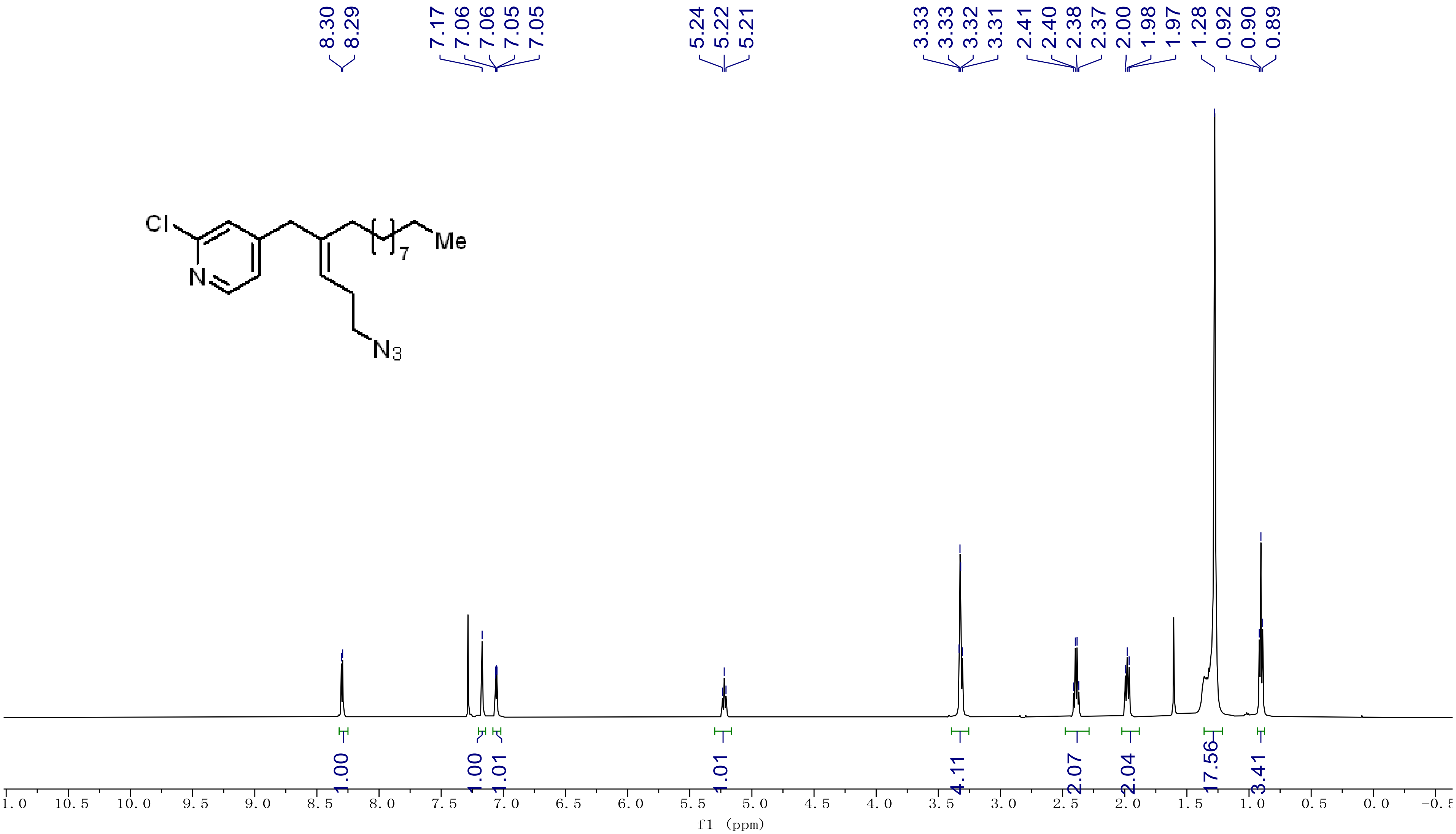

**$^{13}$C NMR of rearrangement product S57’**

$CDCl_3$, 23 ºC

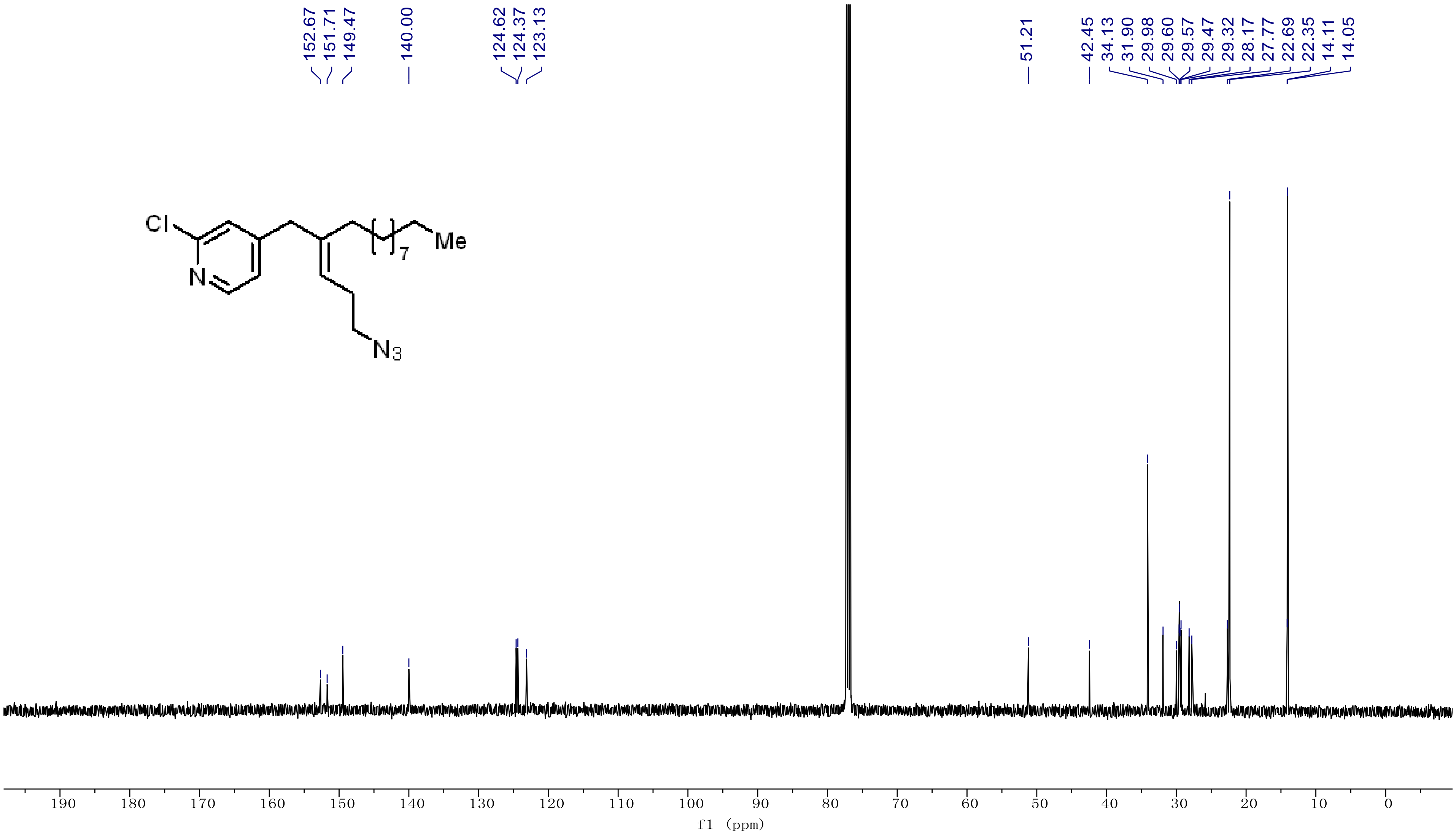

**$^{1}$H NMR of nimesulide-derived thianthrenium salt TT-5**

CDCl3, 23 ºC

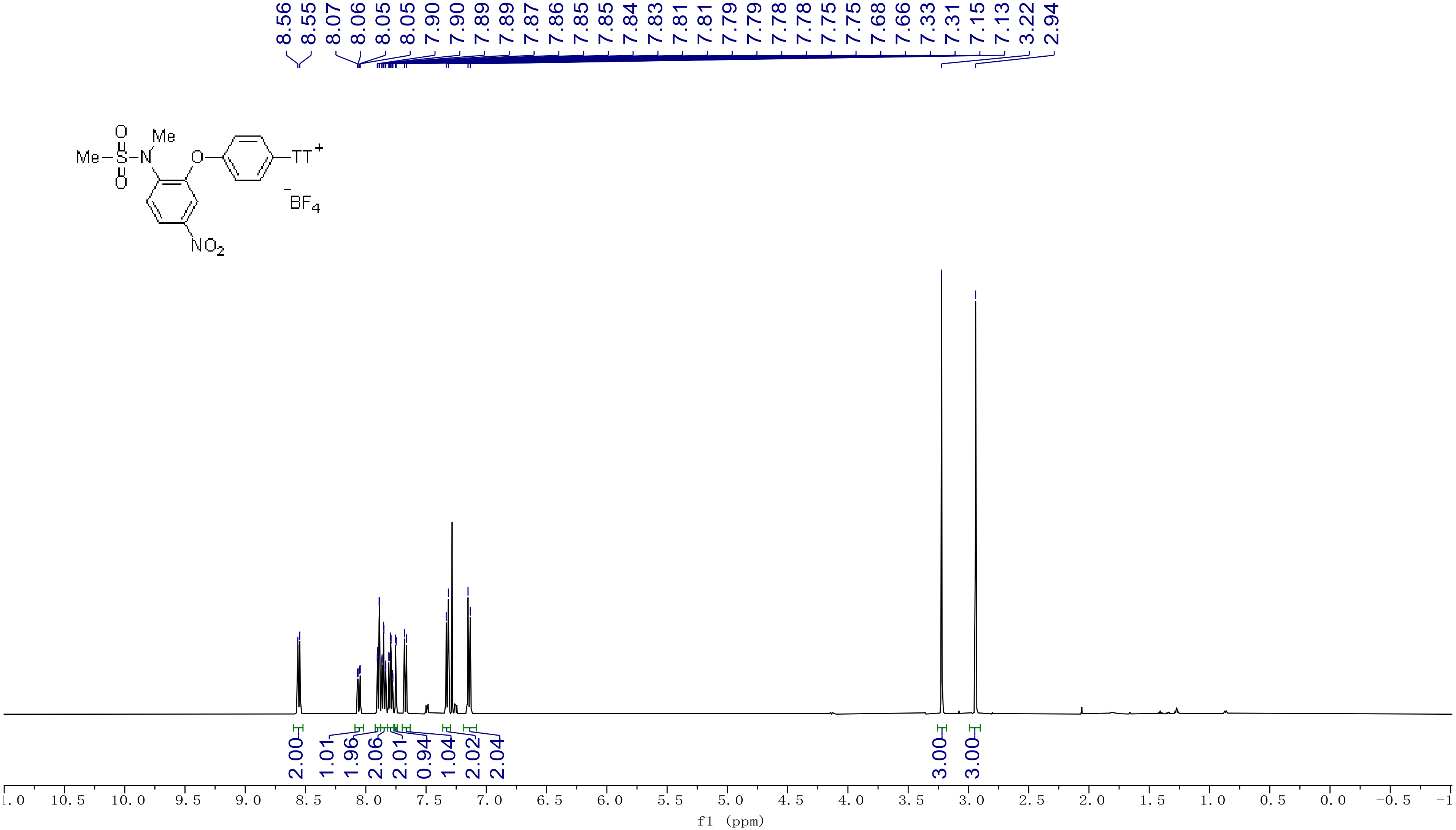

## $^{19}F$ NMR of nimesulide-derived thianthrenium salt TT-5

$CDCl_3$, 23 ºC

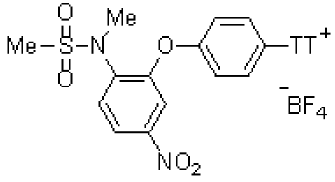


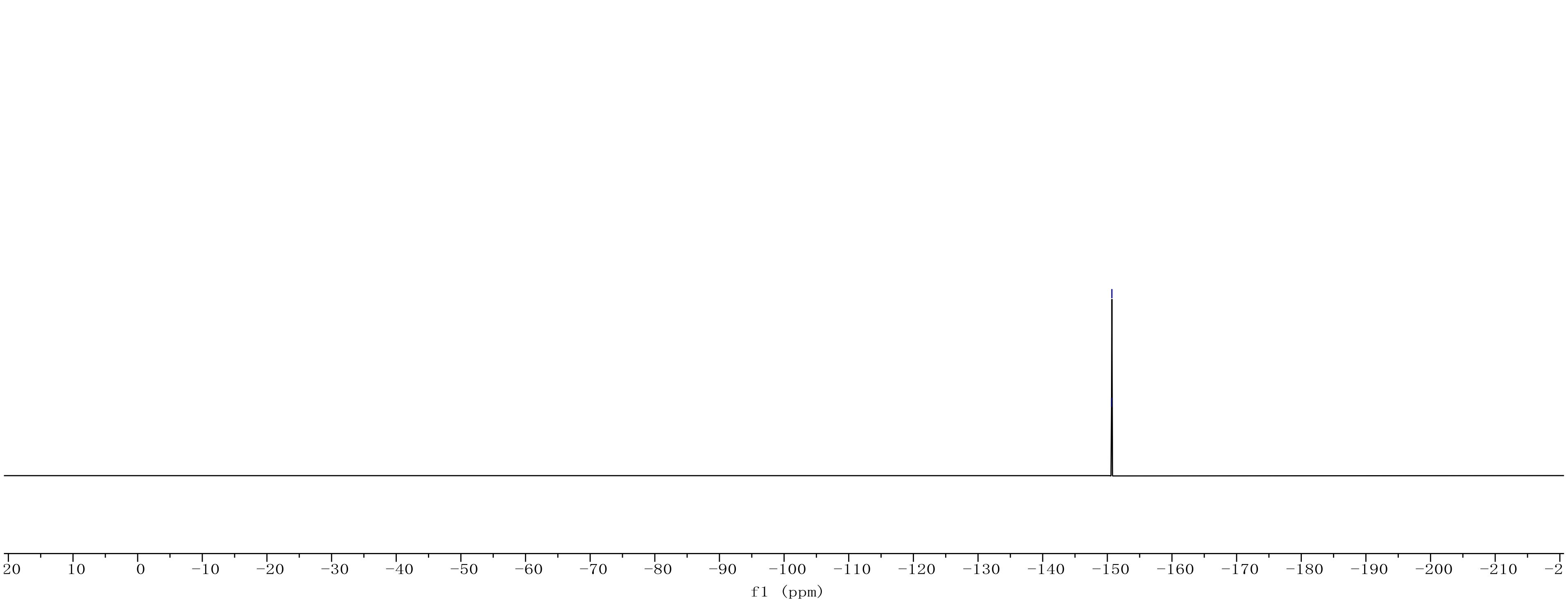

## $^{13}C$ NMR of nimesulide-derived thianthrenium salt TT-5

$CDCl_3$, 23 ºC

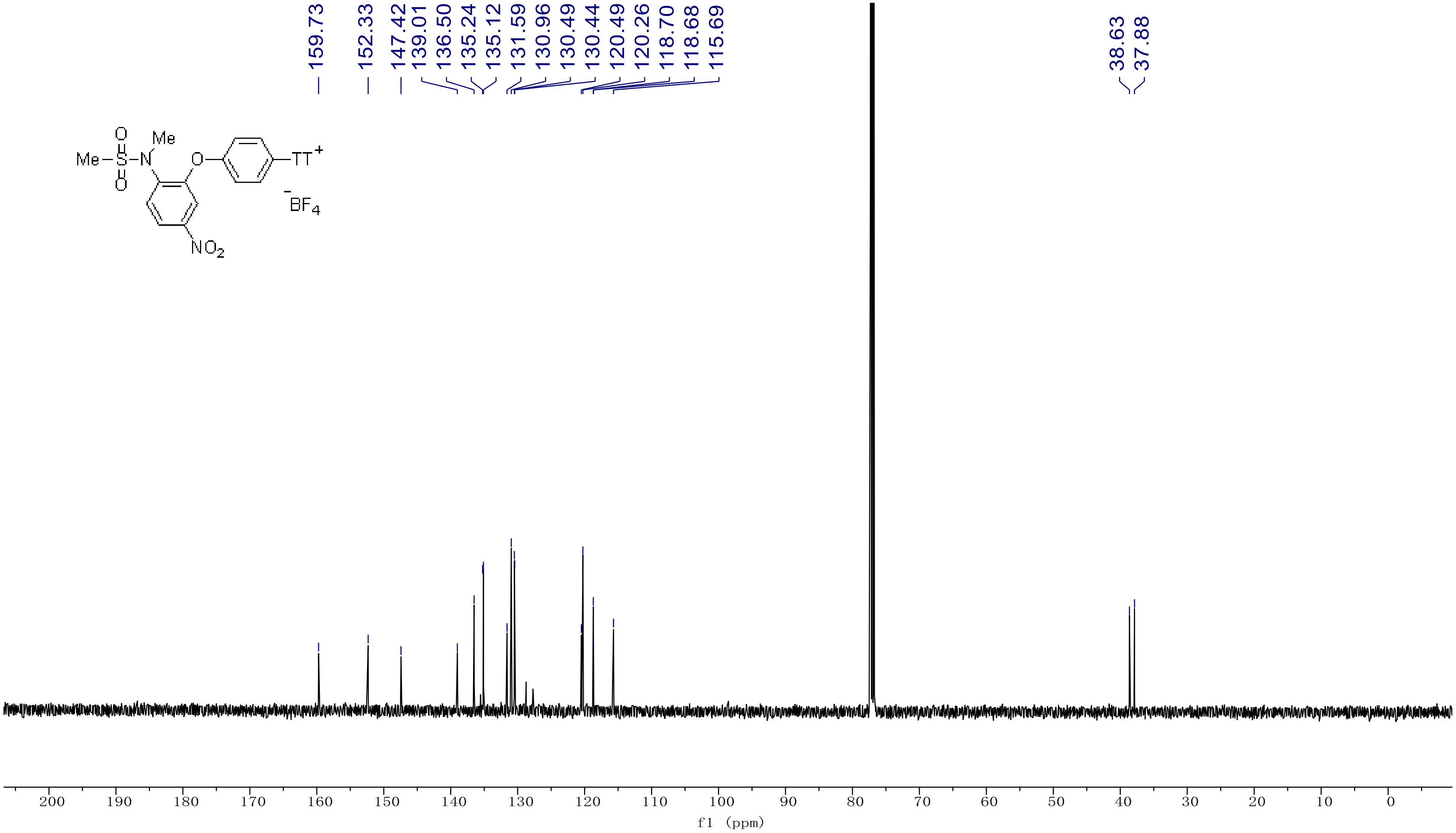

**$^{1}$H NMR of meclofenamic acid-derived thianthrenium salt TT-6**

$CDCl_3$, 23 ºC

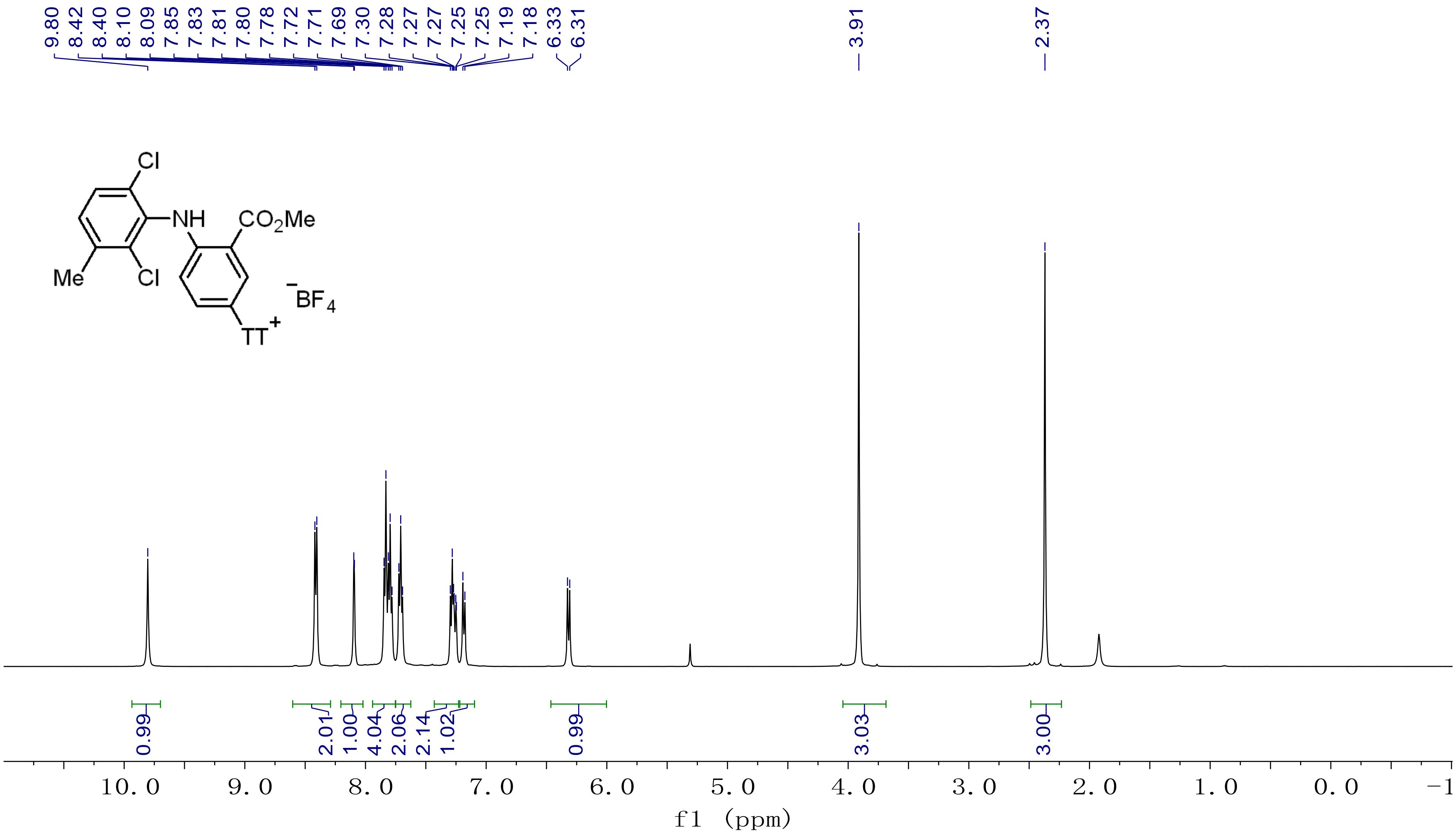

**$^{19}$F NMR of meclofenamic acid-derived thianthrenium salt TT-6**

$CDCl_3$, 23 ºC

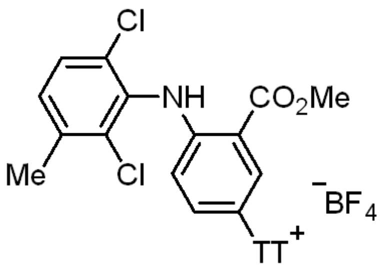


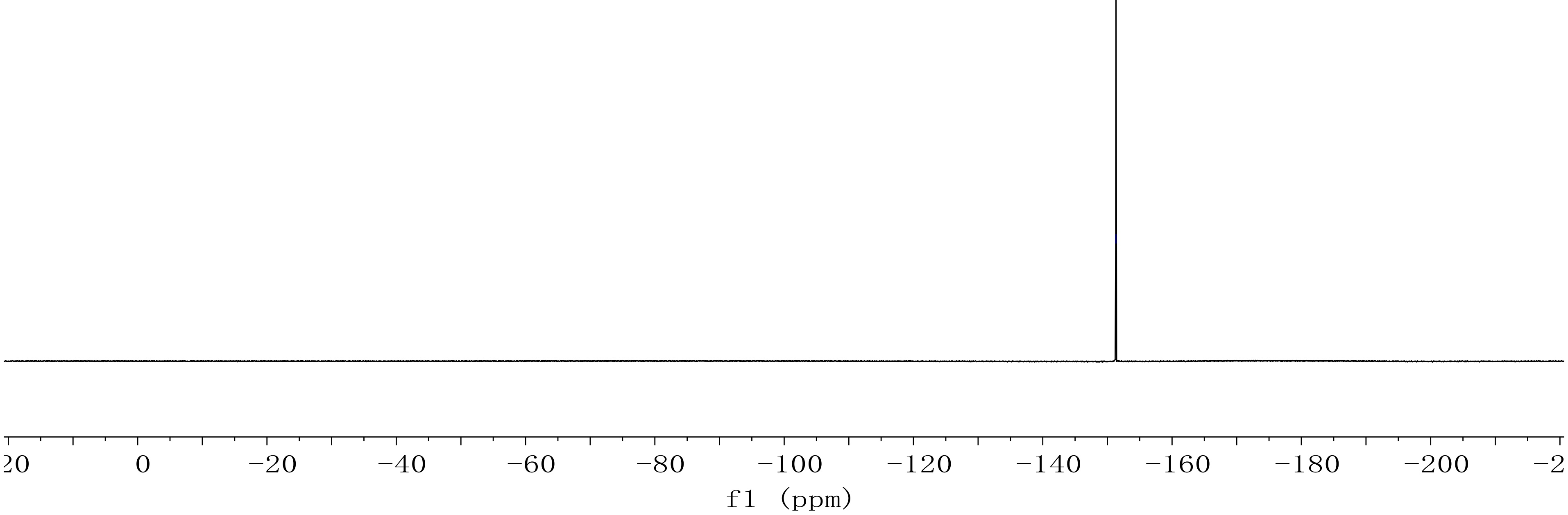

## $^{13}$C NMR of meclofenamic acid-derived thianthrenium salt TT-6

$CDCl_3$, 23 ºC

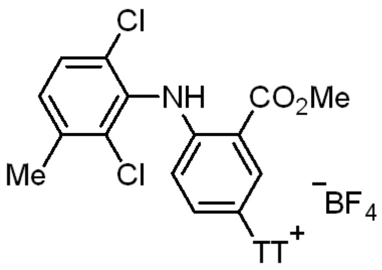


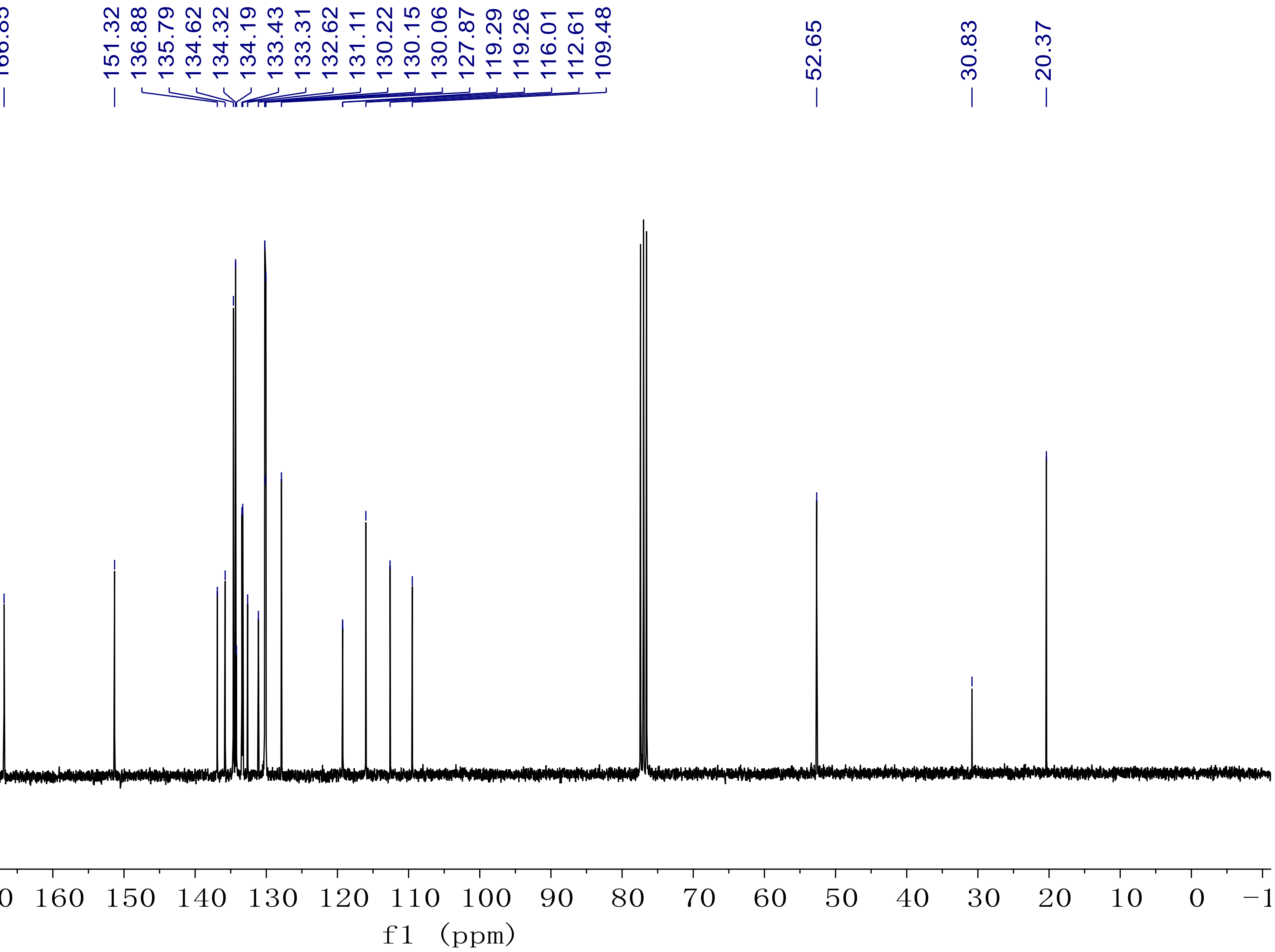

**$^{1}$H NMR of benzbromarone-derived thianthrenium salt TT-7**

$CDCl_3$, 23 ºC

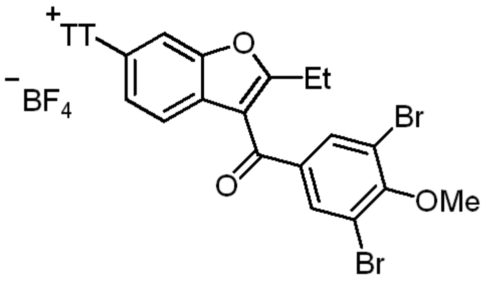


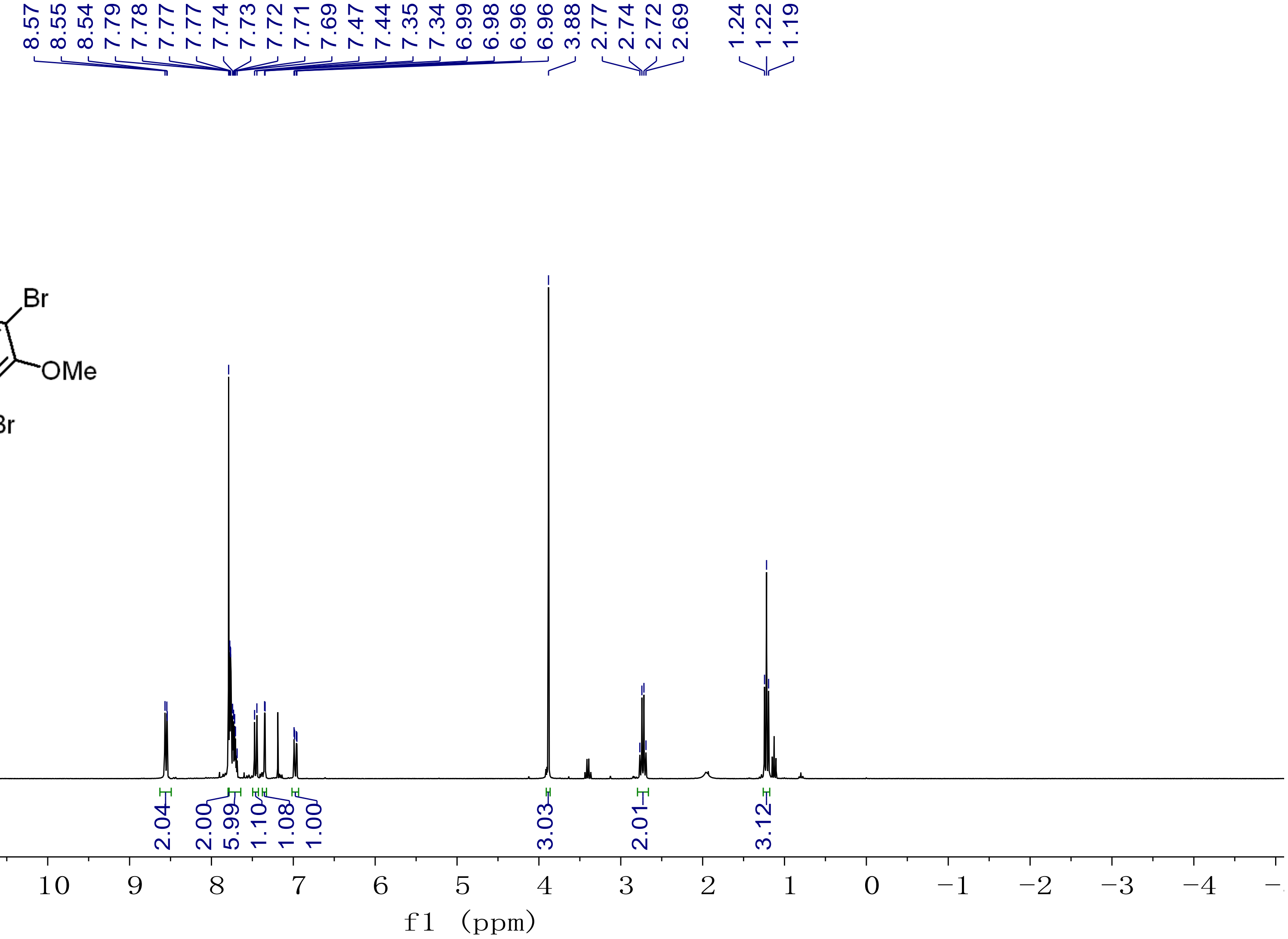

**$^{19}$F NMR of benzbromarone-derived thianthrenium salt TT-7**

$CDCl_3$, 23 ºC

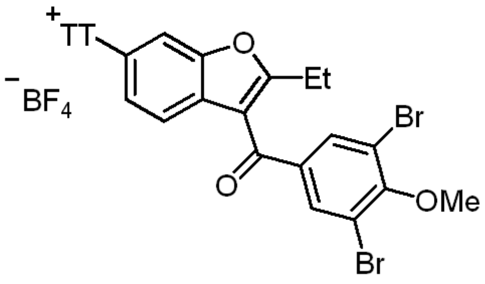


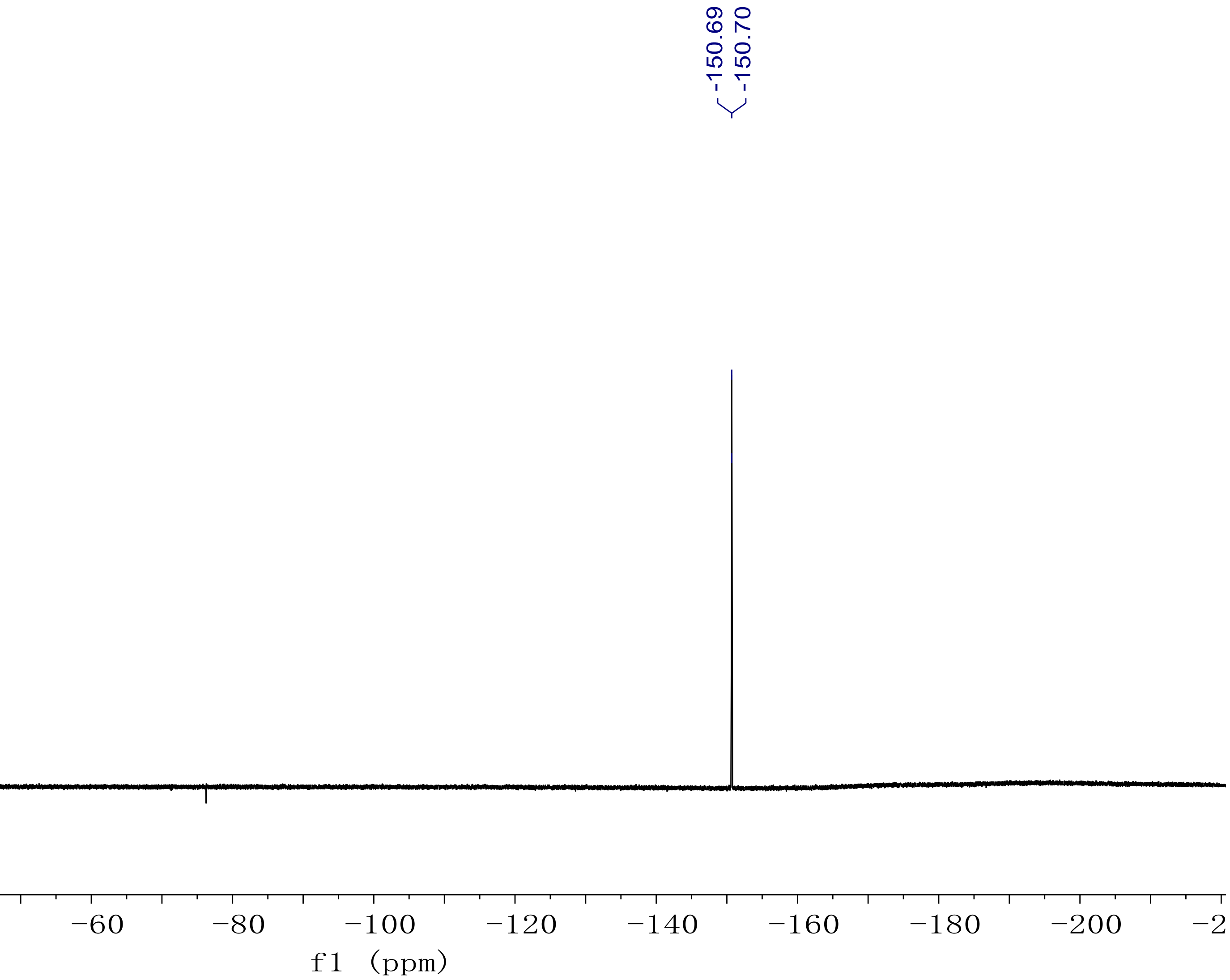

**$^{13}$C NMR of benzbromarone-derived thianthrenium salt TT-7**

$CDCl_3$, 23 ºC

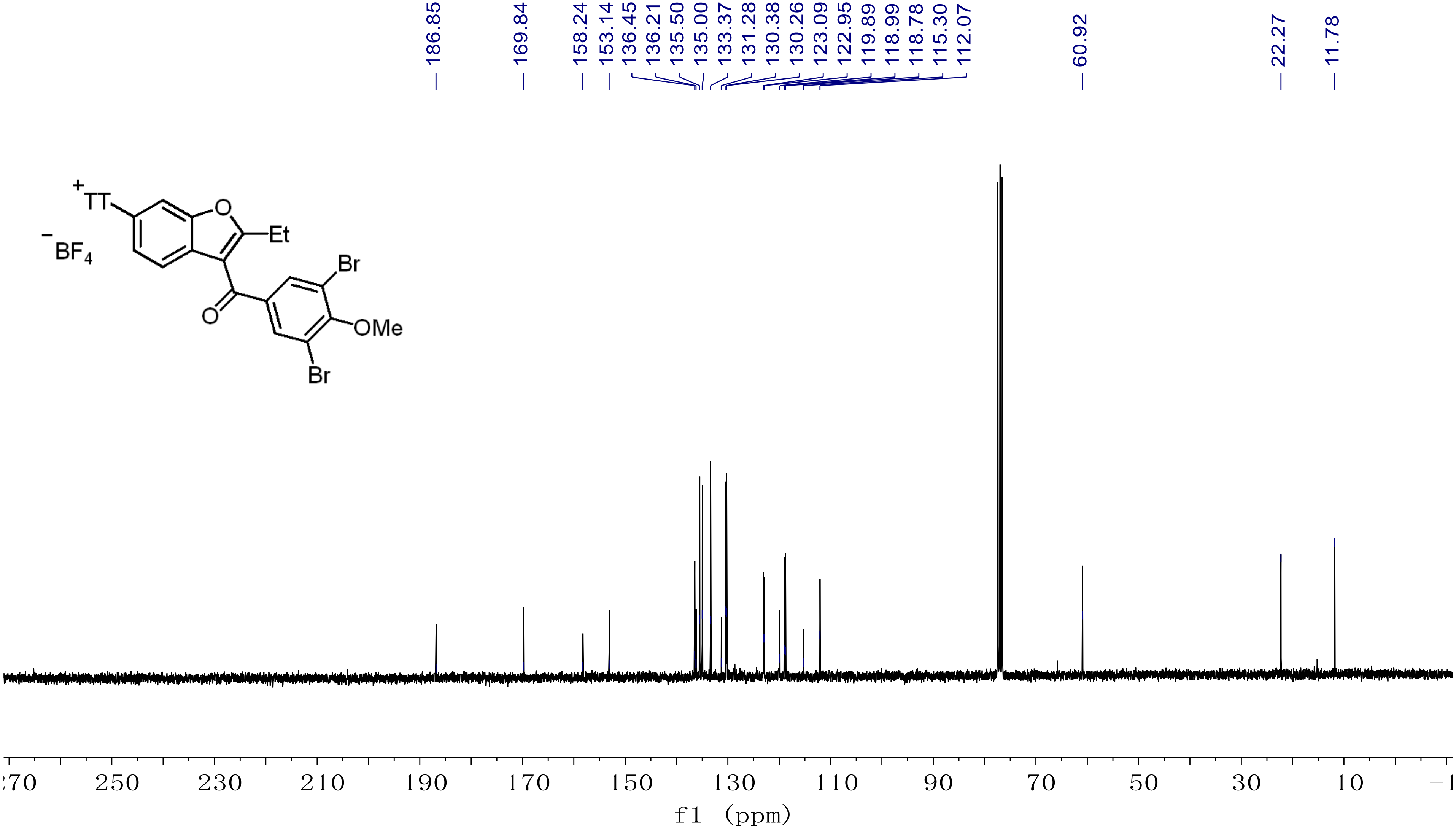

## $^1$H NMR of estrone-derived thianthrenium salt TT-10

$CDCl_3$, 23 ºC

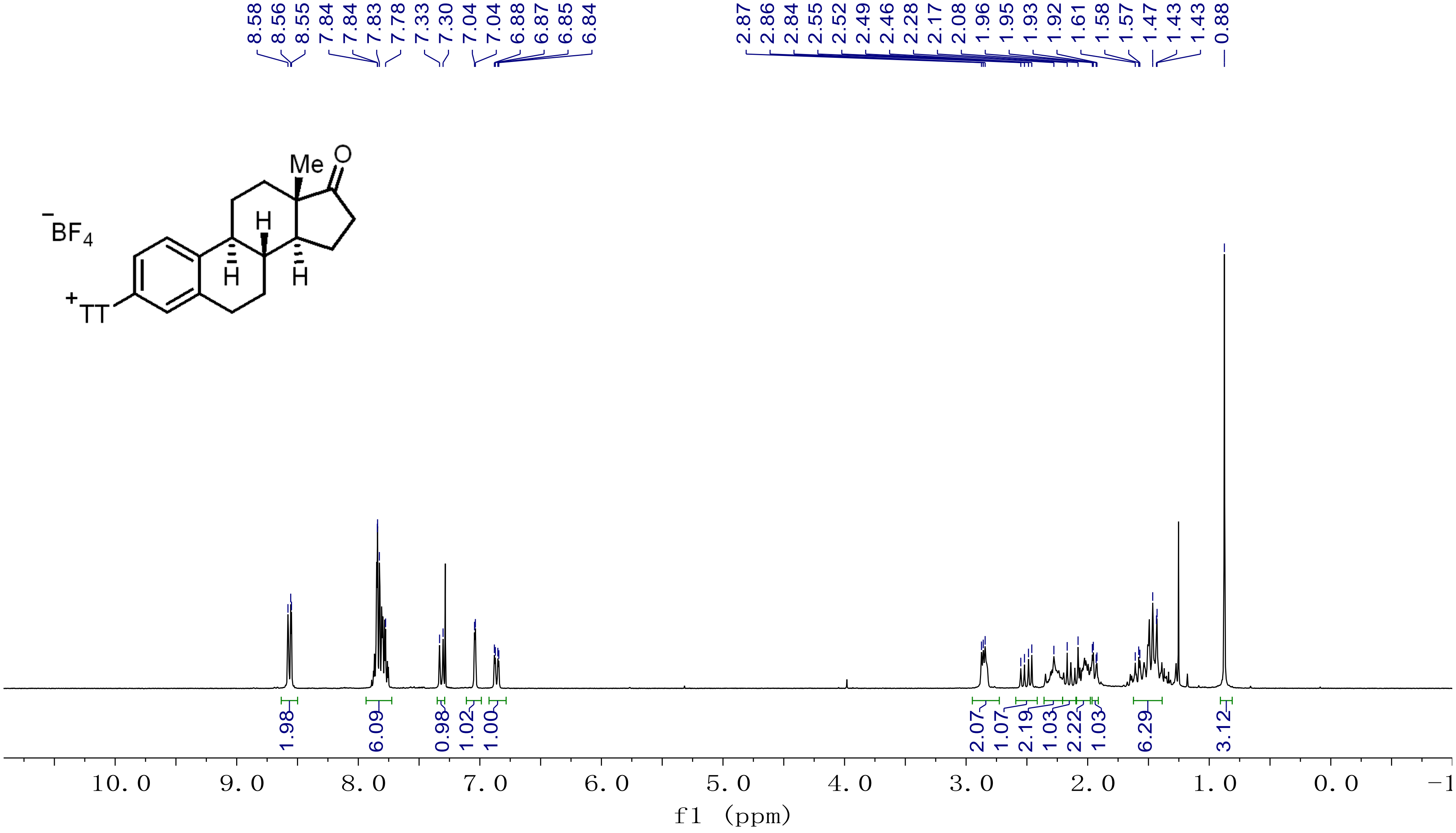

**$^{19}$F NMR of estrone-derived thianthrenium salt TT-10**

$CDCl_3$, 23 ºC

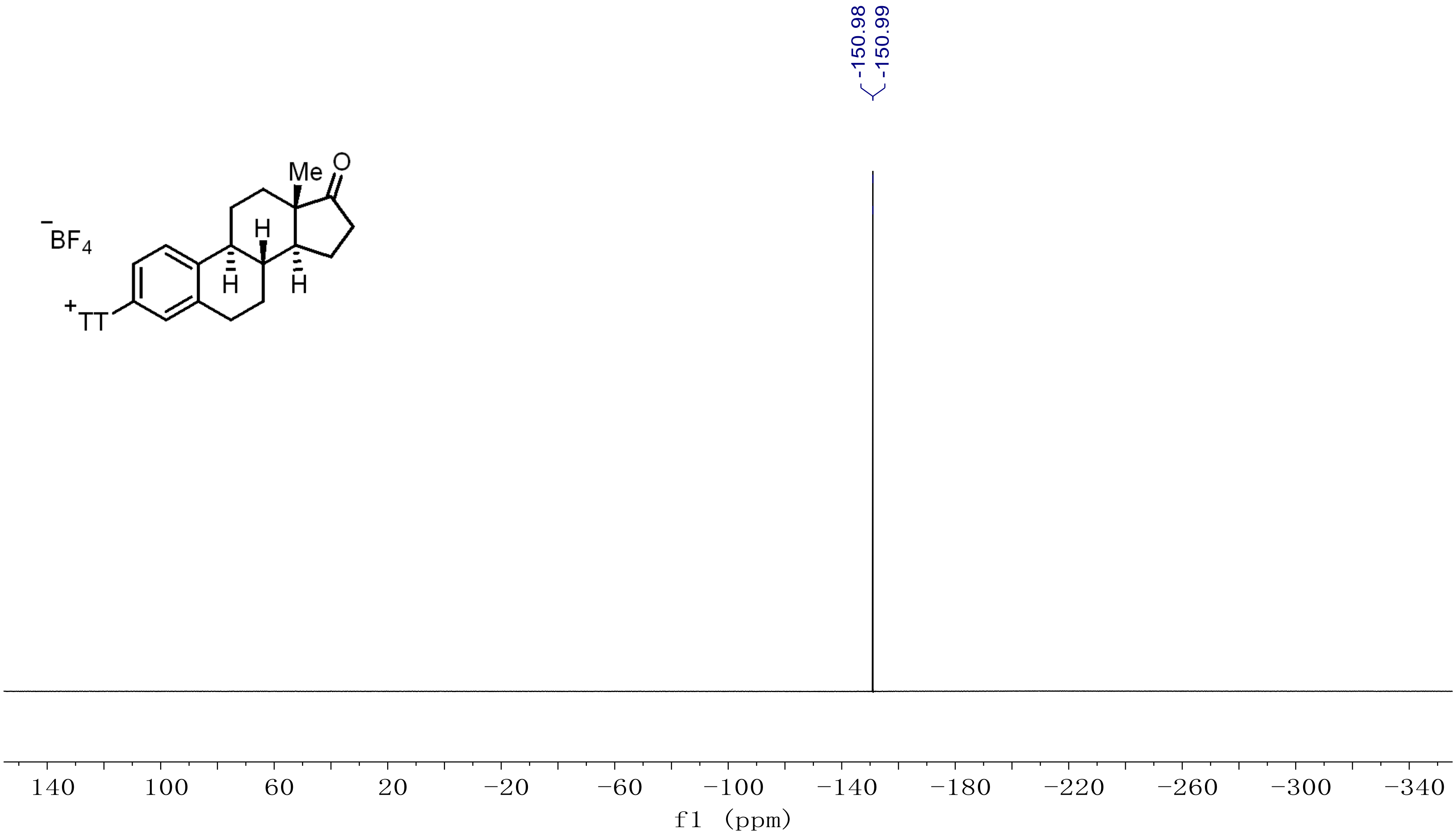

# $^{13}$C NMR of estrone-derived thianthrenium salt TT-10

$CDCl_3$, 23 ºC

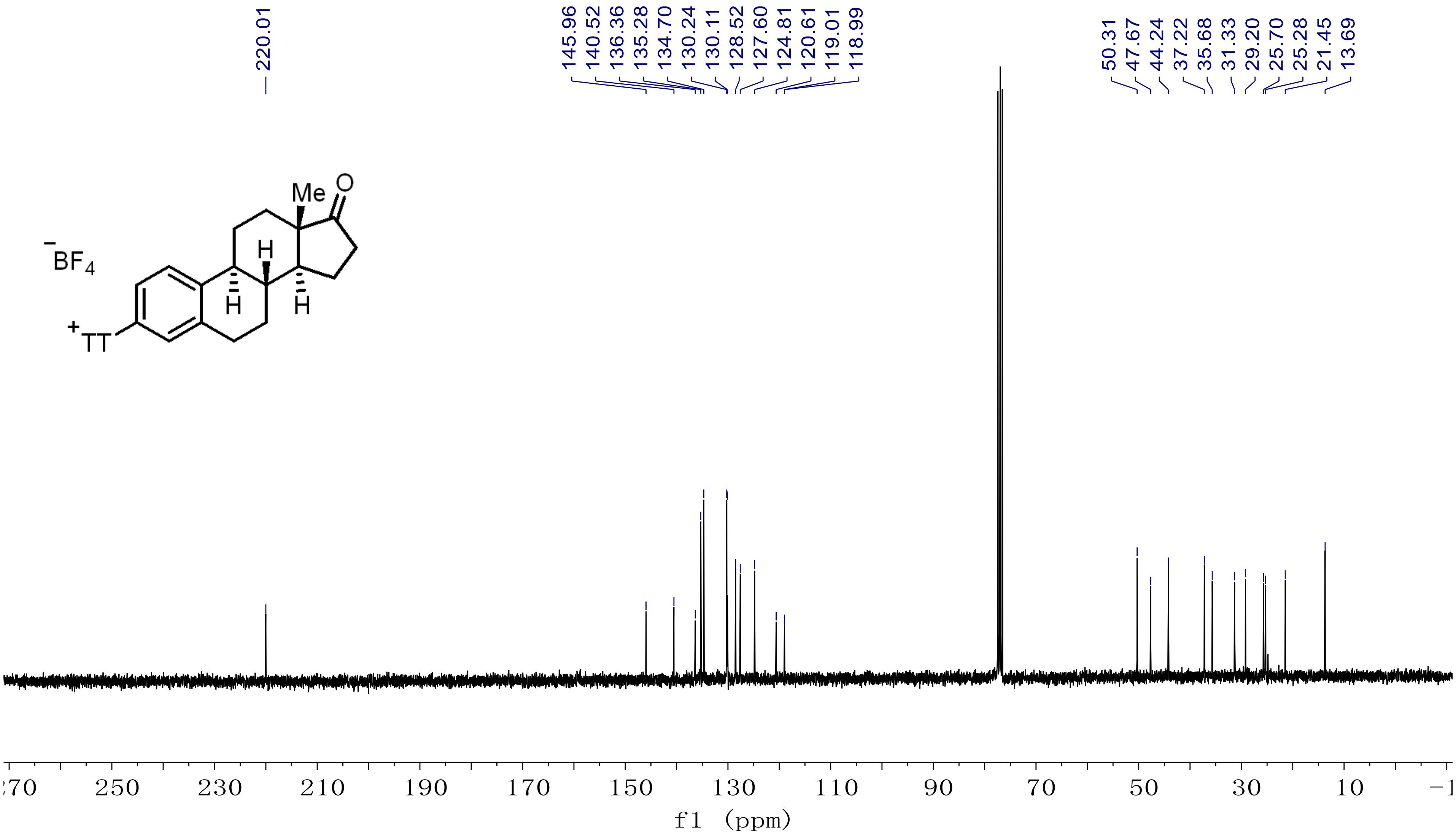

**$^1$H NMR of tianeptine intermediate-derived thianthrenium salt TT-11**

$CDCl_3$, 23 ºC

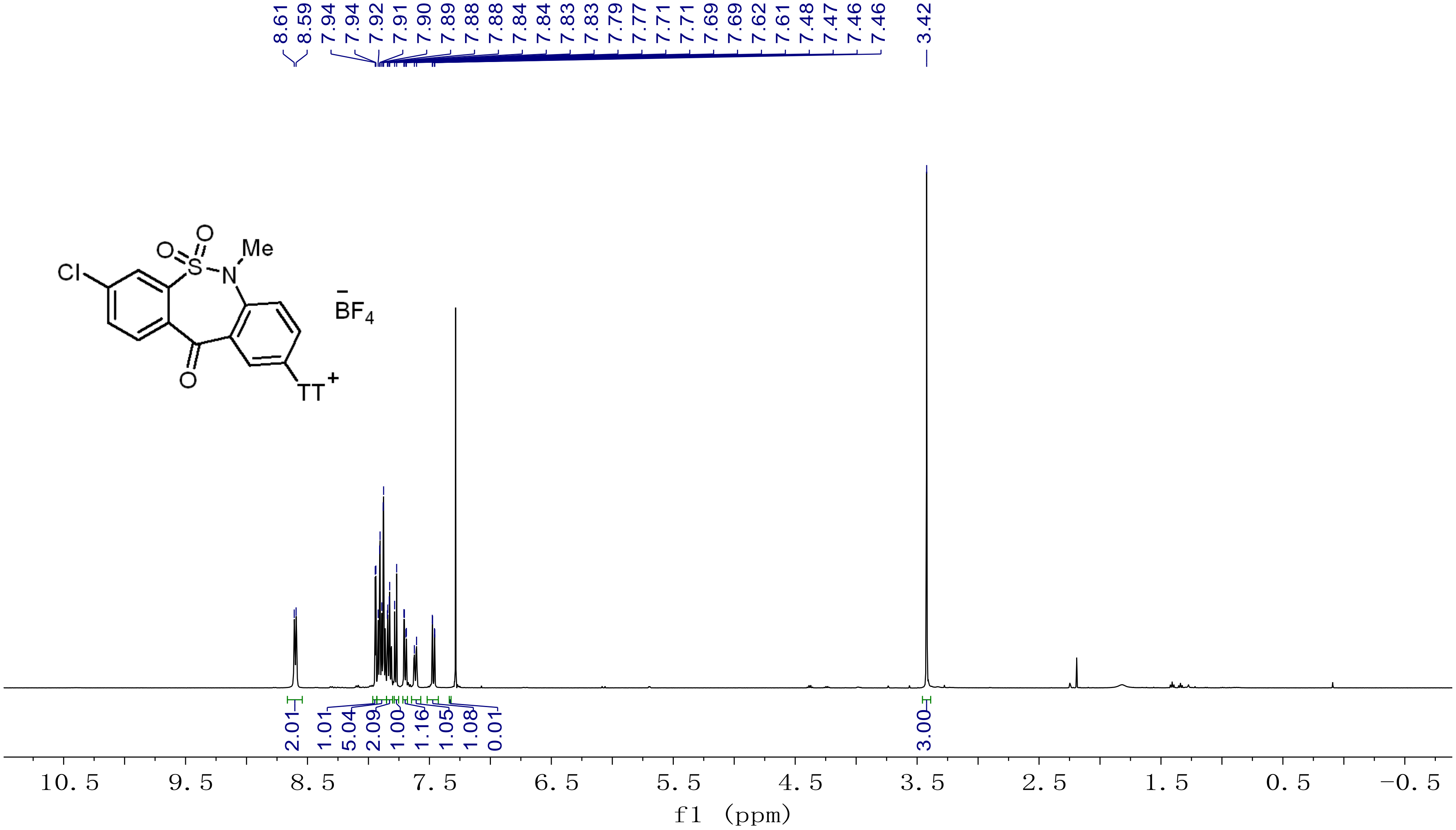

**$^{19}$F NMR of tianeptine intermediate-derived thianthrenium salt TT-11**

$CDCl_3$, 23 ºC

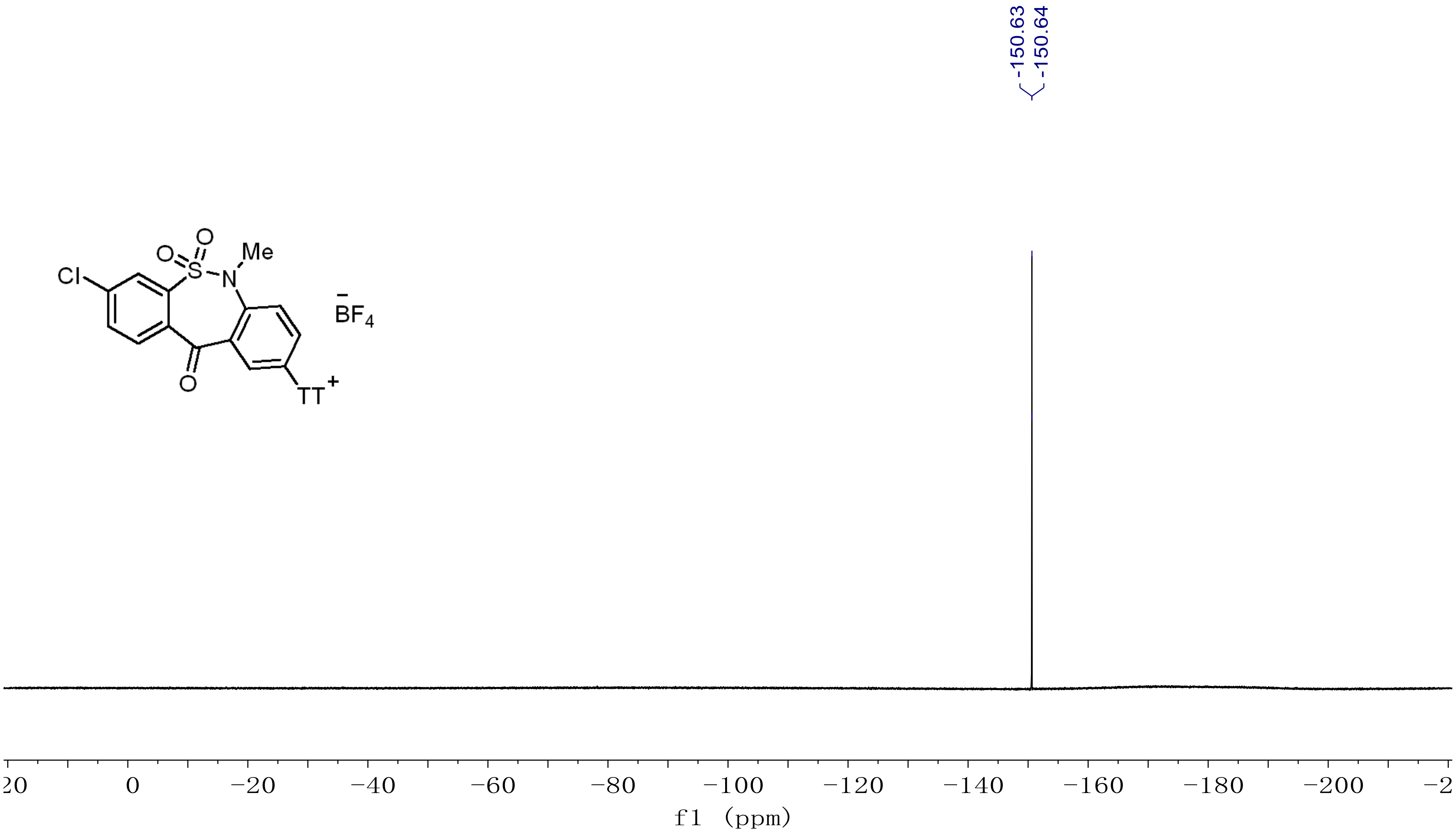

# $^{13}$C NMR of tianeptine intermediate-derived thianthrenium salt TT-11

CDCl$_3$, 23 ºC

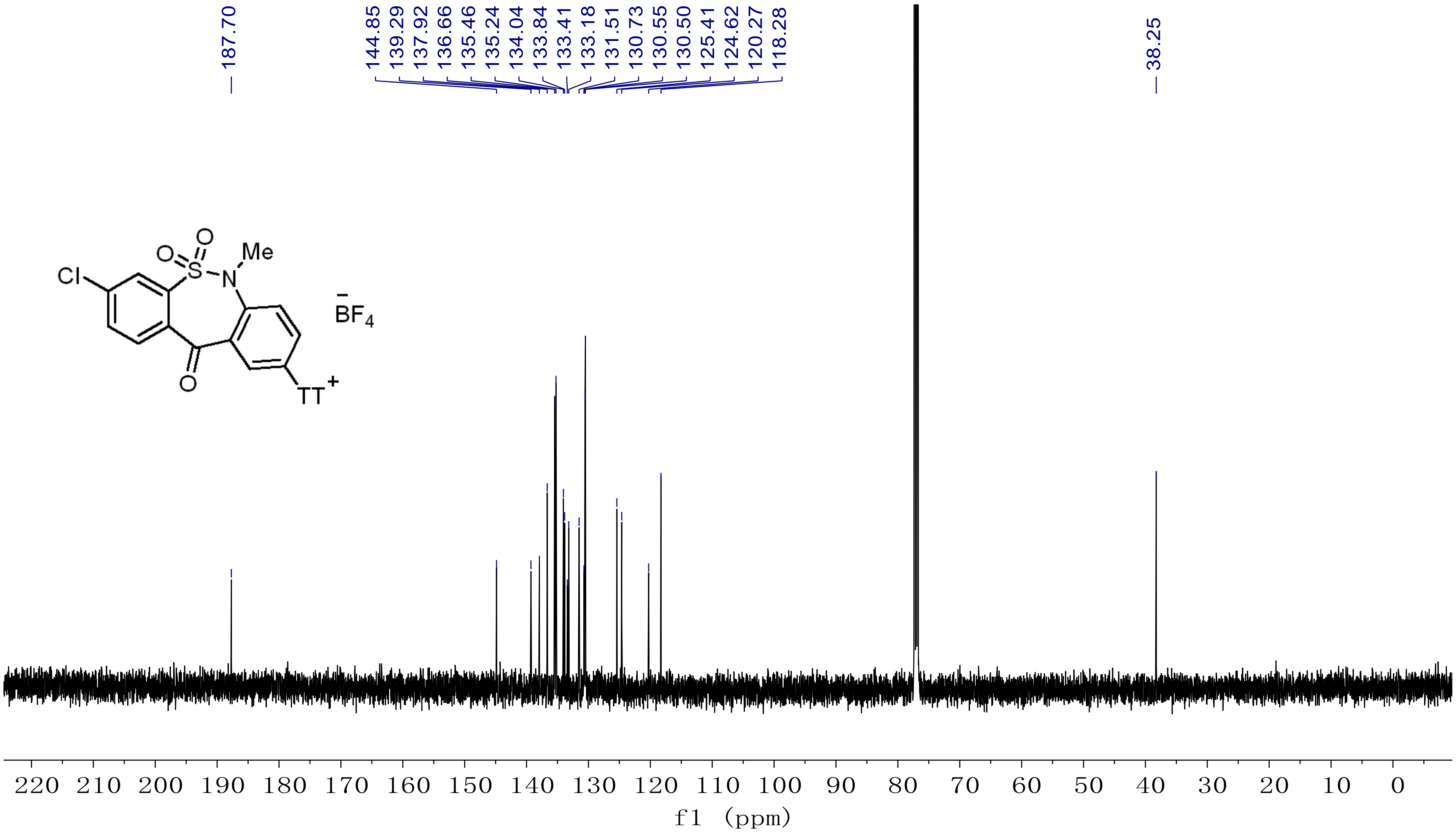

## $^{1}$H NMR of quinoline-derived thianthrenium salt TT-14

$CDCl_3$, 23 ºC

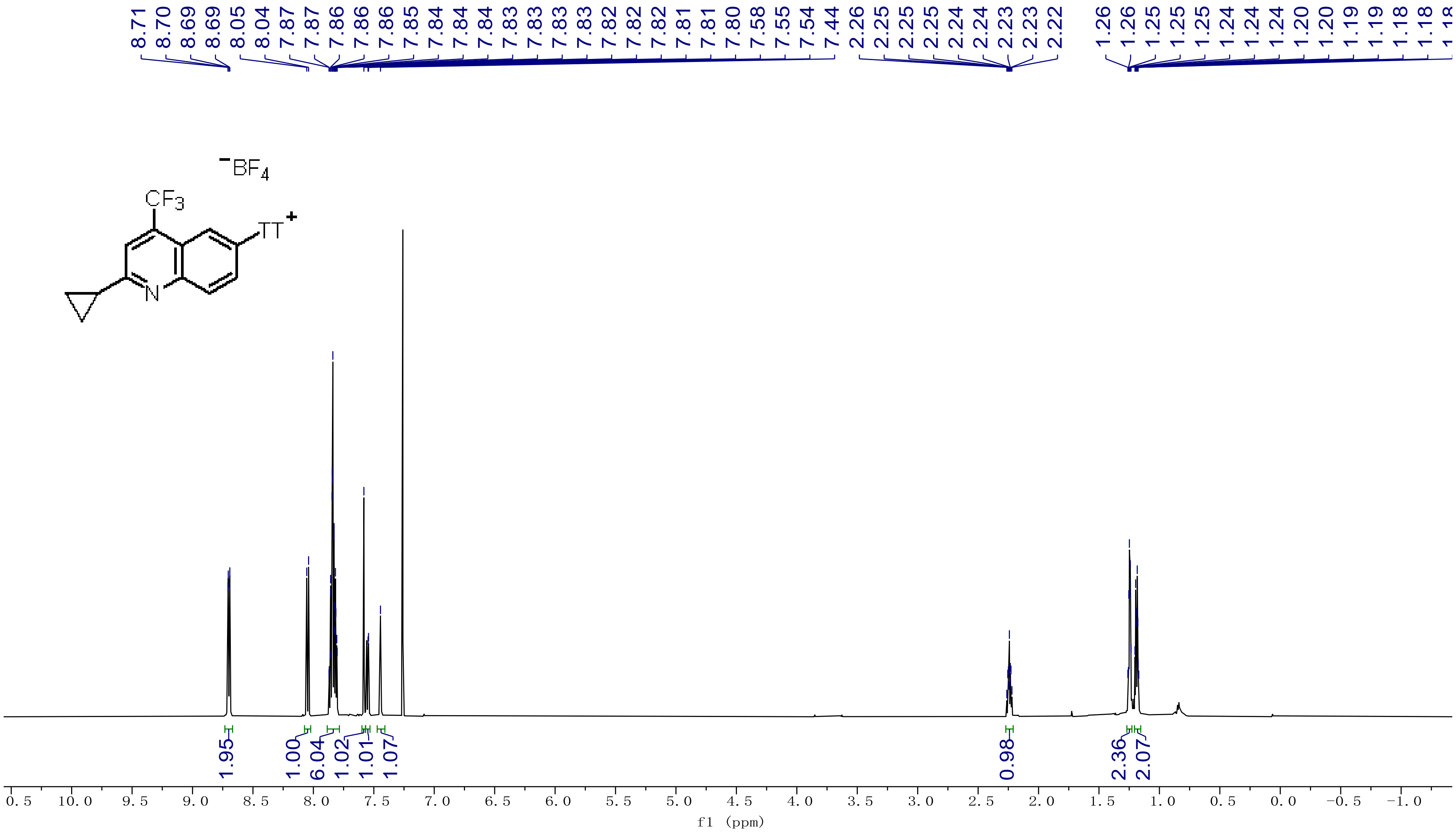

## $^{19}F$ NMR of quinoline -derived thianthrenium salt TT-14

$CDCl_3$, 23 ºC

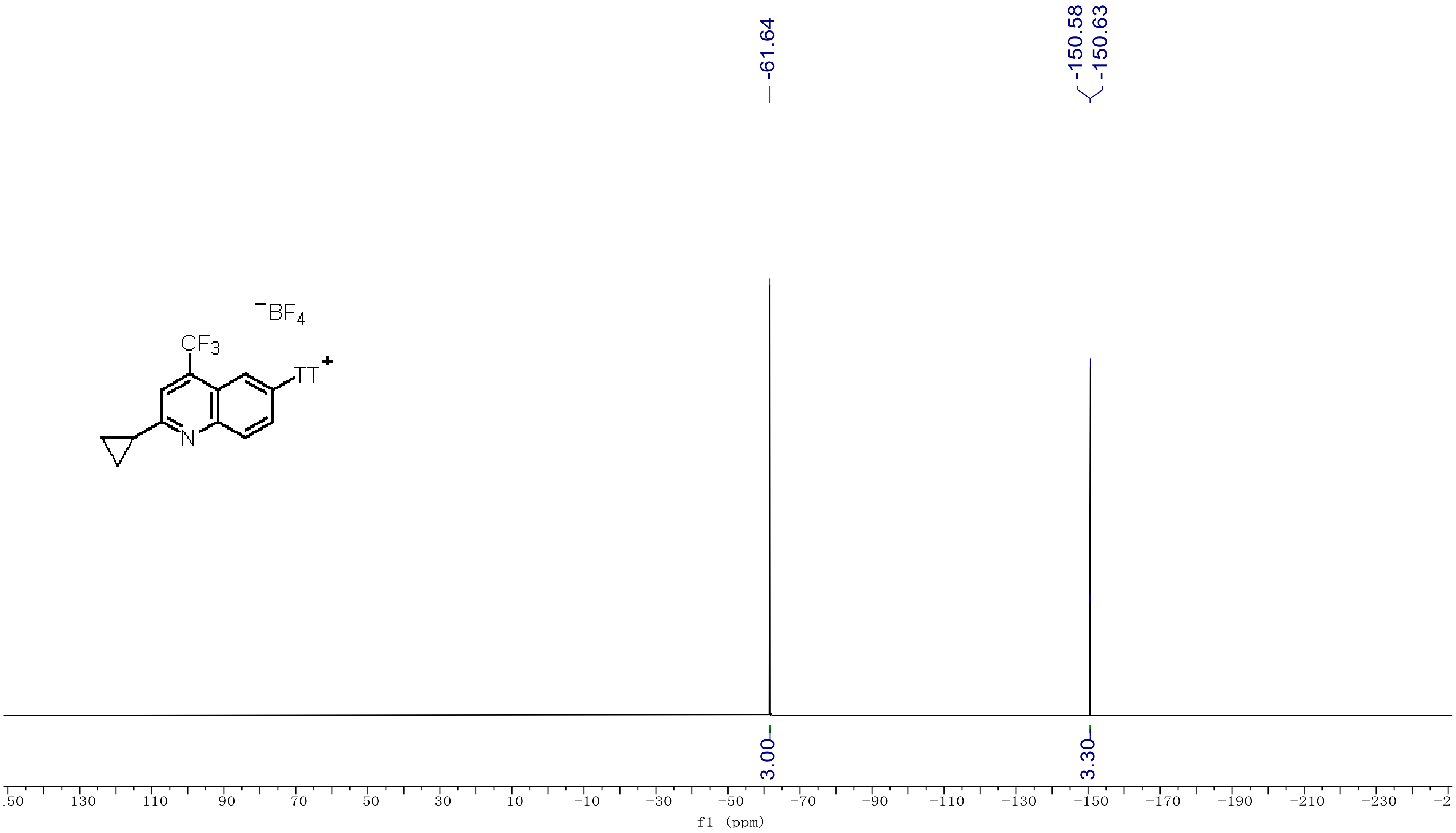

## $^{13}$C NMR of quinoline-derived thianthrenium salt TT-14

$CDCl_3$, 23 ºC

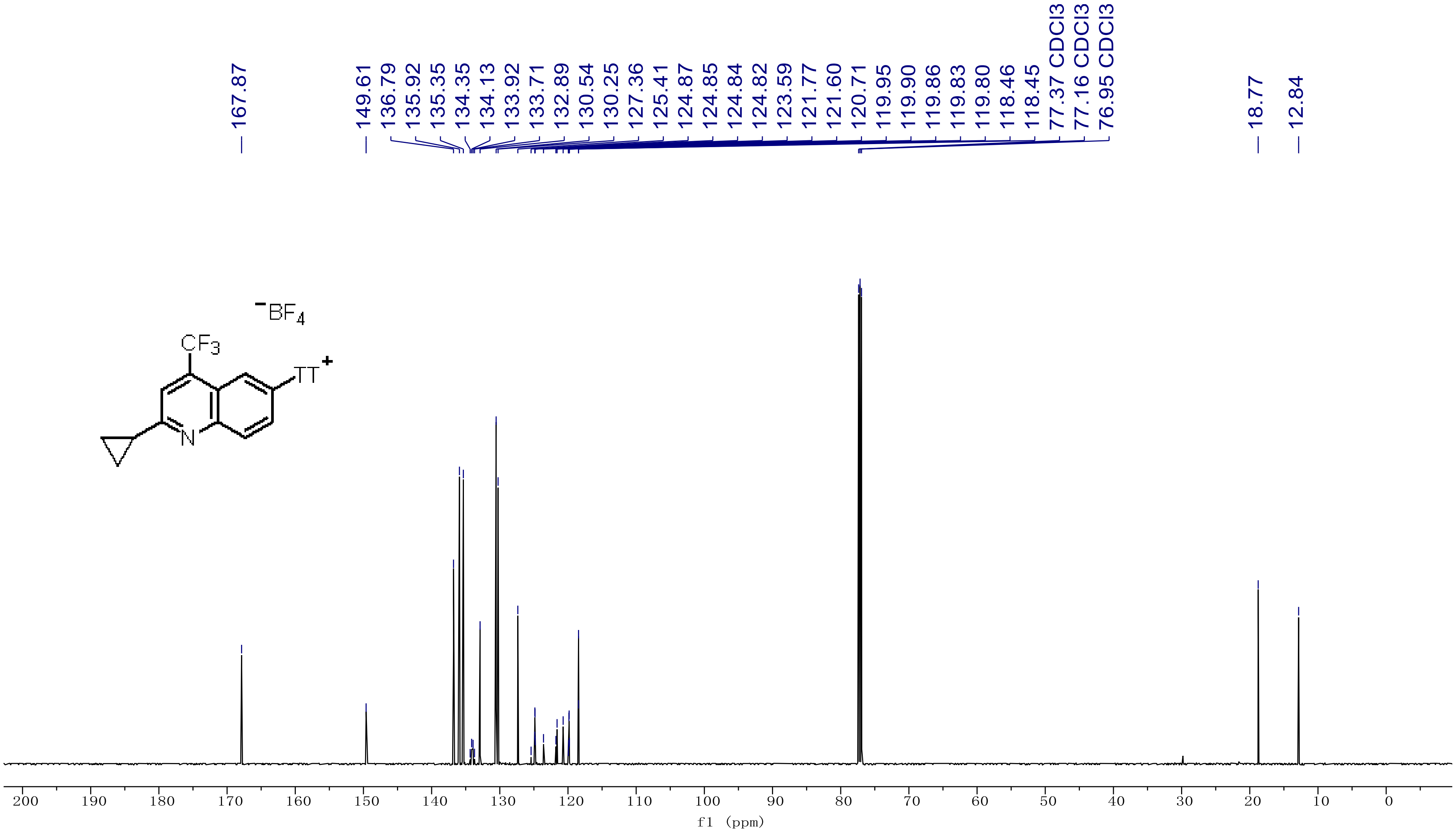

# $^{1}H$–$^{13}C$ HSQC of quinoline-derived thianthrenium salt TT-14

$CDCl_3$, 23 ºC

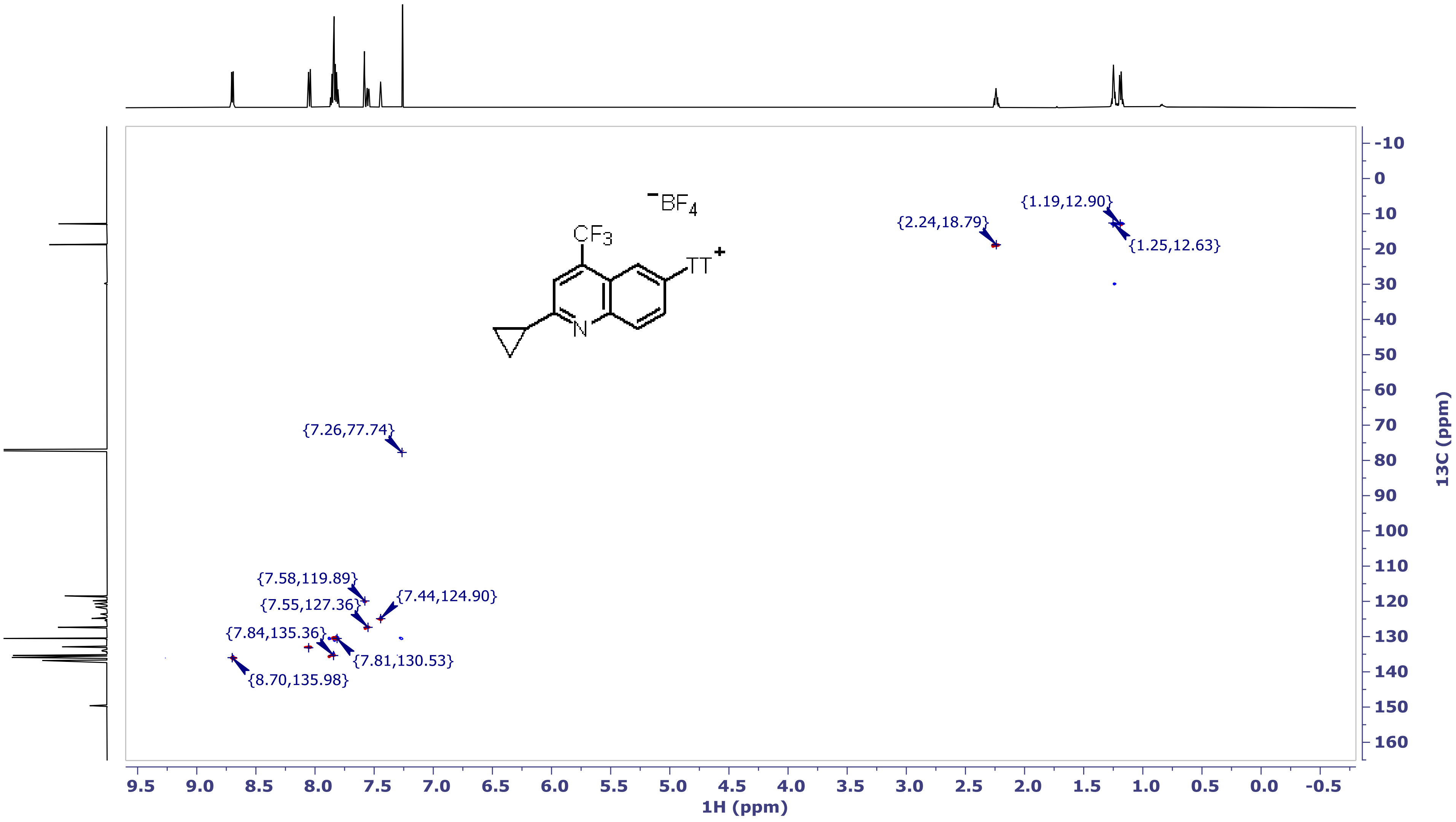

# $^{1}H$–$^{13}C$ HMBC of quinoline-derived thianthrenium salt TT-14

CDCl$_3$, 23 ºC

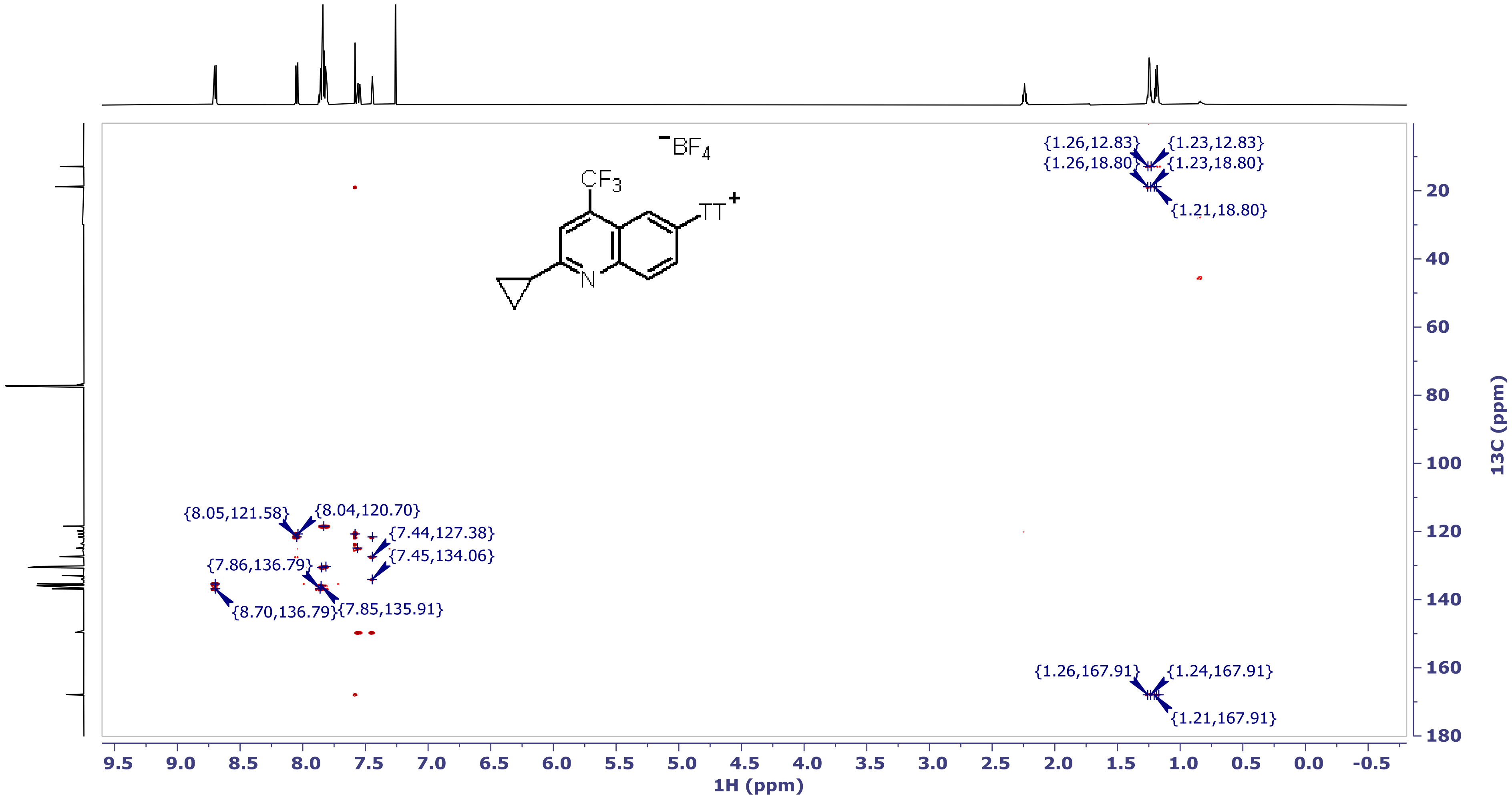

**$^1$H–$^1$H COSY of quinoline-derived thianthrenium salt TT-14**

$CDCl_3$, 23 °C

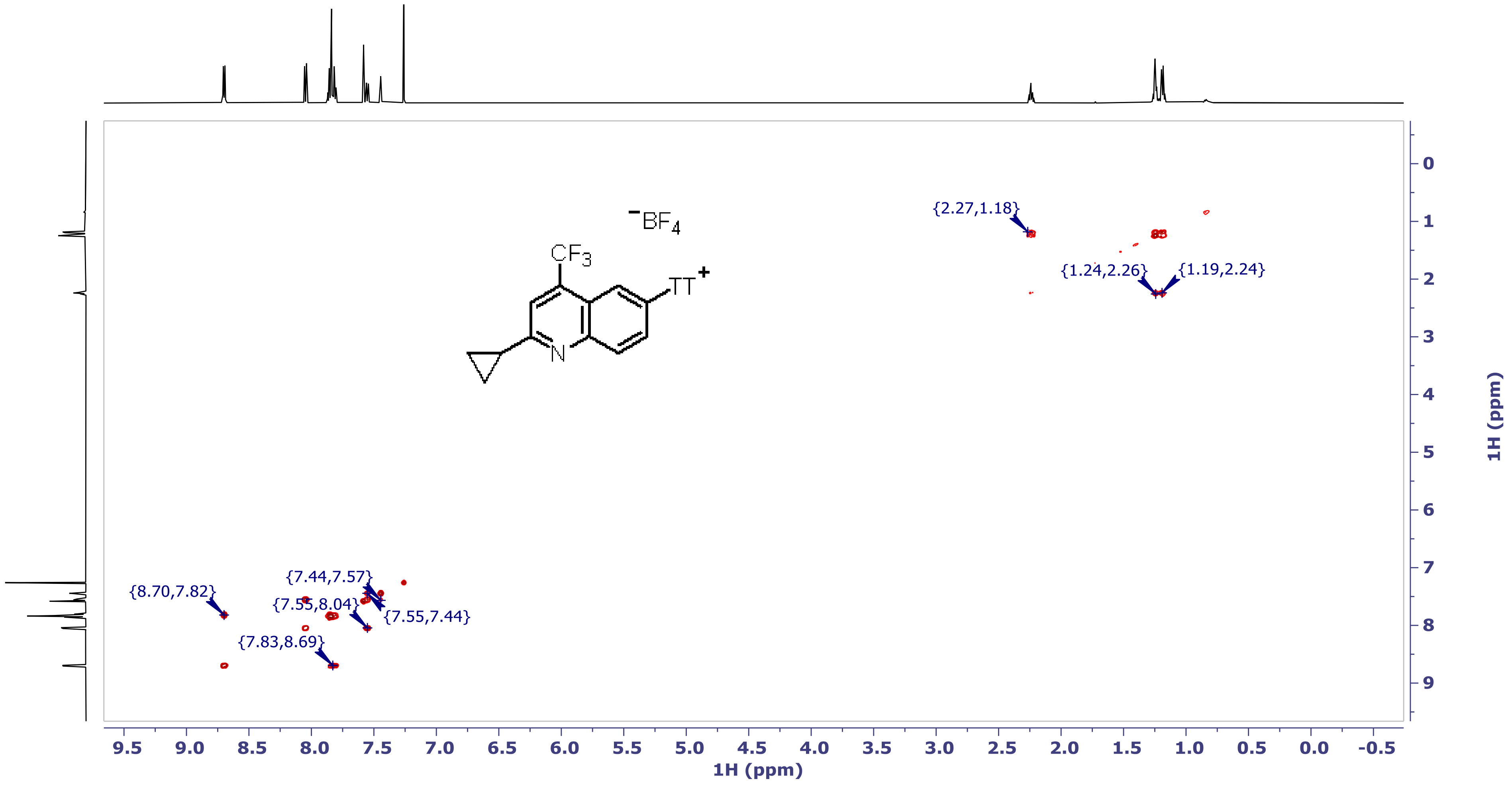

## $^1$H–$^1$H NOSEY of quinoline-derived thianthrenium salt TT-14

CDCl$_3$, 23 ºC

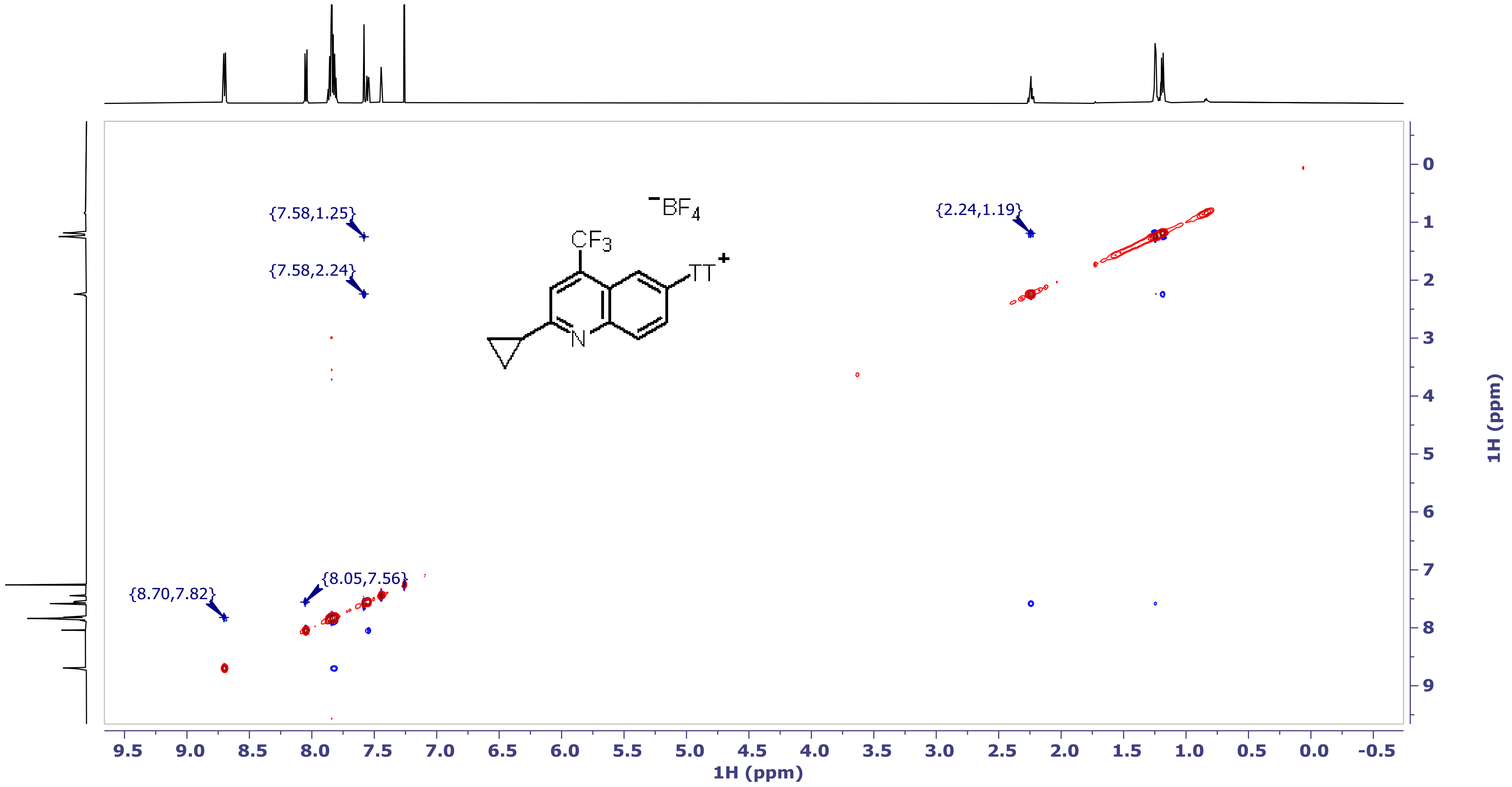

**$^{1}$H NMR of 4-methoxydiphenyl ether-derived thianthrenium salt TT-19**

$CDCl_3$, 23 ºC

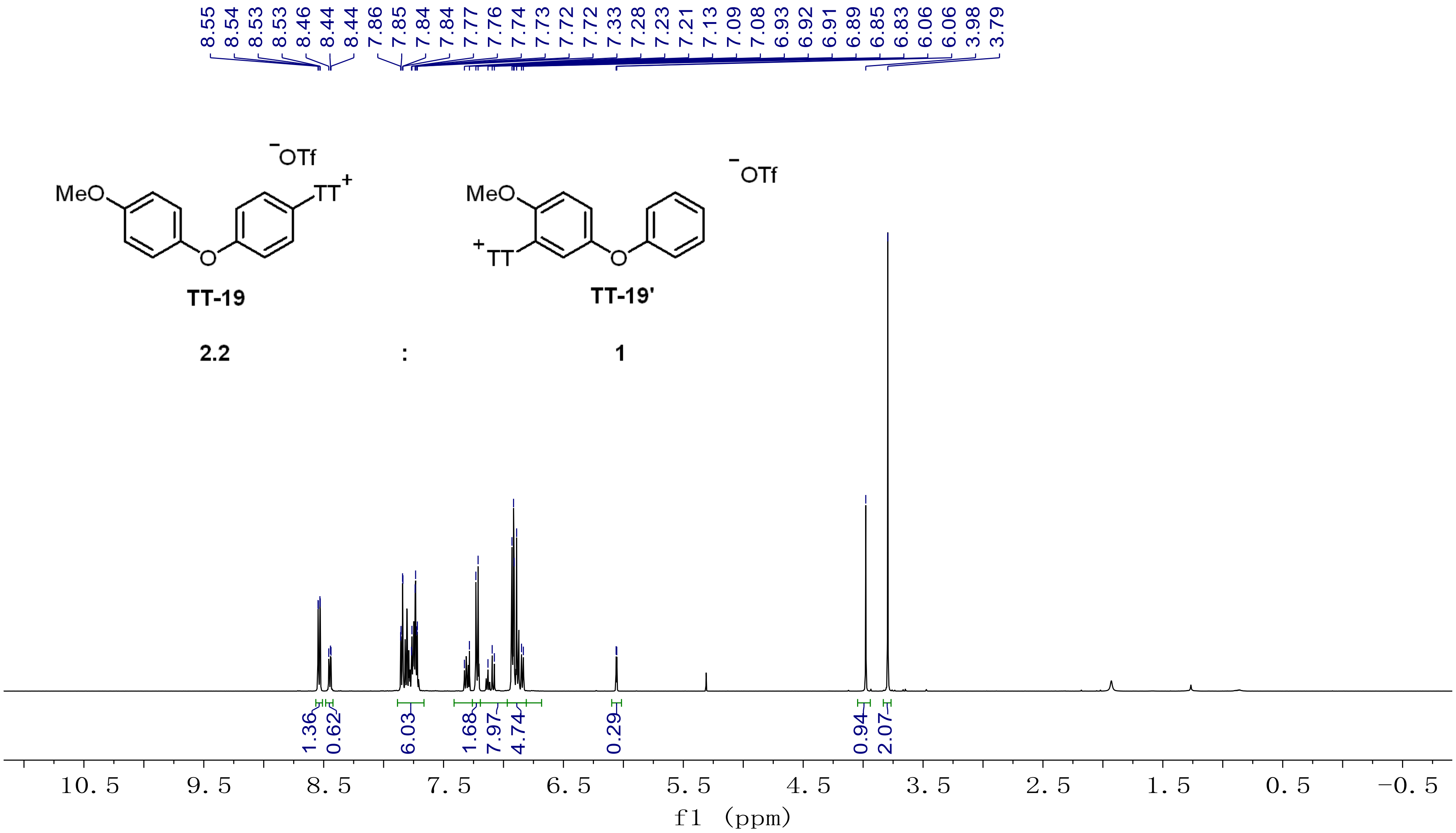

### $^{19}$F NMR of 4-methoxydiphenyl ether-derived thianthrenium salt TT-19

$CDCl_3$, 23 ºC

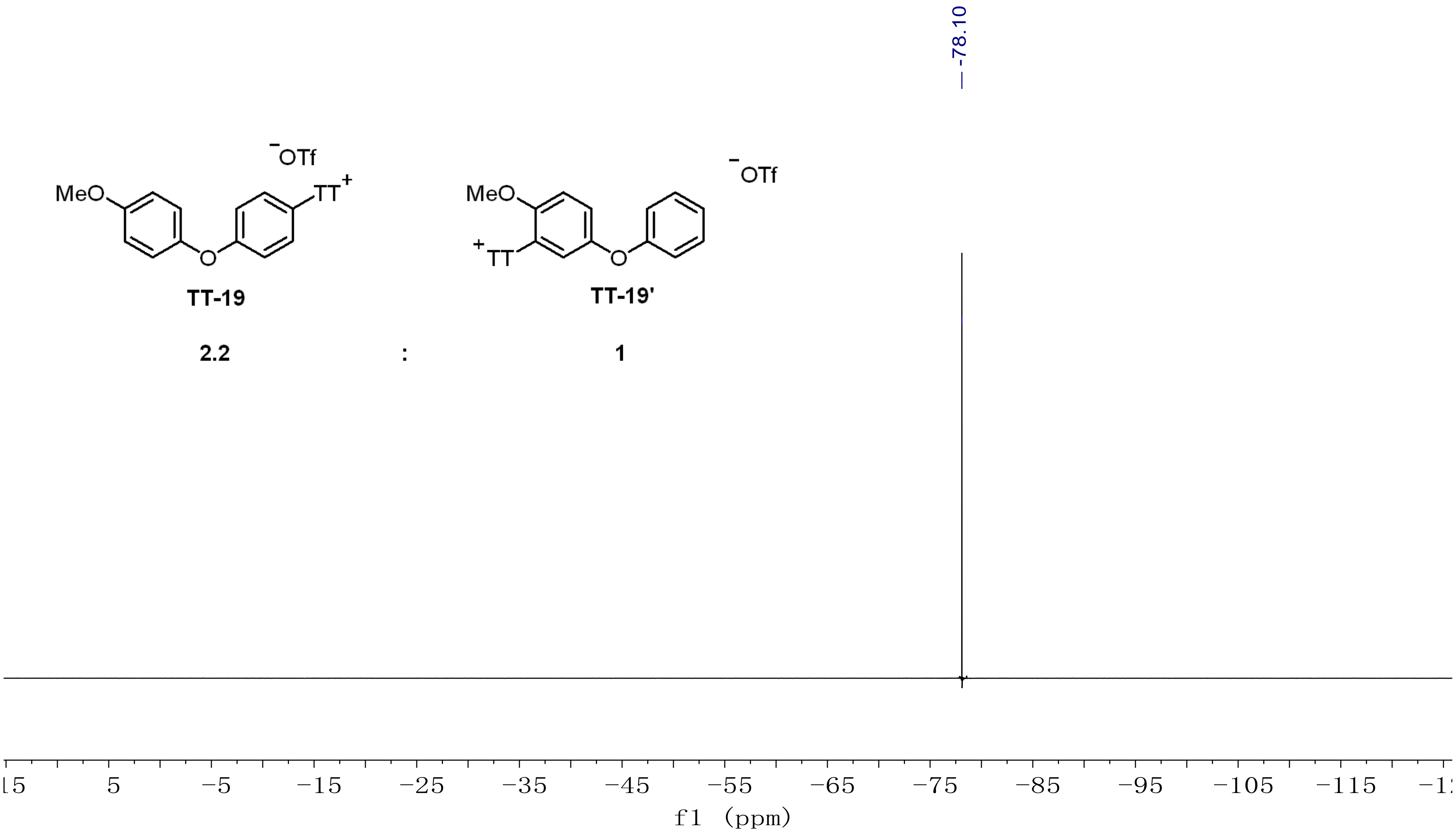

## $^{13}C$ NMR of 4-methoxydiphenyl ether-derived thianthrenium salt TT-19

$CDCl_3$, 23 ºC

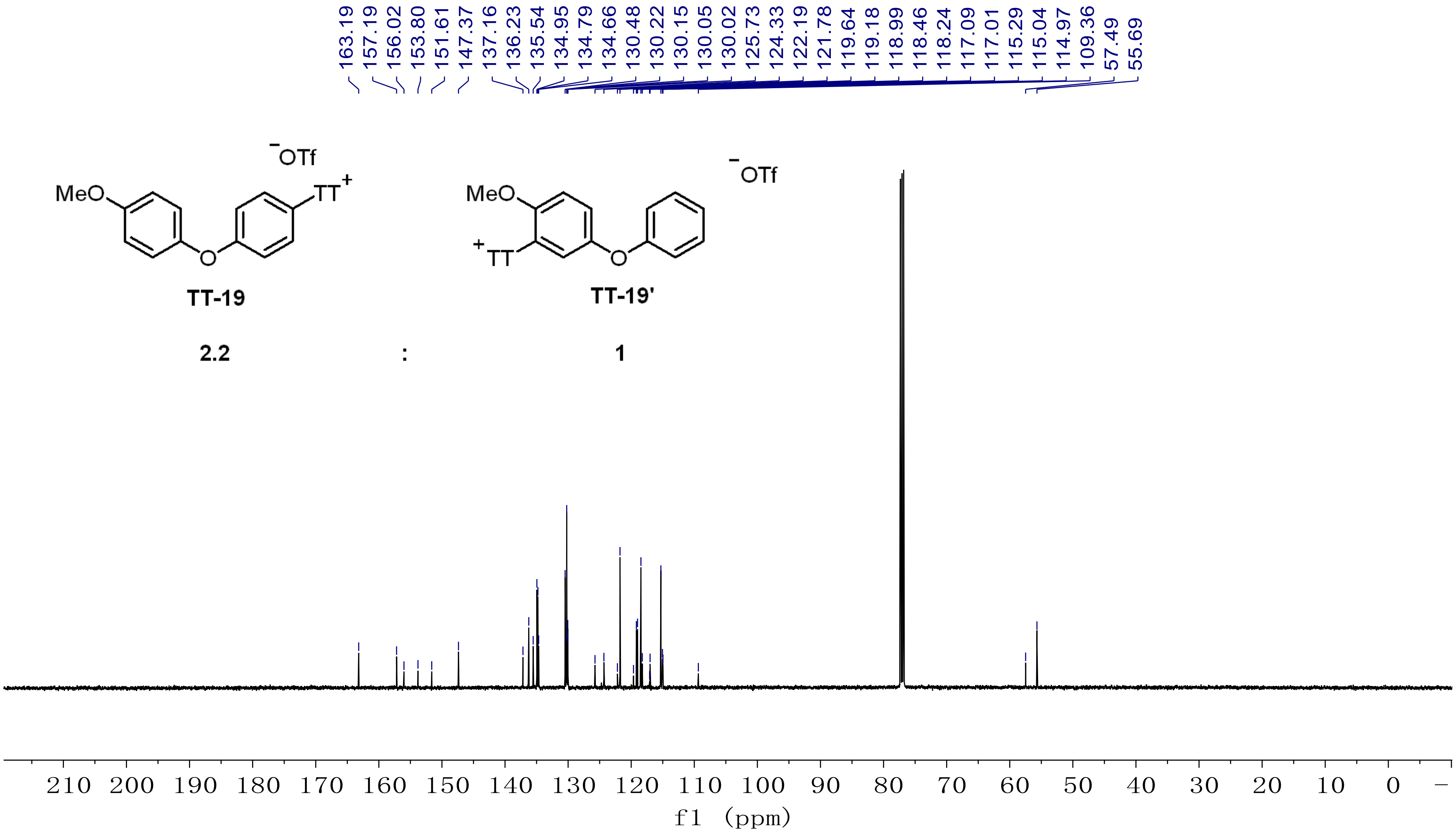

**$^1$H NMR of 2-chloroanisole-derived thianthrenium salt TT-21**

$CDCl_3$, 23 ºC

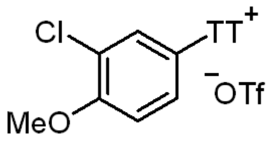

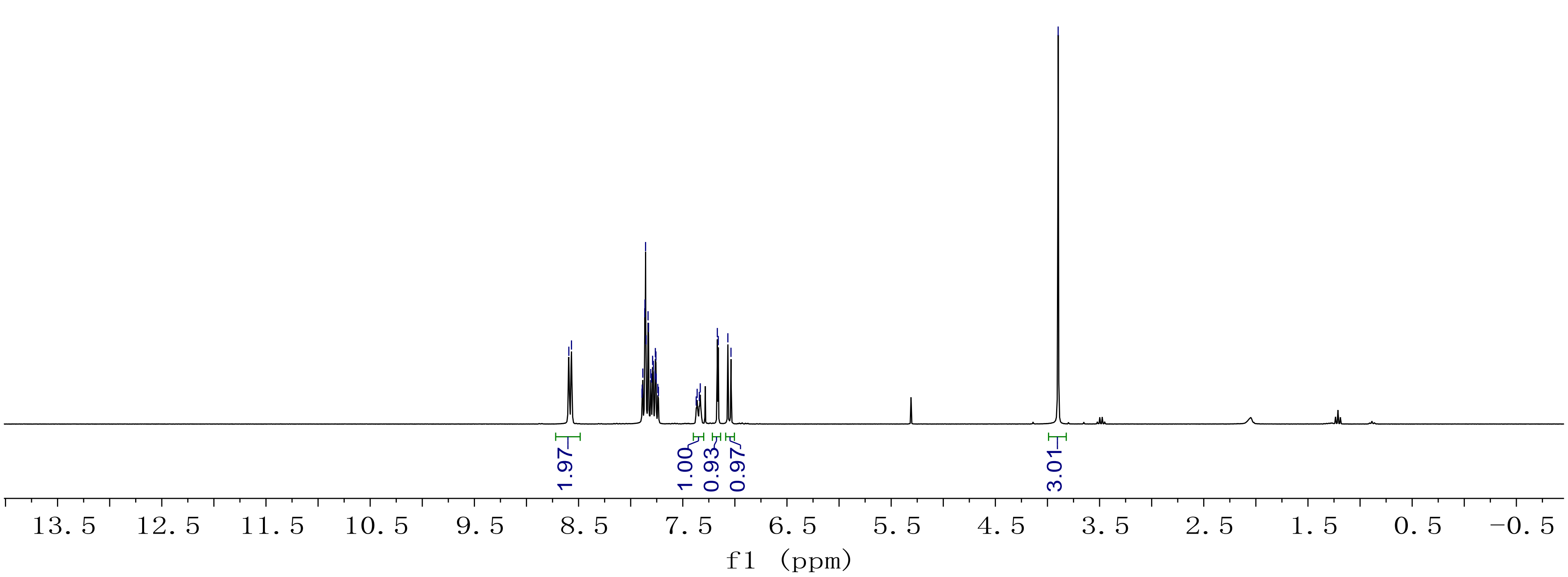

**$^{19}$F NMR of 2-chloroanisole-derived thianthrenium salt TT-21**

$CDCl_3$, 23 ºC

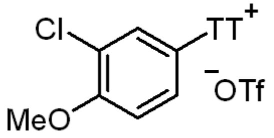


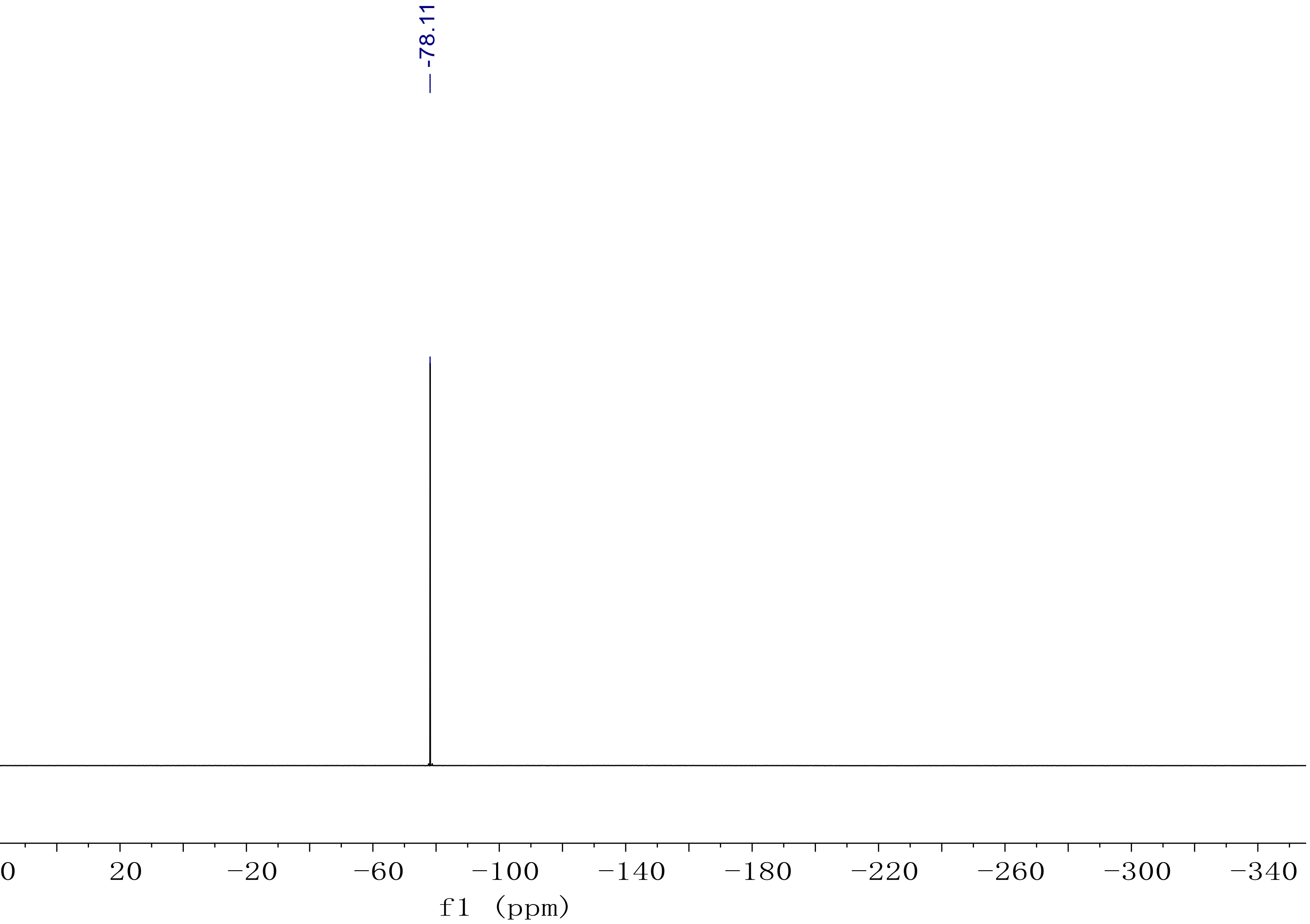

**$^{13}$C NMR of 2-chloroanisole-derived thianthrenium salt TT-21**

$CDCl_3$, 23 ºC

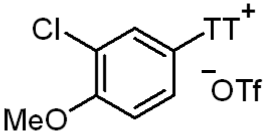


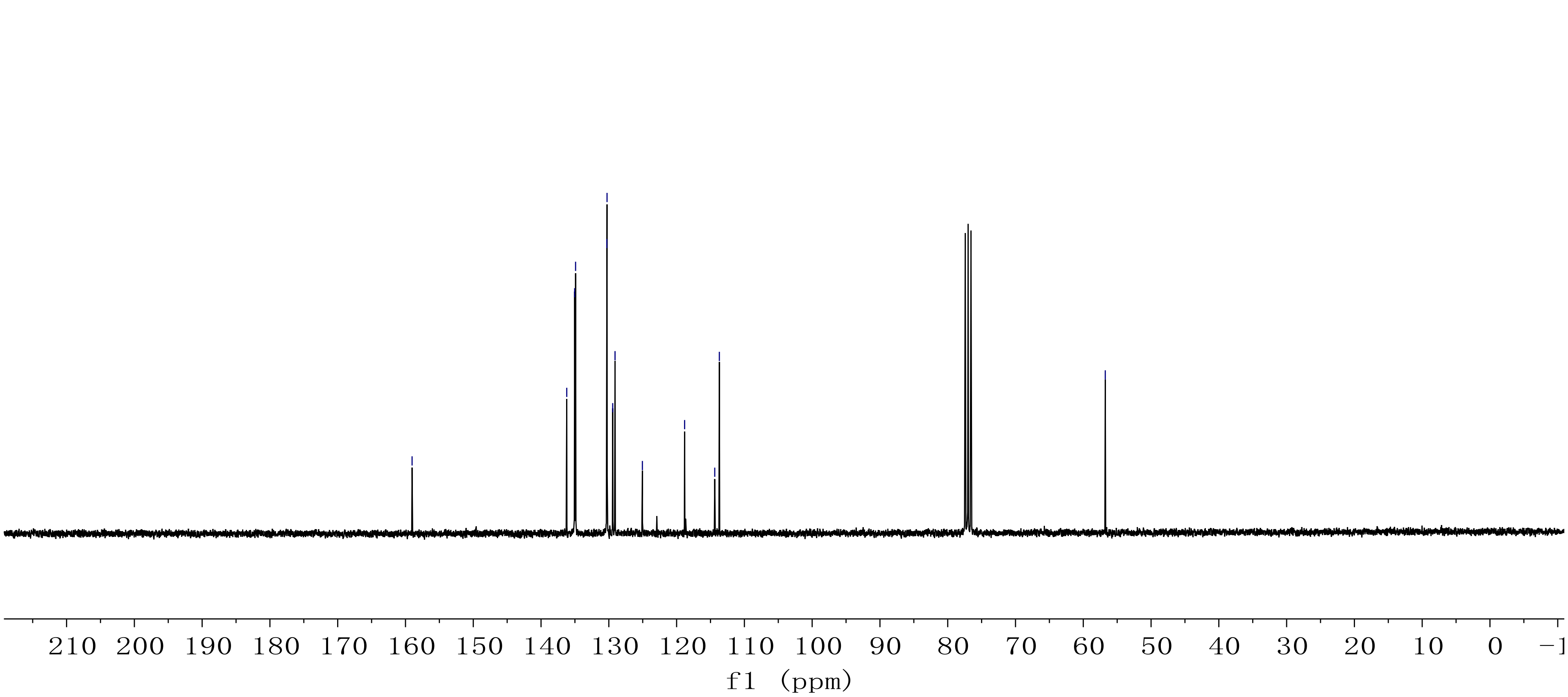

**$^{1}$H NMR of thianaphthene-derived thianthrenium salt TT-25**

$CDCl_3$, 23 ºC

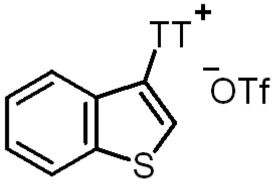


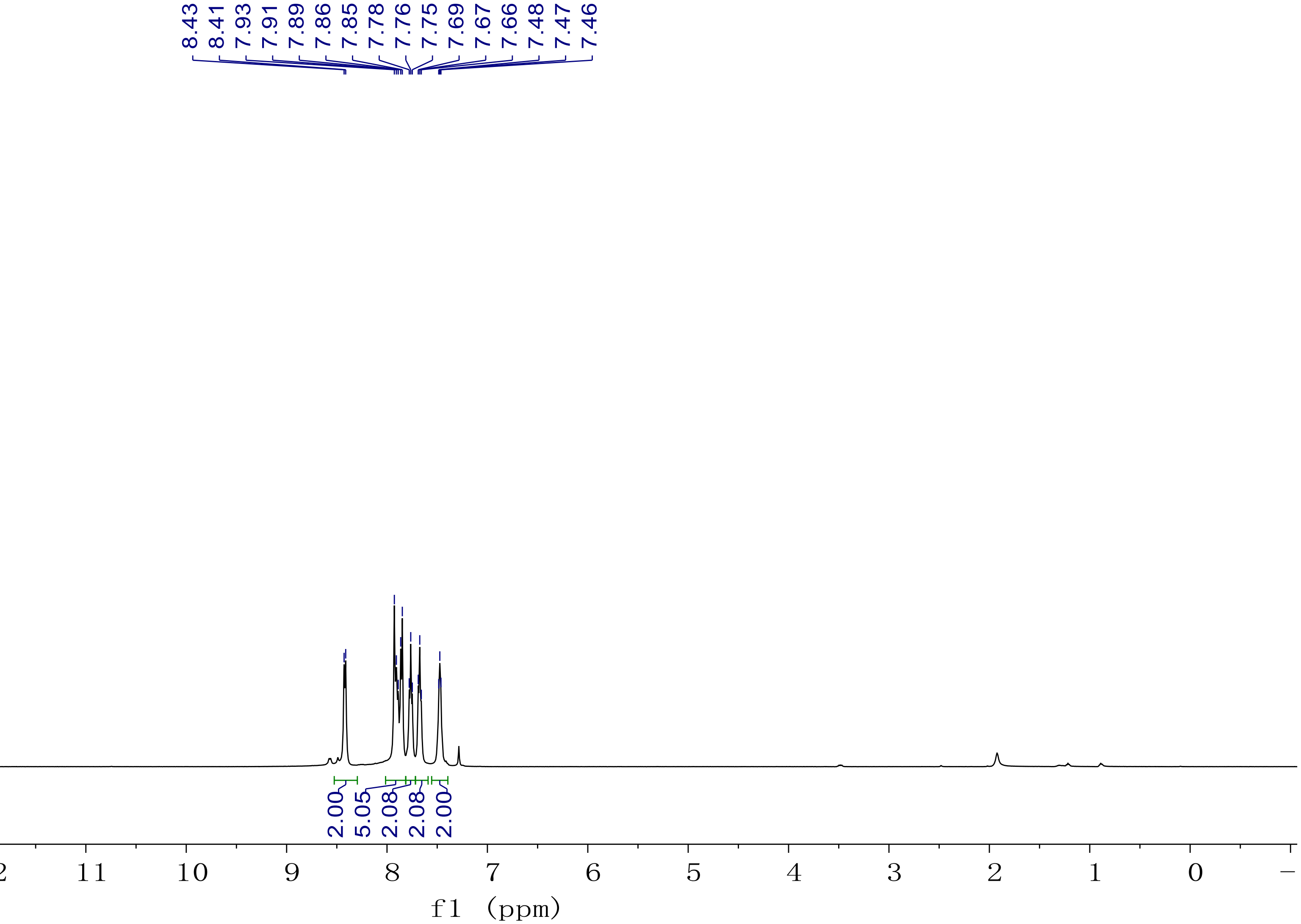

**$^{19}$F NMR of thianaphthene-derived thianthrenium salt TT-25**

$CDCl_3$, 23 ºC

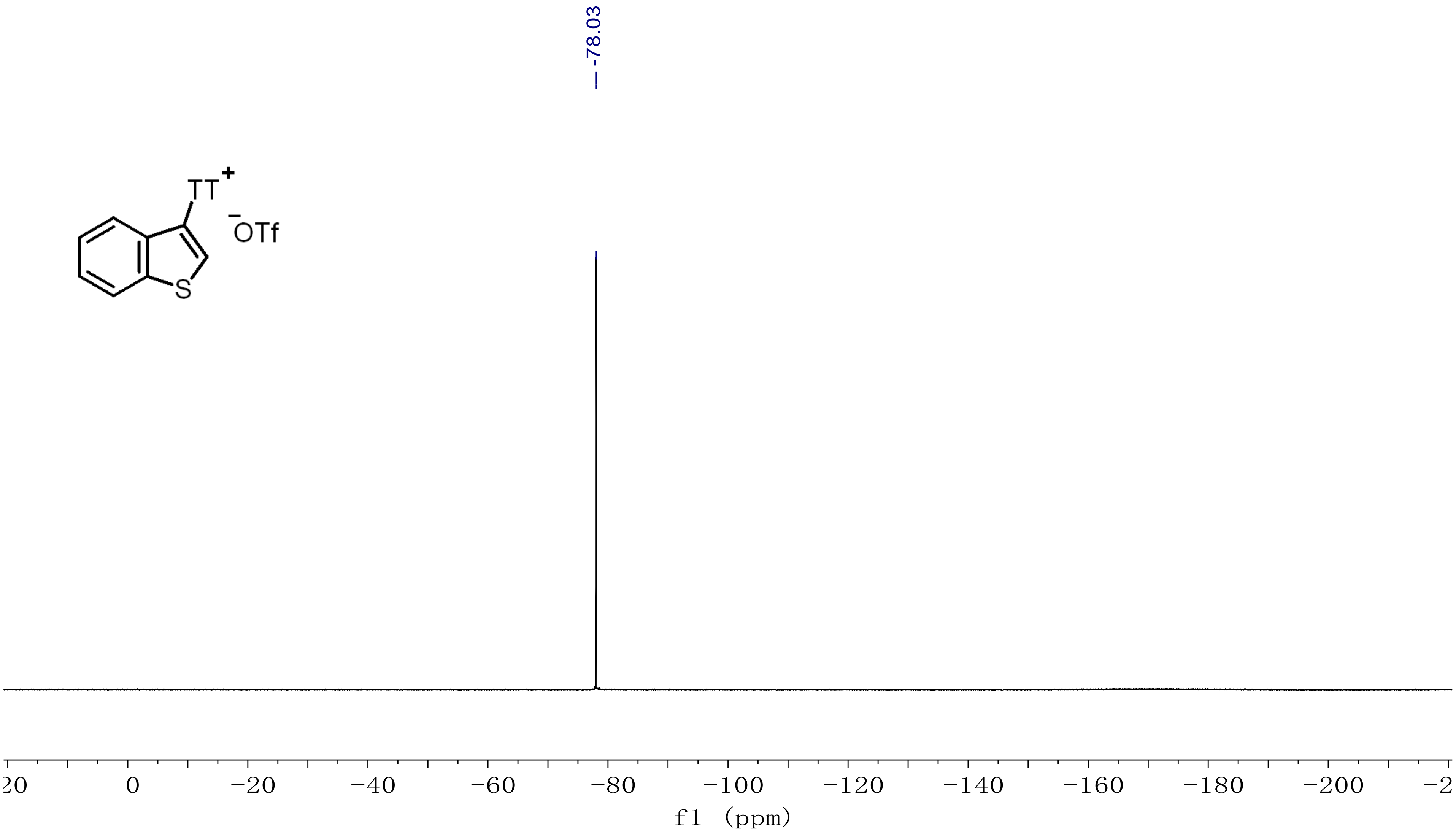

**$^{13}$C NMR of thianaphthene-derived thianthrenium salt TT-25**

$CDCl_3$, 23 ºC

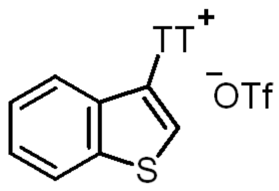

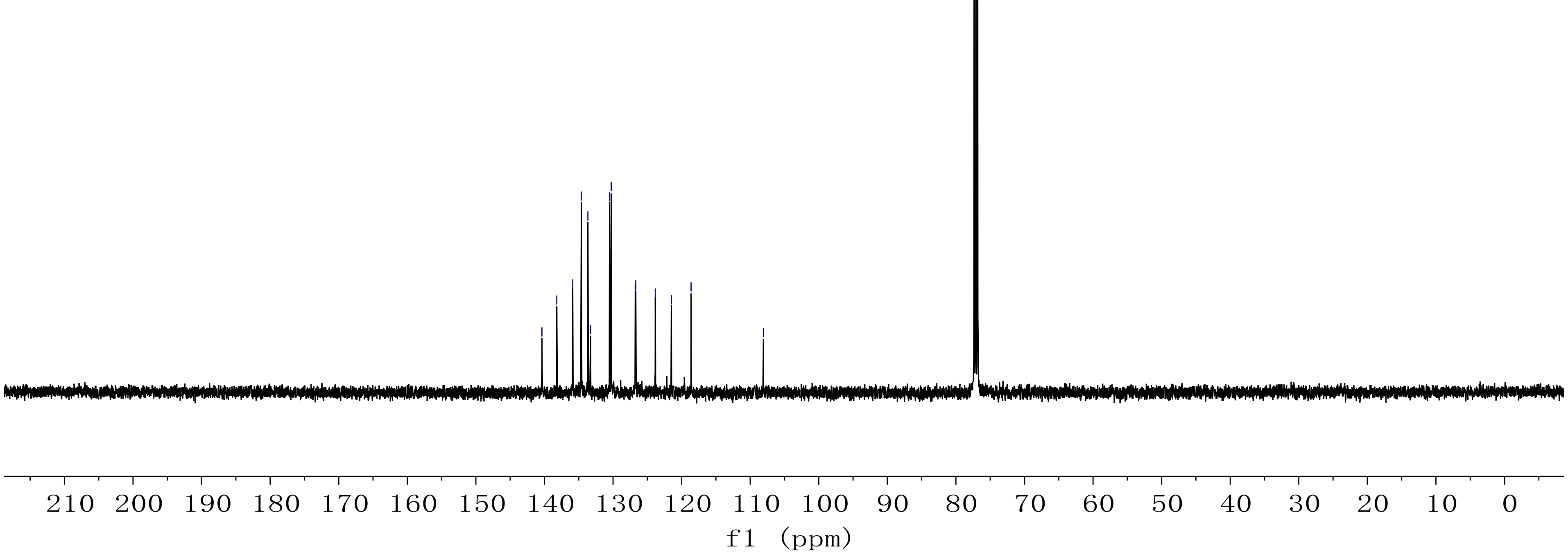

# $^{1}$H NMR of pyrazol-derived thianthrenium salt TT-26

CDCl$_3$, 23 ºC

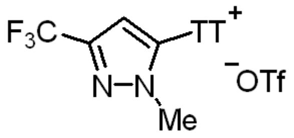


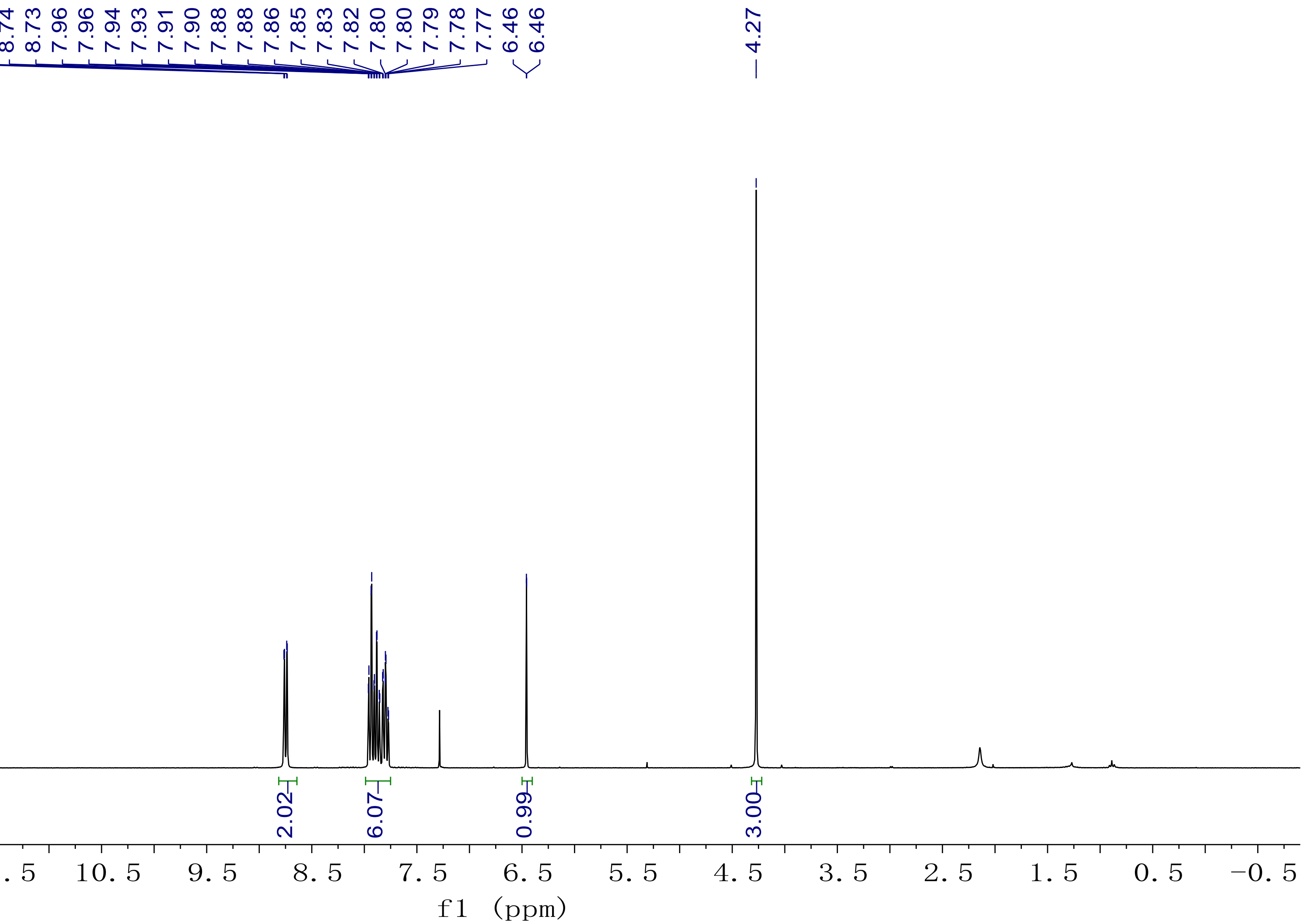

**$^{19}$F NMR of pyrazol-derived thianthrenium salt TT-26**

$CDCl_3$, 23 ºC

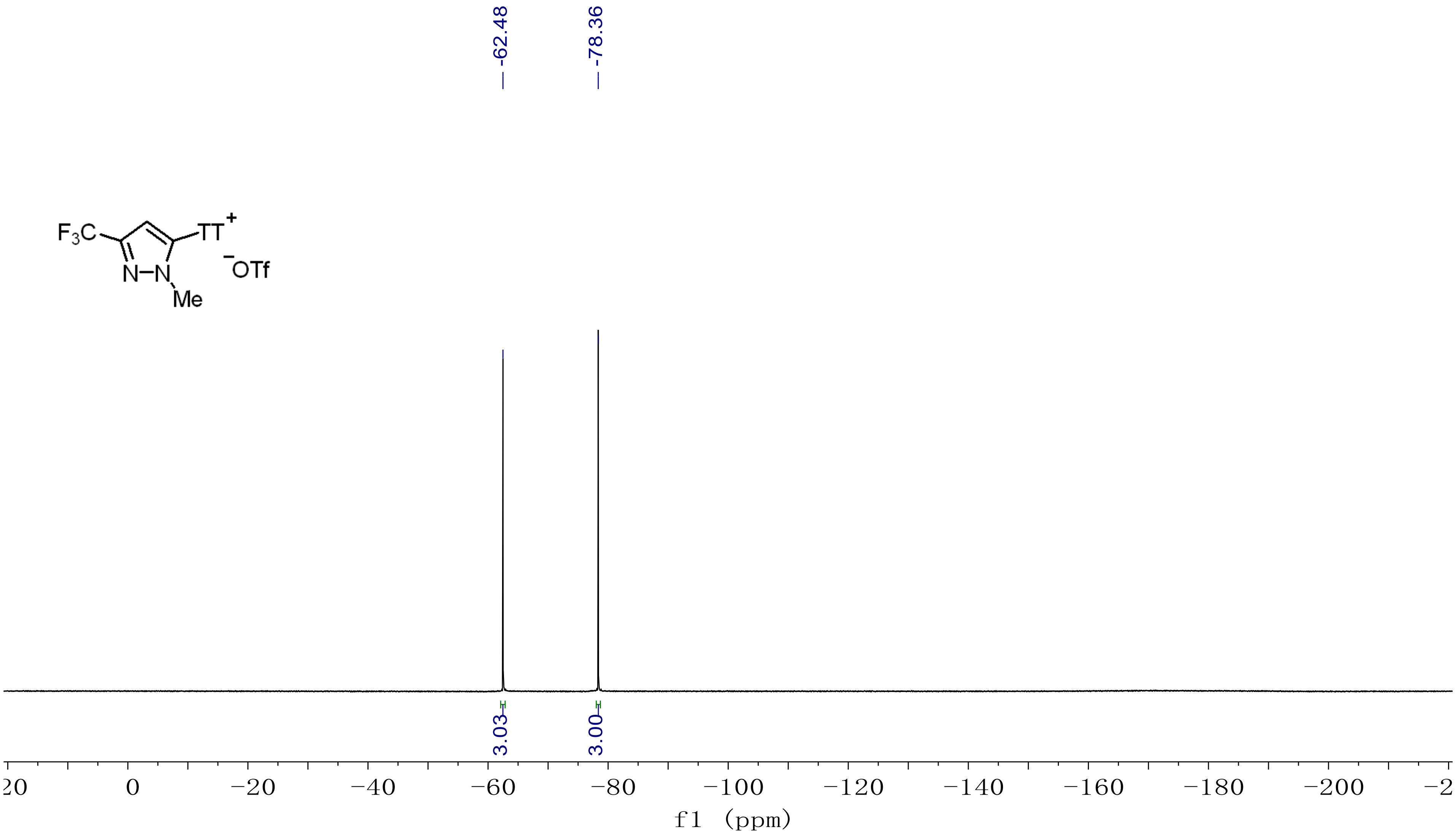

**$^{13}$C NMR of pyrazol-derived thianthrenium salt TT-26**

$CDCl_3$, 23 ºC

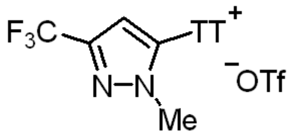


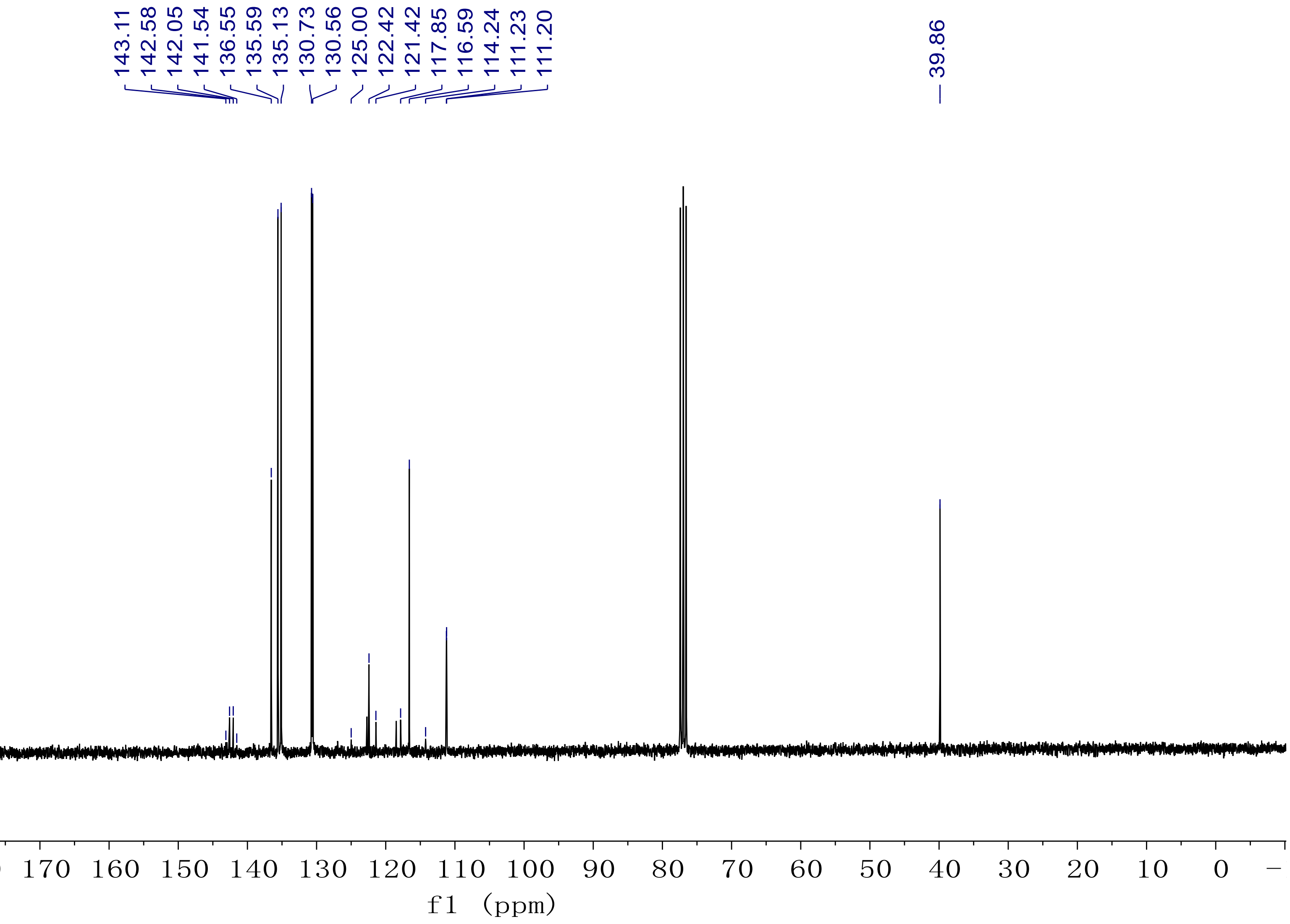

**$^{1}$H NMR of isoquinoline-derived thianthrenium salt TT-27**

$CDCl_3$, 23 ºC

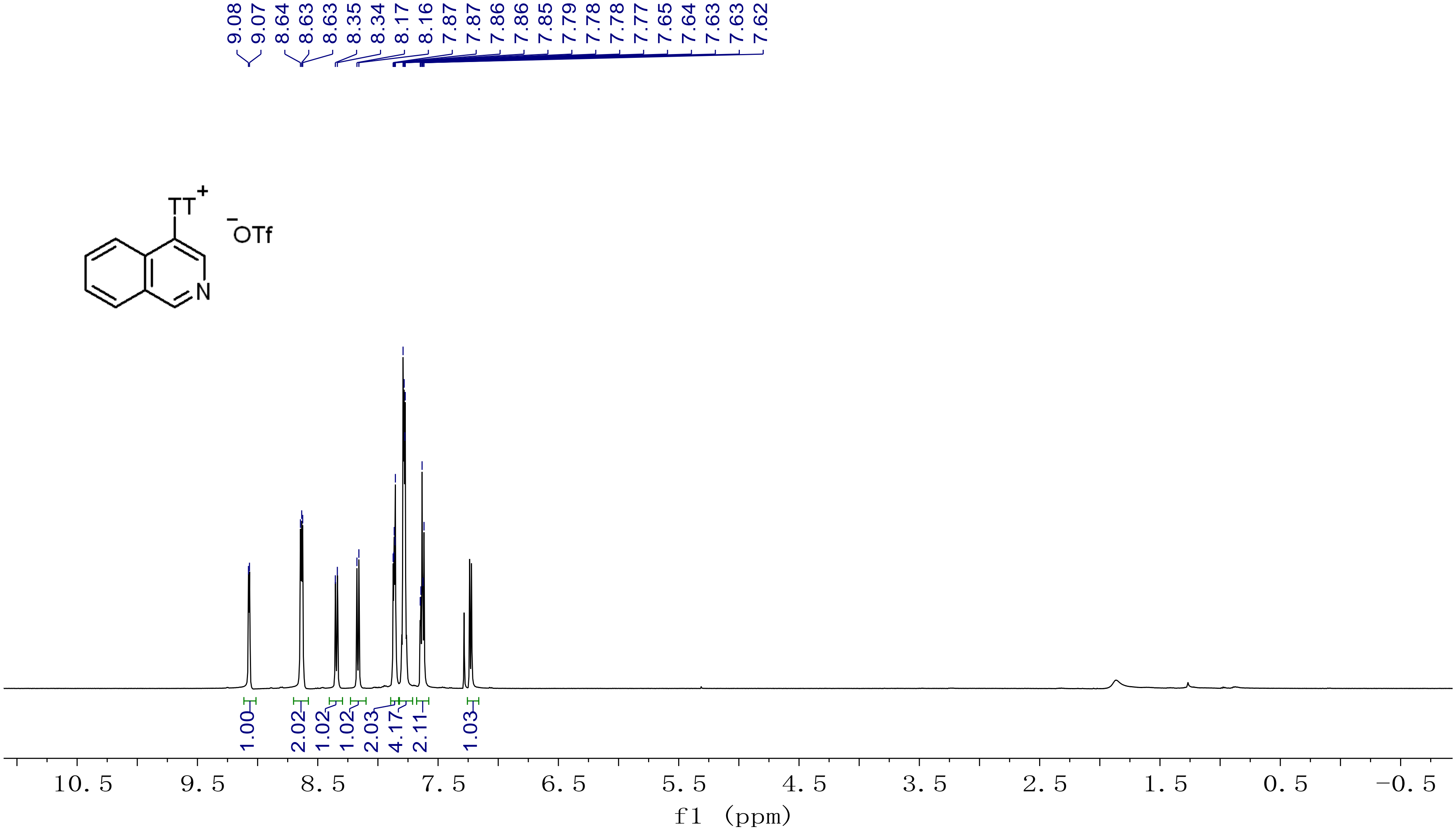

**$^{19}$F NMR of isoquinoline-derived thianthrenium salt TT-27**

$CDCl_3$, 23 ºC

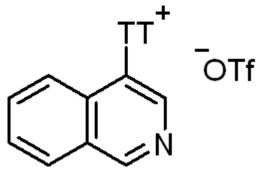


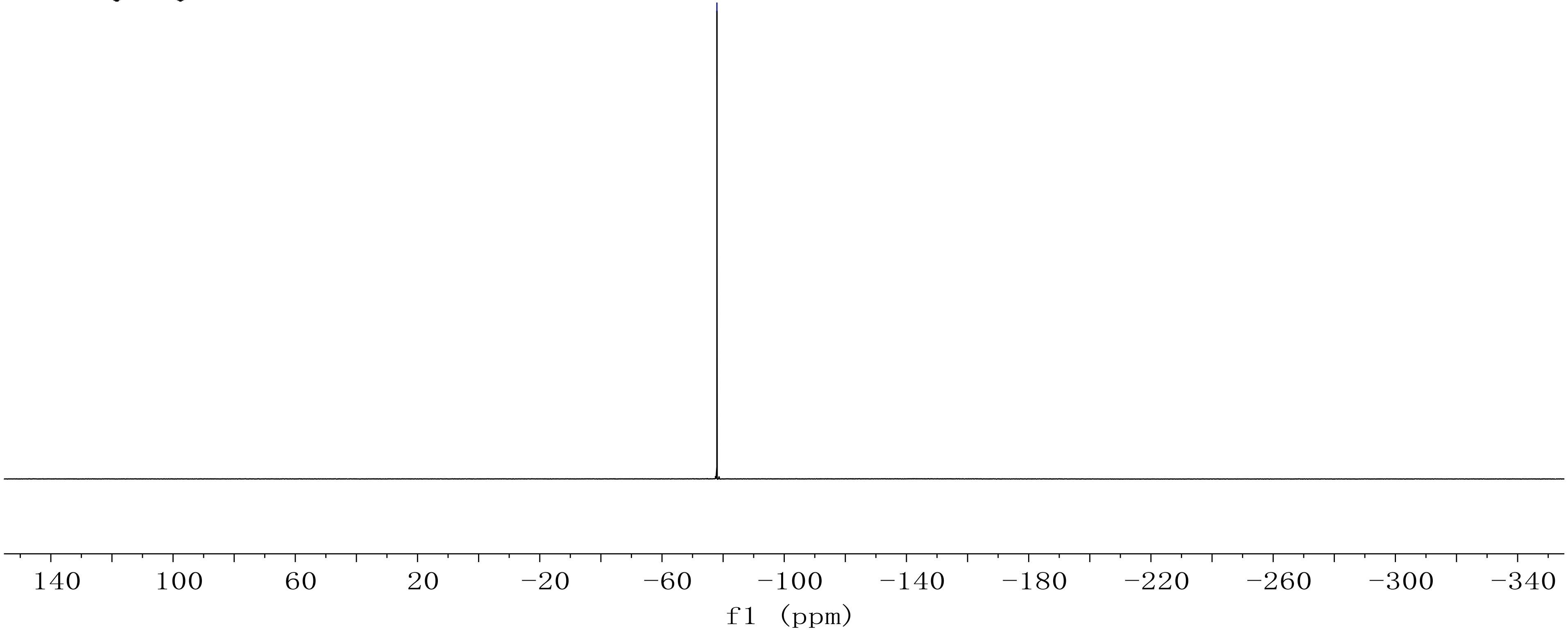

**$^{13}$C NMR of isoquinoline-derived thianthrenium salt TT-27**

$CDCl_3$, 23 ºC

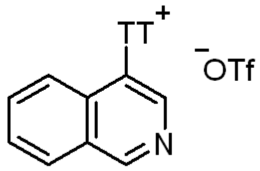


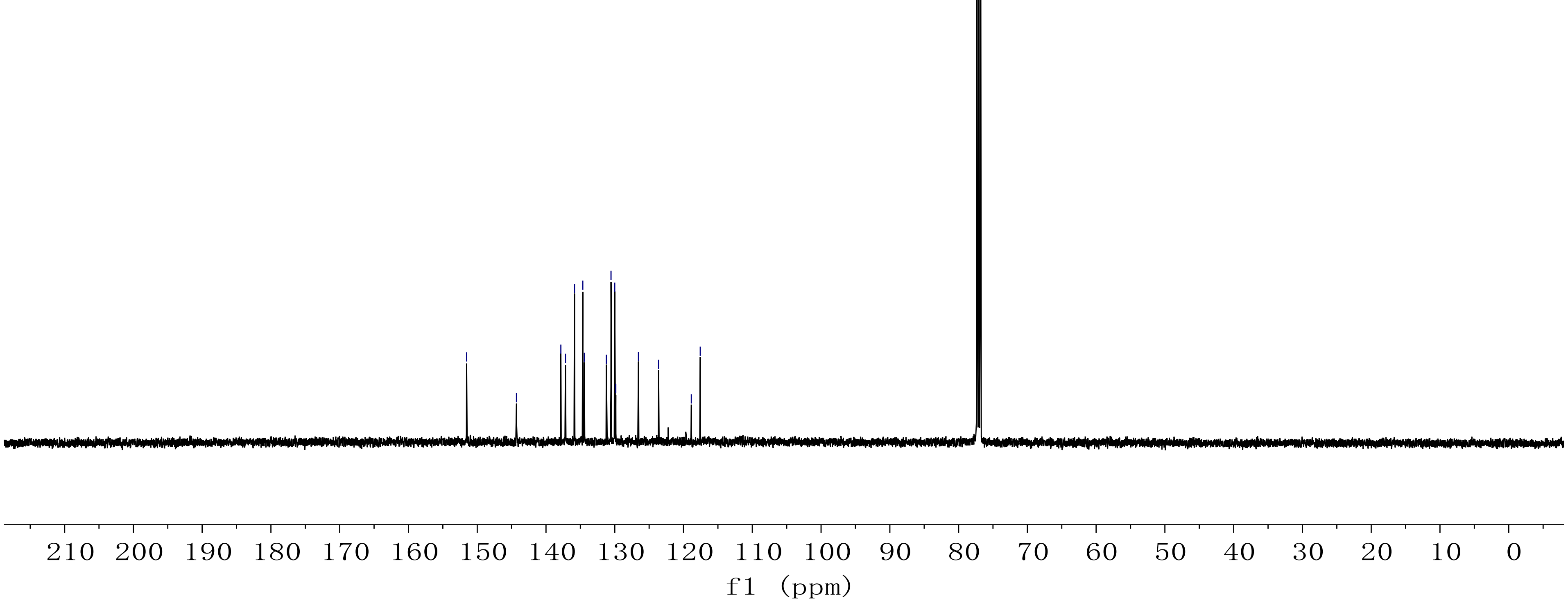

**$^{1}$H NMR of quinoline-derived thianthrenium salt TT-28**

$CDCl_3$, 23 ºC

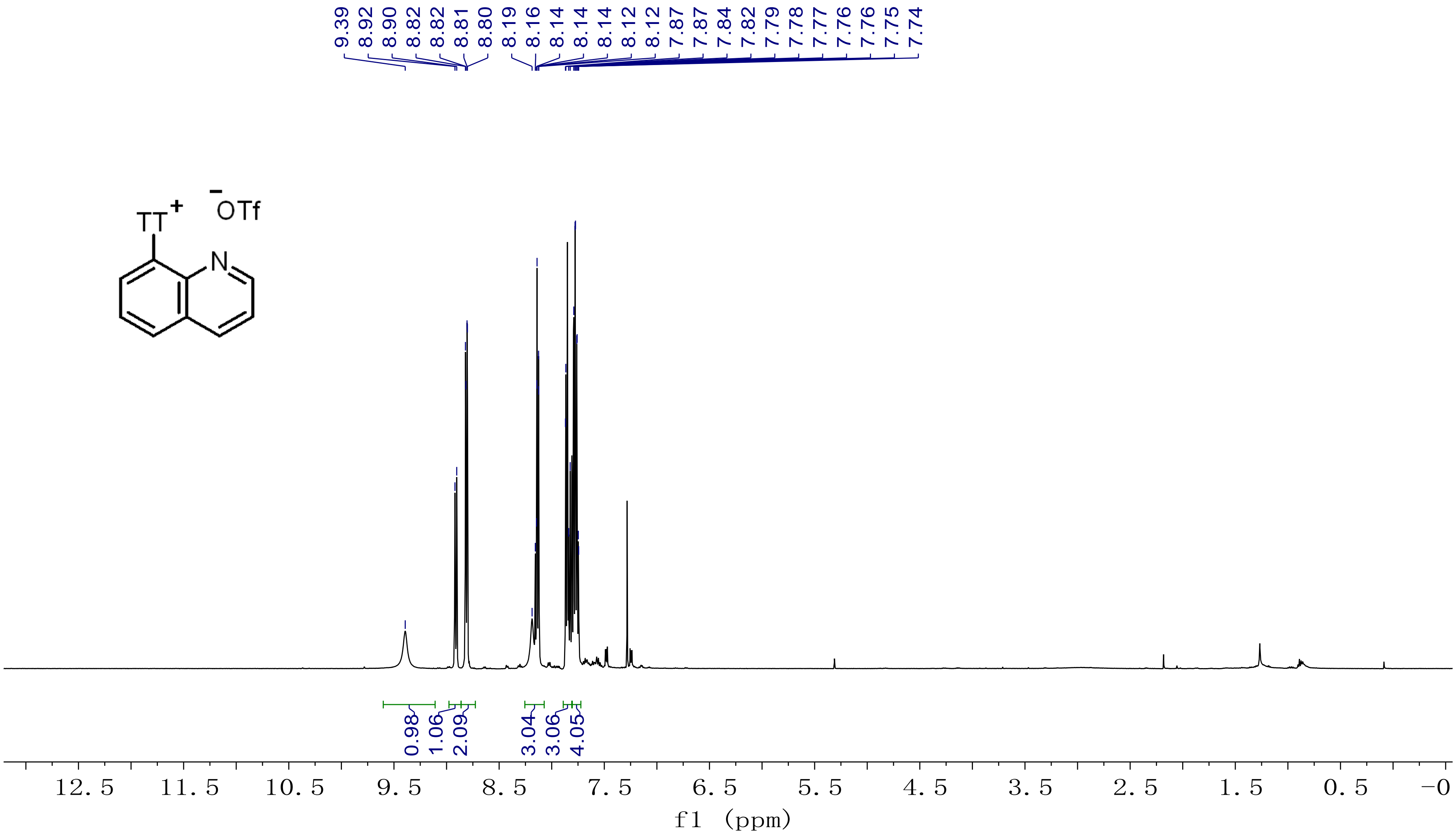

**$^{19}$F NMR of quinoline-derived thianthrenium salt TT-28**

$CDCl_3$, 23 ºC

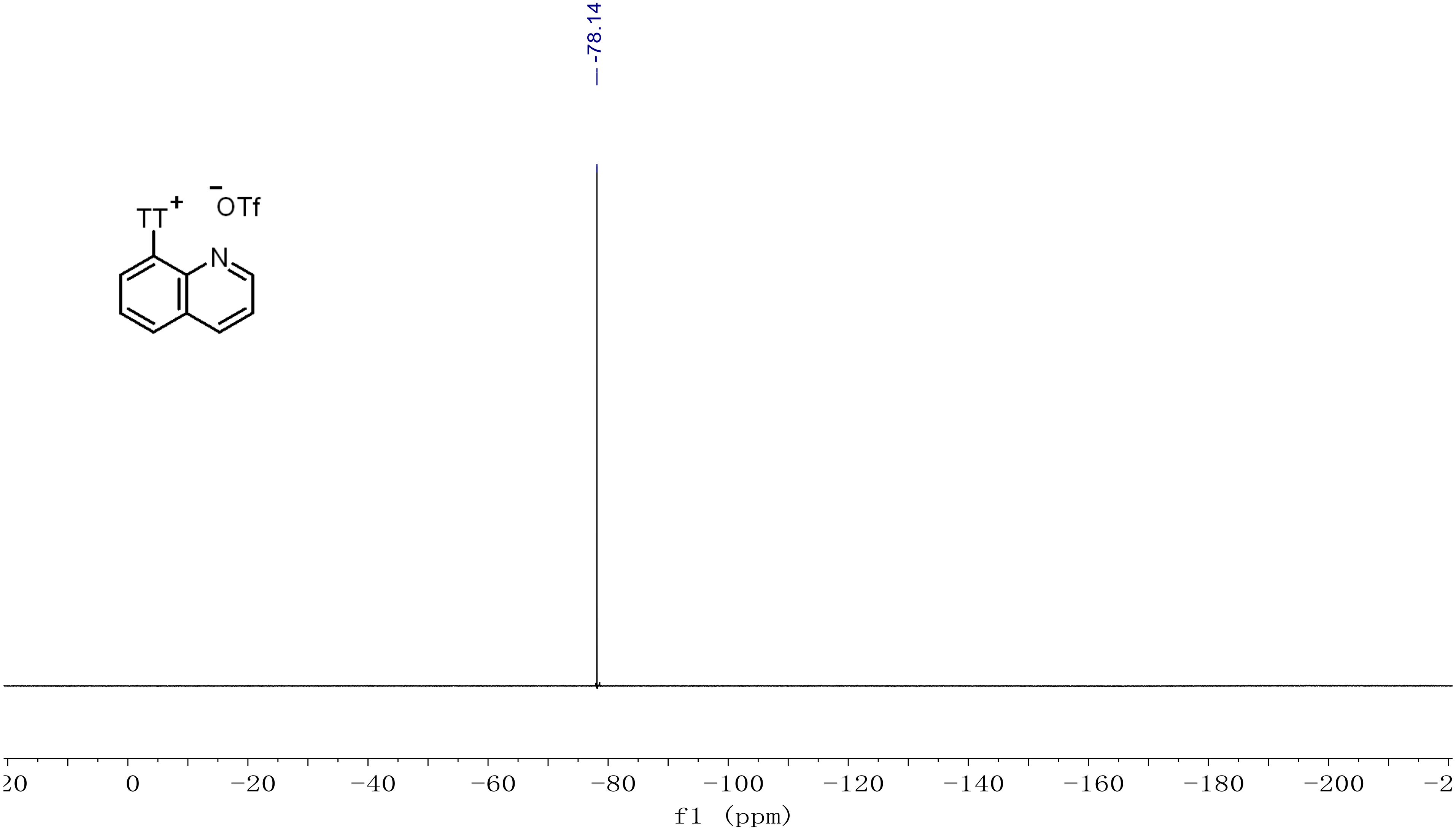

**$^{13}$C NMR of quinoline-derived thianthrenium salt TT-28**

$CDCl_3$, 23 ºC

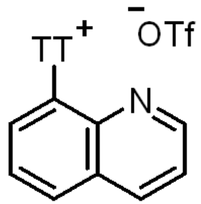


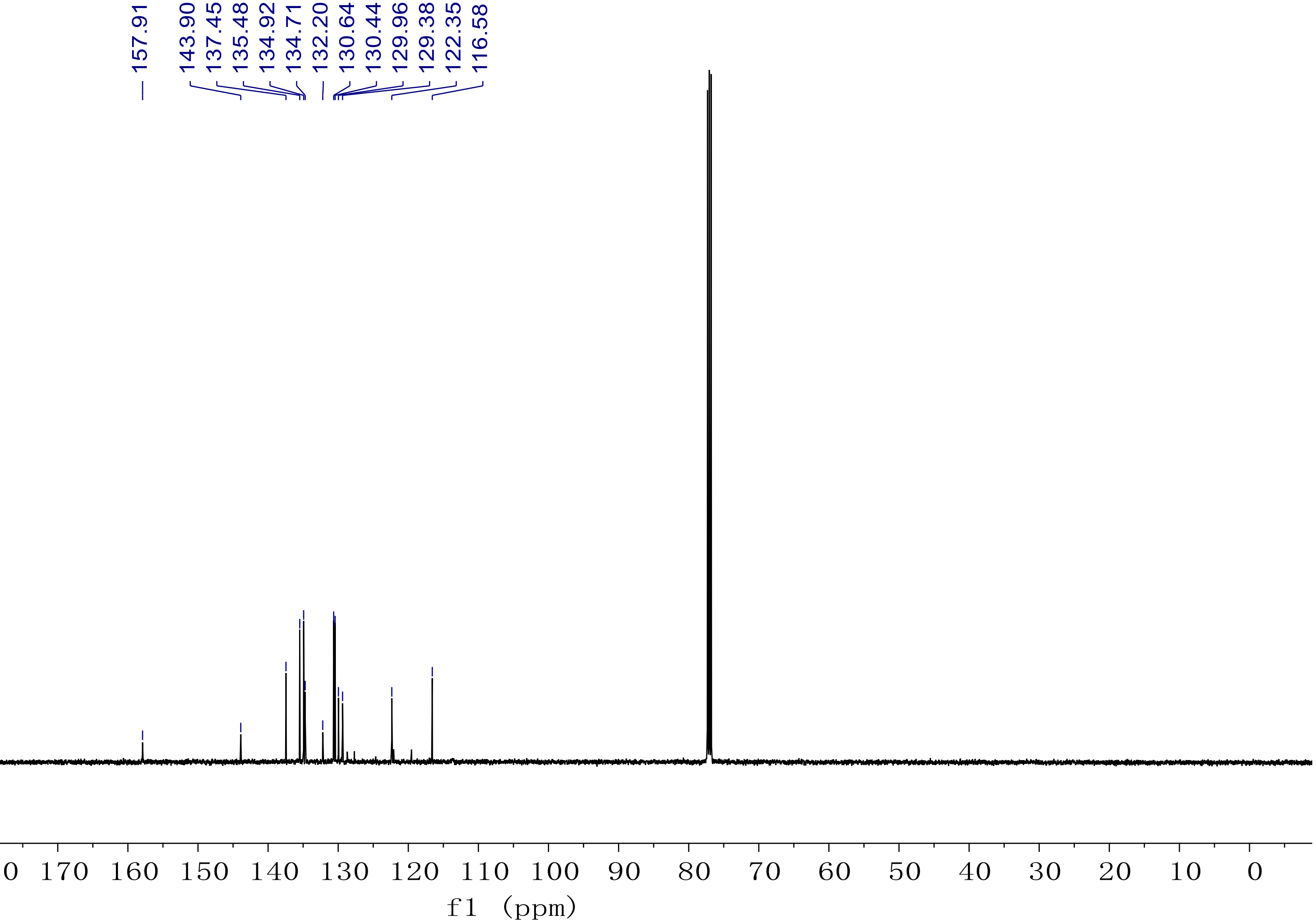

### $^1$H NMR of pyridine-derived thianthrenium salt TT-29

CDCl$_3$, 23 ºC

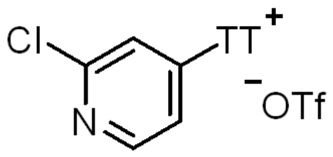


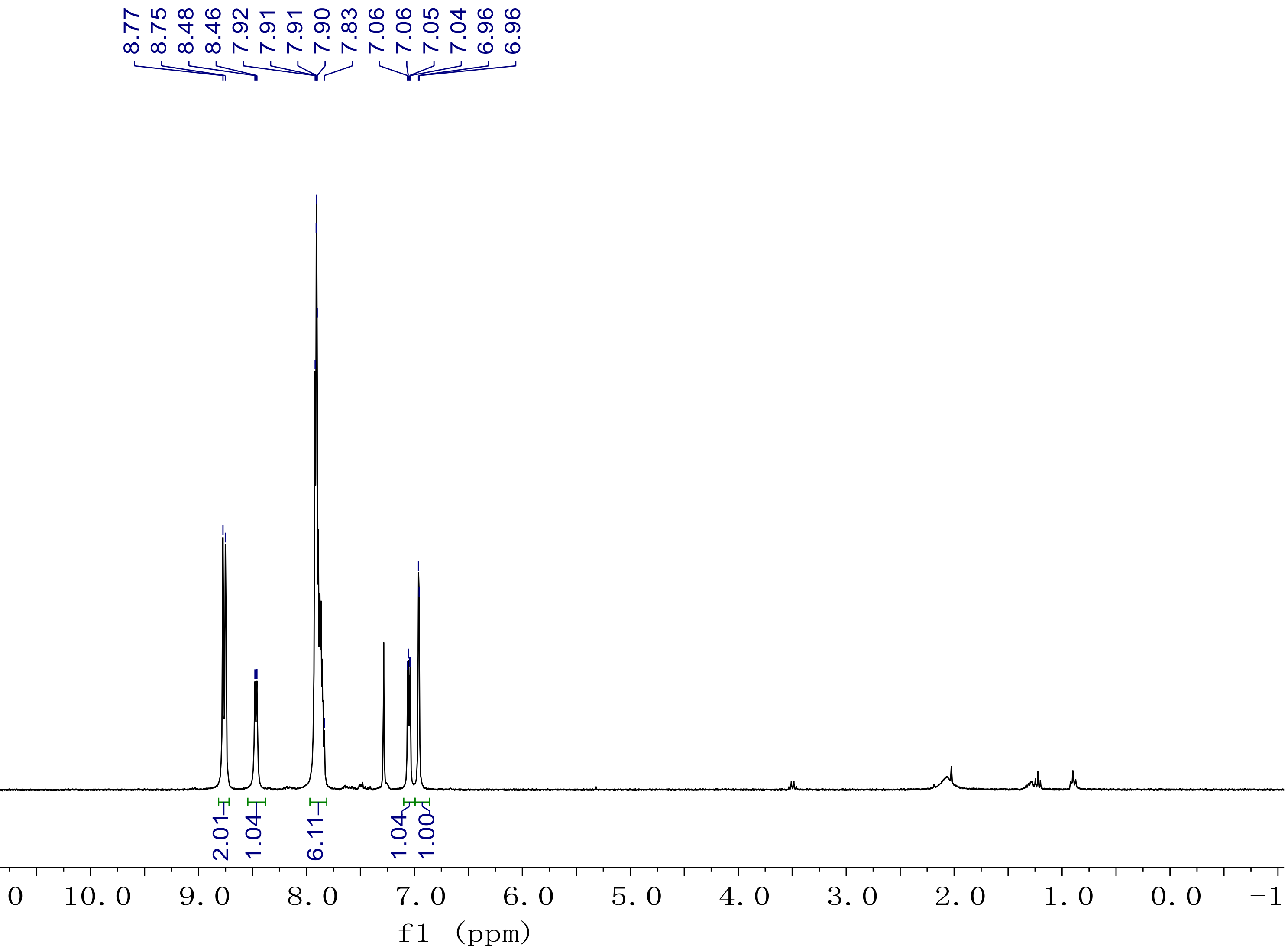

## $^{19}$F NMR of pyridine-derived thianthrenium salt TT-29

$CDCl_3$, 23 ºC

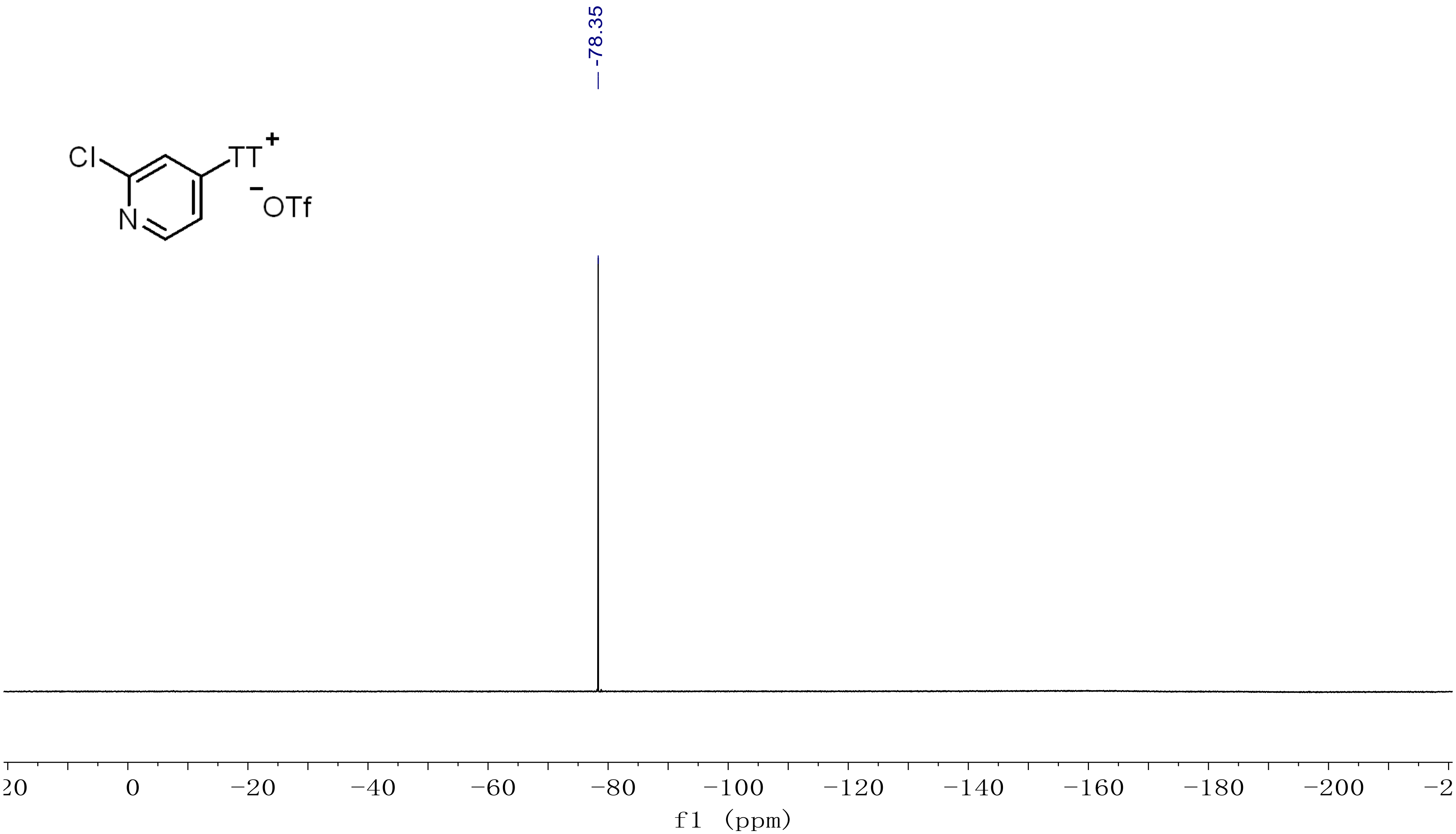

**$^{13}$C NMR of pyridine-derived thianthrenium salt TT-29**

$CDCl_3$, 23 ºC

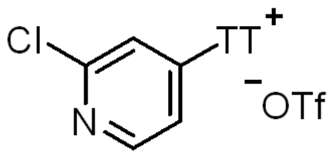


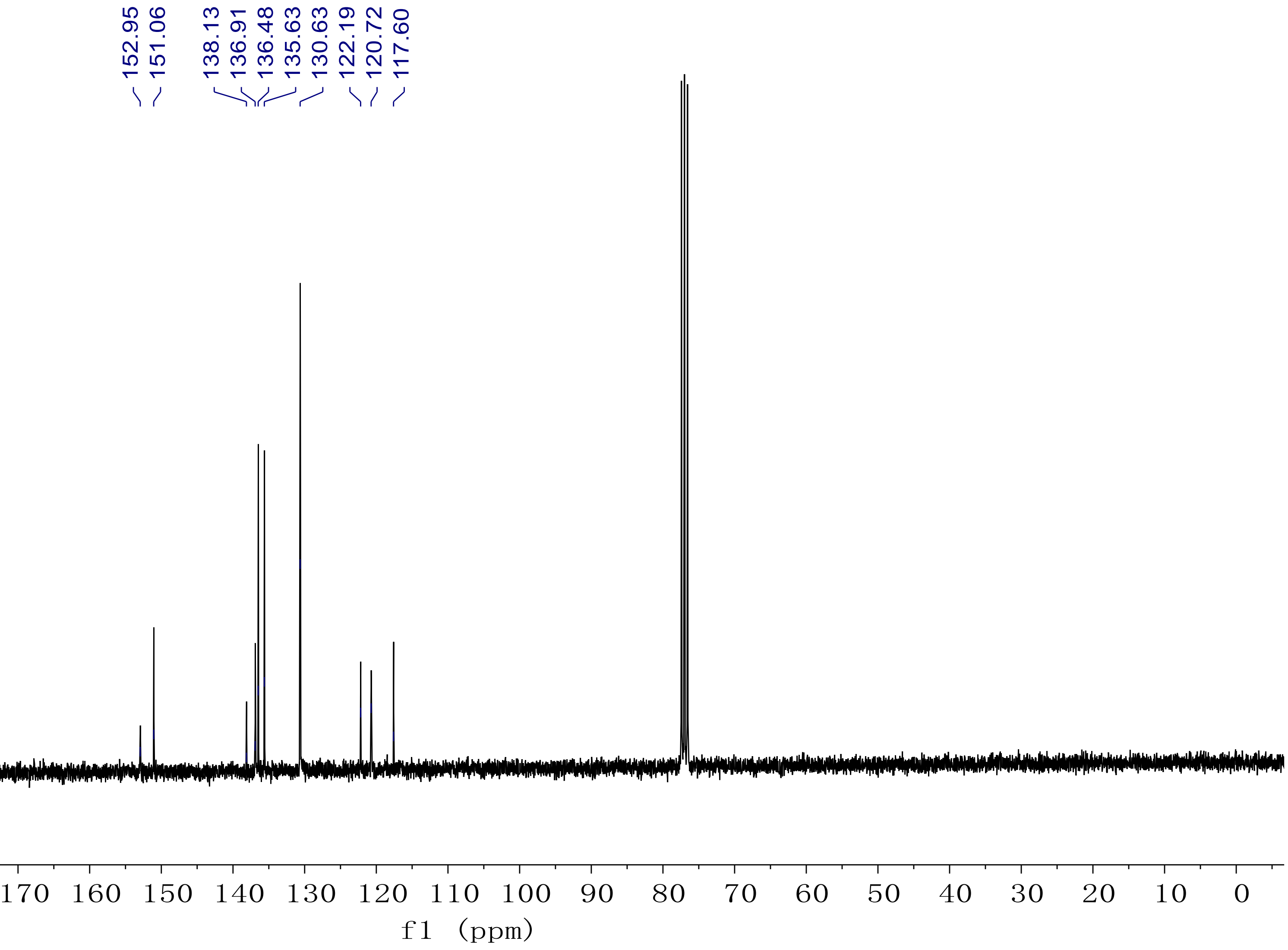

**$^1$H NMR of *rac*-BINAPCu(MeCN)BF$_4$**

$CD_2Cl_2$, 23 ºC

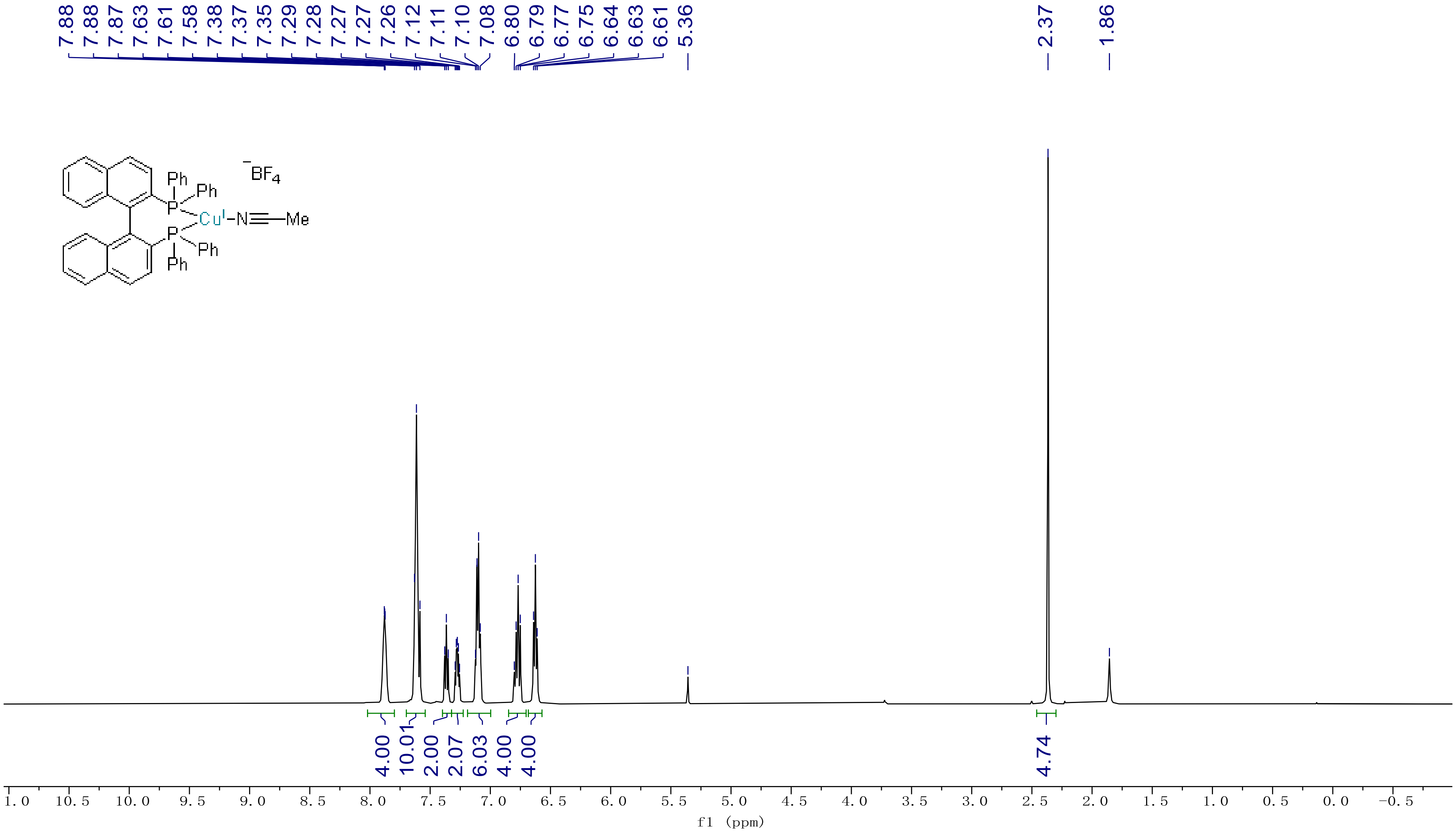

**$^{13}$C NMR of *rac*-BINAPCu(MeCN)BF$_4$**

$CD_2Cl_2$, 23 ºC

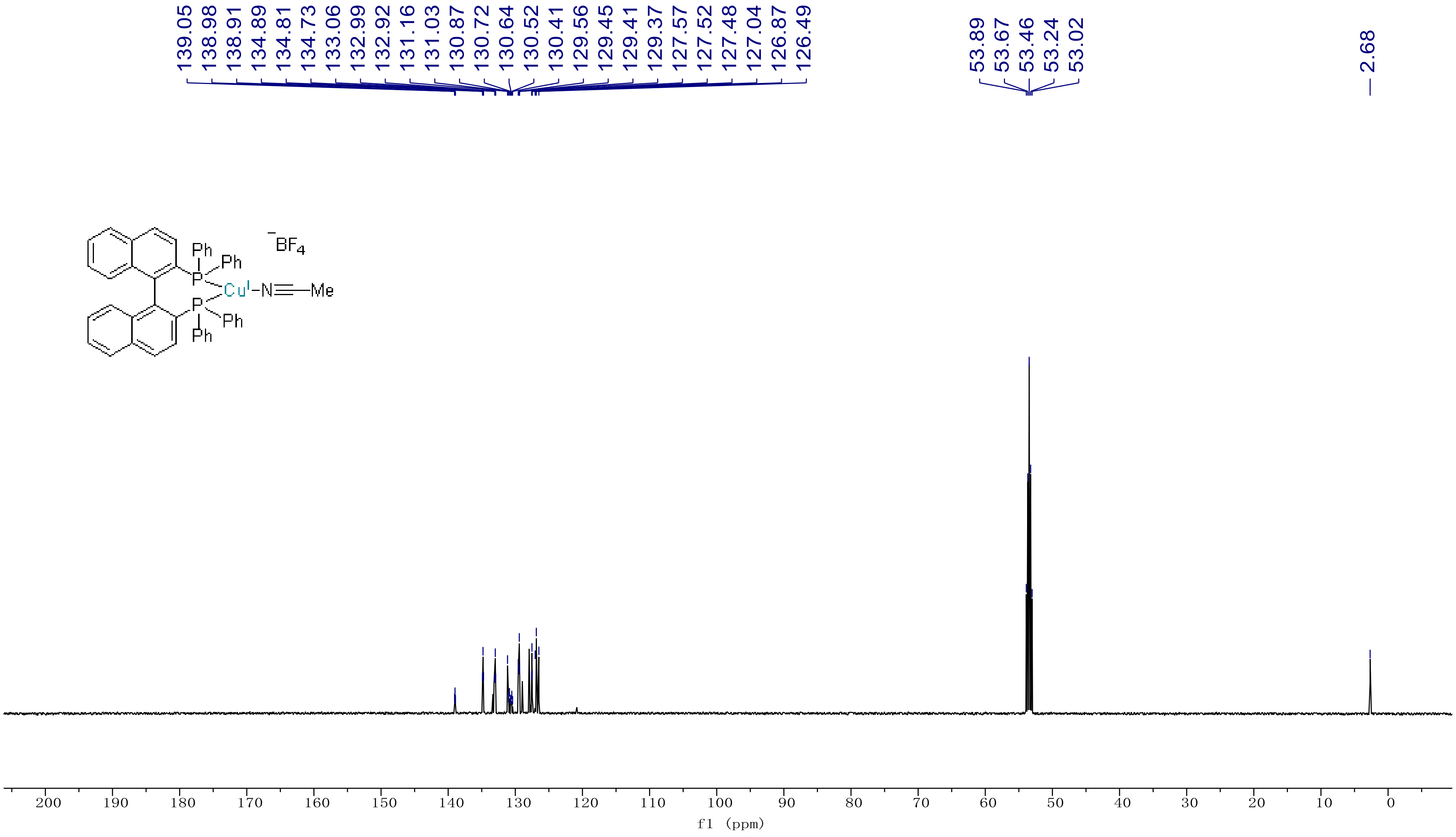

**$^{19}$F NMR of *rac*-BINAPCu(MeCN)$BF_4$**

$CD_2Cl_2$, 23 ºC

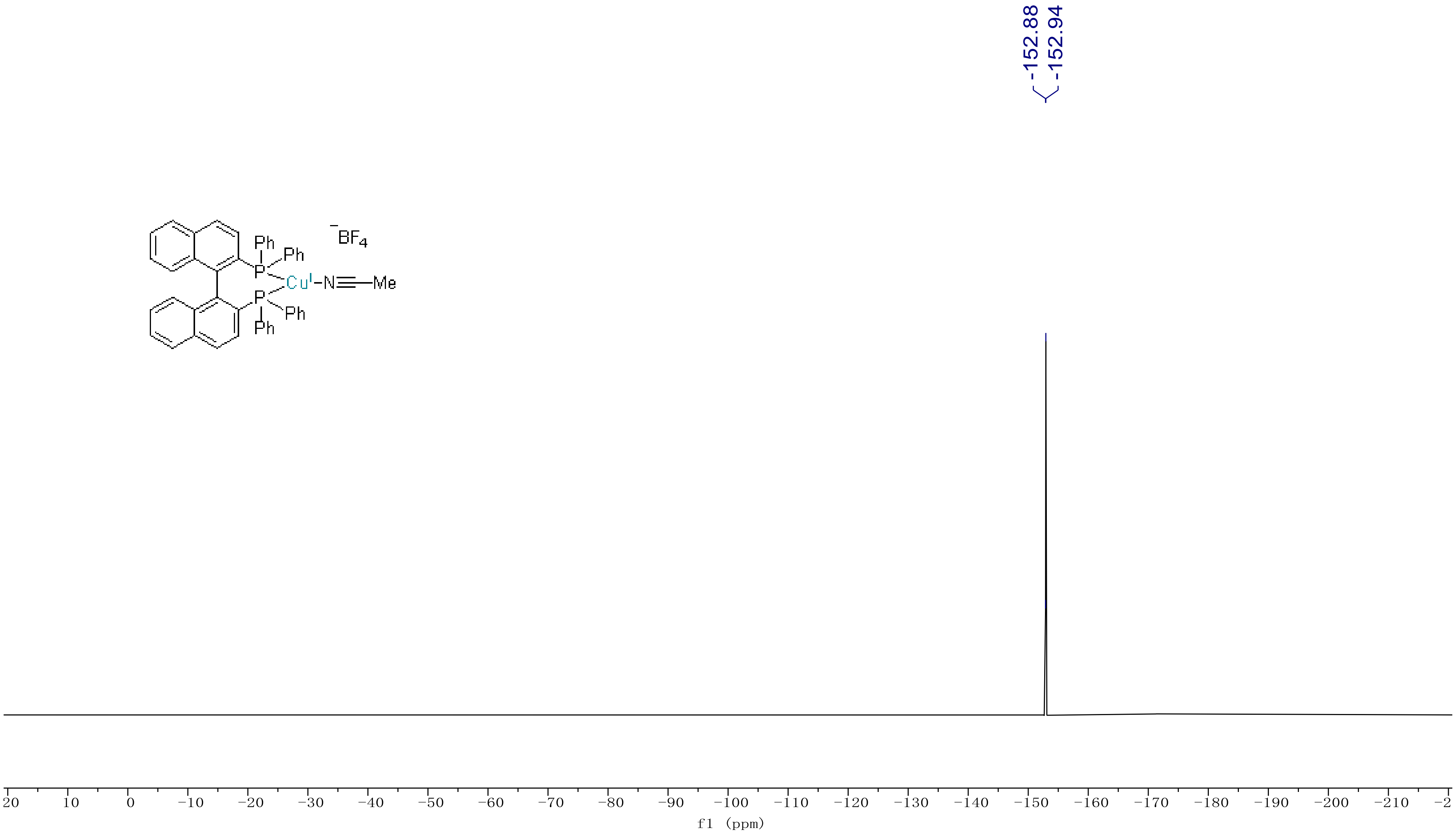

# $^{31}$P NMR of *rac*-BINAPCu(MeCN)$BF_4$

$CD_2Cl_2$, 23 ºC

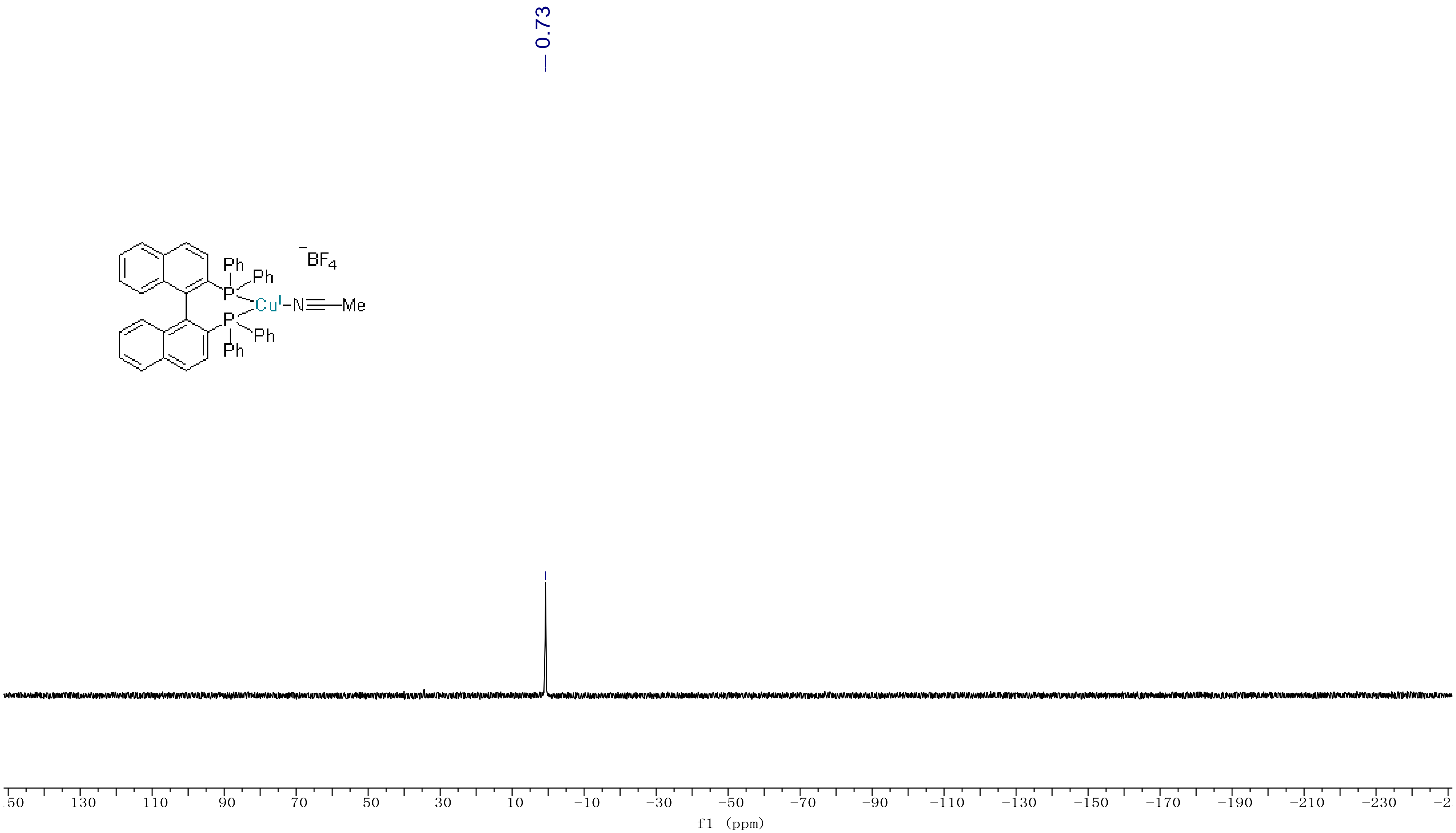

## $^{1}$H NMR of *rac*-BINAPCuN$_3$ (2)

$CD_2Cl_2$, 23 ºC

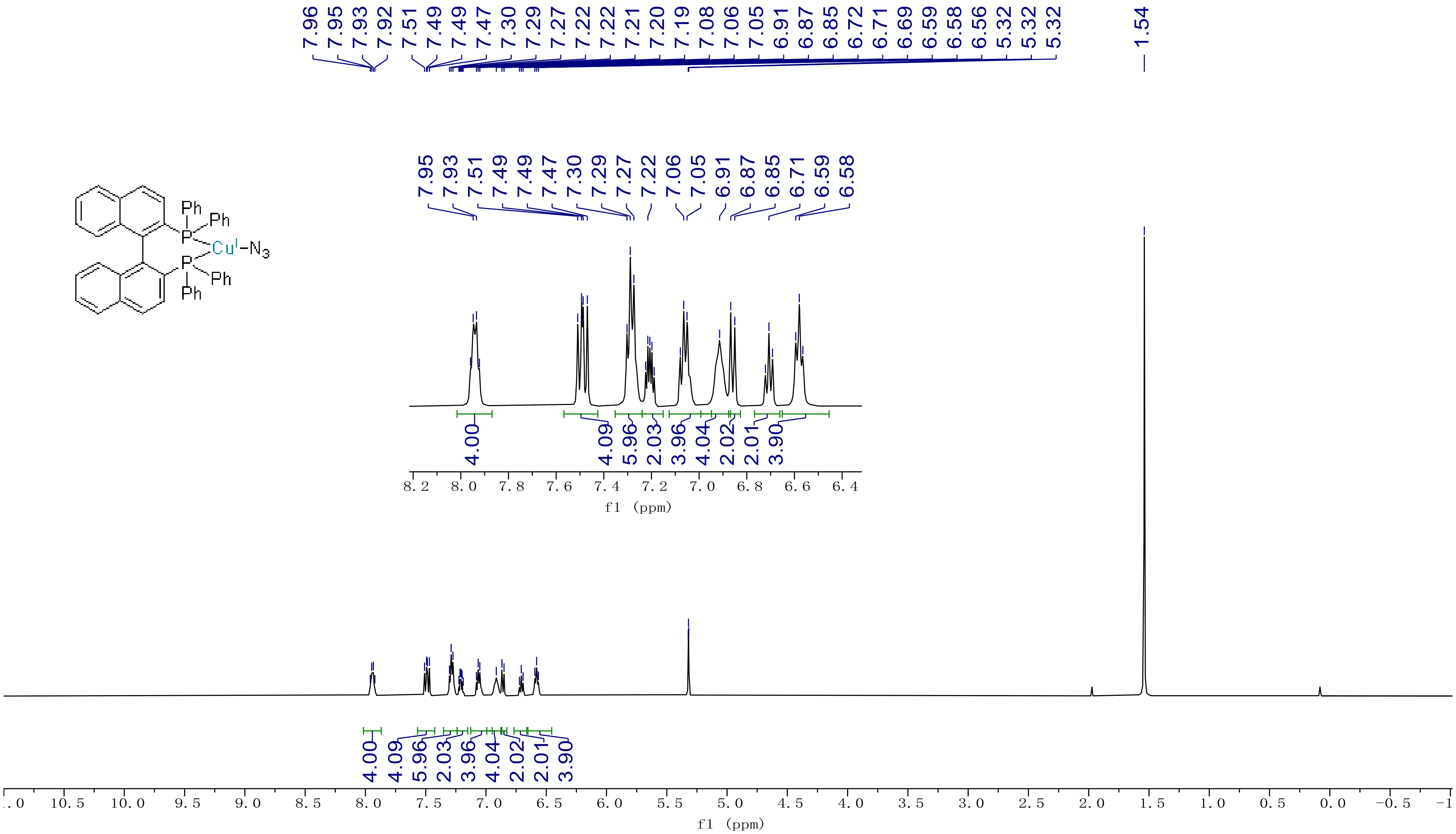

## $^{13}$C NMR of *rac*-BINAPCuN$_3$ (2)

$CD_2Cl_2$, 23 ºC

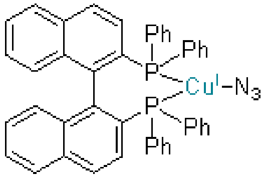


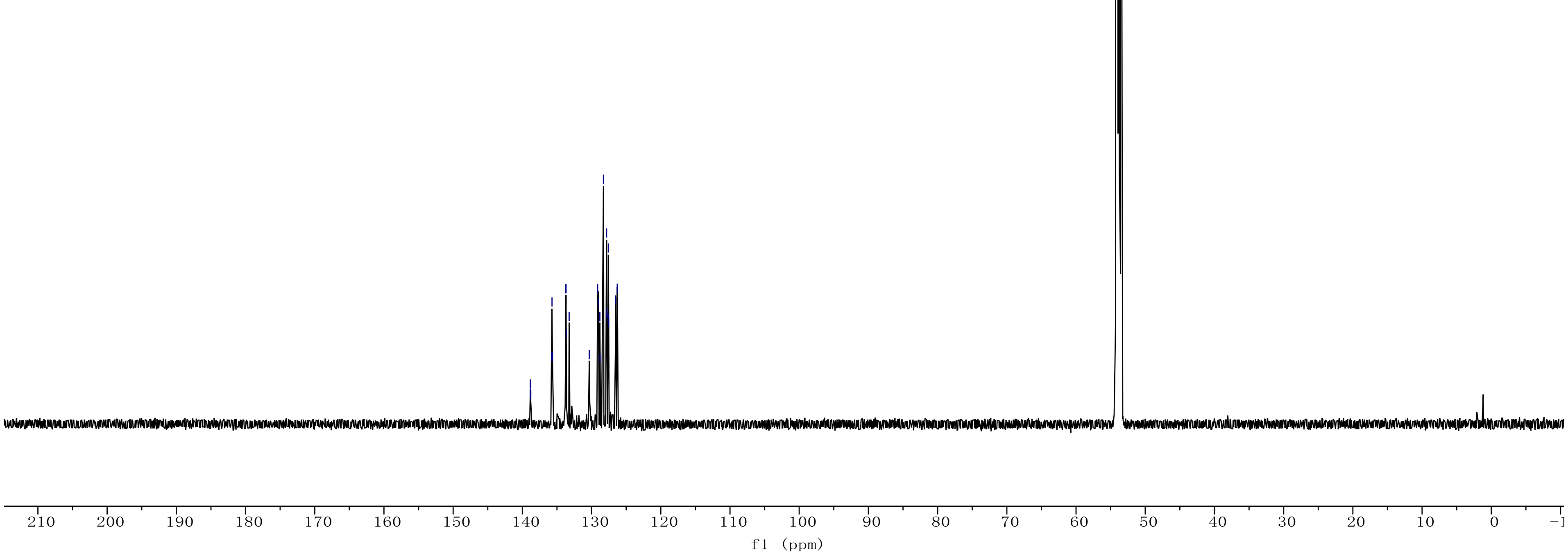

## $^{31}$P NMR of *rac*-BINAPCuN$_3$ (2)

$CD_2Cl_2$, 23 ºC

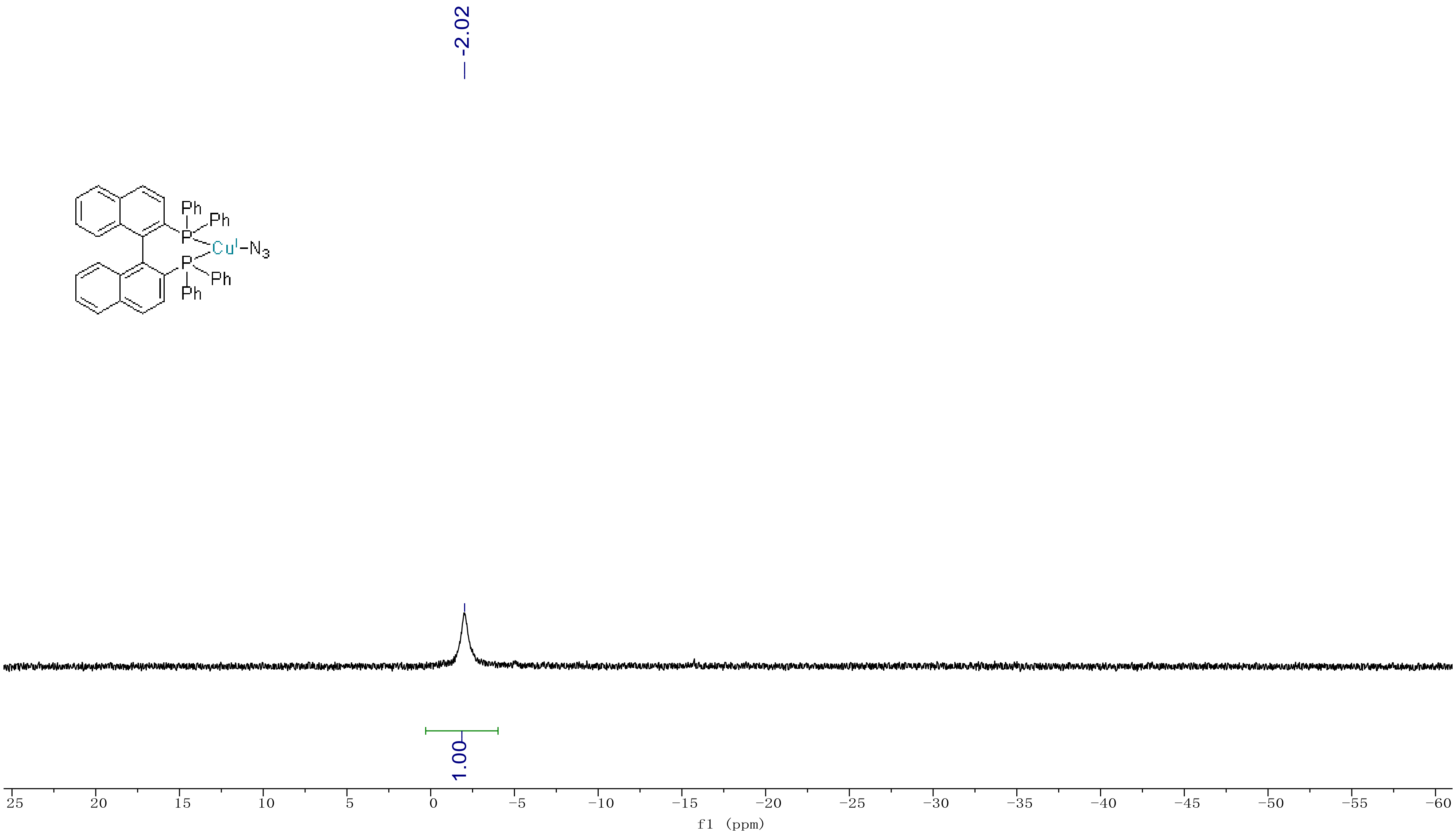

# REFERENCES

1. Fulmer, G. R.; Miller, A. J. M.; Sherden, N. H.; Gottlieb, H. E.; Nudelman, A.; Stoltz, B. M.; Bercaw, J. E.; Goldberg, K. I. *Organometallics*, **2010**, *29*, 2176–2179.
2. Berger, F.; Plutschack, M. B.; Riegger, J.; Yu, W.; Speicher, S.; Ho, M.; Frank, N.; Ritter, T. *Nature*, **2019**, *567*, 223–228.
3. Chen, X-Y.; Wu, Y.; Bai, J-H.; Guo, Y-L.; Wang, P. *ChemRxiv*. **2022**, 10.26434/chemrxiv-2022-4jd98.
4. Chen, C.; Peters, J. C.; Fu, G. C. *Nature* **2021**, *596*, 250–256.
5. Bartra, M.; Romea, P.; Urpí, F.; Vilarrasa, J. *Tetrahedron*. **1990**, *46*, 587–594.
6. Blue, E. D.; Davis, A.; Conner, D.; Gunnoe, T. B.; Boyle, P. D.; White, P. S. *J. Am. Chem. Soc*. **2003**, *125*, 9435−9441.
